# Determinants of access to basic handwashing facilities and handwashing with soap in low-income areas of four Kenyan cities

**DOI:** 10.1371/journal.pgph.0004921

**Published:** 2025-07-17

**Authors:** Sheillah N. Simiyu, Phylis J. Busienei, Nelson Mbaya, Kelly K. Baker, Robert Dreibelbis, Oliver Cumming

**Affiliations:** 1 Population Dynamics and Urbanization, African Population and Health Research Center, Nairobi, Kenya; 2 Data Synergy and Evaluation, African Population and Health Research Center, Nairobi, Kenya; 3 Department of Epidemiology and Environmental Health, University at Buffalo State University of New York, Buffalo, New York, United States of America; 4 Department of Disease Control, London School of Hygiene and Tropical Medicine, London, United Kingdom; UP Manila: University of the Philippines Manila, PHILIPPINES

## Abstract

Handwashing with soap is an effective public health measure against infectious disease and is enabled by availability of handwashing facilities, soap and sufficient water. However, access to handwashing facilities in low-income urban areas is often low, which hinders effective handwashing with soap. We assessed access to basic handwashing facilities and handwashing with soap practices in low-income areas across four cities in Kenya. A cross-sectional survey was conducted and observations made at household level to assess availability of basic handwashing facilities. Respondents demonstrated how they usually washed hands and observations were made on whether hands were washed with soap or not. Multivariable logistic regression models were used to assess determinants of access to basic handwashing facilities and of handwashing with soap across the cities. Results show that most handwashing facilities were basins (77%) and customised containers (4.6%). Less than half of respondents (40%) reported always using soap during handwashing and 59% reported sometimes using soap. Those with secondary education had higher odds of having basic handwashing facilities (Adjusted Odds Ratio (AOR)-1.92, P = 0.02, CI 1.14- 3.24) while those without any compound enclosure had lower odds of having handwashing facilities (AOR = 0.42, P = 0.00, CI 0.28-0.62). Respondents with a handwashing facility (AOR = 69.52, P = 0.00, CI 42.88-112.73) and those with a water point in their compound (AOR 2.4, P = 0.00 CI: 1.43-3.98) had higher odds of handwashing with soap. Across the cities, residents from Mombasa had lower odds of having handwashing facilities (AOR = 0.47, P = 0.01 CI 0.28-0.80) and of handwashing with soap (AOR-0.19; P = 0.00; CI 0.08-0.42) compared to those from Nairobi. These results buttress the important role played by water and the presence of a handwashing facility in promoting handwashing with soap. Interventions in low-income areas should focus on increasing access to conditions such as consistent supply of water to promote adequate and sustained handwashing with soap.

## Introduction

Handwashing with soap is a key public health measure. Recent systematic reviews report that handwashing with soap reduces the risk of diarrhoeal diseases and acute respiratory infections by 30% and 17% respectively [1,2]. The COVID-19 pandemic further highlighted the importance of handwashing with soap in preventing global pandemics [3].

Studies have shown an increase in handwashing with soap when handwashing facilities and soap are available [4,5]. According to the Joint Monitoring Program (JMP) of the World Health Organisation (WHO) and the United Nations Childrens’ Fund (UNICEF), handwashing facilities can be fixed or mobile facilities and include sinks with tap water, buckets with taps, tippy-taps, and jugs or basins designated for handwashing. Washing agents that qualify as soap include bar soap, liquid soap, powder detergent, and soapy water [6]. Access to handwashing facilities is classified into three categories of ‘none’, ‘limited’, and ‘basic’ facilities. Households with ‘basic’ facilities are those with a handwashing facility with soap and water on the premises, while households with handwashing facilities but which lack water and/or soap are classified as ‘limited’ facilities [6]. The JMP estimated that by 2020, 71% of the global population had access to at least a basic handwashing facility [6]. Coverage of basic handwashing facilities is low in Sub-Saharan Africa since 70% of the population still lack basic hygiene services [7].

Generally, there are disparities in access to handwashing facilities between urban and rural areas, with urban areas generally having better access than rural areas [6]. Low-income settlements are common in urban areas of Sub Saharan Africa, as it is estimated that over half of the population in Sub Saharan Africa lives in low-income settlements [8]. Residents in these settlements face various challenges including inadequate water and sanitation services [9]. With these limitations, the estimates by the JMP disaggregated by urban and rural settings alone may not adequately reflect differences in coverage within urban settings, especially in Sub Saharan Africa.

In Kenya, an estimated 33% of the urban population had access to basic handwashing facilities by 2020 [6]. Recent studies have confirmed higher access to handwashing facilities in urban areas, generally highlighting that poor households are less likely to have basic handwashing facilities at the household level [10–12]. In addition, most of the urban population in Kenya lives in low income areas, yet there are limited studies that have focused on access to handwashing facilities or on handwashing with soap in low-income areas [13,14].

The few studies available have revealed a general lack of handwashing facilities at the household level due to factors such as lack of space for stationing handwashing facilities in the household and a lack of water [15–17]. More evidence characterizing the broader set of determinants or factors that explain the practice of handwashing with soap is needed, a knowledge gap that is especially critical given the importance of hand hygiene interventions highlighted during the COVID-19 pandemic. In addition, it is necessary to evaluate the types of handwashing facilities in these low-income areas as defined by the JMP, assess how handwashing with soap is done, and identify determinants of access to basic handwashing facilities and of handwashing with soap. This paper therefore aims to fill this knowledge gap by assessing the determinants of access to basic handwashing facilities and of proper handwashing with soap in selected low-income areas from four cities in Kenya.

## Materials and methods

### Study sites

**Fig 1:**
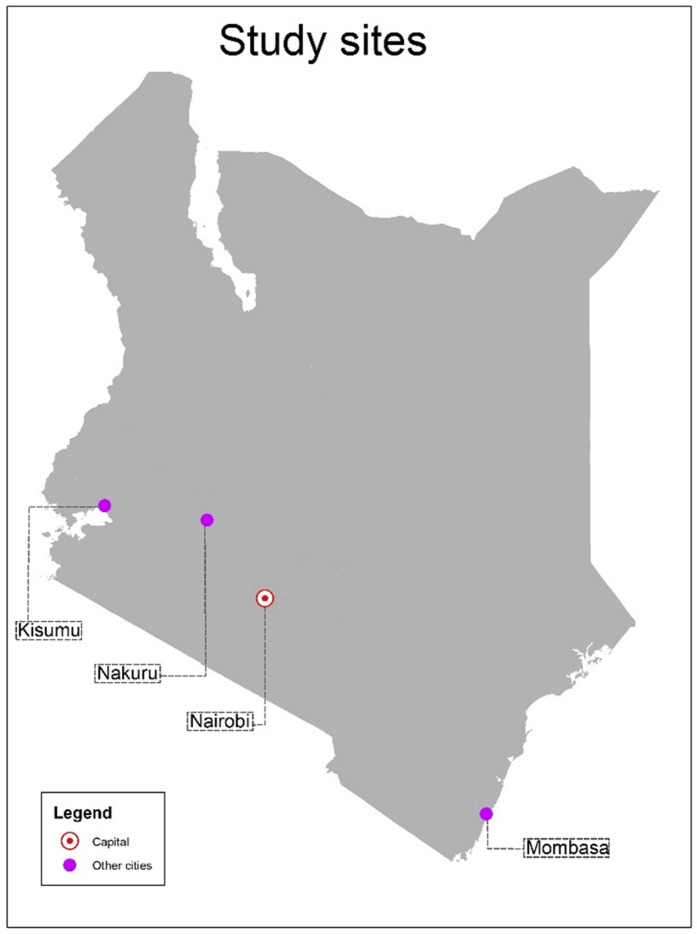
Map of Kenya showing the four study cities. The shape files used to construct the map are from OpenAfrica, covered by Creative Commons Attribution 4.0 International. License and downloadable from https://open.africa/dataset/kenya-counties-shapefile.

Kenya had four main cities by 2022: Nairobi, Kisumu, Mombasa and Nakuru. Nairobi is the capital and largest city, followed by Mombasa, Kisumu and Nakuru. According to the World Bank, more than half of the urban population in Kenya lives in low-income areas(https://data.worldbank.org/indicator/EN.POP.SLUM.UR.ZS). Geographical positions and population characteristics of these cities are summarised in Table 1 and their location in the country are shown in Fig 1 [18–24].

**Table 1:** Characteristics of the four cities in Kenya.

	City (and County)			
	Nairobi	Mombasa	Kisumu	Nakuru
Geographical location in Kenya	South Central	South-East	Western	South-Western
Population (Estimate)	4.4 million	1.4 million	500,000	450,000
% of population in low-income areas	60-70%	65%	60%	50%
Selected Sub County	Westlands	Kisauni	Kisumu Central	Nakuru Town West
Selected low-income area	Kangemi	Junda	Manyatta and Obunga	Kaptembwo and Rhonda
Approx. no of HH in the selected low-income area	16181	2956	Obunga-5554 Manyatta-3760	Kaptembwo-8525 Rhonda-7086

Study sites in each city were selected after consultations with County Government representatives. One sub-county was selected from each of the four Counties, and low-income areas were selected from each of the selected sub-counties. The low-income areas were selected if they did not have any ongoing handwashing interventions from other development partners at the time. The selected low-income study sites were representative of low-income areas in other areas in the country, that are densely populated with unreliable supply of services such as water, sanitation, solid waste management and electricity [25]. Residents live in housing structures that are made of stones, mud or iron sheets and which are organized in plots/compounds. A plot/compound consists of several housing units under one landowner, and services such as water and sanitation are often shared within the plots [26,27]. The settlements are prone to climate extremities of floods and heat; for example, sea level rise due to extreme rainfall may intensify flooding in low-lying areas where the settlements are in Mombasa, leading to contamination and/or salinization of freshwater sources and inadequate supply of water sources [28]. Settlements in Nakuru, Kisumu and Nairobi are prone to flooding during heavy rainfall due to their low-lying nature, proximity to rivers, and poor drainage systems [29–32].

### Study design and sample size

A cross-sectional survey was conducted to estimate the prevalence (P) of basic handwashing facilities in each city. The minimum sample size required in each city to achieve 80% power (Beta = 0.80) and 95% confidence (alpha = 0.05) in estimates was determined by:


$$n=\left(\frac{\left(Z{)}^{2}\times P(q\right)}{(d{)}^{2}}\right)$$

Where Z is the statistic corresponding to the level of confidence, P is the expected prevalence, q is determined as (1-p), and *d* is the precision/margin of error. The precision was set to 5%, The Z-statistic corresponding to the 95% confidence level is set at 1.96, and estimates of prevalence were assumed to be 66% in Kisumu [13], 18.6% in Nakuru [23] 27% in Mombasa [33], and 21.1% in Nairobi [34]. The calculated sample size of 345 respondents in Kisumu, 237 respondents in Nakuru, 303 respondents in Mombasa, and 255 respondents in Nairobi was adjusted upwards by 10% to cater for refusals or dropouts. The final adjusted sample size was 280 respondents in Nairobi, 333 respondents in Mombasa, 379 respondents in Kisumu, and 260 respondents in Nakuru.

These sample sizes were applied in Nairobi and Mombasa as there was one low-income area selected as a study site in each of the two cities (Kangemi in Nairobi and Junda in Mombasa). In Nakuru and Kisumu, where there were two low-income areas selected (Kaptembwo and Rhonda in Nakuru, and Manyatta and Obunga in Kisumu), the estimated sample size was further apportioned proportionately to each of the two low-income areas to determine the number of participants to select from each of the two areas.

### Sampling and selection of participants

Administrative and community leaders in each city and study site were engaged to obtain the necessary approvals and gain community entry. The geographic limits of each study site in each of the four cities was determined with community leaders, and the areas were further divided into smaller administrative units such as villages. Each site had 2–4 villages, and two villages were selected from each site. Leaders from the Department of Health assisted in identifying Community Health Promoters (CHPs) working within the selected villages and one community health promoter was selected from each village. The CHPs provided the approximate number of households within the village, and the total sample size for each site was proportionately apportioned to each study village to ensure representativeness. A transect walk was conducted in each village for the field team to familiarise themselves with the study site, and to ensure that field staff worked within the selected villages.

A systematic sampling approach was applied, and the sampling interval was determined by dividing the total number of households and the required sample size. The first household and the starting point were selected randomly from one corner of the village, and subsequent households selected after the estimated sampling interval. Each field staff worked with one community health promoter. At the household level, respondents were considered eligible for the study if they were adults (at least 18 years), or partners to the household heads, and had lived in their household for at least 3 months. The community health promoters and field staff identified the eligible respondents and introduced themselves and the study before proceeding with the survey questions.

### Data collection

Data collection was conducted between 1st October and 29^th^ November 2022 across the four cities. Field staff who assisted in data collection were selected if they were residents in each of the four cities and could communicate in the common local language, if they had a basic university degree, and if they had previous experience in similar data collection exercises. These staff were trained for 5 days on the data collection aspects of the study. Respondents were interviewed in their preferred language (Dholuo in Kisumu and Swahili in other study sites) using a structured survey tool. The tool contained questions covering household demographics, housing and compound characteristics, access to and types of Water, Sanitation and Hygiene (WASH) facilities, and handwashing practices. To assess and verify the availability of handwashing facilities, field staff used three approaches: they asked the respondents where they washed their hands, observed if there were any handwashing facilities at the household, and asked the respondents to show where and how they normally washed hands. Field staff recorded the types of observed and reported handwashing facilities and soap, and how hands were washed (whether soap was used or not). Only one respondent was selected and interviewed in the residence (whether it was a compound or a stand-alone type of residence). Field staff also made observations and took note of compound level characteristics such as water and sanitation/toilet facilities, type of compound enclosure if any, and the number of households within the compounds (to further verify the information provided by respondents). All the data was collected electronically on tablets through survey CTO (a mobile data collection platform by Dobility Inc., Cambridge Massachusetts and Washington, DC), which was then relayed and stored on a secure server hosted at the African Population and Health Research Center (APHRC). Consistency checks were incorporated into the electronic version of the survey tool, to minimise errors and ensure completeness of the data.

### Data management and analysis

Data was downloaded from the APHRC server to STATA (Statacorp, V.15) for cleaning and analysis. There were two main outcomes of interest: 1) access to/availability of a basic handwashing facility, and 2) observed handwashing practice (whether hands were washed with soap or not).

**Fig 2:**
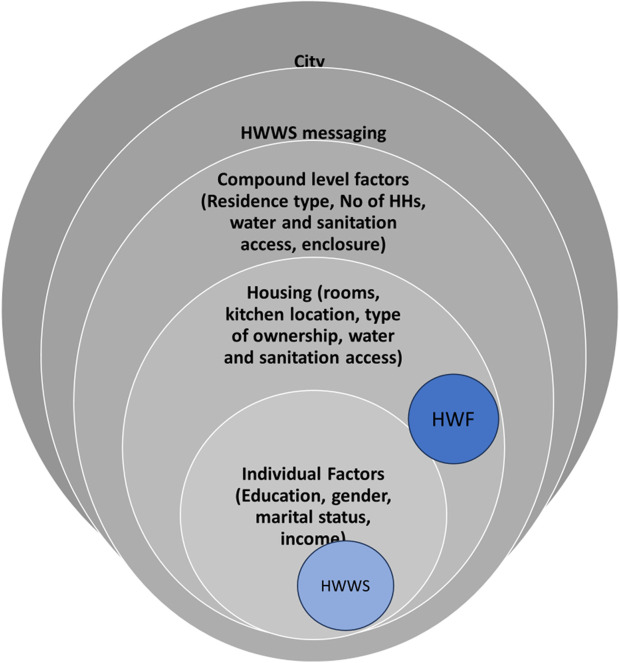
Conceptual model of the relationship between the variables.

A basic handwashing facility was defined based on the JMP definition of a handwashing facility with water and soap. A respondent was classified as having a basic handwashing facility if field staff observed a handwashing facility, water and soap. The second outcome was based on observations when respondents were asked to demonstrate how they normally washed their hands. This outcome was classified as handwashing with soap -if soap was used for washing hands – or not (irrespective of technique used and duration of handwashing). Predictor variables included individual and housing characteristics, compound level characteristics such as compound enclosure, and water and sanitation characteristics such as time taken to water sources, consistency of water sources, and availability of sanitation facilities (See Fig 2 for the conceptual model).

Although the primary aim of the analysis was to assess the effect of predictor variables on the outcome variables, additional analysis also focused on assessing the differences between the cities. Data analysis entailed running descriptive statistics-percentages and means- to provide a summary of the results. Bivariate models were built using Pearson’s chi square test to assess corelation between the predictor variables, associations between the outcome and predictor variables, and differences between cities. Significant associations with each outcome variable in the bivariate analysis were further assessed using univariate logistic regression to estimate the effect of each independent variable on the outcome variables (S1 Table). Predictor variables with significant effects on the outcome variables were then assessed in a multivariable logistic regression model. Two multi-variable logistic regression models were conducted based on the two outcomes of interest. The analysis was adjusted for the effect of confounding factors, including gender, marital status, income, and education level. Adjusted Odds Ratios (AOR) with 95% Confidence Interval (CI) were used to report the effect of predictor variables on the outcomes. Statistical significance was declared at a p-value of ≤ 0.05. The Pearson or Hosmer–Lemeshow goodness-of-fit test was used to determine if the fitted models adequately described the outcomes.

### Ethical approval

The study protocol was approved by the AMREF Health Africa Research and Ethics committee (Protocol number P1212-2022). A study permit was obtained from the National Commission for Science and Technology and Innovation (NACOSTI) (Permit number NACOSTI/P/22/19335). At the County level, permits were obtained from the County Governments in each of the cities and from the administrative leaders in each of the 4 study sites. All participants were provided with full details of the study after which they signed a consent form to show their consent to participate in the study. Participants retained a copy of the information sheet and the signed consent form. To ensure privacy and confidentiality, the respondents were allowed to select locations where they were comfortable, and where other individuals were not present. The respondents were assured that the information would only be used for research purposes, their names were not captured during data collection, that the data would be analysed anonymously, and the results shared through various outputs and to relevant stakeholders.

## Results

### Descriptive summary of the results

A total of 1,347 respondents were interviewed comprising 302 from Nairobi, 292 from Nakuru, 350 from Mombasa and 403 from Kisumu. The sample sizes in each of the cities were increased due to the opportunity to improve the study reliability and generalizability-rather than any methodological concerns- allowing a more robust conclusion.

Descriptive results are presented in Table 2. In summary, up to 42% of the respondents had secondary level of education, and the majority (80%) of the respondents had income levels below KES 20,000. In terms of living conditions, 74% rented the houses they lived in, and 65% of compounds were enclosed by a wall/fence with lockable gates.

**Table 2:** Demographic, housing, compound and WASH characteristics of respondents across the four cities.

	County					
Demographic Characteristics						
	Nairobi N=302	Nakuru N=292	Mombasa N=350	Kisumu N=403	Total N=1347	
	Freq (%)	Freq (%)	Freq (%)	Freq (%)	Freq (%)	
**Age**	31.6 (18-76)	35.5 (18-77)	37.3 (18-72)	36.1 (18-86)	35.2 (18-86) SD 12.9	
	SD 10.8	SD 12.1	SD 12.8	SD 14.4		
**Gender**						
Male	81 (26.8)	37 (12.7)	105 (30)	52 (12.9)	275 (20.4)	
Female	220 (72.8)	255 (87.3)	245 (70)	351 (87.1)	1071 (79.5)	χ2 =52.35 P = 0.00
Prefer not to say	1 (0.3)	0 (0)	0 (0)	0 (0)	1 (0.1)	
**Marital status**						
Single	73 (24.2)	37 (12.7)	70 (20)	70 (17.4)	250 (18.6)	
Married/in partnership.	212 (70.2)	208 (71.2)	212 (60.6)	270 (67)	902 (67)	
Widowed	7 (2.3)	9 (3.1)	34 (9.7)	51 (12.7)	101 (7.5)	χ2= 76.72 P= 0.00
Separated/divorced.	9 (3.0)	33 (11.3)	30 (8.6)	12 (3)	84 (6.2)	
Prefer not to say	1 (0.3)	5 (1.7)	4 (1.1)	0 (0)	10 (0.7)	
**Education status**						
None	2 (0.7)	5 (1.7)	58 (16.6)	21 (5.2)	86 (6.4)	
Primary	86 (28.5)	108 (37)	172 (49.1)	161 (40)	527 (39.1)	
Secondary	175 (57.9)	138 (47.3)	88 (25.1)	163 40.4)	564 (41.9)	χ2=64.42 P=0.00
Higher	38 (12.6)	41 (14)	31 (8.9)	58 (14.4)	168 (12.5)	
Prefer not to say	1 (0.3)	0 (0)	1 (0.3)	0 (0)	2 (0.1)	
Income (KES)*						
Below 10, 000	74 (27.4)	129 (44.9)	126 (39.4)	238 (60.3)	567 (44.6)	
10, 001-20, 000	110 (40.7)	121 (42.2)	141 (44.1)	88 (22.3)	460 (36.2)	
20, 001-30,000	44 (16.3)	21 (7.3)	33 (10.3)	55 (13.9)	153 (12)	χ2=86.21 P= 0.00.
Above 30,001	14 (5.2)	14 (4.9)	13 (4.1)	10 (2.5)	51 (4)	
Prefer not to say	28 (10.4)	2 (0.7)	7 (2.2)	4 (1)	41 (3.2)	
**Household size**	3.7 (1-11)	4.5 (1-17)	5 (1-20)	4.8 (1-14)	4.5 (1-20)	
	SD 1.9	SD 2.0	SD 2.9	SD 2.2	SD 2.3	
**Housing characteristics**						
**Type of residence**						
Room in a multi-unit building with no yard.	75 (24.8)	2 (0.7)	100 (28.6)	2 (0.5)	179 (13.3)	
Freestanding house with/without yard	6 (2)	21 (7.2)	152 (43.4)	41 (10.2)	220 (16.3)	
Compound with family houses	6 (2)	11 (3.7)	12 (3.4)	22 (5.5)	51 (3.8)	
Compound shared with unrelated families.	191 (63.2)	187 (64)	52 (14.9)	182 (45.2)	612 (45.4)	
A block with several houses	24 (7.9)	71 (24.3)	34 (9.7)	156 (38.7)	285 (21.2)	
**Length of stay in the house.**						
Less than 1 year	85 (28.1)	43 (14.7)	33 (9.4)	53 (13.2)	214 (15.9)	
1-2years	57 (22.2)	69 (23.6)	41 (11.7)	74 (18.4)	251 (18.6)	
3-5 years	54 (17.9)	47 (16.1)	42 (12)	72 (17.9)	215 (16)	χ2= 103.20 P= 0.00
Above 5 years	88 (29.1)	126 (43.2)	224 (64)	193 (47.9)	631 (46.8)	
Since birth	8 (2.6)	7 (2.4)	10 (2.9)	11 (2.7)	36 (2.7)	
**Type of house ownership**						
Own	18 (6)	39 (13.4)	190 (54.3)	70 (17.4)	317 (23.5)	
Rent	279 (92.4)	242 (82.9)	149 (42.6)	327 (81.1)	997 (74)	χ2= 271.07 P=0.00
Live for free	5 (1.7)	11 (3.8)	11 (3.1)	6 (1.5)	33 (2.4)	
**Location of the kitchen**						
Inside the house	302 (100)	275 (94.2)	284 (81.1)	391 (97)	1252 (92.9)	
Outside the house	-	17 (5.8)	66 (18.9)	12 (3)	95 (7.1)	χ2= 108.2 P=0.00
**Compound Characteristics**						
Number of households	22.5 (1-120)	21.5 (1-150)	2.9 (1-14)	8.1 (1-53)	12.9 (1-150)	
	SD 18.6	SD 22.5	SD 3.0	SD 6.1	SD 16.4	
**Compound enclosure**						
Wall/fence with lockable gate	279 (92.4)	274 (93.8)	129 (36.9)	187 (46.4)	869 (64.5)	
Wall/fence with unlockable gate	4 (1.3)	13 (4.5)	2 (0.6)	33 (8.2)	52 (3.9)	
Wall/fence, No gate	15 (5.0)	2 (0.7)	15 (4.3)	70 (17.3)	102 (7.5)	
None (no gate, no fence/wall)	4 (1.3)	3 (1)	204 (58.3)	113 (28)	324 (24.1)	
Water, sanitation, and hygiene						
**Main water source**						
Tap inside the house.	16 (5.3)	21 (7.2)	23 (6.6)	16 (4)	76 (5.6)	
Tap in the compound.	178 (58.9)	249 (85.3)	43 (12.3)	85 (21.1)	555 (41.2)	
Public tap or fountain	64 (21.2)	2 (0.7)	215 (61.4)	251 (62.3)	532 (39.5)	
Other improved sources	2 (0.7)	0 (0)	5 (1.4)	20 (5)	27 (2)	
Other unimproved sources	2 (0.7)	2 0.7)	0 (0)	10 (2.5)	14 (1)	
Borehole	2 (0.7)	0 (0)	64 (18.3)	21 (5.2)	87 (6.5)	
Tap in the neighbouring compound	38 (12.6)	18 (6.2)	0 (0)	0 (0)	56 (4.2)	
**Time taken to water source and back.**						
0-5 mins	235 (77.8)	235 (80.5)	182 (52)	328 (81.4)	980 (72.7)	
6-10 min	39 (12.9)	35 (12)	88 (25.1)	52 (12.9)	214 (15.9)	χ2= 110.91 P=0.00
Above 10min	28 (9.3)	22 (7.5)	80 (22.9)	23 (5.7)	153 (11.4)	
**Mode of payment for water**						
Monthly basis	52 (17.2)	113 (38.7)	36 (10.3)	45 (11.2)	246 (18.3)	
Per 20 Litre Jerrican	111 (36.8)	35 (12)	292 (83.4)	347 (86.1)	785 (58.3)	
Included in rent.	137 (45.4)	137 (46.9)	10 (2.9)	8 (2)	292 (21.7)	
Does not pay	2 (0.7)	7 (2.4)	12 (3.4)	3 (0.7)	24 (1.8)	
**Average paid for water (in KES)**						
Monthly	1025 (100-6500)	574 (0-4500)	670 (0-2000)	982 (30-5000)	758 (0-6500)	
Per 20 Litre Jerrican	6.9 (5-30) SD 3.3	6.1 (5-15) SD 2.4	5.3 (0-20) SD2.4	6.3 (0-30) SD 3.6	6.5 (0-30) SD 3.1	
Included in rent.	0	0	0	0	0	
Does not pay	0	0	0	0	0	
**How often water source runs dry.**						
Never	74 (24.5)	51 (17.5)	101 (28.9)	113 (28)	339 (25.1)	χ2=16.21 P= 0.00
Sometimes	147 (48.7)	227 (77.8)	247 (70.6)	288 (71.5)	909 (67.5)	
Often	81 (26.9)	14 (4.8)	2 (0.6)	2 (0.5)	99 (7.4)	
**Handwashing**						
**HWF used for Handwashing (reported)**						
Toilet/Kitchen/sink inside the house	19 (6.3)	20 (6.8)	21 (6)	21 (5.2)	81 (6)	
Basin inside the house	169 (56)	92 (31.5)	167 (47.7)	183 (45.4)	611 (45.4)	
Beside the water source in the yard	3 (1)	11 (3.8)	14 (4)	10 (2.5)	38 (2.8)	
Customised bucket/jerrycan inside the house	5 (1.7)	22 (7.5)	5 (1.4)	21 (5.2)	53 (3.9)	
Customised bucket/jerrycan/container/ Basin outside the house	91 (30.1)	138 (47.2)	50 (14.3)	161 (39.9)	440 (32.7)	
Basin in the compound	14 (4.6)	6 (2.1)	92 (26.3)	5 (1.2)	117 (8.7)	
Other	1 (0.3)	3 (1.0)	1 (0.3)	2 (0.5)	7 (0.5)	
**Where hands are washed (reported)**						
Inside the house	193 (63.9)	135 (46.2)	193 (55.1)	227 (56.3)	748 (55.5)	χ2=18.93 p=0.00
Outside the house	109 (36.1)	157 (53.8)	157 (44.9)	176 (43.7)	599 (44.5)	
**How hands are washed (multiple response)**						
Using soap and water in a basin	246 (81.5)	217 (74.3)	247 (70.6)	341 (84.6)	1051 (78)	
Using soap and running water	60 (19.9)	117 (40.1)	54 (15.4)	103 (25.6)	334 (24.8)	
Dipping hands in a basin of water	62 (20.5)	99 (33.9)	135 (38.6)	57 (14.1)	353 (26.2)	
Washing with running water	35 (11.6)	65 (22.3)	36 (10.3)	31 (7.7)	167 (12.4)	
**Use of soap during handwashing**						
Always	202 (66.2)	79 (27)	106 (30.3)	159 (39.5)	544 (40.4)	χ2=124.30 P= 0.00
Sometimes	96 (31.8)	211 (72.3)	238 (68)	243 (60.3)	788 (58.5)	
No	6 (2)	2 (0.7)	6 (1.7)	1 (0.25)	15 (1.1)	
**Soap used for handwashing.**						
Bar soap, same as what is used for dishes.	63 (21.3)	69 (23.8)	33 (9.5)	210 (52.2)	375 (28.1)	
Bathing soap, same as what is used for bathing.	87 (29.4)	121 (41.8)	13 (3.8)	64 (15.9)	284 (21.3)	
Liquid soap, dedicated only for handwashing.	43 (14.5)	25 (8.6)	182 (52.6)	53 (13.2)	303 (22.7)	
Bar soap, dedicated only for handwashing.	55 (18.6)	35 (12.1)	25 (7.2)	33 (8.2)	148 (11.1)	
Detergent/powder soap	3 (1)	1 (0.3)	77 (22.2)	3 (0.8)	83 (6.2)	
Bar soap for all household uses.	41 (13.9)	36 (12.4)	1 (0.29)	38 (9.5)	113 (8.5)	
Liquid soap also for other uses	2 (0.7)	3 (1.03)	12 (3.5)	1 (0.3)	17 (1.3)	
No soap used.	0 (0)	0 (0)	2 (0.6)	0 (0)	8 (0.6)	
Other (specify)	2 (0.7)	0 (0)	1 (0.3)	0 (0)	3 (0.2)	
**Seen or heard messages on HW with soap.**						
Yes	258 (85.4)	266 (91.1)	90 (25.7)	360 (89.3)	974 (72.3)	χ2 = 515.25 P= 0.00
No	44 (14.6)	26 (8.9)	260 (74.3)	43 (10.7)	373 (27.7)	
**Have sanitation facility**	300 (99.3)	292 (100)	328 (93.7)	394 (97.8)	1314 (97.6)	
**Type of sanitation facility**						
Flush to piped sewer system.	160 (53.3)	82 (28.1)	14 (4.3)	3 (0.8)	259 (19.7)	
Flush to septic tank	30 (10)	67 (22.9)	154 (47)	57 (14.5)	308 (23.4)	
Flush to pit latrine	3 (1)	48 (16.4)	70 (21.3)	32 (8.1)	153 (11.6)	
Flush to don’t know where.	10 (3.3)	7 (2.4)	4 (1.2)	0 (0)	21 (1.6)	
Pit latrine with concrete slab	86 (28.7)	81 (27.7)	73 (22.3)	275 (69.8)	515 (39.2)	
Pit latrine without slab	7 (2.3)	5 (1.7)	8 (2.4)	26 (6.6)	46 (3.5)	
**Location of sanitation facility**						
In the house	20 (6.7)	36 (12.3)	96 (29.3)	18 (4.6)	170 (12.9)	
In compound, not shared.	5 (1.7)	20 (6.8)	103 (31.4)	21 (5.3)	149 (11.3)	
In compound, shared.	236 (78.7)	234 (80.1)	119 (36.3)	324 (82.2)	913 (69.5)	
Neighbouring compound	36 (12)	2 (0.7)	9 (2.7)	19 (4.8)	66 (5)	
Community latrine	3 (1)	0 (0)	0 (0)	10 (2.5)	13 (1)	
Public latrine	0 (0)	0 (0)	1 (0.3)	2 (0.5)	3 (0.2)	
**Sharing sanitation**						
No	10 (3.3)	23 (7.9)	204 (58.3)	30 (7.4)	267 (19.8)	χ2 = 442.68 P= 0.00
Yes	292 (96.7)	269 (96.1)	146 (41.7)	373 (92.6)	1080 (80.2)	
**Relationship of those sharing**						
Close family	193 (63.9)	282 (96.6)	278 (79.4)	276 (68.5)	1029 (76.4)	
Extended family	69 (22.8)	17 (5.8)	46 (13.1)	74 (18.4)	206 (15.3)	
Next door neighbours	208 (68.9)	192 (65.8)	171 (48.9)	322 (79.9)	893 (66.3)	
Well known neighbours not next door	193 (63.9)	149 (51)	50 (14.3)	102 (25.3)	494 (36.7)	
Not well-known neighbours not next door	105 (34.8)	41 (14)	9 (2.6)	55 (13.6)	210 (15.6)	
Friends outside neighbourhood	74 (24.5)	18 (6.2)	4 (1.1)	45 (11.2)	141 (10.5)	
People not known	38 (12.6)	15 (5.1)	13 (3.7)	31 (7.7)	97 (7.2)	
**Summary from observation of handwashing**						
**Observed type of handwashing facility**						
Sink	9 (3)	16 (5.5)	19 (5.4)	18 (4.4)	62 (4.6)	
Bucket fitted with tap.	2 (2)	7 (2.4)	5 (1.4)	17 (4.2)	35 (2.6)	
Jerrycan/container fitted with tap.	10 (3.3)	27 (9.3)	5 (1.4)	20 (5.0)	62 (4.6)	
Happy/ tippy/leaky tin	3 (1)	15 (5.1)	2 (0.6)	17 (4.2)	37 (2.7)	
Basin/Bucket	261 (86.4)	166 (56.8)	295 (84.3)	323 (80.1)	1045 (77.5)	
Compound water point	2 (0.6)	8 (2.7)	13 (3.7)	3 (0.7)	26 (1.9)	
None	11 (3.6)	53 (18.1)	11 (3.1)	5 (1.2)	80 (5.9)	
**Soap observed at HW place.**						
Yes	239 (79.1)	161 (55.1)	185 (52.9)	324 (80.4)	909 (67.7)	χ2 = 103.72 P= 0.00
No	63 (20.9)	131 (44.9)	165 (47.1)	79 (19.6)	438 (32.3)	
**Classification of HWFs** ^**#**^						
None	10 (3.3)	50 (17.1)	8 (2.3)	4 (1)	72 (5.3)	χ2 = 197.12 P= 0.00
Limited	54 (17.9)	84 (28.8)	160 (45.7)	76 (18.9)	374 (27.8)	
Basic	238 (78.8)	158 (54.1)	182 (52.0)	323 (80.1)	901 (66.9)	
**How hands were washed**						
Used only water, washed one hand.	3 (1)	2 (0.7)	8 (2.3)	3 (0.7)	16 (1.2)	
Used only water, washed two hands.	30 (9.9)	52 (17.8)	154 (44)	66 (16.4)	302 (22.4)	
Used soap and water to wash one hand.	0 (0)	16 (5.5)	6 (1.7)	12 (3)	34 (2.5)	
Used soap and water to wash two hands.	266 (88.1)	222 (76)	181 (51.7)	322 (80)	991 (73.6)	
Used soap and ash/sand to wash hands	3 (1)	0 (0)	1 (0.3)	0 (0)	4 (0.3)	
**Hands washed with soap.**						
Yes	266 (88.1)	238 (81.5)	187 (53.4)	334 (82.9)	1025 (76.1)	
No	33 (11.9)	54 (18.5)	163 (46.5)	69 (17.1)	322 (23.9)	χ2 = 137.59 P=0.00

^#^Categorised from the observed types of HWFs and soap

*1 USD=122 KES in November 2022.

Approximately 41% of respondents obtained water from taps located within their compounds and over 70% of water sources were reachable within five mins for a round trip. A quarter of these water sources were available throughout the year (consistent water sources), and the rest were sometimes (68%) or often (7%) running dry (inconsistent water sources). Close to 98% had sanitation facilities (toilets), most (80%) of which were shared with other users.

Respondents washed their hands from a basin or bucket within (45%) or outside their houses (33%). Less than half (40%) of respondents reported that they always used soap during handwashing, and 59% reported that they sometimes used soap. Common types of soap used were bar soap, liquid soap, and powder soap, which were also used for other purposes such as cleaning dishes (28%) and bathing (21%).

From the observation of handwashing facilities and of handwashing with soap, 67% of the respondents had basic handwashing facilities (field staff observed a handwashing facility and soap), 28% had limited handwashing facilities (field staff observed only a handwashing facility or soap), and 5% lacked handwashing facilities. Comparing across the cities, Kisumu had the highest proportion of respondents with basic handwashing facilities (80%), and Mombasa had the least as slightly over half of the respondents had basic handwashing facilities. Overall, 76% of respondents used water and soap to wash hands and 24% used water only. Across the cities, Nairobi had the highest percentage (88%) of respondents using soap during handwashing, while a slightly lower proportion was observed in Mombasa (53%). A summary of these and all other variables is presented in Table 2.

### Determinants of access to basic handwashing facilities

Results of the multivariable logistic regression models are presented in Table 3. These results suggest that secondary level of education increased the odds of having basic handwashing facilities compared to a lack of formal education (AOR-1.92, P = 0.02, CI 1.14- 3.24). Respondents whose water sources sometimes ran dry had 1.7 times higher odds of having basic handwashing facilities (P = 0.00, CI 1.26-2.30), and those whose water sources often ran dry had 2 times higher odds (P = 0.01, CI 1.20-3.90) of having basic handwashing facilities compared to respondents whose water sources never ran dry.

**Table 3:** Binary multivariable logistic regression of predictors of availability of basic handwashing facilities (HWF) and handwashing with soap (HWWS).

Predictors	Model 1: Predictors of availability of basic HHWF			Model 2: Predictors of HWWS	
	P	OR (CI)	P		OR (CI)
**City (ref: Nairobi)**					
Nakuru	0.00	0.32 (0.22-0.49) *	0.46		1.29 (0.65-2.56)
Mombasa	0.01	0.47 (0.28- 0.80) *	0.00		0.19 (0.08-0.42) *
Kisumu	0.07	1.52 (0.96-2.41)	0.08		0.50 (0.22-1.10)
**Education status (ref: none)**					
Primary	0.24	1.35 (0.82- 2.23)			
Secondary	0.01	1.92 (1.14- 3.24) *			
Higher	0.1	1.66 (0.92 -3.01)			
**Housing characteristics**					
**Type of residence** (ref: compound with family houses)					
Room in a multi-unit building with no yard.	0.85	0.92 (0.39-2.14)	0.67		1.41 (0.29-6.77)
Freestanding house with/without yard	0.48	0.75 (0.33-1.68)	0.23		0.40 (0.09-1.81)
Compound shared with unrelated families.	0.11	0.53 (0.24-1.15)	0.23		0.40 (0.09-1.78)
A block with several houses	0.96	1.02 (0.45-2.32)	0.31		0.45 (0.09- 2.10)
**Type of house ownership** (ref: own)					
Rent	0.86	1.04 (0.65-1.68)	0.61		0.83 (0.41- 1.67)
Live for free	0.26	0.61 (0.26-1.44)	0.83		1.18 (0.24-5.70)
**Number of rooms in the house**	0.99	1.00 (0.89 -1.12)	0.21		1.12 (0.94-1.33)
**Location of kitchen (ref: Inside the house)**					
Outside the house	0.5	0.85 (0.52-1.36)	0.02		0.37 (0.16-0.87) *
**Compound**					
**Compound enclosure (ref: Wall/fence with lockable gate)**					
Wall/fence with unlockable gate	0.33	0.69 (0.33-1.46)	0.47		1.50 (0.50-4.50)
No gate/Wall/fence	0.74	1.10 (0.63-1.91)	0.64		0.85 (0.43-1.68)
None (no gate, no fence/wall)	0.00	0.42 (0.28-0.62) *	0.31		0.74 (0.41-1.33)
**Time to water source (ref: 0-5min)**					
6-10 min	0.6	0.91(0.63-1.30)	0.98		1.01 (0.57- 1.78)
Above 10 min	0.22	0.78 (0.52- 1.16)	0.11		0.59 (0.31-1.13)
**Frequency of water payment (ref: Monthly)**			0.71		1.13 (0.60-2.12)
Per 20 Litre Jerrycan			0.84		1.07 (0.57-1.99)
Included in rent			0.21		0.28 (0.04-2.08)
Do not pay					
**How often water runs dry (ref: never)**					
Sometimes	0.00	1.70 (1.26-2.30) *	0.01		2.01(1.21-3.37) *
Often	0.01	2.16 (1.20-3.90) *	0.24		0.61 (0.27-1.40)
Seen/heard HW messages (ref: Y)	0.83	0.96 (0.66-1.39)	0.86		1.05 (0.61-1.79)
Sharing sanitation (ref No)	0.21	0.73 (0.45-1.19)	0.76		1.13 (0.52-2.44)
Water in compound (ref: No)			0.00		2.38 (1.43-3.98) *
Have a basic HWF			0.00		69.52 (42.88-112.73) *
Pseudo R2	0.11				0.51
Wald Chi square	159.07				380.52
No of observations	1347				1347
Log likelihood	762.23				358.82
Hosmer-Lemeshow chi2(8)	6.3				3.55
Prob > chi2	0.61				0.89

Predictors that lowered the odds of having basic handwashing facilities included the city and compound enclosure. Residents from the low-income areas of Nakuru (AOR = 0.32, P = 0.00, CI 0.22-0.49), and those from the low-income areas of Mombasa (AOR = 0.47, P = 0.01, CI 0.28-0.80) had lower odds of having basic handwashing facilities compared to their counterparts from Nairobi. Similarly, compounds without any form of enclosure (such as a wall and/or gate) had lower odds of having basic hand washing facilities compared to compounds with a lockable gate and a fence (AOR = 0.42, P = 0.00, CI 0.28-0.62) (Table 3, model 1).

### Determinants of handwashing with soap

Access to handwashing facilities greatly increased the odds of handwashing with soap (AOR = 69.52, P = 0.00, CI 42.88-112.73). Respondents who had access to a water point in their compounds had 2.4 times higher odds of washing their hands with soap compared to those without a water point in their compounds (P = 0.00 CI: 1.43-3.98). Respondents whose water sources sometimes ran dry had close to 2 times higher odds of washing their hands with soap compared to those whose water sources never ran dry (P = 0.01; CI 1.21-3.37).

Residents from the low-income areas of Mombasa had lower odds of washing their hands with soap compared to residents of low-income areas of Nairobi (AOR-0.19; P = 0.00; CI 0.08-0.42) after adjusting for handwashing facility. Furthermore, respondents whose kitchens were outside the house had lower odds of washing their hands with soap compared to those with kitchens inside the house (AOR = 0.37, P = 0.02 CI 0.16-0.87) (Table 3, model 2).

## Discussion

Our study aimed to investigate the determinants of access to basic handwashing facilities and the practice of handwashing with soap in low-income areas within four cities of Kenya. We sought to investigate individual, housing, compound, water, and sanitation factors which influence access to basic handwashing facilities and to handwashing with soap, as well as differences within the four cities. Basic handwashing facilities were defined according to the JMP definition of a handwashing facility [6], while handwashing with soap was based on observations of how respondents washed hands. Results indicated that the main factors influencing access to basic handwashing facilities were education level, access to water sources, and the compound enclosure. Factors that influenced handwashing with soap included the availability of water and a handwashing facility, and the location of the kitchen. There were differences within the cities in access to handwashing facilities and in handwashing with soap.

The role played by housing and compound conditions in facilitating handwashing with soap were noted in the individual effects of handwashing with soap. Compounds without any form of enclosure were less likely to have handwashing facilities. Upon further scrutiny, it was noted that close to 60% of the respondents without a compound enclosure were from Mombasa, who also generally lacked basic handwashing facilities. Insecurity and theft are common problems in low-income areas, and having no form of enclosure within a residence means that handwashing facilities are at risk of theft or vandalism if placed outside the house/compound [35]. The lack of compound enclosure also highlights the type of compounds within the cities, as well as where handwashing facilities should be located to encourage handwashing with soap. Additionally, those whose kitchens were located outside the house were less likely to wash hands with soap. The location of the kitchen and the sanitation facility are important factors in handwashing as they often determine where handwashing facilities are located, and which could in turn influence handwashing practices. Results from univariate logistic models showed that respondents with shared sanitation facilities were more likely to have handwashing facilities and to wash hands with soap. Although this effect became insignificant in the multivariable logistic model, these results might suggest that when sanitation facilities are shared at the compound, handwashing facilities may also be shared; for example, among those with compound water points as their handwashing facility. Further, the location of the kitchen and the toilet determines where handwashing facilities are likely to be located and consequently, when handwashing is done. Studies have shown that having handwashing facilities near sanitation facilities is a cue for handwashing with soap after toilet use, but not necessarily at other critical handwashing moments such as before cooking or feeding infants [36–38]. Our results therefore support the finding that where handwashing facilities are located in the domestic space influences when hands are washed.

Water and soap are key factors in defining basic handwashing facilities. The important role played by a handwashing facility and water in promoting handwashing has been highlighted in our study and in other studies [4,39–41]. From the results, respondents with inconsistent water sources were likely to have handwashing facilities. Inconsistent water supply is common in low-income areas of African cities, and residents often adapt to the insecurity and inconsistencies of water sources by storing water, borrowing from social networks, reducing water related tasks, purchasing water from informal providers whose costs are usually higher, or using water for multiple uses [42–45]. Therefore, since handwashing facilities were mainly basins and were used within the house or outside the house, it is possible that residents purchased such basins for storage of water, and these basins were also used as handwashing facilities. In addition, the results show that soap was used for multiple purposes other than handwashing. In such circumstances of inconsistent water sources and insufficient quantity, water and soap are likely to be prioritised for other uses as evidently mentioned among the uses of water and soap, and less likely to be primarily used for hygiene including handwashing with soap [46]. On the other hand, respondents with water sources within the compound and those with basic handwashing facilities were more likely to wash hands with soap. When water sources are within the compound, respondents save time to fetch water and are more likely to have enough water that can be used for handwashing. Such practices indicate how respondents adapt to challenges of handwashing with soap-such as lack of resources- that hinder purchasing soap and handwashing facilities only designated for handwashing, small spaces within the house that limit locating handwashing facilities within the household, and perceptions or beliefs about handwashing with soap [17,41,37,47]

Our results indicate that secondary education increased the odds of access to basic handwashing facilities. This finding is consistent with other studies which have confirmed the important role played by education in promoting the uptake of positive handwashing messaging and practices [41,48–50]. Education translates to knowledge of the importance of handwashing facilities, and of positive practices such as handwashing with soap. Having seen or heard handwashing-focused messages was not associated with access to basic handwashing facilities and to handwashing with soap (after adjusting for education); suggesting that messaging on handwashing with soap alone is inadequate without developing foundational thinking skills. Respondents from Mombasa stood out as being less likely to have handwashing facilities and to wash hands with soap, and they had lower education levels and lower access to handwashing messaging. It is likely that the low education levels in Mombasa were a major hindrance to positive handwashing practices as they may have been less knowledgeable about the importance of handwashing facilities or of proper handwashing with soap. These results point to the need to further investigate factors within Mombasa that hinder handwashing with soap. Interventions in such cities therefore need to be cognisant of existing knowledge and practice, they should take advantage of existing social norms, and messaging should be simplified to facilitate uptake [37,46,51].

Our study has strengths and limitations. The strength of the study is that data was collected from low-income areas from four different cities in Kenya, thus providing a diverse snapshot of access to basic handwashing facilities and handwashing practices across the four cities. With regards to limitations, this was a cross-sectional study design, and therefore our results may not be an indication of a cause and effect, especially for handwashing with soap. Our outcome measure of handwashing with soap was observed over a short time and respondents, especially the more educated ones, may have demonstrated their best behaviour because of being observed. Thus, the outcome may reflect their knowledge of handwashing with soap, ability to wash hands, or priority placed on handwashing with soap rather than everyday practice. It is therefore possible, that handwashing with soap, if measured in another study design, may yield different results. We also did not investigate the technique used in handwashing, willingness to purchase handwashing products, differences among vulnerable groups, the duration of handwashing; or the quality of water used for handwashing. These are opportunities and gaps for further qualitative work and study designs with robust observation methods.

## Conclusions and implications

Our study provides findings on the access to basic handwashing facilities and the practice of handwashing with soap in low-income settlements in Kenyan Cities. Different types of mobile handwashing facilities and soap are used in low-income areas to facilitate handwashing with soap. Availability of water is a major determinant and although water sources may be inconsistent, residents of low-income areas may have ways of ensuring availability of water within their households. In these areas, availability of a handwashing facility may not necessarily be a hindrance, but the availability of water and soap to practice handwashing with soap. These findings, therefore, highlights important factors for consideration in practice and policy at national and global levels. Key among these is consideration of where handwashing facilities and soap should be located especially in shared or small spaces, the quantity and quality of water used for handwashing, how handwashing is done especially in contexts where water supply is limited, the use of soap for other purposes for handwashing, how to sustain handwashing with soap, and how to ensure that respondents do not move from basic to limited services due to lack of soap, water, or handwashing facilities [52]. There is need to interrogate local level guidelines on handwashing with soap especially in resource constrained settings such as low-income areas that inform possible interventions. Stakeholders should consider the ‘what’, ‘when’ ‘why’, ‘where’ and ‘how’ when designing sustainable handwashing messaging or interventions, especially given the amount of messaging already relayed during the COVID period. The successful promotion of handwashing with soap requires that the necessary conditions for practising handwashing with soap - that is availability of water and soap - are met.

## Supporting information

S1 Table Appendix 1. (DOCX)

S1 Data Data. (PDF)
