# Supplementary material for: Determinants of access to basic handwashing facilities and handwashing with soap in low-income areas of four Kenyan cities
Source: PLOS Glob Public Health. 2025 Jul 17;5(7):e0004921. doi: 10.1371/journal.pgph.0004921 (PMC12270180; doi:10.1371/journal.pgph.0004921)
Supplement: S1 Table — (DOCX) [file pgph.0004921.s001.docx]

**Appendix 1: Individual effects of predictors on availability of basic Handwashing facilities (HWF) and handwashing with soap (HWWS)**

| **Predictors** | **Independent effect on availability of basic HWF** | | **Independent effect on HWWS** | |
| --- | --- | --- | --- | --- |
|  | P | OR(CI) | P (CI) | OR (CI) |
| **City (ref: Nairobi)**  Nakuru  Mombasa  Kisumu | 0.00  0.00  0.66 | 0.31 (0.22- 0.44) *  0.29 (0.20-0.41) *  1.06 (0.75- 1.57) | 0.03  0.00  0.06 | 0.60 (0.38-0.94) *  0.15 (0.10- 0.23) *  0.55 (0.42- 1.01) |
| **Gender (ref: male)**  Female | 0.01 | 1.43 (1.10-1.89) * | 0.01 | 1.65 (1.23-2.22) * |
| **Marital status (ref: single)**  Married/in partnership.  Widowed  Separated/divorced.  Prefer not to say | 0.43  0.71  0.01  0.10 | 1.13 (0.84- 1.52)  0.91 (0.56-1.48)  0.53 (0.32- 0.88) *  0.34 (0.09-1.22) | 0.30  0.26  0.08  0.29 | 1.19 (0.86-1.65)  0.74 (0.45-1.25)  0.62 (0.37-1.06)  0.49 (0.13-1.81) |
| **Education status (ref: none)**  Primary  Secondary  Higher | 0.01  0.00  0.00 | -  1.85 (1.71-2.93) *  2.95 (1.86-4.68) *  2.52 (1.47- 4.30) * | 0.00  0.00  0.00 | 2.54 (1.60-4.03) *  4.87 (3.03- 7.85) *  3.94 (2.24-6.94) * |
| **Income (KES) (ref** Below 10, 000)  10, 001-20, 000  20, 001-30,000  Above 30,001  Prefer not to answer | 0.89  0.02  0.74  0.09 | 1.02 (0.79- 1.33)  1.62 (1.08-2.43) *  0.90 (0.50-1.63)  1.91 (0.89-4.08) |  |  |
| **Housing characteristics** |  |  |  |  |
| **Type of residence (ref: Compound with family houses)**  Room in a multi-unit building with no yard.  Freestanding house with/without yard  Compound shared with unrelated families.  A block with several houses | 0.28  0.02  0.18  0.68 | 0.68 (0.33-1.37)  0.45 (0.22-0.89) *  0.64 (0.33-1.22)  1.15 (0.58-2.30) | 0.21  0.00  0.38  0.66 | 0.59 (0.26-1.35)  0.26 (0.12-0.59) *  0.70 (0.32- 1.53)  0.83 (0.37-1.88) |
| **Length of stay in house (ref: less than 1 yr)**  1-2years  3-5 years  Above 5 years  Since birth | 0.15  0.85  0.05  0.10 | 0.75 (0.51-1.11)  0.96 (0.63-1.46)  0.71 (0.51-1.00)  0.54 (0.26-1.12) | 0.88  0.25  0.04  0.18 | 0.95 (0.61 -1.48)  1.37 (0.84- 2.23)  0.69 (0.47 -1.00) *  0.60 (0.27- 1.32) |
| **Type of house ownership (ref: own)**  Rent  Live for free | 0.00  0.62 | 1.61 (1.24-2.09) *  0.83 (0.40-1.71) | 0.00  0.54 | 2.22 (1.68-2.93) *  1.27 (0.58- 2.77) |
| **Number of rooms** | 0.01 | 0.90 (0.83-0.98) * | 0.01 | 0.90 (0.82-0.97) * |
| **Location of kitchen** (ref: Inside the house)  Outside the house | 0.00 | 0.55 (0.36 - 0.84) * | 0.00 | 0.31 (0.21-0.48) * |
| **Compound** |  |  |  |  |
| **Compound enclosure (ref:** Wall/fence with lockable gate)  Wall/fence with unlockable gate  No gate/Wall/fence  None (no gate, no fence/wall) | 0.43  0.04  0.00 | 0.79 (0.44-1.42)  1.71 (1.03-2.85) *  0.47 (0.36- 0.61) * | 0.46  0.90  0.00 | 0.77 (0.39 -1.54)  0.97 (0.56- 1.65)  0.26 (0.19-0.34) * |
| **Water and sanitation characteristics** |  |  |  |  |
| **Time to water source (ref:0-5min)**  6-10 min  Above 10min | 0.11  0.00 | 0.78 (0.57-1.05) *  0.59 (0.41-0.83) * | 0.00  0.00 | 0.60 (0.43-0.84) *  0.36 (0.25-0.51) * |
| **Frequency of water payment (ref: monthly)**  Per 20 Litre Jerrycan  Included in rent.  Does not pay | 0.59  0.22  0.80 | 0.92 (0.67-1.25)  0.80 (0.55-1.14)  0.89 (0.37-2.18) | 0.00  0.46  0.01 | 0.46 (0.31-0.67) *  0.84 (0.53 1.33)  0.30 (0.12-0.74) * |
| **How often water runs dry (ref: never)**  Sometimes  Often | 0.00  0.00 | 1.72 (1.33-2.23) *  2.50 (1.50-4.18) * | 0.00  0.01 | 2.14 (1.63- 2.83) *  2.44 (1.25- 3.66) * |
| Seen/heard HW messages (ref: Y) | 0.00 | 0.57 (0.45-0.73) * | 0.00 | 0.40 (0.31-0.53) * |
| Access to sanitation (ref: Yes) | 0.06 | 0.52 (0.26-1.03) | 0.00 | 0.36 (0.18- 0.73) * |
| Sharing sanitation (ref: No) | 0.00 | 1.89 (1.43-2.48) * | 0.00 | 3.06 (2.30- 4.07) * |
| Water in compound (ref: Yes) | 0.23 | 0.87 (0.69-1.09) | 0.00 | 0.38 (0.29-0.50) * |
| Basic handwashing facility (Ref: No) |  |  | 0.00 | 42.95 (29.21-63.16) * |
| **Observed type of HWF (ref: None)**  Sink  Bucket fitted with tap.  Jerrycan/container fitted with tap.  Happy/ tippy/leaky tin  Basin/Bucket  Compound water point |  |  | 0.55  0.07  0.55  0.40  0.26  0.74 | 1.3(0.54-3.10)  4.12(0.89-19.03)  0.78(0.35-1.74)  1.6(0.54-4.76)  0.72(0.41-1.27)  0.83(0.29-2.41) |
