# Supplementary material for: Determinants of access to basic handwashing facilities and handwashing with soap in low-income areas of four Kenyan cities
Source: PLOS Glob Public Health. 2025 Jul 17;5(7):e0004921. doi: 10.1371/journal.pgph.0004921 (PMC12270180; doi:10.1371/journal.pgph.0004921)
Supplement: S1 Data — (PDF) [file pgph.0004921.s002.pdf]

county	gender	marital	q4	q5	q6a	q6b	q7a	q7b
Mombasa	Male	Separated/	Secondary	2	Business		Self-employment	
Kisumu	Female	Married/Pa	Primary	8	Business		Self-employment	
Mombasa	Male	Single	Primary	8	None		None	
Mombasa	Male	Separated/	None		Casual Worker		Casual work	
Mombasa	Male	Married/Pa	Secondary	4	Casual Worker		Casual work	
Nairobi	Female	Married/Pa	Secondary	1	Business		Employment (private s	
Mombasa	Female	Widowed	Secondary	4	None		Family Sup	Remmittan
Mombasa	Male	Married/Pa	Secondary	4	Business		Self-employment	
Mombasa	Male	Married/Pa	Primary	8	Casual Worker		Self-employment	
Nakuru	Female	Married/Pa	Secondary	4	Business		Self-employment	
Nairobi	Male	Married/Pa	Primary	8	Janitorial/guard		Employment (private s	
Kisumu	Female	Married/Pa	Primary	8	None		Self-employment	
Mombasa	Male	Single	Higher	2	None		Employment (private s	
Nakuru	Male	Married/Pa	Primary	8	Casual Worker		Self-employment	
Kisumu	Female	Single	Secondary	4	Business		Self-employment	
Nairobi	Male	Married/Pa	None		Casual Worker		Employment (private s	
Nairobi	Female	Married/Pa	Secondary	2	Casual Worker		Casual work	
Mombasa	Female	Single	Secondary	4	None		Casual work	
Nairobi	Female	Married/Pa	Secondary	2	Business		Employment (private s	
Mombasa	Male	Single	Primary	8	Casual Worker		Casual work	
Kisumu	Female	Single	Primary	8	Business		Self-employment	
Nakuru	Male	Married/Pa	Primary	7	Business		Self-employment	
Kisumu	Male	Married/Pa	Secondary	4	Business		Self-employment	
Mombasa	Female	Separated/	None		None		Casual work	
Kisumu	Female	Married/Pa	Higher	4	Professional		Self-employment	
Kisumu	Female	Married/Pa	Primary	3	Business		Self-employment	
Mombasa	Female	Widowed	Primary	7	Janitorial/guard		Employment (private s	
Nairobi	Male	Married/Pa	Higher	2	Business		Self-employment	
Kisumu	Female	Married/Pa	Higher	1.5	Professional		Employment (private s	
Mombasa	Male	Married/Pa	Primary	8	Casual Worker		Casual work	
Nakuru	Female	Married/Pa	Primary	7	Business		Self-employment	
Mombasa	Female	Married/Pa	None		None		Self-employment	
Nakuru	Female	Widowed	Higher	2	Casual Worker		Casual work	
Mombasa	Male	Single	None		None		Casual work	
Mombasa	Male	Single	Secondary	4	Casual Worker		Casual work	
Mombasa	Female	Married/Pa	None		None		None	
Nakuru	Female	Widowed	Primary	4	Business		Self-employment	
Mombasa	Female	Married/Pa	Primary	8	None		Casual work	
Kisumu	Female	Married/Pa	Primary	8	Business		Self-employment	
Mombasa	Female	Married/Pa	Higher	6	None		Self-employment	
Nakuru	Female	Widowed	None		None		Casual work	
Mombasa	Female	Married/Pa	Primary	8	None		Casual work	
Mombasa	Male	Married/Pa	Primary	7	None		Self-employment	
Mombasa	Female	Widowed	Secondary	2	Casual Worker		Self-employment	
Nakuru	Female	Separated/	Secondary	4	Business		Self-employment	
Nakuru	Female	Married/Pa	Higher	2	Business		Self-employment	
Nairobi	Female	Married/Pa	Secondary	4	Business		Self-employment	
Mombasa	Female	Separated/	None		None		Self-employment	

Mombasa	Male	Widowed	Primary	7	Business	Self-employment
Nakuru	Female	Married/Pa	None		Casual Worker	Casual work
Mombasa	Male	Widowed	None		None	Self-employment
Mombasa	Female	Married/Pa	Primary	8	Business	Self-employment
Mombasa	Female	Married/Pa	Primary	8	None	Casual work
Nairobi	Prefer not	Single	Secondary	4	None	Casual work
Mombasa	Female	Separated/	Primary	3	Business	Self-employment
Mombasa	Male	Married/Pa	Primary	8	Casual Worker	Casual work
Mombasa	Male	Single	Secondary	4	Professional	Employment (private s
Nakuru	Female	Single	Higher	1	None	None
Mombasa	Female	Widowed	Primary	8	Business	Self-employment
Mombasa	Female	Married/Pa	Primary	8	Business	Self-employment
Kisumu	Female	Married/Pa	Primary	8	Business	Self-employment
Mombasa	Female	Separated/	Primary	7	None	Family Sup Remmittan
Mombasa	Male	Married/Pa	Primary	6	Casual Worker	Casual work
Kisumu	Female	Single	Higher	2	Professional	Employment (civil serv
Nakuru	Female	Separated/	Secondary	4	Business	Employment (private s
Nairobi	Female	Single	Secondary	4	None	Employment (private s
Nakuru	Female	Married/Pa	Secondary	4	None	Employment (private s
Nakuru	Female	Widowed	Primary	7	Business	Self-employment
Kisumu	Female	Single	Higher	2	Student	Self-employment
Nakuru	Female	Married/Pa	Secondary	1	None	None
Nairobi	Female	Separated/	Primary	6	Casual Worker	Casual work
Kisumu	Female	Single	Secondary	4	Student	Self-employment
Nairobi	Male	Single	Primary	8	None	None
Mombasa	Female	Married/Pa	Primary	7	None	Casual work
Mombasa	Female	Married/Pa	Primary	8	Casual Worker	Casual work
Kisumu	Female	Married/Pa	Higher	2	None	Self-employment
Mombasa	Female	Separated/	Primary	7	Casual Worker	Self-employment
Mombasa	Female	Married/Pa	None		Casual Worker	Employment (private s
Mombasa	Female	Widowed	Primary	6	Casual Worker	Casual work
Mombasa	Female	Married/Pa	None		Casual Worker	Casual work
Mombasa	Female	Single	Secondary	2	None	None
Kisumu	Female	Widowed	Primary	8	None	Self-employment
Mombasa	Female	Married/Pa	Secondary	2	Business	Self-employment
Kisumu	Female	Single	Secondary	4	Business	Self-employment
Mombasa	Female	Married/Pa	Primary	8	None	Employment (private s
Mombasa	Male	Single	Secondary	4	Casual Worker	Casual work
Kisumu	Female	Married/Pa	Higher	4	Professional	Self-employment
Mombasa	Male	Separated/	Primary	7	Casual Worker	Employment (private s
Nairobi	Female	Married/Pa	Higher	3	Janitorial/guard	Employment (private s
Kisumu	Female	Single	Secondary	4	None	Self-employment
Mombasa	Male	Married/Pa	None		Casual Worker	Self-employment
Mombasa	Female	Married/Pa	Primary	5	Casual Worker	Self-employment
Mombasa	Female	Married/Pa	Primary	6	None	Casual work
Mombasa	Female	Single	Secondary	4	Professional	Self-employment
Mombasa	Female	Married/Pa	Primary	8	Business	Casual work
Mombasa	Female	Separated/	Secondary	1	Casual Worker	Casual work
Kisumu	Female	Married/Pa	Secondary	2	None	Casual work

Kisumu	Female	Married/Pa	Higher	2	Business		Self-employment	
Mombasa	Male	Single	Secondary	4	Casual Worker		Casual work	
Kisumu	Female	Widowed	Primary	7	Casual Worker		Casual work	
Mombasa	Female	Married/Pa	Primary	8	Casual Worker		Employment (private s	
Kisumu	Female	Married/Pa	Primary	8	Casual Worker		Casual work	
Mombasa	Male	Single	Secondary	4	None		Employment (private s	
Kisumu	Female	Single	Higher	3	Student		Self-employment	
Mombasa	Female	Single	Secondary	4	None		Employment (private s	
Mombasa	Male	Single	Secondary	4	Casual Worker		Self-employment	
Mombasa	Female	Prefer not t	None		Casual Worker		Casual work	
Kisumu	Female	Single	Primary	7	Business		Self-employment	
Nairobi	Female	Single	Higher	2	Other	Land Owne	Self-employment	
Nakuru	Female	Married/Pa	Primary	7	Business		Self-employment	
Mombasa	Male	Married/Pa	Primary	7	Casual Worker		Casual work	
Kisumu	Female	Married/Pa	Primary	8	Business		Self-employment	
Mombasa	Male	Separated/	Secondary	6	None		None	
Kisumu	Female	Married/Pa	Secondary	3	Business		Self-employment	
Mombasa	Female	Married/Pa	Primary	8	Casual Worker		Self-employment	
Kisumu	Female	Married/Pa	Higher	4	Professional		Employment (civil serv	
Mombasa	Female	Married/Pa	Secondary	4	Business		Employment (private s	
Mombasa	Male	Married/Pa	Secondary	1	Casual Worker		Self-employment	
Mombasa	Female	Married/Pa	Primary	8	None		Casual work	
Mombasa	Male	Separated/	Higher	4	Casual Worker		Casual work	
Mombasa	Female	Married/Pa	None		Business		Self-employment	
Mombasa	Female	Married/Pa	Primary	8	None		Self-employment	
Kisumu	Female	Married/Pa	Primary	7	Business		Self-employment	
Mombasa	Male	Married/Pa	Primary	5	None		Casual work	
Kisumu	Female	Widowed	None		None		None	
Nakuru	Male	Prefer not t	Secondary	3	Business		Self-employment	
Nakuru	Female	Married/Pa	Primary	8	None		Casual work	
Mombasa	Female	Married/Pa	Primary	8	Business		Casual work	
Nakuru	Female	Married/Pa	Secondary	4	Business		Self-employment	
Mombasa	Female	Separated/	None		None		Casual wor	Landlady
Nakuru	Female	Married/Pa	Primary	7	Business		Employment (private s	
Mombasa	Female	Widowed	Primary	8	None		Casual work	
Nakuru	Female	Married/Pa	Secondary	2	Business		Self-employment	
Kisumu	Female	Married/Pa	Primary	6	Business		Self-employment	
Kisumu	Female	Married/Pa	Primary	7	Business		Self-employment	
Mombasa	Female	Married/Pa	None		Business		Employment (private s	
Mombasa	Female	Married/Pa	Secondary	2	None		Self-employment	
Mombasa	Male	Married/Pa	Primary	8	Casual Worker		Casual work	
Mombasa	Female	Married/Pa	Primary	3	Other	Pastor	Casual wor	Pastor
Nairobi	Female	Married/Pa	Secondary	2	None		Employment (private s	
Mombasa	Female	Single	Secondary	4	None		Casual work	
Kisumu	Female	Married/Pa	Primary	8	Business		Self-employment	
Mombasa	Female	Married/Pa	None		Other	Mama mta	Self-employment	
Mombasa	Male	Married/Pa	Primary	6	Casual Worker		Casual work	
Mombasa	Male	Married/Pa	Secondary	4	Casual Worker		Casual work	
Nakuru	Female	Married/Pa	Secondary	4	Business		Self-employment	

Mombasa	Male	Married/Pa	None		Casual Worker	Casual work
Mombasa	Female	Married/Pa	Primary	8	None	Casual work
Nakuru	Female	Single	Secondary	4	None	Employment (private s
Nakuru	Female	Married/Pa	Higher	3	None	Employment (private s
Mombasa	Female	Married/Pa	Secondary	4	Business	Self-employment
Mombasa	Female	Married/Pa	Primary	8	Business	Self-employment
Nairobi	Female	Separated/	Primary	3	Casual Worker	Casual work
Kisumu	Female	Single	Secondary	2	None	Self-employment
Nairobi	Female	Married/Pa	Secondary	4	None	Casual work
Nakuru	Female	Separated/	Primary	8	Casual Worker	Employment (private s
Nakuru	Female	Single	Higher	1	Student	Employment (civil serv
Kisumu	Female	Married/Pa	Primary	7	Casual Worker	Casual work
Mombasa	Female	Married/Pa	Primary	4	None	None
Nakuru	Female	Married/Pa	Secondary	4	Business	Self-employment
Mombasa	Male	Single	Primary	8	Casual Worker	Casual work
Kisumu	Female	Married/Pa	Secondary	4	Business	Self-employment
Kisumu	Female	Single	Secondary	4	Business	Self-employment
Mombasa	Female	Married/Pa	None		None	Casual work
Mombasa	Male	Married/Pa	None		Casual Worker	Casual work
Mombasa	Male	Single	Primary	8	Casual Worker	Casual work
Kisumu	Female	Widowed	None		Business	Self-employment
Mombasa	Male	Married/Pa	Primary	7	None	Casual work
Mombasa	Male	Married/Pa	Primary	3	Casual Worker	Casual work
Kisumu	Male	Married/Pa	Primary	6	Business	Self-employment
Kisumu	Female	Widowed	Primary	6	Business	Self-employment
Nakuru	Male	Separated/	Primary	4	Casual Worker	Casual work
Kisumu	Female	Single	Secondary	4	Business	Self-employment
Mombasa	Female	Married/Pa	Primary	8	None	Casual work
Mombasa	Male	Married/Pa	Primary	5	Casual Worker	None
Mombasa	Female	Married/Pa	Primary	3	None	None
Mombasa	Female	Married/Pa	Primary	2	Casual Worker	Casual work
Mombasa	Female	Widowed	None		None	Self-employment
Mombasa	Male	Separated/	Primary	4	None	None
Mombasa	Female	Married/Pa	Primary	1	None	Self-employment
Mombasa	Male	Married/Pa	Primary	8	None	Self-employment
Mombasa	Male	Married/Pa	Primary	7	Casual Worker	Casual work
Mombasa	Female	Married/Pa	None		None	None
Nakuru	Female	Married/Pa	Primary	8	Business	Self-employment
Nakuru	Female	Married/Pa	Secondary	3	None	Self-employment
Mombasa	Male	Single	Primary	8	None	Employment (civil serv
Kisumu	Female	Widowed	None		None	Self-employment
Mombasa	Female	Separated/	Primary	6	Business	Self-employment
Kisumu	Female	Married/Pa	Secondary	2	Business	Self-employment
Nakuru	Female	Married/Pa	Secondary	4	Casual Worker	Employment (private s
Mombasa	Female	Married/Pa	Secondary	4	None	Casual work
Mombasa	Female	Married/Pa	Primary	8	None	Casual work
Mombasa	Male	Separated/	Primary	6	Casual Worker	Casual work
Nakuru	Female	Prefer not t	None		Business	Self-employment
Mombasa	Female	Married/Pa	Primary	7	None	Casual work

Kisumu	Female	Married/Pa	Higher	4	Business	Self-employment
Mombasa	Female	Married/Pa	None		None	Employment (private s
Kisumu	Female	Married/Pa	Secondary	4	Business	Self-employment
Mombasa	Female	Married/Pa	None		Casual Worker	Self-employment
Mombasa	Female	Single	None		Casual Worker	Casual work
Kisumu	Female	Married/Pa	Secondary	4	Casual Worker	Casual work
Kisumu	Female	Separated/	Primary	8	Business	Self-employment
Mombasa	Male	Widowed	None		None	Self-employment
Mombasa	Male	Separated/	Secondary	4	None	Self-employment
Nakuru	Male	Married/Pa	Primary	8	Casual Worker	Casual work
Mombasa	Male	Single	Secondary	4	Casual Worker	Casual work
Nakuru	Female	Married/Pa	Primary	8	None	Self-employment
Nakuru	Female	Single	Secondary	3	Casual Worker	Self-employment
Kisumu	Female	Married/Pa	Primary	7	Business	Self-employment
Mombasa	Female	Separated/	Primary	7	Business	Self-employment
Kisumu	Female	Married/Pa	Secondary	2	None	Self-employment
Mombasa	Female	Widowed	Primary	7	None	Self-employment
Nairobi	Male	Single	Secondary	4	Casual Worker	Casual work
Nairobi	Female	Married/Pa	Primary	8	Business	Self-employment
Mombasa	Female	Single	Secondary	4	Casual Worker	Casual work
Mombasa	Female	Married/Pa	Higher	2	Business	Self-employment
Nairobi	Female	Married/Pa	Secondary	4	None	Employment (private s
Nakuru	Female	Single	Secondary	4	None	Casual work
Mombasa	Male	Single	Higher	4	Casual Worker	Casual work
Mombasa	Male	Married/Pa	None		None	Self-employment
Nakuru	Female	Married/Pa	Primary	6	Business	Casual work
Mombasa	Female	Prefer not t	Primary	2	Casual Worker	Casual work
Kisumu	Female	Widowed	None		Business	Self-employment
Nakuru	Female	Married/Pa	Primary	8	Business	Employment (private s
Nakuru	Female	Single	Secondary	4	None	Employment (private s
Mombasa	Female	Widowed	Primary	8	None	None
Mombasa	Female	Married/Pa	Primary	2	Business	Self-employment
Nairobi	Female	Married/Pa	Primary	8	Casual Worker	Casual work
Mombasa	Female	Married/Pa	None		None	Employment (private s
Nairobi	Male	Single	Higher	4	Casual Worker	Casual work
Mombasa	Male	Married/Pa	None		Casual Worker	None
Mombasa	Female	Married/Pa	Secondary	8	Casual Worker	Casual work
Mombasa	Male	Married/Pa	Primary	5	Casual Worker	Self-employment
Mombasa	Female	Married/Pa	None		None	Employment (private s
Mombasa	Female	Single	Secondary	1	Casual Worker	Casual work
Nakuru	Female	Widowed	Primary	2	Business	Self-employment
Mombasa	Female	Single	Primary	6	None	Self-employment
Mombasa	Female	Married/Pa	Secondary	4	None	Self-employment
Mombasa	Female	Married/Pa	Higher	3	Professional	Employment (civil serv
Mombasa	Male	Married/Pa	Primary	8	Casual Worker	Casual work
Nairobi	Male	Married/Pa	Higher	2	Casual Worker	Casual work
Nairobi	Female	Married/Pa	Higher	4	Professional	Casual work
Mombasa	Male	Single	Higher	2	Casual Worker	Self-employment
Kisumu	Female	Married/Pa	Secondary	4	None	Casual work

Mombasa	Male	Widowed	Primary	4	None	None
Kisumu	Female	Married/Pa	Secondary	4	Business	Employment (civil serv
Mombasa	Female	Widowed	Primary	3	Business	Self-employment
Nairobi	Male	Married/Pa	Primary	7	Casual Worker	Casual work
Mombasa	Male	Married/Pa	Secondary	2	Casual Worker	Self-employment
Nairobi	Female	Married/Pa	Primary	8	None	Casual work
Mombasa	Male	Widowed	Primary	7	Professional	Casual work
Nairobi	Female	Married/Pa	Primary	4	Casual Worker	Casual work
Nakuru	Female	Separated/	Primary	6	Casual Worker	Casual work
Mombasa	Male	Married/Pa	Higher	3	Professional	Self-employment
Kisumu	Female	Married/Pa	Primary	3	None	None
Mombasa	Female	Widowed	None		Casual Worker	Casual work
Kisumu	Female	Married/Pa	Secondary	2	Business	Self-employment
Mombasa	Female	Married/Pa	None		Casual Worker	None
Nairobi	Male	Single	Primary	8	Janitorial/guard	Employment (private s
Nairobi	Male	Single	Secondary	4	Casual Worker	Casual work
Mombasa	Female	Married/Pa	None		None	Casual work
Nakuru	Female	Single	Primary	7	Casual Worker	Casual work
Mombasa	Male	Single	Secondary	4	Casual Worker	Casual work
Kisumu	Male	Married/Pa	Primary	8	Professional	Employment (private s
Mombasa	Male	Married/Pa	Primary	2	None	Casual work
Kisumu	Female	Married/Pa	Primary	8	Business	Self-employment
Mombasa	Male	Married/Pa	Secondary	4	None	Self-employment
Nakuru	Female	Married/Pa	Secondary	4	None	Employment (private s
Nakuru	Female	Married/Pa	Primary	4	Casual Worker	Casual work
Mombasa	Male	Married/Pa	Secondary	4	Casual Worker	Casual work
Kisumu	Female	Married/Pa	Secondary	4	None	Self-employment
Mombasa	Male	Married/Pa	Secondary	4	Casual Worker	Casual work
Kisumu	Female	Single	Secondary	1	Business	Self-employment
Kisumu	Female	Married/Pa	Higher	3	None	Employment (civil serv
Nakuru	Female	Married/Pa	Primary	8	None	Casual work
Mombasa	Male	Married/Pa	Primary	8	None	Casual work
Nakuru	Male	Married/Pa	Secondary	2	Business	Self-employment
Mombasa	Female	Married/Pa	Secondary	4	None	Self-employment
Mombasa	Female	Married/Pa	Primary	8	Business	Employment (private s
Mombasa	Female	Separated/	Primary	8	Casual Worker	Casual work
Nairobi	Male	Married/Pa	Secondary	2	Casual Worker	Casual work
Mombasa	Female	Married/Pa	Secondary	4	Business	Self-employment
Nakuru	Female	Married/Pa	Primary	7	Business	Self-employment
Mombasa	Female	Single	Primary	5	None	Casual work
Kisumu	Female	Married/Pa	Primary	8	None	Employment (private s
Mombasa	Female	Separated/	Primary	3	None	None
Kisumu	Female	Married/Pa	Higher	4	Business	Self-employment
Mombasa	Female	Married/Pa	Primary	6	None	Self-employment
Mombasa	Female	Married/Pa	Primary	8	None	Casual work
Kisumu	Female	Married/Pa	Higher	2	Business	Self-employment
Nakuru	Female	Married/Pa	Primary	8	None	Casual work
Nakuru	Female	Married/Pa	Primary	8	Casual Worker	Self-employment
Nakuru	Female	Separated/	Primary	8	Business	Self-employment

Nakuru	Female	Married/Pa	Primary	3	Casual Worker	Self-employment
Kisumu	Male	Married/Pa	Primary	8	Business	Self-employment
Nairobi	Female	Married/Pa	Primary	7	None	Self-employment
Mombasa	Female	Married/Pa	Primary	7	Casual Worker	Self-employment
Nakuru	Female	Married/Pa	Primary	8	Business	Self-employment
Kisumu	Female	Separated/	Primary	7	Business	Self-employment
Kisumu	Female	Widowed	None		Business	Self-employment
Mombasa	Female	Married/Pa	None		Casual Worker	Casual work
Mombasa	Female	Widowed	None		None	Self-employment
Kisumu	Female	Married/Pa	Secondary	4	None	Self-employment
Nairobi	Female	Single	Secondary	4	Casual Worker	Casual work
Mombasa	Female	Married/Pa	Primary	6	Casual Worker	Casual work
Kisumu	Female	Married/Pa	Secondary	3	Casual Worker	Self-employment
Mombasa	Male	Single	Secondary	2	None	Casual work
Kisumu	Female	Married/Pa	Primary	8	None	Casual work
Nairobi	Male	Single	Secondary	4	Casual Worker	Casual work
Nakuru	Female	Single	Higher	2	None	None
Mombasa	Female	Married/Pa	Primary	8	Business	Self-employment
Nairobi	Female	Married/Pa	Primary	8	Casual Worker	Casual work
Kisumu	Female	Married/Pa	Primary	8	Business	Self-employment
Mombasa	Male	Married/Pa	Higher	4	None	None
Mombasa	Female	Married/Pa	Primary	8	None	Casual work
Nakuru	Female	Married/Pa	Secondary	2	None	Self-employment
Mombasa	Male	Married/Pa	Primary	5	Casual Worker	Self-employment
Kisumu	Female	Married/Pa	Primary	7	Business	Self-employment
Nakuru	Female	Married/Pa	Primary	8	Casual Worker	Casual work
Nakuru	Female	Married/Pa	Primary	8	None	Casual work
Mombasa	Female	Married/Pa	Primary	6	Business	Self-employment
Kisumu	Female	Widowed	Secondary	4	Business	Self-employment
Nakuru	Female	Married/Pa	Primary	8	Business	Casual work
Mombasa	Female	Separated/	Secondary	1	Casual Worker	Casual work
Kisumu	Female	Single	Secondary	3	None	Employment (private s
Nakuru	Female	Separated/	Primary	8	Business	Employment (private s
Kisumu	Female	Married/Pa	Primary	5	Business	Self-employment
Mombasa	Male	Married/Pa	Higher	3	None	Self-employment
Mombasa	Female	Married/Pa	Secondary	4	None	Self-employment
Nakuru	Female	Married/Pa	Secondary	4	Professional	Employment (private s
Nakuru	Female	Separated/	Secondary	4	Business	Self-employment
Nakuru	Female	Married/Pa	Secondary	4	None	Casual work
Kisumu	Female	Single	Secondary	4	Casual Worker	Casual work
Kisumu	Female	Married/Pa	Primary	8	Professional	Self-employment
Nakuru	Female	Married/Pa	Secondary	2	Business	Self-employment
Nakuru	Male	Married/Pa	Higher	4	Professional	Employment (civil serv
Mombasa	Female	Single	Secondary	4	Casual Worker	Casual work
Nakuru	Female	Separated/	Primary	1	Casual Worker	Casual work
Nakuru	Female	Married/Pa	Higher	1	None	Self-employment
Mombasa	Male	Married/Pa	Primary	8	Business	Self-employment
Kisumu	Female	Widowed	Primary	3	Business	Employment (private s
Kisumu	Female	Widowed	None		Business	Self-employment

Nakuru	Female	Married/Pa	Secondary	4	None		Family Sup	Receives s
Nakuru	Female	Married/Pa	Higher	2	Business		Self-employment	
Kisumu	Female	Separated/	Primary	7	Casual Worker		Casual work	
Nakuru	Female	Married/Pa	Primary	8	None		Employment (private s	
Kisumu	Female	Married/Pa	Secondary	2	Business		Self-employment	
Kisumu	Female	Single	None		Business		Self-employment	
Nakuru	Female	Married/Pa	Primary	8	Casual Worker		Casual work	
Kisumu	Female	Single	Primary	7	Business		Self-employment	
Nakuru	Female	Separated/	Secondary	3	Business		Self-employment	
Kisumu	Female	Single	Higher	1	None		None	
Kisumu	Female	Widowed	Primary	6	Business		Self-employment	
Mombasa	Male	Married/Pa	Secondary	4	Business		Self-employment	
Mombasa	Male	Single	Primary	6	None		Casual work	
Kisumu	Male	Single	Secondary	2	Casual Worker		Casual work	
Kisumu	Female	Single	Secondary	3	Student		Self-employment	
Nairobi	Female	Married/Pa	Secondary	4	Casual Worker		Casual work	
Nakuru	Female	Separated/	Higher	3	Business		Self-employment	
Nakuru	Female	Married/Pa	Secondary	4	None		Self-employment	
Kisumu	Female	Married/Pa	Secondary	4	None		Self-employment	
Kisumu	Female	Widowed	Primary	4	Business		Self-employment	
Kisumu	Male	Separated/	Primary	8	Other	Farmer	Self-employment	
Nakuru	Female	Married/Pa	Secondary	4	Casual Worker		Employment (private s	
Nairobi	Female	Single	Secondary	4	None		Employment (private s	
Mombasa	Male	Single	Higher	4	None		None	
Nairobi	Female	Single	Higher	2	None		None	
Nakuru	Female	Married/Pa	Secondary	4	None		Employment (private s	
Mombasa	Female	Married/Pa	Secondary	1	Business		Self-employment	
Mombasa	Female	Married/Pa	Primary	8	None		Self-employment	
Mombasa	Male	Single	Primary	4	None		None	
Kisumu	Female	Married/Pa	Higher	3	None		Self-employment	
Nairobi	Female	Married/Pa	Secondary	2	None		Employment (private s	
Nairobi	Female	Married/Pa	Secondary	4	None		Employment (private s	
Kisumu	Female	Single	Primary	8	Professional		Employment (private s	
Mombasa	Female	Married/Pa	Primary	7	Casual Worker		Self-employment	
Kisumu	Female	Married/Pa	Secondary	2	None		Self-employment	
Kisumu	Female	Single	Secondary	4	Business		Self-employment	
Nairobi	Female	Married/Pa	Primary	7	Business		Casual work	
Nakuru	Female	Married/Pa	Secondary	4	None		Employment (private s	
Kisumu	Female	Married/Pa	Secondary	4	None		Self-employment	
Kisumu	Female	Widowed	Primary	8	Business		Self-employment	
Nakuru	Female	Single	Primary	4	Casual Worker		Casual work	
Kisumu	Female	Married/Pa	Primary	8	Business		Self-employment	
Kisumu	Male	Single	Higher	1	Student		Self-employment	
Nairobi	Female	Married/Pa	Primary	8	None		None	
Mombasa	Female	Married/Pa	Primary	8	None		Casual work	
Nairobi	Female	Single	Secondary	4	Casual Worker		Casual work	
Nairobi	Female	Married/Pa	Higher	2	None		Self-employment	
Nairobi	Female	Married/Pa	Primary	8	Casual Worker		Casual work	
Kisumu	Female	Widowed	Primary	8	Business		Self-employment	

Mombasa	Female	Married/Pa	Secondary	4	Business	Employment (civil serv
Kisumu	Female	Separated/	Secondary	4	Casual Worker	Casual work
Nairobi	Female	Married/Pa	Higher	2	None	Casual work
Nakuru	Female	Married/Pa	Primary	8	Casual Worker	Casual work
Nakuru	Female	Married/Pa	Secondary	2	Business	Self-employment
Nakuru	Female	Separated/	Primary	7	None	Casual work
Kisumu	Male	Married/Pa	Secondary	4	Casual Worker	Casual work
Nairobi	Female	Married/Pa	Secondary	3	None	Self-emplo business
Nairobi	Female	Married/Pa	Secondary	4	None	Casual work
Nakuru	Female	Married/Pa	Primary	8	None	Casual work
Nairobi	Female	Married/Pa	Secondary	4	None	Employment (private s
Nakuru	Female	Separated/	Secondary	4	Business	Self-employment
Mombasa	Male	Married/Pa	Primary	8	Casual Worker	Self-employment
Mombasa	Female	Separated/	None		Business	Casual work
Nairobi	Female	Married/Pa	Secondary	4	Professional	Employment (private s
Nairobi	Female	Single	Secondary	2	None	Employment (private s
Nakuru	Female	Separated/	Primary	8	Casual Worker	Casual work
Kisumu	Female	Married/Pa	Primary	7	Business	Self-employment
Nairobi	Male	Married/Pa	Secondary	4	Casual Worker	Self-employment
Kisumu	Female	Single	Secondary	4	Business	Self-employment
Nakuru	Male	Single	Secondary	4	None	Self-employment
Nakuru	Female	Married/Pa	Primary	8	None	Casual work
Kisumu	Female	Married/Pa	Secondary	4	Business	Self-employment
Kisumu	Female	Married/Pa	Primary	6	Business	Self-employment
Kisumu	Female	Married/Pa	Higher	4	Casual Worker	Casual work
Nairobi	Female	Single	Higher	2	Casual Worker	Casual work
Kisumu	Female	Married/Pa	Primary	8	Business	Self-employment
Nakuru	Female	Married/Pa	Secondary	4	Business	Employment (private s
Kisumu	Female	Married/Pa	Secondary	4	Business	Self-employment
Kisumu	Female	Married/Pa	Secondary	2	None	Employment (private s
Nakuru	Female	Single	Secondary	4	Business	Self-employment
Kisumu	Female	Married/Pa	Secondary	2	Business	Self-employment
Nairobi	Female	Separated/	Primary	8	Casual Worker	Casual work
Nairobi	Female	Widowed	Primary	8	Casual Worker	Casual work
Nairobi	Male	Married/Pa	Secondary	4	Casual Worker	Casual work
Kisumu	Female	Single	Higher	1	Student	Self-employment
Nakuru	Female	Separated/	Secondary	2	None	None
Kisumu	Male	Single	Higher	3	None	Self-employment
Nairobi	Female	Married/Pa	Primary	5	None	None
Mombasa	Female	Married/Pa	Secondary	2	Business	Employment (private s
Nairobi	Female	Married/Pa	Secondary	4	None	Employment (private s
Nakuru	Female	Married/Pa	Higher	4	Business	Self-employment
Nairobi	Female	Single	Secondary	4	None	Employment (private s
Nairobi	Female	Married/Pa	Secondary	4	None	None
Nairobi	Male	Married/Pa	Secondary	4	Casual Worker	Casual work
Nairobi	Female	Widowed	Primary	8	None	Casual work
Nakuru	Female	Married/Pa	Primary	8	Casual Worker	Casual work
Mombasa	Female	Single	Secondary	4	None	Casual work
Nairobi	Male	Married/Pa	Higher	2	Casual Worker	Casual work

Nairobi	Female	Single	Secondary	4	Business	Employment (private s
Nakuru	Female	Separated/	Primary	2	Casual Worker	Casual work
Nakuru	Female	Married/Pa	Primary	8	Business	Self-employment
Kisumu	Female	Married/Pa	Primary	7	Business	Self-employment
Kisumu	Female	Married/Pa	Primary	8	Casual Worker	Employment (civil serv
Nairobi	Female	Married/Pa	Secondary	4	None	Self-employment
Kisumu	Female	Married/Pa	Secondary	4	Business	Self-employment
Nairobi	Female	Married/Pa	Secondary	6	None	Casual work
Nakuru	Female	Married/Pa	Secondary	4	None	Casual work
Kisumu	Female	Married/Pa	Primary	3	Casual Worker	Casual work
Kisumu	Female	Married/Pa	Secondary	4	Business	Self-employment
Mombasa	Female	Married/Pa	Primary	7	Business	Self-employment
Mombasa	Female	Married/Pa	Primary	5	Casual Worker	Self-employment
Kisumu	Female	Married/Pa	Secondary	4	None	Employment (private s
Nakuru	Female	Married/Pa	Primary	8	None	Casual work
Nakuru	Female	Married/Pa	Secondary	4	Casual Worker	Self-employment
Mombasa	Female	Married/Pa	Higher	2.5	None	Self-employment
Nairobi	Male	Single	Primary	7	Casual Worker	Casual work
Mombasa	Female	Married/Pa	None		None	Casual work
Mombasa	Female	Single	Primary	5	None	Self-employment
Nairobi	Female	Married/Pa	Secondary	4	None	Employment (private s
Kisumu	Male	Married/Pa	Primary	4	None	Self-employment
Mombasa	Male	Married/Pa	Secondary	4	Casual Worker	Casual work
Nakuru	Female	Married/Pa	Secondary	2	None	Self-employment
Kisumu	Male	Married/Pa	Higher	2	Business	Self-employment
Kisumu	Female	Widowed	Primary	8	Business	Self-employment
Mombasa	Female	Married/Pa	Primary	8	Business	Casual work
Kisumu	Female	Married/Pa	Secondary	2	Casual Worker	Self-employment
Kisumu	Female	Married/Pa	Primary	8	Business	Self-employment
Kisumu	Female	Married/Pa	Secondary	4	None	Employment (private s
Kisumu	Male	Married/Pa	Secondary	4	Business	Self-employment
Nairobi	Female	Married/Pa	Secondary	4	Casual Worker	Casual work
Nakuru	Female	Prefer not t	Secondary	4	None	Casual work
Nakuru	Female	Married/Pa	Secondary	2	Professional	Employment (civil serv
Kisumu	Female	Married/Pa	Primary	8	Casual Worker	Casual work
Mombasa	Male	Married/Pa	Secondary	4	Business	Self-employment
Kisumu	Female	Married/Pa	Primary	8	None	Casual work
Nakuru	Female	Married/Pa	Secondary	4	Business	None
Mombasa	Female	Single	Primary	8	None	Casual work
Nakuru	Female	Married/Pa	Higher	1	Janitorial/guard	Employment (private s
Nakuru	Female	Married/Pa	Secondary	3	None	Self-employment
Nakuru	Female	Married/Pa	Primary	8	Business	Self-employment
Mombasa	Female	Separated/	Primary	5	Casual Worker	Casual work
Kisumu	Female	Married/Pa	Higher	3	Business	Self-employment
Nakuru	Male	Single	Secondary	4	Business	Self-employment
Nairobi	Female	Separated/	Primary	7	Business	Self-employment
Nairobi	Male	Single	Secondary	4	None	Employment (private s
Kisumu	Female	Married/Pa	Secondary	4	Business	Self-employment
Nairobi	Male	Married/Pa	Secondary	4	Casual Worker	Casual work

Nakuru	Female	Married/Pa	Secondary	4	None		Self-employment	
Kisumu	Female	Married/Pa	Primary	8	None		Casual work	
Nairobi	Female	Married/Pa	Primary	6	Casual Worker		Casual work	
Kisumu	Male	Married/Pa	Primary	7	Business		Self-employment	
Nairobi	Female	Married/Pa	Secondary	2	Other	Land Owne	Self-employment	
Mombasa	Female	Single	Primary	8	Casual Worker		Casual work	
Mombasa	Female	Married/Pa	Primary	8	Business		Employment (private s	
Mombasa	Female	Married/Pa	Higher	3	Casual Worker		Self-employment	
Nairobi	Female	Widowed	None		Casual Worker		Casual work	
Nairobi	Male	Single	Secondary	4	Casual Worker		Casual work	
Kisumu	Female	Widowed	Primary	7	Casual Worker		Self-employment	
Kisumu	Female	Married/Pa	Primary	8	Casual Worker		Casual work	
Kisumu	Female	Married/Pa	Primary	8	Business		Self-employment	
Mombasa	Female	Married/Pa	None		Casual Worker		Casual work	
Kisumu	Female	Married/Pa	None		Business		Self-employment	
Kisumu	Female	Married/Pa	Primary	8	None		Casual work	
Kisumu	Female	Married/Pa	Higher	4	Other	CHV	Self-employment	
Nairobi	Female	Married/Pa	Secondary	2	None		Employment (private s	
Kisumu	Female	Widowed	Primary	7	Business		Casual wo	Tailoring
Nakuru	Female	Married/Pa	Primary	8	Casual Worker		Casual work	
Nairobi	Male	Married/Pa	Secondary	4	Janitorial/guard		Employment (private s	
Nakuru	Female	Married/Pa	Primary	8	None		Casual work	
Nakuru	Female	Married/Pa	Secondary	4	None		Self-employment	
Kisumu	Female	Married/Pa	Secondary	4	Casual Worker		Employment (private s	
Nairobi	Male	Single	Secondary	4	Professional		Employment (private s	
Kisumu	Female	Married/Pa	Secondary	2	Business		Employment (private s	
Mombasa	Female	Married/Pa	Primary	8	None		Self-employment	
Nairobi	Male	Single	Secondary	4	None		Casual work	
Nairobi	Female	Single	Higher	2	None		Casual work	
Nakuru	Female	Married/Pa	Primary	4	Casual Worker		Casual work	
Kisumu	Male	Widowed	None		Business		Self-employment	
Nairobi	Female	Married/Pa	Primary	8	None		None	
Nairobi	Male	Single	Secondary	4	Casual Worker		Casual work	
Nairobi	Female	Single	Secondary	4	Casual Worker		Casual work	
Mombasa	Female	Married/Pa	Primary	8	None		Self-employment	
Nairobi	Female	Married/Pa	Primary	7	None		None	
Kisumu	Female	Married/Pa	Primary	6	Business		Self-employment	
Nakuru	Female	Married/Pa	Secondary	4	None		Family Sup	Wellwisher
Nairobi	Female	Single	Secondary	2	Casual Worker		Casual work	
Nairobi	Female	Married/Pa	Secondary	3	None		Self-employment	
Mombasa	Female	Separated/	None		Business		Self-employment	
Nairobi	Female	Married/Pa	Secondary	4	Casual Worker		Self-employment	
Nairobi	Male	Married/Pa	Secondary	4	Janitorial/guard		Employment (civil serv	
Nakuru	Female	Married/Pa	Higher	2	None		Employment (private s	
Kisumu	Female	Married/Pa	Secondary	4	Casual Worker		Self-employment	
Mombasa	Female	Single	Higher	3	None		Employment (private s	
Nairobi	Female	Single	Higher	1	None		None	
Mombasa	Female	Single	Higher	3	Professional		Employment (private s	
Nakuru	Female	Married/Pa	Primary	8	Casual Worker		Self-employment	

Nairobi	Male	Married/Pa	Secondary	4	Casual Worker		Casual work
Kisumu	Female	Married/Pa	Primary	8	Business		Self-employment
Nairobi	Male	Married/Pa	Secondary	4	Janitorial/guard		Employment (civil serv
Nakuru	Female	Separated/	Primary	8	Casual Worker		Casual work
Nakuru	Female	Married/Pa	Secondary	4	Business		Self-employment
Kisumu	Female	Married/Pa	Primary	7	None		Self-employment
Kisumu	Female	Married/Pa	Primary	8	Business		Self-employment
Kisumu	Female	Married/Pa	Primary	7	None		Self-employment
Mombasa	Female	Widowed	Primary	8	Business		Self-employment
Kisumu	Female	Widowed	Primary	7	Business		Self-employment
Mombasa	Female	Married/Pa	Secondary	4	Casual Worker		Casual work
Nairobi	Male	Single	Secondary	4	Casual Worker		Casual work
Mombasa	Female	Single	Higher	1	None		None
Kisumu	Female	Married/Pa	Secondary	4	None		Casual work
Mombasa	Female	Married/Pa	Primary	8	Business		Self-employment
Nairobi	Female	Married/Pa	Secondary	4	Business		Employment (private s
Nakuru	Female	Married/Pa	Secondary	4	Casual Worker		Casual work
Nakuru	Male	Single	Secondary	4	Casual Worker		Self-employment
Mombasa	Female	Widowed	Primary	7	None		Self-employment
Kisumu	Female	Married/Pa	Secondary	2	Business		Self-employment
Nakuru	Female	Married/Pa	Higher	3	Business		Self-employment
Mombasa	Female	Married/Pa	None		Business		Self-employment
Nairobi	Female	Single	Higher	4	Business		Self-employment
Kisumu	Female	Married/Pa	Secondary	4	Business		Self-employment
Nairobi	Female	Single	Secondary	4	Professional		Casual work
Kisumu	Female	Married/Pa	Secondary	2	None		Self-employment
Mombasa	Female	Married/Pa	Primary	4	None		Casual work
Nakuru	Female	Married/Pa	Secondary	3	Business		Employment (private s
Kisumu	Female	Married/Pa	Primary	8	Business		Self-employment
Nairobi	Male	Married/Pa	Secondary	4	Other	Land Owne	Self-employment
Kisumu	Female	Separated/	Secondary	4	Business		Self-employment
Nakuru	Female	Married/Pa	Higher	3	Business		Self-employment
Nairobi	Female	Single	Primary	6	Casual Worker		Casual work
Kisumu	Female	Married/Pa	Primary	8	Business		Self-employment
Kisumu	Male	Single	Higher	3	Student		Self-employment
Nakuru	Female	Single	Primary	8	None		Casual work
Kisumu	Female	Single	Higher	4	None		Employment (civil serv
Nairobi	Female	Married/Pa	Secondary	4	None		Casual work
Kisumu	Female	Single	Secondary	4	None		Employment (private s
Kisumu	Female	Married/Pa	Secondary	2	Business		Self-employment
Kisumu	Female	Widowed	Primary	7	Business		Self-employment
Mombasa	Male	Married/Pa	Primary	7	Casual Worker		Casual work
Kisumu	Female	Married/Pa	Higher	3	None		Self-employment
Kisumu	Female	Married/Pa	Secondary	4	Casual Worker		Employment (private s
Kisumu	Female	Single	Higher	1	None		Self-employment
Mombasa	Female	Single	Secondary	4	Casual Worker		Casual work
Kisumu	Female	Married/Pa	Secondary	4	None		Employment (private s
Kisumu	Female	Married/Pa	Secondary	4	Casual Worker		Employment (private s
Kisumu	Female	Married/Pa	Secondary	4	Casual Worker		Employment (private s

Nakuru	Female	Single	Secondary	4	Student	Self-employment
Nairobi	Female	Married/Pa	Secondary	4	None	Employment (private s
Mombasa	Female	Widowed	None		None	Self-employment
Kisumu	Female	Widowed	None		Business	Self-employment
Nairobi	Female	Married/Pa	Primary	8	None	Employment (private s
Kisumu	Female	Single	Secondary	3	None	Employment (private s
Nakuru	Female	Married/Pa	Secondary	2	None	Employment (private s
Nakuru	Female	Married/Pa	Higher	3	Business	Self-employment
Nairobi	Female	Single	Secondary	4	None	Self-employment
Nairobi	Male	Married/Pa	Secondary	4	Student	Self-employment
Nakuru	Female	Married/Pa	Secondary	3	None	Employment (private s
Nairobi	Female	Separated/	Primary	3	None	Self-employment
Nakuru	Female	Married/Pa	Primary	8	Business	Employment (private s
Nairobi	Female	Married/Pa	Secondary	2	None	Casual work
Nakuru	Male	Married/Pa	Secondary	3	Casual Worker	Casual work
Nairobi	Male	Married/Pa	Higher	1	Casual Worker	Casual work
Mombasa	Male	Married/Pa	Secondary	7	Casual Worker	Self-employment
Kisumu	Male	Single	Higher	3	Business	Self-employment
Mombasa	Female	Married/Pa	Primary	8	None	Self-employment
Kisumu	Female	Married/Pa	Primary	8	None	Self-employment
Kisumu	Female	Married/Pa	Higher	2	Business	Self-employment
Mombasa	Male	Single	Secondary	4	Casual Worker	Employment (private s
Mombasa	Male	Widowed	None		Casual Worker	Casual work
Kisumu	Female	Married/Pa	Higher	2	Casual Worker	Self-employment
Kisumu	Female	Married/Pa	Primary	7	Casual Worker	Casual work
Mombasa	Female	Married/Pa	None		None	Casual work
Mombasa	Female	Married/Pa	Primary	8	Business	Employment (private s
Kisumu	Female	Married/Pa	Primary	8	Business	Self-employment
Mombasa	Female	Married/Pa	Primary	8	None	Self-employment
Mombasa	Male	Separated/	Secondary	4	Business	Casual work
Kisumu	Female	Married/Pa	Primary	8	None	Self-employment
Mombasa	Female	Married/Pa	Secondary	4	Business	None
Kisumu	Female	Married/Pa	Primary	8	Business	Self-employment
Kisumu	Female	Married/Pa	Higher	3	Professional	Employment (private s
Kisumu	Female	Widowed	Primary	8	Casual Worker	Casual work
Nakuru	Female	Married/Pa	Secondary	2	Business	Self-employment
Nairobi	Male	Married/Pa	Secondary	4	Janitorial/guard	Employment (private s
Kisumu	Female	Widowed	Primary	7	None	None
Nairobi	Female	Single	Primary	2	Casual Worker	Casual work
Kisumu	Female	Married/Pa	Primary	8	Business	Self-employment
Nakuru	Female	Married/Pa	Primary	8	Business	Employment (private s
Nakuru	Female	Single	Secondary	4	Casual Worker	Self-employment
Nakuru	Female	Married/Pa	Secondary	3	Business	Self-employment
Nairobi	Male	Married/Pa	Secondary	4	Janitorial/guard	Employment (private s
Mombasa	Female	Widowed	None		None	None
Kisumu	Female	Married/Pa	Higher	4	Professional	Employment (civil serv
Nairobi	Female	Single	Primary	6	None	Self-employment
Nairobi	Female	Married/Pa	Higher	3	Casual Worker	Casual work
Kisumu	Female	Married/Pa	Secondary	4	None	Self-employment

Nakuru	Female	Married/Pa	Higher	3	Business		Self-employment
Kisumu	Female	Married/Pa	Higher	4	Business		Self-employment
Nairobi	Male	Single	Secondary	4	Casual Worker		Casual work
Mombasa	Female	Single	Secondary	4	None		Casual work
Mombasa	Female	Married/Pa	Primary	7	Business		Self-employment
Kisumu	Female	Single	Higher	1	None		Employment (private s
Mombasa	Male	Single	Secondary	4	None		Employment (private s
Kisumu	Female	Single	Higher	4	Professional		Employment (civil serv
Kisumu	Female	Married/Pa	Primary	7	Business		Self-employment
Nakuru	Female	Married/Pa	Secondary	4	Business		Casual work
Mombasa	Female	Married/Pa	Primary	3	Business		Casual work
Nairobi	Male	Single	Secondary	3	None		Employment (private s
Nairobi	Female	Married/Pa	Secondary	4	None		Self-employment
Nairobi	Female	Single	Secondary	2	None		Casual work
Nakuru	Female	Single	Primary	8	Casual Worker		Employment (private s
Kisumu	Female	Widowed	Primary	7	None		Self-employment
Kisumu	Male	Single	Higher	4	Professional		Employment (private s
Kisumu	Female	Married/Pa	Secondary	3	Business		Self-employment
Kisumu	Female	Married/Pa	Primary	8	Business		Self-employment
Nakuru	Female	Married/Pa	Secondary	4	None		Casual work
Mombasa	Female	Widowed	Primary	5	None		Casual work
Nakuru	Female	Married/Pa	Secondary	4	Business		Self-employment
Nakuru	Female	Married/Pa	Secondary	4	None		Self-employment
Nakuru	Male	Single	Higher	4	Casual Worker		Casual work
Nakuru	Female	Single	Secondary	4	Business		Employment (private s
Nakuru	Female	Married/Pa	Secondary	4	None		Self-employment
Nakuru	Female	Single	Higher	2	Business		Self-employment
Kisumu	Female	Married/Pa	Higher	3	None		Self-employment
Nairobi	Female	Married/Pa	Primary	7	Casual Worker		Self-employment
Kisumu	Female	Married/Pa	Primary	8	None		Self-employment
Kisumu	Female	Married/Pa	Secondary	4	Casual Worker		Self-employment
Mombasa	Female	Married/Pa	Secondary	4	Casual Worker		Self-employment
Kisumu	Female	Married/Pa	None		Business		Self-employment
Mombasa	Female	Married/Pa	Secondary	4	None		Casual work
Nakuru	Female	Separated/	Secondary	1	Business		Self-employment
Mombasa	Male	Married/Pa	None		Casual Worker		Casual work
Kisumu	Female	Married/Pa	Primary	8	None		None
Nairobi	Male	Married/Pa	Secondary	4	Janitorial/guard		Employment (private s
Nairobi	Female	Married/Pa	Higher	2	Professional		Casual work
Kisumu	Male	Single	Secondary	4	Business		Self-employment
Nakuru	Male	Married/Pa	Primary	8	Business		Self-employment
Nakuru	Male	Married/Pa	Secondary	4	Other	Farmer	Self-employment
Nakuru	Female	Separated/	Primary	8	Business		Self-employment
Nakuru	Female	Married/Pa	Higher	3	Business		Employment (private s
Kisumu	Male	Single	Secondary	3	Casual Worker		Employment (private s
Kisumu	Female	Married/Pa	Primary	8	Business		Self-employment
Nakuru	Female	Married/Pa	Primary	2	None		Casual work
Mombasa	Female	Married/Pa	Higher	3	Casual Worker		Casual work
Kisumu	Female	Married/Pa	Higher	3	None		Employment (private s

Nairobi	Female	Married/Pa	Primary	8	None	None
Kisumu	Female	Married/Pa	Higher	4	None	Self-employment
Mombasa	Female	Single	Secondary	4	Professional	Employment (private s
Kisumu	Female	Single	Secondary	4	Business	Self-employment
Kisumu	Female	Married/Pa	Secondary	4	Business	Self-employment
Kisumu	Female	Married/Pa	Secondary	4	Business	Self-employment
Nakuru	Female	Married/Pa	Primary	7	None	Self-employment
Nakuru	Female	Married/Pa	Primary	8	None	Employment (private s
Kisumu	Female	Separated/	Primary	8	Casual Worker	Casual work
Nakuru	Female	Married/Pa	Higher	6	Business	Self-employment
Nakuru	Male	Single	Secondary	3	Business	Self-employment
Kisumu	Female	Married/Pa	Secondary	2	None	Casual work
Nakuru	Female	Married/Pa	Secondary	4	None	Self-employment
Nakuru	Female	Married/Pa	Primary	8	None	Self-employment
Kisumu	Female	Widowed	Primary	8	Business	Self-employment
Nakuru	Female	Married/Pa	Primary	6	Business	Self-employment
Kisumu	Female	Married/Pa	Secondary	4	None	Self-employment
Kisumu	Male	Married/Pa	Secondary	1	Casual Worker	Casual work
Nairobi	Male	Single	Secondary	4	None	Family Sup for now not
Kisumu	Female	Married/Pa	Secondary	1	None	Self-employment
Nakuru	Female	Married/Pa	Primary	8	None	Employment (private s
Kisumu	Female	Married/Pa	Secondary	2	Business	Self-employment
Mombasa	Female	Married/Pa	Primary	6	Business	Self-employment
Nairobi	Female	Married/Pa	Primary	8	None	Self-employment
Nairobi	Female	Married/Pa	Secondary	4	None	Employment (private s
Nairobi	Female	Married/Pa	Higher	2	Casual Worker	Casual work
Mombasa	Female	Married/Pa	Higher	4	None	Self-employment
Nairobi	Female	Married/Pa	Secondary	4	None	Employment (private s
Nakuru	Female	Separated/	Secondary	1	Casual Worker	Casual work
Nakuru	Female	Married/Pa	Secondary	4	Business	Self-employment
Nairobi	Female	Married/Pa	Primary	8	None	None
Nakuru	Female	Separated/	Secondary	4	Casual Worker	Casual work
Nakuru	Female	Prefer not t	Higher	4	None	Self-employment
Nairobi	Female	Married/Pa	Primary	5	None	Casual work
Kisumu	Male	Single	Primary	8	Casual Worker	Casual work
Kisumu	Male	Married/Pa	Secondary	4	Professional	Employment (private s
Nakuru	Female	Married/Pa	Higher	2	Professional	Self-employment
Nairobi	Female	Married/Pa	Secondary	4	Casual Worker	Casual work
Mombasa	Male	Single	Primary	8	Casual Worker	Casual work
Nairobi	Male	Married/Pa	Secondary	4	Casual Worker	Casual work
Nakuru	Female	Married/Pa	Primary	7	None	Self-employment
Mombasa	Female	Single	Primary	8	Business	Casual work
Kisumu	Female	Married/Pa	Primary	8	Business	Employment (private s
Nakuru	Female	Single	Higher	2	Professional	Employment (private s
Nairobi	Female	Widowed	Primary	8	Casual Worker	Casual work
Mombasa	Female	Married/Pa	Secondary	4	None	Self-employment
Nakuru	Female	Single	Higher	3	Professional	Self-employment
Kisumu	Female	Single	Secondary	2	Business	Self-employment
Nakuru	Female	Married/Pa	Primary	7	Business	Self-employment

Nairobi	Female	Married/Pa	Secondary	4	Business	Self-employment	
Nakuru	Male	Married/Pa	Secondary	4	Business	Employment (private s	
Nairobi	Female	Single	Secondary	4	None	Casual work	
Nakuru	Female	Married/Pa	Higher	2	Business	Employment (private s	
Mombasa	Female	Single	Secondary	4	Professional	Casual wo	Volunteers
Kisumu	Female	Married/Pa	Primary	7	Business	Self-employment	
Kisumu	Female	Married/Pa	Primary	8	Business	Self-employment	
Kisumu	Female	Widowed	Primary	8	Business	Self-employment	
Nakuru	Female	Married/Pa	Secondary	2	None	Employment (private s	
Nairobi	Female	Married/Pa	Secondary	4	None	Casual work	
Nakuru	Female	Married/Pa	Secondary	2	None	Casual work	
Kisumu	Female	Widowed	Primary	6	Casual Worker	Self-employment	
Nairobi	Female	Married/Pa	Secondary	4	Professional	Employment (private s	
Nairobi	Female	Married/Pa	Secondary	4	Professional	None	
Kisumu	Female	Married/Pa	Secondary	4	None	Self-employment	
Kisumu	Male	Married/Pa	Secondary	4	None	Self-employment	
Mombasa	Female	Married/Pa	Primary	8	Casual Worker	Casual work	
Kisumu	Female	Married/Pa	Secondary	2	Business	Self-employment	
Nairobi	Female	Single	Secondary	1	None	Employment (private s	
Kisumu	Female	Married/Pa	Secondary	4	None	Self-employment	
Mombasa	Female	Separated/	Secondary	3	Business	Self-employment	
Nakuru	Female	Single	Higher	2	Business	Self-employment	
Kisumu	Female	Married/Pa	Primary	5	Business	Self-employment	
Nairobi	Female	Married/Pa	Primary	8	Business	Self-employment	
Nakuru	Female	Married/Pa	Secondary	4	None	Casual work	
Nakuru	Male	Married/Pa	Secondary	4	Business	Self-employment	
Nakuru	Female	Married/Pa	Primary	8	Business	Employment (private s	
Nakuru	Male	Married/Pa	Secondary	4	Casual Worker	Casual work	
Nairobi	Female	Single	Secondary	4	Professional	Self-emplo	Business
Kisumu	Male	Married/Pa	Primary	8	Casual Worker	Casual work	
Mombasa	Female	Married/Pa	Primary	1	Business	Casual work	
Kisumu	Female	Single	Secondary	1	None	None	
Nairobi	Female	Married/Pa	Secondary	2	None	Employment (private s	
Nairobi	Female	Single	Primary	7	Casual Worker	Casual work	
Mombasa	Female	Separated/	None		None	None	
Mombasa	Female	Married/Pa	Secondary	3	None	Self-employment	
Nairobi	Female	Single	Secondary	4	Casual Worker	Casual work	
Nakuru	Female	Married/Pa	Secondary	4	Business	Self-employment	
Nairobi	Male	Married/Pa	Primary	8	None	None	
Nairobi	Female	Married/Pa	Secondary	4	None	Employment (civil serv	
Mombasa	Female	Married/Pa	Primary	8	None	Casual work	
Kisumu	Female	Married/Pa	Primary	8	Business	Self-employment	
Kisumu	Female	Married/Pa	Secondary	2	Business	Self-employment	
Kisumu	Female	Widowed	Primary	7	Business	Self-employment	
Nakuru	Female	Married/Pa	Higher	3	None	Employment (private s	
Mombasa	Female	Married/Pa	Higher	4	Professional	Employment (civil serv	
Nairobi	Female	Married/Pa	Secondary	4	Professional	Self-employment	
Kisumu	Female	Married/Pa	Higher	3	Professional	Employment (private s	
Nakuru	Female	Married/Pa	Higher	2	None	Employment (private s	

Kisumu	Female	Widowed	None		Casual Worker	Casual work	
Mombasa	Male	Married/Pa	Primary	5	Casual Worker	Casual work	
Nairobi	Female	Separated/	Secondary	1	Casual Worker	Casual work	
Nairobi	Female	Married/Pa	Secondary	4	None	Employment (private s	
Nakuru	Female	Married/Pa	Primary	7	None	Casual work	
Nakuru	Female	Widowed	None		Business	Self-employment	
Mombasa	Female	Single	Secondary	4	Casual Worker	Self-employment	
Nairobi	Female	Married/Pa	Secondary	4	None	Employment (private s	
Nakuru	Female	Prefer not t	Secondary	4	None	Self-employment	
Nakuru	Male	Single	Secondary	4	None	Self-emplo	Farming
Nakuru	Female	Married/Pa	Secondary	4	None	Self-employment	
Kisumu	Female	Married/Pa	Secondary	4	Business	Self-employment	
Nairobi	Female	Married/Pa	Secondary	4	None	Casual work	
Nakuru	Female	Married/Pa	Primary	8	Casual Worker	Self-employment	
Nakuru	Female	Married/Pa	Higher	2	Casual Worker	Casual work	
Nakuru	Male	Single	Secondary	3	None	Self-employment	
Nairobi	Male	Married/Pa	Secondary	4	Janitorial/guard	Employment (civil serv	
Nairobi	Female	Married/Pa	Secondary	2	None	None	
Mombasa	Female	Married/Pa	Primary	8	Casual Worker	Casual work	
Kisumu	Female	Widowed	Primary	5	Casual Worker	Casual work	
Kisumu	Male	Single	Secondary	4	Student	Casual work	
Nairobi	Male	Single	Secondary	4	None	Self-employment	
Mombasa	Female	Single	Primary	8	None	Employment (private s	
Nairobi	Male	Single	Secondary	1	Casual Worker	Casual work	
Nairobi	Female	Married/Pa	Secondary	4	None	Casual work	
Kisumu	Female	Widowed	None		Casual Worker	Casual work	
Mombasa	Female	Married/Pa	None		None	Employment (private s	
Mombasa	Female	Single	Primary	8	None	Employment (private s	
Nakuru	Female	Married/Pa	Primary	7	Casual Worker	Employment (private s	
Nairobi	Female	Single	Secondary	1	Casual Worker	Casual work	
Kisumu	Male	Single	Secondary	4	Casual Worker	Casual work	
Nakuru	Female	Married/Pa	Secondary	4	Business	Self-employment	
Nairobi	Male	Married/Pa	Secondary	4	Casual Worker	Casual work	
Mombasa	Female	Married/Pa	Primary	8	None	Casual work	
Kisumu	Female	Married/Pa	Secondary	2	None	Self-employment	
Nairobi	Female	Married/Pa	Primary	8	Professional	Employment (private s	
Nairobi	Female	Married/Pa	Higher	2	None	Self-employment	
Nakuru	Female	Separated/	Primary	8	Casual Worker	Casual work	
Kisumu	Female	Married/Pa	Primary	8	Business	Self-employment	
Nairobi	Male	Married/Pa	Prefer not to answer		Casual Worker	Casual work	
Kisumu	Female	Married/Pa	Primary	8	Business	Self-employment	
Nairobi	Male	Married/Pa	Secondary	4	Janitorial/guard	Employment (civil serv	
Mombasa	Female	Married/Pa	Primary	8	Business	Casual work	
Nakuru	Male	Married/Pa	Secondary	4	Casual Worker	Casual work	
Nairobi	Female	Married/Pa	Secondary	4	None	None	
Mombasa	Female	Married/Pa	Secondary	4	None	Self-employment	
Mombasa	Female	Married/Pa	Primary	1	Business	Self-employment	
Kisumu	Female	Single	Secondary	4	None	Casual work	
Mombasa	Female	Separated/	Primary	4	Business	Self-employment	

Nairobi	Male	Married/Pa	Primary	8	Professional		Employment (private s
Nairobi	Female	Married/Pa	Primary	7	Casual Worker		None
Nairobi	Female	Married/Pa	Secondary	4	Business		Self-employment
Nairobi	Male	Married/Pa	Higher	2	Janitorial/guard		Employment (private s
Kisumu	Male	Married/Pa	Higher	4	Business		Self-employment
Kisumu	Female	Married/Pa	None		Business		Self-employment
Kisumu	Female	Married/Pa	Secondary	4	None		Self-employment
Kisumu	Male	Single	Secondary	4	None		Self-employment
Nakuru	Female	Married/Pa	Secondary	3	None		Employment (civil serv
Nairobi	Female	Married/Pa	Secondary	2	None		Casual work
Kisumu	Female	Married/Pa	Secondary	2	Business		Self-employment
Nairobi	Female	Single	Secondary	4	None		Casual work
Mombasa	Female	Married/Pa	Secondary	4	None		Casual work
Nakuru	Female	Married/Pa	Secondary	2	Business		Self-employment
Nairobi	Female	Married/Pa	Secondary	4	None		Self-employment
Mombasa	Female	Widowed	Primary	8	Business		Self-employment
Kisumu	Female	Single	Primary	7	None		Employment (private s
Kisumu	Female	Married/Pa	None		Business		Self-employment
Nairobi	Female	Widowed	Secondary	2	Casual Worker		Casual work
Mombasa	Female	Married/Pa	Primary	1	Business		Employment (private s
Nairobi	Female	Married/Pa	Primary	7	None		Self-employment
Nakuru	Female	Married/Pa	Secondary	4	Business		Self-employment
Nakuru	Male	Single	Higher	2	None		Employment (private s
Nakuru	Female	Separated/	Primary	8	Casual Worker		Casual work
Nairobi	Male	Single	Higher	4	Casual Worker		Casual work
Nakuru	Female	Married/Pa	Secondary	2	None		Employment (private s
Kisumu	Female	Married/Pa	Higher	4	Business		Self-employment
Mombasa	Female	Married/Pa	Primary	7	Casual Worker		Casual work
Nairobi	Female	Married/Pa	Secondary	4	None		Casual work
Nairobi	Female	Married/Pa	Primary	8	Other	Land Owne	Casual work
Nairobi	Female	Married/Pa	Primary	8	Business		Self-employment
Mombasa	Female	Married/Pa	Higher	0.5	None		Employment (private s
Kisumu	Female	Single	Secondary	4	None		Self-employment
Mombasa	Female	Separated/	Primary	1	None		Self-employment
Nakuru	Male	Married/Pa	Primary	7	Business		Self-employment
Kisumu	Male	Single	Secondary	4	None		Self-employment
Kisumu	Female	Married/Pa	Secondary	4	Business		Self-employment
Nakuru	Male	Married/Pa	Secondary	4	Business		Self-employment
Kisumu	Female	Married/Pa	Secondary	2	Business		Self-employment
Nakuru	Female	Separated/	Secondary	3	Other	Farmer	Self-employment
Nairobi	Female	Married/Pa	Primary	6	Casual Worker		Employment (private s
Nakuru	Female	Married/Pa	Secondary	3	Business		Self-employment
Mombasa	Female	Married/Pa	Primary	4	None		Casual work
Mombasa	Female	Single	Higher	3	Casual Worker		Casual work
Nairobi	Male	Married/Pa	Secondary	1	Janitorial/guard		Employment (private s
Nairobi	Female	Married/Pa	Primary	8	None		Casual work
Mombasa	Female	Married/Pa	Higher	2	Professional		Employment (civil serv
Nairobi	Male	Married/Pa	Primary	8	Casual Worker		Casual work
Kisumu	Female	Married/Pa	Secondary	4	None		Self-employment

Kisumu	Female	Married/Pa	Primary	8	None	Self-employment	
Mombasa	Female	Married/Pa	Primary	7	Business	Employment (private s	
Kisumu	Female	Married/Pa	Primary	5	Casual Worker	Casual work	
Nakuru	Female	Separated/	Primary	8	Casual Worker	Casual work	
Nairobi	Male	Single	Higher	1	Business	Self-employment	
Kisumu	Female	Married/Pa	Primary	8	Business	Self-employment	
Nakuru	Female	Single	Primary	7	None	Self-employment	
Nairobi	Female	Married/Pa	Secondary	4	None	None	
Kisumu	Female	Married/Pa	Secondary	4	Business	Self-employment	
Nakuru	Female	Separated/	Primary	7	Casual Worker	Casual work	
Kisumu	Female	Married/Pa	Secondary	3	Business	Self-employment	
Mombasa	Female	Married/Pa	None		None	Casual work	
Kisumu	Female	Widowed	Secondary	1	Business	Self-employment	
Kisumu	Female	Single	Higher	2	None	Self-employment	
Kisumu	Male	Married/Pa	Secondary	4	None	Self-employment	
Kisumu	Female	Widowed	None		None	Casual wor	Saloonist
Mombasa	Male	Widowed	Primary	8	Casual Worker	None	
Nakuru	Female	Married/Pa	Primary	8	Business	Self-employment	
Kisumu	Male	Married/Pa	Primary	8	Business	Self-employment	
Nakuru	Female	Married/Pa	Primary	5	None	Casual work	
Nakuru	Female	Single	Secondary	4	Casual Worker	Self-employment	
Nairobi	Female	Married/Pa	Higher	1	Student	Employment (private s	
Mombasa	Male	Married/Pa	Primary	8	Casual Worker	Self-employment	
Mombasa	Female	Married/Pa	Higher	3	None	Employment (private s	
Mombasa	Male	Single	Higher	3	None	Self-employment	
Nairobi	Female	Married/Pa	Secondary	4	None	Employment (private s	
Nairobi	Female	Married/Pa	Secondary	4	Casual Worker	Casual work	
Kisumu	Male	Married/Pa	Secondary	4	Business	Self-employment	
Nairobi	Female	Single	Secondary	1	Casual Worker	Self-employment	
Kisumu	Female	Married/Pa	Primary	8	None	Casual work	
Nairobi	Female	Married/Pa	Secondary	2	Business	Casual work	
Kisumu	Female	Married/Pa	Primary	8	Business	Self-employment	
Kisumu	Female	Married/Pa	Secondary	2	Business	Self-employment	
Nakuru	Female	Married/Pa	Higher	3	Business	Self-employment	
Kisumu	Female	Separated/	Primary	8	Casual Worker	Casual work	
Nakuru	Female	Married/Pa	Secondary	4	Business	Self-employment	
Kisumu	Female	Married/Pa	Secondary	4	None	Employment (private s	
Nairobi	Female	Married/Pa	Secondary	2	Business	Self-employment	
Mombasa	Male	Married/Pa	Primary	8	Casual Worker	Casual work	
Kisumu	Female	Separated/	Secondary	4	Business	Self-employment	
Nakuru	Female	Married/Pa	None		Casual Worker	Casual work	
Kisumu	Female	Married/Pa	Primary	8	None	Self-employment	
Kisumu	Female	Married/Pa	Secondary	4	None	Self-employment	
Nairobi	Female	Married/Pa	Secondary	1	None	None	
Kisumu	Female	Married/Pa	Secondary	4	Business	Self-employment	
Kisumu	Female	Married/Pa	Secondary	4	Business	Self-employment	
Nairobi	Female	Married/Pa	Secondary	4	None	Self-employment	
Nairobi	Female	Married/Pa	Primary	7	None	Self-employment	
Nairobi	Male	Single	Secondary	4	Student	None	

Kisumu	Female	Married/Pa	Secondary	4	Business		Employment (private s	
Nairobi	Female	Single	Higher	1	Student		Self-employment	
Kisumu	Female	Married/Pa	Higher	4	Professional		Employment (civil serv	
Kisumu	Female	Married/Pa	Secondary	4	None		Self-employment	
Nakuru	Male	Married/Pa	Secondary	4	Other	Farmer	Self-employment	
Mombasa	Female	Married/Pa	Secondary	4	Business		Self-employment	
Nairobi	Female	Married/Pa	Primary	7	Business		Employment (civil serv	
Mombasa	Female	Married/Pa	Primary	8	None		Self-employment	
Nairobi	Female	Married/Pa	Secondary	3	Casual Worker		Casual work	
Kisumu	Female	Married/Pa	Primary	8	Business		Self-employment	
Nairobi	Female	Prefer not t	Secondary	2	Business		Self-employment	
Nakuru	Female	Married/Pa	Primary	8	None		Casual work	
Nairobi	Female	Married/Pa	Secondary	2	None		Casual work	
Mombasa	Female	Married/Pa	Secondary	2	None		Casual work	
Kisumu	Female	Married/Pa	Secondary	4	None		Self-employment	
Nakuru	Female	Married/Pa	Secondary	4	Business		Self-employment	
Nakuru	Female	Married/Pa	Secondary	3	None		Casual work	
Nairobi	Female	Single	Primary	2	Casual Worker		Casual work	
Nakuru	Female	Married/Pa	Primary	8	None		Casual work	
Nakuru	Female	Married/Pa	Higher	2	Business		Self-employment	
Kisumu	Male	Married/Pa	Higher	4	Professional		Employment (private s	
Nairobi	Female	Married/Pa	Higher	2	None		Employment (private s	
Mombasa	Female	Married/Pa	Secondary	4	Business		Self-employment	
Nakuru	Female	Married/Pa	Secondary	4	None		Self-employment	
Kisumu	Female	Widowed	Primary	4	Casual Worker		Casual work	
Kisumu	Female	Married/Pa	Higher	3	None		Employment (private s	
Nakuru	Male	Married/Pa	Primary	8	Business		Self-employment	
Mombasa	Male	Married/Pa	Primary	8	Casual Worker		Casual work	
Kisumu	Female	Married/Pa	Primary	8	None		Employment (private s	
Mombasa	Female	Single	Secondary	4	None		Casual work	
Nairobi	Female	Married/Pa	Secondary	4	None		None	
Mombasa	Female	Single	Secondary	2	Casual Worker		Casual work	
Nakuru	Female	Separated/	Primary	7	Business		Self-emplo	Chikd /day
Nairobi	Female	Widowed	Secondary	2	None		Self-emplo	landlord
Mombasa	Female	Married/Pa	Primary	4	Casual Worker		Self-employment	
Kisumu	Female	Married/Pa	Primary	8	None		Self-employment	
Nakuru	Male	Single	Secondary	4	Casual Worker		Employment (private s	
Nairobi	Male	Married/Pa	Secondary	4	Casual Worker		Casual work	
Nairobi	Female	Married/Pa	Secondary	4	None		Casual work	
Nakuru	Female	Single	Secondary	4	Business		Self-employment	
Mombasa	Female	Single	None		None		Employment (private s	
Kisumu	Female	Married/Pa	Secondary	4	Business		Self-employment	
Mombasa	Female	Married/Pa	Primary	8	Business		Employment (civil serv	
Kisumu	Female	Single	Secondary	4	None		Casual work	
Nairobi	Male	Married/Pa	Primary	7	None		None	
Kisumu	Female	Married/Pa	Primary	8	Business		Self-employment	
Nairobi	Male	Married/Pa	Primary	7	Business		Casual work	
Kisumu	Female	Married/Pa	Secondary	2	None		Employment (private s	
Nairobi	Female	Married/Pa	Secondary	8	Business		Self-employment	

Nairobi	Female	Married/Pa	Higher	2	None	Employment (private s
Mombasa	Female	Married/Pa	Primary	7	None	Casual work
Kisumu	Female	Married/Pa	Secondary	2	None	Self-employment
Mombasa	Female	Married/Pa	Secondary	4	None	None
Mombasa	Female	Single	Secondary	1	None	Self-employment
Nairobi	Male	Married/Pa	Secondary	4	Casual Worker	Casual work
Kisumu	Female	Single	Primary	8	Casual Worker	Casual work
Mombasa	Female	Married/Pa	Primary	1	Business	Casual work
Nakuru	Female	Married/Pa	Primary	8	None	Self-employment
Kisumu	Male	Married/Pa	Primary	8	Business	Self-employment
Nairobi	Female	Married/Pa	Primary	8	Business	Employment (private s
Kisumu	Male	Married/Pa	Secondary	4	Business	Employment (private s
Nairobi	Female	Married/Pa	Higher	2	Professional	Employment (private s
Nairobi	Male	Married/Pa	Secondary	4	Professional	Casual work
Nakuru	Female	Married/Pa	Primary	4	Business	Casual work
Kisumu	Female	Widowed	None		None	Self-employment
Mombasa	Female	Married/Pa	Primary	8	None	Casual work
Kisumu	Female	Married/Pa	Primary	8	None	Employment (private s
Mombasa	Female	Married/Pa	Secondary	4	Business	Self-employment
Nairobi	Female	Married/Pa	Primary	8	Casual Worker	Casual work
Nairobi	Female	Single	Higher	4	Business	Self-employment
Kisumu	Female	Widowed	Secondary	4	Business	Self-employment
Nakuru	Female	Separated/	Primary	8	Business	Self-employment
Mombasa	Female	Married/Pa	Primary	8	None	Casual work
Nairobi	Female	Married/Pa	Primary	6	Casual Worker	Casual work
Nakuru	Female	Married/Pa	Primary	8	Business	Self-employment
Nairobi	Female	Married/Pa	Secondary	4	Business	Self-employment
Nakuru	Female	Married/Pa	Primary	8	None	Self-employment
Kisumu	Female	Widowed	Secondary	2	Business	Self-employment
Nairobi	Male	Married/Pa	Primary	8	Casual Worker	Casual work
Kisumu	Female	Married/Pa	Secondary	2	None	Self-employment
Nairobi	Female	Married/Pa	Secondary	4	None	Casual work
Mombasa	Female	Married/Pa	Primary	8	None	Casual work
Kisumu	Male	Single	Higher	2	Student	Self-employment
Nairobi	Female	Married/Pa	Secondary	3	None	Casual work
Kisumu	Female	Married/Pa	Primary	7	None	Casual work
Mombasa	Female	Widowed	Primary	5	None	Casual work
Nakuru	Female	Married/Pa	Primary	8	Business	Self-employment
Kisumu	Female	Married/Pa	Primary	6	None	Self-employment
Nakuru	Male	Married/Pa	Higher	2	Casual Worker	Casual work
Kisumu	Female	Married/Pa	Secondary	4	None	Casual work
Mombasa	Male	Married/Pa	Secondary	1	Casual Worker	Self-employment
Kisumu	Male	Married/Pa	Secondary	4	Casual Worker	Casual work
Nairobi	Female	Single	Primary	7	None	Self-employment
Nairobi	Female	Married/Pa	Primary	8	None	None
Mombasa	Female	Married/Pa	Secondary	4	None	Casual work
Mombasa	Female	Married/Pa	Primary	2	Business	Employment (private s
Nakuru	Female	Married/Pa	Secondary	4	Casual Worker	Self-employment
Nairobi	Female	Married/Pa	Primary	8	Casual Worker	Self-employment

Nakuru	Female	Married/Pa	Secondary	4	None	Self-employment
Nairobi	Male	Single	Secondary	4	Student	Employment (private s
Mombasa	Female	Married/Pa	Primary	8	Business	Self-employment
Nakuru	Female	Married/Pa	Primary	8	None	Casual work
Nairobi	Female	Married/Pa	Secondary	4	Casual Worker	Employment (civil serv
Nairobi	Male	Married/Pa	Higher	2	Business	Self-employment
Mombasa	Female	Single	Primary	8	None	Employment (private s
Nairobi	Male	Single	Higher	2	Casual Worker	Employment (private s
Mombasa	Female	Widowed	Primary	8	None	None
Nairobi	Female	Married/Pa	Secondary	1	None	None
Mombasa	Male	Single	Primary	5	None	Casual work
Kisumu	Male	Married/Pa	Primary	8	Business	Self-employment
Kisumu	Female	Married/Pa	Primary	8	None	Self-employment
Nakuru	Female	Married/Pa	Primary	8	Business	Self-employment
Kisumu	Female	Single	Primary	8	Casual Worker	Casual work
Kisumu	Female	Single	Secondary	3	None	Self-employment
Mombasa	Female	Married/Pa	Secondary	7	None	Self-employment
Kisumu	Female	Married/Pa	Secondary	4	None	Self-employment
Mombasa	Female	Widowed	None		None	Casual work
Nakuru	Male	Married/Pa	Higher	2	Casual Worker	Casual work
Nairobi	Male	Single	Secondary	4	Casual Worker	Casual work
Mombasa	Male	Married/Pa	None		Casual Worker	Self-employment
Kisumu	Female	Married/Pa	Primary	8	None	Self-employment
Nakuru	Male	Married/Pa	Secondary	4	Casual Worker	Employment (private s
Mombasa	Male	Married/Pa	Primary	8	Janitorial/guard	Casual work
Kisumu	Female	Married/Pa	Secondary	2	None	Self-employment
Kisumu	Male	Married/Pa	Primary	8	Business	Self-employment
Nakuru	Female	Married/Pa	Primary	8	None	Self-employment
Nairobi	Female	Married/Pa	Secondary	1	None	Casual work
Nakuru	Female	Married/Pa	Secondary	4	Business	Employment (private s
Mombasa	Female	Married/Pa	Secondary	1	Business	Casual work
Kisumu	Female	Married/Pa	Primary	2	Business	Self-employment
Nairobi	Female	Married/Pa	Secondary	4	None	Employment (private s
Nairobi	Female	Married/Pa	Secondary	4	Casual Worker	Casual work
Kisumu	Female	Widowed	Secondary	4	None	Self-employment
Nairobi	Female	Married/Pa	Secondary	4	Business	Self-employment
Kisumu	Female	Married/Pa	Higher	2	Business	Self-employment
Kisumu	Female	Married/Pa	Secondary	4	None	Casual work
Nairobi	Female	Married/Pa	Secondary	4	None	Employment (civil serv
Nakuru	Female	Separated/	Primary	8	Casual Worker	Casual work
Nairobi	Female	Married/Pa	Secondary	4	Business	Self-employment
Kisumu	Female	Widowed	Secondary	4	Business	Self-employment
Nakuru	Female	Married/Pa	Secondary	2	Business	Self-employment
Kisumu	Male	Single	Secondary	4	Casual Worker	Casual work
Kisumu	Female	Married/Pa	Primary	8	Business	Self-employment
Nakuru	Female	Married/Pa	Primary	8	Business	Self-employment
Kisumu	Male	Married/Pa	Primary	8	Casual Worker	Casual work
Nairobi	Female	Married/Pa	Secondary	4	None	Employment (private s
Nairobi	Male	Married/Pa	Secondary	4	Casual Worker	Employment (private s

Kisumu	Female	Widowed	Secondary	8	None	Self-employment	
Kisumu	Female	Separated/	Secondary	3	Casual Worker	Self-employment	
Kisumu	Female	Married/Pa	Primary	8	None	Casual work	
Kisumu	Female	Married/Pa	Primary	8	None	Self-employment	
Kisumu	Female	Married/Pa	Primary	8	Business	Self-employment	
Kisumu	Female	Separated/	Primary	4	Business	Self-employment	
Nairobi	Female	Married/Pa	Secondary	4	None	Employment (civil serv	
Kisumu	Female	Married/Pa	Secondary	2	Casual Worker	Casual work	
Nairobi	Female	Married/Pa	Primary	8	Casual Worker	Casual work	
Nairobi	Female	Single	Primary	7	None	Self-employment	
Mombasa	Female	Separated/	None		None	None	
Kisumu	Female	Married/Pa	Primary	8	Business	Self-employment	
Nairobi	Female	Married/Pa	Secondary	4	Business	Casual work	
Nakuru	Female	Married/Pa	Secondary	4	None	Employment (private s	
Kisumu	Male	Single	Secondary	4	None	Casual work	
Kisumu	Female	Married/Pa	Secondary	2	Business	Casual wo	Sell French
Kisumu	Female	Single	Secondary	1	None	Casual work	
Nairobi	Female	Married/Pa	Primary	4	None	None	
Nairobi	Male	Married/Pa	Higher	2	Casual Worker	Casual work	
Nakuru	Female	Separated/	Primary	8	Business	Self-employment	
Kisumu	Female	Single	Primary	8	Business	Self-employment	
Mombasa	Female	Married/Pa	Primary	6	None	Casual work	
Nairobi	Male	Married/Pa	Primary	8	Casual Worker	Casual work	
Nairobi	Male	Single	Primary	8	Casual Worker	Casual work	
Nakuru	Female	Married/Pa	Primary	8	Business	Employment (private s	
Nakuru	Female	Widowed	Secondary	2	Business	Self-emplo	Day care
Nairobi	Female	Married/Pa	Secondary	4	None	Casual work	
Kisumu	Female	Widowed	Primary	7	Casual Worker	Casual work	
Kisumu	Male	Single	Higher	3	None	None	
Kisumu	Female	Single	Higher	1	None	Self-employment	
Mombasa	Female	Married/Pa	Primary	8	Casual Worker	Casual work	
Nakuru	Female	Married/Pa	Secondary	2	Business	Casual work	
Nakuru	Female	Married/Pa	Secondary	4	None	Self-employment	
Nairobi	Male	Married/Pa	Secondary	4	Professional	Employment (private s	
Nakuru	Female	Single	Secondary	4	None	Self-employment	
Kisumu	Female	Separated/	Primary	7	Business	Self-employment	
Nakuru	Male	Married/Pa	Primary	8	Business	Casual work	
Nairobi	Female	Married/Pa	Primary	8	Casual Worker	Casual work	
Kisumu	Male	Married/Pa	Secondary	2	Business	Self-employment	
Mombasa	Female	Widowed	None		None	Self-employment	
Kisumu	Female	Married/Pa	Primary	8	Business	Self-employment	
Mombasa	Female	Married/Pa	Secondary	2	None	Casual work	
Mombasa	Female	Widowed	Primary	7	Business	Self-employment	
Nakuru	Male	Married/Pa	Secondary	4	Business	Self-employment	
Kisumu	Female	Single	Secondary	2	None	Self-employment	
Kisumu	Female	Married/Pa	Primary	6	None	Self-employment	
Mombasa	Female	Married/Pa	Primary	7	None	Self-employment	
Nakuru	Female	Married/Pa	Secondary	4	None	Employment (private s	
Nakuru	Male	Married/Pa	Higher	1	Business	Self-employment	

Nakuru	Female	Single	Higher	2	Janitorial/guard	Self-employment	
Nairobi	Female	Married/Pa	Primary	8	None	Casual work	
Nakuru	Female	Single	Secondary	4	Business	Self-employment	
Kisumu	Female	Widowed	None		Business	Self-employment	
Nakuru	Male	Married/Pa	Higher	3	Professional	Employment (private s	
Kisumu	Female	Married/Pa	Secondary	4	Casual Worker	Casual work	
Nakuru	Female	Married/Pa	Primary	8	None	Self-employment	
Nakuru	Female	Married/Pa	Secondary	3	None	Self-employment	
Kisumu	Female	Married/Pa	Primary	7	Business	Self-employment	
Kisumu	Female	Single	Primary	8	None	Self-employment	
Kisumu	Female	Married/Pa	Primary	8	Business	Self-employment	
Nairobi	Female	Married/Pa	Secondary	4	Casual Worker	Casual work	
Mombasa	Female	Married/Pa	Higher	2	None	None	
Kisumu	Female	Married/Pa	Secondary	4	Business	Self-employment	
Nairobi	Female	Married/Pa	Primary	8	None	None	
Kisumu	Female	Married/Pa	Secondary	4	Business	Self-employment	
Kisumu	Female	Single	Primary	8	Business	Self-employment	
Kisumu	Female	Married/Pa	Secondary	4	Business	Employment (private s	
Mombasa	Female	Married/Pa	Higher	0.5	None	Employment (civil serv	
Mombasa	Female	Widowed	Primary	8	Casual Worker	Casual work	
Nairobi	Female	Married/Pa	Primary	8	None	None	
Kisumu	Female	Single	Higher	4	None	Self-employment	
Nairobi	Male	Married/Pa	Higher	4	Professional	Employment (private s	
Nairobi	Female	Married/Pa	Secondary	1	Business	Self-employment	
Nakuru	Female	Married/Pa	Secondary	2	None	Employment (civil serv	
Mombasa	Male	Single	Higher	2	None	None	
Nairobi	Male	Separated/	Primary	6	Casual Worker	Self-employment	
Nairobi	Female	Single	Higher	1	None	Family Sup	Support fro
Nairobi	Female	Single	Secondary	4	None	Self-employment	
Mombasa	Female	Married/Pa	Primary	3	Business	Self-employment	
Kisumu	Female	Married/Pa	Primary	8	Business	Self-employment	
Nairobi	Female	Married/Pa	Secondary	4	None	Self-employment	
Mombasa	Female	Widowed	None		None	Casual work	
Kisumu	Female	Married/Pa	Primary	8	None	Employment (private s	
Mombasa	Male	Prefer not t	Primary	6	Business	Casual work	
Nairobi	Female	Married/Pa	Primary	8	Casual Worker	Casual work	
Nairobi	Female	Married/Pa	Primary	7	Casual Worker	Casual work	
Mombasa	Female	Married/Pa	Primary	8	None	Employment (private s	
Mombasa	Male	Single	Primary	8	None	Casual work	
Kisumu	Female	Single	Higher	2	Business	Self-employment	
Kisumu	Female	Married/Pa	Secondary	4	Business	Self-employment	
Nairobi	Female	Married/Pa	Secondary	4	None	Casual work	
Kisumu	Female	Married/Pa	Secondary	4	None	Self-employment	
Nakuru	Female	Widowed	Primary	4	Business	Self-employment	
Nakuru	Female	Married/Pa	Secondary	1	Business	Self-employment	
Nakuru	Female	Separated/	Secondary	4	Janitorial/guard	Employment (private s	
Nairobi	Female	Married/Pa	Secondary	4	None	Self-employment	
Mombasa	Male	Married/Pa	Higher	2	None	Casual wo	Hotelier
Nakuru	Female	Married/Pa	Secondary	4	Business	Self-employment	

Mombasa	Female	Single	None		None		Employment (private s
Nairobi	Female	Married/Pa	Secondary	4	Business		Employment (private s
Nakuru	Female	Married/Pa	Primary	8	Business		Self-employment
Nairobi	Male	Married/Pa	Primary	7	Janitorial/guard		Employment (private s
Kisumu	Male	Married/Pa	Secondary	4	Business		Self-employment
Mombasa	Male	Married/Pa	Secondary	4	Casual Worker		Casual work
Kisumu	Female	Married/Pa	Primary	8	Business		Self-employment
Mombasa	Female	Married/Pa	Primary	8	Business		Self-employment
Nairobi	Male	Married/Pa	Primary	8	Professional		Casual work
Nairobi	Female	Married/Pa	Primary	7	Other	CHV	Casual work
Mombasa	Female	Single	Primary	7	Business		Self-employment
Nairobi	Male	Separated/	Secondary	4	Casual Worker		Casual work
Mombasa	Male	Married/Pa	Higher	2	Casual Worker		Self-employment
Nairobi	Female	Married/Pa	Secondary	2	None		Casual work
Nakuru	Female	Married/Pa	Primary	8	Business		Self-employment
Nakuru	Female	Married/Pa	Secondary	4	Business		Casual work
Nakuru	Female	Married/Pa	Higher	2.5	Business		Self-employment
Nakuru	Female	Married/Pa	Secondary	4	Business		Self-employment
Nairobi	Female	Married/Pa	Primary	8	None		Casual work
Mombasa	Male	Separated/	Primary	8	Casual Worker		Casual work
Nakuru	Male	Single	Primary	8	Casual Worker		Self-employment
Nairobi	Female	Married/Pa	Secondary	4	Casual Worker		Casual work
Nakuru	Female	Married/Pa	Secondary	2	Business		Self-employment
Mombasa	Female	Prefer not t	Prefer not to answer		None		Casual work
Nakuru	Female	Married/Pa	Secondary	2	None		Self-employment
Nakuru	Female	Married/Pa	Secondary	1	None		Self-employment
Nakuru	Female	Married/Pa	Secondary	4	Casual Worker		Employment (civil serv
Kisumu	Female	Married/Pa	Primary	8	Business		Self-employment
Nakuru	Female	Married/Pa	Primary	8	Business		Casual work
Nairobi	Female	Married/Pa	Secondary	4	None		Casual work
Kisumu	Female	Married/Pa	Primary	8	Casual Worker		Casual work
Kisumu	Female	Married/Pa	Primary	7	None		Casual work
Nairobi	Female	Married/Pa	Primary	8	None		Casual work
Nairobi	Male	Married/Pa	Primary	8	Casual Worker		Casual work
Kisumu	Female	Widowed	None		Casual Worker		Casual work
Nakuru	Female	Married/Pa	Higher	2	Casual Worker		Employment (private s
Kisumu	Female	Single	Higher	4	Professional		Employment (private s
Mombasa	Female	Single	Secondary	4	None		Employment (private s
Kisumu	Male	Married/Pa	Secondary	4	None		Self-employment
Mombasa	Female	Single	Higher	4	None		None
Kisumu	Female	Married/Pa	Primary	4	Business		Self-employment
Kisumu	Female	Married/Pa	Primary	6	None		Self-employment
Nairobi	Male	Single	Secondary	4	Casual Worker		Casual work
Kisumu	Female	Married/Pa	Primary	8	Business		Self-employment
Kisumu	Male	Single	Secondary	4	None		Employment (private s
Nakuru	Female	Married/Pa	Primary	8	None		Casual work
Kisumu	Female	Married/Pa	Secondary	3	Business		Self-employment
Mombasa	Male	Single	None		Casual Worker		Casual work
Mombasa	Female	Married/Pa	None		Business		Employment (private s

Nairobi	Female	Married/Pa	Secondary	3	None		Casual work	
Nakuru	Female	Widowed	Primary	4	Business		Self-employment	
Nairobi	Male	Single	Secondary	4	Casual Worker		Casual work	
Mombasa	Female	Married/Pa	Secondary	4	Business		Self-employment	
Kisumu	Female	Married/Pa	Secondary	4	None		Employment (civil serv	
Nakuru	Female	Married/Pa	Secondary	2	None		Casual work	
Nairobi	Female	Married/Pa	Secondary	3	None		Self-employment	
Nakuru	Female	Married/Pa	Secondary	4	None		Self-employment	
Mombasa	Female	Single	Secondary	4	None		Casual work	
Nakuru	Female	Married/Pa	Primary	7	Casual Worker		Casual work	
Mombasa	Female	Widowed	Primary	6	Business		Self-employment	
Nakuru	Female	Married/Pa	Secondary	4	Business		Self-employment	
Kisumu	Female	Married/Pa	Secondary	2	None		Employment (private s	
Nairobi	Female	Single	Secondary	4	None		None	
Nairobi	Female	Married/Pa	Primary	6	None		Self-employment	
Nairobi	Female	Married/Pa	Secondary	4	None		None	
Nairobi	Female	Married/Pa	Primary	7	None		None	
Kisumu	Female	Married/Pa	Primary	8	Business		Self-employment	
Nakuru	Female	Married/Pa	Secondary	3	Business		Self-employment	
Mombasa	Female	Married/Pa	Secondary	4	None		Self-employment	
Kisumu	Female	Married/Pa	Higher	2	None		Self-employment	
Nairobi	Female	Married/Pa	Higher	2	None		None	
Nakuru	Female	Married/Pa	Secondary	1	None		Self-employment	
Nairobi	Female	Married/Pa	Secondary	4	None		None	
Nairobi	Female	Married/Pa	Higher	2	Casual Worker		Casual work	
Nairobi	Male	Single	Secondary	4	None		None	
Nakuru	Female	Married/Pa	Secondary	3	Casual Worker		Casual work	
Kisumu	Female	Married/Pa	Secondary	3	None		Casual work	
Mombasa	Female	Married/Pa	Secondary	4	None		Self-employment	
Kisumu	Female	Married/Pa	Secondary	4	None		Self-employment	
Mombasa	Female	Single	Primary	8	None		None	
Kisumu	Female	Married/Pa	Secondary	4	None		Self-employment	
Nakuru	Female	Married/Pa	Secondary	4	Janitorial/guard		Employment (private s	
Nairobi	Female	Married/Pa	Secondary	3	None		Self-employment	
Kisumu	Female	Married/Pa	Primary	8	Business		Self-employment	
Kisumu	Female	Married/Pa	Secondary	4	None		Employment (private s	
Kisumu	Female	Widowed	Primary	5	Business		Self-employment	
Nairobi	Male	Married/Pa	Secondary	4	Janitorial/guard		Employment (private s	
Kisumu	Male	Single	Secondary	4	Business		Self-employment	
Nakuru	Female	Married/Pa	Primary	6	Business		Employment (private s	
Kisumu	Female	Single	Primary	8	None		Employment (private s	
Nakuru	Female	Married/Pa	Secondary	4	Business		Self-employment	
Kisumu	Female	Married/Pa	Secondary	4	Business		Self-employment	
Nairobi	Female	Married/Pa	Secondary	4	None		Self-employment	
Kisumu	Female	Widowed	Primary	7	Business		Self-employment	
Nakuru	Female	Separated/	Secondary	4	None		Self-emplo	Farming
Nairobi	Male	Married/Pa	Primary	8	Other	NBS	Employment (civil serv	
Kisumu	Female	Married/Pa	Primary	8	Business		Self-employment	
Nakuru	Female	Married/Pa	Secondary	4	None		Self-employment	

Kisumu	Male	Married/Pa	Higher	4	Professional	Self-employment
Mombasa	Female	Married/Pa	Primary	6	None	Casual work
Kisumu	Female	Married/Pa	Secondary	3	Business	Self-employment
Kisumu	Male	Single	Secondary	4	Casual Worker	Casual work
Nairobi	Female	Married/Pa	Higher	4	Business	Self-employment
Mombasa	Female	Married/Pa	Higher	2	None	Casual work
Nairobi	Male	Single	Secondary	4	Casual Worker	Casual work
Nakuru	Male	Married/Pa	Secondary	4	Janitorial/guard	Employment (private s
Nairobi	Female	Single	Secondary	4	None	None
Nairobi	Female	Married/Pa	Primary	8	None	Self-employment
Kisumu	Female	Married/Pa	Primary	8	Business	Employment (private s
Kisumu	Female	Widowed	Primary	4	None	None
Nairobi	Female	Married/Pa	Secondary	2	None	Employment (private s
Nairobi	Female	Widowed	Primary	4	None	Family Sup Support fro
Nairobi	Female	Married/Pa	Secondary	1	None	Casual work
Kisumu	Male	Married/Pa	Secondary	4	Professional	Employment (private s
Mombasa	Male	Married/Pa	None		Casual Worker	Casual work
Kisumu	Female	Married/Pa	Higher	3	Business	Self-employment
Kisumu	Female	Married/Pa	Secondary	4	None	Self-employment
Mombasa	Male	Single	Secondary	4	Casual Worker	Casual work
Kisumu	Female	Married/Pa	Primary	8	Casual Worker	Casual work
Nairobi	Female	Married/Pa	Primary	8	Casual Worker	Casual work
Mombasa	Female	Married/Pa	None		Business	Self-employment
Nakuru	Female	Single	Secondary	4	None	Self-employment
Nairobi	Female	Married/Pa	Secondary	4	None	Casual work
Nakuru	Female	Married/Pa	Secondary	4	Business	Self-employment
Mombasa	Male	Married/Pa	Primary	8	None	None
Kisumu	Female	Married/Pa	Secondary	1	Business	Self-employment
Mombasa	Female	Married/Pa	Primary	8	None	Employment (private s
Kisumu	Female	Married/Pa	Primary	4	Business	Self-employment
Nairobi	Female	Separated/	Primary	8	Casual Worker	Casual work
Kisumu	Female	Married/Pa	Higher	3	Casual Worker	Self-employment
Kisumu	Female	Widowed	Secondary	4	Business	Self-employment
Kisumu	Female	Married/Pa	Secondary	1	Business	Self-employment
Nairobi	Female	Married/Pa	Secondary	4	Professional	Self-employment
Nairobi	Female	Married/Pa	Secondary	4	Professional	Self-employment
Mombasa	Female	Married/Pa	Primary	6	Casual Worker	Self-employment
Nakuru	Female	Single	Secondary	2	Professional	Employment (private s
Nakuru	Female	Married/Pa	Secondary	1	Business	Employment (private s
Nairobi	Female	Married/Pa	Secondary	2	Casual Worker	Casual work
Kisumu	Female	Married/Pa	Secondary	1	Casual Worker	Employment (private s
Kisumu	Female	Widowed	Primary	4	None	Self-employment
Nakuru	Female	Married/Pa	Higher	2	None	Employment (private s
Nairobi	Female	Married/Pa	Secondary	4	None	Employment (private s
Nakuru	Female	Married/Pa	Primary	7	None	Casual work
Nakuru	Female	Married/Pa	Secondary	1	None	Casual work
Mombasa	Male	Single	Secondary	4	Casual Worker	Casual work
Nairobi	Female	Single	Higher	1	None	Employment (civil serv
Kisumu	Female	Married/Pa	Secondary	4	None	Employment (private s

Kisumu	Female	Married/Pa	Primary	8	None	Employment (private s	
Nakuru	Female	Married/Pa	Primary	8	None	Self-employment	
Kisumu	Female	Married/Pa	Secondary	5	None	Self-employment	
Kisumu	Female	Married/Pa	Primary	8	None	Self-employment	
Nairobi	Female	Married/Pa	Secondary	4	None	Casual work	
Nakuru	Female	Separated/	Primary	8	Casual Worker	Casual work	
Nakuru	Male	Married/Pa	Primary	4	Business	Self-employment	
Kisumu	Female	Widowed	None		None	Self-employment	
Nairobi	Male	Married/Pa	Higher	2	Casual Worker	Casual work	
Kisumu	Female	Married/Pa	Secondary	4	Business	Self-employment	
Mombasa	Female	Married/Pa	Primary	6	None	Self-employment	
Mombasa	Male	Single	Primary	8	Casual Worker	Casual work	
Mombasa	Female	Married/Pa	Primary	8	None	Self-employment	
Mombasa	Female	Married/Pa	Primary	4	None	Self-employment	
Mombasa	Female	Married/Pa	Primary	3	None	Self-employment	
Kisumu	Female	Widowed	None		None	Family Sup	Brother and
Nairobi	Male	Single	Primary	8	Casual Worker	Casual work	
Nakuru	Female	Married/Pa	Primary	8	None	Self-employment	
Kisumu	Female	Married/Pa	Higher	3	Professional	Employment (civil serv	
Kisumu	Male	Married/Pa	Higher	4	Casual Worker	Self-employment	
Kisumu	Female	Married/Pa	Primary	8	Business	Self-employment	
Nairobi	Female	Single	Secondary	4	Casual Worker	Casual work	
Nairobi	Female	Single	Secondary	2	None	Employment (private s	
Mombasa	Female	Widowed	None		None	Casual work	
Nairobi	Female	Single	Primary	6	Casual Worker	Casual work	

q9	q10	q11	q12	q13a	q13b	q14	q15	q16
1	0	1	1-2 years	A block with houses		Rent	1200	1
4	2	1	Above 5 ye	A block with houses		Own		2
2	0	0	Above 5 ye	Compound shared wit		Rent	4000	1
1	0	0	1-2 years	A block with houses		Rent	1300	1
3	1	0	Above 5 ye	House with a private y		Own		2
2	1	1	Above 5 ye	Compound shared wit		Rent	4000	1
2	2	0	Above 5 ye	A block with houses		Own		7
4	5	1	Above 5 ye	House without compo		Rent	1500	1
2	0	1	Above 5 ye	A block with houses		Rent	2000	1
2	2	1	Above 5 ye	Compound shared wit		Rent	5000	2
1	0	0	Above 5 ye	Compound shared wit		Rent	2500	1
2	2	0	Above 5 ye	Compound shared wit		Rent	3000	1
4	0	0	Since birth	House without compo		Rent	6000	2
1	0	0	1-2 years	Compound shared wit		Rent	2300	1
2	1	0	1-2 years	Compound shared wit		Rent	2500	1
1	0	0	3-5 years	House without compo		Rent	5000	1
2	0	0	Less than	Compound shared wit		Rent	3000	1
2	0	0	1-2 years	Room in a multi-unit b		Rent	1000	1
2	1	1	3-5 years	Compound shared wit		Rent	7500	1
3	2	0	Since birth	House without compo		Own		4
1	2	0	1-2 years	A block with houses		Rent	2500	1
1	0	0	Above 5 ye	Compound shared wit		Rent	2500	1
2	0	0	Above 5 ye	A block with houses		Own		1
3	0	0	Above 5 ye	Room in a multi-unit b		Own		2
3	1	1	Above 5 ye	Compound shared wit		Rent	4500	3
1	0	0	1-2 years	Compound shared wit		Rent	2000	1
2	0	2	Above 5 ye	A block with houses		Live here for free		1
1	0	0	Above 5 ye	Compound shared wit		Rent	6000	1
2	0	1	3-5 years	A block with houses		Rent	2500	2
6	3	0	Above 5 ye	House without compo		Own		6
3	1	0	Less than	A block with houses		Rent	2000	1
6	0	0	3-5 years	House without compo		Own		3
1	1	1	Above 5 ye	Compound shared wit		Rent	2000	1
2	0	0	Less than	Room in a multi-unit b		Rent	1500	1
2	0	0	1-2 years	Room in a multi-unit b		Rent	1500	1
3	0	1	1-2 years	Room in a multi-unit b		Own		1
1	1	1	3-5 years	Compound shared wit		Rent	1800	1
7	0	0	Above 5 ye	House without compo		Own		2
2	3	1	Above 5 ye	Compound shared wit		Live here for free		1
2	0	1	1-2 years	House without compo		Rent	8500	2
1	3	0	Above 5 ye	A block with houses		Rent	3000	3
2	0	1	1-2 years	A block with houses		Rent	1500	1
7	0	0	Above 5 ye	House without compo		Own		4
4	0	0	Above 5 ye	House without compo		Own		6
1	2	0	1-2 years	Compound shared wit		Rent	2500	1
2	2	2	1-2 years	Compound shared wit		Rent	5000	3
2	2	0	1-2 years	Compound shared wit		Rent	12000	1
1	0	4	Above 5 ye	Room in a multi-unit b		Live here for free		1

1	0	0	Above 5 ye	A block with houses	Rent	2500	1
3	1	0	Less than :	A block with houses	Rent	2500	1
7	0	0	Above 5 ye	House without compoı	Own		7
2	3	1	1-2 years	House without compoı	Own		4
11	0	6	Above 5 ye	House without compoı	Own		5
3	1	0	Above 5 ye	Room in a multi-unit b	Rent	3500	1
3	1	2	Above 5 ye	House without compoı	Own		2
2	5	1	Above 5 ye	House with a private y	Own		6
3	2	0	Above 5 ye	House with a private y	Own		2
2	3	0	Above 5 ye	House without compoı	Own		3
6	0	0	Above 5 ye	House without compoı	Own		1
2	2	2	3-5 years	Compound shared wit	Rent	1500	2
2	0	2	3-5 years	A block with houses	Rent	2500	2
1	5	5	Above 5 ye	House without compoı	Live here for free		4
3	0	0	1-2 years	House without compoı	Own		2
1	3	1	Less than :	Compound shared wit	Rent	10000	4
3	3	1	Above 5 ye	Compound shared wit	Rent	6000	3
2	0	1	Above 5 ye	Compound shared wit	Own		6
2	0	0	Less than :	Compound shared wit	Rent	2200	1
4	3	0	Above 5 ye	Compound shared wit	Rent	1500	1
2	1	0	1-2 years	Compound shared wit	Rent	3000	1
2	2	0	Above 5 ye	Compound shared wit	Rent	3000	1
2	0	0	Less than :	A block with houses	Rent	3150	1
3	3	2	Above 5 ye	House with a private y	Own		3
3	0	1	Less than :	Compound shared wit	Rent	3500	1
3	0	1	Less than :	House without compoı	Own		2
2	0	1	Above 5 ye	A block with houses	Rent	1500	1
2	0	1	3-5 years	Compound shared wit	Rent	3000	1
3	0	0	3-5 years	Room in a multi-unit b	Rent	1500	1
2	1	0	1-2 years	A block with houses	Rent	1000	1
3	2	1	Above 5 ye	House without compoı	Own		1
2	0	1	Less than :	A block with houses	Rent	1000	1
6	0	0	Above 5 ye	House without compoı	Own		7
3	2	1	Above 5 ye	Compound shared wit	Rent	5000	3
2	2	1	Above 5 ye	A block with houses	Own		2
3	4	1	Since birth	House with a private y	Own		4
3	4	3	1-2 years	Compound shared wit	Rent	1500	1
1	0	0	1-2 years	Room in a multi-unit b	Rent	2000	1
2	2	1	Less than :	Compound shared wit	Rent	3500	1
1	2	0	Above 5 ye	House without compoı	Own		2
2	2	1	Above 5 ye	Compound shared wit	Rent	4500	1
2	0	0	3-5 years	Compound shared wit	Own		3
3	3	0	Above 5 ye	Compound shared wit	Own		2
2	0	1	Above 5 ye	House without compoı	Own		2
4	6	1	Above 5 ye	House without compoı	Own		3
2	1	0	3-5 years	Compound shared wit	Rent	2000	1
2	4	1	Above 5 ye	A block with houses	Own		2
5	0	0	Above 5 ye	House without compoı	Own		1
2	0	1	3-5 years	Compound shared wit	Rent	2500	1

2	1	1	Less than :	Compound shared wit	Rent	4000	1
1	0	0	1-2 years	House without compo	Live here for free		1
1	1	0	1-2 years	A block with houses	Rent	1000	1
2	1	1	3-5 years	A block with houses	Rent	1800	1
2	3	0	Above 5 ye	Compound shared wit	Rent	3000	2
12	8	0	Above 5 ye	House without compo	Own		7
2	0	0	Since birth	House with a private y	Rent	8000	7
3	3	0	Above 5 ye	House without compo	Own		4
4	3	2	Above 5 ye	Compound with family	Own		6
4	0	0	Less than :	Room in a multi-unit b	Rent	1200	1
1	1	0	3-5 years	Compound shared wit	Rent	1700	1
1	1	1	Since birth	Compound with family	Own		1
3	0	0	Above 5 ye	Compound with family	Own		4
3	0	1	Above 5 ye	Compound with family	Own		5
2	3	0	1-2 years	House with a private y	Own		3
1	0	0	Above 5 ye	House without compo	Own		2
2	1	1	Above 5 ye	A block with houses	Rent	4000	3
7	4	0	Above 5 ye	House without compo	Own		4
3	0	2	3-5 years	Compound shared wit	Rent	4000	2
1	2	0	Above 5 ye	Room in a multi-unit b	Own		2
6	6	1	Above 5 ye	Compound shared wit	Own		3
3	4	0	Above 5 ye	Compound shared wit	Own		1
1	0	0	1-2 years	Room in a multi-unit b	Rent	1500	1
3	0	0	Above 5 ye	House with a private y	Own		2
3	1	4	Above 5 ye	House without compo	Own		4
3	1	3	Above 5 ye	Compound shared wit	Own		1
3	1	2	Above 5 ye	Room in a multi-unit b	Rent	2000	1
4	0	1	Above 5 ye	House with a private y	Own		3
2	2	0	3-5 years	Compound with family	Own		4
2	3	1	Above 5 ye	Compound shared wit	Rent	6000	2
2	2	0	Above 5 ye	A block with houses	Rent	2500	1
2	1	1	Less than :	Compound shared wit	Rent	3500	2
2	2	0	Above 5 ye	Room in a multi-unit b	Own		2
2	2	1	1-2 years	Compound shared wit	Rent	2400	2
3	0	0	Above 5 ye	Room in a multi-unit b	Rent	2500	1
2	0	1	Above 5 ye	Compound shared wit	Rent	3500	2
2	2	1	3-5 years	A block with houses	Rent	2300	1
2	2	2	Above 5 ye	A block with houses	Rent	2000	2
4	5	2	Above 5 ye	House without compo	Own		3
2	0	2	1-2 years	Room in a multi-unit b	Rent	3000	1
2	2	1	Less than :	Room in a multi-unit b	Rent	1500	1
6	3	1	Above 5 ye	House with a private y	Own		6
2	0	1	3-5 years	Compound shared wit	Rent	5000	1
2	0	0	Above 5 ye	Compound shared wit	Rent	1500	1
2	3	1	Above 5 ye	A block with houses	Rent	2500	2
2	5	1	Above 5 ye	House without compo	Own		1
2	5	1	Above 5 ye	House without compo	Own		3
2	0	0	Less than :	Room in a multi-unit b	Rent	1500	1
3	0	1	Above 5 ye	Compound shared wit	Rent	5000	2

1	0	0	Above 5 ye	House without compo	Own		2
2	0	1	1-2 years	Room in a multi-unit b	Rent	1000	4
2	2	0	1-2 years	Compound shared wit	Rent	3000	1
2	0	0	1-2 years	Compound shared wit	Rent	2500	1
2	0	1	1-2 years	House without compo	Own		3
3	0	1	Above 5 ye	Room in a multi-unit b	Own		2
3	2	1	Above 5 ye	Compound shared wit	Rent	3000	1
4	2	1	Since birth	A block with houses	Rent	2500	2
2	0	1	Less than :	Compound shared wit	Rent	3000	1
2	3	1	Less than :	House without compo	Rent	2000	1
1	4	0	Above 5 ye	Compound shared wit	Rent	3500	1
2	2	2	Less than :	Compound shared wit	Rent	2500	1
2	1	1	Above 5 ye	House without compo	Rent	1200	1
2	1	1	Less than :	Compound shared wit	Rent	5000	2
2	0	0	Above 5 ye	House without compo	Own		3
2	1	2	3-5 years	Compound shared wit	Rent	5000	2
2	1	1	Less than :	Compound shared wit	Rent	4000	3
4	0	0	1-2 years	Room in a multi-unit b	Rent	4500	1
3	0	0	Less than :	Room in a multi-unit b	Rent	1500	1
3	0	0	Less than :	Room in a multi-unit b	Rent	2000	1
2	0	0	Above 5 ye	A block with houses	Rent	2000	1
5	0	0	Above 5 ye	House without compo	Own		5
3	3	0	Above 5 ye	Compound with family	Own		3
3	3	1	1-2 years	A block with houses	Rent	4500	2
4	1	2	Above 5 ye	Compound shared wit	Own		3
3	1	0	Above 5 ye	A block with houses	Rent	800	1
2	1	1	Less than :	Compound shared wit	Rent	2500	2
4	2	1	3-5 years	Compound shared wit	Rent	1300	1
1	0	0	Above 5 ye	Room in a multi-unit b	Rent	1500	1
2	5	0	Above 5 ye	House without compo	Rent	1000	1
4	2	0	Above 5 ye	A block with houses	Rent	4000	1
2	4	0	1-2 years	Compound shared wit	Rent	2000	1
1	4	0	Above 5 ye	House without compo	Own		2
2	2	1	Above 5 ye	House without compo	Own		3
3	1	0	Above 5 ye	House with a private y	Rent	5000	3
6	0	0	Above 5 ye	House without compo	Own		2
3	0	0	Above 5 ye	House without compo	Own		6
3	1	1	Above 5 ye	Compound shared wit	Rent	4000	2
2	0	1	Less than :	Compound shared wit	Rent	2700	1
3	1	0	Above 5 ye	Compound shared wit	Own		2
4	2	2	Above 5 ye	House without compo	Rent	3000	2
6	2	2	Since birth	A block with houses	Own		5
3	4	2	Above 5 ye	Compound with family	Rent	5000	2
2	2	0	Above 5 ye	Compound shared wit	Rent	3000	1
3	0	0	1-2 years	Room in a multi-unit b	Rent	2300	1
4	2	0	Above 5 ye	Room in a multi-unit b	Rent	4000	2
3	2	0	Above 5 ye	House without compo	Own		3
2	1	1	1-2 years	Compound shared wit	Rent	2000	1
2	0	1	Less than :	Room in a multi-unit b	Rent	2000	1

2	3	0	1-2 years	Compound shared wit	Rent	5000	2
1	2	0	3-5 years	House without compo	Own		5
3	4	1	Less than :	Compound shared wit	Rent	2500	2
4	1	0	Above 5 ye	House without compo	Own		4
1	0	0	Above 5 ye	Room in a multi-unit b	Rent	1000	1
2	0	0	1-2 years	Compound shared wit	Rent	4500	1
1	0	0	Less than :	Compound shared wit	Rent	1000	1
2	0	0	Above 5 ye	House without compo	Own		9
2	0	0	Above 5 ye	House without compo	Own		2
2	0	1	1-2 years	Compound shared wit	Rent	1500	1
3	0	0	Above 5 ye	Room in a multi-unit b	Rent	1000	1
3	4	0	3-5 years	Compound shared wit	Rent	5000	2
4	3	1	Since birth	Compound Compound	Own		4
3	2	2	Above 5 ye	Compound shared wit	Rent	2000	1
1	3	0	Above 5 ye	House without compo	Own		1
2	4	1	Above 5 ye	A block with houses	Rent	2500	1
7	6	0	Above 5 ye	House without compo	Own		2
1	0	0	Less than :	Compound shared wit	Rent	3300	1
2	1	1	Above 5 ye	Compound shared wit	Rent	2700	1
3	3	0	Above 5 ye	House without compo	Own		2
2	2	0	Above 5 ye	House with a private y	Own		3
2	1	1	Above 5 ye	A block with houses	Rent	4000	1
3	2	0	1-2 years	Compound shared wit	Rent	4000	4
1	0	0	1-2 years	Room in a multi-unit b	Live here for free		6
3	1	0	Above 5 ye	House with a private y	Own		3
2	2	0	Above 5 ye	Compound shared wit	Live here for free		1
5	0	0	Above 5 ye	Room in a multi-unit b	Rent	2500	1
1	0	0	Above 5 ye	Room in a multi-unit b	Live here for free		2
4	1	0	Above 5 ye	Compound shared wit	Rent	3500	3
3	1	0	Above 5 ye	Compound shared wit	Rent	1500	1
3	0	1	Less than :	Room in a multi-unit b	Rent	1800	1
3	6	1	Above 5 ye	House with a private y	Own		3
2	1	1	Less than :	Compound shared wit	Rent	2500	1
4	0	0	Above 5 ye	Room in a multi-unit b	Rent	1500	1
2	0	0	Less than :	Compound shared wit	Rent	4000	1
3	5	0	Above 5 ye	Room in a multi-unit b	Own		3
2	0	0	Above 5 ye	A block with houses	Rent	1000	1
2	0	0	Less than :	A block with houses	Rent	2500	3
2	2	1	Above 5 ye	House without compo	Own		3
4	0	0	Above 5 ye	House without compo	Rent	3000	2
5	0	3	Above 5 ye	Compound shared wit	Own		3
3	0	2	3-5 years	House without compo	Rent	3000	1
2	0	1	3-5 years	Room in a multi-unit b	Rent	6000	1
2	0	0	Above 5 ye	Compound shared wit	Own		6
2	0	4	Above 5 ye	A block with houses	Own		2
2	1	0	Above 5 ye	Compound shared wit	Rent	3500	1
2	0	0	Less than :	Compound shared wit	Rent	5000	1
1	1	0	Above 5 ye	Compound shared wit	Own		2
3	0	1	1-2 years	Compound shared wit	Rent	4500	2

1	0	0	Above 5 ye	House without compoı	Own		2
3	1	1	1-2 years	Compound shared wit	Rent	10000	4
1	0	0	3-5 years	Compound shared wit	Rent	1500	1
1	0	0	Above 5 ye	Compound shared wit	Own		1
8	0	3	Above 5 ye	Compound shared wit	Rent	600	3
2	0	1	Less than :	A block with houses	Rent	3000	1
2	0	0	Above 5 ye	House without compoı	Own		2
2	1	1	Above 5 ye	A block with houses	Rent	2700	1
4	2	0	Above 5 ye	House with a private y	Own		2
2	0	0	Above 5 ye	House without compoı	Own		2
2	1	2	3-5 years	A block with houses	Rent	3000	1
3	1	0	Above 5 ye	House without compoı	Own		1
3	0	1	Above 5 ye	A block with houses	Rent	3000	3
2	1	1	Above 5 ye	House without compoı	Own		3
1	0	0	Less than :	A block with houses	Rent	5000	1
3	0	1	Above 5 ye	Compound shared wit	Rent	5000	1
3	0	0	Above 5 ye	House without compoı	Own		3
3	4	1	1-2 years	Compound shared wit	Rent	2000	1
6	0	0	Above 5 ye	House without compoı	Own		5
1	0	0	Above 5 ye	Compound shared wit	Rent	2500	1
4	2	0	Above 5 ye	A block with houses	Live here for free		1
3	2	0	3-5 years	A block with houses	Rent	3500	2
2	4	0	Above 5 ye	Room in a multi-unit b	Rent	5000	2
2	0	1	Less than :	Compound shared wit	Rent	6500	3
3	6	3	Above 5 ye	Compound shared wit	Rent	3000	2
2	0	0	3-5 years	Room in a multi-unit b	Rent	1200	2
2	3	1	Above 5 ye	Compound shared wit	Own		2
6	5	2	Since birth	House without compoı	Own		7
3	1	0	Above 5 ye	House with a private y	Rent	5000	4
2	1	1	Less than :	Compound shared wit	Rent	4500	1
3	2	0	Above 5 ye	Compound shared wit	Rent	4500	2
4	2	0	Above 5 ye	House with a private y	Own		3
2	5	0	1-2 years	A block with houses	Rent	3000	2
2	4	0	Since birth	Room in a multi-unit b	Rent	3000	1
2	1	1	Above 5 ye	Compound shared wit	Rent	2000	2
4	1	0	Less than :	Room in a multi-unit b	Rent	1400	1
2	3	0	3-5 years	A block with houses	Rent	3500	1
2	0	2	Above 5 ye	Compound shared wit	Rent	3000	1
4	2	1	1-2 years	Compound shared wit	Rent	4500	2
2	0	0	Less than :	Room in a multi-unit b	Rent	7000	4
2	0	1	3-5 years	Compound shared wit	Rent	3000	1
4	2	1	Since birth	House without compoı	Own		2
1	2	0	1-2 years	Compound shared wit	Rent	5000	3
4	4	0	3-5 years	House with a private y	Own		2
3	3	0	Above 5 ye	House with a private y	Own		2
2	1	1	1-2 years	A block with houses	Rent	2500	1
2	0	0	1-2 years	Compound shared wit	Rent	2000	1
2	3	0	Above 5 ye	Compound shared wit	Rent	3500	2
1	4	0	1-2 years	Compound shared wit	Rent	2300	2

2	0	0	Above 5 ye	Compound shared wit	Live here for free		1
2	1	1	Above 5 ye	Compound with family	Own		3
2	0	1	1-2 years	Compound shared wit	Rent	3000	1
3	1	0	3-5 years	Compound shared wit	Own		1
2	2	0	3-5 years	Compound shared wit	Rent	1500	2
1	2	2	Less than :	A block with houses	Rent	1800	1
2	0	0	Above 5 ye	House with a private y	Own		2
4	2	1	Above 5 ye	Compound shared wit	Own		3
6	0	0	Above 5 ye	House without compoı	Own		2
2	0	2	3-5 years	A block with houses	Rent	3000	1
1	0	0	Less than :	Compound shared wit	Rent	2200	1
6	1	0	Above 5 ye	Room in a multi-unit b	Rent	1000	3
2	2	2	Above 5 ye	Compound shared wit	Rent	2000	1
4	0	0	Above 5 ye	House without compoı	Own		3
3	2	4	1-2 years	Compound shared wit	Rent	2500	1
2	0	0	Less than :	Compound shared wit	Rent	5000	1
3	3	0	Above 5 ye	Compound shared wit	Own		4
2	1	0	Above 5 ye	House without compoı	Rent	1800	1
2	3	0	3-5 years	Compound shared wit	Rent	2500	1
3	2	1	1-2 years	Compound shared wit	Rent	5500	2
2	2	0	Above 5 ye	House without compoı	Own		2
2	0	1	Less than :	A block with houses	Rent	1000	1
2	0	2	1-2 years	Compound shared wit	Rent	2500	1
2	3	1	Above 5 ye	Room in a multi-unit b	Own		4
2	2	1	Above 5 ye	Compound shared wit	Rent	2000	2
2	0	1	Less than :	Compound shared wit	Rent	2500	1
6	2	0	Above 5 ye	Compound shared wit	Rent	4000	2
2	2	2	Above 5 ye	Room in a multi-unit b	Rent	1000	1
4	3	0	Above 5 ye	Compound shared wit	Own		4
2	1	1	1-2 years	Compound shared wit	Rent	3500	2
6	0	0	Above 5 ye	House without compoı	Live here for free		3
3	0	1	1-2 years	Compound shared wit	Rent	4000	2
2	0	0	1-2 years	A block with houses	Rent	2800	1
2	4	1	3-5 years	Compound shared wit	Rent	1300	1
2	0	1	1-2 years	Room in a multi-unit b	Live here for free		4
4	1	0	Above 5 ye	House without compoı	Own		2
2	1	1	Since birth	Compound with family	Own		2
1	3	0	1-2 years	Compound shared wit	Rent	2500	1
2	0	1	Less than :	A block with houses	Rent	1600	1
1	1	1	Less than :	Compound shared wit	Rent	1000	1
3	1	0	Above 5 ye	Compound shared wit	Own		5
2	1	1	1-2 years	Compound shared wit	Rent	3200	1
2	0	0	1-2 years	Compound shared wit	Rent	6000	2
1	0	0	1-2 years	A block with houses	Rent	1800	1
1	3	3	Less than :	Compound shared wit	Rent	2000	1
2	0	0	Less than :	Compound shared wit	Rent	2200	1
4	3	0	Above 5 ye	Room in a multi-unit b	Own		3
2	5	3	Above 5 ye	Compound with family	Own		3
2	1	0	Above 5 ye	Compound shared wit	Rent	2000	2

1	0	0	1-2 years	Compound shared wit	Rent	2900	1
1	0	0	Less than :	Compound shared wit	Rent	3500	1
1	1	1	3-5 years	House without compo	Own		1
2	2	0	Above 5 ye	Compound shared wit	Rent	4000	2
4	1	1	Above 5 ye	Compound shared wit	Rent	10000	4
3	5	2	Above 5 ye	Compound shared wit	Rent	4500	3
2	2	1	Above 5 ye	Compound shared wit	Rent	2500	1
1	0	0	1-2 years	Compound shared wit	Rent	4000	2
2	2	0	Above 5 ye	Compound shared wit	Rent	8000	4
2	0	0	Less than :	Compound shared wit	Own		1
4	3	0	Above 5 ye	Compound shared wit	Rent	2000	1
2	0	0	Above 5 ye	House with a private y	Own		2
2	0	1	Less than :	Compound with family	Rent	1500	1
1	0	0	3-5 years	A block with houses	Rent	1500	1
3	0	2	Above 5 ye	Compound shared wit	Rent	2500	2
2	1	0	1-2 years	Room in a multi-unit b	Rent	3500	1
1	1	0	Since birth	Compound shared wit	Live here for free		2
2	1	0	1-2 years	A block with houses	Rent	3500	2
4	2	2	Above 5 ye	A block with houses	Rent	5000	3
1	2	0	Above 5 ye	A block with houses	Rent	1500	1
3	0	0	Since birth	Compound shared wit	Own		1
2	0	2	1-2 years	A block with houses	Rent	2600	1
4	3	0	Above 5 ye	Compound shared wit	Rent	3300	1
3	0	0	1-2 years	Room in a multi-unit b	Rent	3000	1
4	3	0	3-5 years	Compound shared wit	Rent	6600	2
2	1	2	3-5 years	A block with houses	Rent	1500	1
4	1	0	Above 5 ye	Room in a multi-unit b	Own		6
7	3	0	Above 5 ye	A block with houses	Own		1
2	0	0	3-5 years	Room in a multi-unit b	Live here for free		1
2	3	1	Above 5 ye	Compound shared wit	Rent	5000	2
2	1	1	1-2 years	Compound shared wit	Rent	6000	1
2	1	0	Above 5 ye	Compound shared wit	Rent	3000	1
1	2	0	Less than :	Compound shared wit	Rent	2200	1
7	5	0	3-5 years	House without compo	Own		7
2	1	2	Less than :	Compound shared wit	Rent	2000	1
2	3	0	Less than :	Compound shared wit	Rent	3000	1
2	0	1	1-2 years	Compound shared wit	Rent	3000	1
2	1	1	1-2 years	Compound shared wit	Rent	2500	1
2	1	1	1-2 years	A block with houses	Rent	2500	1
3	0	0	Above 5 ye	House with a private y	Own		4
1	3	1	3-5 years	Compound shared wit	Rent	700	1
2	1	1	Above 5 ye	Compound shared wit	Rent	4500	3
3	2	1	3-5 years	Compound shared wit	Rent	3500	2
2	2	0	Above 5 ye	Compound shared wit	Rent	3500	1
2	0	1	3-5 years	Room in a multi-unit b	Rent	2250	1
1	0	0	Above 5 ye	Compound shared wit	Rent	3000	1
2	0	2	Less than :	Compound shared wit	Rent	4000	1
2	1	0	1-2 years	Room in a multi-unit b	Rent	2500	1
1	2	2	3-5 years	A block with houses	Rent	2000	1

2	3	0	Above 5 ye	House without compo	Own		6
1	3	1	3-5 years	Compound shared wit	Rent	3500	1
2	0	0	1-2 years	Compound shared wit	Rent	3500	1
1	3	1	3-5 years	A block with houses	Rent	3500	2
3	4	1	Less than :	Compound shared wit	Rent	8000	4
1	1	0	3-5 years	A block with houses	Rent	1500	1
3	0	1	Less than :	A block with houses	Rent	7000	3
3	2	2	1-2 years	Compound shared wit	Rent	14000	2
2	1	1	3-5 years	Compound shared wit	Rent	5500	1
2	0	1	1-2 years	Compound shared wit	Rent	2100	1
2	1	0	1-2 years	Compound shared wit	Rent	5000	1
2	0	0	Above 5 ye	Compound shared wit	Rent	2000	1
3	0	1	Above 5 ye	Room in a multi-unit b	Rent	7000	2
1	2	1	Less than :	A block with houses	Rent	1000	1
2	1	1	1-2 years	Compound shared wit	Rent	4100	1
3	2	0	Above 5 ye	Compound shared wit	Rent	5500	1
1	2	1	Less than :	Compound shared wit	Rent	3500	2
2	3	2	Above 5 ye	A block with houses	Rent	4000	2
1	0	0	Above 5 ye	Room in a multi-unit b	Rent	2500	1
3	1	1	1-2 years	Compound shared wit	Rent	4000	2
2	0	0	Less than :	Compound shared wit	Rent	3000	1
2	2	1	Above 5 ye	Compound shared wit	Rent	3000	2
3	3	1	3-5 years	A block with houses	Rent	3500	1
2	1	0	Less than :	A block with houses	Rent	2500	1
2	0	0	1-2 years	A block with houses	Rent	3300	2
1	0	4	Above 5 ye	A block with houses	Live here for free		1
2	2	1	Above 5 ye	Compound shared wit	Rent	1500	1
2	2	1	3-5 years	House with a private y	Own		2
2	0	0	Less than :	A block with houses	Rent	2000	1
2	2	1	3-5 years	A block with houses	Own		2
4	2	1	Since birth	House with a private y	Own		3
3	2	0	Above 5 ye	A block with houses	Rent	3000	1
5	2	1	Above 5 ye	Compound shared wit	Rent	3000	1
1	2	0	1-2 years	Room in a multi-unit b	Rent	4000	1
2	0	0	1-2 years	Room in a multi-unit b	Rent	2000	1
2	1	0	1-2 years	House with a private y	Own		3
1	1	1	1-2 years	Compound shared wit	Rent	1300	1
2	0	0	Less than :	A block with houses	Rent	3000	1
2	1	1	3-5 years	Compound shared wit	Rent	3200	1
2	0	1	Less than :	House with a private y	Own		1
2	0	0	Less than :	Room in a multi-unit b	Rent	3000	1
3	2	0	3-5 years	Compound shared wit	Rent	5000	2
2	0	0	Above 5 ye	Compound shared wit	Rent	4000	1
2	0	3	Less than :	Compound shared wit	Rent	3100	1
1	0	0	Above 5 ye	Compound shared wit	Rent	3000	1
3	4	2	Above 5 ye	A block with houses	Rent	2000	1
2	7	0	Less than :	Compound shared wit	Rent	1800	1
3	2	0	3-5 years	Compound Compound	Rent	2500	1
3	2	2	1-2 years	Compound shared wit	Rent	3500	1

3	0	0	1-2 years	Compound shared wit	Rent	3500	1
1	1	0	1-2 years	A block with houses	Rent	1800	1
2	1	0	3-5 years	A block with houses	Rent	3000	1
4	3	0	Above 5 ye	A block with houses	Rent	3000	1
4	2	0	Above 5 ye	A block with houses	Rent	2000	1
2	0	0	1-2 years	Room in a multi-unit b	Rent	4500	1
2	0	1	Above 5 ye	Compound shared wit	Rent	2500	1
2	0	2	1-2 years	Compound shared wit	Rent	3000	1
2	0	1	1-2 years	Compound shared wit	Rent	2600	1
4	1	0	Above 5 ye	House without compo	Own		3
2	0	0	1-2 years	A block with houses	Rent	3500	1
2	3	0	Above 5 ye	Compound shared wit	Rent	1500	1
2	0	0	3-5 years	House without compo	Own		2
2	1	2	Above 5 ye	Compound with family	Rent	2200	1
2	1	1	3-5 years	Compound shared wit	Rent	2500	1
2	3	2	3-5 years	House with a private y	Rent	4500	4
2	0	1	1-2 years	House with a private y	Own		2
1	0	0	3-5 years	Room in a multi-unit b	Rent	3500	1
2	4	1	Above 5 ye	House without compo	Own		3
2	2	0	3-5 years	Room in a multi-unit b	Rent	2000	1
2	0	1	3-5 years	Room in a multi-unit b	Rent	3500	1
2	3	0	Above 5 ye	Compound shared wit	Rent	1000	2
6	0	0	Above 5 ye	House without compo	Own		8
5	1	0	Above 5 ye	Compound shared wit	Live here for free		2
5	2	0	Above 5 ye	Compound shared wit	Own		4
3	0	0	Above 5 ye	House with a private y	Own		3
2	3	0	Above 5 ye	House with a private y	Own		3
2	1	1	Above 5 ye	Compound shared wit	Rent	2000	1
3	1	0	Above 5 ye	A block with houses	Rent	3000	2
2	0	1	Above 5 ye	Compound shared wit	Rent	3500	2
2	1	1	Since birth	Compound shared wit	Rent	2500	1
2	0	1	1-2 years	Compound shared wit	Rent	2500	1
2	0	1	1-2 years	A block with houses	Rent	2100	1
8	3	0	3-5 years	Compound shared wit	Rent	7000	1
2	2	1	3-5 years	Compound shared wit	Rent	2500	1
2	0	1	Above 5 ye	Room in a multi-unit b	Rent	3500	1
2	4	2	Above 5 ye	House without compo	Own		2
5	1	0	Above 5 ye	Compound with family	Own		5
4	2	1	3-5 years	House with a private y	Own		1
2	0	2	Above 5 ye	A block with houses	Rent	3300	1
2	0	1	Less than :	Compound shared wit	Rent	4000	4
2	3	1	Above 5 ye	Compound shared wit	Rent	3000	1
1	0	0	1-2 years	Room in a multi-unit b	Rent	2700	1
6	4	4	Above 5 ye	Compound with family	Rent	7000	4
2	1	0	Less than :	A block with houses	Rent	2500	1
3	2	1	3-5 years	Compound shared wit	Rent	3500	1
2	1	0	1-2 years	Room in a multi-unit b	Rent	5000	1
2	1	1	1-2 years	A block with houses	Rent	7000	3
2	2	0	Above 5 ye	Compound shared wit	Rent	4000	1

2	1	1	Above 5 ye	Compound shared wit	Rent	4000	1
2	2	2	3-5 years	A block with houses	Rent	1000	1
2	1	1	Above 5 ye	Compound shared wit	Rent	3500	1
2	4	0	Above 5 ye	A block with houses	Rent	4500	2
2	1	1	Above 5 ye	Compound shared wit	Own		2
2	0	0	Less than :	House without compo	Own		5
2	1	0	Above 5 ye	House without compo	Own		2
2	2	1	Above 5 ye	A block with houses	Rent	4500	1
3	3	1	Less than :	Compound shared wit	Rent	4700	1
1	0	0	Since birth	Room in a multi-unit b	Live here for free		1
2	3	0	Above 5 ye	Compound shared wit	Rent	3000	2
2	3	2	1-2 years	A block with houses	Rent	2500	1
5	1	1	Above 5 ye	A block with houses	Rent	5000	2
5	0	0	Less than :	House without compo	Own		2
2	5	0	Above 5 ye	Compound shared wit	Rent	2000	1
2	1	2	Above 5 ye	A block with houses	Own		2
2	2	0	Above 5 ye	Compound shared wit	Own		6
2	2	1	1-2 years	Compound shared wit	Rent	8500	1
1	2	0	Above 5 ye	Compound shared wit	Rent	3000	1
2	4	0	Above 5 ye	A block with houses	Rent	4000	2
2	4	1	Above 5 ye	Compound shared wit	Rent	3000	1
2	2	0	Above 5 ye	Compound shared wit	Rent	3000	2
2	0	1	3-5 years	Compound shared wit	Rent	2300	1
4	1	1	3-5 years	A block with houses	Rent	7000	3
3	0	0	Above 5 ye	Compound with family	Rent	4000	2
2	3	0	Less than :	A block with houses	Rent	2500	1
3	1	1	Above 5 ye	Compound with family	Own		5
1	0	0	Less than :	Room in a multi-unit b	Rent	4500	1
5	2	0	Above 5 ye	Compound shared wit	Rent	13000	3
2	2	0	Above 5 ye	Compound shared wit	Rent	2000	2
10	0	0	Above 5 ye	A block with houses	Own		3
2	0	2	Less than :	Compound shared wit	Rent	3000	1
2	0	0	3-5 years	Compound shared wit	Rent	3000	1
1	0	0	Less than :	Compound shared wit	Rent	4600	1
2	3	0	3-5 years	Room in a multi-unit b	Rent	3000	1
2	0	0	Less than :	Compound shared wit	Rent	3500	1
4	2	0	Above 5 ye	A block with houses	Rent	3500	2
3	1	0	Above 5 ye	A block with houses	Rent	2000	1
2	1	2	Above 5 ye	Compound shared wit	Rent	3000	1
2	2	1	Above 5 ye	Room in a multi-unit b	Rent	3000	1
2	1	1	Less than :	Room in a multi-unit b	Rent	3500	1
2	0	1	3-5 years	Room in a multi-unit b	Rent	3500	1
1	0	0	3-5 years	Compound shared wit	Rent	5000	1
2	2	1	Above 5 ye	Compound shared wit	Live here for free		4
6	1	1	Above 5 ye	Compound shared wit	Rent	2000	1
4	3	1	1-2 years	House without compo	Own		4
1	0	0	Less than :	Compound shared wit	Rent	3000	1
4	1	0	Above 5 ye	Compound with family	Own		6
2	0	0	Less than :	Compound shared wit	Rent	2600	1

1	0	0	Above 5 ye	Room in a multi-unit b	Rent	3500	1
4	3	0	3-5 years	A block with houses	Rent	5000	3
1	0	0	Above 5 ye	Compound shared wit	Rent	4000	1
2	2	0	3-5 years	Compound shared wit	Rent	2500	1
2	1	0	Above 5 ye	Compound shared wit	Rent	4000	2
3	3	1	Above 5 ye	Compound shared wit	Own		2
2	0	3	Above 5 ye	Compound shared wit	Rent	3000	1
2	2	1	Above 5 ye	A block with houses	Rent	1000	1
1	4	0	Above 5 ye	House with a private y	Own		1
2	2	1	Above 5 ye	A block with houses	Rent	2000	2
5	6	2	Above 5 ye	House without compou	Own		6
3	0	0	Less than :	Room in a multi-unit b	Rent	4000	1
1	0	0	1-2 years	Compound shared wit	Rent	3700	1
2	2	0	Above 5 ye	A block with houses	Rent	2500	1
2	1	1	3-5 years	House with a private y	Own		3
2	2	0	Above 5 ye	Compound shared wit	Rent	4000	1
4	1	1	3-5 years	Compound shared wit	Rent	2000	1
3	0	0	1-2 years	Compound shared wit	Rent	2500	2
4	3	0	Above 5 ye	Room in a multi-unit b	Rent	6000	1
2	1	2	3-5 years	A block with houses	Rent	3000	1
3	0	2	3-5 years	A block with houses	Rent	3150	1
2	3	0	Above 5 ye	House without compou	Own		2
6	2	1	Less than :	Compound shared wit	Rent	4800	1
2	2	0	1-2 years	Compound shared wit	Rent	7000	3
1	0	1	1-2 years	Room in a multi-unit b	Rent	3500	1
2	0	1	3-5 years	A block with houses	Rent	1500	1
2	2	2	Above 5 ye	House without compou	Own		4
2	3	0	1-2 years	A block with houses	Rent	3000	3
2	6	0	Above 5 ye	House with a private y	Own		3
3	0	1	Above 5 ye	Compound shared wit	Own		5
1	2	0	Above 5 ye	A block with houses	Rent	3000	1
2	0	1	Less than :	A block with houses	Rent	5500	2
1	1	0	1-2 years	Compound shared wit	Rent	3400	1
3	3	1	Above 5 ye	Compound shared wit	Rent	3500	3
2	0	0	Above 5 ye	A block with houses	Rent	7000	4
1	1	1	1-2 years	A block with houses	Rent	2500	1
2	1	0	Above 5 ye	Compound shared wit	Rent	7000	4
2	1	2	3-5 years	Compound shared wit	Rent	5000	2
3	1	0	Above 5 ye	House without compou	Own		3
2	2	0	1-2 years	House without compou	Rent	3000	2
3	2	2	Above 5 ye	Compound shared wit	Rent	2000	1
4	3	2	Above 5 ye	House with a private y	Own		3
2	0	1	1-2 years	A block with houses	Rent	2000	1
2	1	1	1-2 years	A block with houses	Rent	3000	1
6	0	0	Less than :	A block with houses	Own		7
1	0	0	Less than :	Room in a multi-unit b	Rent	2600	1
4	1	0	Above 5 ye	Compound shared wit	Rent	5000	4
2	3	0	Above 5 ye	A block with houses	Live here for free		2
2	2	3	3-5 years	A block with houses	Rent	2000	1

4	4	0	3-5 years	House with a private y	Own		6
2	2	1	3-5 years	A block with houses	Rent	4000	1
2	0	1	Above 5 ye	House with a private y	Own		4
4	1	3	Above 5 ye	Compound with family	Own		2
2	0	1	Above 5 ye	Compound shared wit	Rent	3500	1
3	1	0	1-2 years	House with a private y	Rent	3000	1
2	0	0	Less than :	A block with houses	Rent	2000	1
2	0	0	Above 5 ye	Compound shared wit	Rent	2500	1
3	1	0	Above 5 ye	Room in a multi-unit b	Rent	5000	1
2	0	0	1-2 years	Room in a multi-unit b	Rent	6000	1
2	3	1	Above 5 ye	Compound shared wit	Rent	8500	4
2	0	0	1-2 years	Room in a multi-unit b	Rent	3000	1
2	1	0	Above 5 ye	House without compo	Own		5
2	3	0	Above 5 ye	Compound shared wit	Own		3
2	0	1	Above 5 ye	Compound shared wit	Rent	2500	1
2	2	1	3-5 years	A block with houses	Rent	6500	1
3	2	1	1-2 years	House without compo	Own		3
2	1	0	Above 5 ye	Compound shared wit	Rent	3500	3
2	0	2	1-2 years	Compound shared wit	Rent	2600	1
2	1	2	3-5 years	A block with houses	Rent	4500	2
2	2	0	3-5 years	Compound with family	Rent	5000	2
4	3	0	Above 5 ye	House without compo	Own		5
1	0	0	Above 5 ye	A block with houses	Rent	1200	1
2	1	1	Less than :	A block with houses	Rent	4500	1
2	9	0	1-2 years	House without compo	Rent	1500	2
4	4	1	Above 5 ye	Compound with family	Own		3
2	2	1	Above 5 ye	House without compo	Own		3
2	1	1	Above 5 ye	Compound with family	Rent	2500	1
2	2	2	Above 5 ye	Room in a multi-unit b	Rent	3000	1
2	2	0	Above 5 ye	Room in a multi-unit b	Own		2
2	0	1	3-5 years	A block with houses	Rent	1000	1
7	0	1	Above 5 ye	House with a private y	Own		7
3	1	0	Above 5 ye	Compound shared wit	Rent	3000	1
3	3	1	3-5 years	House with a private y	Rent	9000	4
1	3	1	3-5 years	A block with houses	Rent	2000	1
3	2	0	1-2 years	A block with houses	Rent	6500	1
2	0	0	1-2 years	Compound shared wit	Rent	3600	1
2	0	0	Above 5 ye	House with a private y	Own		3
2	3	0	Above 5 ye	Compound shared wit	Rent	3000	1
2	0	1	Above 5 ye	Compound shared wit	Rent	3000	2
2	3	0	Above 5 ye	Compound shared wit	Rent	5500	4
2	1	0	1-2 years	A block with houses	Rent	5000	2
2	2	2	Above 5 ye	Compound with family	Own		2
1	0	0	1-2 years	Compound shared wit	Rent	2500	1
4	1	0	Since birth	House without compo	Own		5
2	0	1	1-2 years	A block with houses	Rent	6000	2
2	2	1	1-2 years	Compound shared wit	Rent	3500	1
2	0	1	1-2 years	Compound shared wit	Rent	5000	1
2	0	1	3-5 years	A block with houses	Rent	4500	2

2	1	2	Above 5 ye	A block with houses	Rent	2500	2
2	0	1	1-2 years	Compound shared wit	Rent	14000	2
1	0	0	3-5 years	Room in a multi-unit b	Rent	3500	1
4	3	1	Above 5 ye	Compound shared wit	Own		1
8	0	0	Above 5 ye	House without compo	Own		2
3	0	2	Less than :	Compound shared wit	Rent	7000	3
4	1	0	Above 5 ye	A block with houses	Own		4
1	2	1	Less than :	A block with houses	Rent	4500	1
3	0	3	Above 5 ye	Compound shared wit	Own		3
3	4	1	1-2 years	Compound shared wit	Rent	4000	2
2	0	2	Less than :	Compound shared wit	Rent	2500	1
6	1	0	Above 5 ye	A block with houses	Rent	8000	3
2	1	1	Above 5 ye	Compound shared wit	Rent	3500	1
3	0	0	1-2 years	Compound shared wit	Rent	3000	1
2	0	1	Less than :	Compound shared wit	Rent	1850	1
3	4	0	Above 5 ye	Compound shared wit	Rent	4000	2
3	0	0	Above 5 ye	A block with houses	Own		2
3	3	3	1-2 years	Compound shared wit	Rent	3000	2
2	0	1	Less than :	A block with houses	Rent	1500	1
2	2	0	Above 5 ye	Compound with family	Own		4
8	2	6	Above 5 ye	House without compo	Own		4
2	0	1	1-2 years	Compound shared wit	Rent	4500	1
2	1	0	Above 5 ye	Compound shared wit	Rent	2500	1
3	0	0	1-2 years	A block with houses	Rent	5000	2
3	2	2	1-2 years	Compound shared wit	Rent	4500	2
2	1	1	1-2 years	Compound shared wit	Rent	8000	4
1	0	0	Less than :	A block with houses	Rent	2500	1
2	2	1	3-5 years	A block with houses	Rent	2500	1
2	1	1	Above 5 ye	House with a private y	Rent	3000	1
2	3	1	3-5 years	A block with houses	Rent	3000	1
3	5	0	Above 5 ye	Compound shared wit	Rent	2000	2
2	0	1	1-2 years	Compound shared wit	Rent	1500	1
3	0	0	Above 5 ye	House without compo	Own		2
2	0	1	1-2 years	Compound shared wit	Rent	2000	1
2	2	0	Above 5 ye	Compound shared wit	Rent	4500	2
2	2	0	Less than :	Compound shared wit	Rent	2500	1
2	2	1	Above 5 ye	Compound with family	Rent	5000	2
2	0	0	Less than :	Compound shared wit	Rent	2500	1
2	0	1	Less than :	Compound shared wit	Rent	6000	1
3	3	0	Above 5 ye	Compound shared wit	Rent	2000	1
2	1	2	1-2 years	Compound shared wit	Rent	6500	3
3	1	0	Above 5 ye	House with a private y	Own		3
1	4	1	1-2 years	Compound shared wit	Rent	2500	1
5	3	1	Since birth	A block with houses	Rent	8000	4
1	0	0	1-2 years	Compound with family	Rent	1000	1
2	3	1	1-2 years	Compound shared wit	Rent	3000	2
2	0	1	3-5 years	Compound shared wit	Rent	3500	1
2	2	0	Above 5 ye	Room in a multi-unit b	Rent	5000	2
2	2	1	1-2 years	A block with houses	Rent	6500	1

2	2	1	Above 5 ye	Compound shared wit	Rent	3000	1
2	0	1	3-5 years	Compound shared wit	Rent	3500	1
2	2	0	Above 5 ye	House with a private y	Own		6
2	0	3	1-2 years	Compound shared wit	Rent	2000	1
3	3	2	3-5 years	Compound shared wit	Rent	6500	5
2	3	1	1-2 years	A block with houses	Rent	3500	1
2	3	1	Less than :	Compound shared wit	Rent	3000	1
2	1	0	3-5 years	Compound shared wit	Rent	5800	3
1	11	0	3-5 years	Compound shared wit	Rent	2500	2
2	1	1	3-5 years	A block with houses	Rent	5500	3
1	0	0	1-2 years	A block with houses	Rent	2000	1
2	1	1	3-5 years	A block with houses	Rent	3000	1
2	1	1	1-2 years	Compound shared wit	Rent	4500	3
2	1	2	1-2 years	Compound shared wit	Rent	7000	3
4	0	3	1-2 years	Compound shared wit	Rent	2000	1
2	4	0	Less than :	A block with houses	Rent	1500	1
2	0	1	Less than :	Compound shared wit	Rent	3000	1
4	5	1	1-2 years	Compound shared wit	Rent	5500	3
1	0	0	Less than :	Compound a compour	Own		1
2	5	1	Above 5 ye	A block with houses	Own		5
2	1	0	Above 5 ye	Compound shared wit	Rent	3000	2
3	3	1	Above 5 ye	Compound shared wit	Rent	4000	2
2	2	2	Above 5 ye	House with a private y	Own		3
2	1	1	Less than :	Compound shared wit	Rent	800	1
2	1	1	Above 5 ye	A block with houses	Rent	5500	1
2	0	1	3-5 years	Compound shared wit	Rent	4000	1
2	0	0	Less than :	Room in a multi-unit b	Rent	3500	1
2	0	0	1-2 years	Compound shared wit	Rent	3500	1
1	2	0	1-2 years	A block with houses	Rent	3500	1
4	3	1	Above 5 ye	A block with houses	Rent	4000	2
2	0	1	Above 5 ye	Compound shared wit	Rent	2600	1
2	3	1	1-2 years	Compound shared wit	Rent	2500	1
1	1	0	Above 5 ye	House with a private y	Live here for free		7
2	0	0	Less than :	Compound shared wit	Rent	3500	1
1	0	0	Above 5 ye	House with a private y	Live here for free		3
2	0	0	Above 5 ye	Compound shared wit	Rent	2500	1
2	2	1	3-5 years	Compound shared wit	Rent	4500	2
2	0	1	Less than :	Room in a multi-unit b	Rent	2500	1
7	0	0	Above 5 ye	Room in a multi-unit b	Own		10
2	1	1	Less than :	Room in a multi-unit b	Rent	3500	1
2	3	0	1-2 years	Compound shared wit	Rent	4000	3
2	1	1	3-5 years	Compound shared wit	Rent	3000	2
3	4	0	Above 5 ye	Compound shared wit	Rent	3500	2
4	0	0	Since birth	Compound with family	Own		5
4	1	0	Above 5 ye	Compound shared wit	Rent	3000	1
2	2	1	3-5 years	Room in a multi-unit b	Rent	2500	1
3	1	0	Above 5 ye	Compound with family	Own		4
1	0	1	3-5 years	A block with houses	Rent	1500	1
2	2	0	3-5 years	Compound shared wit	Rent	4100	2

3	0	1	Less than :	Compound shared wit	Rent	3500	1
2	1	0	Less than :	A block with houses	Rent	2000	1
4	1	1	Above 5 ye	Room in a multi-unit b	Rent	6000	2
3	1	0	Above 5 ye	Room in a multi-unit b	Rent	4000	2
5	0	0	Above 5 ye	House without compo	Own		3
4	2	0	Above 5 ye	House without compo	Own		4
2	1	1	Above 5 ye	A block with houses	Rent	3000	2
1	0	0	Above 5 ye	Compound shared wit	Rent	1500	1
2	2	2	Above 5 ye	Compound shared wit	Rent	4100	2
2	0	1	Less than :	Room in a multi-unit b	Rent	4000	1
2	3	0	Above 5 ye	Compound shared wit	Rent	4500	4
2	1	0	Above 5 ye	Compound shared wit	Rent	1000	1
2	2	1	Since birth	Compound with family	Rent	8000	1
2	0	1	1-2 years	Room in a multi-unit b	Rent	5500	1
2	0	2	Less than :	Compound shared wit	Rent	2500	1
2	1	0	Above 5 ye	Compound with family	Rent	9000	5
2	1	1	Above 5 ye	Room in a multi-unit b	Rent	2000	1
3	0	0	Above 5 ye	A block with houses	Rent	5000	3
4	2	1	Less than :	Compound shared wit	Rent	3000	1
2	0	0	Less than :	A block with houses	Rent	4000	1
1	1	0	3-5 years	A block with houses	Rent	1600	1
1	0	1	Less than :	Compound shared wit	Rent	2500	1
2	2	0	1-2 years	A block with houses	Rent	4000	2
2	0	0	3-5 years	Room in a multi-unit b	Rent	3000	1
3	2	1	3-5 years	Compound shared wit	Rent	4100	2
2	2	1	1-2 years	Compound shared wit	Rent	4000	2
2	2	1	1-2 years	Compound shared wit	Rent	5000	2
2	4	1	Above 5 ye	House with a private y	Live here for free		3
2	0	0	Less than :	Room in a multi-unit b	Rent	2000	1
1	0	0	Above 5 ye	A block with houses	Rent	2000	1
6	1	0	1-2 years	Room in a multi-unit b	Own		2
2	3	2	Less than :	Compound shared wit	Rent	2000	2
2	1	0	1-2 years	Compound shared wit	Rent	3500	1
3	3	0	3-5 years	Compound One bedro	Rent	5000	2
1	2	2	Above 5 ye	House without compo	Own		2
2	0	1	Above 5 ye	House with a private y	Own		7
2	0	0	Less than :	Room in a multi-unit b	Rent	3000	1
2	1	1	3-5 years	Compound shared wit	Rent	4000	2
2	0	0	Above 5 ye	Compound shared wit	Rent	3000	1
2	0	1	Less than :	Room in a multi-unit b	Rent	5600	1
2	0	1	Less than :	Room in a multi-unit b	Rent	1000	1
2	1	2	1-2 years	Room in a multi-unit b	Rent	1500	1
2	3	1	Less than :	A block with houses	Rent	2500	2
2	0	0	Above 5 ye	A block with houses	Rent	4000	2
2	1	1	Above 5 ye	Compound shared wit	Rent	4500	2
4	3	0	Above 5 ye	Room in a multi-unit b	Own		4
2	2	1	Above 5 ye	Room in a multi-unit b	Rent	6000	2
2	0	2	1-2 years	A block with houses	Rent	4000	1
2	0	1	Less than :	A block with houses	Live here for free		1

1	0	0	Above 5 ye	House without compou	Own		2
3	2	0	3-5 years	Room in a multi-unit b	Own		2
2	0	0	3-5 years	Compound shared wit	Rent	3000	1
2	0	2	1-2 years	A block with houses	Rent	4000	1
2	0	0	1-2 years	Compound shared wit	Rent	1000	1
3	1	3	Above 5 ye	House with a private y	Own		2
4	2	0	Above 5 ye	Compound shared wit	Rent	5500	2
2	0	0	Less than :	Compound shared wit	Rent	3500	1
1	0	0	Above 5 ye	House with a private y	Rent	6000	2
1	0	0	Above 5 ye	A block with houses	Rent	3000	1
2	4	0	3-5 years	Compound shared wit	Rent	6500	2
2	2	1	Less than :	A block with houses	Rent	6000	3
2	2	1	3-5 years	Compound shared wit	Rent	3000	1
3	5	0	3-5 years	Compound with family	Own		4
2	0	1	Less than :	Room in a multi-unit b	Rent	3000	1
2	0	0	Above 5 ye	A block with houses	Rent	2500	1
1	0	0	1-2 years	Compound shared wit	Rent	2500	1
2	0	1	3-5 years	Room in a multi-unit b	Rent	3000	1
2	3	0	Above 5 ye	Room in a multi-unit b	Rent	1000	1
2	0	1	Above 5 ye	A block with houses	Rent	2500	1
2	4	0	3-5 years	Compound shared wit	Rent	2500	1
4	4	0	Less than :	Compound shared wit	Rent	7500	2
4	3	1	Above 5 ye	Compound shared wit	Rent	4000	1
1	0	0	Less than :	Room in a multi-unit b	Rent	3500	1
2	0	2	Less than :	Room in a multi-unit b	Rent	4000	1
5	0	0	Above 5 ye	A block with houses	Own		3
5	3	0	Above 5 ye	Room in a multi-unit b	Rent	1500	2
5	3	0	Above 5 ye	Compound shared wit	Own		1
2	0	1	1-2 years	Compound shared wit	Rent	3000	1
2	0	0	Less than :	Compound shared wit	Rent	4000	1
1	0	0	Less than :	A block with houses	Rent	2300	1
2	2	0	Above 5 ye	Compound shared wit	Rent	2000	3
2	0	2	1-2 years	Compound shared wit	Rent	3000	1
2	3	1	Above 5 ye	House without compou	Own		2
2	4	1	Above 5 ye	Compound shared wit	Rent	3500	1
2	3	0	Above 5 ye	Room in a multi-unit b	Rent	4500	1
2	1	1	1-2 years	Compound shared wit	Rent	13000	2
1	2	1	3-5 years	Compound shared wit	Rent	2500	1
2	1	1	Above 5 ye	Compound shared wit	Rent	6000	4
2	2	1	Above 5 ye	Compound shared wit	Rent	5500	1
4	5	3	Above 5 ye	Compound shared wit	Own		5
1	0	0	Less than :	Compound shared wit	Rent	3000	1
3	4	0	Above 5 ye	A block with houses	Rent	2000	1
3	2	0	Above 5 ye	A block with houses	Rent	3500	2
2	0	2	3-5 years	Compound shared wit	Rent	3000	1
2	0	2	3-5 years	Compound shared wit	Rent	2000	1
2	4	2	Above 5 ye	House without compou	Own		2
3	1	1	1-2 years	A block with houses	Rent	2500	1
1	1	0	Less than :	Compound shared wit	Rent	1800	1

2	3	0	Above 5 ye	A block with houses	Rent	4500	1
2	1	1	Above 5 ye	Room in a multi-unit b	Rent	3000	1
2	1	0	1-2 years	Compound shared wit	Rent	4100	1
2	2	0	Above 5 ye	Compound shared wit	Rent	3500	1
2	2	1	Since birth	Compound with family	Rent	2500	1
2	0	0	Above 5 ye	Compound shared wit	Rent	5000	1
2	0	0	1-2 years	A block with houses	Rent	1950	1
4	1	0	Above 5 ye	Compound with family	Own		3
2	0	1	Less than :	Compound shared wit	Rent	1500	1
2	3	1	3-5 years	Room in a multi-unit b	Rent	3200	1
2	2	1	Above 5 ye	Compound shared wit	Rent	4000	2
1	2	0	Above 5 ye	Compound shared wit	Rent	3500	1
2	5	1	Above 5 ye	House without compo	Own		4
2	2	0	1-2 years	Compound shared wit	Rent	3100	2
2	1	1	1-2 years	A block with houses	Rent	5000	1
1	2	0	Above 5 ye	Room in a multi-unit b	Rent	3000	1
4	3	2	Less than :	Compound shared wit	Rent	4500	4
3	1	0	Above 5 ye	Compound shared wit	Rent	1500	1
1	4	1	3-5 years	Compound shared wit	Rent	3000	1
2	4	2	Above 5 ye	House without compo	Own		4
2	1	0	3-5 years	Compound shared wit	Rent	3000	1
4	1	1	Above 5 ye	A block with houses	Own		7
3	3	0	Above 5 ye	Compound Compound	Own		4
1	3	0	Above 5 ye	Compound shared wit	Rent	1500	1
2	0	0	Less than :	Compound shared wit	Rent	3700	1
2	0	1	1-2 years	A block with houses	Rent	2700	1
3	4	1	Above 5 ye	House with a private y	Rent	5000	4
2	1	2	Less than :	Room in a multi-unit b	Rent	3000	2
2	0	2	3-5 years	Compound shared wit	Rent	3000	1
2	6	2	Above 5 ye	Compound shared wit	Own		2
5	0	2	Above 5 ye	Compound shared wit	Rent	6000	1
2	0	0	1-2 years	Compound shared wit	Rent	2700	1
5	5	1	Less than :	Compound shared wit	Rent	3500	1
2	0	0	1-2 years	Room in a multi-unit b	Rent	2500	1
5	2	1	Above 5 ye	Compound shared wit	Own		3
2	2	0	Since birth	Compound shared wit	Rent	2000	1
2	3	1	Less than :	Compound shared wit	Rent	2800	2
2	1	1	3-5 years	Compound shared wit	Rent	2500	1
2	1	0	Above 5 ye	A block with houses	Rent	3500	2
1	1	0	Above 5 ye	A block with houses	Rent	2500	1
2	0	0	3-5 years	Room in a multi-unit b	Rent	4500	1
2	3	1	Above 5 ye	Compound shared wit	Rent	4500	1
2	0	1	1-2 years	Room in a multi-unit b	Rent	1500	1
5	1	0	Less than :	Room in a multi-unit b	Rent	8000	2
2	2	1	Above 5 ye	Compound shared wit	Rent	6000	2
2	0	0	Less than :	Compound shared wit	Rent	2000	1
2	3	0	Above 5 ye	Room in a multi-unit b	Rent	4500	1
2	0	2	Less than :	Compound shared wit	Rent	3500	1
2	0	1	Since birth	Compound shared wit	Rent	2000	1

3	1	2	1-2 years	A block with houses	Rent	4500	1
4	2	0	Above 5 ye	Compound with family	Own		4
1	8	0	3-5 years	A block with houses	Live here for free		1
1	2	1	1-2 years	Compound shared wit	Rent	2500	1
2	0	0	Less than :	Compound shared wit	Rent	3300	1
2	4	1	Above 5 ye	Compound with family	Rent	2500	2
1	0	0	Above 5 ye	Compound shared wit	Own		5
2	1	2	Less than :	Compound shared wit	Rent	2500	1
2	3	0	Above 5 ye	Compound shared wit	Rent	1500	1
2	1	0	Above 5 ye	Compound shared wit	Rent	2500	1
1	1	0	Above 5 ye	Compound shared wit	Rent	1500	1
2	1	1	3-5 years	Room in a multi-unit b	Rent	2500	1
2	1	0	3-5 years	A block with houses	Rent	2500	2
1	0	0	Less than :	A block with houses	Rent	2200	1
2	2	1	Above 5 ye	Compound with family	Rent	5000	2
4	2	1	Above 5 ye	Compound shared wit	Rent	2000	2
3	0	1	3-5 years	Room in a multi-unit b	Rent	2000	1
1	2	1	Less than :	House with a private y	Own		5
1	0	0	3-5 years	Compound shared wit	Rent	1700	1
2	0	0	Above 5 ye	A block with houses	Rent	2600	1
2	2	1	3-5 years	Compound shared wit	Rent	3000	2
2	0	3	Less than :	Compound shared wit	Rent	6500	1
3	2	1	3-5 years	House with a private y	Own		2
2	1	1	3-5 years	House with a private y	Rent	6500	3
6	0	0	Above 5 ye	Room in a multi-unit b	Own		3
2	1	2	1-2 years	Room in a multi-unit b	Live here for free		1
2	2	1	1-2 years	Compound shared wit	Rent	4200	1
4	2	0	Above 5 ye	Compound shared wit	Rent	2500	1
4	3	3	1-2 years	A block with houses	Rent	6500	1
2	6	0	3-5 years	A block with houses	Rent	2500	1
2	2	2	Above 5 ye	Room in a multi-unit b	Rent	4000	1
4	3	2	1-2 years	A block with houses	Rent	3000	1
2	1	1	3-5 years	A block with houses	Rent	1800	1
2	2	1	Above 5 ye	Compound shared wit	Rent	4000	3
1	1	1	Above 5 ye	Compound shared wit	Rent	3000	1
3	2	0	Above 5 ye	Compound shared wit	Own		5
2	1	1	3-5 years	A block with houses	Rent	3500	1
2	2	2	Above 5 ye	Compound shared wit	Rent	3000	2
3	3	0	Above 5 ye	Compound shared wit	Own		2
1	0	0	3-5 years	Compound shared wit	Rent	7000	3
2	2	2	3-5 years	Compound shared wit	Rent	2000	1
2	0	0	1-2 years	A block with houses	Rent	1500	1
2	0	1	1-2 years	Compound with family	Rent	3000	2
2	2	0	3-5 years	Compound shared wit	Rent	6000	1
5	2	0	Above 5 ye	Compound shared wit	Own		4
5	1	0	Above 5 ye	Compound shared wit	Own		3
3	4	0	3-5 years	Compound shared wit	Rent	20000	4
3	0	0	Above 5 ye	Compound with family	Own		5
2	0	0	Less than :	Room in a multi-unit b	Rent	3500	1

3	1	1	1-2 years	A block with houses	Live here for free		4
2	0	0	1-2 years	Compound shared wit	Rent	6000	1
4	4	0	3-5 years	Compound shared wit	Rent	7500	4
2	0	2	1-2 years	Compound shared wit	Rent	3400	1
5	0	0	Above 5 ye	House with a private y	Own		3
2	2	2	Above 5 ye	Room in a multi-unit b	Own		2
7	0	1	Above 5 ye	Compound shared wit	Rent	3600	1
2	1	2	Above 5 ye	Compound shared wit	Rent	3000	1
3	3	1	1-2 years	Compound Double roo	Rent	10000	2
2	5	0	Above 5 ye	A block with houses	Rent	4500	2
1	0	0	3-5 years	Compound shared wit	Rent	7100	1
2	2	0	Above 5 ye	A block with houses	Rent	4100	2
3	0	1	3-5 years	Compound shared wit	Rent	2500	1
2	3	1	Above 5 ye	Room in a multi-unit b	Rent	5000	1
2	2	1	Less than :	A block with houses	Rent	4500	1
2	1	1	Above 5 ye	Compound shared wit	Rent	4000	2
2	3	0	Above 5 ye	A block with houses	Rent	3000	2
1	0	0	Above 5 ye	Room in a multi-unit b	Rent	2500	1
2	0	2	Less than :	Compound shared wit	Rent	2500	2
2	3	2	Less than :	A block with houses	Rent	3000	2
2	0	1	3-5 years	A block with houses	Rent	8000	4
2	0	2	1-2 years	A block with houses	Rent	4000	1
3	3	1	3-5 years	Room in a multi-unit b	Rent	2000	1
2	0	0	1-2 years	Compound shared wit	Rent	2500	1
6	0	2	Above 5 ye	A block with houses	Rent	3000	2
2	1	4	Above 5 ye	A block with houses	Rent	1500	2
2	4	1	Above 5 ye	Compound shared wit	Rent	6500	4
2	0	1	3-5 years	A block with houses	Own		1
3	3	2	Above 5 ye	A block with houses	Rent	4000	3
1	0	1	3-5 years	Room in a multi-unit b	Own		3
2	0	0	Less than :	Compound shared wit	Rent	3000	1
3	1	2	Above 5 ye	House without compo	Own		4
2	3	0	Less than :	Compound shared wit	Rent	2000	1
2	1	0	Above 5 ye	Compound shared wit	Own		3
2	2	1	Above 5 ye	House with a private y	Own		1
2	2	0	Above 5 ye	A block with houses	Rent	2500	1
4	3	1	Above 5 ye	House with a private y	Own		2
2	1	0	3-5 years	Room in a multi-unit b	Rent	5000	1
2	0	0	Less than :	Room in a multi-unit b	Rent	3700	1
1	0	0	3-5 years	A block with houses	Live here for free		2
8	7	0	1-2 years	House without compo	Own		4
2	4	1	Above 5 ye	Compound shared wit	Rent	5000	3
2	0	1	3-5 years	Compound shared wit	Own		4
3	2	2	Above 5 ye	A block with houses	Rent	1700	2
2	0	0	Above 5 ye	House with a private y	Own		3
3	2	0	Above 5 ye	Compound shared wit	Rent	2500	1
2	0	0	Since birth	Compound shared wit	Own		4
3	1	0	Less than :	Compound shared wit	Rent	3000	1
4	4	2	1-2 years	Compound shared wit	Rent	2500	1

2	1	1	1-2 years	Compound shared wit	Rent	3500	1
4	3	0	1-2 years	House with a private y	Own		3
3	2	1	Since birth	House with a private y	Own		3
2	0	1	Above 5 ye	House without compou	Own		4
3	1	2	Above 5 ye	House without compou	Own		3
2	2	1	3-5 years	Compound shared wit	Rent	5500	1
2	1	1	Above 5 ye	House with a private y	Own		1
2	1	1	Above 5 ye	Room in a multi-unit b	Rent	2000	1
2	0	2	1-2 years	Compound shared wit	Rent	4000	2
2	4	3	Above 5 ye	Compound shared wit	Own		2
3	4	2	Above 5 ye	Compound shared wit	Rent	7000	2
2	2	0	Less than :	A block with houses	Rent	4000	2
3	1	1	Above 5 ye	Compound shared wit	Rent	14000	4
3	0	0	Less than :	Room in a multi-unit b	Rent	3500	1
4	1	0	Above 5 ye	Compound shared wit	Rent	3600	2
1	0	0	1-2 years	Compound shared wit	Rent	1500	1
3	3	0	Above 5 ye	House with a private y	Own		2
2	3	0	1-2 years	Compound shared wit	Rent	3000	1
2	0	1	3-5 years	Room in a multi-unit b	Rent	4000	1
2	1	1	1-2 years	Room in a multi-unit b	Rent	3600	1
3	3	0	Since birth	Compound shared wit	Own		5
2	1	0	3-5 years	Compound shared wit	Own		2
2	0	0	Above 5 ye	Compound shared wit	Rent	6800	4
3	6	3	Above 5 ye	House without compou	Own		5
2	1	1	1-2 years	A block with houses	Rent	2000	1
4	3	0	Above 5 ye	Compound shared wit	Rent	2500	2
2	1	1	Less than :	Room in a multi-unit b	Rent	5000	1
2	3	1	Above 5 ye	Compound shared wit	Rent	4000	2
2	3	1	Above 5 ye	Compound shared wit	Rent	5000	2
2	2	3	Less than :	Compound shared wit	Rent	3500	1
2	1	1	3-5 years	Compound shared wit	Rent	3500	1
2	0	2	Less than :	Room in a multi-unit b	Rent	3500	1
2	0	1	3-5 years	Room in a multi-unit b	Rent	2300	1
3	2	0	Above 5 ye	Compound shared wit	Rent	2000	1
2	0	1	Less than :	Room in a multi-unit b	Rent	3500	1
2	1	2	Above 5 ye	A block with houses	Rent	3000	1
7	0	0	Above 5 ye	Room in a multi-unit b	Rent	6500	1
2	3	2	3-5 years	A block with houses	Rent	3000	1
2	1	1	Less than :	House with a private y	Own		1
2	0	2	1-2 years	A block with houses	Rent	3000	1
2	1	1	Less than :	A block with houses	Rent	3500	1
4	1	0	Above 5 ye	Room in a multi-unit b	Own		3
1	0	0	Above 5 ye	A block with houses	Rent	1000	1
2	0	2	1-2 years	Room in a multi-unit b	Rent	3000	1
2	2	6	1-2 years	Compound shared wit	Rent	3000	1
2	0	1	Above 5 ye	A block with houses	Rent	5000	2
2	2	1	Above 5 ye	House with a private y	Own		2
2	3	0	1-2 years	Compound shared wit	Rent	2500	1
2	0	1	3-5 years	Compound shared wit	Rent	4000	1

2	2	1	Above 5 ye	Compound shared wit	Own		4
3	1	1	1-2 years	Room in a multi-unit b	Rent	4500	1
2	5	0	Above 5 ye	House without compo	Own		3
2	2	2	Above 5 ye	Compound shared wit	Rent	2550	1
2	1	1	Less than :	Room in a multi-unit b	Rent	4000	1
2	1	1	3-5 years	Compound shared wit	Rent	3000	1
3	3	3	Above 5 ye	Compound shared wit	Rent	2000	2
2	0	0	Less than :	Compound shared wit	Rent	3000	1
2	3	2	Above 5 ye	House with a private y	Own		4
2	0	1	Less than :	Compound shared wit	Rent	3000	1
2	1	0	Above 5 ye	Room in a multi-unit b	Rent	1000	1
2	0	2	Above 5 ye	Compound shared wit	Rent	3000	1
2	3	2	Less than :	A block with houses	Rent	2500	1
5	0	1	Above 5 ye	House with a private y	Own		3
1	2	0	Above 5 ye	A block with houses	Rent	1500	1
3	3	1	Less than :	Compound shared wit	Rent	2000	1
8	3	1	Above 5 ye	House with a private y	Own		9
2	0	1	1-2 years	Compound shared wit	Rent	5000	2
1	0	0	Above 5 ye	House without compo	Own		2
2	6	1	3-5 years	Compound shared wit	Rent	5800	2
1	0	0	Less than :	Room in a multi-unit b	Rent	3500	1
2	2	0	Above 5 ye	Compound with family	Own		3
3	4	1	3-5 years	Compound shared wit	Rent	2000	1
3	0	1	Above 5 ye	Compound shared wit	Live here for free		4
2	7	2	Above 5 ye	Compound with family	Own		4
2	1	0	1-2 years	Compound shared wit	Rent	1500	1
2	1	1	1-2 years	A block with houses	Rent	3000	1
2	0	2	1-2 years	A block with houses	Rent	3000	1
3	2	1	3-5 years	Compound shared wit	Rent	3000	1
3	2	1	Above 5 ye	Compound with family	Own		4
4	2	0	Above 5 ye	House without compo	Own		4
2	1	1	Less than :	Compound shared wit	Rent	3500	1
2	0	0	Less than :	Room in a multi-unit b	Rent	3600	1
2	1	0	Above 5 ye	Room in a multi-unit b	Rent	5500	1
3	0	1	1-2 years	Compound shared wit	Rent	2500	1
3	0	0	Less than :	Compound shared wit	Rent	3500	1
4	2	0	Above 5 ye	A block with houses	Rent	5000	3
2	0	0	Less than :	Compound shared wit	Rent	3000	1
2	1	1	Less than :	Room in a multi-unit b	Rent	4000	1
1	3	0	Above 5 ye	A block with houses	Rent	5000	1
2	1	2	1-2 years	A block with houses	Rent	6000	2
1	1	0	3-5 years	Compound with family	Own		3
2	1	1	Above 5 ye	Compound shared wit	Rent	3500	2
2	0	0	3-5 years	Compound shared wit	Rent	1500	1
2	1	2	1-2 years	A block with houses	Rent	2500	1
2	3	1	Above 5 ye	Compound shared wit	Rent	4500	2
6	4	3	Since birth	Compound shared wit	Rent	2400	1
2	0	1	Less than :	Compound shared wit	Rent	3500	1
2	1	1	3-5 years	Compound shared wit	Rent	7000	1

2	0	1	Above 5 ye	Compound shared wit	Rent	1500	1
2	1	1	3-5 years	A block with houses	Rent	1400	1
2	0	2	Above 5 ye	A block with houses	Rent	5000	2
2	3	2	1-2 years	A block with houses	Rent	3500	3
2	1	0	Above 5 ye	House without compo	Rent	1500	1
2	3	1	Above 5 ye	A block with houses	Rent	1500	1
2	2	0	Above 5 ye	Compound shared wit	Rent	3500	1
2	0	2	1-2 years	Compound shared wit	Rent	4000	2
2	0	1	Above 5 ye	Compound shared wit	Rent	2500	1
2	1	1	1-2 years	Compound shared wit	Rent	2600	1
2	1	0	Above 5 ye	House with a private y	Own		1
2	3	1	Above 5 ye	A block with houses	Rent	4400	2
2	3	0	1-2 years	Compound shared wit	Rent	3700	1
2	1	0	1-2 years	A block with houses	Rent	3500	2
2	0	0	Less than :	Compound shared wit	Rent	3500	1
2	3	0	3-5 years	Compound shared wit	Rent	3500	1
2	1	1	1-2 years	Compound shared wit	Rent	1800	1
5	2	2	3-5 years	Compound shared wit	Rent	2500	1
2	0	1	1-2 years	A block with houses	Rent	3000	1
2	3	2	3-5 years	Compound shared wit	Rent	3000	1
2	2	0	Above 5 ye	Compound shared wit	Own		2
2	1	1	Less than :	Room in a multi-unit b	Rent	4000	1
2	0	0	1-2 years	Compound shared wit	Rent	4000	1
2	0	0	1-2 years	Room in a multi-unit b	Rent	2000	1
4	2	2	Above 5 ye	A block with houses	Rent	6500	2
10	4	3	Above 5 ye	Compound with family	Own		3
2	0	2	1-2 years	Compound shared wit	Rent	3000	1
6	3	0	Above 5 ye	A block with houses	Rent	5000	3
1	0	0	1-2 years	Compound shared wit	Rent	4500	1
2	0	1	Less than :	A block with houses	Rent	2500	1
2	0	1	Above 5 ye	Compound shared wit	Rent	2000	1
3	4	3	Above 5 ye	Compound shared wit	Rent	3700	4
2	1	1	3-5 years	Compound shared wit	Rent	2500	1
1	0	0	Above 5 ye	Room in a multi-unit b	Rent	3000	1
3	2	0	3-5 years	Compound shared wit	Rent	3000	2
1	1	0	Less than :	A block with houses	Rent	2000	1
2	0	0	Above 5 ye	Compound shared wit	Rent	2500	1
3	3	0	Above 5 ye	Compound shared wit	Rent	5000	2
6	1	1	Above 5 ye	Compound shared wit	Rent	2000	1
8	6	3	Above 5 ye	Compound with family	Own		4
4	1	1	3-5 years	A block with houses	Rent	6000	2
2	1	1	Above 5 ye	Room in a multi-unit b	Rent	1500	1
3	1	1	Above 5 ye	Compound shared wit	Rent	2800	1
2	3	1	3-5 years	Compound shared wit	Rent	6000	2
2	0	1	1-2 years	Compound shared wit	Rent	2700	1
2	3	0	Above 5 ye	House with a private y	Own		2
4	3	0	Above 5 ye	House with a private y	Own		4
2	0	2	1-2 years	Compound shared wit	Rent	3000	1
2	4	0	Above 5 ye	A block with houses	Rent	5000	2

1	0	0	Above 5 ye	A block with houses	Live here for free		2
2	0	1	Less than :	A block with houses	Rent	3500	1
2	2	1	3-5 years	A block with houses	Rent	2700	1
2	0	0	3-5 years	Compound shared wit	Own		3
3	1	1	Above 5 ye	Compound shared wit	Rent	4500	2
3	2	2	1-2 years	A block with houses	Rent	5500	2
7	1	0	Above 5 ye	House with a private y	Own		2
2	2	2	Above 5 ye	A block with houses	Rent	5000	1
3	0	0	Above 5 ye	Compound shared wit	Rent	4000	3
1	1	0	Above 5 ye	Compound shared wit	Rent	1000	1
3	4	0	Above 5 ye	A block with houses	Rent	3000	2
2	0	1	Since birth	Room in a multi-unit b	Live here for free		1
1	0	0	Above 5 ye	Room in a multi-unit b	Rent	3000	1
2	1	1	Above 5 ye	A block with houses	Rent	5000	2
2	0	0	1-2 years	Compound shared wit	Rent	3100	1
2	2	0	Above 5 ye	Compound shared wit	Rent	10000	2
2	4	0	Less than :	Compound shared wit	Rent	2500	1
2	1	1	3-5 years	Compound shared wit	Rent	5500	3
2	1	2	Above 5 ye	Compound shared wit	Own		2
5	5	2	Above 5 ye	House with a private y	Own		3
2	3	0	Above 5 ye	Compound shared wit	Rent	4500	1
6	0	0	1-2 years	A block with houses	Rent	10000	2
2	2	1	1-2 years	Compound shared wit	Rent	9000	1
4	2	0	Above 5 ye	Compound with family	Own		4
2	0	0	Above 5 ye	Compound shared wit	Rent	4000	2
1	0	0	3-5 years	Compound shared wit	Rent	3500	1
1	0	0	Less than :	Compound shared wit	Rent	5000	1
2	0	0	Less than :	Compound shared wit	Rent	3000	1
2	2	0	Above 5 ye	Compound shared wit	Rent	3500	1
2	3	0	3-5 years	House with a private y	Own		2
3	2	0	Above 5 ye	House with a private y	Own		3
2	1	1	Above 5 ye	Compound shared wit	Rent	3500	1
1	0	0	Less than :	Compound shared wit	Rent	2000	1
2	3	2	Above 5 ye	Compound with family	Rent	5000	4
5	0	0	Above 5 ye	House without compo	Own		3
5	2	2	Since birth	Compound with family	Own		3
2	0	1	3-5 years	Room in a multi-unit b	Rent	4000	1
4	1	1	Above 5 ye	Compound shared wit	Rent	2000	1
1	0	0	Less than :	Room in a multi-unit b	Rent	2000	1
3	2	0	Above 5 ye	Compound with family	Own		2
2	3	1	3-5 years	Compound with family	Rent	1500	1
2	0	1	3-5 years	A block with houses	Rent	3500	1
2	0	1	3-5 years	A block with houses	Rent	4000	2
1	2	2	Above 5 ye	Compound shared wit	Rent	1000	1
1	2	2	Less than :	A block with houses	Rent	3500	1
1	1	0	3-5 years	Compound shared wit	Rent	6500	3
3	0	1	Less than :	Compound shared wit	Rent	3000	1
1	0	0	Since birth	Room in a multi-unit b	Own		2
3	0	0	Above 5 ye	Compound shared wit	Own		5

2	0	0	Less than :	Room in a multi-unit b	Rent	1500	1
2	1	2	Above 5 ye	Compound shared wit	Rent	3500	1
4	3	0	Above 5 ye	Compound shared wit	Rent	7500	3
1	0	0	3-5 years	Compound shared wit	Rent	3500	1
2	0	2	Above 5 ye	House with a private y	Own		3
2	2	1	Above 5 ye	House without compo	Own		3
3	4	0	Above 5 ye	Compound shared wit	Rent	2000	3
4	1	0	Above 5 ye	Compound shared wit	Own		2
2	0	1	Above 5 ye	House with a private y	Rent	3500	1
2	3	1	Above 5 ye	House with a private y	Own		3
2	2	0	3-5 years	Compound shared wit	Rent	2500	1
2	2	0	3-5 years	Compound shared wit	Rent	5500	1
3	2	1	Above 5 ye	Room in a multi-unit b	Rent	4500	2
2	0	0	Above 5 ye	Compound shared wit	Rent	3200	1
2	3	0	Above 5 ye	A block with houses	Rent	2000	1
2	0	2	Less than :	Compound shared wit	Rent	2500	1
2	2	0	1-2 years	A block with houses	Rent	4500	2
3	1	0	Above 5 ye	Compound shared wit	Rent	5000	5
2	2	0	Less than :	Compound shared wit	Rent	3500	1
1	0	0	Above 5 ye	Room in a multi-unit b	Own		2
6	0	0	Above 5 ye	House with a private y	Own		4
2	0	0	Less than :	Compound shared wit	Rent	3000	1
2	2	0	3-5 years	Compound shared wit	Rent	2500	1
1	0	0	1-2 years	House without compo	Live here for free		3
1	0	0	Above 5 ye	A block with houses	Rent	4000	2
2	1	0	1-2 years	Compound shared wit	Rent	4500	2
2	0	0	Less than :	Compound shared wit	Rent	3000	1
2	2	1	Above 5 ye	A block with houses	Rent	3000	2
2	2	2	3-5 years	Compound shared wit	Rent	4000	1
2	2	0	Less than :	Room in a multi-unit b	Rent	4500	1
1	4	2	1-2 years	A block with houses	Rent	1500	2
3	2	1	3-5 years	Compound shared wit	Rent	2500	2
2	1	1	3-5 years	Compound shared wit	Rent	3100	1
2	0	0	Above 5 ye	Compound shared wit	Rent	3500	1
2	2	0	Above 5 ye	House without compo	Own		2
2	0	0	Less than :	A block with houses	Rent	3050	1
3	1	1	1-2 years	Compound shared wit	Rent	1500	1
3	2	0	Since birth	House without compo	Own		7
3	3	2	Above 5 ye	A block with houses	Own		2
1	0	0	1-2 years	Room in a multi-unit b	Rent	5000	1
6	3	0	Above 5 ye	Compound shared wit	Rent	2500	2
2	1	2	Above 5 ye	A block with houses	Rent	1700	1
3	0	0	Above 5 ye	House without compo	Rent	3500	1
2	2	3	Above 5 ye	House with a private y	Own		1
2	0	0	1-2 years	A block with houses	Rent	3500	1
2	2	1	Less than :	Compound shared wit	Rent	2000	1
1	2	1	1-2 years	A block with houses	Rent	4000	1
1	0	0	Above 5 ye	A block with houses	Live here for free		1
7	1	1	Above 5 ye	House without compo	Own		7

2	2	1	Above 5 ye	Room in a multi-unit b	Rent	5000	1
2	1	0	Above 5 ye	A block with houses	Rent	2000	2
2	0	0	3-5 years	Room in a multi-unit b	Rent	3500	1
2	3	1	Above 5 ye	House without compo	Own		2
2	0	1	1-2 years	Compound shared wit	Rent	1700	1
2	2	0	Above 5 ye	Compound shared wit	Rent	2000	1
2	2	1	Less than :	Room in a multi-unit b	Rent	5000	1
2	0	1	Less than :	A block with houses	Rent	2700	1
3	1	0	Above 5 ye	A block with houses	Rent	3500	1
2	2	1	1-2 years	Compound shared wit	Rent	2000	1
1	2	2	Above 5 ye	Room in a multi-unit b	Own		1
2	2	0	3-5 years	A block with houses	Rent	5000	3
2	2	2	Above 5 ye	A block with houses	Rent	5000	2
2	1	0	1-2 years	Room in a multi-unit b	Rent	5000	1
2	0	2	1-2 years	Room in a multi-unit b	Rent	3500	1
2	0	0	Less than :	Compound shared wit	Rent	3000	1
2	0	3	Less than :	Compound shared wit	Rent	2000	1
2	1	1	3-5 years	A block with houses	Rent	2000	1
2	3	0	Above 5 ye	Compound shared wit	Rent	2200	1
2	0	1	3-5 years	Compound shared wit	Rent	4000	1
2	0	1	3-5 years	Compound shared wit	Rent	2500	1
2	2	1	3-5 years	Compound shared wit	Rent	3000	1
2	0	1	1-2 years	Compound shared wit	Rent	4000	2
2	0	1	Less than :	Compound shared wit	Rent	3000	1
2	2	1	1-2 years	Compound shared wit	Rent	3500	1
2	0	0	3-5 years	Compound shared wit	Rent	2800	1
2	4	1	Above 5 ye	Compound shared wit	Rent	2700	1
2	0	2	Less than :	A block with houses	Rent	3000	1
2	0	0	Above 5 ye	Compound shared wit	Rent	4000	2
2	0	0	Less than :	A block with houses	Rent	3000	1
3	0	1	Above 5 ye	House without compo	Own		6
2	0	1	Less than :	A block with houses	Rent	2000	1
5	0	0	Above 5 ye	A block with houses	Own		3
3	2	1	3-5 years	Room in a multi-unit b	Rent	3500	1
2	1	1	Above 5 ye	Compound with family	Rent	1200	1
2	1	2	1-2 years	A block with houses	Rent	4500	3
1	3	1	Above 5 ye	House without compo	Own		2
2	0	0	Less than :	Room in a multi-unit b	Rent	3100	1
2	1	0	3-5 years	A block with houses	Rent	3500	1
2	2	1	Above 5 ye	Compound shared wit	Rent	5000	2
2	2	2	Above 5 ye	Compound shared wit	Rent	2500	1
4	1	0	Above 5 ye	Compound shared wit	Rent	5000	2
2	2	0	Above 5 ye	Compound shared wit	Rent	6000	2
2	0	0	Less than :	Room in a multi-unit b	Rent	3500	1
4	0	0	Above 5 ye	A block with houses	Rent	3000	1
3	0	0	Less than :	A block with houses	Rent	2000	1
1	0	0	Less than :	Compound shared wit	Rent	4500	1
5	5	1	Above 5 ye	House with a private y	Own		3
2	1	1	1-2 years	Compound shared wit	Rent	3000	1

2	2	0	Above 5 ye	Compound shared wit	Own		5
3	2	0	Above 5 ye	House without compo	Own		5
2	1	1	1-2 years	Compound shared wit	Rent	3000	1
2	2	1	3-5 years	Compound with family	Own		6
2	1	1	Above 5 ye	Compound shared wit	Rent	7000	1
2	0	1	Above 5 ye	A block with houses	Rent	3000	1
2	0	0	Above 5 ye	Room in a multi-unit b	Rent	3500	1
2	1	1	1-2 years	A block with houses	Rent	4500	2
3	3	1	Since birth	Compound shared wit	Own		7
2	0	1	1-2 years	Compound shared wit	Rent	3600	1
2	4	1	Above 5 ye	Compound shared wit	Rent	3000	1
1	0	0	Above 5 ye	House without compo	Own		2
2	0	1	3-5 years	Room in a multi-unit b	Rent	3200	1
1	2	0	3-5 years	A block with houses	Rent	3500	1
2	2	1	Above 5 ye	Compound shared wit	Rent	4000	1
2	0	0	Above 5 ye	A block with houses	Own		3
2	2	0	Above 5 ye	House without compo	Rent	3000	3
2	1	1	1-2 years	Compound shared wit	Rent	3500	1
2	0	0	Above 5 ye	A block with houses	Rent	2500	2
3	0	0	Since birth	Compound with family	Own		8
2	2	2	Above 5 ye	House without compo	Own		3
2	0	0	Less than :	Compound shared wit	Rent	3600	1
7	1	1	Above 5 ye	House without compo	Own		6
1	1	0	Since birth	Compound shared wit	Own		6
2	1	1	Less than :	Compound shared wit	Rent	5600	1
2	1	0	Above 5 ye	Compound shared wit	Rent	2550	1
6	0	0	Above 5 ye	Compound shared wit	Own		3
3	1	1	3-5 years	A block with houses	Rent	2700	1
2	2	2	Above 5 ye	Compound shared wit	Rent	1500	1
3	2	2	Above 5 ye	House without compo	Own		2
1	0	2	1-2 years	Compound shared wit	Rent	1500	1
2	1	1	Above 5 ye	A block with houses	Rent	4000	1
6	0	0	Above 5 ye	A block with houses	Own		3
2	2	0	3-5 years	A block with houses	Rent	2500	2
2	2	1	Less than :	Room in a multi-unit b	Rent	4000	1
2	1	1	Less than :	Room in a multi-unit b	Rent	3500	1
2	3	0	Above 5 ye	Room in a multi-unit b	Own		2
3	5	2	3-5 years	Compound shared wit	Rent	3000	2
2	0	0	Above 5 ye	House with a private y	Own		6
4	2	0	Above 5 ye	Compound shared wit	Rent	4000	1
4	1	0	Above 5 ye	Compound shared wit	Rent	1500	1
3	0	0	Above 5 ye	Compound shared wit	Rent	2000	1
2	0	3	Above 5 ye	A block with houses	Rent	2800	1
2	0	1	3-5 years	Room in a multi-unit b	Live here for free		1
2	0	2	3-5 years	Compound shared wit	Rent	4700	2
2	1	0	3-5 years	Compound shared wit	Rent	2000	1
4	0	1	Above 5 ye	House without compo	Rent	3500	1
3	1	1	Less than :	Compound shared wit	Own		3
2	2	0	Above 5 ye	Compound shared wit	Rent	1500	2

2	1	2	Above 5 ye	A block with houses	Rent	2000	1
2	0	1	1-2 years	Compound shared wit	Rent	5600	4
1	0	2	Less than :	Compound shared wit	Rent	1500	1
2	1	1	3-5 years	A block with houses	Rent	2000	1
2	0	1	Less than :	Compound shared wit	Rent	5000	1
2	2	2	Less than :	Compound shared wit	Rent	1800	1
3	0	0	Above 5 ye	House with a private y	Own		3
4	0	0	Above 5 ye	House with a private y	Own		3
2	0	1	3-5 years	A block with houses	Rent	4500	1
2	3	1	3-5 years	A block with houses	Rent	3000	1
2	3	1	Above 5 ye	House without compo	Rent	12000	3
1	0	0	Less than :	Room in a multi-unit b	Rent	2000	1
2	0	2	1-2 years	Compound shared wit	Rent	1700	1
2	2	1	Above 5 ye	House without compo	Rent	1300	1
5	3	1	3-5 years	House with a private y	Own		5
3	0	0	Above 5 ye	Compound shared wit	Rent	4000	2
1	0	0	1-2 years	Compound shared wit	Rent	3000	1
2	1	3	1-2 years	A block with houses	Rent	3500	2
2	0	1	1-2 years	Compound shared wit	Rent	2500	1
4	5	1	Since birth	Compound shared wit	Rent	3000	1
4	1	1	Above 5 ye	Compound shared wit	Rent	1500	1
3	1	0	1-2 years	Compound shared wit	Rent	3000	1
3	0	0	1-2 years	Compound shared wit	Rent	3800	1
2	1	1	Above 5 ye	House with a private y	Own		2
2	7	2	Less than :	Compound shared wit	Rent	3000	1

q17	q18a	q18b	q19a	q19b	q20a	q20b	q21	q22
In the corn	Cement		Stone		Iron sheet (corrugated		Yes	No
In a separa	Cement		Iron sheets(mabati)		Iron sheet (corrugated		Yes	Yes
In the corn	Polished wood		Stone		Iron sheet (corrugated		Yes	Yes
In the corn	Cement		Mud		Iron sheet (corrugated		Yes	Yes
In a separa	Cement		Stone		Iron sheet (corrugated		Yes	Yes
In the corn	Cement		Iron sheets(mabati)		Iron sheet (corrugated		Yes	Yes
In the corn	Cement		Stone		Iron sheet (corrugated		Yes	No
In another	Cement		Stone		Iron sheet (corrugated		Yes	Yes
In the corn	Cement		Stone		Iron sheet (corrugated		Yes	Yes
In a separa	Polished wood		Cemented mud		Iron sheet (corrugated		Yes	Yes
In the corn	Cement		Iron sheets(mabati)		Metal sheets/tin		Yes	Yes
In the corn	Cement		Cemented mud		Iron sheet (corrugated		Yes	Yes
In the corn	Cement		Stone		Iron sheet (corrugated		Yes	Yes
In the corn	Cement		Stone		Metal sheets/tin		Yes	Yes
In the corn	Cement		Cemented mud		Iron sheet (corrugated		Yes	Yes
In the corn	Cement		Concrete blocks		Iron sheet (corrugated		Yes	Yes
In the corn	Cement		Iron sheets(mabati)		Iron sheet (corrugated		Yes	Yes
In the corn	Cement		Mud		Iron sheet (corrugated		No	Yes
In the corn	Polished wood		Concrete blocks		Iron sheet (corrugated		Yes	Yes
In a separa	Natural floor		Mud		Iron sheet (corrugated		Yes	Yes
In the corn	Cement		Iron sheets(mabati)		Iron sheet (corrugated		No window	Yes
In the corn	Cement		Concrete blocks		Iron sheet (corrugated		Yes	Yes
In the corn	Cement		Iron sheets(mabati)		Iron sheet (corrugated		No window	Yes
Outside in	Natural floor		Mud		Iron sheet (corrugated		No	No
In a separa	Cement		Cemented mud		Iron sheet (corrugated		Yes	Yes
In the corn	Cement		Iron sheets(mabati)		Iron sheet (corrugated		Yes	Yes
In a separa	Cement		Stone		Iron sheet (corrugated		Yes	Yes
In the corn	Cement		Concrete blocks		Iron sheet (corrugated		Yes	Yes
In a separa	Polished wood		Mud/Stone/Bricks mix		Iron sheet (corrugated		Yes	Yes
In a separa	Natural floor		Mud		Iron sheet (corrugated		No	Yes
In the corn	Cement		Cemented mud		Iron sheet (corrugated		Yes	Yes
In a separa	Cement		Concrete blocks		Iron sheet (corrugated		No	Yes
In the corn	Cement		Concrete blocks		Iron sheet (corrugated		Yes	Yes
In the corn	Cement		Concrete blocks		Iron sheet (corrugated		No	Yes
In the corn	Cement		Concrete blocks		Iron sheet (corrugated		No	Yes
Outside in	Natural floor		Mud		Iron sheet (corrugated		No	Yes
In the corn	Cement		Cemented mud		Iron sheet (corrugated		Yes	Yes
In the corn	Natural floor		Mud		Iron sheet (corrugated		No	Yes
In the corn	Cement		Concrete blocks		Iron sheet (corrugated		Yes	Yes
In a separa	Cement		Stone		Iron sheet (corrugated		Yes	Yes
In another	Natural floor		Mud		Iron sheet (corrugated		Yes	Yes
In the corn	Natural floor		Mud		Iron sheet (corrugated		Yes	Yes
In a separa	Cement		Concrete blocks		Iron sheet (corrugated		No	Yes
In a separa	Cement		Concrete blocks		Iron sheet (corrugated		No	No
In the corn	Cement		Concrete blocks		Iron sheet (corrugated		Yes	Yes
In a separa	Polished wood		Concrete blocks		Iron sheet (corrugated		Yes	Yes
In the corn	Cement		Concrete blocks		Iron sheet (corrugated		Yes	Yes
In the corn	Cement		Concrete blocks		Iron sheet (corrugated		No	Yes

In the corn	Cement		Stone	Iron sheet (corrugated	Yes	Yes
In the corn	Cement		Stone	Iron sheet (corrugated	Yes	Yes
In a separa	Natural floor		Mud	Iron sheet (corrugated	No	Yes
In a separa	Natural floor		Stone	Iron sheet (corrugated	Yes	Yes
In a separa	Cement		Concrete blocks	Iron sheet (corrugated	No	Yes
In the corn	Cement		Iron sheets(mabati)	Iron sheet (corrugated	Yes	Yes
Outside in	Cement		Mud	Iron sheet (corrugated	Yes	Yes
In another	Natural floo	Partly earth	Mud	Iron sheet (corrugated	Yes	Yes
In a separa	Cement		Concrete blocks	Iron sheet (corrugated	Yes	Yes
In a separa	Cement		Cemented mud	Iron sheet (corrugated	Yes	Yes
In the corn	Cement		Stone	Iron sheet (corrugated	Yes	Yes
In the corn	Cement		Concrete blocks	Iron sheet (corrugated	Yes	Yes
In a separa	Cement		Cemented mud	Iron sheet (corrugated	Yes	Yes
In a separa	Cement		Concrete blocks	Iron sheet (corrugated	Yes	Yes
In the corn	Natural floor		Mud	Iron sheet (corrugated	No	Yes
In a separa	Polished wood		Concrete blocks	Iron sheet (corrugated	Yes	Yes
In a separa	Cement		Concrete blocks	Iron sheet (corrugated	Yes	Yes
In a separa	Polished wood		Concrete blocks	Iron sheet (corrugated	Yes	Yes
In the corn	Cement		Concrete blocks	Iron sheet (corrugated	Yes	Yes
In the corn	Cement		Cemented mud	Iron sheet (corrugated	Yes	Yes
In the corn	Cement		Cemented mud	Iron sheet (corrugated	Yes	Yes
In the corn	Cement		Stone	Metal sheets/tin	Yes	Yes
In the corn	Cement		Iron sheets(mabati)	Iron sheet (corrugated	Yes	Yes
In another	Cement		Mud	Iron sheet (corrugated	Yes	Yes
In the corn	Cement		Iron sheets(mabati)	Metal sheets/tin	Yes	Yes
In a separa	Natural floor		Mud	Iron sheet (corrugated	No	Yes
In the corn	Cement		Stone	Iron sheet (corrugated	Yes	No
In the corn	Cement		Cemented mud	Iron sheet (corrugated	Yes	Yes
In the corn	Natural floor		Mud	Iron sheet (corrugated	No	Yes
In the corn	Natural floor		Stone	Iron sheet (corrugated	No window	Yes
In the corn	Cement		Stone	Iron sheet (corrugated	Yes	Yes
In the corn	Natural floor		Mud	Iron sheet (corrugated	Yes	Yes
In a separa	Cement		Concrete blocks	Iron sheet (corrugated	No	Yes
In a separa	Cement		Cemented mud	Iron sheet (corrugated	No	Yes
In the corn	Cement	Vinyl(PVC)	Mud	Iron sheet (corrugated	Yes	Yes
In a separa	Cement		Cemented mud	Iron sheet (corrugated	Yes	Yes
In the corn	Natural floor		Cemented mud	Iron sheet (corrugated	Yes	Yes
In the corn	Cement		Concrete blocks	Iron sheet (corrugated	No	Yes
In the corn	Cement		Cemented mud	Iron sheet (corrugated	Yes	Yes
In another	Cement		Stone	Iron sheet (corrugated	Yes	Yes
In the corn	Cement		Concrete blocks	Iron sheet (corrugated	Yes	Yes
In a separa	Cement		Cemented mud	Iron sheet (corrugated	Yes	Yes
In a separa	Natural floor		Mud	Iron sheet (corrugated	Yes	Yes
In another	Cement		Stone	Iron sheet (corrugated	Yes	Yes
Outside in	Cement		Stone	Iron sheet (corrugated	Yes	Yes
In the corn	Cement		Stone	Iron sheet (corrugated	Yes	Yes
In a separa	Cement		Stone	Iron sheet (corrugated	Yes	Yes
In a separa	Cement		Concrete blocks	Iron sheet (corrugated	No	Yes
In the corn	Cement		Cemented mud	Iron sheet (corrugated	No window	Yes

In the corn	Cement	Iron sheets(mabati)	Iron sheet (corrugated	Yes	Yes
In the corn	Cement	Stone	Iron sheet (corrugated	Yes	Yes
In the corn	Natural floor	Mud	Iron sheet (corrugated	No window	Yes
In another	Cement	Stone	Iron sheet (corrugated	Yes	Yes
In a separa	Natural floor	Concrete blocks	Iron sheet (corrugated	Yes	Yes
In the corn	Polished wood	Concrete blocks	Iron sheet (corrugated	No	Yes
In a separa	Cement	Mud/Stone/Bricks mix	Iron sheet (corrugated	Yes	Yes
In the corn	Polished wood	Stone	Iron sheet (corrugated	Yes	Yes
In another	Natural floor	Mud	Iron sheet (corrugated	Yes	Yes
Outside in	Cement	Concrete blocks	Iron sheet (corrugated	No	Yes
In the corn	Cement	Iron sheets(mabati)	Iron sheet (corrugated	Yes	Yes
In the corn	Cement	Iron sheets(mabati)	Metal sheets/tin	Yes	Yes
In a separa	Cement	Concrete blocks	Iron sheet (corrugated	Yes	Yes
In a separa	Cement	Stone	Iron sheet (corrugated	Yes	Yes
In another	Cement	Iron sheets(mabati)	Iron sheet (corrugated	Yes	Yes
In a separa	Natural floor	Mud	Iron sheet (corrugated	Yes	Yes
In a separa	Cement	Cemented mud	Iron sheet (corrugated	No	Yes
In another	Polished wood	Concrete blocks	Iron sheet (corrugated	No	Yes
In a separa	Cement	Cemented mud	Iron sheet (corrugated	Yes	Yes
In a separa	Cement	Stone	Iron sheet (corrugated	Yes	Yes
In another	Natural floor	Mud	Iron sheet (corrugated	Yes	Yes
In the corn	Cement	Stone	Iron sheet (corrugated	Yes	Yes
In the corn	Cement	Concrete blocks	Iron sheet (corrugated	No	Yes
In a separa	Natural floor	Mud	Iron sheet (corrugated	Yes	Yes
In a separa	Cement	Concrete blocks	Iron sheet (corrugated	No	Yes
In the corn	Cement	Cemented mud	Iron sheet (corrugated	Yes	Yes
In the corn	Cement	Stone	Iron sheet (corrugated	Yes	Yes
In a separa	Polished wood	Concrete blocks	Iron sheet (corrugated	Yes	Yes
In a separa	Cement	Concrete blocks	Iron sheet (corrugated	Yes	Yes
In a separa	Cement	Concrete blocks	Iron sheet (corrugated	Yes	Yes
In the corn	Cement	Stone	Iron sheet (corrugated	Yes	Yes
In a separa	Polished wood	Concrete blocks	Iron sheet (corrugated	Yes	Yes
Outside in	Cement	Stone	Iron sheet (corrugated	Yes	Yes
In a separa	Cement	Cemented mud	Iron sheet (corrugated	Yes	Yes
Outside in	Cement	Concrete blocks	Iron sheet (corrugated	No	Yes
In a separa	Cement	Mud/Stone/Bricks mix	Iron sheet (corrugated	Yes	Yes
In the corn	Cement	Iron sheets(mabati)	Iron sheet (corrugated	No window	Yes
In a separa	Cement	Cemented mud	Iron sheet (corrugated	Yes	Yes
In another	Natural floor	Mud	Iron sheet (corrugated	Yes	Yes
In the corn	Cement	Concrete blocks	Iron sheet (corrugated	No	Yes
In the corn	Cement	Concrete blocks	Iron sheet (corrugated	No	Yes
In a separa	Polished wood	Stone	Iron sheet (corrugated	Yes	Yes
In the corn	Cement	Concrete blocks	Iron sheet (corrugated	Yes	Yes
In the corn	Cement	Concrete blocks	Iron sheet (corrugated	Yes	No
In a separa	Cement	Iron sheets(mabati)	Iron sheet (corrugated	No window	Yes
In the corn	Cement	Stone	Iron sheet (corrugated	Yes	Yes
In a separa	Natural floor	Stone	Iron sheet (corrugated	Yes	Yes
In the corn	Cement	Concrete blocks	Iron sheet (corrugated	No	Yes
In a separa	Cement	Cemented mud	Iron sheet (corrugated	Yes	Yes

Outside in	Natural floor	Mud	Iron sheet (corrugated	No	Yes
In a separa	Natural floor	Mud	Iron sheet (corrugated	No	Yes
In the corn	Cement	Cemented mud	Iron sheet (corrugated	Yes	Yes
In the corn	Cement	Concrete blocks	Iron sheet (corrugated	Yes	Yes
In a separa	Natural floor	Mud	Iron sheet (corrugated	Yes	Yes
In the corn	Cement	Stone	Iron sheet (corrugated	Yes	Yes
In the corn	Cement	Iron sheets(mabati)	Metal sheets/tin	Yes	Yes
In a separa	Cement	Cemented mud	Iron sheet (corrugated	Yes	Yes
In the corn	Cement	Iron sheets(mabati)	Iron sheet (corrugated	Yes	Yes
In the corn	Cement	Iron sheets(mabati)	Iron sheet (corrugated	Yes	Yes
In the corn	Cement	Cemented mud	Iron sheet (corrugated	Yes	Yes
In the corn	Cement	Cemented mud	Iron sheet (corrugated	Yes	Yes
In the corn	Natural floor	Mud	Iron sheet (corrugated	Yes	Yes
In the corn	Cement	Concrete blocks	Iron sheet (corrugated	Yes	Yes
In a separa	Cement	Concrete blocks	Iron sheet (corrugated	No	Yes
In a separa	Cement	Cemented mud	Iron sheet (corrugated	Yes	Yes
In a separa	Cement	Cemented mud	Iron sheet (corrugated	Yes	Yes
In the corn	Polished wood	Concrete blocks	Iron sheet (corrugated	No	Yes
In the corn	Cement	Concrete blocks	Iron sheet (corrugated	No	Yes
In the corn	Cement	Concrete blocks	Iron sheet (corrugated	No	Yes
In the corn	Cement	Cemented mud	Iron sheet (corrugated	Yes	Yes
In a separa	Cement	Concrete blocks	Iron sheet (corrugated	No	Yes
In another	Natural floor	Mud/Stone/Bricks mix	Iron sheet (corrugated	Yes	Yes
In a separa	Polished wood	Mud/Stone/Bricks mix	Iron sheet (corrugated	Yes	Yes
In a separa	Cement	Cemented mud	Iron sheet (corrugated	Yes	Yes
Outside in	Natural floor	Mud	Iron sheet (corrugated	No	Yes
In a separa	Cement	Cemented mud	Iron sheet (corrugated	Yes	Yes
In the corn	Natural floor	Mud	Iron sheet (corrugated	Yes	Yes
In the corn	Natural floor	Mud	Iron sheet (corrugated	No	Yes
In another	Natural floor	Concrete blocks	Iron sheet (corrugated	Yes	Yes
In a separa	Cement	Stone	Iron sheet (corrugated	Yes	Yes
In the corn	Cement	Concrete blocks	Iron sheet (corrugated	Yes	Yes
In the corn	Natural floor	Mud	Iron sheet (corrugated	No window	Yes
In a separa	Natural floor	Stone	Iron sheet (corrugated	Yes	Yes
In a separa	Polished wood	Stone	Iron sheet (corrugated	Yes	Yes
In the corn	Natural floor	Concrete blocks	Iron sheet (corrugated	No	Yes
In a separa	Natural floor	Concrete blocks	Iron sheet (corrugated	No	Yes
In a separa	Polished wood	Cemented mud	Iron sheet (corrugated	Yes	Yes
In the corn	Cement	Concrete blocks	Iron sheet (corrugated	Yes	Yes
In a separa	Natural floor	Concrete blocks	Iron sheet (corrugated	Yes	Yes
In a separa	Cement	Mud	Iron sheet (corrugated	Yes	Yes
In another	Cement	Mud	Iron sheet (corrugated	Yes	Yes
In another	Cement	Cemented mud	Iron sheet (corrugated	Yes	Yes
In the corn	Cement	Concrete blocks	Iron sheet (corrugated	Yes	Yes
Outside in	Cement	Concrete blocks	Iron sheet (corrugated	No	Yes
In the corn	Cement	Concrete blocks	Iron sheet (corrugated	No	Yes
Outside in	Cement	Mud	Iron sheet (corrugated	No	Yes
In the corn	Cement	Cemented mud	Iron sheet (corrugated	Yes	Yes
In the corn	Cement	Concrete blocks	Iron sheet (corrugated	No	Yes

In a separa	Cement	Concrete blocks	Iron sheet (corrugated	Yes	Yes
In a separa	Polished wood	Stone	Iron sheet (corrugated	Yes	Yes
In a separa	Cement	Cemented mud	Iron sheet (corrugated	Yes	Yes
In a separa	Cement	Concrete blocks	Iron sheet (corrugated	No	Yes
In the corn	Natural floor	Mud	Iron sheet (corrugated	No window	Yes
In the corn	Cement	Cemented mud	Iron sheet (corrugated	Yes	Yes
In the corn	Natural floor	Mud	Iron sheet (corrugated	No	Yes
In a separa	Cement	Concrete blocks	Iron sheet (corrugated	No	Yes
In a separa	Cement	Concrete blocks	Iron sheet (corrugated	No	Yes
In the corn	Cement	Concrete blocks	Iron sheet (corrugated	Yes	Yes
In the corn	Cement	Concrete blocks	Iron sheet (corrugated	No	Yes
In a separa	Polished wood	Stone	Metal sheets/tin	Yes	Yes
In another	Cement	Cemented mud	Iron sheet (corrugated	Yes	Yes
In the corn	Cement	Iron sheets(mabati)	Iron sheet (corrugated	Yes	Yes
In the corn	Cement	Stone	Iron sheet (corrugated	Yes	Yes
In the corn	Cement	Concrete blocks	Iron sheet (corrugated	Yes	Yes
Outside in	Natural floor	Mud	Iron sheet (corrugated	No	Yes
In the corn	Cement	Iron sheets(mabati)	Iron sheet (corrugated	Yes	Yes
In the corn	Cement	Iron sheets(mabati)	Metal sheets/tin	Yes	Yes
In a separa	Cement	Stone	Iron sheet (corrugated	Yes	Yes
In a separa	Natural floor	Concrete blocks	Iron sheet (corrugated	Yes	Yes
In the corn	Cement	Stone	Iron sheet (corrugated	Yes	Yes
In a separa	Cement	Concrete blocks	Iron sheet (corrugated	Yes	Yes
In the corn	Cement	Stone	Iron sheet (corrugated	Yes	Yes
In a separa	Cement	Concrete blocks	Iron sheet (corrugated	No	No
In another	Cement	Concrete blocks	Iron sheet (corrugated	Yes	Yes
In the corn	Cement	Concrete blocks	Iron sheet (corrugated	No	Yes
Outside in	Cement	Stone	Iron sheet (corrugated	No	Yes
In a separa	Cement	Cemented mud	Iron sheet (corrugated	Yes	Yes
In the corn	Cement	Cemented mud	Iron sheet (corrugated	Yes	Yes
In the corn	Cement	Concrete blocks	Iron sheet (corrugated	No	Yes
In a separa	Natural floor	Concrete blocks	Iron sheet (corrugated	Yes	Yes
In the corn	Cement	Iron sheets(mabati)	Metal sheets/tin	Yes	Yes
Outside in	Cement	Concrete blocks	Iron sheet (corrugated	No	Yes
In the corn	Cement	Iron sheets(mabati)	Iron sheet (corrugated	Yes	Yes
In the corn	Natural floor	Mud	Iron sheet (corrugated	No window	Yes
In the corn	Natural floor	Mud	Other (Spe Grass/thatc	No window	Yes
In a separa	Cement	Stone	Iron sheet (corrugated	Yes	Yes
In another	Cement	Stone	Iron sheet (corrugated	Yes	Yes
In the corn	Cement	Stone	Iron sheet (corrugated	Yes	Yes
In a separa	Cement	Cemented mud	Iron sheet (corrugated	Yes	Yes
In the corn	Cement	Stone	Iron sheet (corrugated	Yes	Yes
In the corn	Cement	Concrete blocks	Iron sheet (corrugated	No	Yes
In a separa	Cement	Stone	Iron sheet (corrugated	Yes	Yes
Outside in	Natural floor	Mud/Stone Partly mud	Iron sheet (corrugated	Yes	Yes
In the corn	Cement	Iron sheets(mabati)	Iron sheet (corrugated	Yes	Yes
In the corn	Cement	Concrete blocks	Iron sheet (corrugated	Yes	Yes
In a separa	Cement	Concrete blocks	Iron sheet (corrugated	Yes	Yes
In a separa	Cement	Cemented mud	Iron sheet (corrugated	Yes	Yes

In the corn	Natural floor	Mud	Iron sheet (corrugated	Yes	Yes
In a separa	Polished wood	Concrete blocks	Iron sheet (corrugated	Yes	Yes
In the corn	Cement	Mud/Stone One side b	Iron sheet (corrugated	Yes	Yes
In the corn	Cement	Concrete blocks	Iron sheet (corrugated	Yes	Yes
In another	Natural floor	Stone	Iron sheet (corrugated	Yes	Yes
In the corn	Cement	Iron sheets(mabati)	Metal sheets/tin	Yes	Yes
Outside in	Cement	Concrete blocks	Iron sheet (corrugated	No	Yes
In the corn	Cement	Iron sheets(mabati)	Iron sheet (corrugated	No	Yes
In a separa	Cement	Cemented mud	Iron sheet (corrugated	Yes	Yes
Outside in	Cement	Concrete blocks	Iron sheet (corrugated	No	Yes
In the corn	Cement	Stone	Iron sheet (corrugated	Yes	Yes
In the corn	Natural floor	Stone	Iron sheet (corrugated	No	Yes
In a separa	Cement	Cemented mud	Iron sheet (corrugated	Yes	Yes
Outside in	Cement	Stone	Iron sheet (corrugated	Yes	Yes
In the corn	Cement	Stone	Iron sheet (corrugated	Yes	Yes
In the corn	Cement	Stone	Iron sheet (corrugated	Yes	Yes
In the corn	Natural floor	Concrete blocks	Iron sheet (corrugated	No	Yes
In the corn	Cement	Concrete blocks	Iron sheet (corrugated	Yes	Yes
In the corn	Cement	Concrete blocks	Iron sheet (corrugated	No	Yes
In the corn	Cement	Cemented mud	Iron sheet (corrugated	Yes	Yes
In the corn	Natural floor	Mud	Iron sheet (corrugated	Yes	Yes
In a separa	Cement	Mud	Iron sheet (corrugated	Yes	Yes
In the corn	Cement	Concrete blocks	Iron sheet (corrugated	No	Yes
In a separa	Polished wood	Cemented mud	Iron sheet (corrugated	Yes	Yes
In a separa	Cement	Cemented mud	Iron sheet (corrugated	Yes	Yes
Outside in	Cement	Concrete blocks	Iron sheet (corrugated	No	Yes
In a separa	Natural floor	Cemented mud	Iron sheet (corrugated	Yes	Yes
In a separa	Cement	Stone	Iron sheet (corrugated	Yes	Yes
In a separa	Cement	Concrete blocks	Iron sheet (corrugated	No	Yes
In the corn	Polished wood	Mud/Stone/Bricks mix	Iron sheet (corrugated	Yes	Yes
In a separa	Cement	Concrete blocks	Iron sheet (corrugated	Yes	Yes
In a separa	Natural floor	Concrete blocks	Iron sheet (corrugated	Yes	Yes
In a separa	Cement	Cemented mud	Iron sheet (corrugated	Yes	Yes
In the corn	Cement	Concrete blocks	Iron sheet (corrugated	No	Yes
In another	Cement	Stone	Iron sheet (corrugated	Yes	Yes
In the corn	Natural floor	Mud	Iron sheet (corrugated	No	Yes
In the corn	Cement	Iron sheets(mabati)	Iron sheet (corrugated	Yes	Yes
In the corn	Cement	Concrete blocks	Iron sheet (corrugated	Yes	Yes
In a separa	Cement	Concrete blocks	Iron sheet (corrugated	Yes	Yes
In a separa	Cement	Concrete blocks	Iron sheet (corrugated	No	Yes
In the corn	Cement	Cemented mud	Iron sheet (corrugated	Yes	Yes
Outside in	Natural floor	Mud	Iron sheet (corrugated	No window	Yes
In a separa	Polished wood	Concrete blocks	Iron sheet (corrugated	Yes	Yes
In another	Natural floor	Concrete blocks	Iron sheet (corrugated	Yes	Yes
In a separa	Natural floor	Concrete blocks	Iron sheet (corrugated	Yes	Yes
In the corn	Cement	Iron sheets(mabati)	Iron sheet (corrugated	Yes	Yes
In the corn	Cement	Concrete blocks	Iron sheet (corrugated	Yes	Yes
In a separa	Cement	Concrete blocks	Iron sheet (corrugated	Yes	Yes
In the corn	Cement	Cemented mud	Metal sheets/tin	Yes	Yes

In the corn	Cement	Concrete blocks	Iron sheet (corrugated	Yes	Yes
In a separa	Cement	Cemented mud	Iron sheet (corrugated	Yes	Yes
In the corn	Cement	Iron sheets(mabati)	Iron sheet (corrugated	Yes	Yes
In the corn	Natural floor	Mud/Stone Mud bricks	Iron sheet (corrugated	Yes	Yes
In a separa	Cement	Mud	Iron sheet (corrugated	Yes	Yes
In the corn	Polished wood	Mud	Iron sheet (corrugated	No window	Yes
In a separa	Natural floor	Cemented mud	Iron sheet (corrugated	Yes	Yes
In a separa	Natural floor	Stone	Iron sheet (corrugated	Yes	Yes
In the corn	Cement	Concrete blocks	Iron sheet (corrugated	No	Yes
In the corn	Cement	Cemented mud	Iron sheet (corrugated	No	Yes
In the corn	Cement	Iron sheets(mabati)	Iron sheet (corrugated	Yes	Yes
Outside in	Natural floor	Mud	Iron sheet (corrugated	No	Yes
In the corn	Cement	Cemented mud	Iron sheet (corrugated	No	Yes
In a separa	Cement	Concrete blocks	Iron sheet (corrugated	No	Yes
In the corn	Cement	Cemented mud	Iron sheet (corrugated	Yes	Yes
In the corn	Polished wood	Concrete blocks	Iron sheet (corrugated	Yes	Yes
In a separa	Cement	Cemented mud	Iron sheet (corrugated	Yes	Yes
In the corn	Cement	Concrete blocks	Iron sheet (corrugated	Yes	Yes
In the corn	Cement	Iron sheets(mabati)	Metal sheets/tin	Yes	Yes
In the corn	Cement	Concrete blocks	Iron sheet (corrugated	No	Yes
Outside in	Cement	Stone	Iron sheet (corrugated	Yes	Yes
In the corn	Natural floor	Mud	Iron sheet (corrugated	No window	Yes
In the corn	Cement	Concrete blocks	Iron sheet (corrugated	Yes	Yes
Outside in	Natural floor	Mud	Iron sheet (corrugated	No window	Yes
In a separa	Cement	Concrete blocks	Iron sheet (corrugated	Yes	Yes
In the corn	Cement	Concrete blocks	Iron sheet (corrugated	Yes	Yes
In a separa	Cement	Mud	Iron sheet (corrugated	Yes	Yes
Outside in	Natural floor	Mud	Iron sheet (corrugated	No window	Yes
In a separa	Cement	Cemented mud	Iron sheet (corrugated	Yes	Yes
In a separa	Cement	Cemented mud	Iron sheet (corrugated	Yes	Yes
In a separa	Polished wood	Concrete blocks	Iron sheet (corrugated	Yes	Yes
In a separa	Cement	Cemented mud	Iron sheet (corrugated	No	Yes
In the corn	Polished wood	Concrete blocks	Iron sheet (corrugated	Yes	Yes
In the corn	Cement	Iron sheets(mabati)	Iron sheet (corrugated	Yes	Yes
In another	Cement	Stone	Iron sheet (corrugated	Yes	Yes
In a separa	Cement	Stone	Iron sheet (corrugated	Yes	Yes
In another	Cement	Cemented mud	Iron sheet (corrugated	Yes	Yes
In the corn	Cement	Concrete blocks	Iron sheet (corrugated	Yes	Yes
In the corn	Polished wood	Cemented mud	Iron sheet (corrugated	Yes	Yes
In the corn	Cement	Iron sheets(mabati)	Iron sheet (corrugated	No	Yes
In a separa	Cement	Concrete blocks	Iron sheet (corrugated	Yes	Yes
In the corn	Cement	Concrete blocks	Iron sheet (corrugated	Yes	Yes
In a separa	Cement	Concrete blocks	Iron sheet (corrugated	Yes	Yes
In the corn	Cement	Stone	Iron sheet (corrugated	Yes	Yes
In the corn	Cement	Cemented mud	Iron sheet (corrugated	Yes	Yes
In the corn	Cement	Concrete blocks	Iron sheet (corrugated	Yes	Yes
In the corn	Natural floor	Mud	Iron sheet (corrugated	No window	Yes
In a separa	Cement	Stone	Iron sheet (corrugated	Yes	Yes
In a separa	Cement	Cemented mud	Iron sheet (corrugated	Yes	Yes

In the corn	Cement	Concrete blocks	Iron sheet (corrugated	Yes	Yes
In the corn	Polished wood	Concrete blocks	Iron sheet (corrugated	Yes	Yes
In the corn	Cement	Iron sheets(mabati)	Iron sheet (corrugated	No	Yes
In a separa	Polished wood	Concrete blocks	Iron sheet (corrugated	Yes	Yes
In a separa	Cement	Concrete blocks	Iron sheet (corrugated	Yes	Yes
In a separa	Cement	Cemented mud	Iron sheet (corrugated	Yes	Yes
In the corn	Polished wood	Concrete blocks	Iron sheet (corrugated	Yes	Yes
In a separa	Cement	Iron sheets(mabati)	Iron sheet (corrugated	Yes	Yes
In a separa	Polished wood	Concrete blocks	Iron sheet (corrugated	Yes	Yes
In the corn	Cement	Cemented mud	Iron sheet (corrugated	No	Yes
In the corn	Natural floor	Iron sheets(mabati)	Iron sheet (corrugated	No window	Yes
In the corn	Natural floor	Mud	Iron sheet (corrugated	Yes	Yes
In the corn	Cement	Concrete blocks	Iron sheet (corrugated	No	Yes
In the corn	Natural floor	Mud	Iron sheet (corrugated	No	Yes
In the corn	Cement	Mud	Iron sheet (corrugated	Yes	Yes
In the corn	Cement	Iron sheets(mabati)	Iron sheet (corrugated	Yes	Yes
In a separa	Cement	Cemented mud	Iron sheet (corrugated	Yes	Yes
In a separa	Cement	Stone	Iron sheet (corrugated	Yes	Yes
In a separa	Cement	Stone	Iron sheet (corrugated	Yes	Yes
In the corn	Polished wood	Mud	Iron sheet (corrugated	No window	Yes
In the corn	Cement	Stone	Iron sheet (corrugated	No	Yes
In the corn	Cement	Stone	Iron sheet (corrugated	Yes	Yes
In the corn	Cement	Iron sheets(mabati)	Iron sheet (corrugated	Yes	Yes
In the corn	Cement	Stone	Iron sheet (corrugated	No	Yes
In the corn	Cement	Concrete blocks	Iron sheet (corrugated	Yes	Yes
In the corn	Cement	Cemented mud	Iron sheet (corrugated	Yes	Yes
In a separa	Cement	Stone	Iron sheet (corrugated	Yes	Yes
In the corn	Cement	Stone	Iron sheet (corrugated	Yes	Yes
In the corn	Natural floor	Mud	Iron sheet (corrugated	No window	Yes
In the corn	Cement	Concrete blocks	Iron sheet (corrugated	Yes	Yes
In the corn	Cement	Stone	Iron sheet (corrugated	Yes	Yes
In the corn	Cement	Iron sheets(mabati)	Iron sheet (corrugated	Yes	Yes
In the corn	Cement	Iron sheets(mabati)	Iron sheet (corrugated	Yes	Yes
In a separa	Natural floor	Concrete blocks	Iron sheet (corrugated	No	Yes
In the corn	Cement	Cemented mud	Iron sheet (corrugated	No	Yes
In the corn	Cement	Cemented mud	Iron sheet (corrugated	Yes	Yes
In the corn	Cement	Iron sheets(mabati)	Iron sheet (corrugated	Yes	Yes
In the corn	Cement	Concrete blocks	Iron sheet (corrugated	Yes	Yes
In the corn	Cement	Iron sheets(mabati)	Iron sheet (corrugated	Yes	Yes
In a separa	Cement	Stone	Iron sheet (corrugated	Yes	Yes
In the corn	Natural floor	Mud	Metal sheets/tin	No	Yes
In a separa	Polished wood	Cemented mud	Iron sheet (corrugated	Yes	Yes
In a separa	Cement	Concrete blocks	Iron sheet (corrugated	No	Yes
In the corn	Cement	Iron sheets(mabati)	Iron sheet (corrugated	Yes	Yes
Outside in	Cement	Stone	Iron sheet (corrugated	Yes	Yes
In the corn	Cement	Iron sheets(mabati)	Iron sheet (corrugated	Yes	Yes
In the corn	Cement	Concrete blocks	Iron sheet (corrugated	Yes	Yes
In the corn	Cement	Iron sheets(mabati)	Iron sheet (corrugated	Yes	Yes
In the corn	Cement	Cemented mud	Iron sheet (corrugated	Yes	Yes

In a separa	Polished wood	Concrete blocks	Iron sheet (corrugated	Yes	Yes
In the corn	Cement	Cemented mud	Iron sheet (corrugated	Yes	Yes
In the corn	Cement	Iron sheets(mabati)	Iron sheet (corrugated	Yes	Yes
In a separa	Cement	Cemented mud	Iron sheet (corrugated	Yes	Yes
In a separa	Cement	Concrete blocks	Iron sheet (corrugated	Yes	Yes
In the corn	Cement	Cemented mud	Iron sheet (corrugated	Yes	Yes
In a separa	Cement	Stone	Iron sheet (corrugated	Yes	Yes
In a separa	Cement	Concrete blocks	Iron sheet (corrugated	Yes	Yes
In the corn	Cement	Concrete blocks	Iron sheet (corrugated	Yes	Yes
In the corn	Cement	Cemented mud	Iron sheet (corrugated	Yes	Yes
In the corn	Cement	Stone	Iron sheet (corrugated	Yes	Yes
In the corn	Cement	Cemented mud	Iron sheet (corrugated	Yes	Yes
In the corn	Cement	Stone	Iron sheet (corrugated	Yes	Yes
In the corn	Cement	Stone	Iron sheet (corrugated	Yes	Yes
In the corn	Cement	Iron sheets(mabati)	Iron sheet (corrugated	Yes	Yes
In the corn	Cement	Iron sheets(mabati)	Iron sheet (corrugated	Yes	Yes
In a separa	Cement	Stone	Metal sheets/tin	Yes	Yes
In a separa	Cement	Stone	Iron sheet (corrugated	Yes	Yes
In the corn	Cement	Iron sheets(mabati)	Iron sheet (corrugated	Yes	Yes
In a separa	Cement	Concrete blocks	Iron sheet (corrugated	No	Yes
In the corn	Cement	Stone	Metal sheets/tin	Yes	Yes
In a separa	Cement	Stone	Metal sheets/tin	Yes	Yes
In the corn	Cement	Stone	Iron sheet (corrugated	Yes	Yes
In the corn	Cement	Cemented mud	Iron sheet (corrugated	Yes	Yes
In a separa	Cement	Stone	Iron sheet (corrugated	Yes	Yes
In the corn	Cement	Iron sheets(mabati)	Iron sheet (corrugated	Yes	Yes
In the corn	Natural floor	Mud	Iron sheet (corrugated	No	Yes
In another	Cement	Concrete blocks	Iron sheet (corrugated	Yes	Yes
In the corn	Natural floor	Iron sheets(mabati)	Iron sheet (corrugated	Yes	Yes
In the corn	Cement	Stone	Iron sheet (corrugated	Yes	Yes
In a separa	Cement	Concrete blocks	Iron sheet (corrugated	Yes	Yes
In the corn	Polished wood	Iron sheets(mabati)	Iron sheet (corrugated	Yes	Yes
In the corn	Cement	Iron sheets(mabati)	Metal sheets/tin	Yes	Yes
In the corn	Cement	Concrete blocks	Iron sheet (corrugated	Yes	Yes
In the corn	Cement	Iron sheets(mabati)	Iron sheet (corrugated	Yes	Yes
In a separa	Cement	Concrete blocks	Iron sheet (corrugated	Yes	Yes
In the corn	Natural floor	Mud	Metal sheets/tin	Yes	Yes
In the corn	Cement	Stone	Iron sheet (corrugated	Yes	Yes
In the corn	Cement	Iron sheets(mabati)	Metal sheets/tin	Yes	Yes
In the corn	Cement	Iron sheets(mabati)	Iron sheet (corrugated	Yes	Yes
In the corn	Cement	Iron sheets(mabati)	Iron sheet (corrugated	Yes	Yes
In a separa	Cement	Cemented mud	Iron sheet (corrugated	Yes	Yes
In the corn	Cement	Iron sheets(mabati)	Iron sheet (corrugated	Yes	Yes
In the corn	Cement	Iron sheets(mabati)	Metal sheets/tin	Yes	Yes
In the corn	Cement	Iron sheets(mabati)	Iron sheet (corrugated	Yes	Yes
In the corn	Natural floor	Mud/Stone/Bricks mix	Iron sheet (corrugated	No	Yes
In the corn	Natural floor	Mud	Metal sheets/tin	Yes	Yes
In the corn	Cement	Stone	Iron sheet (corrugated	Yes	Yes
In the corn	Cement	Iron sheets(mabati)	Iron sheet (corrugated	Yes	Yes

In the corn	Polished wood	Iron sheets(mabati)	Iron sheet (corrugated	Yes	Yes
In a separa	Cement	Cemented mud	Iron sheet (corrugated	Yes	Yes
In the corn	Cement	Stone	Iron sheet (corrugated	Yes	Yes
In the corn	Cement	Stone	Iron sheet (corrugated	Yes	Yes
In the corn	Cement	Stone	Iron sheet (corrugated	Yes	Yes
In the corn	Cement	Iron sheets(mabati)	Iron sheet (corrugated	Yes	Yes
In the corn	Cement	Iron sheets(mabati)	Metal sheets/tin	No	Yes
In the corn	Cement	Iron sheets(mabati)	Iron sheet (corrugated	Yes	Yes
In the corn	Cement	Concrete blocks	Iron sheet (corrugated	Yes	Yes
In a separa	Natural floor	Mud	Iron sheet (corrugated	No	Yes
In the corn	Cement	Iron sheets(mabati)	Iron sheet (corrugated	Yes	Yes
In the corn	Cement	Concrete blocks	Iron sheet (corrugated	Yes	Yes
In the corn	Natural floor	Mud	Iron sheet (corrugated	No window	Yes
In the corn	Cement	Cemented mud	Iron sheet (corrugated	No	Yes
In the corn	Cement	Concrete blocks	Iron sheet (corrugated	Yes	Yes
In a separa	Cement	Concrete blocks	Iron sheet (corrugated	Yes	Yes
In a separa	Natural floor	Mud	Iron sheet (corrugated	Yes	Yes
In the corn	Cement	Mud/Stone/Bricks mix	Iron sheet (corrugated	Yes	Yes
In a separa	Cement	Stone	Iron sheet (corrugated	Yes	Yes
In the corn	Cement	Stone	Iron sheet (corrugated	Yes	Yes
In the corn	Cement	Iron sheets(mabati)	Iron sheet (corrugated	Yes	Yes
In a separa	Cement	Cemented mud	Iron sheet (corrugated	Yes	Yes
In a separa	Cement	Concrete blocks	Iron sheet (corrugated	No	Yes
In a separa	Polished wood	Stone	Metal sheets/tin	Yes	Yes
In a separa	Cement	Cemented mud	Iron sheet (corrugated	Yes	Yes
In a separa	Cement	Cemented mud	Iron sheet (corrugated	Yes	Yes
In another	Cement	Concrete blocks	Iron sheet (corrugated	Yes	Yes
In the corn	Cement	Cemented mud	Iron sheet (corrugated	Yes	Yes
In a separa	Cement	Cemented mud	Iron sheet (corrugated	Yes	Yes
In a separa	Cement	Concrete blocks	Iron sheet (corrugated	Yes	Yes
In the corn	Cement	Cemented mud	Metal sheets/tin	Yes	No
In the corn	Cement	Iron sheets(mabati)	Metal sheets/tin	Yes	Yes
In the corn	Cement	Cemented mud	Iron sheet (corrugated	Yes	Yes
In another	Polished wood	Cemented mud	Iron sheet (corrugated	Yes	Yes
In the corn	Cement	Iron sheets(mabati)	Iron sheet (corrugated	Yes	Yes
In the corn	Polished wood	Stone	Iron sheet (corrugated	Yes	Yes
In a separa	Natural floor	Mud	Iron sheet (corrugated	No	Yes
In a separa	Cement	Concrete blocks	Iron sheet (corrugated	Yes	Yes
In the corn	Natural floor	Iron sheets(mabati)	Iron sheet (corrugated	Yes	Yes
In the corn	Cement	Stone	Iron sheet (corrugated	Yes	Yes
In a separa	Polished wood	Stone	Metal sheets/tin	Yes	Yes
In the corn	Cement	Concrete blocks	Iron sheet (corrugated	Yes	Yes
Outside in	Cement	Stone	Iron sheet (corrugated	Yes	Yes
In a separa	Cement	Stone	Iron sheet (corrugated	Yes	Yes
In the corn	Cement	Stone	Iron sheet (corrugated	Yes	Yes
In the corn	Cement	Iron sheets(mabati)	Metal sheets/tin	Yes	Yes
In the corn	Cement	Concrete blocks	Iron sheet (corrugated	Yes	Yes
In a separa	Cement	Concrete blocks	Iron sheet (corrugated	Yes	Yes
In the corn	Cement	Concrete blocks	Metal sheets/tin	Yes	Yes

In the corn	Cement	Stone	Metal sheets/tin	Yes	Yes
In the corn	Cement	Mud	Iron sheet (corrugated	No	Yes
In the corn	Cement	Iron sheets(mabati)	Metal sheets/tin	Yes	Yes
In the corn	Cement	Mud	Iron sheet (corrugated	No	Yes
In a separa	Cement	Cemented mud	Iron sheet (corrugated	Yes	Yes
In a separa	Cement	Concrete blocks	Iron sheet (corrugated	No	Yes
In a separa	Cement	Mud/Stone Mud and c	Iron sheet (corrugated	Yes	Yes
In the corn	Cement	Stone	Iron sheet (corrugated	Yes	Yes
In the corn	Cement	Concrete blocks	Metal sheets/tin	Yes	Yes
In the corn	Cement	Iron sheets(mabati)	Iron sheet (corrugated	Yes	Yes
In the corn	Natural floor	Mud	Iron sheet (corrugated	Yes	Yes
In the corn	Cement	Cemented mud	Iron sheet (corrugated	No	Yes
In a separa	Polished wood	Iron sheets(mabati)	Iron sheet (corrugated	Yes	Yes
In the corn	Cement	Concrete blocks	Iron sheet (corrugated	No	Yes
In the corn	Cement	Mud	Iron sheet (corrugated	No	Yes
In a separa	Natural floor	Mud	Iron sheet (corrugated	No	Yes
In a separa	Polished wood	Concrete blocks	Iron sheet (corrugated	Yes	Yes
In the corn	Polished wood	Concrete blocks	Iron sheet (corrugated	Yes	Yes
In the corn	Cement	Concrete blocks	Iron sheet (corrugated	No	Yes
In the corn	Cement	Stone	Iron sheet (corrugated	Yes	Yes
In the corn	Cement	Concrete blocks	Metal sheets/tin	Yes	Yes
In a separa	Cement	Stone	Metal sheets/tin	Yes	Yes
In the corn	Cement	Stone	Metal sheets/tin	Yes	Yes
In a separa	Cement	Cemented mud	Iron sheet (corrugated	Yes	Yes
In the corn	Cement	Iron sheets(mabati)	Iron sheet (corrugated	Yes	Yes
In the corn	Cement	Cemented mud	Iron sheet (corrugated	No	Yes
In another	Cement	Concrete blocks	Iron sheet (corrugated	Yes	Yes
In the corn	Cement	Concrete blocks	Iron sheet (corrugated	Yes	Yes
In the corn	Cement	Iron sheets(mabati)	Iron sheet (corrugated	Yes	Yes
Outside in	Cement	Cemented mud	Iron sheet (corrugated	Yes	Yes
In a separa	Natural floor	Mud	Iron sheet (corrugated	No	Yes
In the corn	Polished wood	Iron sheets(mabati)	Iron sheet (corrugated	Yes	Yes
In the corn	Cement	Iron sheets(mabati)	Iron sheet (corrugated	Yes	Yes
In the corn	Cement	Iron sheets(mabati)	Iron sheet (corrugated	Yes	Yes
In the corn	Cement	Concrete blocks	Iron sheet (corrugated	No	Yes
In the corn	Cement	Iron sheets(mabati)	Iron sheet (corrugated	Yes	Yes
In a separa	Cement	Cemented mud	Iron sheet (corrugated	Yes	Yes
In the corn	Cement	Cemented mud	Iron sheet (corrugated	Yes	Yes
In the corn	Cement	Iron sheets(mabati)	Iron sheet (corrugated	Yes	Yes
In the corn	Natural floor	Iron sheets(mabati)	Iron sheet (corrugated	Yes	Yes
In the corn	Polished wood	Stone	Iron sheet (corrugated	Yes	Yes
In the corn	Cement	Iron sheets(mabati)	Iron sheet (corrugated	Yes	Yes
In the corn	Polished wood	Concrete blocks	Iron sheet (corrugated	Yes	Yes
In a separa	Cement	Iron sheets(mabati)	Iron sheet (corrugated	Yes	Yes
In the corn	Cement	Cemented mud	Iron sheet (corrugated	No	Yes
In a separa	Cement	Concrete blocks	Iron sheet (corrugated	No	Yes
In the corn	Cement	Iron sheets(mabati)	Metal sheets/tin	Yes	Yes
In another	Cement	Concrete blocks	Iron sheet (corrugated	Yes	Yes
In the corn	Cement	Concrete blocks	Iron sheet (corrugated	Yes	Yes

In the corn	Cement	Mud/Stone/Bricks mix	Iron sheet (corrugated	Yes	Yes
In a separa	Cement	Mud/Stone/Bricks mix	Iron sheet (corrugated	Yes	Yes
In the corn	Cement	Concrete blocks	Iron sheet (corrugated	Yes	Yes
In the corn	Cement	Stone	Metal sheets/tin	Yes	Yes
In a separa	Cement	Concrete blocks	Iron sheet (corrugated	Yes	Yes
In a separa	Cement	Cemented mud	Iron sheet (corrugated	Yes	No
In the corn	Cement	Concrete blocks	Iron sheet (corrugated	Yes	Yes
In the corn	Natural floor	Mud	Iron sheet (corrugated	No	Yes
In the corn	Cement	Concrete blocks	Iron sheet (corrugated	Yes	Yes
In a separa	Polished wood	Mud	Iron sheet (corrugated	Yes	Yes
In a separa	Cement	Stone	Iron sheet (corrugated	Yes	Yes
In the corn	Cement	Iron sheets(mabati)	Iron sheet (corrugated	No window	Yes
In the corn	Cement	Stone	Iron sheet (corrugated	Yes	Yes
In the corn	Cement	Iron sheets(mabati)	Iron sheet (corrugated	No	Yes
In a separa	Polished wood	Stone	Iron sheet (corrugated	Yes	Yes
In the corn	Cement	Iron sheets(mabati)	Iron sheet (corrugated	Yes	Yes
In the corn	Cement	Concrete blocks	Iron sheet (corrugated	Yes	Yes
In a separa	Cement	Iron sheets(mabati)	Metal sheets/tin	Yes	Yes
In the corn	Cement	Concrete blocks	Iron sheet (corrugated	No	Yes
In the corn	Cement	Iron sheets(mabati)	Iron sheet (corrugated	Yes	Yes
In the corn	Cement	Stone	Iron sheet (corrugated	Yes	Yes
In a separa	Natural floor	Stone	Iron sheet (corrugated	No window	Yes
In the corn	Cement	Iron sheets(mabati)	Metal sheets/tin	Yes	Yes
In a separa	Polished wood	Concrete blocks	Iron sheet (corrugated	Yes	Yes
In the corn	Cement	Iron sheets(mabati)	Iron sheet (corrugated	Yes	Yes
In the corn	Polished wood	Mud	Iron sheet (corrugated	Yes	Yes
In a separa	Cement	Stone	Iron sheet (corrugated	Yes	Yes
In a separa	Cement	Stone	Iron sheet (corrugated	Yes	Yes
In another	Natural floor	Cemented mud	Iron sheet (corrugated	Yes	Yes
In a separa	Polished wood	Concrete blocks	Iron sheet (corrugated	Yes	Yes
In the corn	Cement	Cemented mud	Iron sheet (corrugated	Yes	Yes
In a separa	Cement	Stone	Iron sheet (corrugated	Yes	Yes
In the corn	Cement	Iron sheets(mabati)	Iron sheet (corrugated	Yes	Yes
In a separa	Cement	Mud	Iron sheet (corrugated	Yes	Yes
In a separa	Polished wood	Concrete blocks	Iron sheet (corrugated	Yes	Yes
In the corn	Cement	Stone	Iron sheet (corrugated	Yes	Yes
In a separa	Cement	Concrete blocks	Iron sheet (corrugated	Yes	Yes
In the corn	Cement	Iron sheets(mabati)	Iron sheet (corrugated	Yes	Yes
In a separa	Cement	Stone	Iron sheet (corrugated	Yes	Yes
In a separa	Cement	Mud/Stone/Bricks mix	Metal sheets/tin	Yes	Yes
In the corn	Natural floor	Mud	Iron sheet (corrugated	No	Yes
In a separa	Cement	Concrete blocks	Iron sheet (corrugated	Yes	Yes
In the corn	Cement	Iron sheets(mabati)	Iron sheet (corrugated	Yes	Yes
In the corn	Cement	Stone	Iron sheet (corrugated	Yes	Yes
In a separa	Cement	Stone	Iron sheet (corrugated	Yes	Yes
In the corn	Cement	Concrete blocks	Iron sheet (corrugated	No	Yes
In a separa	Cement	Concrete blocks	Iron sheet (corrugated	Yes	Yes
In the corn	Cement	Stone	Iron sheet (corrugated	Yes	Yes
In the corn	Cement	Stone	Iron sheet (corrugated	Yes	Yes

In a separa	Polished wood	Concrete blocks	Iron sheet (corrugated	Yes	Yes
In the corn	Cement	Iron sheets(mabati)	Iron sheet (corrugated	Yes	Yes
In another	Natural floor	Mud	Iron sheet (corrugated	Yes	Yes
In a separa	Cement	Cemented mud	Iron sheet (corrugated	Yes	Yes
In the corn	Cement	Iron sheets(mabati)	Iron sheet (corrugated	Yes	Yes
In a separa	Cement	Stone	Iron sheet (corrugated	Yes	Yes
In the corn	Cement	Stone	Iron sheet (corrugated	Yes	Yes
In the corn	Cement	Stone	Metal sheets/tin	Yes	Yes
In the corn	Cement	Concrete blocks	Iron sheet (corrugated	Yes	Yes
In the corn	Cement	Concrete blocks	Other (Spe Slab	Yes	Yes
In a separa	Cement	Cemented mud	Metal sheets/tin	Yes	Yes
In the corn	Cement	Mud/Stone/Bricks mix	Iron sheet (corrugated	Yes	Yes
In a separa	Polished wood	Concrete blocks	Iron sheet (corrugated	Yes	Yes
In a separa	Cement	Iron sheets(mabati)	Iron sheet (corrugated	Yes	Yes
In the corn	Cement	Cemented mud	Iron sheet (corrugated	Yes	Yes
In the corn	Cement	Iron sheets(mabati)	Iron sheet (corrugated	Yes	Yes
In a separa	Cement	Stone	Iron sheet (corrugated	Yes	Yes
In a separa	Cement	Concrete blocks	Iron sheet (corrugated	Yes	Yes
In the corn	Cement	Concrete blocks	Iron sheet (corrugated	Yes	Yes
In a separa	Cement	Mud/Stone/Bricks mix	Iron sheet (corrugated	Yes	Yes
In the corn	Cement	Concrete blocks	Iron sheet (corrugated	Yes	Yes
In a separa	Cement	Concrete blocks	Iron sheet (corrugated	Yes	Yes
In the corn	Natural floor	Mud	Iron sheet (corrugated	Yes	Yes
In the corn	Cement	Stone	Iron sheet (corrugated	Yes	Yes
In a separa	Natural floor	Mud	Iron sheet (corrugated	No	Yes
In another	Natural floor	Mud	Iron sheet (corrugated	No window	No
In a separa	Natural floor	Cemented mud	Iron sheet (corrugated	Yes	Yes
In the corn	Cement	Mud/Stone/Bricks mix	Iron sheet (corrugated	Yes	Yes
In the corn	Cement	Stone	Iron sheet (corrugated	Yes	Yes
In another	Cement	Stone	Iron sheet (corrugated	No window	Yes
In the corn	Cement	Cemented mud	Iron sheet (corrugated	No	Yes
In a separa	Polished wood	Concrete blocks	Iron sheet (corrugated	Yes	Yes
In the corn	Cement	Cemented mud	Iron sheet (corrugated	Yes	Yes
In a separa	Cement	Stone	Iron sheet (corrugated	Yes	Yes
In the corn	Natural floor	Mud	Iron sheet (corrugated	No	Yes
In the corn	Polished wood	Stone	Iron sheet (corrugated	Yes	Yes
In the corn	Cement	Iron sheets(mabati)	Iron sheet (corrugated	Yes	Yes
In a separa	Cement	Cemented mud	Iron sheet (corrugated	Yes	Yes
In the corn	Cement	Iron sheets(mabati)	Iron sheet (corrugated	Yes	Yes
In a separa	Cement	Concrete blocks	Iron sheet (corrugated	No	Yes
In a separa	Cement	Stone	Metal sheets/tin	Yes	Yes
In a separa	Cement	Stone	Iron sheet (corrugated	Yes	Yes
In a separa	Polished wood	Concrete blocks	Iron sheet (corrugated	Yes	Yes
In the corn	Cement	Iron sheets(mabati)	Iron sheet (corrugated	Yes	Yes
In a separa	Natural floor	Mud	Iron sheet (corrugated	No	Yes
In a separa	Polished wood	Stone	Iron sheet (corrugated	Yes	Yes
In the corn	Cement	Iron sheets(mabati)	Iron sheet (corrugated	Yes	Yes
In the corn	Cement	Stone	Iron sheet (corrugated	Yes	Yes
In a separa	Cement	Stone	Iron sheet (corrugated	Yes	Yes

In a separa	Cement	Cemented mud	Iron sheet (corrugated	Yes	Yes
In a separa	Cement	Concrete blocks	Iron sheet (corrugated	Yes	Yes
In the corn	Cement	Iron sheets(mabati)	Iron sheet (corrugated	Yes	Yes
In the corn	Cement	Concrete blocks	Iron sheet (corrugated	Yes	Yes
In the corn	Natural floor	Iron sheets(mabati)	Iron sheet (corrugated	Yes	Yes
In a separa	Polished wood	Concrete blocks	Iron sheet (corrugated	Yes	Yes
In the corn	Cement	Stone	Iron sheet (corrugated	Yes	Yes
In the corn	Polished wood	Stone	Iron sheet (corrugated	Yes	Yes
In a separa	Cement	Cemented mud	Iron sheet (corrugated	Yes	Yes
In a separa	Cement	Concrete blocks	Iron sheet (corrugated	Yes	Yes
In the corn	Cement	Concrete blocks	Iron sheet (corrugated	Yes	Yes
In a separa	Cement	Iron sheets(mabati)	Iron sheet (corrugated	Yes	Yes
In the corn	Cement	Iron sheets(mabati)	Iron sheet (corrugated	Yes	Yes
In the corn	Cement	Iron sheets(mabati)	Iron sheet (corrugated	Yes	Yes
In the corn	Cement	Cemented mud	Iron sheet (corrugated	Yes	Yes
In a separa	Cement	Mud/Stone/Bricks mix	Iron sheet (corrugated	Yes	Yes
In a separa	Polished wood	Concrete blocks	Iron sheet (corrugated	Yes	Yes
In the corn	Cement	Mud	Iron sheet (corrugated	Yes	Yes
In the corn	Cement	Iron sheets(mabati)	Iron sheet (corrugated	No	Yes
In a separa	Cement	Stone	Metal sheets/tin	Yes	Yes
In a separa	Natural floor	Concrete blocks	Iron sheet (corrugated	No	Yes
In the corn	Polished wood	Concrete blocks	Iron sheet (corrugated	Yes	Yes
In the corn	Cement	Stone	Metal sheets/tin	Yes	Yes
In a separa	Cement	Cemented mud	Iron sheet (corrugated	Yes	Yes
In a separa	Polished wood	Concrete blocks	Metal sheets/tin	Yes	Yes
In a separa	Polished wood	Concrete blocks	Iron sheet (corrugated	Yes	Yes
In the corn	Cement	Cemented mud	Iron sheet (corrugated	Yes	Yes
In the corn	Cement	Mud/Stone/Bricks mix	Iron sheet (corrugated	Yes	Yes
In the corn	Cement	Iron sheets(mabati)	Iron sheet (corrugated	Yes	Yes
In the corn	Cement	Cemented mud	Iron sheet (corrugated	Yes	Yes
In a separa	Cement	Cemented mud	Iron sheet (corrugated	Yes	Yes
In the corn	Cement	Concrete blocks	Iron sheet (corrugated	Yes	Yes
In a separa	Natural floor	Mud	Iron sheet (corrugated	No	No
In the corn	Cement	Concrete blocks	Iron sheet (corrugated	Yes	Yes
In a separa	Cement	Stone	Metal sheets/tin	Yes	Yes
In the corn	Cement	Concrete blocks	Iron sheet (corrugated	Yes	Yes
In a separa	Cement	Concrete blocks	Iron sheet (corrugated	Yes	Yes
In the corn	Cement	Iron sheets(mabati)	Iron sheet (corrugated	Yes	Yes
In a separa	Cement	Concrete blocks	Iron sheet (corrugated	Yes	Yes
In the corn	Cement	Iron sheets(mabati)	Iron sheet (corrugated	No	Yes
In a separa	Polished wood	Concrete blocks	Iron sheet (corrugated	Yes	Yes
In another	Cement	Stone	Iron sheet (corrugated	Yes	Yes
In the corn	Cement	Concrete blocks	Iron sheet (corrugated	Yes	Yes
In a separa	Polished wood	Concrete blocks	Iron sheet (corrugated	Yes	Yes
In the corn	Cement	Concrete blocks	Iron sheet (corrugated	No	Yes
In the corn	Cement	Mud/Stone/Bricks mix	Iron sheet (corrugated	Yes	Yes
In the corn	Cement	Cemented mud	Iron sheet (corrugated	Yes	Yes
In a separa	Polished wood	Stone	Iron sheet (corrugated	Yes	Yes
In the corn	Cement	Stone	Iron sheet (corrugated	Yes	Yes

In the corn	Cement	Iron sheets(mabati)	Metal sheets/tin	Yes	Yes
In the corn	Cement	Cemented mud	Iron sheet (corrugated	Yes	Yes
In a separa	Cement	Concrete blocks	Iron sheet (corrugated	Yes	Yes
In the corn	Cement	Cemented mud	Iron sheet (corrugated	Yes	Yes
In a separa	Cement	Concrete blocks	Iron sheet (corrugated	Yes	Yes
In the corn	Cement	Cemented mud	Iron sheet (corrugated	Yes	Yes
In the corn	Cement	Concrete blocks	Iron sheet (corrugated	Yes	Yes
In a separa	Polished wood	Concrete blocks	Iron sheet (corrugated	Yes	Yes
In a separa	Cement	Cemented mud	Iron sheet (corrugated	No	Yes
In a separa	Cement	Stone	Iron sheet (corrugated	Yes	Yes
In the corn	Cement	Stone	Iron sheet (corrugated	Yes	Yes
In the corn	Polished wood	Mud/Stone/Bricks mix	Iron sheet (corrugated	No window	Yes
In a separa	Cement	Stone	Metal sheets/tin	Yes	Yes
In a separa	Cement	Concrete blocks	Iron sheet (corrugated	Yes	Yes
In the corn	Cement	Iron sheets(mabati)	Iron sheet (corrugated	Yes	Yes
In the corn	Cement	Cemented mud	Metal sheets/tin	Yes	Yes
In the corn	Cement	Cemented mud	Iron sheet (corrugated	Yes	Yes
In a separa	Cement	Cemented mud	Iron sheet (corrugated	Yes	Yes
In the corn	Cement	Iron sheets(mabati)	Iron sheet (corrugated	Yes	Yes
In a separa	Cement	Mud/Stone/Bricks mix	Iron sheet (corrugated	No	Yes
In a separa	Cement	Cemented mud	Metal sheets/tin	Yes	Yes
In a separa	Polished wood	Iron sheets(mabati)	Metal sheets/tin	Yes	Yes
In a separa	Cement	Concrete blocks	Iron sheet (corrugated	Yes	Yes
In the corn	Polished wood	Concrete blocks	Iron sheet (corrugated	Yes	Yes
In the corn	Cement	Stone	Iron sheet (corrugated	Yes	Yes
In the corn	Cement	Iron sheets(mabati)	Iron sheet (corrugated	Yes	Yes
In the corn	Cement	Concrete blocks	Iron sheet (corrugated	No	Yes
In the corn	Cement	Iron sheets(mabati)	Iron sheet (corrugated	Yes	Yes
In a separa	Cement	Cemented mud	Iron sheet (corrugated	Yes	Yes
In a separa	Cement	Cemented mud	Iron sheet (corrugated	Yes	Yes
In the corn	Cement	Iron sheets(mabati)	Metal sheets/tin	Yes	Yes
In the corn	Cement	Stone	Metal sheets/tin	Yes	Yes
In a separa	Cement	Stone	Iron sheet (corrugated	Yes	Yes
In the corn	Cement	Iron sheets(mabati)	Iron sheet (corrugated	Yes	Yes
In a separa	Cement	Iron sheets(mabati)	Iron sheet (corrugated	Yes	Yes
In the corn	Cement	Iron sheets(mabati)	Iron sheet (corrugated	Yes	Yes
In a separa	Cement	Concrete blocks	Iron sheet (corrugated	Yes	Yes
In the corn	Cement	Iron sheets(mabati)	Iron sheet (corrugated	Yes	Yes
In a separa	Cement	Concrete blocks	Iron sheet (corrugated	No	Yes
In the corn	Cement	Iron sheets(mabati)	Iron sheet (corrugated	Yes	Yes
In a separa	Polished wood	Stone	Metal sheets/tin	Yes	Yes
In a separa	Polished wood	Stone	Iron sheet (corrugated	Yes	Yes
In a separa	Cement	Cemented mud	Iron sheet (corrugated	No	Yes
In a separa	Cement	Concrete blocks	Iron sheet (corrugated	Yes	Yes
In the corn	Cement	Iron sheets(mabati)	Iron sheet (corrugated	Yes	Yes
Outside in	Natural floor	Stone	Iron sheet (corrugated	No window	Yes
In a separa	Cement	Stone	Metal sheets/tin	Yes	Yes
In the corn	Cement	Stone	Iron sheet (corrugated	Yes	Yes
In a separa	Cement	Cemented mud	Metal sheets/tin	Yes	Yes

In the corn	Cement	Iron sheets(mabati)	Iron sheet (corrugated		Yes	No
In the corn	Cement	Cemented mud	Iron sheet (corrugated		Yes	Yes
In the corn	Cement	Iron sheets(mabati)	Iron sheet (corrugated		Yes	No
In a separa	Cement	Concrete blocks	Iron sheet (corrugated		Yes	Yes
In a separa	Cement	Concrete blocks	Iron sheet (corrugated		Yes	Yes
In a separa	Natural floor	Mud	Iron sheet (corrugated		No	Yes
In a separa	Cement	Cemented mud	Iron sheet (corrugated		Yes	Yes
In the corn	Natural floor	Mud	Iron sheet (corrugated		No	Yes
In a separa	Cement	Stone	Metal sheets/tin		Yes	Yes
In the corn	Cement	Iron sheets(mabati)	Iron sheet (corrugated		Yes	Yes
In a separa	Cement	Stone	Other (Spe	Stone	Yes	Yes
In the corn	Natural floor	Mud	Iron sheet (corrugated		No	Yes
In a separa	Cement	Iron sheets(mabati)	Iron sheet (corrugated		Yes	Yes
In the corn	Cement	Iron sheets(mabati)	Iron sheet (corrugated		Yes	Yes
In the corn	Cement	Iron sheets(mabati)	Iron sheet (corrugated		No	Yes
In a separa	Cement	Concrete blocks	Iron sheet (corrugated		Yes	Yes
In the corn	Cement	Stone	Iron sheet (corrugated		Yes	Yes
In a separa	Cement	Stone	Iron sheet (corrugated		Yes	Yes
In the corn	Cement	Iron sheets(mabati)	Iron sheet (corrugated		Yes	Yes
In the corn	Cement	Mud/Stone/Bricks mix	Iron sheet (corrugated		Yes	Yes
In the corn	Cement	Stone	Iron sheet (corrugated		Yes	Yes
In the corn	Cement	Concrete blocks	Iron sheet (corrugated		Yes	Yes
In a separa	Polished wood	Mud/Stone/Bricks mix	Iron sheet (corrugated		Yes	Yes
In the corn	Cement	Iron sheets(mabati)	Iron sheet (corrugated		Yes	Yes
In a separa	Cement	Cemented mud	Metal sheets/tin		Yes	Yes
In a separa	Polished wood	Concrete blocks	Iron sheet (corrugated		Yes	Yes
In a separa	Cement	Mud/Stone/Bricks mix	Iron sheet (corrugated		Yes	Yes
In a separa	Cement	Cemented mud	Iron sheet (corrugated		Yes	Yes
In the corn	Cement	Iron sheets(mabati)	Iron sheet (corrugated		Yes	Yes
In the corn	Cement	Cemented mud	Iron sheet (corrugated		Yes	Yes
Outside in	Natural floor	Mud	Metal sheets/tin		Yes	Yes
In a separa	Cement	Cemented mud	Iron sheet (corrugated		Yes	Yes
In the corn	Cement	Iron sheets(mabati)	Iron sheet (corrugated		Yes	Yes
In the corn	Cement	Iron sheets(mabati)	Iron sheet (corrugated		Yes	Yes
In the corn	Cement	Stone	Iron sheet (corrugated		Yes	Yes
In a separa	Polished wood	Stone	Iron sheet (corrugated		Yes	Yes
In the corn	Cement	Iron sheets(mabati)	Iron sheet (corrugated		Yes	Yes
In a separa	Cement	Concrete blocks	Iron sheet (corrugated		Yes	Yes
In the corn	Cement	Iron sheets(mabati)	Metal sheets/tin		Yes	Yes
In the corn	Cement	Concrete blocks	Metal sheets/tin		Yes	Yes
In the corn	Cement	Stone	Iron sheet (corrugated		Yes	Yes
In the corn	Cement	Iron sheets(mabati)	Iron sheet (corrugated		No	Yes
In a separa	Cement	Cemented mud	Iron sheet (corrugated		Yes	Yes
In the corn	Cement	Stone	Iron sheet (corrugated		Yes	Yes
In a separa	Polished wood	Stone	Metal sheets/tin		Yes	Yes
In a separa	Cement	Stone	Iron sheet (corrugated		Yes	Yes
In a separa	Cement	Iron sheets(mabati)	Iron sheet (corrugated		Yes	Yes
In the corn	Cement	Stone	Iron sheet (corrugated		Yes	Yes
In the corn	Polished wood	Stone	Iron sheet (corrugated		Yes	Yes

In a separa	Natural floor	Mud	Iron sheet (corrugated	No	Yes
In the corn	Natural floor	Mud	Iron sheet (corrugated	No	Yes
In the corn	Cement	Iron sheets(mabati)	Iron sheet (corrugated	Yes	Yes
In the corn	Cement	Iron sheets(mabati)	Iron sheet (corrugated	Yes	Yes
In the corn	Cement	Cemented mud	Metal sheets/tin	Yes	Yes
In a separa	Cement	Iron sheets(mabati)	Iron sheet (corrugated	No	Yes
In the corn	Cement	Concrete blocks	Iron sheet (corrugated	Yes	Yes
In the corn	Cement	Iron sheets(mabati)	Iron sheet (corrugated	Yes	Yes
In a separa	Cement	Stone	Iron sheet (corrugated	Yes	Yes
In the corn	Cement	Stone	Iron sheet (corrugated	Yes	Yes
In a separa	Cement	Stone	Metal sheets/tin	Yes	Yes
In a separa	Cement	Stone	Iron sheet (corrugated	Yes	Yes
In the corn	Cement	Concrete blocks	Iron sheet (corrugated	Yes	Yes
In another	Cement	Concrete blocks	Iron sheet (corrugated	Yes	Yes
In the corn	Polished wood	Cemented mud	Iron sheet (corrugated	Yes	Yes
In the corn	Cement	Stone	Iron sheet (corrugated	Yes	Yes
In the corn	Cement	Iron sheets(mabati)	Iron sheet (corrugated	Yes	Yes
In the corn	Cement	Iron sheets(mabati)	Iron sheet (corrugated	Yes	Yes
Outside in	Natural floor	Mud	Iron sheet (corrugated	Yes	Yes
In the corn	Cement	Stone	Iron sheet (corrugated	Yes	Yes
In the corn	Cement	Cemented mud	Iron sheet (corrugated	Yes	Yes
In the corn	Cement	Iron sheets(mabati)	Iron sheet (corrugated	Yes	Yes
In another	Cement	Concrete blocks	Iron sheet (corrugated	Yes	Yes
In the corn	Cement	Iron sheets(mabati)	Iron sheet (corrugated	Yes	Yes
In the corn	Cement	Iron sheets(mabati)	Iron sheet (corrugated	Yes	Yes
In a separa	Cement	Cemented mud	Iron sheet (corrugated	Yes	Yes
In the corn	Cement	Concrete blocks	Iron sheet (corrugated	No	Yes
In the corn	Cement	Stone	Iron sheet (corrugated	Yes	Yes
In the corn	Polished wood	Concrete blocks	Iron sheet (corrugated	Yes	Yes
In the corn	Cement	Iron sheets(mabati)	Iron sheet (corrugated	Yes	Yes
In the corn	Cement	Iron sheets(mabati)	Iron sheet (corrugated	Yes	Yes
In a separa	Cement	Concrete blocks	Iron sheet (corrugated	Yes	Yes
In the corn	Cement	Iron sheets(mabati)	Iron sheet (corrugated	Yes	Yes
In a separa	Natural floor	Mud	Iron sheet (corrugated	Yes	Yes
In the corn	Cement	Cemented mud	Iron sheet (corrugated	Yes	Yes
In the corn	Cement	Iron sheets(mabati)	Iron sheet (corrugated	Yes	Yes
In a separa	Polished wood	Concrete blocks	Iron sheet (corrugated	Yes	Yes
In the corn	Cement	Concrete blocks	Iron sheet (corrugated	Yes	Yes
In a separa	Cement	Concrete blocks	Iron sheet (corrugated	Yes	Yes
In the corn	Cement	Concrete blocks	Iron sheet (corrugated	Yes	Yes
In a separa	Cement	Concrete blocks	Iron sheet (corrugated	Yes	Yes
In the corn	Cement	Iron sheets(mabati)	Iron sheet (corrugated	Yes	Yes
In the corn	Cement	Mud	Iron sheet (corrugated	Yes	Yes
In a separa	Cement	Cemented mud	Iron sheet (corrugated	Yes	Yes
In the corn	Cement	Iron sheets(mabati)	Metal sheets/tin	Yes	Yes
In the corn	Cement	Concrete blocks	Iron sheet (corrugated	Yes	Yes
Outside in	Natural floor	Mud	Iron sheet (corrugated	Yes	Yes
In the corn	Cement	Iron sheets(mabati)	Iron sheet (corrugated	No	Yes
In the corn	Cement	Concrete blocks	Iron sheet (corrugated	Yes	Yes

In the corn	Cement	Iron sheets(mabati)	Iron sheet (corrugated	Yes	Yes
In the corn	Cement	Iron sheets(mabati)	Iron sheet (corrugated	Yes	Yes
In the corn	Cement	Iron sheets(mabati)	Iron sheet (corrugated	Yes	Yes
In the corn	Cement	Iron sheets(mabati)	Metal sheets/tin	Yes	Yes
In the corn	Cement	Cemented mud	Iron sheet (corrugated	Yes	Yes
In the corn	Cement	Concrete blocks	Iron sheet (corrugated	No	Yes
In the corn	Cement	Iron sheets(mabati)	Iron sheet (corrugated	Yes	Yes
In a separa	Cement	Concrete blocks	Iron sheet (corrugated	Yes	Yes
In the corn	Cement	Cemented mud	Metal sheets/tin	Yes	Yes
In the corn	Cement	Iron sheets(mabati)	Iron sheet (corrugated	Yes	Yes
In the corn	Cement	Cemented mud	Iron sheet (corrugated	Yes	Yes
In the corn	Cement	Iron sheets(mabati)	Iron sheet (corrugated	Yes	Yes
In a separa	Cement	Stone	Iron sheet (corrugated	Yes	Yes
In a separa	Cement	Cemented mud	Metal sheets/tin	Yes	Yes
In the corn	Polished wood	Concrete blocks	Iron sheet (corrugated	Yes	Yes
In the corn	Cement	Stone	Iron sheet (corrugated	Yes	Yes
In a separa	Cement	Cemented mud	Iron sheet (corrugated	Yes	Yes
In the corn	Cement	Cemented mud	Iron sheet (corrugated	No	Yes
In the corn	Cement	Iron sheets(mabati)	Metal sheets/tin	Yes	Yes
In a separa	Cement	Concrete blocks	Iron sheet (corrugated	Yes	Yes
In the corn	Cement	Concrete blocks	Iron sheet (corrugated	Yes	Yes
In a separa	Cement	Stone	Iron sheet (corrugated	Yes	Yes
In a separa	Cement	Stone	Metal sheets/tin	Yes	Yes
In the corn	Cement	Cemented mud	Metal sheets/tin	Yes	Yes
In the corn	Cement	Iron sheets(mabati)	Iron sheet (corrugated	Yes	Yes
In the corn	Cement	Cemented mud	Iron sheet (corrugated	Yes	Yes
In a separa	Cement	Stone	Iron sheet (corrugated	Yes	Yes
In the corn	Cement	Stone	Iron sheet (corrugated	Yes	Yes
In the corn	Cement	Iron sheets(mabati)	Iron sheet (corrugated	Yes	Yes
In a separa	Cement	Concrete blocks	Iron sheet (corrugated	Yes	Yes
In the corn	Cement	Iron sheets(mabati)	Metal sheets/tin	Yes	Yes
In the corn	Cement	Concrete blocks	Iron sheet (corrugated	Yes	Yes
In another	Cement	Cemented mud	Iron sheet (corrugated	Yes	Yes
In the corn	Cement	Concrete blocks	Iron sheet (corrugated	No	Yes
In a separa	Cement	Concrete blocks	Iron sheet (corrugated	Yes	Yes
In the corn	Cement	Iron sheets(mabati)	Iron sheet (corrugated	No	Yes
Outside in	Natural floor	Mud	Iron sheet (corrugated	Yes	Yes
In the corn	Cement	Stone	Metal sheets/tin	Yes	Yes
In a separa	Cement	Cemented mud	Iron sheet (corrugated	No	Yes
In a separa	Cement	Cemented mud	Iron sheet (corrugated	Yes	Yes
In the corn	Cement	Iron sheets(mabati)	Iron sheet (corrugated	Yes	Yes
In the corn	Cement	Concrete blocks	Iron sheet (corrugated	Yes	Yes
In the corn	Cement	Stone	Iron sheet (corrugated	Yes	Yes
In the corn	Polished wood	Stone	Iron sheet (corrugated	Yes	Yes
In a separa	Cement	Iron sheets(mabati)	Metal sheets/tin	Yes	Yes
In the corn	Cement	Iron sheets(mabati)	Metal sheets/tin	Yes	Yes
In the corn	Cement	Concrete blocks	Iron sheet (corrugated	No	Yes
In the corn	Cement	Iron sheets(mabati)	Iron sheet (corrugated	Yes	Yes
In the corn	Cement	Iron sheets(mabati)	Iron sheet (corrugated	No	Yes

In the corn	Cement	Stone	Iron sheet (corrugated		Yes	Yes
In another	Natural floor	Mud	Iron sheet (corrugated		Yes	Yes
In the corn	Cement	Stone	Iron sheet (corrugated		Yes	Yes
In the corn	Cement	Cemented mud	Iron sheet (corrugated		Yes	Yes
In the corn	Cement	Iron sheets(mabati)	Iron sheet (corrugated		Yes	Yes
In a separa	Natural floor	Mud	Iron sheet (corrugated		No	Yes
In a separa	Cement	Cemented mud	Metal sheets/tin		Yes	Yes
In the corn	Cement	Iron sheets(mabati)	Metal sheets/tin		Yes	Yes
In the corn	Natural floor	Cemented mud	Iron sheet (corrugated		Yes	Yes
In the corn	Cement	Cemented mud	Iron sheet (corrugated		Yes	Yes
In the corn	Natural floor	Iron sheets(mabati)	Iron sheet (corrugated		No	Yes
In the corn	Cement	Concrete blocks	Iron sheet (corrugated		No	Yes
In a separa	Cement	Cemented mud	Iron sheet (corrugated		Yes	Yes
In the corn	Cement	Cemented mud	Iron sheet (corrugated		No	Yes
In the corn	Polished wood	Concrete blocks	Iron sheet (corrugated		Yes	Yes
In a separa	Cement	Mud	Iron sheet (corrugated		Yes	Yes
In the corn	Cement	Concrete blocks	Iron sheet (corrugated		No	Yes
In a separa	Polished wood	Stone	Metal sheets/tin		Yes	Yes
In the corn	Natural floor	Cemented mud	Iron sheet (corrugated		No window	Yes
In the corn	Cement	Cemented mud	Iron sheet (corrugated		Yes	Yes
In a separa	Cement	Concrete blocks	Iron sheet (corrugated		Yes	Yes
In the corn	Polished wood	Concrete blocks	Iron sheet (corrugated		Yes	Yes
In another	Natural floor	Mud	Iron sheet (corrugated		Yes	Yes
In a separa	Cement	Stone	Iron sheet (corrugated		Yes	Yes
In another	Cement	Concrete blocks	Iron sheet (corrugated		Yes	Yes
In the corn	Cement	Mud/Stone/Bricks mix	Iron sheet (corrugated		Yes	Yes
In the corn	Cement	Iron sheets(mabati)	Iron sheet (corrugated		Yes	Yes
In the corn	Cement	Cemented mud	Other (Spe	Tiles	Yes	Yes
In the corn	Cement	Concrete blocks	Iron sheet (corrugated		Yes	Yes
In the corn	Cement	Cemented mud	Iron sheet (corrugated		No	Yes
In the corn	Cement	Iron sheets(mabati)	Iron sheet (corrugated		Yes	Yes
In the corn	Cement	Cemented mud	Iron sheet (corrugated		Yes	Yes
In the corn	Cement	Iron sheets(mabati)	Iron sheet (corrugated		No window	Yes
In a separa	Cement	Stone	Metal sheets/tin		Yes	Yes
In the corn	Natural floor	Cemented mud	Iron sheet (corrugated		Yes	Yes
In a separa	Polished wood	Stone	Metal sheets/tin		Yes	Yes
In the corn	Cement	Stone	Iron sheet (corrugated		Yes	Yes
In the corn	Cement	Iron sheets(mabati)	Iron sheet (corrugated		Yes	Yes
In a separa	Natural floor	Concrete blocks	Iron sheet (corrugated		Yes	Yes
In a separa	Cement	Cemented mud	Iron sheet (corrugated		Yes	Yes
In the corn	Cement	Concrete blocks	Iron sheet (corrugated		Yes	Yes
In the corn	Cement	Cemented mud	Iron sheet (corrugated		No	Yes
In the corn	Cement	Concrete blocks	Iron sheet (corrugated		Yes	Yes
In the corn	Cement	Iron sheets(mabati)	Iron sheet (corrugated		Yes	Yes
In a separa	Cement	Concrete blocks	Iron sheet (corrugated		Yes	Yes
In a separa	Cement	Concrete blocks	Iron sheet (corrugated		Yes	Yes
In a separa	Cement	Stone	Other (Spe	Tiles	Yes	Yes
In a separa	Cement	Stone	Iron sheet (corrugated		Yes	Yes
In the corn	Cement	Iron sheets(mabati)	Iron sheet (corrugated		Yes	Yes

In the corn	Cement	Cemented mud	Iron sheet (corrugated	Yes	Yes
In the corn	Polished wood	Concrete blocks	Iron sheet (corrugated	Yes	Yes
In a separa	Cement	Concrete blocks	Iron sheet (corrugated	Yes	Yes
In the corn	Cement	Iron sheets(mabati)	Iron sheet (corrugated	Yes	Yes
In another	Natural floor	Mud	Iron sheet (corrugated	Yes	Yes
In another	Cement	Stone	Iron sheet (corrugated	Yes	Yes
In the corn	Cement	Iron sheets(mabati)	Iron sheet (corrugated	Yes	Yes
In the corn	Polished wood	Concrete blocks	Iron sheet (corrugated	Yes	Yes
In the corn	Cement	Concrete blocks	Iron sheet (corrugated	Yes	Yes
In a separa	Cement	Concrete blocks	Iron sheet (corrugated	Yes	Yes
In the corn	Cement	Concrete blocks	Iron sheet (corrugated	Yes	Yes
In a separa	Cement	Stone	Iron sheet (corrugated	Yes	Yes
In the corn	Cement	Iron sheets(mabati)	Metal sheets/tin	Yes	Yes
In the corn	Polished wood	Concrete blocks	Iron sheet (corrugated	No	Yes
In a separa	Cement	Stone	Iron sheet (corrugated	Yes	Yes
In a separa	Polished wood	Concrete blocks	Iron sheet (corrugated	Yes	Yes
In a separa	Cement	Cemented mud	Iron sheet (corrugated	Yes	Yes
In the corn	Cement	Iron sheets(mabati)	Iron sheet (corrugated	Yes	Yes
In the corn	Cement	Stone	Metal sheets/tin	Yes	Yes
In a separa	Cement	Cemented mud	Iron sheet (corrugated	Yes	Yes
In a separa	Polished wood	Concrete blocks	Iron sheet (corrugated	Yes	Yes
In the corn	Cement	Iron sheets(mabati)	Iron sheet (corrugated	Yes	Yes
In the corn	Cement	Stone	Iron sheet (corrugated	Yes	Yes
In the corn	Cement	Concrete blocks	Iron sheet (corrugated	Yes	Yes
In the corn	Natural floor	Mud	Iron sheet (corrugated	No	Yes
In a separa	Cement	Cemented mud	Iron sheet (corrugated	No	Yes
In a separa	Cement	Stone	Metal sheets/tin	Yes	Yes
In the corn	Cement	Stone	Iron sheet (corrugated	Yes	Yes
In a separa	Cement	Stone	Iron sheet (corrugated	Yes	Yes
In a separa	Cement	Stone	Iron sheet (corrugated	Yes	Yes
In the corn	Cement	Iron sheets(mabati)	Metal sheets/tin	Yes	Yes
In a separa	Cement	Concrete blocks	Iron sheet (corrugated	Yes	Yes
In the corn	Cement	Concrete blocks	Iron sheet (corrugated	Yes	Yes
In a separa	Cement	Concrete blocks	Iron sheet (corrugated	Yes	Yes
In the corn	Natural floor	Mud	Iron sheet (corrugated	Yes	Yes
In the corn	Cement	Iron sheets(mabati)	Iron sheet (corrugated	Yes	Yes
Outside in	Natural floor	Mud	Iron sheet (corrugated	Yes	Yes
In the corn	Cement	Concrete blocks	Iron sheet (corrugated	Yes	Yes
In the corn	Cement	Iron sheets(mabati)	Iron sheet (corrugated	Yes	Yes
In a separa	Polished wood	Stone	Iron sheet (corrugated	Yes	Yes
In a separa	Cement	Concrete blocks	Iron sheet (corrugated	No	Yes
In a separa	Cement	Cemented Cement	Iron sheet (corrugated	Yes	Yes
In a separa	Polished wood	Concrete blocks	Iron sheet (corrugated	Yes	Yes
In a separa	Natural floor	Mud	Iron sheet (corrugated	No	Yes
In a separa	Polished wood	Concrete blocks	Iron sheet (corrugated	Yes	Yes
In the corn	Natural floor	Mud	Iron sheet (corrugated	No	Yes
In a separa	Polished wood	Concrete blocks	Iron sheet (corrugated	Yes	Yes
In the corn	Cement	Cemented mud	Iron sheet (corrugated	Yes	Yes
In the corn	Cement	Iron sheets(mabati)	Metal sheets/tin	No	Yes

In the corn	Cement	Iron sheets(mabati)	Iron sheet (corrugated	Yes	Yes
In a separa	Polished wood	Stone	Iron sheet (corrugated	Yes	Yes
In a separa	Polished wood	Cemented mud	Iron sheet (corrugated	Yes	Yes
In a separa	Cement	Stone	Iron sheet (corrugated	Yes	Yes
In the corn	Natural floor	Stone	Iron sheet (corrugated	No	Yes
In the corn	Cement	Concrete blocks	Iron sheet (corrugated	Yes	Yes
In the corn	Cement	Cemented mud	Iron sheet (corrugated	Yes	Yes
In the corn	Natural floor	Stone	Iron sheet (corrugated	No window	Yes
In the corn	Cement	Concrete blocks	Iron sheet (corrugated	Yes	Yes
Outside in	Cement	Mud/Stone/Bricks mix	Iron sheet (corrugated	Yes	Yes
In a separa	Cement	Iron sheets(mabati)	Iron sheet (corrugated	Yes	Yes
In the corn	Cement	Stone	Iron sheet (corrugated	Yes	Yes
In a separa	Polished wood	Concrete blocks	Iron sheet (corrugated	Yes	Yes
In the corn	Cement	Iron sheets(mabati)	Iron sheet (corrugated	Yes	Yes
In a separa	Cement	Mud	Metal sheets/tin	Yes	Yes
In the corn	Cement	Cemented mud	Iron sheet (corrugated	No	Yes
In a separa	Natural floor	Concrete blocks	Iron sheet (corrugated	Yes	Yes
In the corn	Cement	Iron sheets(mabati)	Iron sheet (corrugated	No	No
In the corn	Cement	Stone	Iron sheet (corrugated	Yes	Yes
In the corn	Cement	Iron sheets(mabati)	Iron sheet (corrugated	Yes	Yes
In a separa	Cement	Iron sheets(mabati)	Iron sheet (corrugated	Yes	Yes
In a separa	Cement	Cemented mud	Iron sheet (corrugated	Yes	Yes
In a separa	Cement	Stone	Metal sheets/tin	Yes	Yes
In a separa	Natural floor	Mud	Iron sheet (corrugated	Yes	Yes
In the corn	Cement	Iron sheets(mabati)	Iron sheet (corrugated	Yes	Yes
In a separa	Cement	Cemented mud	Iron sheet (corrugated	Yes	Yes
In the corn	Cement	Iron sheets(mabati)	Metal sheets/tin	Yes	Yes
In a separa	Cement	Stone	Metal sheets/tin	Yes	Yes
In a separa	Cement	Concrete blocks	Iron sheet (corrugated	No	Yes
In the corn	Cement	Mud/Stone/Bricks mix	Metal sheets/tin	Yes	Yes
In a separa	Cement	Concrete blocks	Iron sheet (corrugated	Yes	Yes
In the corn	Cement	Iron sheets(mabati)	Iron sheet (corrugated	Yes	Yes
In the corn	Cement	Stone	Iron sheet (corrugated	Yes	Yes
In the corn	Cement	Cemented mud	Iron sheet (corrugated	Yes	Yes
In the corn	Cement	Iron sheets(mabati)	Metal sheets/tin	Yes	Yes
In the corn	Cement	Cemented mud	Iron sheet (corrugated	No	Yes
In a separa	Cement	Concrete blocks	Iron sheet (corrugated	No	Yes
In the corn	Cement	Stone	Iron sheet (corrugated	Yes	Yes
In the corn	Natural floor	Mud	Iron sheet (corrugated	No window	Yes
In the corn	Cement	Stone	Iron sheet (corrugated	Yes	Yes
In the corn	Cement	Stone	Iron sheet (corrugated	Yes	Yes
In another	Natural floor	Stone	Iron sheet (corrugated	Yes	Yes
In the corn	Natural floor	Mud	Iron sheet (corrugated	No	Yes
In the corn	Cement	Iron sheets(mabati)	Iron sheet (corrugated	Yes	Yes
In the corn	Cement	Iron sheets(mabati)	Metal sheets/tin	Yes	Yes
In a separa	Polished wood	Stone	Iron sheet (corrugated	Yes	Yes
In a separa	Cement	Concrete blocks	Iron sheet (corrugated	Yes	Yes
In the corn	Cement	Stone	Metal sheets/tin	Yes	Yes
In the corn	Cement	Iron sheets(mabati)	Iron sheet (corrugated	Yes	Yes

In a separa	Cement	Cemented mud	Metal sheets/tin	Yes	Yes
In the corn	Cement	Iron sheets(mabati)	Iron sheet (corrugated	Yes	Yes
In a separa	Natural floor	Mud/Stone Stones anc	Iron sheet (corrugated	Yes	Yes
In the corn	Cement	Stone	Metal sheets/tin	Yes	Yes
In the corn	Cement	Iron sheets(mabati)	Iron sheet (corrugated	Yes	Yes
In the corn	Cement	Iron sheets(mabati)	Iron sheet (corrugated	Yes	Yes
In a separa	Cement	Concrete blocks	Iron sheet (corrugated	Yes	Yes
In the corn	Cement	Iron sheets(mabati)	Iron sheet (corrugated	Yes	Yes
In a separa	Cement	Stone	Iron sheet (corrugated	Yes	Yes
In the corn	Cement	Iron sheets(mabati)	Metal sheets/tin	Yes	Yes
In the corn	Cement	Stone	Iron sheet (corrugated	No	Yes
In the corn	Cement	Cemented mud	Iron sheet (corrugated	No	Yes
In the corn	Cement	Iron sheets(mabati)	Iron sheet (corrugated	Yes	Yes
In a separa	Cement	Stone	Iron sheet (corrugated	Yes	Yes
In the corn	Cement	Cemented mud	Iron sheet (corrugated	Yes	Yes
In the corn	Cement	Cemented mud	Iron sheet (corrugated	Yes	Yes
In a separa	Cement	Concrete blocks	Iron sheet (corrugated	Yes	Yes
In a separa	Cement	Concrete blocks	Iron sheet (corrugated	Yes	Yes
In the corn	Natural floor	Mud/Stone Quarry sto	Iron sheet (corrugated	No window	Yes
In a separa	Cement	Stone	Metal sheets/tin	Yes	Yes
In the corn	Cement	Iron sheets(mabati)	Iron sheet (corrugated	Yes	Yes
Outside in	Natural floor	Mud	Iron sheet (corrugated	Yes	Yes
In the corn	Cement	Mud	Iron sheet (corrugated	No	Yes
In a separa	Polished wood	Concrete blocks	Iron sheet (corrugated	Yes	Yes
In another	Cement	Concrete blocks	Iron sheet (corrugated	Yes	Yes
In the corn	Cement	Mud	Iron sheet (corrugated	No	Yes
In the corn	Cement	Stone	Iron sheet (corrugated	Yes	Yes
In the corn	Cement	Stone	Iron sheet (corrugated	Yes	Yes
In the corn	Cement	Iron sheets(mabati)	Metal sheets/tin	Yes	Yes
In a separa	Cement	Concrete blocks	Iron sheet (corrugated	Yes	Yes
In a separa	Cement	Stone	Iron sheet (corrugated	Yes	Yes
In the corn	Cement	Iron sheets(mabati)	Iron sheet (corrugated	Yes	Yes
In the corn	Cement	Iron sheets(mabati)	Iron sheet (corrugated	Yes	Yes
In the corn	Cement	Concrete blocks	Iron sheet (corrugated	Yes	Yes
In the corn	Cement	Cemented mud	Iron sheet (corrugated	No	Yes
In the corn	Cement	Iron sheets(mabati)	Metal sheets/tin	Yes	Yes
In a separa	Cement	Concrete blocks	Iron sheet (corrugated	Yes	Yes
In the corn	Cement	Cemented mud	Iron sheet (corrugated	Yes	Yes
In the corn	Cement	Iron sheets(mabati)	Iron sheet (corrugated	Yes	Yes
In the corn	Cement	Cemented mud	Iron sheet (corrugated	Yes	Yes
In the corn	Cement	Stone	Iron sheet (corrugated	Yes	Yes
In a separa	Cement	Cemented mud	Iron sheet (corrugated	Yes	Yes
In a separa	Cement	Concrete blocks	Iron sheet (corrugated	Yes	Yes
In the corn	Cement	Iron sheets(mabati)	Iron sheet (corrugated	No	Yes
In the corn	Polished wood	Iron sheets(mabati)	Iron sheet (corrugated	No window	Yes
In a separa	Cement	Concrete blocks	Iron sheet (corrugated	Yes	Yes
In the corn	Cement	Cemented mud	Iron sheet (corrugated	No	Yes
In the corn	Cement	Iron sheets(mabati)	Iron sheet (corrugated	Yes	Yes
In the corn	Polished wood	Concrete blocks	Iron sheet (corrugated	Yes	Yes

In the corn	Cement	Mud	Iron sheet (corrugated	Yes	Yes
In the corn	Cement	Cemented mud	Iron sheet (corrugated	No	Yes
In the corn	Cement	Stone	Iron sheet (corrugated	Yes	Yes
In a separa	Cement	Mud	Iron sheet (corrugated	No window	Yes
In the corn	Cement	Iron sheets(mabati)	Iron sheet (corrugated	Yes	Yes
In the corn	Natural floor	Mud	Iron sheet (corrugated	No	Yes
In the corn	Cement	Iron sheets(mabati)	Iron sheet (corrugated	Yes	Yes
In a separa	Cement	Mud/Stone/Bricks mix	Iron sheet (corrugated	Yes	Yes
In the corn	Cement	Iron sheets(mabati)	Iron sheet (corrugated	Yes	Yes
In the corn	Cement	Iron sheets(mabati)	Iron sheet (corrugated	Yes	Yes
In the corn	Natural floor	Concrete blocks	Iron sheet (corrugated	Yes	Yes
In a separa	Cement	Mud/Stone/Bricks mix	Iron sheet (corrugated	Yes	Yes
In the corn	Cement	Iron sheets(mabati)	Iron sheet (corrugated	Yes	Yes
In a separa	Cement	Stone	Iron sheet (corrugated	Yes	Yes
In the corn	Cement	Cemented mud	Iron sheet (corrugated	Yes	Yes
In the corn	Cement	Cemented mud	Iron sheet (corrugated	No	Yes
Outside in	Cement	Mud/Stone/Bricks mix	Iron sheet (corrugated	No	Yes
In the corn	Cement	Iron sheets(mabati)	Metal sheets/tin	Yes	Yes
In the corn	Cement	Iron sheets(mabati)	Iron sheet (corrugated	Yes	Yes
In the corn	Cement	Concrete blocks	Iron sheet (corrugated	Yes	Yes
In a separa	Natural floor	Mud	Iron sheet (corrugated	Yes	Yes
In the corn	Polished wood	Stone	Iron sheet (corrugated	Yes	Yes
In the corn	Cement	Concrete blocks	Other (Spe Tiles	Yes	Yes
In the corn	Cement	Iron sheets(mabati)	Other (Spe Tiles	Yes	Yes
In a separa	Polished wood	Iron sheets(mabati)	Iron sheet (corrugated	Yes	Yes
In another	Natural floor	Mud	Iron sheet (corrugated	Yes	Yes
In the corn	Cement	Iron sheets(mabati)	Iron sheet (corrugated	Yes	Yes
In a separa	Cement	Stone	Iron sheet (corrugated	Yes	Yes
In the corn	Cement	Cemented mud	Iron sheet (corrugated	Yes	Yes
In the corn	Cement	Cemented mud	Iron sheet (corrugated	Yes	Yes
In the corn	Cement	Concrete blocks	Iron sheet (corrugated	Yes	Yes
In a separa	Cement	Stone	Metal sheets/tin	Yes	Yes
In the corn	Cement	Stone	Metal sheets/tin	Yes	Yes
In the corn	Cement	Iron sheets(mabati)	Iron sheet (corrugated	Yes	Yes
In a separa	Cement	Cemented mud	Metal sheets/tin	Yes	Yes
In the corn	Cement	Mud	Iron sheet (corrugated	Yes	Yes
In the corn	Cement	Stone	Metal sheets/tin	Yes	Yes
In the corn	Cement	Iron sheets(mabati)	Iron sheet (corrugated	Yes	Yes
In the corn	Cement	Cemented mud	Iron sheet (corrugated	Yes	Yes
In another	Natural floor	Mud	Iron sheet (corrugated	Yes	Yes
In the corn	Cement	Stone	Iron sheet (corrugated	Yes	Yes
In the corn	Cement	Stone	Iron sheet (corrugated	Yes	Yes
In the corn	Cement	Concrete blocks	Iron sheet (corrugated	Yes	Yes
In the corn	Cement	Mud/Stone/Bricks mix	Iron sheet (corrugated	Yes	Yes
In the corn	Cement	Cemented mud	Iron sheet (corrugated	Yes	Yes
In a separa	Cement	Iron sheets(mabati)	Iron sheet (corrugated	No window	Yes
In a separa	Polished wood	Stone	Iron sheet (corrugated	Yes	Yes
In the corn	Cement	Concrete blocks	Iron sheet (corrugated	Yes	Yes
In a separa	Cement	Stone	Iron sheet (corrugated	Yes	Yes

In a separa	Cement	Stone	Iron sheet (corrugated	Yes	Yes
In the corn	Cement	Iron sheets(mabati)	Iron sheet (corrugated	Yes	Yes
In the corn	Cement	Stone	Iron sheet (corrugated	Yes	Yes
In a separa	Cement	Cemented mud	Iron sheet (corrugated	Yes	Yes
In a separa	Cement	Stone	Metal sheets/tin	Yes	Yes
In the corn	Cement	Stone	Iron sheet (corrugated	Yes	Yes
In another	Cement	Stone	Iron sheet (corrugated	Yes	Yes
In the corn	Polished wood	Stone	Iron sheet (corrugated	Yes	Yes
In a separa	Cement	Cemented mud	Iron sheet (corrugated	Yes	Yes
In the corn	Cement	Mud	Iron sheet (corrugated	Yes	Yes
In a separa	Cement	Cemented mud	Iron sheet (corrugated	Yes	Yes
In the corn	Cement	Iron sheets(mabati)	Other (Spe Tiles	No window	Yes
In the corn	Cement	Stone	Iron sheet (corrugated	Yes	Yes
In a separa	Polished wood	Concrete blocks	Iron sheet (corrugated	Yes	Yes
In the corn	Cement	Iron sheets(mabati)	Metal sheets/tin	Yes	Yes
In a separa	Cement	Concrete blocks	Iron sheet (corrugated	Yes	Yes
In the corn	Cement	Iron sheets(mabati)	Iron sheet (corrugated	Yes	Yes
In a separa	Polished wood	Concrete blocks	Iron sheet (corrugated	Yes	Yes
In the corn	Polished wood	Stone	Iron sheet (corrugated	Yes	Yes
In a separa	Cement	Concrete blocks	Iron sheet (corrugated	Yes	Yes
In the corn	Cement	Concrete blocks	Metal sheets/tin	Yes	Yes
In the corn	Cement	Stone	Iron sheet (corrugated	Yes	Yes
In the corn	Polished wood	Concrete blocks	Iron sheet (corrugated	Yes	Yes
In a separa	Cement	Concrete blocks	Iron sheet (corrugated	Yes	Yes
In a separa	Cement	Stone	Metal sheets/tin	Yes	Yes
In the corn	Polished wood	Stone	Iron sheet (corrugated	Yes	Yes
In the corn	Cement	Concrete blocks	Iron sheet (corrugated	Yes	Yes
In the corn	Cement	Iron sheets(mabati)	Iron sheet (corrugated	Yes	Yes
In the corn	Cement	Iron sheets(mabati)	Iron sheet (corrugated	Yes	Yes
In the corn	Cement	Concrete blocks	Iron sheet (corrugated	Yes	Yes
In a separa	Cement	Cemented mud	Iron sheet (corrugated	No	Yes
In the corn	Cement	Iron sheets(mabati)	Iron sheet (corrugated	Yes	Yes
In the corn	Cement	Concrete blocks	Iron sheet (corrugated	Yes	Yes
In a separa	Cement	Concrete blocks	Iron sheet (corrugated	Yes	Yes
In the corn	Natural floor	Mud	Iron sheet (corrugated	No window	Yes
In a separa	Natural floor	Concrete blocks	Iron sheet (corrugated	Yes	Yes
In the corn	Cement	Iron sheets(mabati)	Iron sheet (corrugated	Yes	Yes
In the corn	Cement	Concrete blocks	Iron sheet (corrugated	Yes	Yes
In the corn	Natural floor	Concrete blocks	Iron sheet (corrugated	No	Yes
In a separa	Cement	Cemented mud	Iron sheet (corrugated	Yes	Yes
In the corn	Cement	Iron sheets(mabati)	Metal sheets/tin	No	Yes
In the corn	Cement	Iron sheets(mabati)	Iron sheet (corrugated	Yes	Yes
In a separa	Cement	Concrete blocks	Iron sheet (corrugated	Yes	Yes
In the corn	Cement	Cemented mud	Metal sheets/tin	Yes	Yes
In the corn	Polished wood	Stone	Iron sheet (corrugated	Yes	Yes
In a separa	Polished wood	Concrete blocks	Iron sheet (corrugated	Yes	Yes
In the corn	Cement	Iron sheets(mabati)	Iron sheet (corrugated	Yes	Yes
In the corn	Cement	Stone	Iron sheet (corrugated	Yes	Yes
In a separa	Cement	Concrete blocks	Iron sheet (corrugated	Yes	Yes

In the corn	Cement	Concrete blocks	Iron sheet (corrugated		No	Yes
In the corn	Cement	Iron sheets(mabati)	Iron sheet (corrugated		Yes	Yes
In a separa	Cement	Stone	Metal sheets/tin		Yes	Yes
In the corn	Cement	Iron sheets(mabati)	Metal sheets/tin		Yes	Yes
In a separa	Cement	Stone	Iron sheet (corrugated		Yes	Yes
In a separa	Cement	Concrete blocks	Iron sheet (corrugated		Yes	Yes
In a separa	Natural floor	Mud	Iron sheet (corrugated		Yes	Yes
In another	Cement	Concrete blocks	Iron sheet (corrugated		Yes	Yes
In the corn	Cement	Iron sheets(mabati)	Iron sheet (corrugated		Yes	Yes
In the corn	Cement	Iron sheets(mabati)	Iron sheet (corrugated		Yes	Yes
In the corn	Cement	Concrete blocks	Iron sheet (corrugated		Yes	Yes
In the corn	Cement	Concrete blocks	Iron sheet (corrugated		Yes	Yes
Outside in	Cement	Stone	Iron sheet (corrugated		Yes	Yes
In the corn	Cement	Iron sheets(mabati)	Iron sheet (corrugated		Yes	Yes
Outside in	Cement	Mud	Iron sheet (corrugated		Yes	Yes
In the corn	Cement	Stone	Metal sheets/tin		Yes	Yes
In a separa	Polished wood	Cemented mud	Iron sheet (corrugated		Yes	Yes
In a separa	Cement	Stone	Metal sheets/tin		Yes	Yes
In the corn	Cement	Iron sheets(mabati)	Iron sheet (corrugated		Yes	Yes
In the corn	Natural floor	Mud	Iron sheet (corrugated		Yes	Yes
In a separa	Cement	Stone	Iron sheet (corrugated		Yes	Yes
In the corn	Cement	Iron sheets(mabati)	Metal sheets/tin		Yes	Yes
In the corn	Cement	Stone	Metal sheets/tin		Yes	Yes
In a separa	Natural floor	Mud	Other (Spe	Grass/thatc	Yes	Yes
In a separa	Cement	Cemented mud	Iron sheet (corrugated		Yes	Yes
In a separa	Cement	Stone	Metal sheets/tin		Yes	Yes
In the corn	Polished wood	Concrete blocks	Iron sheet (corrugated		Yes	Yes
In a separa	Cement	Cemented mud	Iron sheet (corrugated		Yes	Yes
In the corn	Cement	Cemented mud	Iron sheet (corrugated		Yes	Yes
In the corn	Cement	Iron sheets(mabati)	Iron sheet (corrugated		Yes	Yes
In a separa	Cement	Cemented mud	Iron sheet (corrugated		No	Yes
In a separa	Cement	Iron sheets(mabati)	Iron sheet (corrugated		Yes	Yes
In the corn	Cement	Iron sheets(mabati)	Iron sheet (corrugated		Yes	Yes
In the corn	Cement	Iron sheets(mabati)	Iron sheet (corrugated		Yes	Yes
In a separa	Natural floor	Mud	Iron sheet (corrugated		No	Yes
In the corn	Cement	Stone	Iron sheet (corrugated		Yes	Yes
In the corn	Polished wood	Iron sheets(mabati)	Iron sheet (corrugated		No	Yes
In a separa	Natural floor	Stone	Iron sheet (corrugated		Yes	Yes
In another	Natural floor	Mud	Iron sheet (corrugated		No	No
In the corn	Polished wood	Stone	Iron sheet (corrugated		Yes	Yes
In another	Cement	Concrete blocks	Iron sheet (corrugated		Yes	Yes
In the corn	Cement	Cemented mud	Iron sheet (corrugated		Yes	Yes
In the corn	Cement	Iron sheets(mabati)	Iron sheet (corrugated		Yes	Yes
In the corn	Cement	Iron sheets(mabati)	Iron sheet (corrugated		Yes	Yes
In the corn	Cement	Stone	Iron sheet (corrugated		Yes	Yes
In the corn	Cement	Cemented mud	Metal sheets/tin		Yes	Yes
In the corn	Cement	Stone	Iron sheet (corrugated		Yes	Yes
In the corn	Natural floor	Mud	Other (Spe	Grass/thatc	No window	No
In a separa	Polished wood	Concrete blocks	Iron sheet (corrugated		Yes	Yes

In the corn	Cement	Concrete blocks	Iron sheet (corrugated	Yes	Yes
In a separa	Cement	Cemented mud	Iron sheet (corrugated	Yes	Yes
In the corn	Cement	Iron sheets(mabati)	Iron sheet (corrugated	Yes	Yes
In a separa	Polished wood	Concrete blocks	Iron sheet (corrugated	Yes	Yes
In the corn	Cement	Cemented mud	Iron sheet (corrugated	Yes	Yes
In the corn	Cement	Cemented mud	Iron sheet (corrugated	Yes	Yes
In the corn	Cement	Cemented mud	Iron sheet (corrugated	Yes	Yes
In the corn	Cement	Cemented mud	Iron sheet (corrugated	Yes	Yes
In the corn	Cement	Stone	Iron sheet (corrugated	Yes	Yes
In the corn	Cement	Concrete blocks	Iron sheet (corrugated	Yes	Yes
In the corn	Cement	Stone	Iron sheet (corrugated	Yes	Yes
In a separa	Cement	Stone	Iron sheet (corrugated	Yes	Yes
In a separa	Cement	Stone	Iron sheet (corrugated	Yes	Yes
In the corn	Cement	Concrete blocks	Iron sheet (corrugated	Yes	Yes
In the corn	Cement	Iron sheets(mabati)	Iron sheet (corrugated	No	Yes
In the corn	Cement	Iron sheets(mabati)	Metal sheets/tin	Yes	Yes
In the corn	Cement	Iron sheets(mabati)	Metal sheets/tin	Yes	Yes
In the corn	Cement	Cemented mud	Iron sheet (corrugated	Yes	Yes
In the corn	Cement	Stone	Metal sheets/tin	Yes	Yes
In the corn	Cement	Stone	Iron sheet (corrugated	Yes	Yes
In the corn	Cement	Cemented mud	Iron sheet (corrugated	Yes	Yes
In the corn	Cement	Iron sheets(mabati)	Metal sheets/tin	Yes	Yes
In a separa	Cement	Concrete blocks	Iron sheet (corrugated	Yes	Yes
In the corn	Cement	Iron sheets(mabati)	Metal sheets/tin	Yes	Yes
In the corn	Cement	Mud/Stone/Bricks mix	Iron sheet (corrugated	Yes	Yes
In the corn	Cement	Iron sheets(mabati)	Metal sheets/tin	Yes	Yes
In the corn	Cement	Stone	Metal sheets/tin	Yes	Yes
In the corn	Cement	Cemented mud	Iron sheet (corrugated	No	Yes
In the corn	Polished wood	Stone	Iron sheet (corrugated	Yes	Yes
In the corn	Polished wood	Mud/Stone/Bricks mix	Iron sheet (corrugated	No	Yes
Outside in	Cement	Concrete blocks	Iron sheet (corrugated	No	Yes
In the corn	Cement	Iron sheets(mabati)	Iron sheet (corrugated	No window	Yes
In a separa	Cement	Stone	Iron sheet (corrugated	Yes	Yes
In the corn	Cement	Iron sheets(mabati)	Iron sheet (corrugated	Yes	Yes
In the corn	Natural floor	Cemented mud	Iron sheet (corrugated	Yes	Yes
In a separa	Cement	Stone	Iron sheet (corrugated	Yes	Yes
In another	Cement	Cemented mud	Iron sheet (corrugated	Yes	Yes
In the corn	Cement	Iron sheets(mabati)	Iron sheet (corrugated	Yes	Yes
In the corn	Cement	Iron sheets(mabati)	Iron sheet (corrugated	No	Yes
In a separa	Cement	Concrete blocks	Iron sheet (corrugated	Yes	Yes
In the corn	Cement	Mud	Iron sheet (corrugated	No	Yes
In a separa	Cement	Cemented mud	Metal sheets/tin	Yes	Yes
In a separa	Cement	Cemented mud	Iron sheet (corrugated	Yes	Yes
In the corn	Cement	Iron sheets(mabati)	Iron sheet (corrugated	Yes	Yes
In the corn	Cement	Stone	Iron sheet (corrugated	Yes	Yes
In the corn	Cement	Cemented mud	Iron sheet (corrugated	Yes	Yes
In the corn	Cement	Concrete blocks	Iron sheet (corrugated	Yes	Yes
In a separa	Polished wood	Mud/Stone/Bricks mix	Iron sheet (corrugated	Yes	Yes
In the corn	Cement	Stone	Metal sheets/tin	Yes	Yes

In a separa	Cement	Concrete blocks	Iron sheet (corrugated	Yes	Yes
In a separa	Natural floor	Mud	Iron sheet (corrugated	Yes	Yes
In the corn	Cement	Concrete blocks	Iron sheet (corrugated	Yes	Yes
In a separa	Cement	Stone	Iron sheet (corrugated	Yes	Yes
In the corn	Polished wood	Concrete blocks	Iron sheet (corrugated	Yes	Yes
In the corn	Cement	Stone	Iron sheet (corrugated	Yes	Yes
In the corn	Cement	Iron sheets(mabati)	Iron sheet (corrugated	Yes	Yes
In another	Polished wood	Stone	Iron sheet (corrugated	Yes	Yes
In a separa	Polished wood	Concrete blocks	Iron sheet (corrugated	Yes	Yes
In the corn	Cement	Iron sheets(mabati)	Iron sheet (corrugated	Yes	Yes
In the corn	Cement	Concrete blocks	Metal sheets/tin	Yes	Yes
In a separa	Cement	Cemented mud	Iron sheet (corrugated	Yes	Yes
In the corn	Cement	Iron sheets(mabati)	Iron sheet (corrugated	Yes	Yes
In the corn	Cement	Iron sheets(mabati)	Iron sheet (corrugated	Yes	Yes
In the corn	Cement	Mud/Stone/Bricks mix	Iron sheet (corrugated	Yes	Yes
In a separa	Cement	Stone	Iron sheet (corrugated	Yes	Yes
Outside in	Natural floor	Stone	Iron sheet (corrugated	Yes	Yes
In the corn	Cement	Cemented mud	Iron sheet (corrugated	No	Yes
In a separa	Cement	Cemented mud	Iron sheet (corrugated	Yes	Yes
Outside in	Natural floor	Stone	Iron sheet (corrugated	Yes	Yes
In a separa	Natural floor	Mud	Iron sheet (corrugated	No	Yes
In the corn	Cement	Iron sheets(mabati)	Iron sheet (corrugated	Yes	Yes
In another	Cement	Concrete blocks	Iron sheet (corrugated	Yes	Yes
In a separa	Cement	Concrete blocks	Iron sheet (corrugated	Yes	Yes
In the corn	Cement	Concrete blocks	Iron sheet (corrugated	Yes	Yes
In the corn	Cement	Concrete blocks	Iron sheet (corrugated	Yes	Yes
In a separa	Polished wood	Stone	Iron sheet (corrugated	No window	Yes
In the corn	Cement	Cemented mud	Iron sheet (corrugated	Yes	Yes
In the corn	Cement	Concrete blocks	Iron sheet (corrugated	Yes	Yes
In a separa	Natural floor	Iron sheets(mabati)	Iron sheet (corrugated	No	Yes
In the corn	Cement	Mud/Stone/Bricks mix	Metal sheets/tin	Yes	Yes
In the corn	Cement	Stone	Iron sheet (corrugated	Yes	Yes
In a separa	Cement	Mud/Stone/Bricks mix	Iron sheet (corrugated	Yes	Yes
In a separa	Cement	Cemented mud	Iron sheet (corrugated	No	Yes
In the corn	Cement	Concrete blocks	Iron sheet (corrugated	Yes	Yes
In the corn	Cement	Iron sheets(mabati)	Iron sheet (corrugated	Yes	Yes
In the corn	Cement	Stone	Iron sheet (corrugated	Yes	Yes
In a separa	Cement	Concrete blocks	Iron sheet (corrugated	Yes	Yes
In a separa	Polished wood	Cemented mud	Iron sheet (corrugated	Yes	Yes
In the corn	Cement	Iron sheets(mabati)	Iron sheet (corrugated	Yes	Yes
In the corn	Cement	Concrete blocks	Iron sheet (corrugated	Yes	Yes
In the corn	Cement	Cemented mud	Iron sheet (corrugated	Yes	Yes
In the corn	Cement	Stone	Iron sheet (corrugated	Yes	Yes
In the corn	Cement	Iron sheets(mabati)	Iron sheet (corrugated	Yes	Yes
In a separa	Cement	Stone	Metal sheets/tin	Yes	Yes
In the corn	Cement	Concrete blocks	Iron sheet (corrugated	Yes	Yes
In the corn	Cement	Stone	Iron sheet (corrugated	Yes	Yes
In a separa	Polished wood	Iron sheets(mabati)	Iron sheet (corrugated	Yes	Yes
In the corn	Cement	Mud	Iron sheet (corrugated	No	Yes

In the corn	Cement	Cemented mud	Iron sheet (corrugated	No	Yes
In a separa	Polished wood	Concrete blocks	Iron sheet (corrugated	Yes	Yes
In the corn	Cement	Cemented mud	Iron sheet (corrugated	Yes	Yes
In the corn	Cement	Cemented mud	Iron sheet (corrugated	Yes	Yes
In the corn	Cement	Concrete blocks	Iron sheet (corrugated	Yes	Yes
In the corn	Cement	Cemented mud	Iron sheet (corrugated	Yes	Yes
In a separa	Polished wood	Concrete blocks	Iron sheet (corrugated	Yes	Yes
In a separa	Natural floor	Mud	Iron sheet (corrugated	Yes	Yes
In the corn	Cement	Iron sheets(mabati)	Iron sheet (corrugated	Yes	Yes
In the corn	Cement	Cemented mud	Iron sheet (corrugated	Yes	Yes
In a separa	Polished wood	Stone	Iron sheet (corrugated	Yes	Yes
In the corn	Natural floor	Concrete blocks	Iron sheet (corrugated	No	Yes
In the corn	Cement	Stone	Iron sheet (corrugated	Yes	Yes
In the corn	Natural floor	Mud	Iron sheet (corrugated	Yes	Yes
In a separa	Cement	Mud/Stone Bricks and	Iron sheet (corrugated	No window	Yes
In a separa	Cement	Mud/Stone/Bricks mix	Iron sheet (corrugated	No	Yes
In the corn	Cement	Iron sheets(mabati)	Iron sheet (corrugated	Yes	Yes
Outside in	Cement	Stone	Iron sheet (corrugated	Yes	Yes
In the corn	Cement	Iron sheets(mabati)	Iron sheet (corrugated	No	Yes
In the corn	Cement	Mud	Iron sheet (corrugated	Yes	Yes
In the corn	Cement	Cemented mud	Iron sheet (corrugated	Yes	Yes
In the corn	Cement	Iron sheets(mabati)	Metal sheets/tin	Yes	Yes
In the corn	Cement	Iron sheets(mabati)	Iron sheet (corrugated	Yes	Yes
In a separa	Cement	Mud/Stone Mud bricks	Iron sheet (corrugated	Yes	Yes
In the corn	Cement	Iron sheets(mabati)	Iron sheet (corrugated	Yes	Yes

q23	q24a	q24b	q25	q26a	q26b	q27	q28a	q28_other
5	Earth		None	Public tap or fountain		4	Per 20 liter jerrican	
6	Earth		None	Tap in the compound		0	Monthly	
5	Cement		lockable ga	Public tap or fountain		10	Per 20 liter jerrican	
5	Grass		None	Borehole		10	Per 20 liter jerrican	
1	Earth		lockable ga	Public tap or fountain		15	Per 20 liter jerrican	
60	Cement		lockable ga	Public tap or fountain		5	Per 20 liter jerrican	
3	Earth		lockable ga	Public tap or fountain		15	Per 20 liter jerrican	
11	Earth		No gate	Public tap or fountain		5	Per 20 liter jerrican	
5	Earth		None	Public tap or fountain		10	Per 20 liter jerrican	
4	Cement		lockable ga	Tap in the compound		1	Do not pay	
56	Earth		lockable ga	Tap in neig	At the land	5	Per 20 liter jerrican	
15	Grass		No gate	Tap in the compound		10	Per 20 liter jerrican	
1	Earth		No gate	Public tap or fountain		1	Per 20 liter jerrican	
5	Earth		lockable ga	Tap in the compound		2	Included in Rent	
9	Grass		lockable ga	Other unim	From wate	0	Per 20 liter jerrican	
20	Cement		lockable ga	Public tap or fountain		5	Per 20 liter jerrican	
20	Earth		lockable ga	Tap in the compound		5	Included in Rent	
2	Earth		None	Borehole		25	Per 20 liter jerrican	
60	Cement		lockable ga	Tap in the compound		5	Included in Rent	
1	Earth		None	Public tap or fountain		7	Per 20 liter jerrican	
22	Earth		lockable ga	Tap in the compound		1	Per 20 liter jerrican	
35	Grass		lockable ga	Tap inside the house		5	Per 20 liter jerrican	
7	Earth		lockable ga	Public tap or fountain		20	Per 20 liter jerrican	
1	Earth		None	Public tap or fountain		20	Per 20 liter jerrican	
10	Cement		lockable ga	Public tap or fountain		0	Monthly	
6	Earth		lockable ga	Other unim	Water vend	0	Per 20 liter jerrican	
9	Cement		lockable ga	Public tap or fountain		3	Per 20 liter jerrican	
24	Cement		lockable ga	Tap in the compound		2	Included in Rent	
3	Earth		None	Tap in the compound		2	Per 20 liter jerrican	
1	Earth		None	Borehole		10	Per 20 liter jerrican	
9	Earth		lockable ga	Tap in the compound		5	Monthly	
1	Earth		None	Borehole		5	Per 20 liter jerrican	
30	Grass		lockable ga	Tap in the compound		2	Monthly	
10	Earth		None	Public tap or fountain		10	Per 20 liter jerrican	
1	Earth		None	Public tap or fountain		10	Per 20 liter jerrican	
1	Earth		None	Public tap or fountain		15	Per 20 liter jerrican	
20	Earth		lockable ga	Tap in the compound		0	Per 20 liter jerrican	
1	Earth		None	Borehole		0	Per 20 liter jerrican	
5	Earth		lockable ga	Public tap or fountain		1	Per 20 liter jerrican	
4	Cement		lockable ga	Borehole		1	Included in Rent	
23	Earth		No gate	Tap in the compound		30	Monthly	
4	Earth		None	Other Improved sourc		5	Per 20 liter jerrican	
1	Earth		None	Tap in the compound		10	Per 20 liter jerrican	
1	Earth		None	Public tap or fountain		10	Per 20 liter jerrican	
31	Cement		lockable ga	Tap in the compound		1	Monthly	
28	Cement		lockable ga	Tap in the compound		3	Monthly	
15	Cement		lockable ga	Tap inside the house		1	Included in Rent	
1	Earth		None	Borehole		10	Per 20 liter jerrican	

14	Earth	No gate	Public tap or fountain	15	Per 20 liter jerrican
8	Earth	lockable ga	Tap in the compound	30	Included in Rent
1	Earth	None	Borehole	15	Per 20 liter jerrican
1	Earth	No gate	Public tap or fountain	5	Per 20 liter jerrican
1	Earth	None	Borehole	15	Per 20 liter jerrican
69	Cement	lockable ga	Borehole	1	Included in Rent
1	Earth	None	Public tap or fountain	5	Per 20 liter jerrican
1	Earth	lockable ga	Public tap or fountain	4	Per 20 liter jerrican
1	Earth	No gate	Public tap or fountain	10	Per 20 liter jerrican
1	Cement	lockable ga	Tap in the compound	0	Monthly
6	Earth	lockable ga	Public tap or fountain	2	Per 20 liter jerrican
9	Earth	None	Public tap or fountain	3	Per 20 liter jerrican
14	Earth	lockable ga	Public tap or fountain	5	Per 20 liter jerrican
1	Cement	lockable ga	Public tap or fountain	3	Per 20 liter jerrican
1	Earth	None	Borehole	20	Per 20 liter jerrican
8	Cement	lockable ga	Tap inside the house	0	Monthly
5	Cement	lockable ga	Tap in neig Tap in a ne	5	Per 20 liter jerrican
17	Cement	lockable ga	Tap inside the house	1	Monthly
45	Cement	lockable ga	Tap in the compound	2	Monthly
150	Earth	lockable ga	Tap in the compound	2	Included in Rent
9	Earth	lockable ga	Public tap or fountain	2	Per 20 liter jerrican
3	Earth	lockable ga	Tap in the compound	1	Included in Rent
36	Earth	lockable ga	Public tap or fountain	3	Per 20 liter jerrican
1	Grass	None	Public tap or fountain	10	Per 20 liter jerrican
34	Earth	lockable ga	Tap in neig Tap water	6	Per 20 liter jerrican
1	Earth	None	Other Improved sourc	20	Per 20 liter jerrican
3	Earth	None	Borehole	20	Per 20 liter jerrican
3	Earth	lockable ga	Tap in the compound	5	Monthly
1	Earth	None	Borehole	10	Included in Rent
8	Earth	None	Borehole	2	Per 20 liter jerrican
1	Earth	None	Borehole	20	Per 20 liter jerrican
3	Earth	None	Public tap or fountain	10	Per 20 liter jerrican
1	Earth	None	Public tap or fountain	10	Per 20 liter jerrican
3	Earth	lockable ga	Public tap or fountain	1	Per 20 liter jerrican
2	Earth	None	Public tap or fountain	10	Per 20 liter jerrican
1	Earth	None	Public tap or fountain	2	Per 20 liter jerrican
6	Earth	lockable ga	Public tap or fountain	1	Per 20 liter jerrican
1	Earth	None	Public tap or fountain	10	Per 20 liter jerrican
11	Earth	lockable ga	Borehole	2	Per 20 liter jerrican
1	Earth	None	Public tap or fountain	5	Per 20 liter jerrican
58	Cement	lockable ga	Tap in neig They get w	15	Per 20 liter jerrican
4	Cement	lockable ga	Tap in the compound	5	Monthly
4	Earth	lockable ga	Public tap or fountain	25	Per 20 liter jerrican
1	Earth	None	Borehole	10	Per 20 liter jerrican
1	Earth	None	Public tap or fountain	15	Per 20 liter jerrican
8	Cement	lockable ga	Tap in the compound	2	Per 20 liter jerrican
8	Earth	None	Public tap or fountain	5	Per 20 liter jerrican
1	Earth	None	Tap in the compound	10	Per 20 liter jerrican
12	Earth	None	Public tap or fountain	10	Per 20 liter jerrican

10	Earth	unlockable	Public tap or fountain	20	Per 20 liter jerrican	
1	Earth	None	Borehole	3	Per 20 liter jerrican	
11	Grass	None	Public tap or fountain	20	Per 20 liter jerrican	
3	Earth	None	Public tap or fountain	20	Per 20 liter jerrican	
4	Earth	lockable ga	Public tap or fountain	30	Per 20 liter jerrican	
1	Earth	None	Borehole	5	Do not pay	
2	Grass	None	Borehole	3	Included in Rent	
1	Earth	None	Public tap or fountain	15	Per 20 liter jerrican	
1	Earth	lockable ga	Public tap or fountain	15	Per 20 liter jerrican	
4	Earth	None	Public tap or fountain	10	Per 20 liter jerrican	
5	Cement	lockable ga	Public tap or fountain	15	Per 20 liter jerrican	
2	Earth	lockable ga	Public tap or fountain	1	Monthly	
3	Cement	lockable ga	Tap in the compound	3	Monthly	
3	Cement	None	Tap inside the house	1	Monthly	
1	Grass	None	Public tap or fountain	20	Per 20 liter jerrican	
1	Earth	None	Public tap or fountain	3	Per 20 liter jerrican	
15	Earth	lockable ga	Public tap or fountain	10	Per 20 liter jerrican	
1	Earth	None	Tap inside the house	0	Do not pay	
9	Earth	None	Borehole	2	Monthly	
6	Cement	lockable ga	Public tap or fountain	10	Per 20 liter jerrican	
5	Earth	lockable ga	Public tap or fountain	7	Per 20 liter jerrican	
7	Earth	None	Public tap or fountain	30	Per 20 liter jerrican	
1	Earth	None	Borehole	20	Per 20 liter jerrican	
1	Earth	unlockable	Public tap or fountain	1	Monthly	
1	Earth	None	Borehole	15	Per 20 liter jerrican	
7	Earth	No gate	Tap in the compound	2	Per 20 liter jerrican	
1	Cement	lockable ga	Public tap or fountain	20	Per 20 liter jerrican	
11	Earth	None	Tap in the compound	1	Monthly	
5	Grass	lockable ga	Tap in the compound	4	Do not pay	
45	Cement	lockable ga	Tap in the compound	5	Monthly	
13	Cement	No gate	Public tap or fountain	5	Per 20 liter jerrican	
51	Cement	lockable ga	Tap in the compound	1	Included in Rent	
7	Cement	lockable ga	Public tap or fountain	2	Per 20 liter jerrican	
15	Earth	unlockable	Tap in the compound	1	Included in Rent	
6	Earth	None	Public tap or fountain	5	Per 20 liter jerrican	
6	Cement	lockable ga	Tap in the compound	2	Included in Rent	
14	Cement	unlockable	Public tap or fountain	10	Per 20 liter jerrican	
5	Earth	unlockable	Public tap or fountain	5	Per 20 liter jerrican	
1	Earth	lockable ga	Public tap or fountain	2	Per 20 liter jerrican	
1	Earth	None	Tap in the compound	5	Per 20 liter jerrican	
1	Earth	None	Borehole	15	Included in Rent	
1	Earth	lockable ga	Other Improved sourc	2	Do not pay	Only pay tc
40	Earth	lockable ga	Tap in the compound	3	Per 20 liter jerrican	
4	Earth	No gate	Public tap or fountain	10	Per 20 liter jerrican	
8	Cement	lockable ga	Public tap or fountain	15	Per 20 liter jerrican	
10	Cement	lockable ga	Tap inside the house	1	Monthly	
1	Earth	None	Public tap or fountain	3	Per 20 liter jerrican	
1	Earth	None	Public tap or fountain	15	Per 20 liter jerrican	
6	Earth	unlockable	Tap in the compound	2	Do not pay	

1	Earth	None	Public tap or fountain	10	Per 20 liter jerrican
1	Earth	None	Public tap or fountain	20	Per 20 liter jerrican
4	Cement	lockable ga	Tap in the compound	0	Included in Rent
35	Cement	lockable ga	Tap in the compound	3	Included in Rent
1	Earth	lockable ga	Public tap or fountain	2	Per 20 liter jerrican
4	Cement	lockable ga	Public tap or fountain	5	Per 20 liter jerrican
23	Earth	lockable ga	Tap in the compound	10	Included in Rent
7	Earth	unlockable	Public tap or fountain	10	Per 20 liter jerrican
30	Cement	lockable ga	Tap in the compound	2	Included in Rent
1	Earth	None	Tap in the compound	0	Monthly
30	Earth	lockable ga	Tap in the compound	2	Included in Rent
7	Earth	No gate	Tap in the compound	2	Per 20 liter jerrican
4	Earth	lockable ga	Public tap or fountain	3	Per 20 liter jerrican
11	Cement	lockable ga	Tap in the compound	5	Monthly
1	Earth	None	Borehole	15	Per 20 liter jerrican
3	Grass	No gate	Borehole	2	Per 20 liter jerrican
5	Earth	unlockable	Public tap or fountain	5	Per 20 liter jerrican
1	Earth	None	Borehole	6	Included in Rent
1	Earth	None	Public tap or fountain	5	Per 20 liter jerrican
1	Earth	None	Borehole	10	Per 20 liter jerrican
14	Earth	None	Public tap or fountain	5	Per 20 liter jerrican
1	Earth	None	Borehole	15	Per 20 liter jerrican
1	Earth	None	Public tap or fountain	3	Per 20 liter jerrican
9	Earth	lockable ga	Public tap or fountain	6	Per 20 liter jerrican
2	Earth	None	Public tap or fountain	20	Per 20 liter jerrican
1	Earth	None	Tap in neig Tap in the	15	Per 20 liter jerrican
6	Grass	None	Public tap or fountain	3	Per 20 liter jerrican
3	Earth	None	Public tap or fountain	5	Per 20 liter jerrican
1	Earth	None	Borehole	20	Per 20 liter jerrican
1	Earth	None	Public tap or fountain	3	Per 20 liter jerrican
2	Earth	lockable ga	Public tap or fountain	10	Per 20 liter jerrican
12	Earth	None	Public tap or fountain	3	Per 20 liter jerrican
1	Earth	None	Public tap or fountain	1	Per 20 liter jerrican
1	Earth	None	Public tap or fountain	5	Per 20 liter jerrican
1	Cement	lockable ga	Tap inside the house	2	Monthly
1	Earth	None	Borehole	15	Per 20 liter jerrican
1	Earth	None	Tap in the compound	10	Per 20 liter jerrican
35	Earth	lockable ga	Tap in the compound	2	Included in Rent
6	Grass	lockable ga	Tap in the compound	1	Included in Rent
7	Earth	None	Borehole	2	Per 20 liter jerrican
1	Earth	None	Public tap or fountain	5	Per 20 liter jerrican
1	Earth	None	Borehole	2	Do not pay
5	Earth	lockable ga	Public tap or fountain	1	Per 20 liter jerrican
19	Cement	lockable ga	Tap in the compound	2	Included in Rent
6	Earth	None	Public tap or fountain	15	Per 20 liter jerrican
1	Earth	None	Tap inside the house	2	Included in Rent
1	Earth	None	Borehole	30	Per 20 liter jerrican
35	Earth	lockable ga	Tap in the compound	15	Included in Rent
1	Earth	None	Borehole	10	Per 20 liter jerrican

8	Earth	lockable ga	Public tap or fountain		1	Per 20 liter jerrican
1	Earth	None	Tap in the compound		1	Monthly
3	Earth	None	Public tap or fountain		10	Per 20 liter jerrican
1	Earth	None	Tap inside the house		0	Do not pay
2	Earth	None	Public tap or fountain		10	Per 20 liter jerrican
8	Cement	lockable ga	Tap in the compound		1	Monthly
4	Grass	None	Public tap or fountain		30	Per 20 liter jerrican
1	Grass	None	Borehole		10	Per 20 liter jerrican
1	Earth	None	Tap in the compound		10	Per 20 liter jerrican
50	Earth	lockable ga	Tap in the compound		5	Monthly
1	Earth	None	Tap inside the house		2	Included in Rent
4	Cement	lockable ga	Tap in the compound		1	Included in Rent
9	Earth	lockable ga	Tap in the compound		5	Monthly
10	Cement	lockable ga	Public tap or fountain		1	Per 20 liter jerrican
8	Cement	No gate	Tap in the compound		1	Per 20 liter jerrican
4	Earth	None	Public tap or fountain		2	Per 20 liter jerrican
1	Earth	None	Public tap or fountain		5	Per 20 liter jerrican
25	Cement	lockable ga	Tap in the compound		10	Included in Rent
30	Earth	lockable ga	Tap in the compound		3	Included in Rent
1	Earth	None	Public tap or fountain		5	Per 20 liter jerrican
1	Earth	None	Tap in the compound		1	Monthly
40	Cement	lockable ga	Public tap or fountain		30	Per 20 liter jerrican
6	Earth	lockable ga	Tap in the compound		5	Monthly
4	Earth	None	Public tap or fountain		20	Per 20 liter jerrican
1	Earth	None	Tap in the compound		10	Per 20 liter jerrican
7	Earth	unlockable	Tap in the compound		3	Monthly
6	Earth	None	Public tap or fountain		10	Per 20 liter jerrican
1	Grass	None	Public tap or fountain		20	Included in Rent
10	Earth	lockable ga	Tap in the compound		1	Included in Rent
63	Earth	lockable ga	Tap in the compound		30	Per 20 liter jerrican
1	Earth	None	Tap inside the house		0	Per 20 liter jerrican
1	Earth	None	Public tap or fountain		1	Per 20 liter jerrican
46	Earth	lockable ga	Tap in the compound		3	Included in Rent
3	Earth	None	Public tap or fountain		12	Per 20 liter jerrican
18	Cement	lockable ga	Tap in neig	Piped regu	3	Per 20 liter jerrican
1	Earth	None	Borehole		10	Per 20 liter jerrican
10	Earth	None	Public tap or fountain		20	Per 20 liter jerrican
6	Earth	lockable ga	Public tap or fountain		10	Per 20 liter jerrican
1	Earth	None	Borehole		5	Per 20 liter jerrican
12	Cement	lockable ga	Public tap or fountain		30	Per 20 liter jerrican
10	Earth	None	Tap in the compound		5	Monthly
2	Cement	lockable ga	Public tap or fountain		4	Per 20 liter jerrican
1	Earth	None	Tap inside the house		5	Included in Rent
3	Cement	lockable ga	Public tap or fountain		1	Per 20 liter jerrican
1	Earth	None	Public tap or fountain		5	Per 20 liter jerrican
12	Earth	lockable ga	Public tap or fountain		10	Per 20 liter jerrican
40	Earth	lockable ga	Tap in the compound		2	Included in Rent
10	Earth	None	Public tap or fountain		4	Per 20 liter jerrican
13	Earth	lockable ga	Public tap or fountain		2	Per 20 liter jerrican

1	Earth	None	Public tap or fountain	1	Monthly
3	Cement	lockable ga	Tap inside the house	0	Monthly
9	Cement	lockable ga	Public tap or fountain	5	Per 20 liter jerrican
8	Cement	lockable ga	Tap in neig Tap water	20	Per 20 liter jerrican
9	Earth	lockable ga	Other Improved sourc	5	Per 20 liter jerrican
34	Earth	lockable ga	Tap in the compound	2	Included in Rent
1	Earth	None	Public tap or fountain	15	Per 20 liter jerrican
16	Earth	lockable ga	Public tap or fountain	5	Per 20 liter jerrican
1	Earth	lockable ga	Tap in neig Tap in ano	5	Per 20 liter jerrican
1	Earth	No gate	Public tap or fountain	10	Per 20 liter jerrican
8	Earth	lockable ga	Public tap or fountain	5	Per 20 liter jerrican
1	Earth	None	Tap inside the house	1	Monthly
6	Earth	lockable ga	Public tap or fountain	5	Per 20 liter jerrican
1	Earth	None	Public tap or fountain	15	Per 20 liter jerrican
7	Cement	lockable ga	Tap in the compound	2	Included in Rent
6	Earth	lockable ga	Tap in the compound	2	Included in Rent
1	Earth	None	Public tap or fountain	15	Per 20 liter jerrican
100	Earth	lockable ga	Tap in the compound	60	Included in Rent
1	Earth	None	Public tap or fountain	10	Per 20 liter jerrican
7	Cement	lockable ga	Public tap or fountain	5	Per 20 liter jerrican
3	Earth	No gate	Borehole	2	Per 20 liter jerrican
3	Earth	None	Public tap or fountain	5	Per 20 liter jerrican
1	Earth	None	Tap in the compound	10	Per 20 liter jerrican
9	Cement	lockable ga	Tap in the compound	2	Included in Rent
112	Earth	lockable ga	Public tap or fountain	10	Per 20 liter jerrican
2	Earth	None	Tap in the compound	10	Per 20 liter jerrican
16	Earth	None	Public tap or fountain	5	Per 20 liter jerrican
1	Earth	None	Public tap or fountain	3	Per 20 liter jerrican
3	Earth	lockable ga	Public tap or fountain	3	Do not pay
6	Grass	None	Borehole	5	Per 20 liter jerrican
20	Grass	lockable ga	Tap in the compound	2	Included in Rent
1	Earth	lockable ga	Public tap or fountain	5	Per 20 liter jerrican
25	Cement	lockable ga	Tap in the compound	10	Included in Rent
1	Earth	None	Tap in the compound	10	Per 20 liter jerrican
7	Earth	No gate	Borehole	8	Per 20 liter jerrican
4	Grass	None	Public tap or fountain	10	Per 20 liter jerrican
20	Cement	lockable ga	Other unimproved sou	3	Included in Rent
14	Cement	lockable ga	Public tap or fountain	20	Per 20 liter jerrican
20	Earth	lockable ga	Tap in the compound	4	Included in Rent
1	Earth	None	Borehole	20	Per 20 liter jerrican
30	Grass	lockable ga	Public tap or fountain	10	Per 20 liter jerrican
1	Earth	None	Borehole	3	Per 20 liter jerrican
2	Earth	None	Public tap or fountain	2	Per 20 liter jerrican
1	Grass	None	Borehole	20	Per 20 liter jerrican
1	Earth	None	Public tap or fountain	998	Per 20 liter jerrican
10	Earth	lockable ga	Public tap or fountain	5	Per 20 liter jerrican
120	Earth	lockable ga	Tap in the compound	5	Monthly
13	Earth	lockable ga	Tap in the compound	1	Monthly
16	Earth	lockable ga	Tap in the compound	1	Included in Rent

60	Cement	lockable ga	Tap in the compound		20	Included in Rent
2	Grass	None	Public tap or fountain		10	Per 20 liter jerrican
16	Cement	lockable ga	Tap in the compound		1	Included in Rent
4	Grass	None	Public tap or fountain		5	Per 20 liter jerrican
9	Earth	unlockable	Tap in neig	Tap in the	2	Per 20 liter jerrican
4	Earth	unlockable	Public tap or fountain		10	Per 20 liter jerrican
3	Earth	lockable ga	Public tap or fountain		10	Per 20 liter jerrican
7	Earth	None	Public tap or fountain		5	Per 20 liter jerrican
1	Earth	None	Borehole		15	Per 20 liter jerrican
10	Earth	lockable ga	Public tap or fountain		3	Per 20 liter jerrican
9	Earth	No gate	Public tap or fountain		2	Per 20 liter jerrican
1	Earth	None	Borehole		10	Per 20 liter jerrican
18	Earth	lockable ga	Public tap or fountain		1	Per 20 liter jerrican
1	Earth	None	Borehole		15	Per 20 liter jerrican
4	Earth	None	Borehole		10	Per 20 liter jerrican
20	Cement	lockable ga	Tap in the compound		5	Included in Rent
27	Cement	lockable ga	Tap in the compound		5	Per 20 liter jerrican
4	Earth	lockable ga	Public tap or fountain		1	Per 20 liter jerrican
7	Earth	lockable ga	Other unim	Water vend	3	Per 20 liter jerrican
10	Cement	lockable ga	Public tap or fountain		3	Per 20 liter jerrican
1	Earth	None	Public tap or fountain		5	Per 20 liter jerrican
9	Earth	None	Public tap or fountain		5	Per 20 liter jerrican
2	Earth	lockable ga	Tap in the compound		2	Included in Rent
1	Earth	lockable ga	Public tap or fountain		10	Per 20 liter jerrican
4	Earth	No gate	Public tap or fountain		2	Per 20 liter jerrican
30	Grass	lockable ga	Tap in the compound		3	Included in Rent
21	Grass	unlockable	Public tap or fountain		2	Per 20 liter jerrican
4	Earth	None	Borehole		5	Per 20 liter jerrican
2	Cement	lockable ga	Tap inside the house		2	Monthly
15	Grass	lockable ga	Tap in the compound		2	Per 20 liter jerrican
1	Earth	None	Public tap or fountain		5	Per 20 liter jerrican
3	Earth	lockable ga	Public tap or fountain		5	Per 20 liter jerrican
12	Cement	lockable ga	Tap in the compound		2	Included in Rent
5	Earth	None	Public tap or fountain		5	Per 20 liter jerrican
1	Cement	lockable ga	Public tap or fountain		10	Per 20 liter jerrican
1	Earth	None	Public tap or fountain		20	Per 20 liter jerrican
4	Earth	lockable ga	Tap in neig	Fectch fror	2	Monthly
40	Cement	lockable ga	Tap in neig	Tap in the	15	Included in Rent
12	Grass	lockable ga	Tap in the compound		1	Included in Rent
6	Earth	unlockable	Borehole		3	Per 20 liter jerrican
4	Grass	lockable ga	Tap inside the house		1	Monthly
75	Cement	lockable ga	Tap in the compound		5	Included in Rent
42	Cement	lockable ga	Tap inside the house		0	Included in Rent
10	Earth	None	Public tap or fountain		30	Per 20 liter jerrican
22	Cement	lockable ga	Tap in the compound		1	Included in Rent
20	Grass	lockable ga	Tap in the compound		1	Monthly
1	Earth	None	Borehole		10	Per 20 liter jerrican
6	Grass	unlockable	Public tap or fountain		5	Per 20 liter jerrican
7	Earth	unlockable	Tap in the compound		2	Per 20 liter jerrican

49	Earth	lockable ga	Tap in the compound		3	Included in Rent
21	Grass	lockable ga	Tap in the compound		1	Included in Rent
3	Grass	unlockable	Public tap or fountain		5	Per 20 liter jerrican
20	Cement	lockable ga	Tap in the compound		1	Included in Rent
3	Grass	unlockable	Borehole		3	Per 20 liter jerrican
5	Earth	None	Borehole		20	Per 20 liter jerrican
46	Cement	lockable ga	Tap in the compound		2	Included in Rent
4	Earth	None	Public tap or fountain		10	Per 20 liter jerrican
12	Cement	lockable ga	Tap inside the house		0	Monthly
15	Cement	None	Other unim	Water vend	0	Per 20 liter jerrican
15	Earth	unlockable	Public tap or fountain		3	Per 20 liter jerrican
1	Earth	None	Public tap or fountain		1	Per 20 liter jerrican
3	Earth	None	Public tap or fountain		10	Per 20 liter jerrican
6	Earth	None	Public tap or fountain		4	Per 20 liter jerrican
7	Earth	lockable ga	Public tap or fountain		1	Per 20 liter jerrican
15	Earth	lockable ga	Public tap or fountain		3	Per 20 liter jerrican
5	Grass	lockable ga	Tap in the compound		0	Do not pay
22	Cement	lockable ga	Tap in the compound		20	Per 20 liter jerrican
4	Earth	lockable ga	Public tap or fountain		10	Per 20 liter jerrican
4	Grass	None	Public tap or fountain		10	Per 20 liter jerrican
5	Earth	lockable ga	Other Improved sourc		1	Per 20 liter jerrican
33	Earth	lockable ga	Tap in the compound		5	Included in Rent
26	Cement	lockable ga	Tap in the compound		2	Monthly
10	Cement	lockable ga	Public tap or fountain		10	Per 20 liter jerrican
12	Cement	lockable ga	Tap in the compound		5	Included in Rent
22	Earth	lockable ga	Tap in the compound		60	Per 20 liter jerrican
3	Cement	lockable ga	Tap inside the house		1	Monthly
1	Earth	lockable ga	Public tap or fountain		2	Per 20 liter jerrican
1	Earth	None	Borehole		5	Per 20 liter jerrican
6	Earth	lockable ga	Public tap or fountain		1	Per 20 liter jerrican
10	Cement	lockable ga	Public tap or fountain		5	Included in Rent
40	Cement	lockable ga	Tap in the compound		2	Included in Rent
8	Earth	lockable ga	Borehole		1	Per 20 liter jerrican
1	Earth	None	Borehole		15	Per 20 liter jerrican
6	Earth	None	Tap in the compound		2	Per 20 liter jerrican
3	Grass	lockable ga	Public tap or fountain		1	Per 20 liter jerrican
20	Cement	lockable ga	Tap in the compound		5	Monthly
18	Grass	lockable ga	Tap in the compound		10	Included in Rent
12	Earth	None	Public tap or fountain		5	Per 20 liter jerrican
3	Grass	lockable ga	Public tap or fountain		30	Per 20 liter jerrican
7	Earth	lockable ga	Tap in neig	Tap in anot	10	Per 20 liter jerrican
10	Earth	None	Tap in the compound		3	Monthly
4	Earth	No gate	Public tap or fountain		1	Per 20 liter jerrican
12	Earth	lockable ga	Tap in the compound		10	Per 20 liter jerrican
5	Cement	lockable ga	Public tap or fountain		5	Per 20 liter jerrican
30	Earth	lockable ga	Tap in the compound		3	Per 20 liter jerrican
30	Cement	lockable ga	Public tap or fountain		10	Per 20 liter jerrican
8	Earth	lockable ga	Public tap or fountain		30	Per 20 liter jerrican
5	Grass	None	Public tap or fountain		2	Per 20 liter jerrican

1	Earth		lockable ga	Public tap or fountain		3	Per 20 liter jerrican
53	Earth		lockable ga	Borehole		20	Per 20 liter jerrican
25	Cement		lockable ga	Tap in the compound		10	Included in Rent
20	Earth		lockable ga	Tap in the compound		10	Included in Rent
80	Cement		lockable ga	Tap inside the house		1	Monthly
25	Earth		lockable ga	Tap in the compound		20	Included in Rent
5	Earth		lockable ga	Tap inside the house		0	Monthly
24	Cement		lockable ga	Tap inside the house		0	Monthly
60	Earth		lockable ga	Tap in the compound		1	Included in Rent
25	Cement		lockable ga	Tap in the compound		1	Per 20 liter jerrican
6	Cement		lockable ga	Tap in the compound		5	Monthly
25	Grass		lockable ga	Tap in the compound		2	Monthly
6	Cement		lockable ga	Borehole		5	Per 20 liter jerrican
2	Cement		lockable ga	Public tap or fountain		5	Per 20 liter jerrican
24	Cement		lockable ga	Tap in the compound		5	Monthly
25	Cement		lockable ga	Tap in the compound		3	Included in Rent
12	Cement		lockable ga	Tap in the compound		2	Included in Rent
32	Cement		lockable ga	Tap in the compound		1	Per 20 liter jerrican
35	Earth		lockable ga	Public tap or fountain		10	Per 20 liter jerrican
11	Earth		lockable ga	Public tap or fountain		2	Per 20 liter jerrican
12	Earth		lockable ga	Tap in the compound		2	Per 20 liter jerrican
31	Earth		lockable ga	Tap in neig	Tap in ano	5	Per 20 liter jerrican
2	Earth		None	Tap inside the house		0	Monthly
8	Earth		None	Public tap or fountain		20	Per 20 liter jerrican
10	Grass		lockable ga	Public tap or fountain		1	Per 20 liter jerrican
7	Grass		lockable ga	Tap in the compound		5	Do not pay
7	Earth		lockable ga	Public tap or fountain		3	Per 20 liter jerrican
5	Earth		lockable ga	Tap in the compound		5	Monthly
10	Grass		None	Public tap or fountain		3	Per 20 liter jerrican
10	Earth		lockable ga	Other Improved sourc		5	Per 20 liter jerrican
7	Earth		lockable ga	Tap inside the house		1	Monthly
7	Earth		lockable ga	Tap in the compound		1	Included in Rent
3	Earth		lockable ga	Tap in neig	At the land	5	Per 20 liter jerrican
5	Cement		lockable ga	Tap in the compound		2	Included in Rent
7	Grass		lockable ga	Public tap or fountain		5	Per 20 liter jerrican
8	Earth		None	Tap in the compound		5	Monthly
16	Earth		lockable ga	Tap in the compound		2	Included in Rent
3	Earth		lockable ga	Tap in the compound		1	Included in Rent
45	Cement		lockable ga	Tap in the compound		1	Monthly
1	Earth		None	Public tap or fountain		20	Per 20 liter jerrican
5	Earth		lockable ga	Tap in the compound		2	Per 20 liter jerrican
2	Cement		lockable ga	Tap in the compound		0	Do not pay
8	Cement		lockable ga	Tap in neig	Tap in neig	5	Included in Rent
12	Cement	Stones	lockable ga	Tap in neig	At the land	15	Per 20 liter jerrican
15	Cement		lockable ga	Tap in the compound		10	Monthly
1	Earth		lockable ga	Public tap or fountain		20	Per 20 liter jerrican
10	Earth		lockable ga	Tap in the compound		2	Included in Rent
2	Earth		None	Public tap or fountain		5	Per 20 liter jerrican
120	Cement		unlockable	Tap in the compound		10	Per 20 liter jerrican

24	Earth	lockable ga	Tap in the compound	4	Monthly
23	Cement	lockable ga	Tap in the compound	10	Monthly
81	Cement	lockable ga	Tap in the compound	10	Included in Rent
6	Earth	None	Public tap or fountain	5	Per 20 liter jerrican
18	Earth	No gate	Public tap or fountain	5	Per 20 liter jerrican
7	Cement	lockable ga	Tap in the compound	2	Per 20 liter jerrican
10	Earth	No gate	Tap in the compound	1	Per 20 liter jerrican
8	Earth	lockable ga	Tap in the compound	5	Included in Rent
28	Cement	lockable ga	Tap in the compound	5	Included in Rent
6	Grass	No gate	Public tap or fountain	3	Do not pay
5	Grass	lockable ga	Tap in the compound	1	Monthly
6	Cement	lockable ga	Public tap or fountain	25	Per 20 liter jerrican
1	Earth	None	Public tap or fountain	40	Per 20 liter jerrican
6	Earth	lockable ga	Public tap or fountain	1	Per 20 liter jerrican
67	Cement	lockable ga	Tap in the compound	10	Monthly
7	Grass	lockable ga	Tap in the compound	5	Monthly
1	Earth	lockable ga	Public tap or fountain	10	Per 20 liter jerrican
20	Earth	No gate	Public tap or fountain	30	Included in Rent
1	Earth	None	Public tap or fountain	10	Per 20 liter jerrican
8	Earth	None	Public tap or fountain	5	Per 20 liter jerrican
60	Cement	lockable ga	Public tap or fountain	7	Per 20 liter jerrican
6	Earth	lockable ga	Other Improved sourc	0	Per 20 liter jerrican
2	Earth	None	Borehole	20	Per 20 liter jerrican
16	Cement	lockable ga	Tap in the compound	3	Included in Rent
4	Grass	No gate	Borehole	5	Monthly
1	Earth	None	Public tap or fountain	20	Per 20 liter jerrican
1	Cement	None	Tap in the compound	2	Monthly
9	Grass	lockable ga	Public tap or fountain	10	Per 20 liter jerrican
5	Earth	None	Public tap or fountain	10	Per 20 liter jerrican
10	Earth	lockable ga	Public tap or fountain	1	Per 20 liter jerrican
8	Earth	No gate	Other Improved sourc	1	Per 20 liter jerrican
13	Cement	lockable ga	Tap in neig At the land	3	Per 20 liter jerrican
7	Earth	lockable ga	Tap in the compound	10	Included in Rent
25	Earth	lockable ga	Tap in the compound	0	Included in Rent
13	Earth	unlockable	Other unim Surface wa	10	Included in Rent
3	Cement	lockable ga	Tap in the compound	2	Included in Rent
1	Grass	No gate	Public tap or fountain	5	Per 20 liter jerrican
6	Grass	lockable ga	Tap in the compound	5	Monthly
1	Earth	None	Public tap or fountain	10	Per 20 liter jerrican
27	Cement	lockable ga	Tap in the compound	5	Included in Rent
6	Earth	lockable ga	Tap inside the house	1	Monthly
15	Cement	lockable ga	Tap in the compound	5	Included in Rent
5	Cement	lockable ga	Tap in the compound	5	Monthly
6	Earth	lockable ga	Public tap or fountain	5	Per 20 liter jerrican
5	Earth	lockable ga	Tap in the compound	10	Included in Rent
35	Earth	lockable ga	Tap in neig At the land	5	Included in Rent
10	Earth	lockable ga	Public tap or fountain	10	Per 20 liter jerrican
16	Cement	lockable ga	Tap inside the house	0	Monthly
32	Cement	lockable ga	Tap in the compound	2	Included in Rent

21	Earth	lockable ga	Tap in the compound		4	Included in Rent
5	Earth	lockable ga	Public tap or fountain		10	Per 20 liter jerrican
17	Earth	lockable ga	Tap in neig	At the land	2	Included in Rent
9	Earth	lockable ga	Other Improved sourc		2	Per 20 liter jerrican
17	Cement	lockable ga	Tap in the compound		3	Monthly
1	Earth	None	Tap inside the house		0	Do not pay
1	Earth	None	Public tap or fountain		1	Per 20 liter jerrican
10	Cement	lockable ga	Public tap or fountain		9	Per 20 liter jerrican
26	Cement	lockable ga	Tap in the compound		5	Included in Rent
73	Cement	lockable ga	Public tap or fountain		10	Included in Rent
6	Earth	lockable ga	Public tap or fountain		1	Per 20 liter jerrican
14	Earth	No gate	Public tap or fountain		3	Per 20 liter jerrican
9	Earth	None	Public tap or fountain		30	Per 20 liter jerrican
1	Earth	None	Borehole		15	Per 20 liter jerrican
5	Earth	No gate	Public tap or fountain		3	Per 20 liter jerrican
8	Grass	None	Tap in the compound		2	Per 20 liter jerrican
8	Earth	No gate	Tap inside the house		0	Per 20 liter jerrican
10	Cement	lockable ga	Tap inside the house		1	Included in Rent
6	Grass	No gate	Public tap or fountain		1	Per 20 liter jerrican
11	Earth	lockable ga	Tap in the compound		5	Included in Rent
45	Cement	lockable ga	Tap in the compound		5	Per 20 liter jerrican
7	Cement	lockable ga	Tap in the compound		1	Included in Rent
16	Cement	lockable ga	Tap in the compound		2	Included in Rent
2	Earth	lockable ga	Public tap or fountain		8	Per 20 liter jerrican
10	Earth	lockable ga	Public tap or fountain		5	Included in Rent
13	Earth	lockable ga	Public tap or fountain		10	Per 20 liter jerrican
1	Cement	lockable ga	Public tap or fountain		10	Per 20 liter jerrican
10	Cement	lockable ga	Public tap or fountain		5	Per 20 liter jerrican
10	Cement	lockable ga	Tap in the compound		4	Included in Rent
4	Earth	lockable ga	Tap in the compound		10	Monthly
2	Earth	None	Public tap or fountain		1	Per 20 liter jerrican
47	Cement	lockable ga	Tap in the compound		2	Included in Rent
14	Cement	No gate	Public tap or fountain		10	Per 20 liter jerrican
32	Earth	lockable ga	Tap in the compound		3	Included in Rent
1	Earth	None	Tap inside the house		5	Included in Rent
54	Earth	lockable ga	Tap in the compound		15	Included in Rent
11	Earth	No gate	Public tap or fountain		10	Per 20 liter jerrican
12	Earth	unlockable	Tap in neig	Tap in the	30	Included in Rent
22	Cement	lockable ga	Tap in the compound		1	Included in Rent
50	Earth	lockable ga	Public tap or fountain		15	Included in Rent
3	Cement	lockable ga	Tap in the compound		5	Included in Rent
10	Cement	lockable ga	Public tap or fountain		20	Per 20 liter jerrican
31	Cement	lockable ga	Tap in the compound		5	Included in Rent
85	Earth	lockable ga	Tap in the compound		5	Included in Rent
8	Grass	No gate	Tap in the compound		1	Per 20 liter jerrican
1	Earth	None	Tap inside the house		0	Do not pay
14	Earth	lockable ga	Tap in the compound		5	Included in Rent
1	Cement	None	Public tap or fountain		10	Per 20 liter jerrican
27	Cement	lockable ga	Tap in the compound		1	Monthly

10	Grass		lockable ga	Public tap or fountain	10	Per 20 liter jerrican
4	Earth		lockable ga	Public tap or fountain	10	Per 20 liter jerrican
30	Cement		lockable ga	Tap in the compound	5	Included in Rent
27	Cement		lockable ga	Tap in the compound	4	Monthly
15	Grass		lockable ga	Tap in the compound	2	Monthly
30	Earth		None	Public tap or fountain	4	Per 20 liter jerrican
4	Grass		No gate	Other Improved sourc	1	Per 20 liter jerrican
3	Earth		None	Public tap or fountain	30	Per 20 liter jerrican
1	Grass		lockable ga	Public tap or fountain	5	Per 20 liter jerrican
7	Earth		None	Public tap or fountain	20	Per 20 liter jerrican
1	Cement		lockable ga	Tap inside the house	5	Monthly
4	Cement		lockable ga	Public tap or fountain	5	Per 20 liter jerrican
6	Cement		lockable ga	Tap in the compound	2	Monthly
8	Earth		None	Public tap or fountain	2	Per 20 liter jerrican
1	Cement		None	Public tap or fountain	10	Per 20 liter jerrican
40	Cement		lockable ga	Public tap or fountain	5	Per 20 liter jerrican
20	Earth		lockable ga	Tap in the compound	5	Included in Rent
9	Earth		lockable ga	Tap in the compound	2	Monthly
1	Earth		None	Tap in the compound	10	Per 20 liter jerrican
9	Cement		lockable ga	Public tap or fountain	0	Per 20 liter jerrican
12	Earth		lockable ga	Tap in the compound	10	Included in Rent
1	Earth		None	Public tap or fountain	20	Per 20 liter jerrican
10	Cement	Stones	lockable ga	Tap in the compound	2	Included in Rent
2	Cement		lockable ga	Public tap or fountain	1	Per 20 liter jerrican
16	Grass		lockable ga	Other Improved sourc	2	Per 20 liter jerrican
10	Earth		None	Public tap or fountain	3	Per 20 liter jerrican
1	Earth		None	Tap in the compound	2	Monthly
7	Earth		lockable ga	Tap in the compound	10	Included in Rent
1	Earth		No gate	Public tap or fountain	12	Do not pay
30	Cement		lockable ga	Tap in the compound	2	Monthly
12	Earth		lockable ga	Public tap or fountain	10	Per 20 liter jerrican
5	Earth		lockable ga	Tap inside the house	1	Monthly
50	Earth		lockable ga	Tap in the compound	3	Included in Rent
20	Earth		No gate	Public tap or fountain	0	Per 20 liter jerrican
5	Grass		lockable ga	Tap in the compound	2	Per 20 liter jerrican
9	Earth		lockable ga	Tap in the compound	20	Monthly
7	Grass		lockable ga	Tap in the compound	1	Per 20 liter jerrican
28	Cement		lockable ga	Tap in the compound	5	Monthly
1	Grass		None	Public tap or fountain	6	Per 20 liter jerrican
1	Grass		lockable ga	Other Improved sourc	1	Per 20 liter jerrican
15	Earth		lockable ga	Public tap or fountain	2	Per 20 liter jerrican
1	Grass		None	Public tap or fountain	15	Per 20 liter jerrican
10	Earth		lockable ga	Public tap or fountain	5	Per 20 liter jerrican
13	Earth		No gate	Other Improved sourc	1	Per 20 liter jerrican
4	Earth		None	Public tap or fountain	2	Per 20 liter jerrican
1	Earth		None	Tap in the compound	10	Per 20 liter jerrican
6	Grass		lockable ga	Other Improved sourc	1	Per 20 liter jerrican
3	Grass		None	Tap in the compound	5	Per 20 liter jerrican
3	Earth		lockable ga	Public tap or fountain	5	Per 20 liter jerrican

8	Cement	lockable g	Tap inside the house		2	Monthly
7	Grass	lockable g	Tap in neig	Tap in ano	5	Monthly
1	Earth	lockable g	Public tap or fountain		1	Per 20 liter jerrican
4	Earth	lockable g	Tap in the compound		1	Monthly
14	Earth	lockable g	Public tap or fountain		5	Monthly
5	Grass	unlockable	Public tap or fountain		3	Per 20 liter jerrican
20	Earth	lockable g	Tap in the compound		3	Included in Rent
13	Earth	lockable g	Tap in the compound		2	Included in Rent
6	Cement	lockable g	Public tap or fountain		5	Per 20 liter jerrican
4	Cement	lockable g	Tap in the compound		5	Included in Rent
3	Earth	lockable g	Tap in the compound		2	Monthly
4	Earth	lockable g	Public tap or fountain		10	Per 20 liter jerrican
1	Cement	lockable g	Tap inside the house		0	Monthly
15	Earth	lockable g	Tap in the compound		2	Monthly
30	Cement	lockable g	Tap in the compound		5	Monthly
10	Cement	lockable g	Tap in the compound		2	Monthly
1	Cement	None	Tap inside the house		1	Monthly
15	Grass	None	Public tap or fountain		1	Per 20 liter jerrican
4	Cement	lockable g	Public tap or fountain		15	Per 20 liter jerrican
6	Earth	lockable g	Public tap or fountain		10	Per 20 liter jerrican
2	Grass	No gate	Public tap or fountain		1	Per 20 liter jerrican
1	Earth	None	Tap inside the house		2	Monthly
2	Earth	None	Public tap or fountain		15	Per 20 liter jerrican
5	Earth	None	Public tap or fountain		10	Per 20 liter jerrican
1	Grass	None	Public tap or fountain		10	Per 20 liter jerrican
1	Earth	None	Public tap or fountain		10	Per 20 liter jerrican
1	Earth	lockable g	Public tap or fountain		5	Per 20 liter jerrican
3	Earth	lockable g	Tap in the compound		1	Per 20 liter jerrican
2	Cement	lockable g	Public tap or fountain		10	Per 20 liter jerrican
1	Earth	None	Tap in the compound		3	Monthly
7	Earth	None	Tap in the compound		1	Per 20 liter jerrican
1	Earth	None	Tap inside the house		1	Monthly
6	Earth	lockable g	Public tap or fountain		1	Per 20 liter jerrican
2	Earth	lockable g	Tap inside the house		0	Monthly
2	Earth	No gate	Public tap or fountain		5	Per 20 liter jerrican
5	Cement	lockable g	Tap in the compound		5	Monthly
40	Cement	lockable g	Tap in the compound		2	Included in Rent
2	Grass	None	Public tap or fountain		1	Per 20 liter jerrican
38	Earth	lockable g	Tap in neig	At the land	5	Included in Rent
7	Earth	No gate	Public tap or fountain		1	Per 20 liter jerrican
4	Cement	lockable g	Other unim	Bicycle rid	0	Per 20 liter jerrican
16	Earth	lockable g	Tap inside the house		2	Monthly
6	Cement	lockable g	Tap in the compound		2	Monthly
13	Earth	lockable g	Tap in neig	Tap at the	10	Per 20 liter jerrican
1	Earth	None	Public tap or fountain		10	Per 20 liter jerrican
8	Cement	lockable g	Tap in the compound		1	Monthly
21	Earth	lockable g	Tap in the compound		1	Included in Rent
8	Cement	lockable g	Tap in neig	Tap in ano	90	Monthly
20	Earth	lockable g	Tap in the compound		1	Per 20 liter jerrican

23	Cement	lockable ga	Tap in the compound	5	Monthly
9	Cement	lockable ga	Tap in the compound	0	Monthly
48	Earth	No gate	Public tap or fountain	15	Per 20 liter jerrican
3	Earth	lockable ga	Public tap or fountain	5	Per 20 liter jerrican
1	Earth	None	Public tap or fountain	10	Per 20 liter jerrican
14	Earth	None	Tap inside the house	0	Monthly
1	Earth	None	Tap in the compound	1	Monthly
6	Earth	lockable ga	Public tap or fountain	20	Per 20 liter jerrican
11	Earth	None	Tap in the compound	3	Monthly
27	Cement	lockable ga	Tap in the compound	5	Included in Rent
3	Cement	lockable ga	Public tap or fountain	1	Per 20 liter jerrican
10	Cement	lockable ga	Public tap or fountain	5	Per 20 liter jerrican
9	Earth	lockable ga	Public tap or fountain	5	Per 20 liter jerrican
14	Cement	No gate	Public tap or fountain	20	Per 20 liter jerrican
13	Earth	lockable ga	Tap in neig Tap in the	5	Per 20 liter jerrican
4	Earth	lockable ga	Tap in the compound	1	Per 20 liter jerrican
9	Earth	None	Public tap or fountain	2	Per 20 liter jerrican
15	Earth	No gate	Public tap or fountain	1	Per 20 liter jerrican
6	Earth	None	Public tap or fountain	0	Per 20 liter jerrican
9	Earth	lockable ga	Tap in the compound	1	Monthly
1	Earth	None	Borehole	5	Per 20 liter jerrican
30	Cement	lockable ga	Tap in the compound	5	Included in Rent
18	Cement	lockable ga	Tap in the compound	2	Monthly
15	Earth	lockable ga	Tap in the compound	10	Included in Rent
12	Cement	lockable ga	Tap in the compound	2	Included in Rent
10	Cement	lockable ga	Tap inside the house	0	Monthly
14	Earth	lockable ga	Tap in the compound	5	Included in Rent
6	Grass	None	Public tap or fountain	2	Per 20 liter jerrican
52	Cement	lockable ga	Tap in the compound	2	Per 20 liter jerrican
10	Earth	None	Tap in the compound	5	Per 20 liter jerrican
11	Earth	lockable ga	Public tap or fountain	1	Per 20 liter jerrican
5	Cement	lockable ga	Public tap or fountain	2	Per 20 liter jerrican
1	Earth	No gate	Public tap or fountain	4	Per 20 liter jerrican
6	Cement	lockable ga	Public tap or fountain	15	Per 20 liter jerrican
31	Cement	lockable ga	Tap in the compound	3	Included in Rent
12	Cement	lockable ga	Public tap or fountain	15	Per 20 liter jerrican
8	Cement	lockable ga	Other Improved sourc	1	Per 20 liter jerrican
3	Cement	lockable ga	Tap in neig At the land	5	Included in Rent
25	Cement	lockable ga	Tap in the compound	5	Included in Rent
5	Earth	lockable ga	Other Improved sourc	1	Per 20 liter jerrican
87	Earth	lockable ga	Tap in the compound	60	Included in Rent
1	Earth	lockable ga	Tap in the compound	10	Monthly
100	Cement	lockable ga	Tap in the compound	2	Monthly
12	Grass	lockable ga	Tap inside the house	0	Monthly
5	Earth	lockable ga	Public tap or fountain	1	Per 20 liter jerrican
8	Earth	No gate	Tap in the compound	1	Per 20 liter jerrican
10	Grass	lockable ga	Tap in the compound	2	Included in Rent
1	Cement	lockable ga	Tap inside the house	10	Monthly
8	Cement	lockable ga	Tap in the compound	1	Monthly

42	Earth	lockable ga	Tap in the compound		10	Monthly
11	Cement	lockable ga	Tap in the compound		1	Per 20 liter jerrican
1	Cement	lockable ga	Borehole		2	Per 20 liter jerrican
4	Grass	None	Public tap or fountain		5	Per 20 liter jerrican
15	Earth	lockable ga	Tap in the compound		1	Per 20 liter jerrican
12	Earth	None	Public tap or fountain		2	Per 20 liter jerrican
91	Earth	lockable ga	Tap in the compound		5	Included in Rent
17	Cement	lockable ga	Tap inside the house		0	Monthly
10	Earth	lockable ga	Tap in the compound		1	Per 20 liter jerrican
39	Earth	lockable ga	Tap in the compound		10	Monthly
13	Cement	lockable ga	Tap in the compound		5	Included in Rent
18	Cement	lockable ga	Public tap or fountain		0	Per 20 liter jerrican
13	Earth	lockable ga	Tap in the compound		1	Included in Rent
30	Cement	lockable ga	Tap inside the house		1	Monthly
8	Earth	None	Public tap or fountain		3	Per 20 liter jerrican
1	Earth	lockable ga	Tap in neig	Tap in ano	8	Included in Rent
17	Cement	unlockable	Public tap or fountain		5	Per 20 liter jerrican
2	Earth	unlockable	Borehole		10	Per 20 liter jerrican
10	Earth	None	Tap in the compound		2	Monthly
10	Earth	No gate	Public tap or fountain		3	Per 20 liter jerrican
8	Earth	lockable ga	Tap in the compound		3	Included in Rent
5	Cement	lockable ga	Public tap or fountain		1	Monthly
1	Earth	None	Tap in the compound		2	Do not pay
9	Cement	lockable ga	Tap inside the house		1	Included in Rent
4	Cement	lockable ga	Tap in neig	Tap in ano	5	Included in Rent
24	Cement	lockable ga	Tap in the compound		10	Monthly
1	Earth	None	Tap in the compound		10	Per 20 liter jerrican
100	Cement	lockable ga	Tap in the compound		2	Per 20 liter jerrican
13	Cement	lockable ga	Tap in the compound		5	Included in Rent
9	Earth	lockable ga	Tap in the compound		30	Monthly
43	Earth	lockable ga	Tap in neig	The landlo	1	Per 20 liter jerrican
8	Earth	lockable ga	Tap in the compound		3	Included in Rent
1	Earth	lockable ga	Tap in the compound		2	Monthly
49	Cement	lockable ga	Tap in the compound		5	Included in Rent
1	Grass	unlockable	Tap in the compound		1	Monthly
3	Earth	No gate	Public tap or fountain		10	Per 20 liter jerrican
9	Cement	lockable ga	Tap in the compound		1	Monthly
8	Earth	lockable ga	Public tap or fountain		10	Included in Rent
1	Earth	None	Tap in the compound		5	Per 20 liter jerrican
10	Earth	lockable ga	Tap in the compound		5	Per 20 liter jerrican
13	Cement	lockable ga	Tap in the compound		2	Monthly
1	Cement	lockable ga	Tap inside the house		10	Monthly
12	Earth	lockable ga	Public tap or fountain		2	Per 20 liter jerrican
5	Cement	lockable ga	Tap in the compound		3	Monthly
60	Cement	lockable ga	Tap in the compound		2	Included in Rent
4	Earth	lockable ga	Public tap or fountain		20	Per 20 liter jerrican
1	Earth	lockable ga	Tap in the compound		1	Monthly
6	Earth	lockable ga	Tap in the compound		2	Per 20 liter jerrican
11	Cement	lockable ga	Tap in the compound		3	Included in Rent

20	Earth		lockable ga	Tap in the compound	2	Included in Rent	
50	Grass		lockable ga	Tap in the compound	30	Included in Rent	
19	Earth		lockable ga	Tap in the compound	2	Included in Rent	
28	Cement		lockable ga	Tap in the compound	2	Included in Rent	
1	Earth		None	Public tap or fountain	3	Per 20 liter jerrican	
1	Grass		No gate	Public tap or fountain	6	Per 20 liter jerrican	
11	Earth		unlockable	Public tap or fountain	5	Per 20 liter jerrican	
9	Earth		lockable ga	Public tap or fountain	1	Per 20 liter jerrican	
11	Earth		lockable ga	Tap in the compound	4	Included in Rent	
3	Earth		lockable ga	Tap in the compound	2	Monthly	
1	Earth		lockable ga	Tap in the compound	1	Monthly	
4	Earth		No gate	Tap in the compound	1	Per 20 liter jerrican	
6	Cement		lockable ga	Tap in the compound	2	Monthly	
6	Cement		lockable ga	Tap in the compound	5	Monthly	
7	Earth		lockable ga	Public tap or fountain	5	Per 20 liter jerrican	
6	Earth		No gate	Public tap or fountain	1	Per 20 liter jerrican	
9	Cement		lockable ga	Public tap or fountain	20	Per 20 liter jerrican	
8	Cement		lockable ga	Public tap or fountain	5	Per 20 liter jerrican	
32	Cement		lockable ga	Tap in the compound	15	Monthly	
7	Earth		lockable ga	Tap in the compound	1	Per 20 liter jerrican	
10	Earth		lockable ga	Public tap or fountain	10	Per 20 liter jerrican	
35	Cement		lockable ga	Tap in the compound	5	Included in Rent	
8	Earth		lockable ga	Public tap or fountain	20	Per 20 liter jerrican	
4	Earth		No gate	Tap in the compound	2	Monthly	
9	Cement		lockable ga	Tap in the compound	3	Included in Rent	
39	Cement		lockable ga	Tap in the compound	5	Monthly	
60	Cement		lockable ga	Tap in the compound	10	Included in Rent	
3	Earth		unlockable	Tap in the compound	0	Per 20 liter jerrican	
12	Earth		lockable ga	Tap in the compound	5	Per 20 liter jerrican	
13	Earth		lockable ga	Public tap or fountain	5	Per 20 liter jerrican	
3	Earth		None	Public tap or fountain	3	Per 20 liter jerrican	
5	Earth		None	Public tap or fountain	2	Per 20 liter jerrican	
35	Cement		lockable ga	Tap in the compound	5	Included in Rent	
6	Earth		lockable ga	Public tap or fountain	5	Monthly	
2	Earth		No gate	Public tap or fountain	20	Per 20 liter jerrican	
1	Earth		lockable ga	Tap in the compound	2	Monthly	
16	Earth		lockable ga	Tap in the compound	2	Included in Rent	
60	Cement		lockable ga	Tap in the compound	2	Included in Rent	
63	Cement	Stones	lockable ga	Tap in the compound	5	Per 20 liter jerrican	
9	Cement		lockable ga	Tap in the compound	2	Included in Rent	
8	Cement		lockable ga	Public tap or fountain	10	Per 20 liter jerrican	
1	Grass		No gate	Other Improved sourc	3	Per 20 liter jerrican	
20	Cement		None	Public tap or fountain	10	Per 20 liter jerrican	
5	Earth		unlockable	Public tap or fountain	2	Per 20 liter jerrican	
27	Cement		lockable ga	Tap in the compound	3	Monthly	
1	Earth		lockable ga	Borehole	2	Do not pay	Pay electri
25	Cement		lockable ga	Tap in the compound	5	Per 20 liter jerrican	
13	Cement		lockable ga	Public tap or fountain	3	Per 20 liter jerrican	
8	Earth		lockable ga	Tap in the compound	5	Monthly	

1	Grass		None	Public tap or fountain		5	Per 20 liter jerrican
5	Earth		None	Public tap or fountain		5	Per 20 liter jerrican
35	Earth		lockable ga	Tap in neig	They get w	2	Included in Rent
12	Cement		lockable ga	Tap in the compound		30	Included in Rent
21	Earth		lockable ga	Tap in the compound		5	Per 20 liter jerrican
1	Earth		lockable ga	Tap in neig	Tap in the	10	Per 20 liter jerrican
7	Cement		lockable ga	Public tap or fountain		5	Per 20 liter jerrican
66	Cement		lockable ga	Tap in the compound		20	Included in Rent
20	Cement		lockable ga	Tap in the compound		5	Monthly
8	Earth		lockable ga	Tap in neig	Tap in the	5	Included in Rent
18	Cement		lockable ga	Tap in the compound		3	Included in Rent
5	Cement		lockable ga	Public tap or fountain		5	Per 20 liter jerrican
30	Cement		lockable ga	Tap in the compound		2	Included in Rent
8	Cement		lockable ga	Tap in the compound		3	Monthly
6	Cement		lockable ga	Tap inside the house		0	Included in Rent
36	Cement		lockable ga	Tap in the compound		60	Per 20 liter jerrican
24	Earth		lockable ga	Public tap or fountain		10	Included in Rent
5	Cement		lockable ga	Public tap or fountain		10	Per 20 liter jerrican
4	Earth		lockable ga	Public tap or fountain		2	Per 20 liter jerrican
14	Earth		None	Other Improved sourc		1	Per 20 liter jerrican
10	Earth		No gate	Public tap or fountain		1	Per 20 liter jerrican
28	Cement		lockable ga	Tap in the compound		2	Included in Rent
3	Cement		lockable ga	Public tap or fountain		1	Per 20 liter jerrican
4	Cement		lockable ga	Tap in the compound		3	Included in Rent
4	Cement		lockable ga	Tap in the compound		5	Per 20 liter jerrican
4	Earth		None	Public tap or fountain		5	Per 20 liter jerrican
1	Earth		None	Public tap or fountain		5	Per 20 liter jerrican
9	Cement		lockable ga	Public tap or fountain		2	Per 20 liter jerrican
55	Cement		lockable ga	Tap in the compound		5	Included in Rent
8	Cement		lockable ga	Tap in the compound		3	Included in Rent
8	Grass		None	Tap in the compound		1	Per 20 liter jerrican
50	Cement		lockable ga	Tap in the compound		5	Monthly
35	Earth		lockable ga	Tap in the compound		3	Included in Rent
1	Earth		None	Public tap or fountain		2	Per 20 liter jerrican
12	Cement		No gate	Public tap or fountain		1	Per 20 liter jerrican
7	Cement		lockable ga	Tap in the compound		2	Included in Rent
25	Cement		lockable ga	Tap inside the house		1	Included in Rent
26	Grass		lockable ga	Tap in the compound		1	Included in Rent
6	Earth		No gate	Tap inside the house		1	Per 20 liter jerrican
7	Cement	both grass	lockable ga	Tap in the compound		10	Per 20 liter jerrican
8	Earth		lockable ga	Tap in the compound		1	Monthly
20	Grass		No gate	Tap in the compound		3	Monthly
5	Earth		None	Public tap or fountain		2	Per 20 liter jerrican
15	Cement		lockable ga	Tap in the compound		10	Monthly
41	Earth		lockable ga	Tap in neig	Tap water	2	Per 20 liter jerrican
10	Cement		lockable ga	Borehole		10	Per 20 liter jerrican
1	Earth		None	Public tap or fountain		5	Per 20 liter jerrican
5	Earth		None	Public tap or fountain		2	Per 20 liter jerrican
5	Cement		lockable ga	Public tap or fountain		2	Per 20 liter jerrican

28	Cement		lockable ga	Tap in the compound		5	Included in Rent
4	Cement		lockable ga	Public tap or fountain		5	Included in Rent
34	Earth		No gate	Tap in the compound		5	Included in Rent
13	Earth		lockable ga	Tap in the compound		5	Per 20 liter jerrican
11	Earth		lockable ga	Tap in the compound		1	Per 20 liter jerrican
7	Cement		lockable ga	Public tap or fountain		1	Per 20 liter jerrican
44	Cement		No gate	Tap in the compound		1	Per 20 liter jerrican
2	Earth		lockable ga	Public tap or fountain		1	Per 20 liter jerrican
8	Earth		lockable ga	Tap in the compound		2	Per 20 liter jerrican
24	Cement		lockable ga	Public tap or fountain		5	Per 20 liter jerrican
2	Earth		lockable ga	Public tap or fountain		3	Per 20 liter jerrican
66	Cement		lockable ga	Tap in the compound		3	Included in Rent
1	Cement		lockable ga	Tap inside the house		2	Monthly
20	Cement		lockable ga	Tap in the compound		2	Included in Rent
10	Cement	on the corr	lockable ga	Tap in the compound		5	Included in Rent
10	Cement		lockable ga	Public tap or fountain		20	Per 20 liter jerrican
5	Cement		lockable ga	Public tap or fountain		15	Per 20 liter jerrican
25	Earth		No gate	Other Improved sourc		1	Per 20 liter jerrican
17	Cement		lockable ga	Tap in the compound		10	Included in Rent
1	Grass		None	Tap in the compound		1	Monthly
36	Earth		lockable ga	Tap in neig	At the land	6	Included in Rent
1	Cement		lockable ga	Tap in the compound		5	Monthly
3	Earth		lockable ga	Tap in the compound		1	Monthly
9	Earth		lockable ga	Tap in the compound		2	Per 20 liter jerrican
16	Cement		lockable ga	Tap in the compound		3	Monthly
30	Earth		lockable ga	Tap in the compound		10	Monthly
1	Earth		lockable ga	Other Improved sourc		1	Included in Rent
4	Cement		lockable ga	Public tap or fountain		5	Per 20 liter jerrican
28	Earth		lockable ga	Tap in the compound		1	Included in Rent
8	Earth		lockable ga	Tap in the compound		1	Monthly
18	Cement		lockable ga	Tap in the compound		30	Per 20 liter jerrican
4	Earth		None	Public tap or fountain		10	Per 20 liter jerrican
4	Earth		None	Public tap or fountain		5	Per 20 liter jerrican
1	Earth		None	Tap in the compound		10	Per 20 liter jerrican
14	Earth		unlockable	Tap in the compound		3	Monthly
12	Earth		unlockable	Public tap or fountain		2	Per 20 liter jerrican
4	Earth		No gate	Public tap or fountain		2	Per 20 liter jerrican
10	Earth		lockable ga	Tap in the compound		2	Included in Rent
11	Earth		None	Public tap or fountain		5	Per 20 liter jerrican
8	Earth		lockable ga	Tap in the compound		5	Monthly
7	Cement		lockable ga	Tap in the compound		2	Included in Rent
65	Cement		lockable ga	Tap in the compound		10	Monthly
3	Cement		lockable ga	Public tap or fountain		5	Per 20 liter jerrican
8	Cement		lockable ga	Tap in the compound		2	Monthly
26	Cement		lockable ga	Tap in the compound		2	Per 20 liter jerrican
12	Grass		lockable ga	Tap in neig	Water tap	5	Per 20 liter jerrican
1	Earth		None	Tap in the compound		10	Per 20 liter jerrican
20	Earth		lockable ga	Public tap or fountain		10	Per 20 liter jerrican
8	Earth		lockable ga	Tap in the compound		1	Per 20 liter jerrican

8	Earth	None	Public tap or fountain	5	Per 20 liter jerrican
1	Earth	None	Public tap or fountain	5	Per 20 liter jerrican
13	Earth	No gate	Public tap or fountain	10	Per 20 liter jerrican
20	Earth	lockable ga	Tap in the compound	2	Do not pay
23	Cement	lockable ga	Tap in the compound	5	Monthly
4	Grass	lockable ga	Public tap or fountain	10	Per 20 liter jerrican
3	Earth	lockable ga	Tap in the compound	2	Monthly
24	Earth	lockable ga	Tap in the compound	5	Per 20 liter jerrican
11	Earth	No gate	Public tap or fountain	1	Per 20 liter jerrican
15	Earth	lockable ga	Tap in the compound	0	Per 20 liter jerrican
6	Earth	None	Other unim Water vend	0	Per 20 liter jerrican
8	Cement	lockable ga	Public tap or fountain	30	Per 20 liter jerrican
19	Earth	lockable ga	Public tap or fountain	2	Per 20 liter jerrican
12	Earth	lockable ga	Public tap or fountain	5	Per 20 liter jerrican
2	Earth	lockable ga	Tap in the compound	1	Per 20 liter jerrican
3	Earth	No gate	Public tap or fountain	1	Per 20 liter jerrican
1	Earth	None	Borehole	15	Per 20 liter jerrican
1	Cement	lockable ga	Tap in the compound	2	Monthly
3	Earth	unlockable	Public tap or fountain	10	Per 20 liter jerrican
10	Earth	lockable ga	Tap in the compound	10	Included in Rent
15	Earth	lockable ga	Tap in the compound	4	Monthly
35	Cement	lockable ga	Tap in the compound	3	Included in Rent
1	Earth	lockable ga	Public tap or fountain	5	Per 20 liter jerrican
1	Cement	lockable ga	Tap inside the house	2	Monthly
2	Cement	lockable ga	Public tap or fountain	5	Per 20 liter jerrican
9	Earth	No gate	Tap in the compound	2	Monthly
15	Cement	lockable ga	Tap in the compound	2	Included in Rent
5	Earth	lockable ga	Public tap or fountain	1	Per 20 liter jerrican
21	Cement	lockable ga	Tap in the compound	10	Included in Rent
3	Earth	lockable ga	Public tap or fountain	10	Per 20 liter jerrican
7	Cement	lockable ga	Public tap or fountain	20	Per 20 liter jerrican
13	Earth	lockable ga	Public tap or fountain	1	Per 20 liter jerrican
12	Cement	lockable ga	Public tap or fountain	1	Per 20 liter jerrican
8	Earth	lockable ga	Tap in the compound	2	Monthly
9	Grass	unlockable	Borehole	2	Per 20 liter jerrican
10	Earth	lockable ga	Tap in the compound	2	Monthly
8	Cement	lockable ga	Tap in the compound	1	Per 20 liter jerrican
40	Cement	lockable ga	Tap in the compound	5	Included in Rent
10	Earth	lockable ga	Public tap or fountain	5	Per 20 liter jerrican
6	Grass	None	Borehole	10	Per 20 liter jerrican
45	Cement	lockable ga	Tap in the compound	10	Included in Rent
10	Grass	None	Tap in the compound	1	Per 20 liter jerrican
4	Earth	lockable ga	Other Improved sourc	1	Per 20 liter jerrican
21	Cement	lockable ga	Tap in the compound	3	Monthly
3	Grass	None	Borehole	1	Per 20 liter jerrican
6	Earth	None	Tap in the compound	1	Monthly
4	Cement	lockable ga	Tap inside the house	0	Monthly
3	Earth	lockable ga	Tap inside the house	0	Monthly
10	Earth	lockable ga	Tap in the compound	2	Included in Rent

4	Earth		lockable ga	Public tap or fountain		10	Per 20 liter jerrican
47	Cement		lockable ga	Tap in the compound		3	Included in Rent
3	Earth		No gate	Public tap or fountain		1	Per 20 liter jerrican
14	Cement		lockable ga	Tap in the compound		2	Per 20 liter jerrican
1	Earth		lockable ga	Tap in the compound		5	Monthly
1	Earth		lockable ga	Public tap or fountain		10	Per 20 liter jerrican
18	Earth		lockable ga	Tap in the compound		5	Included in Rent
2	Cement		lockable ga	Public tap or fountain		10	Per 20 liter jerrican
2	Cement		lockable ga	Tap in the compound		2	Per 20 liter jerrican
8	Earth		None	Public tap or fountain		5	Per 20 liter jerrican
16	Cement		lockable ga	Tap in the compound		5	Included in Rent
23	Cement		lockable ga	Tap in the compound		5	Included in Rent
43	Cement	Sand	lockable ga	Tap in neig	At the land	3	Per 20 liter jerrican
1	Earth		None	Borehole		15	Per 20 liter jerrican
11	Earth		lockable ga	Public tap or fountain		10	Per 20 liter jerrican
11	Cement		lockable ga	Tap in the compound		1	Monthly
22	Earth		lockable ga	Tap in the compound		10	Included in Rent
10	Earth		No gate	Public tap or fountain		15	Per 20 liter jerrican
10	Earth		lockable ga	Tap in the compound		2	Monthly
30	Cement		lockable ga	Tap in the compound		5	Per 20 liter jerrican
4	Grass		lockable ga	Tap inside the house		1	Monthly
4	Earth		lockable ga	Tap in neig	Tap in ano	5	Included in Rent
7	Cement		lockable ga	Public tap or fountain		30	Per 20 liter jerrican
25	Earth		lockable ga	Tap in the compound		2	Included in Rent
8	Earth		None	Public tap or fountain		5	Per 20 liter jerrican
9	Earth		lockable ga	Public tap or fountain		5	Per 20 liter jerrican
11	Cement		lockable ga	Tap in the compound		3	Monthly
4	Earth		None	Public tap or fountain		2	Per 20 liter jerrican
10	Earth		None	Public tap or fountain		5	Per 20 liter jerrican
5	Cement		lockable ga	Public tap or fountain		25	Per 20 liter jerrican
86	Cement		lockable ga	Tap in the compound		2	Included in Rent
1	Earth		None	Public tap or fountain		1	Per 20 liter jerrican
60	Cement		lockable ga	Tap in the compound		5	Monthly
20	Earth		lockable ga	Tap inside the house		1	Monthly
1	Earth		None	Borehole		2	Per 20 liter jerrican
20	Cement		lockable ga	Tap in the compound		1	Per 20 liter jerrican
1	Earth		No gate	Tap in neig	Tap in the	30	Per 20 liter jerrican
3	Cement		lockable ga	Tap in the compound		5	Per 20 liter jerrican
4	Earth		lockable ga	Public tap or fountain		10	Per 20 liter jerrican
5	Earth		lockable ga	Tap in the compound		10	Do not pay
1	Earth		None	Public tap or fountain		10	Per 20 liter jerrican
9	Cement		unlockable	Public tap or fountain		2	Per 20 liter jerrican
4	Cement		lockable ga	Public tap or fountain		2	Per 20 liter jerrican
12	Earth		None	Public tap or fountain		4	Per 20 liter jerrican
1	Earth		lockable ga	Tap inside the house		1	Monthly
10	Earth		No gate	Tap in the compound		1	Per 20 liter jerrican
30	Cement		lockable ga	Tap in the compound		1	Monthly
10	Earth		lockable ga	Public tap or fountain		1	Per 20 liter jerrican
30	Earth		lockable ga	Tap in the compound		4	Per 20 liter jerrican

4	Cement	lockable ga	Tap in the compound		2	Included in Rent
1	Earth	None	Public tap or fountain		5	Per 20 liter jerrican
20	Grass	None	Tap in the compound		1	Monthly
1	Cement	lockable ga	Public tap or fountain		10	Per 20 liter jerrican
1	Earth	None	Public tap or fountain		15	Per 20 liter jerrican
27	Earth	lockable ga	Tap in neig	At the land	2	Per 20 liter jerrican
1	Grass	None	Public tap or fountain		2	Per 20 liter jerrican
6	Earth	No gate	Public tap or fountain		20	Per 20 liter jerrican
25	Cement	lockable ga	Tap in neig	Tap in the	10	Included in Rent
2	Earth	unlockable	Public tap or fountain		10	Per 20 liter jerrican
28	Cement	lockable ga	Tap in the compound		10	Monthly
6	Earth	lockable ga	Public tap or fountain		5	Per 20 liter jerrican
9	Cement	lockable ga	Tap in the compound		1	Monthly
10	Cement	lockable ga	Tap in the compound		2	Included in Rent
13	Earth	lockable ga	Tap in the compound		1	Included in Rent
9	Earth	lockable ga	Other Improved sourc		1	Per 20 liter jerrican
1	Earth	None	Borehole		3	Per 20 liter jerrican
10	Earth	No gate	Public tap or fountain		1	Per 20 liter jerrican
8	Cement	lockable ga	Public tap or fountain		5	Per 20 liter jerrican
1	Earth	lockable ga	Public tap or fountain		10	Per 20 liter jerrican
21	Earth	lockable ga	Tap inside the house		5	Monthly
20	Earth	unlockable	Other unim	Water venc	0	Per 20 liter jerrican
30	Cement	lockable ga	Tap in the compound		2	Included in Rent
1	Earth	None	Public tap or fountain		15	Per 20 liter jerrican
5	Earth	lockable ga	Tap in neig	Tap in ano	5	Included in Rent
21	Earth	lockable ga	Tap in the compound		1	Monthly
32	Earth	None	Tap in neig	At the labd	5	Per 20 liter jerrican
9	Earth	lockable ga	Tap in the compound		3	Included in Rent
5	Earth	lockable ga	Public tap or fountain		1	Per 20 liter jerrican
9	Earth	lockable ga	Tap in neig	Tap water	30	Per 20 liter jerrican
14	Grass	No gate	Public tap or fountain		1	Per 20 liter jerrican
2	Cement	lockable ga	Public tap or fountain		5	Per 20 liter jerrican
6	Cement	lockable ga	Public tap or fountain		10	Per 20 liter jerrican
4	Earth	No gate	Public tap or fountain		1	Per 20 liter jerrican
8	Earth	lockable ga	Public tap or fountain		5	Per 20 liter jerrican
13	Earth	lockable ga	Public tap or fountain		5	Per 20 liter jerrican
1	Earth	None	Tap in the compound		10	Per 20 liter jerrican
7	Earth	lockable ga	Tap in the compound		5	Included in Rent
8	Grass	None	Public tap or fountain		5	Per 20 liter jerrican
9	Earth	lockable ga	Tap in the compound		5	Included in Rent
15	Cement	lockable ga	Tap in the compound		1	Per 20 liter jerrican
1	Earth	lockable ga	Public tap or fountain		5	Per 20 liter jerrican
6	Earth	lockable ga	Tap in the compound		1	Per 20 liter jerrican
10	Earth	lockable ga	Tap inside the house		5	Per 20 liter jerrican
18	Earth	None	Tap in neig	Tap in the	5	Per 20 liter jerrican
7	Cement	lockable ga	Public tap or fountain		5	Per 20 liter jerrican
1	Earth	None	Tap in the compound		2	Monthly
19	Earth	lockable ga	Tap in the compound		4	Included in Rent
8	Earth	lockable ga	Tap in the compound		3	Monthly

7	Earth	lockable ga	Tap in the compound	2	Monthly
30	Cement	lockable ga	Tap in the compound	2	Per 20 liter jerrican
1	Earth	None	Tap in the compound	5	Do not pay
17	Cement	lockable ga	Tap in the compound	2	Monthly
4	Earth	No gate	Public tap or fountain	20	Per 20 liter jerrican
24	Cement	unlockable	Public tap or fountain	5	Per 20 liter jerrican
7	Earth	lockable ga	Public tap or fountain	10	Per 20 liter jerrican
18	Cement	lockable ga	Tap in the compound	3	Included in Rent
1	Cement	lockable ga	Tap in the compound	2	Monthly
11	Grass	lockable ga	Tap in the compound	3	Included in Rent
6	Cement	lockable ga	Public tap or fountain	20	Per 20 liter jerrican
8	Earth	lockable ga	Public tap or fountain	5	Per 20 liter jerrican
8	Earth	lockable ga	Tap in the compound	1	Monthly
1	Earth	lockable ga	Tap in the compound	5	Monthly
6	Grass	None	Public tap or fountain	3	Per 20 liter jerrican
6	Cement	unlockable	Other unimproved sou	10	Included in Rent
2	Earth	lockable ga	Tap in the compound	2	Per 20 liter jerrican
9	Cement	lockable ga	Public tap or fountain	1	Per 20 liter jerrican
1	Earth	None	Borehole	10	Per 20 liter jerrican
7	Earth	unlockable	Tap in the compound	3	Included in Rent
6	Cement	lockable ga	Tap in the compound	2	Included in Rent
1	Earth	lockable ga	Public tap or fountain	8	Per 20 liter jerrican
18	Earth	No gate	Public tap or fountain	1	Per 20 liter jerrican
8	Grass	lockable ga	Tap inside the house	0	Monthly
1	Earth	None	Borehole	5	Per 20 liter jerrican
6	Earth	lockable ga	Public tap or fountain	1	Per 20 liter jerrican
6	Earth	None	Public tap or fountain	5	Per 20 liter jerrican
8	Earth	lockable ga	Tap in the compound	5	Monthly
54	Cement	lockable ga	Tap in the compound	10	Included in Rent
7	Earth	lockable ga	Tap in the compound	1	Monthly
1	Cement	lockable ga	Borehole	3	Monthly
13	Cement	lockable ga	Tap in the compound	1	Per 20 liter jerrican
10	Cement	lockable ga	Public tap or fountain	20	Per 20 liter jerrican
7	Grass	unlockable	Tap in the compound	5	Included in Rent
6	Earth	lockable ga	Public tap or fountain	1	Per 20 liter jerrican
16	Cement	lockable ga	Tap in the compound	5	Included in Rent
6	Earth	None	Public tap or fountain	5	Per 20 liter jerrican
16	Earth	lockable ga	Tap in the compound	2	Per 20 liter jerrican
20	Cement	lockable ga	Tap in the compound	2	Do not pay
7	Earth	lockable ga	Tap in the compound	10	Monthly
10	Earth	lockable ga	Public tap or fountain	5	Per 20 liter jerrican
2	Grass	None	Public tap or fountain	10	Per 20 liter jerrican
25	Earth	lockable ga	Tap in the compound	2	Monthly
6	Grass	unlockable	Public tap or fountain	5	Per 20 liter jerrican
4	Earth	lockable ga	Public tap or fountain	1	Per 20 liter jerrican
60	Cement	lockable ga	Tap in the compound	2	Included in Rent
9	Cement	No gate	Public tap or fountain	2	Per 20 liter jerrican
48	Cement	lockable ga	Tap in the compound	30	Included in Rent
10	Cement	lockable ga	Tap in the compound	1	Included in Rent

9	Grass	No gate	Tap in the compound		1	Per 20 liter jerrican
9	Earth	lockable ga	Public tap or fountain		10	Per 20 liter jerrican
4	Grass	lockable ga	Public tap or fountain		5	Per 20 liter jerrican
13	Earth	lockable ga	Tap in the compound		2	Per 20 liter jerrican
1	Earth	lockable ga	Tap in the compound		1	Per 20 liter jerrican
4	Earth	None	Public tap or fountain		8	Per 20 liter jerrican
12	Earth	No gate	Tap in the compound		5	Included in Rent
5	Cement	lockable ga	Public tap or fountain		2	Per 20 liter jerrican
39	Cement	lockable ga	Tap in the compound		2	Per 20 liter jerrican
100	Earth	lockable ga	Tap in the compound		5	Included in Rent
1	Earth	None	Public tap or fountain		3	Per 20 liter jerrican
9	Earth	lockable ga	Public tap or fountain		0	Per 20 liter jerrican
30	Cement	lockable ga	Tap in the compound		10	Monthly
22	Cement	lockable ga	Tap in the compound		5	Included in Rent
10	Cement	lockable ga	Borehole		5	Per 20 liter jerrican
8	Earth	No gate	Tap in the compound		1	Per 20 liter jerrican
3	Earth	lockable ga	Other unim	Surface wa	5	Included in Rent
11	Cement	lockable ga	Tap in neig	Tap at the	5	Per 20 liter jerrican
5	Earth	lockable ga	Tap in neig	Tap in ano	3	Included in Rent
50	Earth	lockable ga	Tap in the compound		30	Included in Rent
2	Earth	unlockable	Tap in the compound		1	Per 20 liter jerrican
2	Cement	lockable ga	Tap in the compound		3	Monthly
13	Earth	lockable ga	Tap in the compound		2	Included in Rent
25	Earth	No gate	Tap in the compound		5	Per 20 liter jerrican
19	Cement	lockable ga	Tap in the compound		5	Per 20 liter jerrican
17	Earth	lockable ga	Tap in the compound		3	Monthly
50	Cement	lockable ga	Tap in the compound		10	Included in Rent
11	Earth	lockable ga	Public tap or fountain		5	Per 20 liter jerrican
9	Earth	lockable ga	Public tap or fountain		10	Per 20 liter jerrican
16	Earth	None	Tap in the compound		1	Per 20 liter jerrican
6	Cement	lockable ga	Public tap or fountain		5	Per 20 liter jerrican
24	Earth	lockable ga	Tap in the compound		3	Included in Rent
6	Earth	lockable ga	Tap in the compound		1	Included in Rent
6	Cement	lockable ga	Public tap or fountain		5	Per 20 liter jerrican
9	Earth	lockable ga	Tap in the compound		2	Included in Rent
13	Earth	None	Public tap or fountain		5	Per 20 liter jerrican
26	Cement	lockable ga	Tap in the compound		2	Monthly
15	Earth	No gate	Tap in the compound		6	Monthly
10	Earth	No gate	Public tap or fountain		2	Per 20 liter jerrican
1	Earth	None	Borehole		6	Per 20 liter jerrican
6	Earth	lockable ga	Tap in the compound		1	Monthly
4	Cement	lockable ga	Public tap or fountain		5	Per 20 liter jerrican
4	Cement	lockable ga	Public tap or fountain		5	Per 20 liter jerrican
35	Earth	lockable ga	Tap in the compound		5	Monthly
17	Grass	lockable ga	Tap in the compound		2	Monthly
1	Earth	No gate	Tap in the compound		1	Monthly
1	Cement	lockable ga	Tap in the compound		2	Monthly
80	Earth	lockable ga	Tap in the compound		5	Included in Rent
18	Cement	lockable ga	Tap in the compound		5	Monthly

12	Cement	lockable ga	Tap inside the house	1	Monthly
14	Cement	lockable ga	Public tap or fountain	5	Per 20 liter jerrican
26	Earth	lockable ga	Tap in the compound	10	Included in Rent
18	Earth	lockable ga	Tap in the compound	2	Monthly
31	Earth	lockable ga	Tap in the compound	2	Included in Rent
7	Earth	lockable ga	Public tap or fountain	10	Per 20 liter jerrican
1	Earth	unlockable	Tap in the compound	5	Monthly
13	Cement	lockable ga	Tap in the compound	5	Included in Rent
2	Cement	lockable ga	Public tap or fountain	5	Per 20 liter jerrican
8	Earth	No gate	Public tap or fountain	1	Per 20 liter jerrican
5	Earth	None	Public tap or fountain	0	Per 20 liter jerrican
3	Earth	No gate	Public tap or fountain	10	Per 20 liter jerrican
6	Cement	lockable ga	Public tap or fountain	10	Per 20 liter jerrican
8	Earth	lockable ga	Public tap or fountain	30	Per 20 liter jerrican
12	Cement	lockable ga	Tap in the compound	2	Included in Rent
7	Grass	No gate	Tap in the compound	1	Per 20 liter jerrican
13	Cement	No gate	Tap in the compound	1	Per 20 liter jerrican
10	Cement	lockable ga	Tap in the compound	1	Per 20 liter jerrican
12	Cement	lockable ga	Public tap or fountain	2	Per 20 liter jerrican
1	Earth	None	Public tap or fountain	3	Per 20 liter jerrican
28	Earth	lockable ga	Tap in the compound	2	Per 20 liter jerrican
6	Earth	lockable ga	Tap inside the house	0	Monthly
50	Cement	lockable ga	Tap inside the house	1	Included in Rent
3	Cement	lockable ga	Tap inside the house	1	Monthly
20	Cement	lockable ga	Tap in the compound	2	Included in Rent
2	Cement	lockable ga	Tap in the compound	2	Monthly
80	Cement	lockable ga	Tap inside the house	1	Monthly
7	Cement	lockable ga	Tap in neig Tap in ano	5	Per 20 liter jerrican
25	Cement	lockable ga	Tap in the compound	10	Included in Rent
1	Earth	None	Public tap or fountain	5	Per 20 liter jerrican
10	Grass	lockable ga	Tap in the compound	0	Monthly
21	Cement	lockable ga	Tap in the compound	2	Per 20 liter jerrican
3	Earth	None	Public tap or fountain	15	Per 20 liter jerrican
4	Earth	lockable ga	Tap in the compound	1	Per 20 liter jerrican
1	Earth	None	Borehole	2	Do not pay
6	Cement	lockable ga	Tap in the compound	2	Per 20 liter jerrican
4	Grass	lockable ga	Tap in the compound	2	Included in Rent
7	Cement	lockable ga	Public tap or fountain	5	Per 20 liter jerrican
1	Earth	None	Public tap or fountain	10	Per 20 liter jerrican
2	Earth	lockable ga	Borehole	5	Per 20 liter jerrican
6	Earth	lockable ga	Public tap or fountain	1	Per 20 liter jerrican
17	Cement	lockable ga	Tap in the compound	20	Included in Rent
13	Earth	unlockable	Tap in the compound	2	Monthly
22	Earth	lockable ga	Tap in the compound	1	Per 20 liter jerrican
10	Cement	lockable ga	Tap in the compound	10	Included in Rent
22	Cement	lockable ga	Tap inside the house	0	Monthly
19	Earth	lockable ga	Tap in the compound	2	Included in Rent
2	Cement	lockable ga	Public tap or fountain	5	Per 20 liter jerrican
10	Grass	lockable ga	Tap in the compound	2	Monthly

1	Earth		None	Borehole		10	Per 20 liter jerrican	
70	Cement		lockable ga	Tap in the compound		2	Included in Rent	
15	Earth		lockable ga	Tap in the compound		2	Included in Rent	
10	Earth		lockable ga	Tap in neig	Tap at land	5	Included in Rent	
1	Earth		No gate	Public tap or fountain		2	Monthly	
1	Earth		None	Borehole		10	Per 20 liter jerrican	
9	Earth		No gate	Other unimproved sou		1	Per 20 liter jerrican	
4	Cement		lockable ga	Public tap or fountain		15	Per 20 liter jerrican	
32	Cement		lockable ga	Tap in the compound		2	Per 20 liter jerrican	
1	Earth		lockable ga	Tap in the compound		2	Monthly	
9	Cement		lockable ga	Public tap or fountain		2	Per 20 liter jerrican	
60	Earth		lockable ga	Tap in the compound		5	Included in Rent	
4	Cement		lockable ga	Tap in the compound		5	Monthly	
28	Cement	Stones	lockable ga	Tap in neig	At the land	2	Included in Rent	
6	Earth		unlockable	Tap in the compound		10	Monthly	
27	Cement		lockable ga	Tap in the compound		3	Included in Rent	
13	Cement		lockable ga	Tap in the compound		5	Monthly	
7	Earth		lockable ga	Tap inside the house		2	Monthly	
56	Cement		lockable ga	Tap in the compound		60	Included in Rent	
2	Earth		lockable ga	Public tap or fountain		5	Per 20 liter jerrican	
1	Earth		lockable ga	Tap in the compound		5	Monthly	
15	Grass		lockable ga	Tap in neig	Tap in the	5	Per 20 liter jerrican	
10	Earth		lockable ga	Tap in the compound		2	Included in Rent	
1	Earth		None	Public tap or fountain		15	Per 20 liter jerrican	
24	Earth		lockable ga	Tap in the compound		10	Included in Rent	
17	Cement		lockable ga	Tap in the compound		2	Monthly	
20	Cement		lockable ga	Tap in the compound		1	Included in Rent	
13	Earth		No gate	Public tap or fountain		5	Per 20 liter jerrican	
8	Cement		lockable ga	Tap in the compound		2	Per 20 liter jerrican	
2	Cement		lockable ga	Public tap or fountain		5	Per 20 liter jerrican	
7	Grass		None	Public tap or fountain		5	Per 20 liter jerrican	
8	Earth		lockable ga	Tap in the compound		2	Monthly	
54	Cement		lockable ga	Tap in the compound		3	Included in Rent	
19	Cement		lockable ga	Tap in the compound		1	Included in Rent	
2	Grass		No gate	Public tap or fountain		3	Per 20 liter jerrican	
11	Earth		lockable ga	Tap in neig	Tap in the	20	Included in Rent	
16	Grass		lockable ga	Tap in the compound		1	Per 20 liter jerrican	
2	Earth		None	Public tap or fountain		2	Per 20 liter jerrican	
4	Earth		None	Borehole		1	Per 20 liter jerrican	
3	Cement		lockable ga	Tap in the compound		2	Monthly	
2	Earth		No gate	Other Improved sourc		1	Per 20 liter jerrican	
11	Earth		None	Public tap or fountain		5	Per 20 liter jerrican	
8	Cement		lockable ga	Tap in the compound		2	Included in Rent	
1	Earth		lockable ga	Other unim	Door delive	0	Per 20 liter jerrican	
12	Earth		lockable ga	Public tap or fountain		3	Per 20 liter jerrican	
13	Earth		lockable ga	Tap in the compound		1	Included in Rent	
9	Earth		No gate	Public tap or fountain		5	Per 20 liter jerrican	
2	Earth		None	Other Improved sourc		6	Do not pay	He takes b
1	Earth		None	Public tap or fountain		2	Per 20 liter jerrican	

3	Cement	lockable ga	Public tap or fountain		5	Per 20 liter jerrican
10	Earth	lockable ga	Tap in neig	Tap in the	20	Per 20 liter jerrican
7	Earth	lockable ga	Public tap or fountain		10	Per 20 liter jerrican
1	Earth	None	Public tap or fountain		1	Per 20 liter jerrican
17	Grass	unlockable	Tap in the compound		1	Per 20 liter jerrican
39	Earth	lockable ga	Tap in the compound		0	Monthly
6	Earth	lockable ga	Tap in the compound		5	Per 20 liter jerrican
7	Earth	lockable ga	Tap in neig	Tap in the	10	Included in Rent
6	Cement	unlockable	Public tap or fountain		15	Per 20 liter jerrican
90	Earth	lockable ga	Tap in the compound		5	Included in Rent
1	Cement	lockable ga	Public tap or fountain		30	Per 20 liter jerrican
6	Cement	lockable ga	Tap in the compound		10	Included in Rent
11	Earth	lockable ga	Other Improved sourc		1	Per 20 liter jerrican
30	Grass	lockable ga	Borehole		2	Included in Rent
25	Cement	lockable ga	Tap in the compound		5	Per 20 liter jerrican
30	Cement	lockable ga	Tap in the compound		2	Included in Rent
10	Grass	lockable ga	Public tap or fountain		20	Per 20 liter jerrican
5	Earth	None	Public tap or fountain		5	Per 20 liter jerrican
9	Earth	lockable ga	Tap in the compound		2	Included in Rent
4	Cement	lockable ga	Public tap or fountain		5	Per 20 liter jerrican
30	Earth	lockable ga	Borehole		2	Per 20 liter jerrican
30	Earth	unlockable	Tap in neig	Landlords t	4	Per 20 liter jerrican
60	Cement	lockable ga	Other unim	Buy from w	30	Per 20 liter jerrican
28	Cement	lockable ga	Tap in the compound		10	Included in Rent
25	Earth	lockable ga	Tap in the compound		3	Included in Rent
32	Cement	lockable ga	Tap in neig	At the land	2	Per 20 liter jerrican
18	Cement	lockable ga	Tap in the compound		4	Included in Rent
10	Earth	No gate	Public tap or fountain		10	Per 20 liter jerrican
12	Cement	lockable ga	Tap in the compound		5	Monthly
14	Cement	lockable ga	Public tap or fountain		5	Per 20 liter jerrican
1	Earth	None	Public tap or fountain		10	Per 20 liter jerrican
12	Earth	lockable ga	Tap in the compound		1	Per 20 liter jerrican
12	Earth	lockable ga	Tap in the compound		5	Monthly
12	Cement	lockable ga	Public tap or fountain		2	Included in Rent
6	Earth	None	Public tap or fountain		2	Per 20 liter jerrican
12	Earth	lockable ga	Public tap or fountain		5	Per 20 liter jerrican
5	Earth	None	Public tap or fountain		5	Per 20 liter jerrican
22	Earth	lockable ga	Public tap or fountain		15	Monthly
8	Earth	lockable ga	Public tap or fountain		4	Per 20 liter jerrican
20	Earth	lockable ga	Tap in the compound		5	Included in Rent
6	Earth	unlockable	Public tap or fountain		1	Per 20 liter jerrican
7	Earth	lockable ga	Tap in the compound		5	Included in Rent
8	Cement	lockable ga	Borehole		2	Per 20 liter jerrican
9	Cement	lockable ga	Other Improved sourc		5	Per 20 liter jerrican
18	Earth	None	Public tap or fountain		10	Per 20 liter jerrican
23	Earth	lockable ga	Tap in the compound		5	Per 20 liter jerrican
21	Cement	lockable ga	Tap in the compound		5	Included in Rent
6	Earth	lockable ga	Tap inside the house		0	Monthly
31	Earth	lockable ga	Tap in the compound		3	Included in Rent

3	Earth	lockable ga	Tap inside the house	2	Monthly
1	Earth	None	Public tap or fountain	5	Per 20 liter jerrican
8	Earth	No gate	Tap in the compound	1	Per 20 liter jerrican
2	Grass	lockable ga	Tap inside the house	1	Monthly
10	Cement	lockable ga	Tap in the compound	1	Included in Rent
6	Cement	lockable ga	Public tap or fountain	15	Per 20 liter jerrican
15	Earth	lockable ga	Tap in the compound	5	Per 20 liter jerrican
8	Cement	lockable ga	Tap in the compound	5	Included in Rent
20	Cement	lockable ga	Tap inside the house	1	Monthly
30	Cement	lockable ga	Tap in the compound	2	Included in Rent
8	Grass	No gate	Other Improved sourc	2	Per 20 liter jerrican
1	Earth	None	Public tap or fountain	2	Per 20 liter jerrican
8	Cement	lockable ga	Tap in the compound	18	Per 20 liter jerrican
12	Earth	lockable ga	Tap in the compound	4	Monthly
35	Earth	lockable ga	Public tap or fountain	5	Per 20 liter jerrican
7	Earth	unlockable	Tap in the compound	2	Monthly
2	Earth	lockable ga	Public tap or fountain	5	Per 20 liter jerrican
14	Earth	lockable ga	Public tap or fountain	2	Per 20 liter jerrican
11	Earth	None	Public tap or fountain	5	Per 20 liter jerrican
3	Earth	lockable ga	Public tap or fountain	15	Per 20 liter jerrican
1	Grass	None	Public tap or fountain	10	Per 20 liter jerrican
25	Cement	lockable ga	Tap in the compound	2	Included in Rent
1	Cement	lockable ga	Public tap or fountain	2	Per 20 liter jerrican
8	Earth	lockable ga	Tap in the compound	2	Monthly
18	Cement	lockable ga	Tap in the compound	2	Included in Rent
23	Earth	lockable ga	Tap in the compound	10	Monthly
3	Cement	lockable ga	Public tap or fountain	25	Per 20 liter jerrican
4	Earth	lockable ga	Public tap or fountain	2	Per 20 liter jerrican
3	Cement	lockable ga	Public tap or fountain	5	Per 20 liter jerrican
1	Grass	None	Public tap or fountain	5	Per 20 liter jerrican
39	Earth	None	Public tap or fountain	2	Per 20 liter jerrican
9	Grass	None	Public tap or fountain	3	Per 20 liter jerrican
11	Earth	None	Public tap or fountain	5	Per 20 liter jerrican
6	Grass	None	Public tap or fountain	10	Per 20 liter jerrican
12	Cement	lockable ga	Tap in the compound	5	Included in Rent
8	Cement	lockable ga	Public tap or fountain	5	Per 20 liter jerrican
4	Earth	No gate	Public tap or fountain	20	Per 20 liter jerrican
40	Earth	unlockable	Tap in the compound	3	Included in Rent
1	Cement	lockable ga	Tap inside the house	0	Monthly
22	Cement	lockable ga	Public tap or fountain	5	Per 20 liter jerrican
10	Grass	lockable ga	Tap in the compound	1	Per 20 liter jerrican
20	Earth	No gate	Tap in the compound	1	Per 20 liter jerrican
13	Earth	lockable ga	Tap in the compound	5	Included in Rent
11	Grass	lockable ga	Tap in the compound	2	Per 20 liter jerrican
35	Cement	lockable ga	Tap in the compound	3	Included in Rent
115	Earth	lockable ga	Tap in the compound	3	Per 20 liter jerrican
10	Cement	lockable ga	Tap in the compound	1	Per 20 liter jerrican
28	Cement	lockable ga	Tap in the compound	4	Monthly
10	Grass	No gate	Public tap or fountain	1	Per 20 liter jerrican

12	Earth		lockable ga	Public tap or fountain	5	Per 20 liter jerrican
35	Cement		lockable ga	Tap inside the house	1	Included in Rent
40	Grass		None	Tap in the compound	5	Per 20 liter jerrican
8	Cement		lockable ga	Public tap or fountain	1	Per 20 liter jerrican
20	Cement	some porti	lockable ga	Tap in the compound	2	Included in Rent
30	Earth		unlockable	Tap in the compound	2	Monthly
3	Cement		lockable ga	Tap in the compound	2	Monthly
1	Grass		None	Public tap or fountain	10	Per 20 liter jerrican
16	Cement		lockable ga	Tap in the compound	10	Included in Rent
6	Grass		None	Public tap or fountain	2	Per 20 liter jerrican
1	Cement		lockable ga	Tap inside the house	5	Monthly
1	Earth		None	Public tap or fountain	10	Per 20 liter jerrican
10	Earth		lockable ga	Public tap or fountain	1	Per 20 liter jerrican
2	Earth		No gate	Public tap or fountain	10	Per 20 liter jerrican
1	Earth		None	Public tap or fountain	20	Per 20 liter jerrican
4	Cement		lockable ga	Public tap or fountain	2	Per 20 liter jerrican
35	Earth		lockable ga	Tap in the compound	1	Included in Rent
6	Cement		lockable ga	Tap in the compound	5	Included in Rent
7	Cement		unlockable	Public tap or fountain	5	Per 20 liter jerrican
15	Earth		No gate	Public tap or fountain	1	Per 20 liter jerrican
11	Earth		No gate	Public tap or fountain	1	Per 20 liter jerrican
22	Earth		lockable ga	Tap in neig Tap at the	5	Included in Rent
13	Cement		lockable ga	Tap in the compound	5	Monthly
1	Earth		None	Borehole	4	Per 20 liter jerrican
39	Earth		lockable ga	Tap in the compound	3	Included in Rent

q28b	q28c	q29	q30a	q31a	q32	q33	q34a	q35a
10	2	Sometimes	1 2	3 7	Yes		14	Basin insid
4000	4	Sometimes	1 2 96	7	Yes		1 2 14	Basin insid
5	2	Sometimes	1 2 3 4	3	Yes		1	Customised
5	2	Never	1 2 3	7	Yes		14	Customised
5	5	Sometimes	1 2 3	3 7	Yes		12 14 96	Basin in the
5	7	Sometimes	1 2 3	2	No		1 4	Basin insid
10	4	Sometimes	1 2	7	Yes		4 9 12 14	Basin in the
5	5	Sometimes	1 2	7	Yes		14	Basin insid
10	2	Sometimes	1 2 3	3 7	Yes		14	Basin in the
	8	Sometimes	1 2 4	96	Yes		1 3 4	Customised
5	2	Sometimes	1 2	96	Yes		1 3 4	Basin insid
10	5	Sometimes	1 2	96	Yes		1 3 4	Basin insid
5	5	Sometimes	1	7	Yes		3 4 9 14	Basin in the
0	2	Sometimes	1 2	96	No	The respor	4	Basin insid
5	2	Never	1 2 4	6	Yes		1 4	Customised
10	2	Never	1 2 3 4	3 6	Yes		1 3 4 9 12	Customized
0	4	Sometimes	1 2 4	96	Yes		1 4 9 14	Basin insid
0	5	Never	1 2 3	3	Yes		3 4 9 14	Basin insid
0	3	Never	1 2	3	Yes		1 7 12 14	Customised
5	5	Never	1 2	7	Yes		3 4 9 10 12	Customised
6	10	Sometimes	1 2	96	Yes		1 4 12 96	Customized
5	4	Sometimes	1 2	96	Yes		1 4	Customised
10	6	Sometimes	1 2	10	Yes		1 4 96	Customized
5	3	Never	1 2 4	3	Yes		4 9 14	Basin insid
500	6	Never	1 2	6	Yes		1 2 4 7	Basin insid
10	2	Never	1 2	96	Yes		1 4 9 14	Basin insid
5		Sometimes	1 2	7	Yes		3 4 9 12 14	Basin insid
0	2	Never	1 2	2	Yes		1 4 14	Customised
3	7	Sometimes	1 2	3	Yes		1 2 4	Customised
5	15	Never	1 2 3 4	3	Yes		3 4 9 14	Basin in the
998	5	Sometimes	1 2	3	Yes		1 4 9 12 14	Customised
5	5	Never	1 2 3 4	3	Yes		4 9 14	Customised
340	2	Never	1 2 3 4	96	Yes		1 3 4 12 14	Basin insid
5	5	Never	1 2	7	Yes		3 9 14	Customised
5	5	Never	1 2 3 4	7	Yes		3 4 9 14	Customised
5	5	Never	1 2 3 4	7	Yes		4 6 9 14	Basin in the
10	6	Sometimes	1 2 3 4	96	Yes		1 3 4 9 12	Basin insid
5	10	Never	1 2 3 4	3	Yes		4 9 10 14	Basin in the
10	3	Sometimes	1 2	8	Yes		1 3 5 8 9 1	Customised
0	4	Never	1 2	11	Yes		3 4 6 9 10	Customised
998	4	Sometimes	1 2 4	3	Yes		1 4 9 12 14	Customized
10	3	Sometimes	1 2	7	Yes		3 4 9 14 96	Customised
5	8	Never	1 2 3 4	3	Yes		4 5 9 12 14	Beside the
5	8	Never	1 2 3 4	7	Yes		4 9 10 14	Basin in the
280	5	Sometimes	1 2 3 4 96	96	Yes		1 3 4 14 96	Basin insid
200	5	Sometimes	1 2 3 4	96	Yes		1 3 9 13 14	Basin insid
0	5	Often	1 2 3 4	96	Yes		1 3 4 5 9 1	Customised
5	3	Never	1 2 3 4	3	Yes		4 9 14	Basin insid

5	5	Sometimes	1 2	7	Yes	4 9 14 96	Customized
0	3	Sometimes	1 2	3	Yes	1 4 9	Customised
5	8	Never	1 2 3 4	3	Yes	4 9 14	Basin in the
10	6	Sometimes	1 2	3	Yes	3 4 9 10 96	Basin insid
5	14	Never	1 2 3 4	3	Yes	3 4 9 14	Basin insid
0	3	Never	2 3 4	3 8	Yes	1 2 3 4 5 9	Basin in the
0	6	Sometimes	1 2	3	Yes	1 3 7	Basin insid
5	6	Sometimes	1 2	3	Yes	1 4 9	Basin insid
10	6	Sometimes	1 2	3	Yes	1 4 96	Basin insid
300	4	Never	1 2 4	96	Yes	1 3 4 12 14	Basin insid
5	15	Sometimes	1 2	7	Yes	3 4 9 96	Customised
5	6	Sometimes	1 2	7	Yes	1 4 6 7 14	Basin insid
5	5	Sometimes	1 2	96	Yes	1 2 3 12	Customised
5	6	Sometimes	1 2 3	3	Yes	3 4 9 96	Basin insid
0	5	Never	1 2 3 4	3	Yes	3 4 9 14	Basin in the
1200	8	Sometimes	1 2 3 4	3	Yes	1 3 5 12	Toilet/Kitch
5	6	Never	1 2 3 4	96	Yes	1 2 4 12 14	Basin insid
200	5	Never	1 2 3 4	2	Yes	4 9 14	Basin insid
260	3	Sometimes	1 2 3 4	96	Yes	1 4 12 14	Basin insid
0	6	Often	1 2 3 4	3	Yes	1 3 4 13 14	Customised
5	6	Sometimes	1 2	6 8	Yes	1 4 9 14	Customised
0	3	Sometimes	1 2	3	Yes	1 4 12 14	Customized
5	2	Sometimes	1 2	2	No	1 3 4 14	Basin insid
10	4	Sometimes	1 2	10	Yes	1 4 9 14	Basin insid
5	3	Often	1 2 4	7	Yes	1 4 9 12	Basin insid
0	5	Never	1 2 3	3	Yes	4 6 9 14	Customised
10	6	Sometimes	1 2 3	3 7	Yes	3 4 9 10	Basin in the
350	2	Never	1 2 3 4	96	Yes	1 3 4 6 7 1	Beside the
0	5	Never	1 2 3 4	3	Yes	4 9 14	Basin insid
10	3	Sometimes	1 2	7	Yes	4 9 14	Basin in the
2	5	Sometimes	1 2 3	3 7	Yes	4 9	Basin insid
10	7	Sometimes	1 2	7	Yes	4 9 96	Customised
5	8	Never	1 2 4	7	Yes	4 9 14	Customised
5	5	Sometimes	1 2	8	Yes	1 3 9 12 14	Customised
3	7	Sometimes	1 2	7	Yes	4 9 96	Customised
7	10	Sometimes	1 2	10	Yes	1 2 4 96	Basin insid
5	6	Never	1 2 3	3	Yes	3 4 9 10 96	Customised
5	4	Never	1 2 3 4	7	Yes	14	Basin in the
10	5	Never	1 2	2	Yes	1 2 9 14	Basin insid
10	3	Sometimes	1 2 3	7	Yes	4 9 12 14	Basin insid
5	3	Often	1 2 3	7	Yes	1 2 3 4 12	Basin insid
5000	3	Sometimes	1 2	6 10	Yes	1 3 4	Customised
2	4	Sometimes	1 2	7	Yes	1 4	Basin insid
10	4	Sometimes	1 2 4	7	Yes	4 9 12 96	Basin in the
10	20	Sometimes	1 2	7	Yes	3 9 96	Customised
5	2	Sometimes	1 2 3 4	7	Yes	1 3 4 12 14	Basin insid
10	5	Sometimes	1 2 3	7	Yes	3 4 9 10 14	Customised
5	10	Never	1 2 3	7	Yes	4 9 10 14	Beside the
5	3	Never	1 2 4	6	Yes	1 2 4 9 12	Basin insid

5	5	Never	1 2 4		96	Yes	3 4 7	Customise
5	1	Never	1 2 3	3 7		Yes	3 4 9 14 96	Customise
5	4	Never	1 2		96	Yes	1 9 96	Basin insid
10	3	Sometimes	1 2 3 4		7	Yes	14	Basin insid
5	5	Sometimes	1 2	6 7 10		Yes	4 12 14	Customise
	10	Never	1 2 3 4		7	Yes	3 4 5 9 10	Basin insid
0	3	Never	1 2		3	Yes	1 4 9	Basin insid
10	17	Sometimes	1 2 3		3	Yes	3 4 9 14	Basin insid
5	3	Sometimes	1 2		7	Yes	4 9 14	Basin insid
5	4	Never	1 2 4		7	Yes	4 9 14	Basin in the
5	5	Never	1 2	6 10		Yes	4 9 12	Basin insid
5000	40	Often	1 2		3	Yes	1 2 9 12	Customise
1000	5	Sometimes	1 2 3 4		96	Yes	1 3 4 9 12	Customise
700	6	Never	1 2		7	Yes	4 14	Basin in the
10	3	Sometimes	1 2		10	Yes	1 3 4	Customise
10	2	Sometimes	1 2		7	Yes	4 9	Toilet/Kitch
4	8	Sometimes	1 2		7	Yes	1 2 4 9 10	Basin insid
	7	Never	1 2 4		3	Yes	3 4 9	Toilet/Kitch
30	8	Never	2 3		3	Yes	1 4 12 14	Customise
5	6	Sometimes	1 2 3	3 7		Yes	1 3 4 9 14	Basin in the
5	6	Sometimes	1 2 96		7	Yes	1 4 14	Basin insid
5	3	Sometimes	1 2		7	Yes	4 9 14 96	Customise
5	5	Never	1 2 3 4		3	Yes	3 4 9 14	Customise
400	4	Sometimes	1 2		7	Yes	1 4 12	Beside the
5	10	Never	1 2 3 4		3	Yes	4 9 14	Basin in the
5	6	Never	1 2 4	6 10		Yes	2 4 14	Basin insid
10	8	Sometimes	1 2 4		3	No	1 4	Customise
1000	998	Sometimes	1 2		7	Yes	1 4 9 96	Customize
	5	Sometimes	1 2 4		6	Yes	1 4 9 12 14	Customise
250	5	Sometimes	1 2 3 4 96		96	Yes	1 2 3 4	Toilet/Kitch
5	4	Sometimes	1 2		7	Yes	14 96	Basin in the
0	4	Never	1 2 4		96	Yes	1 2 4 7 12	Basin insid
5	5	Sometimes	1 2 3		3	Yes	1 3 9 14	Basin insid
0	6	Never	1 2 4		96	Yes	1 2 3 4 12	Basin insid
5	4	Never	1 2 3 4		7	Yes	4 9 14	Basin insid
0	2	Sometimes	1 2 3 4		3	Yes	1 3 4 12 14	Basin insid
6	7	Sometimes	1 2		96	Yes	1 4 12 96	Customise
3	4	Sometimes	1 2		7	Yes	3 4 96	Basin insid
5	10	Sometimes	1 2 3		8	Yes	2 3	Customise
5	5	Never	1 2 3 4		7	Yes	3 4 6 9 14	Basin in the
0	5	Never	1 2 3 4		3	Yes	4 9 14	Basin in the
100	10	Sometimes	1 2		3	Yes	1 4 9 14	Toilet/Kitch
5	2	Sometimes	1 2	6 8		Yes	1 4	Customise
10	3	Sometimes	1 2		3	Yes	1 3 14	Basin insid
7	6	Sometimes	1 2 4		7	Yes	1 6 9	Customise
800	10	Sometimes	1 2 3		7	Yes	3 4 9 10 14	Basin insid
5	6	Sometimes	1 2	3 6 7		Yes	1 4 14	Basin insid
5	2	Never	1 2 3		7	Yes	4 9 10 14	Basin insid
	4	Sometimes	1 2 3 4		96	Yes	1 3 4 12 14	Toilet/Kitch

5	3	Never	1 2 4		3	No	When dirty	4 9 14	Basin insid
0	5	Never	1 2 3 4		7	Yes		4 9 14	Basin in the
0	3	Sometimes	1 2 4		96	Yes		1 3 4	Basin insid
0	2	Sometimes	1 2 3 4 96		3	Yes		1 3 4	Basin insid
5	3	Sometimes	1 2 3		96	Yes		1 3 4 9	Basin insid
3	10	Sometimes	1 2 3	3 7		Yes		1 2 3 4 6 7	Basin insid
0	7	Often	1 2		3	Yes		1 3 9 12	Customised
7	3	Sometimes	1 2		10	Yes		1 2 4 5 7 1	Customised
0	1	Never	1 2		96	Yes		1 4	Customised
400	6	Often	1 2 4		3	Yes		1 3 4 12 96	Basin insid
0	6	Often	1 2 3 4	3 7		No		1 4 9 12 14	Basin insid
5	4	Never	1 2 4	6 10		Yes		1 3 4 9 12	Customised
5	3	Sometimes	1 2 3		7	No		3 4 9 10 14	Basin insid
150	4	Sometimes	1 2 3 4		10	Yes		1 4 12	Basin insid
0	2	Never	1 2 3 4		3	Yes		3 4 9 14	Basin insid
10	6	Never	2 3		3	Yes		2 4 6 7 14	Customised
30	3	Sometimes	1 2		7	Yes		2 3 4	Customized
0	8	Never	1 2 4		7	Yes		3 9	Toilet/Kitch
5	5	Never	1 2 3		7	Yes		3 9 14	Basin insid
5	5	Never	1 2 3 4		3	Yes		4 9 14	Basin in the
7	3	Sometimes	1 2		96	Yes		1 3 14 96	Basin insid
5	10	Never	1 2 3 4		3	Yes		4 9 14	Basin in the
3	3	Sometimes	1 2		7	Yes		1 4 14	Basin insid
7	8	Sometimes	1 2		96	Yes		1 4 96	Basin insid
3	7	Sometimes	1		7	Yes		1 4 9 12 96	Customised
5	4	Sometimes	1 2		6	Yes		1 12 14 96	Basin insid
5	5	Never	1 2		96	Yes		1 4 14	Basin insid
5	5	Sometimes	1 2		3	Yes		1 4 7	Basin insid
0	3	Never	1 2 3		3	Yes		4 9 14	Customised
10	5	Sometimes	1 2	3 6 7		Yes		4 9 12	Basin in the
5	12	Sometimes	1 2		7	Yes		4 9 14	Basin insid
3	6	Sometimes	1 2		7	Yes		1 4	Basin insid
5	2	Sometimes	1 2		7	Yes		96	Customised
5	3	Sometimes	1 2 3		7	Yes		3 4 9 14 96	Customised
500	6	Never	1 2 3 4		1	Yes		1 4 9	Basin in the
0	5	Never	1 2 3		3	Yes		3 4 9 11 14	Basin in the
2	10	Never	1 2 4		3	No		3 4 9	Basin insid
0	4	Sometimes	1 2 3 4		3	Yes		1 3 4 9	Basin insid
0	2	Never	1 2 4		96	Yes		1 2 3 4 6 7	Basin insid
3	3	Sometimes	1 2		7	Yes		1 4 9 14	Basin insid
3	8	Sometimes	1 2		7	Yes		1 2 4 96	Customised
	8	Sometimes	1 2		3	Yes		2 4 7 9	Toilet/Kitch
7	2	Never	1 2	6 10		Yes		1 4 14	Basin insid
0	5	Often	1 2 3 4		96	Yes		1 3 4	Basin insid
5	4	Never	1 2 3 4		7	Yes		4 8 14	Basin insid
0	5	Never	1 2 3 4		11	Yes		2 4 9 14	Basin insid
5	6	Never	1 2 3 4		3	Yes		4 5 8 13 14	Customised
0	4	Sometimes	1 2 3		96	Yes		1 3 4 9	Customised
5	5	Never	2 3 4		3	Yes		4 6 9 14	Basin in the

5	4	Never	1 2		3	Yes	1 3 4 9 10	Customise
800	10	Sometimes	1 2 3		3	Yes	3 4 9 96	Basin insid
5	5	Sometimes	1 2 4		6	Yes	1 2 3	Customise
	4	Never	1 2 3 4		7	Yes	4 5 9 10 14	Customise
5	3	Sometimes	1 2 4	3 7		No	1 4 9 14	Basin in the
500	4	Never	1 2		3	Yes	1 14	Basin insid
10	3	Never	1 2	6 10		Yes	1 3 4 14	Customise
5	3	Never	1 2 3 4		3	Yes	4 9	Basin insid
5	5	Never	1 2 3 4		7	Yes	4 9 14	Basin insid
300	2	Often	1 2 4		96	Yes	1 4 9	Customise
0	7	Sometimes	1 2 3 4		3	Yes	3 4 10 14	Toilet/Kitch
0	8	Sometimes	1		6	Yes	1 4 12	Beside the
800	10	Sometimes	1 2 3		96	No	1 4	Customise
5	5	Sometimes	1 2		6	Yes	1 2	Basin insid
5	5	Sometimes	1 2		3	Yes	4 9 14 96	Basin insid
7	6	Sometimes	1 2		96	Yes	1 2 3 7 96	Customise
5	10	Never	1 2 3		7	Yes	3 4 9 14	Customise
0	2	Often	1 2 3		3	Yes	1 3 4	Basin insid
0	4	Often	1 2 3 4		96	Yes	1 4 9 96	Basin insid
5	5	Sometimes	1 2		7	Yes	4 9	Basin insid
550	3	Sometimes	1 2 96		7	Yes	1 4 14	Basin insid
5	5	Sometimes	1 2 3 4		3	No	1 2 3 4	Basin insid
400	5	Never	1 2 3 4		96	Yes	1 4 12	Beside the
5	1	Never	1 2		7	Yes	4 9 14	Customise
5	6	Never	1 2 3 4		7	Yes	4 9 14	Basin insid
400	5	Sometimes	1 2 4		11	Yes	1 3 4 12 96	Customise
5	5	Never	1 2 3 4		7	Yes	4 9 14	Customise
0	2	Sometimes	1 2		10	Yes	4 9 12 14	Basin insid
0	5	Never	1 2 4		96	Yes	1 3 4 12 14	Basin insid
5	2	Sometimes	1 2 4 96		96	Yes	1 4 9 12 14	Basin insid
5	4	Sometimes	1 2 3 4		7	Yes	3 4 9 14	Basin insid
5	6	Sometimes	1 2		3	Yes	1 4 12 14	Customise
0	3	Often	1 2 3 4		96	Yes	1 3 4 9 12	Customise
5	4	Never	1 2 3 4		7	Yes	3 4 9 10	Basin insid
5	2	Sometimes	1 2		3	No	1 4 12	Basin insid
5	8	Sometimes	1 2	3 7		No	4 14	Basin in the
5	2	Sometimes	1 2		7	Yes	3 4 9 96	Basin in the
5	2	Sometimes	1 2	3 7		Yes	3 4 9 14	Basin in the
10	10	Sometimes	1 2		7	Yes	3 4 9 14 96	Customise
5	5	Sometimes	1 2		7	Yes	4 9 96	Basin in the
2000	8	Sometimes	1 2 3 4	2 6		Yes	1 3 4 9 12	Beside the
5	5	Sometimes	1 2 3	7 9 11		Yes	3 4 9 12 14	Basin in the
0	5	Never	1 2 3 4		7	Yes	4 9 14	Beside the
5	1	Sometimes	1 2		3	Yes	1 4 9 96	Basin insid
5	10	Sometimes	1 2		3	Yes	4 9 10 14 9	Basin in the
5	3	Never	1 2 4		9	Yes	1 4 9 14	Customise
0	2	Never	1 2		2	Yes	1 4 9 12	Basin insid
10	2	Sometimes	1 2		6	Yes	1 4 12 14	Basin insid
5	4	Sometimes	1 2		8	Yes	1 4 9 11 14	Customize

0	2	Sometimes	1 2		96	Yes	4	Basin insid
450	3	Never	1 2 3 4		3	Yes	1 2 4 7 9	Toilet/Kitch
5	4	Sometimes	1 2 3		7	Yes	1 3	Basin insid
10	1	Often	1 2 4		7	Yes	1 4 12 14	Customised
5	10	Sometimes	1 2		3	Yes	4	Basin insid
0	3	Often	1 2 3		6	Yes	1 3 4 6 10	Basin insid
5	4	Never	1 2 3 4		7	Yes	4 9 14	Basin insid
5	6	Sometimes		1	9	Yes	1 9 12	Basin insid
5	5	Sometimes	1 2 3 4		3	Yes	1 3 4 9 12	Basin insid
5	2	Never	1 2 3 4		7	Yes	4 9 14	Toilet/Kitch
5	6	Sometimes	1 2 4		3	Yes	1 4 12	Basin insid
700	10	Sometimes	1 2		7	Yes	1 4 9 10 96	Customised
2	10	Sometimes	1 2 4		7	Yes	1 4 9	Customised
5	6	Sometimes	1 2 3		3	Yes	1 2 4 9 10	Basin in the
0	1	Sometimes	1 2		96	Yes	1 14	Basin insid
0	3	Never	1 2 3		2	Yes	1 4	Basin insid
5	6	Never	1 2 3 4		7	Yes	3 4 9 14	Basin insid
0	4	Sometimes	1 2 3		96	Yes	1 2 3 4 14	Customised
5	6	Never	1 2 4		7	Yes	4 9 10	Basin insid
5	3	Never	1 2	6 10		Yes	1 4 10 12	Basin insid
10	10	Sometimes	1 2		7	Yes	4 9 14	Customised
3	6	Sometimes	1 2 4		96	Yes	1 4 13 96	Basin insid
5	6	Sometimes	1 2 3 4		7	Yes	4 9 14	Basin insid
0	5	Never	1 2 3 4		96	Yes	1 2 3 4 6 7	Basin insid
10	5	Sometimes	1 2 3 4	3 7		Yes	1 4 12	Basin insid
5	5	Never	1 2 4		7	Yes	4 9 14	Basin in the
5	3	Sometimes	1 2	5 7		No	2 4 9 14	Basin insid
5	10	Never	1 2 3		7	Yes	3 4 9 10 14	Basin insid
	5	Sometimes	1 2		96	Yes	1 4 8 96	Basin insid
5	1	Never		2	3	Yes	3 4 9 14	Customised
0	3	Sometimes	1 2 4		96	Yes	1 3 4 9 12	Customised
5	4	Sometimes	1 2		3	Yes	1 4	Basin insid
0	5	Sometimes	1 2 3		3	Yes	1 4 12 14	Customised
5	5	Sometimes	1 2 3 4		3	Yes	3 4 9 14	Basin insid
5	3	Sometimes	1 2 3	3 7		Yes	3 4 9 10 12	Customised
5	8	Never	1 2 4		3	Yes	4 9	Basin insid
0	5	Sometimes	2 3 4		2	No	1 4 9 12 14	Basin insid
5	4	Sometimes	1 2		7	Yes	1 7 14	Basin insid
0	4	Sometimes	1 2 3		96	Yes	1 4 12 14	Customised
5	5	Never	1 2 3 4		3	Yes	4 9 14	Basin insid
25	2	Never	1 2 4		7	Yes	1 2 3 4 6 7	Basin insid
5	2	Sometimes	1 2		7	Yes	1 3 4 9 10	Customised
10	5	Never	1 2 4		96	Yes	3 9 14	Basin insid
5	3	Sometimes	1 2		7	Yes	1 3 4 14	Basin insid
5	5	Sometimes	1 2 3		7	Yes	1 3 4	Basin insid
5	7	Sometimes	1 2		96	Yes	1 2 3 4 12	Basin insid
150	3	Often	1 2 3 4		3	Yes	1 3 4 12 14	Basin insid
200	3	Sometimes	1 2 4		96	Yes	1 3 4 12 14	Basin insid
0	4	Sometimes	1 2 4		4	Yes	1 4 96	Customised

0	3	Sometimes	1 2 3 4	7 96		Yes	1 3 4 9 12	Basin insid
5	2	Never		1 5 6		Yes	12 14	Customised
0	10	Sometimes	1 2		3	Yes	1 4 12	Basin insid
10	3	Sometimes	1 2		7	Yes	1 4 9 14	Basin insid
5	4	Sometimes	1 2 4		3	No	1 4	Customised
5	5	Sometimes	1 2		10	Yes	1 2 3 6 12	Customised
10	5	Sometimes	1 2		7	Yes	4 9 96	Basin insid
5	12	Sometimes	1 2		7	Yes	1 4 12 14	Basin insid
5	2	Never	1 2 3 4		3	Yes	4 9 14	Basin in the
4	5	Sometimes	1 2		96	Yes	1 2 3 4 9 9	Customised
5	1	Sometimes	1 2 4		3	No	1 4 9 14	Basin insid
5	5	Never	1 2 3 4		3	Yes	3 4 5 9 14	Customised
5	6	Never	1 2		8	Yes	1 2 3 4 8 9	Customised
5	15	Never	1 2 3 4		3	Yes	3 4 10 14	Basin insid
5	4	Never	2 4		3	Yes	3 4	Basin insid
0	1	Often	1 2		3	Yes	1 4 12 13 1	Basin in the
5	5	Sometimes	1 2 3 4		3	No	1 3 4 9 12	Basin insid
5	3	Never	1 2		7	Yes	3 4 9 96	Basin in the
5	5	Often	1 2 3 4		7	Yes	1 4 9	Basin insid
5	5	Never	1 2		3	Yes	1 3 9 10 12	Customised
5	2	Never	1 2 3		3	Yes	3 4 9 10	Basin insid
5	2	Sometimes	1 2 3		7	Yes	4 14 96	Customised
0	4	Never	1 2 3 4		6	Yes	1 2 3 4 7 1	Basin insid
3	6	Sometimes	1 2	3 7		Yes	1 4 9 12 14	Basin in the
5	3	Sometimes	1 2		8	Yes	1 6 7 9 12	Customised
0	3	Often	1 2 3 4		3	Yes	1 2 3 9 12	Basin insid
5	5	Never	1 2 4		96	Yes	1 4 9 10 12	Basin insid
5	3	Never	1 2 3		7	Yes	1 3 4 9	Customised
300	10	Never	1 2 3 4		96	Yes	1 3 12 14	Toilet/Kitch
5	4	Sometimes	1 2 3 4 96		3	No	1 3 4 9 12	Basin insid
5	6	Never	1 2		7	Yes	1 4 9 12 96	Beside the
5	2	Never	1 2		7	Yes	1 4	Customized
0	3	Sometimes	1 2 3 4		6	Yes	1 4 10 12 1	Basin insid
5	6	Never	1 2		10	Yes	4 9	Basin insid
5	6	Sometimes	1 2		7	Yes	1 4 9 14	Basin in the
5	6	Sometimes	1 2 3	3 7		Yes	1 3 9 12 14	Customised
400	3	Sometimes	1 2 3		96	Yes	1 2 3 4 9 1	Customised
0	4	Sometimes	1 2 3 96		96	Yes	1 4 9 12	Customised
0	4	Never	1 2 4		96	Yes	1 3 4 12 14	Basin insid
10	3	Never		2	3	Yes	1 2 6 12	Basin insid
300	3	Sometimes	1 2 3 4		8	Yes	1 3 4 9 12	Toilet/Kitch
0	5	Sometimes	1 2 96	7 8		Yes	1 3 4 12	Basin insid
0	3	Never	1 2 3 4		96	Yes	1 4 9	Toilet/Kitch
5	1	Sometimes	1 2 3	3 7		No	3 4 12 13 1	Basin in the
0	3	Never	1 2 3 4		96	Yes	1 2 3 4 9 1	Basin insid
135	2	Sometimes	1 2 3 4		3	Yes	1 4	Basin insid
2	13	Sometimes	1 2 3	3 7		Yes	1 3 4 9 14	Basin in the
5	6	Sometimes	1 2 4		3	Yes	1 3 4 12	Basin insid
20	4	Never	1 2		7	Yes	1 4 14	Customised

0	2	Often	1 2 3 4		3	Yes		1 3 4 12 14	Customised
0	2	Never	1 2 4		96	Yes		1 3 4 12	Basin insid
5	6	Sometimes	1 2		3	Yes		1 3 4 9 12	Basin insid
0	6	Never	1 2 3 4		96	Yes		1 2 3 4 7 1	Basin insid
5	5	Never	2 3		96	Yes		1 11 12 14	Basin insid
5	5	Sometimes	1 2 4		5	Yes		1 3 4 9	Basin insid
0	6	Never	1 2 4 96		96	Yes		1 3 4 9 12	Basin insid
10	3	Sometimes	1 2		6	Yes		1 4 12	Basin insid
670	5	Never	1 2 3 4		96	Yes		1 3 9 12 14	Basin insid
7	3	Never	1 2	3 6		Yes		1 3 4	Customised
7	3	Never	1 2 4		6	Yes		1 3 4 12 14	Other (Spe
5	3	Sometimes	1 2		7	Yes		1 4 96	Basin insid
5	3	Never	1 2		7	Yes		9	Basin insid
10	3	Sometimes	1 2		3	Yes		1 3 4 12 14	Basin insid
5	5	Sometimes	1 2		8	Yes		1 4 9 12 14	Basin insid
10	5	Never	1 2 3 4	3 6		Yes		1 3 4 6 9 1	Basin insid
	4	Sometimes	1 2 3 4		96	Yes		1 3 4 9 12	Basin insid
5	5	Sometimes	1 2 3		3	Yes		1 3 4 9 12	Customised
5	6	Sometimes	1 2		3	Yes		1 3 4 6 7 1	Basin insid
10	6	Sometimes	1 2 4		7	Yes		1 4 9 12	Customised
10	1	Never	1 2		3	Yes		1 4	Customised
0	2	Sometimes	1 2		96	Yes		1 2 4 9	Basin insid
300	5	Sometimes	1 2 3 4		3	Yes		1 4	Customised
5	3	Sometimes	1 2 3 4		3	Yes		1 4 9 12	Basin in the
0	4	Often	1 2 3 4		2	Yes		1 4 9 14	Customised
5	2	Sometimes	1 2		3	Yes		1 2 3 4 9 1	Customised
500	5	Sometimes	1 2 3		7	Yes		1 3 4 12 14	Basin insid
5	30	Sometimes	1 2		4	Yes		3 4 9 96	Basin in the
2	2	Sometimes	1 2		7	No		1 4 14	Basin in the
5	10	Sometimes	1 2		8	Yes		1 3 4 6 9 1	Customised
0	3	Often	1 2		7	No	laziness	1 2 6 96	Basin insid
0	4	Never	1 2 3 4		2	Yes		1 4 12	Basin insid
8	6	Never	2		2	Yes		1 4 14	Basin insid
0	7	Never	1 2 3 4		3	Yes		4 5 9 14	Basin insid
5	2	Sometimes	1 2	6 10		Yes		1 2 3 4	Basin insid
5	5	Sometimes	1 2		8	Yes		1 4 9 12 14	Customised
200	2	Sometimes	1 2 3		3	Yes		1 3 4 9 12	Customised
0	3	Sometimes	1 2 96		6	Yes		1 4 12 96	Beside the
5	4	Sometimes	1 2		96	Yes		1 2 6 12 14	Basin insid
5	6	Sometimes	1 2		3	Yes		1 4 9 12	Basin insid
5	4	Sometimes	1 2		3	Yes		1 4 9 12 14	Basin insid
500	5	Never	1 2		96	Yes		1 3 4 7	Basin insid
10	3	Never	1 2 4		8	Yes		1 4 9 14	Customised
5	3	Often	1 2 3 4		3	Yes		1 4 9	Basin insid
5	3	Sometimes	1 2 3 4		3	Yes		1 2 3 4 6 9	Basin insid
5	2	Sometimes	1 2		3	Yes		1 12	Customised
5	3	Sometimes	1 2		7	Yes		1 2 4	Customised
5	5	Sometimes	1 2 3 4		3	Yes		1 3 4 9 12	Basin insid
3	5	Sometimes	1 2		96	Yes		1 2 4 9 12	Customized

3	5	Sometimes	1 2		7	Yes	1 4 12	Basin insid
20	5	Never	2		3	No	1 3 4	Basin insid
0	2	Never	1 2		96	Yes	1 4 96	Basin in the
0	7	Sometimes	1 2 3		3	Yes	1 3 12 14	Customise
800	5	Never	1 2 3 4		2	Yes	1 2 4 9 12	Toilet/Kitch
0	4	Sometimes	1 2		96	Yes	1 4 9 14	Customise
300	5	Sometimes	1 2 4		3	Yes	1 3 12 14	Toilet/Kitch
500	4	Sometimes	1 2		96	Yes	3 4 7	Toilet/Kitch
0	3	Often	1 2 96		3	Yes	1 4 12	Basin insid
10	3	Sometimes	1 2 3 4		3	Yes	1 2 3 4	Basin insid
200	2	Sometimes	1 2 3		96	Yes	1 14 96	Customise
200	3	Sometimes	1 2 96		96	Yes	1 3 4 12 1	Customise
5	6	Sometimes	1 2 3 4	3 7		Yes	4 9 12 14	Basin insid
5	4	Sometimes	1 2 3		3	Yes	1 3 4 9	Basin insid
500	5	Sometimes	1 2		3	Yes	1 2 3 12 1	Customise
0	3	Sometimes	1 2 3		96	Yes	1 4 12	Customise
0	4	Sometimes	1 2		3	Yes	1 4 12	Customize
3	5	Sometimes	1 2		2	Yes	1 3 4 12 1	Basin insid
5	2	Sometimes	1 2 3 4		3	Yes	1 4 9 11 1	Basin insid
5	3	Never	1 2		8	Yes	1 3 4 9 14	Customise
10	2	Sometimes	1 2		3	Yes	1 4 12 14	Basin insid
10	12	Sometimes	1 2		96	Yes	1 4	Basin insid
500	6	Sometimes	1 2 3 4		1	Yes	1 4 12 14	Customize
2	8	Sometimes	1 2		7	Yes	1 4 9 96	Basin insid
5	3	Sometimes	1 2 4		3	Yes	1 2 3 4 6 1	Basin insid
	3	Sometimes	1 2 3 4		3	Yes	1 3 4 9 12	Basin insid
5	4	Sometimes	1 2		8	Yes	1 4 9 13 1	Customise
400	7	Sometimes	1 2 3		96	Yes	1 4 9 12	Basin in the
7	3	Sometimes	1 2		3	Yes	1 4 13 14	Basin insid
4	6	Sometimes	1 2 4		3	Yes	1 3 4 9 14	Beside the
500	4	Never	1 2 3 4		96	Yes	1 3 4 9 12	Toilet/Kitch
0	5	Sometimes	1 2 4		96	Yes	1 4 14	Other (Spe
5	8	Often	1 2 4	7 96		Yes	1 4 9	Basin insid
0	5	Sometimes	1 2 3 4	4 7		Yes	1 4 5 9 12	Basin insid
10	4	Never	1 2 3 4	3 6		Yes	1 4 5 9 11	Basin insid
2000	2	Sometimes	1 2		3	Yes	1 4 12 96	Basin insid
0	4	Sometimes	1 2 4		96	Yes	1 2 4	Basin in the
0	4	Sometimes	1 2 3 4		3	Yes	1 3 4 12 1	Beside the
100	6	Often	1 2 3		96	Yes	1 3 9 12	Basin insid
5	3	Sometimes	1 2 3		7	Yes	1 4 7	Basin insid
10	5	Sometimes	1 2 3 4	3 6		Yes	1 4 9 12 1	Basin insid
	5	Never	1 2 3 4		96	Yes	1 4 9 12	Basin insid
0	3	Often	1 2		3	No	1 3 4 9	Customise
5	5	Often	1 2 3 4		96	Yes	1 3 4 9 10	Basin insid
500	1	Sometimes	1 2 3 4		3	Yes	1 14	Basin insid
5	3	Sometimes	1 2		3	No	1 2 3 4 12	Customise
0	8	Sometimes	1 2		96	Yes	1 4	Customize
5	2	Sometimes	1 2		96	Yes	1 3 9 14	Basin insid
5	3	Sometimes	1 2		96	Yes	1 4 5	Basin insid

200	2	Sometimes	1 2		96	Yes	1 4 14	Customise
300	4	Sometimes	1 2 3		3	Yes	1 12 14 96	Customize
0	2	Sometimes	1 2		96	Yes	1 3 4 9 12	Customise
10	4	Sometimes	1 2 4		3	Yes	1 4 9 10 12	Basin insid
5	6	Sometimes	1 2 4		3	Yes	1 3 4 12	Basin insid
10	3	Sometimes	1 2 3 4	3 6		Yes	1 3 4 9 12	Basin insid
7	2	Sometimes	1 2		8	Yes	1 4 9 12 14	Customise
0	3	Never	1 2		96	Yes	1 3 4	Customise
0	3	Sometimes	1 2 3 4		96	Yes	1 4 14	Basin insid
	6	Sometimes	1 2		2	Yes	1 3 4 12 14	Basin insid
300	4	Sometimes	1 2		2	Yes	1 3 4 9 10	Toilet/Kitch
10	4	Sometimes	1 2		7	Yes	1 4	Basin insid
2	4	Sometimes	1 2	3 8		Yes	1 3 4	Basin in the
10	4	Sometimes	1 2		3	Yes	1 3 4 9 12	Customise
100	4	Never	1 2 3 4 96		6	Yes	1 4 7 9 12	Basin insid
400	7	Sometimes	1 2 3 4		96	Yes	1 2 3 4 12	Toilet/Kitch
10	5	Sometimes	1 2		7	Yes	1 3 4 6 7 1	Basin insid
0	10	Sometimes	1 2 3 4	3 6		No	1 3 4 9 10	Basin insid
10	5	Sometimes	1 2		7	No	1 3 4 9 14	Basin in the
10	7	Sometimes	1 2 3	6 7		Yes	3 96	Basin insid
10	3	Sometimes	1 2 3 4	3 6		Yes	1 3 4 6 9 1	Basin insid
5	6	Sometimes	1 2		3	Yes	1 4 7 9 11	Customise
5	7	Never	1 2 3 4		3	Yes	4 9 14	Basin insid
0	5	Never	1 2		96	Yes	1 4 10 14	Customise
540	8	Never	2	2 6		Yes	4 9 14	Customise
5	6	Sometimes	1 2		7	Yes	1 4 12 14	Basin insid
550	5	Sometimes	1 2		3	Yes	1 4 9 14	Basin insid
5	3	Never	1 2	6 10		Yes	1 12	Basin insid
5	7	Sometimes	1 2 4		7	Yes	1 3 4 14	Customise
7	5	Sometimes	1 2		3	Yes	1 4 9 12 14	Customise
5	6	Sometimes	1 2		3	Yes	1 4 14	Customise
5	2	Often	1 2 3 4		96	Yes	1 4	Basin insid
0	2	Sometimes	1 2		96	Yes	1 2 12 14	Customise
0	5	Never	1 2 3 4		2	Yes	1 3 4 9	Customize
0	4	Never	2 4		2	Yes	1 3 4 12	Basin insid
0	15	Sometimes	1 2 3	3 7 11		Yes	1 4	Beside the
7	5	Sometimes	1 2		3	Yes	1 2 3 4 7 9	Basin insid
800	8	Sometimes	1 2 3 4	6 11		Yes	1 3 4 9 12	Customise
3	6	Sometimes	1 2 3		7	Yes	1 4	Basin insid
0	2	Sometimes	1 2		6	Yes	1 12 14	Customise
300	4	Sometimes	1 2 3 96		96	Yes	1	Toilet/Kitch
0	3	Sometimes	1 2		96	Yes	1 2 3 4 9 1	Customise
500	2	Sometimes	1 2 3		3	Yes	1 3 4 5 9 1	Basin insid
10	8	Sometimes	1 2 4		3	Yes	3 4 12 14	Customise
0	2	Sometimes	1 2		96	Yes	1 4 9 12 14	Customise
0	10	Often	1 2 3 4		96	Yes	1 4 12 14 9	Customize
10	2	Sometimes	1 2 3 4		3	No	1 3 4 9 10	Basin insid
800	998	Sometimes	1 2 3		3	Yes	1 4 12	Toilet/Kitch
0	2	Often	1 2 4		7	Yes	1 4 9 12 14	Customise

0	4	Never	1 2 4		96	Yes	1 4 12	Customise
3	5	Sometimes	1 2	3 8		Yes	3 4 9	Basin insid
0	5	Often	1 2 3 4		96	Yes	1 2 4 7 9 1	Basin insid
5	5	Sometimes	1 2		8	Yes	1 3 5 12 14	Basin insid
6500	5	Often	1 2		3	Yes	1 3 12	Basin in the
	3	Sometimes	1 2 3 4		3	Yes	4 9 14	Basin insid
5	3	Sometimes	1 2		7	Yes	1 4 9	Basin insid
5	6	Never	1 2 3 4		3	Yes	1 3 4 9 14	Basin in the
0	12	Often	1 2 3		2	Yes	1 4 9 12 14	Basin insid
0	4	Never	1 2 3 4	3 6		Yes	1 3 4 9 10	Basin insid
7	5	Sometimes	1 2		8	Yes	1 4 9 12 14	Customise
5	4	Sometimes	1 2		3	Yes	1 4 12 14	Basin insid
5	4	Sometimes	1 2		7	Yes	1 4 9 96	Customise
5	5	Never	1 2 3 4		7	Yes	4 9 14	Basin insid
5	3	Sometimes	1 2	6 8		Yes	1 4 8 12	Customise
5	3	Sometimes	1 2		2	Yes	1 2 4	Basin insid
5	5	Sometimes	1 2		2	Yes	1 3 4 9 12	Toilet/Kitch
0	3	Never	1 2 3		96	Yes	1 4 12 96	Toilet/Kitch
5	2	Sometimes	1 2		8	Yes	1 3 4 9 12	Customise
0	3	Sometimes	1 2		96	Yes	1 12 14	Customise
5	2	Often	1 2 3		96	Yes	1 4 96	Basin insid
0	7	Never	1 2		3	Yes	1 4 12	Customise
0	3	Sometimes	1 2 4	2 3		Yes	1 2 4	Customise
3	5	Sometimes	1 2 4		3	Yes	1 3 4 5 6 9	Basin insid
0	3	Often	1 2 3 4	3 6		Yes	1 3 4 6 9 1	Basin insid
4	2	Sometimes	1 2		3	Yes	1 3 4 5 10	Basin insid
5	4	Sometimes	1 2		7	Yes	1 4	Basin insid
5	2	Never	1 2 3 4	3 6		Yes	1 4 9 14	Basin insid
0	6	Never	1 2 4 96		96	Yes	1 3 4 12 96	Customise
135	10	Sometimes	1 2 4	6 8		Yes	1 3 4 9 12	Customise
5	2	Sometimes	1 2		3	Yes	1 3 4 12 14	Basin insid
0	3	Sometimes	1 2 3 4		96	Yes	1 3 4 6 7 9	Customise
5	2	Sometimes	1 2 3 4		3	Yes	1 4 9	Customise
0	2	Often	1 2 3 4		3	Yes	4 9 14	Basin insid
0	5	Sometimes	1 2 3 4		7	Yes	4 9 14	Basin insid
0	2	Often	1 2 4		96	Yes	1 4 9 12 14	Customise
3	6	Sometimes	1 2		7	Yes	1 4 96	Basin insid
0	4	Sometimes	1 2		96	Yes	1 4 9 12 14	Customise
0	4	Never	1 2		96	Yes	1 3 12	Customise
0	3	Sometimes	1 2 3 4	3 6		Yes	1 4 9 12 14	Basin insid
0	3	Sometimes	1 2 3		7	Yes	1 3 4	Toilet/Kitch
10	3	Sometimes	1 2 3 4	3 6		Yes	1 3 4 7 9 1	Basin insid
0	1	Never	1 2		96	Yes	4 12	Basin in the
0	6	Sometimes	1 2		96	Yes	1 2 3 4 9 1	Customise
5	4	Sometimes	1 2		8	Yes	1 3 9 14	Customise
	10	Sometimes	1 2 3 4		7	Yes	4 7 8 10 14	Toilet/Kitch
0	2	Often	1 2 3		96	Yes	1 4 9 12	Basin insid
15	5	Sometimes	1 2		7	Yes	1 12 14	Customize
300	4	Sometimes	1 2 3 4		96	Yes	1 4 14	Customise

10	2	Sometimes	1 2 3 4	3 6		Yes	1 3 4 9 12	Basin insid
5	6	Sometimes	1 2		7	Yes	1 4 9 14 96	Basin insid
0	1	Often	1 2		7	Yes	1 3 12	Customise
200	4	Sometimes	1 2		96	Yes	1 4 14	Customise
200	3	Sometimes	1 2 4		96	Yes	1 3 4 12 14	Customise
10	6	Never	1 2	6 10		Yes	1 4 12 14	Customise
5	10	Never	1 2		3	Yes	1 3 9 12 14	Basin insid
3	4	Sometimes	1 2		96	Yes	1 3 96	Basin insid
5	3	Sometimes	1 2 3		7	Yes	1 3 4 9 12	Basin insid
7	5	Sometimes	1 2		96	Yes	3 4 12 14	Customize
1000	10	Sometimes	1 2 3 4	1 2		Yes	1 4 12	Toilet/Kitch
5	2	Never	1 2 3 4	4 7		Yes	1 4 9 14	Customise
200	6	Never	1 2 4		2	Yes	1 3 4 9 12	Basin in the
5	4	Sometimes	1 2 4		3	No	1 3 4 9 12	Basin insid
5	4	Sometimes	1 2		3	Yes	1 4 12	Toilet/Kitch
5	5	Sometimes	1 2 3 4		2	Yes	1 3 4 9 12	Basin insid
0	4	Sometimes	1 2 3		96	Yes	1 3 4 9 12	Customise
300	3	Never	1 2		96	Yes	1 3 4 12 14	Customize
5	5	Sometimes	1 2 3 4		7	Yes	3 4 9 14	Basin in the
7	5	Sometimes	1 2		96	Yes	1 4 12 96	Customise
0	2	Sometimes	1 2		6	Yes	1 2 3 4 9 1	Customise
5	3	Sometimes	1 2	3 7		No	1 3 4 12 14	Customise
0	5	Sometimes	1 2 3 4		7	Yes	1 4 9 12 14	Toilet/Kitch
5	4	Never	1 2		6	Yes	1 4 9 14	Customise
10	3	Never	2 3 4	2 8 9		Yes	1 3 4 9 10	Basin insid
6	3	Sometimes	1 2		7	Yes	1 4 9 14	Customise
1580	10	Sometimes	1 2 3 4	3 7		Yes	1 2 3 4 9 1	Customise
0	3	Sometimes	1 2		6	Yes	1 12 14 96	Customize
	5	Sometimes	1 2		10	Yes	1 4 9 14	Customise
2000	4	Never	1 2 3		1	Yes	12 14	Beside the
15	4	Sometimes	1 2		96	Yes	1 4 12 96	Customise
680	4	Sometimes	1 2 3		3	Yes	1 2 3 4 9 1	Basin insid
0	3	Sometimes	1 2 96		3	Yes	1 12 14 96	Customise
7	5	Sometimes	1 2		8	Yes	1 3 4 7 9 1	Basin in the
5	4	Never	1 2		7	Yes	1 4 12	Basin insid
200	2	Sometimes	1 2 4		3	Yes	1 4 9 12 14	Customise
5	3	Sometimes	1 2 3 4		3	Yes	1 2 3 4 7 1	Basin insid
300	10	Sometimes	1 2 3 4		3	No	1 2 4 9 12	Customise
7	6	Sometimes	1 2		3	Yes	1 3 4 12 14	Basin insid
10	5	Never	2		3	Yes	3 4 9 12	Beside the
10	10	Sometimes	1 2		8	Yes	1 3 4 9 12	Customise
5	11	Often	1 2 3 4		7	Yes	1 3 4 8 14	Basin in the
5	3	Sometimes	1 2		2	Yes	1 2 4 10 14	Basin insid
5	4	Sometimes	1 2 96		2	Yes	1 4 12 14	Customise
5	4	Sometimes	1 2 4		3	Yes	1 4 9 12	Basin insid
5	3	Sometimes	1 2 3 4		11	Yes	3 9 14	Toilet/Kitch
5	5	Sometimes	1 2		3	Yes	1 3 4 9 13	Customise
5	4	Sometimes	1 2		2	Yes	1 3 4 12 14	Basin insid
5	5	Sometimes	1 2 4		3	Yes	1 3 4 12 14	Beside the

1000	6	Never	1 2 3 4		7	Yes	1 3 4 9 12	Other (Spe
500	3	Sometimes	1 2 3		3	Yes	1 3	Customised
5	2	Sometimes	1 2		7	Yes	1 4 12	Basin insid
500	10	Sometimes	1 2	6 10		Yes	1 3 4 12	Customised
300	4	Sometimes	1 2 4		96	Yes	4	Basin insid
5	4	Sometimes	1 2		3	Yes	3 4	Basin insid
0	2	Sometimes	1 2 96		96	Yes	1 4 9 14	Customised
0	5	Never	1 2		96	Yes	1 4 12 14	Customised
7	4	Sometimes	1 2 3 4		3	Yes	1 3 4 9 12	Toilet/Kitch
0	3	Sometimes	1 2 3 4		4	Yes	1 4 9 14	Basin insid
400	6	Sometimes	1 2		96	Yes	1 3 4 7 12	Customised
5	5	Sometimes	1 2 3 4	4 6		Yes	1 3 4 10 12	Basin insid
1600	5	Sometimes	1 2 3 4		96	Yes	1 3 4 12 14	Toilet/Kitch
1000	5	Sometimes	1 2		3	Yes	1 4	Basin in the
150	3	Sometimes	1 2 4		96	Yes	1 12 14 96	Customised
300	4	Sometimes	1 2 4		3	Yes	1 4 9 12	Customised
800	3	Sometimes	1 2		7	Yes	1 4 9 12 14	Basin insid
5	9	Never	1 2		8	Yes	1 3 4 12 13	Customised
5	2	Sometimes	1 2		7	Yes	1 3 4	Basin insid
5	5	Sometimes	1 2		96	Yes	1 2 4 9 12	Basin insid
5	6	Sometimes	1 2		8	Yes	1 4 9 10 14	Customised
500	12	Sometimes	1 2 3		96	Yes	1 4 14	Toilet/Kitch
5	2	Sometimes	1 2		7	Yes	14	Customised
4	6	Sometimes	1 2		3	No	1 3 4 9	Basin insid
10	4	Sometimes	1 2		3	Yes	3 4 5 10 14	Basin insid
5	6	Sometimes	1 2		7	Yes	1 2 3 4 6 9	Customised
5	3	Sometimes	1 2		3	Yes	4 96	Basin insid
5	8	Sometimes	1 2		2	Yes	1 3 5 8 11	Customised
20	6	Sometimes	1 2 3 4		7	Yes	3 4 12	Basin insid
700	5	Sometimes	1 2 3 4 96	3 7		Yes	1 4 8 9 12	Beside the
5	7	Sometimes	1 2 4		3	Yes	1 2 4 14	Basin insid
700	7	Sometimes	1 2 3		96	Yes	1 3 12 14	Basin insid
7	10	Sometimes	1 2		3	Yes	1 4 7 9 12	Customised
500	6	Sometimes	1 2 3 4		1	Yes	3 4 12	Toilet/Kitch
5	4	Sometimes	1 2		3	No	1 3 4	Basin insid
635	5	Sometimes	1 2 3		96	Yes	1 12 96	Basin insid
0	1	Sometimes	1 2 3		96	No	1 4 12	Basin insid
7	3	Sometimes	1 2		7	Yes	1 4	Basin insid
0	4	Often	1 2 4	7 96		Yes	1 4 9 12	Basin insid
5	6	Sometimes	1 2		7	Yes	1 2 4 11 14	Customised
15	10	Sometimes	2 3 96		96	Yes	1 4 9 12 14	Toilet/Kitch
800	998	Sometimes	1 2 3		3	Yes	1 4 9 12 14	Toilet/Kitch
500	5	Sometimes	1 2 3 96	6 7		Yes	1 3 4 7 9 1	Customised
5	2	Sometimes	1 2 3 4		3	Yes	1 3 4 9 96	Customised
5	4	Sometimes	1 2	3 7 8		Yes	1 4 8 12 14	Basin in the
250	5	Sometimes	1 2 4		2	Yes	1 2 4 6 10	Basin insid
0	4	Never	1 2 96		96	Yes	1 2 3 12	Basin in the
200	3	Sometimes	1 2 3 4		3	Yes	1 4	Basin insid
5	6	Sometimes	1 2 4		3	Yes	1 2 3 4	Basin insid

350	10	Sometimes	1 2 3		3	Yes	1 2 3 4 6 7	Customized
275	3	Never	1 2 3	6 10		Yes	1 4 7 9	Beside the
10	2	Sometimes	1 2 3 4		3	No	1 3 4 9 10	Basin in the
10	3	Sometimes	1 2		3	Yes	1 4	Basin insid
5	4	Sometimes	1 2		3	Yes	1 4 14 96	Basin insid
1250	6	Never	1 2 3 4		96	Yes	1 3 14	Toilet/Kitch
700	4	Sometimes	1 2 3		3	Yes	3 4 9 10 96	Customised
10	3	Sometimes	1 2 3		96	Yes	1 4 7 9	Toilet/Kitch
300	6	Never	1 2 4		6	Yes	1 4	Customised
0	4	Sometimes	1 2 3 4 96		11	Yes	1 2 3 4 9 1	Customised
5	5	Sometimes	1 2		7	Yes	1 3 6 96	Basin insid
5	5	Never	1 2 3 4		3	Yes	1 4 14	Customised
5	3	Sometimes	1 2		96	Yes	1 3 4	Customised
5	4	Sometimes	1 2 3		3	Yes	1 4 14	Customised
5	2	Sometimes	1 2 4		6	Yes	1 3 4 7 9 1	Basin in the
5	6	Sometimes	1 2		8	Yes	1 4 9 12 14	Customised
7	2	Sometimes	1 2 4		7	Yes	1 4 12 14	Customised
3	10	Sometimes	1 2		3	Yes	1 2 3 4 6 9	Customised
7	2	Sometimes	1 2 96		7	Yes	1 2 4 9 14	Basin insid
500	10	Sometimes	1 2 4 96		4	Yes	1 12	Beside the
5	6	Never	1 2 3		3	Yes	4 9 14	Basin in the
0	3	Never	1 2 3 4		96	Yes	1 2 3 4 6 9	Basin insid
100	7	Sometimes	1 2		3	Yes	1 3 4 12	Customized
0	2	Sometimes	1 2		3	Yes	1 4 9 12 14	Basin insid
0	4	Sometimes	1 2		96	Yes	1 2 4 6 14	Customized
400	4	Sometimes	1 2 3 4		96	Yes	1 2 4 12 14	Toilet/Kitch
0	1	Sometimes	1 2		3	Yes	1 12 14	Basin insid
7	5	Sometimes	1 2 4		96	Yes	1 2 3 4 9	Customised
10	3	Often	1 2 3 4		3	Yes	1 3 6 7 9 1	Basin insid
5	4	Sometimes	1 2 4		3	Yes	1 2 4 12 14	Basin insid
5	3	Sometimes	1 2		8	Yes	1 4 8 11 13	Customised
5	2	Sometimes	1 2		7	Yes	1 4	Basin insid
7	5	Sometimes	1 2		3	Yes	1 3 4 9 14	Basin insid
5	6	Sometimes	1 2		7	Yes	1 3 4 6 7 1	Basin insid
0	7	Sometimes	1 2		96	Yes	1 4 96	Customized
10	3	Sometimes	1 2		3	Yes	1 4 14	Basin insid
5	2	Never	2 4		3	Yes	1 3 4 9 12	Customised
0	2	Often	1 2 3		96	Yes	1 3 4	Basin insid
0	3	Never	1 2		96	Yes	1 3 4 12	Basin insid
5	6	Sometimes	1 2		3	Yes	1 3 4 9 12	Customised
0	7	Sometimes	1 2 3 4		96	Yes	1 3 4 12	Basin insid
1500	5	Sometimes	1 2	3 7		Yes	1 4 8 9 12	Basin in the
150	4	Sometimes	1 2 3		96	Yes	1 3 4 9 12	Basin insid
750	5	Sometimes	1 2 3 4		2	Yes	1 3 4 12 14	Toilet/Kitch
5	5	Sometimes	1 2		3	Yes	1 4 13 14	Customised
5	5	Sometimes	1 2		8	Yes	1 4 9 12 14	Customised
0	4	Sometimes	1 2 4		3	Yes	1 4 14	Basin insid
500	5	Never	1 2 3		1	Yes	4 14	Toilet/Kitch
500	6	Sometimes	1 2 3		2	Yes	1 3 4 9 12	Toilet/Kitch

500	3	Often	1 2 3 4		3	Yes	1 3 4	Basin insid
7	4	Never	1 2		96	Yes	1 4 14	Basin insid
5	2	Sometimes	1 2		7	Yes	1 4 12	Basin insid
10	6	Sometimes	1 2		10	Yes	1 2 3 7	Basin insid
5	10	Sometimes	1 2		2	Yes	1 2 4 9 12	Customised
5	4	Sometimes	1 2 4		2	Yes	1 3 4 12 14	Basin insid
0	3	Sometimes	1 2 3		96	Yes	1 2 3 4 6 9	Customised
478	5	Often	1 2 3 4		96	Yes	1 4 12	Basin insid
5	20	Never	1 2 4		2	Yes	1 4 9 11 12	Basin in the
500	4	Sometimes	1 2		3	Yes	1 3 4 9 12	Basin insid
0	2	Sometimes	1 2	6 8		Yes	1 3 4 9 12	Customised
8	5	Sometimes	1 2		7	No	1 4 9 12	Basin insid
0	5	Sometimes	1 2 3 96	3 96		Yes	1 2 3 12 14	Customized
250	5	Never	1 2 3 4		1	Yes	1 4 9 12	Toilet/Kitch
8	4	Never	1 2 4		6	Yes	3 4 14	Basin insid
0	6	Never	1 2		96	Yes	1 4 12 14	Customised
7	3	Never	1 2		6	Yes	1 3 6 12	Basin insid
5	20	Never	2 4		3	Yes	1 3 4 14	Basin insid
3000	3	Never	1 2		3	Yes	4 96	Basin in the
5	6	Sometimes	1 2		96	Yes	2 4 7 9 14	Customized
0	5	Never	1 2		2	Yes	1 3	Basin insid
200	5	Never	1 2 4		7	Yes	1 3 9 12	Beside the
	20	Sometimes	1 2 3 4		3	Yes	1 4 9 14	Beside the
0	5	Never	1 2 3		96	Yes	1 3 12 14	Toilet/Kitch
0	15	Sometimes	1 2 3 4		3	Yes	4 9	Customised
200	3	Sometimes	1 2 3 4		3	Yes	1 2 3 4 9 1	Basin insid
5	6	Sometimes	1 2 3 4		3	Yes	4 9 14	Basin insid
5	2	Never	1 2 3		96	Yes	1 3 14	Basin insid
0	5	Sometimes	1 2 4		3	Yes	1 3 4 9 12	Customised
300	5	Sometimes	1 2 3		3	Yes	1 3 4 12 14	Customised
5	4	Often	1 2 3		96	Yes	1 4 12	Basin insid
0	5	Sometimes	1 2		96	Yes	1 3 4 7 12	Customised
1500	5	Sometimes	1 2 3		3	Yes	1 3 4 9 12	Toilet/Kitch
0	2	Sometimes	1 2		3	Yes	1 4 14	Customised
350	2	Sometimes	1 2		7	Yes	1 14	Customized
5	2	Never	1 2 4	5 6		Yes	4 9 14	Toilet/Kitch
250	2	Never	1 2 3 4		96	Yes	1 2 4 12 14	Basin insid
0	3	Never	2 3 4	2 6		No	1 3 4 6 12	Basin insid
5	12	Sometimes	1 2 3 4		7	Yes	4 9 14	Basin in the
5	5	Sometimes	1 2 3 4	2 6		Yes	1 3 4 9 10	Toilet/Kitch
400	6	Sometimes	1 2 3		96	Yes	1 4 9 14	Customised
400	6	Sometimes	1 2 3 4		3	Yes	1 3 4 9 10	Toilet/Kitch
5	8	Never	1 2		7	Yes	1 4 9 12 14	Customised
800	5	Sometimes	1 2 3 4		11	Yes	1 3 4 9 12	Customised
0	3	Never	1 2		96	Yes	1 3 12	Customised
10	6	Sometimes	1 2 3	3 7		Yes	1 3 4	Customised
300	8	Never	1 2 4		3	Yes	1 3 4 12 96	Customised
4	4	Sometimes	1 2		96	Yes	1 4 12 14	Customised
0	5	Sometimes	1 2		96	Yes	1 3 4 12 14	Customised

0	3	Never	1 2 96		96	Yes		1 4 12	Basin insid
0	3	Sometimes	1 2		96	Yes		1 4 9 14	Basin insid
0	5	Sometimes	1 2 3 4		3	Yes		1 2 3 4 6 7	Basin insid
0	5	Never	1 2 3 4		96	Yes		1 3 4 12 14	Basin insid
5	10	Sometimes	1 2 3		7	Yes		1 4 14	Basin insid
10	5	Sometimes	1 2		3	Yes		1 4 12 14	Basin insid
5	4	Sometimes	1 2		7	Yes		1 4 14	Customise
5	7	Sometimes	1 2		3	Yes		1 3 4 8 9 1	Customise
0	5	Never	1 2		2	Yes		1 2 3 4 9 1	Basin insid
500	3	Sometimes	1 3 4		2	Yes		1 3 4 5 6 7	Basin insid
350	6	Sometimes	1 2 3		3	Yes		1 12 14	Basin insid
5	7	Sometimes	1 2		8	Yes		1 4 9 13 14	Customise
1500	2	Sometimes	1 2 3 4	2 6		Yes		1 3 4 9 12	Customise
500	3	Sometimes	1 2 3 4	3 6		Yes		1 2 3 4 9 1	Basin insid
7	6	Never	1 2		10	Yes		2 4 12	Basin insid
5	12	Sometimes	1 2		8	Yes		1 4 5 9 12	Basin insid
5	3	Sometimes	1 2 3 4	3 7 8		Yes		1 4 12 14	Basin in the
5	6	Sometimes	1 2 4		3	Yes		1 3 4 12	Basin insid
100	10	Sometimes	1 2 3		3	Yes		1 4 12	Basin insid
5	3	Sometimes	1 2		3	Yes		1 4 12 14	Customise
5	2	Sometimes	1 2 3	3 7		Yes		3 4 8 14 96	Customise
0	2	Never	1 2 3 4		96	Yes		1 2 3 4 7 9	Basin insid
3	5	Sometimes	1 2		7	Yes		1 14 96	Customise
300	4	Never	1 2 3 4		6	Yes		1 3 4 5 9 1	Basin insid
0	4	Sometimes	1 2		3	Yes		1 4 12 14	Basin insid
300	5	Sometimes	1 2 3 4		96	Yes		1 4 12 14	Basin insid
0	6	Sometimes	1 2 3 4		96	Yes		1 3 4 9 12	Customise
5	10	Often	1 2 3 4 96		3	No	No culture	4 12	Customise
10	3	Never	1 2 3 4	2 6		Yes		1 3 4 9 12	Customise
5	2	Sometimes	1 2 4	3 8		Yes		1 3 12 14	Basin insid
5	4	Sometimes	1 2	7 8 9		Yes		1 3 4 9 12	Basin in the
7	4	Never	1 2	6 10		Yes		1 4 13	Customise
0	4	Never	1 2		96	Yes		1 3 4	Customise
100	15	Sometimes	1 2 3 4	3 6		Yes		1 3 4 9 12	Basin insid
5	5	Sometimes	1 2		7	Yes		1 3 8 12 14	Basin in the
500	10	Sometimes	1 2 3 4		2	Yes		1 2 3 4 12	Toilet/Kitch
0	3	Never	1 2 3 4		2	Yes		1 3 4 5 9 1	Basin insid
0	5	Never	1 2 3 4		96	Yes		1 2 3 4 9 1	Customise
5	2	Often	1 2 3 4		96	Yes		1 4 12	Basin insid
0	5	Sometimes	1 2 3 4		3	Yes		1 2 3 4 9 1	Basin insid
2	2	Sometimes	1 2 3		7	Yes		1 4	Basin in the
0	5	Never	2		3	Yes		1 4 10 14	Customise
5	6	Sometimes	1 2		96	Yes		1 2 4 12 14	Basin insid
5	4	Sometimes	1 2		3	Yes		1 3 4 12 14	Basin insid
300	5	Sometimes	1 2		96	Yes		1 4 12	Basin insid
3500	10	Sometimes	1 2 3 4		7	Yes		1 9 12 13	Customize
10	5	Never	1 2 3 4	2 6		Yes		1 3 4 9 10	Customise
5	6	Sometimes	1 2 4	3 8		Yes		1 2 3 4 6	Basin insid
1000	3	Sometimes	1 2		3	Yes		1 2 14 96	Beside the

7	5	Sometimes	1 2		3	Yes	1 3 4 10 14	Basin insid
5	6	Sometimes	1 2	3 7		Yes	1 4 12 14	Customised
0	3	Sometimes	1 2 4		3	Yes	1 4 9 12	Basin insid
0	2	Sometimes	1 2 3		3	Yes	1 2 4	Customised
5	3	Sometimes	1 2 4		96	Yes	1 4 14	Basin insid
5	5	Sometimes	1 2	6 7		Yes	1 3 4 9 12	Customised
5	10	Sometimes	1 2 3		7	Yes	1 4 12	Basin insid
0	4	Sometimes	1 2 3		3	Yes	1 4 14	Basin insid
800	998	Sometimes	1 2 3		3	Yes	1 3 4 9 12	Beside the
0	1	Never	1 2		96	Yes	1 12 14	Customised
0	6	Sometimes	1 2 3 4		3	Yes	1 4 12 14	Customised
5	6	Sometimes	1 2 4		3	Yes	1 2 3 4	Basin insid
0	6	Often	1 2 3 4		96	Yes	1 2 4 9 14	Customised
500	3	Sometimes	1 2 4 96		96	Yes	1 4 14	Customised
0	1	Sometimes	1 2 3 4		96	Yes	1 4 12	Toilet/Kitch
5	4	Sometimes	1 2 3		3	Yes	1 4 12 14	Basin insid
0	1	Often	1 2 3		7	Yes	1 4	Basin insid
15	3	Sometimes	1 2 3 4	3 6		Yes	1 4 9 10 12	Basin insid
2	5	Sometimes	1 2		3	Yes	1 3 4 9	Basin insid
5	5	Sometimes	1 2 4		3	Yes	1 9 12	Basin insid
5	12	Sometimes	1 2		8	Yes	1 4 9 12 14	Customised
0	5	Sometimes	1 2 4	3 10		Yes	1 4 9	Customised
5	4	Sometimes	1 2		3	Yes	1 3 4	Basin insid
0	1	Sometimes	1 2 3 4		2	Yes	1 4 9 12 14	Basin insid
5	5	Sometimes	1 2 3 4		3	Yes	1 2 3 4 6 9	Basin insid
5	3	Sometimes	1 2 4		3	Yes	1 3 4 12 14	Basin insid
5	6	Sometimes	1 2 3 4		7	Yes	4 9 10 14	Basin in the
5	1	Sometimes	1 2		3	Yes	1 4 12 14	Basin insid
0	3	Sometimes	1 2 3 4		3	Yes	1 2 3 4 7 1	Customised
0	2	Often	1 2		3	Yes	1 4 5	Basin insid
5	3	Sometimes	1 2		2	Yes	1 3 12	Basin insid
300	5	Sometimes	1 2 3 4		11	Yes	1 3 4 12	Customised
0	2	Never	1 2		96	Yes	1 12 14	Customised
5	10	Sometimes	1 2		3	Yes	3 4 9 10 96	Basin insid
5	4	Sometimes	1 2		9	Yes	1 4 9 14	Customised
0	3	Sometimes	1 2 3 4	3 6		Yes	1 3 4 9 12	Basin insid
0	4	Never	1 2 3		96	Yes	1 4 6	Toilet/Kitch
0	4	Sometimes	1 2 3 4		96	Yes	1 3 4 12 14	Basin insid
5	4	Sometimes	1 2		1	Yes	1 2 3 4 6 9	Customised
5	3	Sometimes	1 2		96	Yes	1 4 12	Customised
1400	5	Never	1 2		96	Yes	1 4 12	Toilet/Kitch
300	3	Sometimes	1 2		5	No	1 4	Customised
5	5	Sometimes	1 2		7	Yes	4 9 12	Basin insid
435	998	Sometimes	1 2		3	Yes	1 4 9 12 14	Customised
5	4	Often	1 2 4		96	Yes	1 4 9 12 14	Basin insid
3	2	Sometimes	1 2		7	Yes	1 4 7	Basin insid
5	6	Sometimes	1 2	3 7 8		Yes	1 3 4 9 10	Basin in the
5	4	Sometimes	1 2 4		3	Yes	1 3 4	Basin insid
10	1	Sometimes	1 2		7	Yes	1 4 12	Basin insid

0	4	Sometimes	1 2 3 4		3	No	Lack of tim	1 4 9 12 1	Customise
0	3	Never	1 2 3 4	2 3		Yes		1 2 3 4 9 1	Basin insid
0	2	Never	1 2		96	Yes		1 3 4	Customise
5	6	Often	1 2 3		96	Yes		1 4 9 13 1	Customise
5	5	Sometimes	1 2		8	Yes		1 3 4 7 9 1	Customise
5	6	Never	1 2		8	Yes		1 3 4 9 14	Customise
7	4	Never	1 2		96	Yes		1 3 4 96	Customise
10	3	Sometimes	1 2		3	Yes		1 4 5 9 13	Customise
5	3	Never	2 3 4		3	Yes		1 4 9	Basin insid
10	5	Sometimes	1 2 3 4		3	Yes		1 3 4 9 10	Customise
5	6	Sometimes	1 2	6 7		Yes		1 4 8 9 10	Customise
0	3	Sometimes	1 2		2	Yes		4 14	Customise
800	8	Sometimes	1 2 3		3	Yes		1 3 4 6 12	Basin insid
0	5	Sometimes	1 2		96	Yes		1 3 4 12 1	Customise
0	3	Often	1 2		96	Yes		1 2 12 14	Customise
5	6	Sometimes	1 2 3 4		7	Yes		1 4 9 12 1	Basin in th
5	6	Sometimes	1 2		7	Yes		3 4 6 14	Basin insid
5	5	Sometimes	1 2		3	Yes		1 4 9 12 1	Basin insid
0	6	Often	1 2 3 4		3	Yes		1 4 9 12	Basin in th
1000	2	Sometimes	1 2 3		3	Yes		1 4 9 14	Toilet/Kitch
0	5	Often	1 2 3 4		96	Yes		1 4 9 12	Customise
800	5	Sometimes	1 2		3	Yes		1 3 4 9 12	Customise
1500	20	Sometimes	1 2		3	Yes		1 4 8 10 9	Customise
5	6	Sometimes	1 2		96	Yes		1 4 12 14	Customise
300	2	Sometimes	1 2 4		2	Yes		1 4 9	Basin insid
200	3	Sometimes	1 2		6	Yes		1 2 4 9 14	Customize
0	5	Never	1 2 4		3	Yes		1 3 4 6 9 1	Basin in th
5	5	Never	1 2	3 7		Yes		1 3 4 12 1	Basin in th
0	5	Never	1 2		96	Yes		1 2 4 9 14	Customise
2300	3	Sometimes	1 2 3		96	Yes		1 4 12 96	Toilet/Kitch
5	5	Often	1 2 3 4		96	Yes		1 2 3 4 9 1	Basin insid
10	3	Sometimes	1 2		3	Yes		1 4 9 12	Basin insid
5	10	Sometimes	1 2	6 10		Yes		1 9 14	Basin insid
5	5	Sometimes	1 2 3 4		7	Yes		4 9 14	Basin insid
700	3	Sometimes	1 2 3		96	Yes		1 4 9 12 1	Customise
5	10	Never	1 2	6 10		Yes		1 3 4 14	Basin insid
5	4	Sometimes	1 2		3	Yes		1 3 4 9 14	Customise
0	4	Never	1 2		96	Yes		1 12 14	Customise
12	4	Sometimes	1 2		7	Yes		1 4	Basin insid
200	4	Sometimes	1 2		3	Yes		1 4 9 12 1	Customise
0	2	Sometimes	1 2 3 4		3	Yes		1 4 5 9 96	Basin insid
200	5	Sometimes	1 2 3 4 96		96	Yes		1 3 4 9 11	Customise
2	3	Sometimes	1 2	3 7		Yes		15	Basin in th
200	5	Never	2 3 4		3	Yes		1 3 4 14	Beside the
5	2	Often	1 2 3		96	Yes		1 4 9	Basin insid
10	4	Often	1 2		7	Yes		1 3 4 9	Basin insid
5	5	Sometimes	1 2 3 4		7	Yes		4 9 14	Basin insid
5	2	Sometimes	1 2 3		3	No		1 4 9	Basin insid
5	10	Sometimes	1 2		3	Yes		1 3 4 9 12	Customise

5	5	Sometimes	1 2		3	Yes		1 3 4 12 14	Basin insid
5	3	Sometimes	1 2		7	Yes		1 4 9 12 14	Basin insid
4	7	Sometimes	1 2		3	Yes		3 4 12 14	Basin insid
	3	Often	1 2 3 4	3 8		Yes		1 2 4 6 7 9	Basin insid
500	3	Sometimes	1 2 3 4		2	Yes		4 9	Basin insid
7	6	Sometimes	1 2		8	Yes		1 2 3 4 9 1	Basin insid
800	1	Sometimes	1 2		6	Yes		3 4 12	Customise
5	4	Often	1 2 3		3	Yes		1 2 4 12	Basin insid
5	6	Sometimes	1 2		3	Yes		1 4 7 12 14	Customise
5	3	Sometimes	1 2 4		3	Yes		1 4 12	Customise
5	2	Never	1 2		6	Yes		14	Customize
5	5	Sometimes	1 2	3 7		No		1	Basin in the
5	4	Sometimes	1 2		3	Yes		1 3 4 12 13	Basin insid
3	2	Sometimes	1 2 4		3	Yes		1 3 4 12 14	Basin insid
5	3	Sometimes	1 2		8	Yes		1 3 4 9 12	Customise
5	8	Often	1 2		8	Yes		1 4 8 9 13	Customise
5	5	Never	1 2 3		3	Yes		4 9 14	Basin insid
0	5	Sometimes	1 2		96	Yes		4 7 9 12 14	Basin in the
15	1	Never	1 2		7	Yes		1 4 14	Basin insid
0	2	Sometimes	1 2		3	Yes		1 3 9 12 14	Customise
180	3	Sometimes	1 2 4		96	Yes		1 4 9 11 12	Customise
0	2	Never	1 2		96	Yes		1 3 4	Customise
0	10	Sometimes	1 2		96	Yes		1 4 14	Basin insid
300	3	Sometimes	1 2 3 4	1 7		Yes		1 3 4 9 12	Toilet/Kitch
3	7	Sometimes	1 2 3	3 7		Yes		1 4 9 12 14	Basin in the
100	2	Sometimes	1 2 3 4		5	Yes		2 3 4 9 12	Basin insid
0	2	Never	1 2		96	Yes		1 3 12 14	Customise
5	4	Sometimes	1 2		8	Yes		1 3 5 9 12	Customise
0	5	Sometimes	1 2		8	No	Feeling tire	4 14	Basin insid
4	6	Sometimes	1 2 4		3	Yes		1 2 3 4 7 1	Basin insid
30	6	Sometimes	1 2 3 4	3 6		Yes		1 2 3 4 6 9	Customise
7	5	Sometimes	1 2 4		8	Yes		1 3 4 12 14	Basin insid
5	4	Sometimes	1 2		96	Yes		4 12 14 96	Basin insid
300	5	Sometimes	1 2		96	Yes		1 3 4 12 14	Customize
30	2	Never	2		3	Yes		1 3 12	Beside the
4500	7	Sometimes	1 2 3 96		96	Yes		1 4 12 14	Other (Spe
5	6	Sometimes	1 2 4		2	Yes		1 3 4 14	Basin insid
0	4	Never	1 2		7	Yes		1 3 7	Customise
5	3	Sometimes	1 2		7	Yes		1 3 9	Basin insid
20	4	Never	2 3		96	Yes		1 4	Basin insid
0	3	Sometimes	1 2	11 96		Yes		1 3 4 9 12	Customise
5	3	Sometimes	1 2 4		2	Yes		1 3 4 9 12	Basin insid
5	3	Sometimes	1 2		3	Yes		1 3 4 9 12	Customise
200	3	Often	1 2 3 4		2	Yes		1 3 4 9 12	Basin insid
5	10	Never	2 3		3	Yes		1 4 9 12	Toilet/Kitch
2880	8	Never	1 2 4		10	Yes		1 4 12 14	Customise
500	15	Sometimes	1 2 3 4		3	Yes		1 4 12 14	Customize
500	10	Sometimes	1 2 3 4		1	Yes		1 4 5 12	Beside the
0	3	Sometimes	1 2 3 4	3 6 8		Yes		1 3 4 9 10	Basin insid

4	4	Sometimes	1 2 4		3	Yes	1 3 4 12 14	Customised
0	6	Sometimes	1 2		96	Yes	1 3 4 14	Customised
10	12	Sometimes	1 2		3	Yes	1 3 9 12 14	Customised
5	4	Never	1 2		6	Yes	2 4 7 14	Basin insid
500	6	Sometimes	1 2 96		3	Yes	1 4 9 12 96	Customised
5	5	Sometimes	1 2		3	Yes	1 3 4 12 14	Customised
0	3	Often	1 2		3	Yes	1 4 9 96	Customised
5	3	Sometimes	1 2 3		7	Yes	1 4 6 7 9	Basin insid
10	10	Sometimes	1 2 3 4		2	Yes	1 3 4 5 9 1	Basin insid
5	8	Never	1 2 4		7	Yes	1 2 3 4 12	Customized
0	2	Often	1 2	3 7		Yes	4 9 96	Customised
0	2	Sometimes	1 2 3		3	Yes	1 4 9 14	Customized
10	4	Often	1 2 3 4		96	Yes	1 2 4 9 12	Customised
5	6	Never	1 2 3 4		3	Yes	3 4 9 14	Basin insid
5	5	Sometimes	1 2 4		3	Yes	1 3 4 5 14	Customised
200	5	Sometimes	1 2 4		3	Yes	1 3 4 96	Basin insid
0	5	Sometimes	1 2 3 4		3	Yes	1 3 4 9 12	Customised
10	4	Never	2 3 4	3 4		Yes	1 3 4 9 10	Basin insid
200	5	Sometimes	1 2		96	Yes	1 2 4 12 14	Customised
5	5	Sometimes	1 2		3	Yes	1 3 9 12 14	Customised
600	3	Sometimes	1 2 3 4		7	Yes	1 3 4 9 12	Toilet/Kitch
0	3	Sometimes	1 2 3		3	Yes	1 4 9 12	Basin insid
5	6	Sometimes	1 2 3 4	3 7 8		No	1 3 4 9 12	Basin in the
0	2	Sometimes	1 2 4 96		11	Yes	1 3 4 9 11	Basin insid
5	6	Sometimes	1 2 4		3	Yes	1 3 4 9 12	Basin insid
3	5	Sometimes	1 2 4		3	Yes	1 3 4 6 7 9	Customised
300	6	Never	1 2		96	Yes	1 4 12 14	Customized
5	1	Sometimes	1 2		3	Yes	4 5 9	Basin in the
3	6	Sometimes	1 2 4		8	Yes	1 3 4 12 14	Toilet/Kitch
5	4	Sometimes	1 2 3	3 7		Yes	1 4 9 14	Basin in the
0	2	Often	1 2 3		3	Yes	1 4 9 12	Basin insid
5	5	Sometimes	1 2 3		3	Yes	1 2 3 4 7 9	Toilet/Kitch
200	5	Often	1 2 4		96	Yes	1 4 7 9 12	Basin insid
4000	1	Sometimes	1 2		96	Yes	1 4 12	Toilet/Kitch
3	5	Sometimes	1 2		7	Yes	1 4 14	Basin insid
10	5	Sometimes	1 2		7	Yes	1 2 3 4	Customized
5	5	Sometimes	1 2	6 7		Yes	1 3 4 9 12	Customised
7	2	Sometimes	1 2 3 4		3	Yes	1 3 4 9 12	Basin insid
10	3	Sometimes	1 2 3 4	3 6		Yes	1 3 4 9 12	Basin insid
	2	Sometimes	1 2 3		3	Yes	1 12 14 96	Basin insid
5	10	Never	1 2 3 4		7	Yes	3 4 9 14	Customised
10	10	Never	1 2 4		3	Yes	1 3 4 9 11	Basin insid
5	4	Sometimes	1 2 3		3	Yes	1 4 6 7 12	Basin insid
5	3	Sometimes	1 2		3	Yes	1 2 3 4 7 1	Basin insid
2100	3	Sometimes	1 2 3		96	Yes	1 4 9 12 14	Other (Spe
5	10	Sometimes	1 2		2	Yes	1 3 4 9 12	Customised
5000	3	Sometimes	1 2		96	Yes	4 96	Toilet/Kitch
6	5	Sometimes	1 2		8	Yes	1 4 9 12 14	Customised
5	10	Often	1 2 3		3	Yes	3 4 9 12 14	Customised

0	3	Sometimes	1 2 3 4		3	Yes	1 4 12 14	Customise
5	8	Sometimes	1 2 3 4	3 6 7		Yes	1 2 3 4 6 8	Basin in the
1200	6	Sometimes	1 2		7	Yes	1 4 14	Customise
5	7	Never	1 2 3 4		3	Yes	1 4 12	Basin in the
10	5	Sometimes	1 2	3 7		Yes	1 2 3 4 9 1	Basin in the
5	6	Often	1 2 3	7 96		Yes	1 4 96	Basin insid
5	4	Never	1 2 4	6 7		Yes	3 4 7 12 14	Customise
5	4	Sometimes	1 2 3	3 7		Yes	1 3 4 12 96	Basin in the
0	10	Sometimes	1 2 3 4		96	Yes	1 3 4 9 12	Toilet/Kitch
5	4	Never	1 2		6	Yes	1 4 96	Customise
200	5	Sometimes	1		8	Yes	1 2 3 4 12	Customise
4	4	Sometimes	1 2		3	Yes	1 3 4 9	Basin insid
1500	4	Often	1 2 3 4		1	Yes	1 4 9 12 14	Toilet/Kitch
0	2	Never	1 2 3 4		3	Yes	1 4 9 12 14	Basin insid
0	7	Sometimes	1 2		3	Yes	1 12 14 96	Customise
5	5	Sometimes	1 2		8	Yes	1 4 9 12 14	Basin insid
5	10	Sometimes	1 2		7	Yes	1 4 14	Basin insid
5	6	Sometimes	1 2		2	Yes	1 3 4 9 14	Customise
5	4	Sometimes	1 2 3 4		7	Yes	1 4 9 12 96	Basin insid
10	3	Sometimes	1 2 3 4	3 6		Yes	1 3 4 9 12	Basin insid
1600	3	Never	1 2 3 4		96	Yes	1 4 12	Customize
20	5	Never	1 2 3 4		6	Yes	1 96	Customise
0	5	Sometimes	1 2 96		96	Yes	1 4 9 12 14	Customise
5	1	Never	1		3	Yes	3 4 6 9 96	Basin insid
0	3	Sometimes	1 2 4		3	Yes	1 4 9	Basin insid
250	6	Sometimes	1 2 3 4		96	Yes	1 3 4 12 96	Customize
5	8	Often	1 2 3		96	Yes	1 3 4 6 9 1	Basin insid
0	6	Never	1 2 3		96	Yes	1 4	Customise
5	6	Sometimes	1 2		8	Yes	1 3 4 9 12	Customise
5	4	Often	1 2 4 96		96	Yes	1 4 7 12 96	Basin insid
5	6	Sometimes	1 2		8	Yes	1 4 9 12 14	Customise
7	6	Never	1 2 3 4	3 6		Yes	1 2 3 4 7 9	Basin insid
5	3	Sometimes	1 2	3 7 11		Yes	1 2 3 4 9 1	Basin in the
5	3	Sometimes	1 2		8	Yes	1 4 9 10 12	Customise
7	3	Never	1 2 3 4	3 6		Yes	1 3 4 9 10	Basin in the
3	6	Sometimes	1 2		3	Yes	1 3 4 12 14	Basin insid
5	5	Never	1 2 3 4		7	Yes	4 9 14	Customize
0	6	Sometimes	1 2		8	Yes	1 2 4 9 12	Customise
7	2	Sometimes	1 2		10	Yes	1 3 4 6	Basin insid
0	5	Sometimes	1 2		7	Yes	1 4 9 14	Customise
7	5	Sometimes	1 2 4		3	Yes	1 4 12 14	Basin insid
5	10	Sometimes	1 2 4		3	Yes	1 3 4 10 12	Basin in the
5	3	Sometimes	1 2 4		3	Yes	1 4 12 14	Basin insid
5	3	Sometimes	1 2 3 4	2 6		Yes	1 4 9 10	Basin insid
10	10	Often	1 2		3	Yes	1 4 12 96	Basin insid
5	5	Sometimes	1 2 3 4		3	Yes	1 4 14	Basin in the
1000	5	Sometimes	1 2		7	Yes	1 4 9	Beside the
0	8	Sometimes	1 2		96	Yes	1 4 12	Customise
300	5	Sometimes	1 2 3 4		3	No	1 4 14	Customise

1000	5	Sometimes	1 2 3 96		96	Yes	1 2 3 4 14	Customise
5	6	Sometimes	1 2 3 4	2 3		Yes	1 3 4 9 12	Basin insid
	5	Sometimes	1 2 3 4	2 7		Yes	1 4 14	Basin in the
250	8	Sometimes	1 2		3	Yes	1 3 4 7 9 1	Customise
5	10	Sometimes	1 2 3 4	3 6		Yes	1 2 3 4 7 9	Basin insid
5	3	Sometimes	1 2		7	Yes	1 4 14	Basin in the
3	10	Sometimes	1 2		3	Yes	1 4 12	Basin insid
0	3	Never	1 2		96	Yes	1 4 9	Basin insid
1000	4	Never	1 2 3 4	2 7		Yes	1 4 9	Beside the
0	2	Sometimes	1 2 4		96	Yes	1 2 3 4 14	Customise
5	3	Sometimes	1 2 3		7	Yes	1 4 96	Basin insid
5	3	Sometimes	1 2		8	Yes	1 3 4 9 10	Customise
300	6	Sometimes	1 2 4		2	Yes	1 3 4 12 14	Basin insid
700	998	Sometimes	1 2 3	3 6		Yes	1 3 4 12 14	Beside the
5	7	Sometimes	1 2		3	Yes	1 3 4 12	Basin insid
0	3	Never	2 4		3	Yes	4 9 14	Basin insid
5	10	Sometimes	1 2 3		3	Yes	1 3 4 9 12	Beside the
5	4	Never	1 2		8	Yes	1 4 9 12 14	Customise
5	3	Sometimes	1 2 4	3 7 8		No	1 4 12 14	Basin insid
0	7	Sometimes	1 2 4		6	Yes	1 4 9 14	Customise
0	3	Never	1 2 3 4	2 4		Yes	1 3 4 9 14	Basin insid
5	7	Sometimes	1 2		3	Yes	1 4 14	Basin insid
5	8	Sometimes	1 2		8	Yes	1 4 9 12 14	Customise
550	6	Often	1 2 3 4		96	Yes	9 12 14 96	Toilet/Kitch
5	10	Sometimes	1 2		7	Yes	1 4	Basin insid
7	7	Sometimes	1 2		8	Yes	1 5 9 12 14	Customise
4	6	Sometimes	1 2	3 8		Yes	1 4	Basin insid
998	3	Sometimes	1 2		3	Yes	1 2 3 4 9 1	Basin insid
0	7	Often	1 2 3 4		96	Yes	1 4 9 14	Basin insid
500	4	Sometimes	1 2 3 4		96	Yes	1 12 14	Beside the
2000	10	Sometimes	1 2 3 4	2 7		Yes	1 2 4 9 10	Toilet/Kitch
7	4	Sometimes	1 2 4	6 7		Yes	1 3 14	Customise
10	3	Sometimes	1 2 3 4	3 8		Yes	1 3 4 9 14	Basin insid
0	4	Sometimes	1 2 3 4	2 6		Yes	1 3 4 9 12	Customise
5	6	Sometimes	1 2		8	Yes	1 4 9 12 14	Customise
0	3	Sometimes	1 2		96	Yes	1 3 4 9 12	Basin insid
5	6	Sometimes	1 2		8	Yes	1 4 14	Customise
5	2	Never	1 2 4	3 6		Yes	1 2 3 4	Basin insid
	4	Never	1 2 3 4		2	Yes	1 3 4 9 12	Basin insid
350	3	Sometimes	1 2 3		3	Yes	1 3 4 9 12	Customise
5	2	Never	1 2 4		3	Yes	1 2 4 12 14	Basin insid
8	4	Never	1 2 4		96	Yes	1 4 9 12	Customise
350	3	Never	1 2 3 4		96	Yes	1 2 3 4 9 1	Beside the
15	2	Never	1 2		6	Yes	4 9	Basin insid
7	4	Sometimes	1 2 4		96	Yes	1 3 4 14	Basin insid
0	3	Sometimes	1 2 3 4		96	Yes	1 2 3 4 6 9	Customise
5	15	Sometimes	1 2		8	Yes	1 3 4 9 10	Customise
0	5	Often	1 2 3		3	Yes	1 3 12 14	Customise
0	3	Never	1 2		96	Yes	1 4	Customize

5	4	Sometimes	1 2		2	Yes		1 4 7 9 12	Customised
5	5	Sometimes	1 2 4		3	Yes		1 3 4 10 12	Basin insid
5	5	Sometimes	1 2		3	Yes		1 4 12	Basin insid
5	6	Sometimes	1 2		3	Yes		1 2 14 96	Basin insid
5	5	Sometimes	1 2		2	Yes		1 3 4 9 12	Customised
5	4	Sometimes	1 2		3	Yes		1 3 4 9 12	Basin insid
0	3	Sometimes	1 2 3		3	Yes		1 4 14	Customised
10	5	Never	1 2	3 8		Yes		1 4 9 14	Customised
5	3	Often	1 2 3 4		96	Yes		1 3 4	Basin insid
0	3	Often	1 2		7	No	sometimes	1 4	Basin insid
10	6	Sometimes	1 2		7	Yes		1 3 4	Basin insid
10	6	Sometimes	1 2		7	Yes		1 2 4 12 14	Customized
200	10	Sometimes	1 2 3 96		3	Yes		1 3 4 12	Basin insid
0	3	Sometimes	1 2 3		3	Yes		1 4 9 12 14	Customised
5	2	Never		2	3	Yes		3 4	Basin insid
5	5	Sometimes	1 2		7	Yes		1 3 5 8 9 1	Customised
0	2	Never		2	3	Yes		1 2 3 4	Basin insid
5	6	Often	1 2 3 4		3	Yes		1 4 9 12	Basin insid
0	2	Sometimes	1 2 4		96	Yes		1 4 9 14	Basin insid
0	5	Sometimes	1 2 3 4		96	Yes		1 3 4 9 12	Basin insid
7	2	Never	1 2 4	6 10		Yes		1 3 4	Customised
500	7	Sometimes	1 2 3		3	Yes		1 3 4	Toilet/Kitch
0	2	Never	1 2		96	Yes		1 3 12	Customised
10	2	Sometimes	1 2 3 4		3	No	I have no g	1 3 4 9 12	Basin insid
5	5	Sometimes	1 2		3	Yes		1 3 4 9 12	Basin insid
700	7	Sometimes	1 2 3 4		96	Yes		1 2 3 4 7 9	Customised
0	4	Never	1 2		1	Yes		1 3 4	Basin insid
5	6	Sometimes	1 2 4	3 8		Yes		1 3 4 12 14	Basin insid
5	2	Never		1	7	Yes		1 4 12 14	Customised
5	4	Sometimes	1 2		3	Yes		1 2 3 6 7 9	Basin insid
15	2	Sometimes	1 2		7	Yes		1 4 14	Basin insid
0	4	Never	1 2		96	Yes		1 4 14	Customised
0	5	Sometimes	1 2 4		6	Yes		1 4 6 12	Customized
10	2	Sometimes	1 2 3 4		3	Yes		1 3 4 9 10	Basin insid
0	6	Sometimes	1 2		96	Yes		1 2 7	Basin insid
6	6	Sometimes	1 2 4		96	Yes		1 3 4	Basin insid
100	4	Never	1 2 4		96	Yes		1 4 14	Customised
500	3	Never	1 2 3		3	No		1 4 12	Basin insid
5	4	Never	1 2		8	Yes		1 9 13 14	Customised
10	20	Sometimes	1 2 3		3	Yes		1 4	Basin insid
600	8	Sometimes	1 2 3 4		2	Yes		1 3 4 12 14	Customized
3	6	Sometimes	1 2		7	Yes		1 4 9 12 14	Basin in the
5	4	Sometimes	1 2 3		3	Yes		1 4 12	Basin insid
500	2	Sometimes	1 2 4		96	Yes		1 4 12	Customised
200	2	Never	1 2 3		96	Yes		1 4 12	Customized
600	6	Sometimes	1 2		96	Yes		1 4 12	Customised
500	10	Sometimes	1 2 3 4		2	Yes		1 3 4 14	Beside the
0	4	Sometimes	1 2 4		96	Yes		1 2 4 12	Customised
500	4	Sometimes	1 2		3	Yes		1 4 9 12 14	Customised

450	2	Sometimes	1 2 3		3	Yes		1 3 4 9 12	Other (Spe
5	2	Sometimes	1 2 3		3	Yes		1 4 12 14	Customised
0	4	Sometimes	1 2	4 6		Yes		1 3 4 9 14	Customised
150	2	Never	1 2 4		96	Yes		4 9 12	Customised
0	5	Sometimes	1 2		3	Yes		1 4 12	Customised
5	10	Sometimes	1 2 4	3 8		Yes		1 3 4 7 12	Basin insid
1500	4	Sometimes	1 2		96	Yes		1 4 9 12 14	Customised
0	6	Sometimes	1 2 3		96	Yes		1 2 12 14	Customized
5	5	Never	1 2	6 10		Yes		1 3 4 14	Basin insid
5	5	Sometimes	1 2		8	Yes		1 4 7 9 12	Customised
10	8	Sometimes	1 2		7	Yes		1 3 14	Customised
10	3	Sometimes	1 2 3	3 4 6		Yes		1 2 3 4 10	Basin in the
5	10	Never	1 2 3 4	3 7		Yes		1 12 14	Basin in the
5	10	Often	1 2 3		7	Yes		1 2 4 9 12	Customised
0	2	Sometimes	1 2 3		96	Yes		1 3 4 5 9	Basin insid
5	5	Never	1 2		8	Yes		1 4 12 14	Customised
5	5	Never	1 2	6 10		Yes		1 3 4	Customized
5	3	Never	1 2 4	2 6		Yes		1 3 4 14	Basin insid
5	7	Sometimes	1 2		7	Yes		1 4 9 12	Basin insid
5	5	Sometimes	1 2 3		7	Yes		1 4 96	Basin insid
5	10	Sometimes	1 2 3		2	Yes		1 4 9 96	Customised
500	3	Sometimes	1 2		3	Yes		3 4 9	Toilet/Kitch
0	5	Never	1 2 3		1	Yes		1 4 12	Toilet/Kitch
1000	4	Sometimes	1 2 3 4		6	Yes		1 3 4 12	Toilet/Kitch
0	1	Sometimes	1 2		96	Yes		1 4	Customised
600	2	Sometimes	1 2 3 4		3	Yes		1 4 9	Basin in the
500	1	Never	1 2 3		96	Yes		1 3 4 12 14	Toilet/Kitch
5	2	Sometimes	1 2 3 4		9	No		3 4 9	Basin insid
0	3	Sometimes	1 2 3		3	Yes		1 14	Basin insid
5	7	Sometimes	1 2 3		7	Yes		1 4 96	Basin insid
2500	8	Never	1 2 3		96	Yes		1 4 9 12	Beside the
5	3	Often	1 2	3 7		Yes		1 3 4	Basin insid
10	2	Sometimes	1 2	3 7		Yes		1 4 12	Beside the
5	3	Sometimes	1 2		8	Yes		1 3 4 9 12	Customised
	2	Sometimes	1 2		3	Yes		4 9 12 14 9	Basin insid
15	10	Sometimes	1 2 3 4	2 6		Yes		1 3 4 5 9 1	Customised
0	2	Sometimes	1 2 3 4	4 6		Yes		1 2 3 4 9 1	Basin insid
5	3	Sometimes	1 2 3		7	Yes		1 3 4 14	Basin insid
5	3	Never	1 2 3 4		7	Yes		3 4 9 14	Basin insid
20	8	Never	2 3 4		3	Yes		1 12	Customised
5	6	Sometimes	1 2		8	Yes		1 4 5 12 13	Customised
0	10	Sometimes	1 2		8	No	Feeling tire	1 12 14	Customised
500	3	Sometimes	1 2		7	Yes		1 4	Beside the
5	4	Sometimes	1 2 4		3	Yes		4 5 12 14	Customised
0	998	Sometimes	1 2		2	Yes		1 3 4 9 12	Customised
330	4	Never	1 2 3 4		96	Yes		1 3 9 12 14	Toilet/Kitch
0	2	Never	1 2		96	Yes		1 4 12 96	Customised
5	1	Sometimes	1 2	3 7 96		Yes		1 5 12 13	Basin in the
1000	5	Sometimes	1 2	2 6		Yes		1 4 9 12 14	Basin insid

0	5	Never	1 2 3 4		3	Yes		4 9 14	Basin insid
0	5	Sometimes	1 2 3		3	Yes		1 2 3 4 7 1	Customised
0	5	Sometimes	1 2		96	Yes		1 4 14	Customised
0	1	Often	1 2 3 4	3 96		Yes		1 3 4 12	Toilet/Kitch
500	4	Sometimes	1 2		3	Yes		1 2 4 8 12	Customised
5	7	Sometimes	1 2		96	Yes		1 4 12	Basin insid
10	6	Sometimes	1 2		5	Yes		1 3 4 9 12	Customised
5	3	Sometimes	1 2		3	Yes		1 4	Basin insid
10	6	Often	1 2 3 4		3	Yes		1 3 4 9 10	Basin insid
3000	10	Sometimes	1 2 3 4	6 7		No		1 2 3 4 6 7	Basin insid
5	4	Sometimes	1 2		3	Yes		1 4	Basin insid
0	3	Often	1 2 3		96	Yes		1 4 14	Toilet/Kitch
1000	8	Often	1 2 3	3 7		Yes		1 4	Basin in the
0	3	Often	1 2 3		96	Yes		1 3 4 12 96	Basin insid
1000	7	Sometimes	1 2		6	Yes		1 3 4 9 12	Customised
0	5	Sometimes	1 2 4		96	Yes		1 2 6	Customised
410	3	Sometimes	1 2		3	Yes		1 3 4 9 12	Customised
1000	10	Sometimes	1 2 3		96	Yes		1 4 12 14	Customised
0	4	Often	1 2 3		3	Yes		1 3 4 11 12	Basin insid
5	2	Sometimes	1 2	3 7 8		Yes		2 4	Basin insid
700	6	Sometimes	1 2	6 8		Yes		1 3 4 9 12	Basin in the
5	3	Sometimes	1 2		6	Yes		1 4 12 96	Basin insid
0	5	Sometimes	1 2		2	Yes		1 3 4 12 14	Customised
5	1	Sometimes	1 2		7	No	Am not use	4 9	Basin insid
0	1	Sometimes	1 2		3	Yes		1 3 9 12 14	Basin insid
400	5	Sometimes	1 2		96	Yes		1 4 9 12	Customised
0	2	Never	1 2 3 4		96	Yes		1 4 9 12 14	Basin insid
5	5	Sometimes	1 2		3	Yes		1 3 4 12 14	Basin insid
5	3	Often	1 2 3 4	3 8		Yes		1 3 7 12 14	Basin insid
7	3	Never	2 3 4	3 6		Yes		1 4 9 14	Basin insid
7	4	Sometimes	1 2 4		7	Yes		1 4 6 14	Customised
1500	4	Never	1 2 4	6 10		Yes		1 4 12	Basin insid
0	1	Never	1 2		3	Yes		1 4 12 96	Customised
0	3	Never	1 2		2	Yes		1 4	Customised
7	3	Sometimes	1 2		3	Yes		1 3 4 9 12	Basin insid
0	2	Sometimes	1 2		6	Yes		1 4 9 12 14	Customised
5	3	Sometimes	1 2		8	Yes		1 4 9 12 14	Customised
10	2	Sometimes	1 2		3	Yes		4 14 96	Customized
5	6	Sometimes	1 2		3	Yes		1 4 9 12 14	Basin insid
840	1	Sometimes	1 2 3 4		2	Yes		1 3 4 9 12	Basin insid
5	10	Sometimes	2		3	Yes		1 3 4 9 12	Basin in the
5	3	Sometimes	1 2 4		96	Yes		1 4 14	Customised
0	2	Sometimes	1 2 3 4	3 6		Yes		1 4 9 10 96	Basin insid
10	12	Never	1 2 4		6	Yes		1 2 3 12	Customised
5	4	Sometimes	1 2		3	Yes		1 4 12 14	Basin insid
0	2	Sometimes	1 2 3 4		3	Yes		1 4 9 13 14	Customised
5	5	Sometimes	1 2 4		8	Yes		1 3 4 12 14	Basin insid
20	998	Never	2		11	Yes		4 9 14	Other (Spe
5	8	Sometimes	1 2		7	Yes		1 4 14	Basin insid

7	6	Sometimes	1 2 3 4	3 6		Yes	1 3 4 9 12	Basin insid
5	4	Sometimes	1 2		8	Yes	1 3 4 9 12	Customised
10	5	Sometimes	1 2 3 4	4 7		Yes	1 2 4 9 12	Basin insid
5	998	Sometimes	1 2 3		3	Yes	1 3 4 8	Basin insid
5	3	Sometimes	1 2		10	Yes	1 2 3 4 9	Customised
220	3	Sometimes	1 2 3 4	3 7		Yes	1 3 4 9 10	Basin insid
5	3	Never	1 2 3 4	3 4 6		Yes	1 3 4 9 10	Basin insid
0	2	Sometimes	1 2		96	Yes	1 2 3 14 96	Customised
10	5	Sometimes	1 2 3 4		3	Yes	1 3 4 9	Basin in the
0	3	Sometimes	1 2 96	7 96		Yes	1 2 4 9	Customised
5	25	Sometimes	1 2 3 4 96	3 7		Yes	1 3 4 8 9	Basin in the
0	10	Sometimes	1 2		6	Yes	1 4 9 12 14	Customised
3	4	Sometimes	1 2 4		3	Yes	1 2 3 4 6 7	Basin insid
0	5	Never	2 3 4	2 7		Yes	1 3 4 9 12	Basin insid
10	2	Never	1 2 3 4		2	Yes	1 3 4 7 9 1	Basin insid
0	1	Often	1 2 3		96	Yes	1 4 9 14	Customised
5	4	Sometimes	1 2 3 4		6	Yes	1 2 4 9 12	Basin insid
5	5	Sometimes	1 2 4		3	Yes	1 3 4 5 9 1	Basin insid
0	4	Never	1 2 4		96	Yes	1 4 12 14	Customised
2	5	Never	1 2 3 4		3	Yes	1 4 12 14	Basin insid
10	5	Never	2		3	Yes	1 3 4 13	Basin insid
5	2	Often	1 2 3		96	Yes	1 2 3 4 9	Customised
10	2	Sometimes	1 2 3 4		96	Yes	1 4 7 9 12	Customised
0	2	Often	1 2 3 4		2	Yes	1 2 4 9 12	Basin insid
0	2	Sometimes	1 2		3	Yes	1 4 9 14 96	Basin insid
5	2	Often	1 2 3		96	Yes	1 4 96	Basin insid
0	9	Never	1 2	3 96		Yes	1 3 4 6 9 1	Customised
5	4	Sometimes	1 2		3	Yes	2 3 4 10	Basin insid
300	6	Never	1 2		2	Yes	1 3 4 9 12	Basin in the
10	5	Sometimes	1 2 4		96	Yes	1 4 9 14 96	Basin insid
5	5	Never	1 2 4		3	Yes	3 4 9 14	Basin insid
5	4	Sometimes	1 2		96	Yes	1 9 12	Basin insid
1000	998	Sometimes	1 2	6 96		Yes	1 4 9 12 14	Customised
0	5	Sometimes	1 2 3 4		3	Yes	1 3 4 9 14	Basin insid
5	2	Never	1 2 4		5	Yes	1 2 12	Basin insid
5	8	Sometimes	1 2 4		8	No	1 4	Customised
10	6	Sometimes	1 2 4		7	Yes	1 3 4 12	Customised
100	1	Sometimes	1 2 3 4	3 4		Yes	1 3 4 9 12	Basin insid
7	3	Sometimes	1 2		2	Yes	1 3 4	Basin insid
0	3	Sometimes	1 2 3		96	Yes	1 4 9 12	Customised
5	6	Sometimes	1 2		8	Yes	1 3 4 9 12	Customised
0	5	Sometimes	1 2	3 5		Yes	1 3 4 9 14	Customized
30	3	Never	2 3		3	Yes	4 12	Basin insid
5	5	Sometimes	2 3 4		3	Yes	1 3 4 9 14	Basin insid
4	5	Sometimes	1 2 4		3	Yes	3 9 12 14	Customized
5	2	Sometimes	1 2		96	Yes	1 14	Customised
0	2	Often	1 2 3		96	Yes	1 4	Basin insid
3000	998	Sometimes	1 2 3 4		96	Yes	1 2 4 14	Toilet/Kitch
0	6	Sometimes	1 2 4		3	Yes	1 4 6 7 12	Customised

500	10	Sometimes	1 2 3 4		8	Yes	1 12	Toilet/Kitch
5	10	Sometimes	1 2 3		7	Yes	1 4	Basin in the
7	5	Sometimes	1 2		8	Yes	1 3 9 12 14	Customised
4000	25	Sometimes	1 2 3 4	2 8		Yes	1 3 4 9 12	Toilet/Kitch
0	3	Never	1 2		96	Yes	3 4 12	Beside the
5	3	Sometimes	1 2 3 4	3 7		Yes	1 4 9 14	Basin in the
5	4	Sometimes	1 2 3 4		3	Yes	1 3 4 8 10	Basin insid
0	5	Sometimes	1 2 3		6	Yes	1 4 9 12 14	Basin insid
2000	10	Often	1 2 3	2 6		Yes	1 3 9 12 14	Toilet/Kitch
0	2	Never	1 2		2	Yes	3 4	Customised
5	3	Never	2		3	Yes	1 2 3 4 7 9	Customised
5	1	Never	1 2 4	6 10		Yes	1 3 4 14	Customized
5	6	Sometimes	1 2 3		3	Yes	1 3 4 9 12	Basin insid
300	2	Sometimes	1 2 3 4		3	No	1 4 5 12	Customised
5	4	Never	1 2		3	No	1 4	Basin insid
600	5	Sometimes	1 2		2	Yes	1 4 12 14	Basin insid
5	2	Never	1 2		7	Yes	4 9 14	Basin insid
10	3	Never	1 2 3		3	Yes	1 3 4 9 12	Customised
3	5	Sometimes	1 2		7	Yes	1 4 14	Customised
5	3	Never	1 2 3	3 7		Yes	3 4 9 10 14	Basin in the
10	4	Sometimes	1 2		3	Yes	1 4	Basin insid
0	2	Sometimes	1 2		2	Yes	1 4	Basin insid
5	2	Sometimes	1 2		7	Yes	1 3 4	Basin insid
1500	10	Sometimes	1 2 3 4		96	Yes	1 3 4 12 14	Customized
0	5	Often	1 2 3 4		2	Yes	1 4 14	Basin insid
150	5	Sometimes	1 2 3 96		96	Yes	1 3 4 9 12	Customised
5	12	Sometimes	1 2 3	3 7		Yes	1 4 9 12 96	Basin in the
5	6	Sometimes	1 2		1	Yes	1 3 4	Basin insid
5	3	Sometimes	1 2		3	Yes	1 6 7 96	Basin insid
10	6	Sometimes	1 2		3	Yes	3 4 5 14	Basin insid
5	2	Sometimes	1 2 4		3	Yes	1 3 4 9 12	Basin insid
5	6	Sometimes	1 2 4		3	Yes	1 2 4 6 12	Basin insid
3	3	Sometimes	1 2		7	Yes	1 4 9 96	Basin insid
3	4	Sometimes	1 2		96	Yes	1 4 96	Customised
0	5	Sometimes	1 2 3 4	2 3 8		Yes	1 2 3 4 7 9	Basin insid
10	3	Never	1 2 3 4		3	Yes	1 3 4 9 10	Basin in the
2	7	Sometimes	1 2		7	Yes	1 4 14 96	Basin in the
0	4	Sometimes	1 2 3 4		2	Yes	1 4 12 14	Customised
3000	5	Sometimes	1 2 3 4		6	Yes	1 3 4 9 12	Toilet/Kitch
5	5	Sometimes	1 2 3		3	No	1 4	Customised
5	8	Sometimes	1 2		8	Yes	1 3 12 14	Customised
5	6	Sometimes	1 2		2	Yes	1 4 7 9 12	Basin insid
0	2	Sometimes	1 2		96	Yes	1 2 4 9 14	Customised
10	10	Sometimes	2 3 4	3 6		Yes	1 2 3 4 6 7	Toilet/Kitch
0	6	Sometimes	1 2		96	Yes	1 4 9 14	Customised
5	3	Sometimes	1 2 3 4		3	Yes	1 3 4 9 14	Toilet/Kitch
5	20	Sometimes	1 2 3		11	Yes	4 9 12 14	Basin insid
500	5	Sometimes	1 2 3 4		3	Yes	1 4 14	Basin insid
6	11	Sometimes	1 2		7	Yes	1 3 9 14	Basin insid

4	4	Sometimes	1 2 4	3	Yes	1 3 4 12 14	Basin insid
0	7	Sometimes	1 2 4	96	Yes	1 2 4 7 9 1	Basin insid
6	4	Never	1 2	10	Yes	1 2 4 6	Customise
7	3	Sometimes	1 2	96	Yes	1 3 12	Customize
0	3	Never	1 2	96	Yes	1 3 4	Customise
200	3	Sometimes	1 2 3 4	3	Yes	1 3 4 9 12	Basin insid
500	4	Sometimes	1 2	7	Yes	1 4	Customise
10	3	Sometimes	1 2	10	Yes	1 4 14	Basin insid
0	5	Sometimes	1 2 3 4	3	No	4 9 12 14	Customise
5	3	Sometimes	1 2 4	7	Yes	1 4 10	Basin insid
500	5	Never	1 2 3 4	1	Yes	1 3 4 9 10	Basin insid
5	3	Never	1 2 3 4	7	Yes	3 4 9 14	Basin insid
5	3	Sometimes	1 2	7	Yes	1 3 14	Basin insid
5	3	Sometimes	1 2	3	Yes	4 6 9 12	Basin in the
5	6	Sometimes	1 2	3 7	Yes	1 4 9 14	Basin in the
5	4	Never	1 2	8	Yes	1 4 9 10 14	Customise
0	2	Never	1 2	2	Yes	1 4 5 12 14	Basin insid
0	3	Sometimes	1 2	6	Yes	1 3 4 9 12	Beside the
5	4	Never	1 2 4	6	Yes	1 3 4 7	Basin insid
10	10	Sometimes	1 2	8	Yes	1 4 9 12 13	Basin in the
5	6	Sometimes	1 2	8	Yes	1 3 9 14	Customise
0	3	Often	1 2 4	7 96	Yes	1 3 4 9 12	Toilet/Kitch
300	2	Never	1 2 3 4	3	Yes	1 4 12 14	Basin insid
3	3	Sometimes	1 2	3	Yes	1 3 12 14	Customise
0	10	Often	1 2 3 4	7 96	Yes	1 4 9	Basin insid

q35b	q36a	q36b	q37a	q38	q38b	q39a	q40a	q41
e the house	Same place where I w		1	1	2	9	7	Used only
e the house	Same place where I w		1	1	2	96	7	Used only
d Bucket or	Same place where I w		2 4	1	2	96	7	Used only
d Bucket or	Other (Spe	He is alone	96	1	2	96	7	Used only
e compound	Same place where I w		1	1	2	9 96	7	Used only
e the house	Same place where I w		4	1 4	2 6	9	7	Used only
e compound	Same place where I w		1	1	2	9	7	Used only
e the house	Same place where I w		1	1	2	96	7	Used only
e compound	Same place where I w		1	1	2	9	7	Used only
d Bucket or	Same place where I w		1 96	5 6	8	96	7	Used only
e the house	Other (Spe	Stays alone	1	1 4	2 6	96	7	Used only
e the house	Same place where I w		1 4	1	2	96	7	Used only
e compound	Same place where I w		1	1	2	9	7	Used only
e the house	Other (Spe	He lives alo	3	1	2	96	7	Used only
d Bucket or	Same place where I w		4	1	1	96	7	Used only
d bucket/ je	Same place where I w		1 4	2	3	5 8	1	Used only
e the house	Same place where I w		3	1 4	2 6	9	7	Used only
e the house	Same place where I w		1	3	4	9	3	Used only
d Bucket or	Same place where I w		1	3 4	2	9	7	Used only
d Bucket or	Same place where I w		96	1	2	9	7	Used only
d bucket/ je	Same place where I w		2	2 4	2	4	7	Used only
d Bucket or	Same place where I w		1 3	1 4	4 6	96	7	Used only
d bucket/ je	Same place where I w		2	1	1 5 6	4	7	Used only
e the house	Same place where I w		3	1	2 6 7	9	7	Used only
e the house	Same place where I w		1 2	1	2	96	7	Used only
e the house	Same place where I w		1	1	2	96	7	Used only
e the house	Same place where I w		1	1	2	9	7	Used only
d Bucket or	Other (Spe	I live alone	3	3 4	2	9	7	Used only
d Bucket or	Same place where I w		1	1	2	96	7	Used only
e compound	Same place where I w		1	1 4	2 6	9	7	Used only
d Bucket or	Same place where I w		96	1 5	2 6	96	7	Used only
d Bucket or	Same place where I w		3	1 4	2 6	9	7	Used only
e the house	Same place where I w		1 2 3 4	1	2	96	7	Used only
d Bucket or	Same place where I w		3	4	6	9	7	Used only
d Bucket or	Same place where I w		3	1 4	2 6	9	7	Used only
e compound	Same place where I w		3	1 4	2 6	9	7	Used only
e the house	Same place where I w		1 3	1 4	2 6	96	7	Used only
e compound	Same place where I w		1	4	6	9	7	Used only
d Bucket or	Same place where I w		1 3	1	2	9	1	Used only
d Bucket or	Same place where I w		1	1	2	9	7	Used only
d bucket/ je	Same place where I w		1 4	3 4	2 6	2	7	Used only
d Bucket or	Same place where I w		3	1	2	9	1	Used only
tap/ water :	They do not wash thei		2	2	3	96	2	Used only
e compound	Same place where I w		3	4	2 6	9	7	Used only
e the house	Same place where I w		1 2 3 4	1	2	96	7	Used only
e the house	Same place where I w		1 2 3 4	1	2	96	7	Used only
d Bucket or	Same place where I w		1	1 4	2 6	9	7	Used only
e the house	Same place where I w		3	1 4	2 6	9	7	Used only

d bucket/ je	Same place where I w		1		1		4	2	7	Used only
d Bucket or	Same place where I w		3	1 4		2 6		96	7	Used only
e compoun	Same place where I w		3	1 4		2 6		9	7	Used only
e the house	Same place where I w		1		1		2	96	7	Used only
e the house	Same place where I w		1	1 4			6	9	7	Used only
e compoun	Same place where I w		96		1		7	9	7	Used only
e the house	Same place where I w		1		1		2	9	7	Used only
e the house	Same place where I w		1		1		2	9	7	Used only
e the house	Same place where I w		1		1		2	9	7	Used only
e the house	Same place where I w	1 3			1		2	96	1	Used only
d Bucket or	Same place where I w		1		1		2	96	7	Used only
e the house	Same place where I w		1		1		2	9	7	Used only
d Bucket or	Same place where I w		2		1		2	8	7	Used only
e the house	Same place where I w		1		1		2	96	7	Used only
e compoun	Same place where I w		1		1	2 6		9	7	Used only
ien/sink insi	Same place where I w		2		2		2	1	7	Used only
e the house	Same place where I w	1 3			1		2	96	7	Used only
e the house	Same place where I w	2 4		2 4		2 6		9	7	Used only
e the house	Same place where I w	1 3			1		2	96	7	Used only
d Bucket or	Same place where I w	1 3			1		2	96	7	Used only
d Bucket or	Same place where I w		1		1		2	96	1	Used only
d bucket/ je	Same place where I w		2		3		2	96	7	Used only
e the house	Same place where I w	1 2 3 4		1 5		2 6		9	7	Used only
e the house	Same place where I w		1		1		2	9	7	Used only
e the house	Same place where I w	3 96		1 4		2 6		9	7	Used only
d Bucket or	Same place where I w		3		4		6	9	7	Used only
e compoun	Same place where I w		1		1		2	9	7	Used only
tap/ water	Same place where I w		4		1		2	5	7	Used only
e the house	Same place where I w		3	1 4		2 6		9	7	Used only
e compoun	Same place where I w		3		1		2	9	7	Used only
e the house	Same place where I w		1		1		2	9	7	Used only
d Bucket or	Same place where I w		1		1		2	9	7	Used only
d Bucket or	Same place where I w		1	1 4		2 6		9	7	Used only
d Bucket or	Same place where I w		1		1		2	96	2	Used only
d Bucket or	Same place where I w		1		1		2	9	7	Used only
e the house	Same place where I w	1 4			3		2	9	7	Used only
d Bucket or	Same place where I w		1		1		2	96	7	Used only
e compoun	Same place where I w		1		4	4 6		9	7	Used only
e the house	Same place where I w	1 3			1		2	96	7	Used only
e the house	Same place where I w		1		1		2	9	7	Used only
e the house	Same place where I w		1	1 4		2 6		9	96	Used only
d Bucket or	Same place where I w		2		1		1	3	7	Used only
e the house	Same place where I w		1		1		2	9	3	Used only
e compoun	Same place where I w		3		3		4	9	7	Used only
d Bucket or	Same place where I w		3		1		2	9	7	Used only
e the house	Same place where I w		1		1		3	96	7	Used only
d Bucket or	Same place where I w		4		1		5	4	7	Used only
tap/ water	Same place where I w		2		2	2 6		1	7	Used only
e the house	Same place where I w	1 3			1		1	96	7	Used only

d Bucket or	Same place where I w		1	1	1	96	7	Used only
d Bucket or	Other (Spe	No other fa	1	1	2	9	7	Used only
e the house	Same place where I w		1	3	2	96	7	Used only
e the house	Same place where I w		1	1	2	96	7	Used only
d Bucket or	Same place where I w		1 3	1	2	96	7	Used only
e the house	Same place where I w		1	1	6	96	2	Used only
e the house	Same place where I w		1	1	2	9	7	Used only
e the house	Same place where I w		1	1	2	96	7	Used only
e the house	Same place where I w		1	1	2	9	7	Used only
e compoun	Same place where I w		3	4	2 6	9	7	Used only
e the house	Same place where I w		1 2	1	2	96	7	Used only
d Bucket or	Same place where I w		4	1	2	9	7	Used only
d Bucket or	Same place where I w		1 2 4	1 4	4 6	4	7	Used only
e compoun	Same place where I w		2	1	2	9	7	Used only
d Bucket or	Same place where I w		1	1	2	9	7	Used only
en/sink insi	Same place where I w		96	1	2	96	7	Used only
e the house	Same place where I w		1	1	2	96	3	Used only
en/sink insi	Same place where I w		2	2	3	1	1	Used only
d Bucket or	Same place where I w		2 3	1	1	96	7	Used only
e compoun	Same place where I w		1 3	1	2	9	7	Used only
e the house	Same place where I w		1	1	2	96	7	Used only
d Bucket or	Same place where I w		1	1	2	9	7	Used only
d Bucket or	Same place where I w		1	4	6	9	7	Used only
tap/ water	Same place where I w		2	2	2	96	7	Used only
e compoun	Same place where I w		3	1 4	2 6	9	7	Used only
e the house	Same place where I w		1 3	1	2	96	7	Used only
d Bucket or	Same place where I w		1 3	1	2	9	7	Used only
d bucket/ je	Same place where I w		4	1	2	4	7	Used only
d Bucket or	Same place where I w		1 2 3 4	1 4	4 6	96	7	Used only
en/sink insi	Same place where I w		1	3 4	2 6	96	7	Used only
e compoun	Same place where I w		1	1	2	9	7	Used only
e the house	Same place where I w		1 2 3 4	1	2	96	7	Used only
e the house	Same place where I w		1	1 5	2	9	7	Used only
e the house	Same place where I w		1 3	1	2	96	7	Used only
e the house	Same place where I w		3	1 4	2 6	9	7	Used only
e the house	Same place where I w		1 2 3 4	1	2	96	7	Used only
d Bucket or	Same place where I w		1	3 5	2	96	7	Used only
e the house	Same place where I w		1	1 4	2	96	7	Used only
d Bucket or	Same place where I w		1	1	2	96	2	Used only
e compoun	Same place where I w		1	1	6	9	7	Used only
e compoun	Same place where I w		3	1 4	2 6	9	7	Used only
en/sink insi	Same place where I w		1 2 3 4	2 4	3 6	96	7	Used only
d Bucket or	Same place where I w		2 4	3 5	2 6	9	7	Used only
e the house	Same place where I w		1	1	2	9	7	Used only
d Bucket or	Same place where I w		1	1	2	96	7	Used only
e the house	Same place where I w		1	1	2	9	7	Used only
e the house	Same place where I w		1 3	1	2	9	2	Used only
e the house	Same place where I w		3	4	6	9	2	Used only
en/sink insi	Same place where I w		2	2 3 5	2	5	1	Used only

e the house	Same place where I w	3	1 4	1 2 6	9	7	Used only
e compound	Same place where I w	3	1 4	2 6	9	7	Used only
e the house	Same place where I w	1 3	1	2	96	7	Used only
e the house	Same place where I w	1	3 4	2 6	96	7	Used only
e the house	Same place where I w	1	1	2	9	7	Used only
e the house	Same place where I w	1 3	1	2	9	7	Used only
d Bucket or	Same place where I w	2	1	1	9	7	Used only
d Bucket or	Same place where I w	2	1	3	8	7	Used only
d Bucket or	Same place where I w	1	3 4	2	9	7	Used only
e the house	Same place where I w	1 2 3 4	1	2	96	7	Used only
e the house	Same place where I w	1	1	2	96	1	Used only
d Bucket or	Same place where I w	2 4	1	2	96	7	Used only
e the house	Same place where I w	3	1	2	9	7	Used only
e the house	Same place where I w	3	3 4	2 6	96	7	Used only
e the house	Same place where I w	1	4	6	9	7	Used only
d Bucket or	Same place where I w	1	1	1	96	7	Used only
d bucket/ je	Same place where I w	2	3 4	2	3	7	Used only
ıen/sink insi	Same place where I w	1	1	2	96	7	Used only
e the house	Same place where I w	1	1	2	9	1	Used only
e compound	Same place where I w	1	4	6	9	7	Used only
e the house	Same place where I w	96	1	2	96	7	Used only
e compound	Same place where I w	3	1 4	2 6	9	7	Used only
e the house	Same place where I w	1	1	2	9	7	Used only
e the house	Same place where I w	1	1	2	96	7	Used only
d Bucket or	Same place where I w	1	1	4 6	96	7	Used only
e the house	Same place where I w	1 3	1 4	2 6	96	7	Used only
e the house	Same place where I w	1	1	2	96	7	Used only
e the house	Same place where I w	1	1	2	9	7	Used only
d Bucket or	Same place where I w	3	4	6	9	7	Used only
e compound	Same place where I w	3	1	2	9	7	Used only
e the house	Same place where I w	1	1	2	9	7	Used only
e the house	Same place where I w	1	1	2	9	7	Used only
d Bucket or	Same place where I w	1	1	4	9	7	Used only
d Bucket or	Same place where I w	1	3	4	9	7	Used only
e compound	Same place where I w	1 3	1	2	9	7	Used only
e compound	Same place where I w	3	1 4	2 6	9	7	Used only
e the house	Same place where I w	3		1 2 6	96	7	Used only
e the house	Same place where I w	1 3	1	2	96	7	Used only
e the house	Same place where I w	1 2 3 4	1	2	96	7	Used only
e the house	Same place where I w	1	1	2	96	7	Used only
d Bucket or	Same place where I w	1	1	2	96	7	Used only
ıen/sink insi	Same place where I w	3	1	2	9	7	Used only
e the house	Same place where I w	3	1	2	96	7	Used only
e the house	Same place where I w	1 2 3 4	1	2	96	7	Used only
e the house	Same place where I w	3	1 4	2 6	9	1	Used only
e the house	Same place where I w	3	1 4	2 6	9	7	Used only
d Bucket or	Same place where I w	3	4	6	9	7	Used only
d Bucket or	Same place where I w	1 3	1 4	5 6	96	7	Used only
e compound	Same place where I w	3	4	6	9	7	Used only

d Bucket or	Same place where I w		1		1		2	96	7	Used only
e the hous	Same place where I w		1		1		2	9	7	Used only
d Bucket or	Same place where I w	1 3			1		1	96	7	Used only
d Bucket or	Same place where I w		1		4		6	9	1	Used only
e compoun	Same place where I w	1 3			1		2	9	7	Used only
e the hous	Same place where I w		1		1		2	96	1	Used only
d Bucket or	Same place where I w	1 2			1		2	96	7	Used only
e the hous	Same place where I w		3	1 4		2 6		9	7	Used only
e the hous	Same place where I w		1		5	2 6		9	7	Used only
d Bucket or	Same place where I w	1 3		1 4		4 6		96	7	Used only
en/sink insi	Same place where I w		2	2 4		2 6		96	7	Used only
tap/ water	Same place where I w		2		1		3	96	7	Used only
d Bucket or	Same place where I w	1 2 3		1 4		4 6		96	7	Used only
e the hous	Same place where I w		1		1		2	96	7	Used only
e the hous	Same place where I w		1		1		2	9	7	Used only
d Bucket or	Same place where I w		1	3 4			2	96	7	Used only
d Bucket or	Same place where I w		1		4		4	9	7	Used only
e the hous	Same place where I w	1 3		3 4		2 6		9	7	Used only
e the hous	Same place where I w		1	1 4		2 6		9	7	Used only
e the hous	Same place where I w		1		1		2	9	2	Used only
e the hous	Same place where I w		1		1		1	9	7	Used only
e the hous	Same place where I w	1 3		1 5		2 6		9	7	Used only
tap/ water	Same place where I w	1 2 4		1 4		3 6		96	7	Used only
d Bucket or	Other (Spe	No other fa	96		1		2	96	7	Used only
e the hous	Same place where I w		3	1 4		2 6		9	7	Used only
d Bucket or	Same place where I w	1 2 3 4		1 4		2 3 6		8	7	Used only
d Bucket or	Same place where I w		2	1 4		2 6		9	7	Used only
e the hous	Same place where I w		1		1		2	9	7	Used only
e the hous	Same place where I w	1 2 3 4			1		2	96	7	Used only
e the hous	Same place where I w	1 2 3 4		3 4		2 6		96	7	Used only
e the hous	Same place where I w		1		4		6	9	1	Used only
d Bucket or	Same place where I w		1		1		2	9	7	Used only
d Bucket or	Same place where I w	1 3		1 4		2 6		9	7	Used only
e the hous	Same place where I w		3	1 4		2 6		9	1	Used only
e the hous	Same place where I w	1 2		1 4		2 6		9	7	Used only
e compoun	Same place where I w	1 2 4			1		2	9	7	Used only
e compoun	Other (Spe	Na	1		1		2	9	7	Used only
e compoun	Same place where I w		3		1		2	9	7	Used only
d Bucket or	Same place where I w		1		1		2	9	7	Used only
e compoun	Same place where I w		1		1		2	9	2	Used only
tap/ water	Same place where I w	1 2 3 4			1		2	96	7	Used only
e compoun	Same place where I w		1		1		2	9	7	Used only
tap/ water	Same place where I w		4	2 4		5 6		96	7	Used only
e the hous	Same place where I w		1		1		2	9	7	Used only
e compoun	Same place where I w		1		1		2	9	7	Used only
d Bucket or	Same place where I w	1 3		3 5		2 6		9	7	Used only
e the hous	Same place where I w	1 3		1 5			2	96	7	Used only
e the hous	Same place where I w		2		2		2	1	7	Used only
d bucket/ je	Same place where I w		1		1		2	96	1	Used only

e the house	Same place where I w		3	1	2	96	7	Used only
ıen/sink insi	Same place where I w		2	2	2	1	7	Used only
e the house	Other (Spe	No other m	1	1	2	96	7	Used only
d Bucket or	Other (Spe	NA	1	1 4	2 6	9	7	Used only
e the house	Same place where I w		1	1	2	9	7	Used only
e the house	Same place where I w		3	1 4	2	9	7	Used only
e the house	Same place where I w		3	4	6	9	2	Used only
e the house	Same place where I w		1 3	1 5	2 6	9	7	Used only
e the house	Same place where I w		1 3	1	2	96	7	Used only
ıen/sink insi	Same place where I w		2	5	1	1	7	Used only
e the house	Same place where I w		1	1	1	96	7	Used only
d Bucket or	Same place where I w		2	1	5	96	7	Used only
d Bucket or	Same place where I w		1	1 4	4 6	96	7	Used only
e compound	Same place where I w		1	1	2	9	7	Used only
e the house	Other (Spe	N/A	1 2 3 4	1 5	2 6	9	7	Used only
e the house	Same place where I w		2 4	3 5	2 6	9	7	Used only
e the house	Same place where I w		3	4	6	9	7	Used only
d Bucket or	Same place where I w		1 3	1 4	4 6	96	7	Used only
e the house	Same place where I w		3	1	1 6	9	7	Used only
e the house	Other (Spe	I stay alone	1 2	1	2	96	1	Used only
d Bucket or	Same place where I w		96	1	2	96	7	Used only
e the house	Same place where I w		1	1	2	96	7	Used only
e the house	Same place where I w		3	1 4	2 6	9	7	Used only
e the house	Same place where I w		1	3	1	96	1	Used only
e the house	Same place where I w		1 3	1	2	96	7	Used only
e compound	Same place where I w		3	1 4	2 6	9	7	Used only
e the house	Same place where I w		1 2	1	1	96	7	Used only
e the house	Same place where I w		1	1	2	9	1	Used only
e the house	Same place where I w		1	1	2	96	1	Used only
d Bucket or	Same place where I w		1 3	1	2	96	7	Used only
d Bucket or	Same place where I w		1 2 3 4	1	2	96	7	Used only
e the house	Same place where I w		1	1	2	9	7	Used only
d Bucket or	Same place where I w		1	1 5	2 6	96	7	Used only
e the house	Same place where I w		3	4	2 6	9	7	Used only
d Bucket or	Same place where I w		1	1	2	9	7	Used only
e the house	Same place where I w		3	1	2 6	9	7	Used only
e the house	Same place where I w		1 3	1	2 6	9	7	Used only
e the house	Same place where I w		1	1	2	9	7	Used only
d Bucket or	Same place where I w		3 96	1 4	4 6	96	7	Used only
e the house	Same place where I w		3	1 4	2 6	9	7	Used only
e the house	Same place where I w		1	1	1	96	7	Used only
d Bucket or	Same place where I w		1	1	2	9	7	Used only
e the house	Same place where I w		1	1	2	96	7	Used only
e the house	Same place where I w		1	1	2	9	7	Used only
e the house	Same place where I w		1	1	2	9	7	Used only
e the house	Same place where I w		2	1	2	96	1	Used only
e the house	Same place where I w		1 3	1	2	96	7	Used only
e the house	Same place where I w		1 2 3 4	1	2	96	7	Used only
d Bucket or	Same place where I w		3	1 4	2 3 6	96	7	Used only

e the house	Same place where I w		1 3		3 4		2 6		96		7	Used only
d Bucket or	Same place where I w		1 2 3			1		1	96		7	Used only
e the house	Same place where I w			1	3 4			2	9		7	Used only
e the house	Same place where I w			1		1		2	96		7	Used only
d Bucket or	Same place where I w		1 3		1 4		2 6		96		7	Used only
d Bucket or	Same place where I w			1	2 4			2	96		7	Used only
e the house	Same place where I w			1		1		2	96		7	Used only
e the house	Same place where I w			1		1		2	9		7	Used only
e compoun	Same place where I w			3	1 4		2 6		9		7	Used only
d Bucket or	Same place where I w			1	1 4		2 4 6		96		7	Used only
e the house	Other (Spe	Lives alone	2 4		1 4		2 6		9		7	Used only
d Bucket or	Same place where I w			3	1 4		2 6		9		7	Used only
d Bucket or	Same place where I w			1		1		2	96		1	Used only
e the house	Same place where I w			3	1 4		2 6		9		7	Used only
e the house	Same place where I w			1		1		2	96		7	Used only
e compoun	Same place where I w			1	3 4			2	9		7	Used only
e the house	Same place where I w		1 3 4			1	3 6		96		7	Used only
e compoun	Same place where I w			1		1		2	9		7	Used only
e the house	Same place where I w			1	1 4		2 6		9		1	Used only
d Bucket or	Same place where I w			1		1		2	96		1	Used only
e the house	Same place where I w			1		1		2	9		7	Used only
d Bucket or	Same place where I w			96		1		2	96		7	Used only
e the house	Same place where I w		1 2 3 4			1		2	96		7	Used only
e compoun	Same place where I w		1 3			1		2	9		2	Used only
d Bucket or	Same place where I w			1		1		2	96		1	Used only
e the house	Same place where I w			1		1		2	96		96	Used soap
e the house	Same place where I w			1		1		2	96		1	Used soap
d Bucket or	Same place where I w		1 3			1		2	9		7	Used soap
hen/sink insi	Same place where I w		2 4			2		2	1		7	Used soap
e the house	Same place where I w		1 3			1		2	96		2	Used soap
tap/ water :	Same place where I w			2		2		2	1		3	Used soap
d bucket/ je	Same place where I w			3		1		1	96		1	Used soap
e the house	Same place where I w		1 3			1		2	96		1	Used soap
e the house	Same place where I w			1		1		2	96		7	Used soap
e compoun	Same place where I w		1 3			1		2	9		2	Used soap
d Bucket or	Same place where I w		1 3			1		2	9		2	Used soap
d Bucket or	Same place where I w			1	1 4		4 7		96		1	Used soap
d Bucket or	Same place where I w			1	1 4		4 7		96		1	Used soap
e the house	Same place where I w		1 3			1		2	96		1	Used soap
e the house	Same place where I w			1		1		2	96		1	Used soap
hen/sink insi	Same place where I w		2 4			2		2	1		1	Used soap
e the house	Same place where I w			1	3 5		2 6		96	1 3		Used soap
hen/sink insi	Same place where I w		1 4		2 4			2	1		7	Used soap
e compoun	Same place where I w			1		1		2	9		7	Used soap
e the house	Same place where I w		1 2 3 4			1		2	96		1	Used soap
e the house	Same place where I w		1 3			1		2	96		7	Used soap
e compoun	Same place where I w		1 3			1		2	9		7	Used soap
e the house	Same place where I w			1		1		2	96	1 2		Used soap
d Bucket or	Same place where I w			1		1		1	96		1	Used soap

d Bucket or	Same place where I w		1		1		2	96		1	Used soap
e the house	Same place where I w	1 3			1		2	96		2	Used soap
e the house	Same place where I w		1		1		2	96		1	Used soap
e the house	Same place where I w	1 2 3 4			1		2	96		1	Used soap
e the house	Same place where I w	1 2			1		2	96		1	Used soap
e the house	Same place where I w	2 4			3		2	2		7	Used soap
e the house	Same place where I w	1 3			1		2	96		1	Used soap
e the house	Same place where I w	1 4			1		2	96		1	Used soap
e the house	Same place where I w	1 2 3 4			1		2	96		3	Used soap
d Bucket or	Same place where I w		1		1		2	96		2	Used soap
Customize	Same place where I w	1 2		2 5			2	96		1	Used soap
e the house	Same place where I w		1		1		2	9		2	Used soap
e the house	Same place where I w		3		4	6 7		9		1	Used soap
e the house	Same place where I w		1		1		2	96		1	Used soap
e the house	Same place where I w	1 2			1		2	96		2	Used soap
e the house	Same place where I w		1	1 5		2 6		9		1	Used soap
e the house	Same place where I w	1 2 3 4			1		2	96		2	Used soap
d Bucket or	Same place where I w		1	1 5		2 6		96		1	Used soap
e the house	Same place where I w		1		1		2	96		1	Used soap
d Bucket or	Same place where I w		1	3 5			2	96		1	Used soap
d Bucket or	Same place where I w		1		1		2	96		1	Used soap
e the house	Same place where I w		1	1 5		2 6		96	1 3		Used soap
d Bucket or	Same place where I w	1 3		1 5		2 6		9		1	Used soap
e compoun	Same place where I w	1 2 3 4			1		2	9		1	Used soap
d Bucket or	Same place where I w		2	1 4		2 6		9		2	Used soap
d Bucket or	Same place where I w		1	1 5		2 6		96		1	Used soap
e the house	Same place where I w	1 2 4		3 5		2 7		9		2	Used soap
e compoun	Same place where I w		1		1		2	9		1	Used soap
e compoun	Same place where I w	1 3			1		2	9		1	Used soap
d Bucket or	Same place where I w	1 2			1		2	96		1	Used soap
e the house	Same place where I w		1		3		2	9	1 2		Used soap
e the house	Same place where I w	1 3		1 5		2 6		9		1	Used soap
e the house	Same place where I w		1		1		1	96		1	Used soap
e the house	Same place where I w		1	1 4			6	9		2	Used soap
e the house	Same place where I w		1		1		1	96		3	Used soap
d Bucket or	Same place where I w	1 2			1		2	96		1	Used soap
d Bucket or	Same place where I w	1 3		1 5		2 6		9		1	Used soap
tap/ water	Same place where I w		2	1 4			3	96		1	Used soap
e the house	Same place where I w		1		1		2	96		1	Used soap
e the house	Same place where I w		1		1		2	96		1	Used soap
e the house	Same place where I w		1		1		2	96		7	Used soap
e the house	Same place where I w	1 2			1		1	96		1	Used soap
d Bucket or	Same place where I w		1		1		2	96		1	Used soap
e the house	Same place where I w		1	1 4			2	9		1	Used soap
e the house	Same place where I w	1 2 3 4		3 5			2	9		2	Used soap
d Bucket or	Other (Spe	lives alone	1	3 4			3	9		7	Used soap
d Bucket or	Same place where I w		1	3 5			2	9		1	Used soap
e the house	Same place where I w		1	1 4		2 6		9		1	Used soap
d bucket/ je	Same place where I w		96	1 3 4			2	96		7	Used soap

e the house	Same place where I w		2	1	2	9	2	Used soap
e the house	Same place where I w		1	1	1	96	1	Used soap
e compound	Same place where I w		1	3 5	2	9	1	Used soap
d Bucket or	Same place where I w		1	1 5	2 6	96	1 3	Used soap
en/sink insi	Same place where I w		2	2	2 6	1	1	Used soap
d Bucket or	Same place where I w		1	1 5	2 6	96	1	Used soap
en/sink insi	Same place where I w		2	1 2	2	1	2	Used soap
en/sink insi	Same place where I w		4	2	2	1	2	Used soap
e the house	Same place where I w		1	3 5	2	9	1	Used soap
e the house	Same place where I w		1 2 3 4	1	2	96	7	Used soap
d Bucket or	Same place where I w		1 3	3 5	2 6	9	1	Used soap
d Bucket or	Same place where I w		1 2	1 4	4 7	96	2	Used soap
e the house	Same place where I w		1 2 4	1	2	96	7	Used soap
e the house	Same place where I w		3	1	2	9	3	Used soap
d Bucket or	Same place where I w		1 3	3 5	2 6	9	1	Used soap
d Bucket or	Same place where I w		1 3	3 5	2 6	9	1	Used soap
d bucket/ je	Same place where I w		2 4	3	2 7	8	2	Used soap
e the house	Same place where I w		1 2	1	2	96	1	Used soap
e the house	Same place where I w		1	1 4	2 6	9	1	Used soap
d Bucket or	Same place where I w		1	1	2	96	1	Used soap
e the house	Same place where I w		1	1	2	96	7	Used soap
e the house	Same place where I w		2	1	2	96	7	Used soap
d bucket/ je	Same place where I w		4	2	2	8	1	Used soap
e the house	Same place where I w		1 96	1 3 4	2	96	1	Used soap
e the house	Same place where I w		1	1	2	96	1	Used soap
e the house	Same place where I w		1 3	1 5	2 6	9	1	Used soap
d Bucket or	Same place where I w		1 2	1	2	96	1	Used soap
e compound	Same place where I w		1	1 4	4 7	96	1	Used soap
e the house	Same place where I w		1	1	2	96	1	Used soap
tap/ water	Same place where I w		1	1	2	96	1	Used soap
en/sink insi	Same place where I w		1 2	2 5	2 6	1	1	Used soap
Handwash	Same place where I w		2	2 5	2	2	1	Used soap
e the house	Same place where I w		1	1 4	2 6	9	1	Used soap
e the house	Same place where I w		1	1 5	2 6	9	2	Used soap
e the house	Same place where I w		1	1 4	2 6	9	1	Used soap
e the house	Same place where I w		1	1	2	96	1	Used soap
e compound	Same place where I w		1	1	2	96	7	Used soap
tap/ water	Same place where I w		4	1	2	96	1	Used soap
e the house	Same place where I w		1	1 4	2 6	9	1	Used soap
e the house	Same place where I w		1	1	2	9	1	Used soap
e the house	Same place where I w		1	1 4	2 6	9	1	Used soap
e the house	Same place where I w		1	1 4	2	96	1	Used soap
d Bucket or	Same place where I w		2 4	3 5	2 7	9 96	1	Used soap
e the house	Same place where I w		1	1 4	2 6	9	96	Used soap
e the house	Other (Spe	Lives alone	1 3	1 5	2	9	1	Used soap
d Bucket or	Same place where I w		2 4	3	5	8	1	Used soap
d bucket/ je	Same place where I w		2	2	2	8	1	Used soap
e the house	Same place where I w		1	1	2	96	2	Used soap
e the house	Same place where I w		1	1	2	96	1	Used soap

d Bucket or	Same place where I w		1		3		2		9		1	Used soap
d bucket/ je	Same place where I w		2	3 5		2 6			2	1 3		Used soap
d Bucket or	Same place where I w	1 3		1 5		2 6			96		1	Used soap
e the house	Same place where I w		1		1		2		96		1	Used soap
e the house	Same place where I w		1		1		3		96		1	Used soap
e the house	Same place where I w		1	1 4		2 6			9		1	Used soap
d Bucket or	Same place where I w	1 3			1		2		96		1	Used soap
d Bucket or	Same place where I w		1		1		2		96		7	Used soap
e the house	Same place where I w		1	3 5		2 6			96		1	Used soap
e the house	Same place where I w	1 2			1		2		96		1	Used soap
ien/sink insi	Same place where I w		1		1		3		96		1	Used soap
e the house	Same place where I w		1		1		2		96		3	Used soap
e compoun	Same place where I w	1 2 3 4			1		2		9		2	Used soap
d Bucket or	Same place where I w	1 3			1		2		96		1	Used soap
e the house	Same place where I w	1 3		1 4		2 6			96		1	Used soap
ien/sink insi	Same place where I w	2 4			2	2 6			1		7	Used soap
e the house	Same place where I w		1		1		2		9		2	Used soap
e the house	Same place where I w		1	1 4		2 6			9		1	Used soap
e compoun	Same place where I w	1 3			1		2		9		1	Used soap
e the house	Same place where I w		1		1		2		9		2	Used soap
e the house	Same place where I w		1		1		6		9		1	Used soap
d Bucket or	Same place where I w	1 3			1		2		96		3	Used soap
e the house	Same place where I w		1		5		4		3		3	Used soap
d Bucket or	Same place where I w		1		1		2		96		7	Used soap
d Bucket or	Same place where I w		1		1		2		96		1	Used soap
e the house	Same place where I w		1	1 4		2 6			96		1	Used soap
e the house	Same place where I w		1		1		2	5 9			2	Used soap
e the house	Same place where I w	1 3			1		2		96		1	Used soap
d Bucket or	Same place where I w		1		2		1		8		7	Used soap
d Bucket or	Same place where I w	1 3			1		2		96		1	Used soap
d Bucket or	Same place where I w	1 2			1		2		96		1	Used soap
e the house	Same place where I w		1	1 4		2 6			9		1	Used soap
d Bucket or	Same place where I w		1	1 5		2 6			96		1	Used soap
d bucket/ je	Same place where I w		1	2 5			2		4		1	Used soap
e the house	Same place where I w		1		1		2		96		1	Used soap
tap/ water	Same place where I w	1 2 3 4		1 2			3		96		7	Used soap
e the house	Same place where I w		1		1	2 7			96		1	Used soap
d Bucket or	Same place where I w	2 4		1 4		3 4 7			96		1	Used soap
e the house	Same place where I w		1		1		2		9		1	Used soap
d Bucket or	Same place where I w		1	1 5		2 6			96		1	Used soap
ien/sink insi	Same place where I w	2 4		2 5			2		1		2	Used soap
d Bucket or	Same place where I w	1 3		1 4		3 7			96		2	Used soap
e the house	Same place where I w	1 3			1		2		9		2	Used soap
d Bucket or	Same place where I w		1		1		2		96		1	Used soap
d Bucket or	Same place where I w		96	1 5		2 6			96		1	Used soap
d bucket/ je	Same place where I w		2	2 5		2 6			2		96	Used soap
e the house	Same place where I w	1 3			1		6		9		7	Used soap
ien/sink insi	Same place where I w	2 4		2 5			2		1		1	Used soap
d Bucket or	Same place where I w		1	1 4		2 6			9		1	Used soap

d Bucket or	Same place where I w		1	1	2	96		7	Used soap
e the house	Same place where I w		1	1	2	96		2	Used soap
e the house	Same place where I w		2 1 4	2 6		9		1	Used soap
e the house	Same place where I w		1	1	2	96		1	Used soap
e compound	Same place where I w		1 3 5		2	9		1	Used soap
e the house	Same place where I w		1	4	6	9		7	Used soap
e the house	Same place where I w	1 96		1	2	9		2	Used soap
e compound	Same place where I w	1 3		1	2	9		2	Used soap
e the house	Same place where I w		1 3 5	2 6		9		1	Used soap
e the house	Same place where I w		1	1	6	9		7	Used soap
d Bucket or	Same place where I w	1 2		3	4	8		1	Used soap
e the house	Same place where I w		1	1	2	96		1	Used soap
d Bucket or	Same place where I w		1 1 5	2 6		96		2	Used soap
e the house	Same place where I w		1	5	2	9		3	Used soap
d Bucket or	Same place where I w		1	1	2	96		1	Used soap
e the house	Same place where I w		1	1	2	96		1	Used soap
ien/sink insi	Same place where I w		4	2	2	96	2 96		Used soap
ien/sink insi	Same place where I w	2 4		2	2	1		1	Used soap
d Bucket or	Same place where I w		3	1	2	96		1	Used soap
d Bucket or	Same place where I w		1 1 5	2 6		96	1 2 3		Used soap
e the house	Same place where I w		1 1 4	2 6		9		1	Used soap
d Bucket or	Same place where I w		1	1	2	96		7	Used soap
d Bucket or	Same place where I w	1 3		1	2	96		7	Used soap
e the house	Same place where I w		1	1	2	96		2	Used soap
e the house	Same place where I w		1 1 4	2 6		9		1	Used soap
e the house	Same place where I w		1	1	2	96		1	Used soap
e the house	Same place where I w		1	1	2	9		2	Used soap
e the house	They do not wash thei		1 1 4	2 6		9		1	Used soap
d Bucket or	Same place where I w		2	2	2	5		1	Used soap
d Bucket or	Same place where I w		1 1 5	2 6		96		1	Used soap
e the house	Same place where I w		1	1	2	96		1	Used soap
d Bucket or	Same place where I w	1 2	1 4	2 6		9		1	Used soap
d Bucket or	Same place where I w	1 3	1 5	2 6		9		1	Used soap
e the house	Other (Spe	N/A	1 3 5	2 6		9		1	Used soap
e the house	Same place where I w		3	5 2 6		9		2	Used soap
d Bucket or	Same place where I w		1 1 2	2 6		9		1	Used soap
e the house	Same place where I w		1 1 4	2 6		96		1	Used soap
d Bucket or	Same place where I w		1 1 5	2 6		96		1	Used soap
d Bucket or	Same place where I w		1 3 5		2	9		1	Used soap
e the house	Same place where I w		1 1 4	2 6		9		1	Used soap
ien/sink insi	Same place where I w	2 4	2 5		2	1		2	Used soap
e the house	Same place where I w		1	1	6	9		1	Used soap
e compound	Other (Spe	I live alone	1 3 5		2	9		1	Used soap
d Bucket or	Same place where I w	2 4	1 4		4	2		1	Used soap
d Bucket or	Same place where I w	1 2		3	4	8		1	Used soap
ien/sink insi	Same place where I w		2	2	2	1		2	Used soap
e the house	Other (Spe	NA	1 1 4	2 6		9		1	Used soap
d bucket/ je	Same place where I w		1	1	2	2		2	Used soap
d Bucket or	Same place where I w	1 3		3	2	96		7	Used soap

e the house	Same place where I w	1	1 4	6	9	7	Used soap
e the house	Same place where I w	1	3 5	2	96	1	Used soap
d Bucket or	Same place where I w	1	3 5	2	96	1	Used soap
d Bucket or	Same place where I w	1	1	2	96	7	Used soap
d Bucket or	They do not wash thei	1 2 4	1 4	4 7	96	1	Used soap
d Bucket or	Same place where I w	1 3	1	1	96	3	Used soap
e the house	Same place where I w	1	1	2	96	1	Used soap
e the house	Same place where I w	1	1 4	2 6	96	1	Used soap
e the house	Same place where I w	1	1	2	9	2	Used soap
d bucket/ je	Same place where I w	2	2 5	2	2	1	Used soap
nen/sink insi	Same place where I w	2 4	2 5	2	1	1	Used soap
d Bucket or	Same place where I w	1	1 4	2 5	9	7	Used soap
e compound	Same place where I w	1 3	1	2	9 96	3	Used soap
e the house	Same place where I w	1	1	2	96	1	Used soap
nen/sink insi	Same place where I w	2	2	2	1	2	Used soap
e the house	Same place where I w	1 3	1 5	2 6	9	1	Used soap
d Bucket or	Same place where I w	1	1 4	4 7	96	1	Used soap
d bucket/ je	Same place where I w	2	3	2	3	7	Used soap
e compound	Same place where I w	1	5	2	9	2	Used soap
d Bucket or	Same place where I w	1	1 3	2	96	2	Used soap
d Bucket or	Same place where I w	1 2	1 5	2 6	96	1	Used soap
d Bucket or	Same place where I w	1 3	1	2	9	2	Used soap
nen/sink insi	Same place where I w	1	1 4	2 6	9	1	Used soap
d Bucket or	Same place where I w	1	1	2	96	1	Used soap
e the house	Same place where I w	1	4	6	9	7	Used soap
d Bucket or	Same place where I w	1	1	4	96	7	Used soap
d Bucket or	Same place where I w	1 3 4	1	2	9	2	Used soap
d bucket/ je	Same place where I w	1	1 5	2 6	96	2	Used soap
d Bucket or	Same place where I w	1	1	2	9	1	Used soap
tap/ water	Same place where I w	2	3 4	2	5	7	Used soap
d Bucket or	Same place where I w	1	3 4	2	96	1	Used soap
e the house	Same place where I w	1 3	1 5	2 6	96	1	Used soap
d Bucket or	Same place where I w	1	3 5	2	9	2	Used soap
e compound	Same place where I w	1 3	1	2	9	1	Used soap
e the house	Same place where I w	1	1 3 5	2	96	1	Used soap
d Bucket or	Same place where I w	1	1 5	2 6	96	1 2	Used soap
e the house	Same place where I w	1 3	1	1	96	2	Used soap
d Bucket or	Same place where I w	2 4	1 5	2 6	9	1	Used soap
e the house	Same place where I w	1	1	2	96	1	Used soap
tap/ water	Same place where I w	1	4	6	96	7	Used soap
d Bucket or	Same place where I w	1 3	1	2	96	1	Used soap
e compound	Same place where I w	1 3	1	2	9	3	Used soap
e the house	Same place where I w	1	1	2	96	1	Used soap
d Bucket or	Same place where I w	1	1	2	96	1	Used soap
e the house	Same place where I w	1	1	2	96	1	Used soap
nen/sink insi	Same place where I w	1	1	2 6	9	3	Used soap
d Bucket or	Same place where I w	1 3	1	2	96	1	Used soap
e the house	Same place where I w	1	1	2	96	1	Used soap
tap/ water	Same place where I w	1	1	2	96	1	Used soap

Sink outsid	Same place where I w	2 4		1 4		3 7		1		2	Used soap
d Bucket or	Same place where I w	1 3		1 5		2 6		9		1	Used soap
e the house	Same place where I w		96		1		4	96		2	Used soap
d Bucket or	Same place where I w	1 3			1		2	96		1	Used soap
e the house	Same place where I w	1 3		1 5		2 6		9		1	Used soap
e the house	Same place where I w		1		1		2	96	1 2		Used soap
d Bucket or	Same place where I w		1	1 5		2 6		96		1	Used soap
d Bucket or	Same place where I w	1 3			1		2	96		7	Used soap
ıen/sink insi	Same place where I w		1	1 4		2 6		9		2	Used soap
e the house	Same place where I w		1	2 4		2 6		9		2	Used soap
d Bucket or	Same place where I w	2 4			3		2	2		7	Used soap
e the house	Same place where I w		1	1 5		2 6		9		7	Used soap
ıen/sink insi	Same place where I w	1 2 3 4			2		3	1		1	Used soap
e compound	Same place where I w		1		3		2	9		1	Used soap
d Bucket or	Same place where I w	1 2 4		1 4		4 7		96		1	Used soap
d Bucket or	Same place where I w	1 3		1 5		2 6		9		1	Used soap
e the house	Same place where I w		1		1		2	96		1	Used soap
d Bucket or	Same place where I w		1		1		2	96		1	Used soap
e the house	Same place where I w		1		1		2	9		3	Used soap
e the house	Same place where I w		1	1 3 5			2	96		2	Used soap
d Bucket or	Same place where I w		1		1		2	96		1	Used soap
ıen/sink insi	Same place where I w		2		2		2	96		2	Used soap
d Bucket or	Same place where I w		96		1		2	9		2	Used soap
e the house	Same place where I w		1		1		2	96		1	Used soap
e the house	Same place where I w		1		1		2	9		1	Used soap
d Bucket or	Same place where I w	1 3			1		2	9		7	Used soap
e the house	Same place where I w		1		1		2	9		2	Used soap
d Bucket or	Same place where I w	1 3			1		2	96		1	Used soap
e the house	Same place where I w	1 2 3			1		2	9		1	Used soap
tap/ water :	Same place where I w	1 2 3 4			1		2	9	2 3		Used soap
e the house	Same place where I w		1	3 4			2	96		1	Used soap
e the house	Same place where I w		1		1		2	9		1	Used soap
d Bucket or	Same place where I w	1 3			3		4	2		1	Used soap
ıen/sink insi	Same place where I w		2		2		2	1		1	Used soap
e the house	Same place where I w		1		1		2	96		1	Used soap
e the house	Same place where I w		96	1 5		2 6		96		1	Used soap
e the house	Same place where I w	1 3		1 3 5			2	9		1	Used soap
e the house	Same place where I w		1	3 5			2	96		1	Used soap
e the house	Same place where I w		1	1 5		2 6		96		96	Used soap
d Bucket or	Same place where I w		1		1		2	96		1	Used soap
ıen/sink insi	Same place where I w		2		1		2	96		7	Used soap
ıen/sink insi	Same place where I w		2	1 5		2 6		96	1 2		Used soap
d Bucket or	Same place where I w		2	1 4		3 7		96		2	Used soap
d Bucket or	Other (Spe	NA	1	1 4		2 6		9		1	Used soap
e compound	Same place where I w	1 3		3 5			2	9		3	Used soap
e the house	Same place where I w		1		1		2	96		1	Used soap
e compound	Same place where I w		1		3		2	9		1	Used soap
e the house	Same place where I w	1 3		1 5		2 6		9		1	Used soap
e the house	Same place where I w		1		1		2	96		1	Used soap

d bucket/ je	Same place where I w		2	3 5	2 6	3	1	Used soap
tap/ water	Same place where I w		2 4	1	3 7	1	1	Used soap
e compoun	Same place where I w		1 4	1	7	9	1	Used soap
e the house	Same place where I w		1	1	2	96	2	Used soap
e the house	Same place where I w		1	1	2	9	2	Used soap
ien/sink insi	Same place where I w		4	2	2	1	1	Used soap
d Bucket or	They do not wash thei		96	1	2	96	2	Used soap
ien/sink insi	Same place where I w		2	3 5	2	1 96	1	Used soap
d Bucket or	Same place where I w		1 2	1	2	96	1	Used soap
d Bucket or	Same place where I w		1 2	1 4	4 6	96	1	Used soap
e the house	Same place where I w		1	1	2	9	2	Used soap
d Bucket or	Same place where I w		1 3	1 5	2 6	9	1	Used soap
d Bucket or	Same place where I w		1	3 5	2	9	1	Used soap
d Bucket or	Same place where I w		1 3	1 5	2 6	9	1	Used soap
e compoun	Same place where I w		1 2	1 4	4 7	96	1	Used soap
d Bucket or	Same place where I w		1 2	1	2	96	1	Used soap
d Bucket or	Same place where I w		1	3	2	96	7	Used soap
d Bucket or	Same place where I w		1 3	1	2	9	1	Used soap
e the house	Same place where I w		1	1	2	96	1	Used soap
tap/ water	Same place where I w		2	2	3	1	7	Used soap
e compoun	Same place where I w		1	1 4	2 6	9	2	Used soap
e the house	Same place where I w		2 4	1 5	4 6	96	7	Used soap
d bucket/ je	Same place where I w		2	3	2	4	7	Used soap
e the house	Same place where I w		1	1 5	2 6	96	1	Used soap
d bucket/ je	Same place where I w		2	1	4 7	6	1	Used soap
ien/sink insi	Same place where I w		1 2 3 4	2	2	4	7	Used soap
e the house	Other (Spe	Living alon	1	1 5	2 6	96	1	Used soap
d Bucket or	Same place where I w		1	1	2	96	2	Used soap
e the house	Same place where I w		1 2 3	1	3 4 6	9	1 2	Used soap
e the house	Same place where I w		1	2	2	96	1	Used soap
d Bucket or	Same place where I w		1 2	1	2	96	1	Used soap
e the house	Same place where I w		1	1	2	9	2	Used soap
e the house	Same place where I w		1	1	2	96	1	Used soap
e the house	Same place where I w		1	1	2	9	3	Used soap
d bucket/ je	Same place where I w		2	1	2	96	7	Used soap
e the house	Same place where I w		1	1	2	9	7	Used soap
d Bucket or	Same place where I w		1	4	6	96	1	Used soap
e the house	Same place where I w		1	1 4	2 6	9	1	Used soap
e the house	Same place where I w		1	3 5	2	9	1	Used soap
d Bucket or	Same place where I w		1 2	3	3	2	1	Used soap
e the house	Same place where I w		1	3 5	2 6	96	2	Used soap
e compoun	Same place where I w		1 3	1 5	2 6	96	1	Used soap
e the house	Same place where I w		2	3 5	2 6	96	1	Used soap
ien/sink insi	Same place where I w		1 2 3 4	2	3	1	1	Used soap
d Bucket or	Same place where I w		1 3	1	2	96	1	Used soap
d Bucket or	Same place where I w		1 2	1	2	96	1	Used soap
e the house	Same place where I w		1 2 3 4	1	2	96	1	Used soap
ien/sink insi	Same place where I w		1 3	2 5	2	1	1	Used soap
ien/sink insi	Same place where I w		2	2	2	1	1	Used soap

e the house	Same place where I w			1		1		2	9		1	Used soap
e the house	Same place where I w		1 2			1		2	96		1	Used soap
e the house	Same place where I w			1		1		2	9		2	Used soap
e the house	Same place where I w		1 2			1		2	96		1	Used soap
d Bucket or	Same place where I w		1 3			1		2	9		1	Used soap
e the house	Same place where I w			1		1		2	96		1	Used soap
d Bucket or	Same place where I w		1 3		1 4		5 6		96		7	Used soap
e the house	Same place where I w		1 2 3 4			1		2	96		7	Used soap
e compoun	Same place where I w			1		1		2	96		1	Used soap
e the house	Same place where I w			96	1 5		2 6		96		1	Used soap
d Bucket or	Other (Spe	Living alon		1	1 5		2 6		96		1	Used soap
e the house	Same place where I w			1		1		2	96		1	Used soap
d bucket/ je	Same place where I w		2 4		3 5			2	6	1 2		Used soap
ıen/sink insi	Same place where I w			2	2 5		2 6		1		1	Used soap
e the house	Same place where I w			1		1		1	96		1	Used soap
d Bucket or	Same place where I w		1 3			1		2	96		7	Used soap
e the house	Same place where I w		1 4			1		2	96		1	Used soap
e the house	Same place where I w			1		1		1	96		1	Used soap
e compoun	Other (Spe	I leave alor		1	3 5			2	9		1	Used soap
d bucket/ je	Same place where I w			2		1		2	96		1	Used soap
e the house	Same place where I w			1		1		2	96		7	Used soap
tap/ water :	Same place where I w			2		2		3	1		1	Used soap
tap/ water :	Same place where I w			2		1		3	96		2	Used soap
ıen/sink insi	Same place where I w			1		2		2	96		1	Used soap
d Bucket or	Same place where I w		1 3		1 5		2 6		9	1 2		Used soap
e the house	Same place where I w		1 3		1 5		2 6		9		1	Used soap
e the house	Same place where I w			3		1	2 6		9		1	Used soap
e the house	Same place where I w			1	3 5			2	9		1	Used soap
d Bucket or	Same place where I w			1	1 5		2 6		96		1	Used soap
d Bucket or	Same place where I w			96	1 5		2 6		96		2	Used soap
e the house	Same place where I w			1	1 4		2 6		9		1	Used soap
d Bucket or	Same place where I w			3		1		2	96		7	Used soap
ıen/sink insi	Same place where I w			2	1 5		2 6		1		1	Used soap
d Bucket or	Same place where I w		1 3		1 5		2 6		9		1	Used soap
d bucket/ je	Same place where I w			1		1	3 6		96		7	Used soap
ıen/sink insi	Same place where I w			2		2		2	1	1 2		Used soap
e the house	Same place where I w		1 2 3 4			1		2	96		7	Used soap
e the house	Same place where I w			3		1		7	9		1	Used soap
e compoun	Same place where I w			3	1 4		2 6		9		2	Used soap
ıen/sink insi	Same place where I w			1		1		2	9		1	Used soap
d Bucket or	Same place where I w			1		1		2	96		7	Used soap
ıen/sink insi	Same place where I w		1 2 3 4		2 5			2	9		2	Used soap
d Bucket or	Same place where I w			1		1		2	96		1	Used soap
d Bucket or	Same place where I w		2 4		1 4		3 6		96		7	Used soap
d Bucket or	Same place where I w			2	3 5			2	96	1 2		Used soap
d Bucket or	Same place where I w		1 3			1		2	9		2	Used soap
d Bucket or	Same place where I w		2 4		1 4			3	4		1	Used soap
d Bucket or	Same place where I w			1	1 3			2	96		1	Used soap
d Bucket or	Same place where I w		2 4			1		2	96		7	Used soap

e the house	Same place where I w		1 3 5		2	9		1	Used soap
e the house	Same place where I w		1 1 5	2 6		96		1	Used soap
e the house	Same place where I w		1 2 5	2 6		3		2	Used soap
e the house	Same place where I w	1 2 3 4		1 2 8		96		1	Used soap
e the house	Same place where I w		1	1	2	9		2	Used soap
e the house	Same place where I w		1	1	2	9		2	Used soap
d Bucket or	Same place where I w		96	4	4	96		7	Used soap
d Bucket or	Same place where I w		3	1	2	96		1	Used soap
e the house	Same place where I w	1 3		1	2	96		7	Used soap
e the house	Same place where I w		1 1 4	2 7		9		1	Used soap
e the house	Same place where I w		1	1	3	96		7	Used soap
d Bucket or	Same place where I w	1 2		1	2	96		2	Used soap
d Bucket or	Same place where I w		1 4 5		7	9		2	Used soap
e the house	Same place where I w		1 2 5	2 7		9		1	Used soap
e the house	Same place where I w	1 3		1	2	96		1	Used soap
e the house	Same place where I w	1 2		1	2	3		1	Used soap
e compound	Same place where I w	1 3		1	2	9		3	Used soap
e the house	Same place where I w		1	1	2	96		1	Used soap
e the house	Same place where I w	1 3	1 5	2 6		9		1	Used soap
d Bucket or	Same place where I w		1 3 5		2	96		1	Used soap
d Bucket or	Same place where I w		3	1	2	9	1 3		Used soap
e the house	Same place where I w	1 2 4	3 5	2 6		96		1	Used soap
d Bucket or	Same place where I w		1 1 4		6	96		1	Used soap
e the house	Same place where I w		1 2 4	2 6		9		1	Used soap
e the house	Same place where I w	1 3		1	2	96		7	Used soap
e the house	Same place where I w		1 3 5	2 6		96		1	Used soap
d Bucket or	Same place where I w	1 3	1 4	4 7		96		1	Used soap
d Bucket or	Same place where I w	1 3	1 4	2 6		96	1 2		Used soap
d Bucket or	Same place where I w		1 1 4		6	9		1	Used soap
e the house	Other (Spe	live alone	1	1	2	96		1	Used soap
e compound	Same place where I w		3	1	2	9		3	Used soap
d Bucket or	Same place where I w		1	1	1	96		1	Used soap
d Bucket or	Same place where I w		2 2 4		2	4		3	Used soap
e the house	Same place where I w		1 2 4	2 6		9		2	Used soap
e compound	Same place where I w	1 3		1	2	9		2	Used soap
nen/sink insi	Same place where I w		2 2 5	3 7		1		2	Used soap
e the house	Same place where I w		1 1 5	2 6		9		7	Used soap
d Bucket or	Same place where I w		2 1 4	3 7		4		1	Used soap
e the house	Same place where I w		1 1 4	2 6		9		1	Used soap
e the house	Same place where I w		1 1 4	2 6		9		2	Used soap
e compound	Same place where I w	1 3		1	2	9		1	Used soap
d Bucket or	Same place where I w		1	1	2	96		1	Used soap
e the house	Same place where I w		1	1	2	96		1	Used soap
e the house	Same place where I w		1	1	2	96		2	Used soap
e the house	Same place where I w		1	1	2	96		7	Used soap
d bucket/ je	Same place where I w	2 4	2 5	1 7		8		2	Used soap
d Bucket or	Same place where I w		1 1 4		6	9		1	Used soap
e the house	Same place where I w		2	1	2	96		1	Used soap
tap/ water s	Same place where I w		2 1 5	2 6		96		1	Used soap

e the house	Same place where I w			1		1		2	96	1	Used soap
d Bucket or	Same place where I w		1 3			3		2	9	1 2 3	Used soap
e the house	Same place where I w			1	1 4		2 6		9	1	Used soap
d Bucket or	Same place where I w		1 3		3 5		2 6		9	1	Used soap
e the house	Same place where I w			1		1		2	96	7	Used soap
d Bucket or	Same place where I w			1	1 5		2 6		96	1	Used soap
e the house	Same place where I w			1		1		2	9	1	Used soap
e the house	Same place where I w		2 4		3 5		2 6		9 96	2	Used soap
tap/ water :	Same place where I w		2 4		1 5		2 6		96	1	Used soap
d Bucket or	Other (Spe	Living alon	1 96		1 5		2 6		96	1	Used soap
d Bucket or	Same place where I w			1		1		2	96	7	Used soap
e the house	Same place where I w			1		1		2	96	1	Used soap
d Bucket or	Same place where I w			1	1 4		2 6		9	1	Used soap
d Bucket or	Same place where I w			1	1 4		4 7		96	1	Used soap
ien/sink insi	Same place where I w			2	1 5		3 7		1	1	Used soap
e the house	Same place where I w			1	1 5		2 6		96	1	Used soap
e the house	Other (Spe	I live alone		1		3		2	9	1	Used soap
e the house	Same place where I w			1	1 4			6	9	1	Used soap
e the house	Same place where I w		1 2 3			1		4	9	3	Used soap
e the house	Same place where I w			1		1		2	96	1	Used soap
d Bucket or	Same place where I w		1 2			1		2	96	1	Used soap
d Bucket or	Same place where I w		1 3		1 5		2 6		9	1	Used soap
e the house	Same place where I w			1		1		2	9	3	Used soap
e the house	Same place where I w			1	1 4		2 6		9	7	Used soap
e the house	Same place where I w			1	1 4		2 6		9	1	Used soap
e the house	Same place where I w			1		1		2	96	1	Used soap
e compound	Same place where I w			1	3 4		4 6		9	2	Used soap
e the house	Same place where I w			1		1		2	9	2	Used soap
d Bucket or	Same place where I w		1 3		1 4			4	96	1	Used soap
e the house	Same place where I w		1 3		1 5		2 6		9	1	Used soap
e the house	Same place where I w		1 2			1		2	96	1	Used soap
d Bucket or	Same place where I w			2	1 4		4 7		96	2	Used soap
d Bucket or	Same place where I w			1		3		2	96	7	Used soap
e the house	Same place where I w			1		1		2	9	3	Used soap
d Bucket or	Same place where I w		1 2			1		2	96	1	Used soap
e the house	Same place where I w			1	1 4		2 6		9	1	Used soap
ien/sink insi	Same place where I w			2	2 4			2	96	7	Used soap
e the house	Same place where I w		1 2 3 4			1		2	96	1	Used soap
d Bucket or	Same place where I w		1 3			3		4	2	1	Used soap
d Bucket or	Same place where I w			1	3 5			2	9	1	Used soap
ien/sink insi	Same place where I w		1 2 3 4		2 5			2	1	1	Used soap
d Bucket or	Other (Spe	lives alone		1	3 5		2 8		9	1	Used soap
e the house	Same place where I w			1		1		2	9	2	Used soap
d Bucket or	Same place where I w			1	1 5		2 6		96	1	Used soap
e the house	Same place where I w			1	1 4		2 6		9	1	Used soap
e the house	Same place where I w			1		1		2	9	3	Used soap
e compound	Same place where I w		1 3			1		2	9	2	Used soap
e the house	Same place where I w			1		1		2	96	1	Used soap
e the house	Same place where I w			1		1		2	9	2	Used soap

d Bucket or	Same place where I w	1 3	1 5	2 6	9	1	Used soap
e the house	Same place where I w	1	3 5	4 7	9	1	Used soap
d Bucket or	Same place where I w	1	3 5	2	9	2	Used soap
d Bucket or	Same place where I w	1	1 4	2 6	9	7	Used soap
d Bucket or	Same place where I w	1 3	1	2	96	1	Used soap
d Bucket or	Same place where I w	1	1	2	96	1	Used soap
d Bucket or	Same place where I w	1	3 5	2	9	2	Used soap
d Bucket or	Same place where I w	1 3	1	2	96	1	Used soap
e the house	Same place where I w	1	1	2	96	7	Used soap
d Bucket or	Same place where I w	1	1	6	9	1	Used soap
d Bucket or	Same place where I w	1	1	2	96	1	Used soap
d Bucket or	Same place where I w	1 3	1 5	2 6	9	1	Used soap
e the house	Same place where I w	1 2 3	1	2	9	2	Used soap
d Bucket or	Same place where I w	4	3	2	6	7	Used soap
d Bucket or	Same place where I w	1	3 4	2	96	7	Used soap
e compoun	Same place where I w	1 3	1	2	9	2	Used soap
e the house	Same place where I w	1	1	1	96	1	Used soap
e the house	Same place where I w	1 2	1	2	96	1	Used soap
e compoun	Same place where I w	1	1 5	1 2	9	2	Used soap
en/sink insi	Same place where I w	2	2	2	96	2	Used soap
d Bucket or	Same place where I w	1	1 4	2 6	9	1	Used soap
d Bucket or	Same place where I w	96	1 4	2 7	96	1	Used soap
d Bucket or	Same place where I w	1	1	2	96	7	Used soap
d Bucket or	Same place where I w	1	1	2	96	7	Used soap
e the house	Same place where I w	2 4	3 5	2 6	9	1	Used soap
d bucket/ je	Same place where I w	96	1 5	2 6	96	1	Used soap
e compoun	Same place where I w	1	2	2	96	1	Used soap
e compoun	Same place where I w	1 3	1	1	9	2	Used soap
d Bucket or	Same place where I w	1	3 5	2	9	1	Used soap
en/sink insi	Same place where I w	2	2 5	2	1	1	Used soap
e the house	Same place where I w	1	2 5	2 6	2	1	Used soap
e the house	Same place where I w	1	1	2	9	2	Used soap
e the house	Same place where I w	1	1	2	96	1	Used soap
e the house	Same place where I w	3	1 4	2 6	9	7	Used soap
d Bucket or	Same place where I w	1	1 4	4 7	96	1	Used soap
e the house	Same place where I w	1	1	2	96	1	Used soap
d Bucket or	Same place where I w	1	1	2	96	1	Used soap
d Bucket or	Same place where I w	1 3	1	2	96	7	Used soap
e the house	Same place where I w	1	1 3 5	4	96	1	Used soap
d Bucket or	Same place where I w	1	1 5	2 6	96	1	Used soap
e the house	Same place where I w	1	1 4	2 7	9	1	Used soap
d Bucket or	Same place where I w	2	1 4	4 7	4	2	Used soap
e compoun	Same place where I w	1 3	1	2	9	1	Used soap
tap/ water	Same place where I w	1 2 3 4	1	2	9 96	1	Used soap
e the house	Same place where I w	1	1 4	2 6	9	96	Used soap
e the house	Same place where I w	1	1 4	2 6	9	1	Used soap
e the house	Same place where I w	1	5	2 6	9	1	Used soap
e the house	Same place where I w	1 3	1 5	2 6	9	1	Used soap
d Bucket or	Same place where I w	1 3	1	2	96	1	Used soap

e the house	Same place where I w			1	1	2	96	1	Used soap
e the house	Same place where I w			1	1	2	9	3	Used soap
e the house	Same place where I w			1	1	1	96	1	Used soap
e the house	Same place where I w			1	1	2	96	1	Used soap
e the house	Same place where I w			1 1 5		2	9	2	Used soap
e the house	Same place where I w			1	1	2	96	1	Used soap
d Bucket or	Other (Spe	She lives a		1	1	2	96	7	Used soap
e the house	Same place where I w			1 1 4	2 6		9	1	Used soap
d Bucket or	Same place where I w		1 2		1	2	96	1	Used soap
d Bucket or	Same place where I w		1 3	1 4	2 6		96	7	Used soap
d bucket/ je	Same place where I w		2 4		2	2	2	1	Used soap
e compound	Same place where I w			1	1	2	9	7	Used soap
e the house	Same place where I w			1	1	2	96	1	Used soap
e the house	Same place where I w			1	1	2	96	1	Used soap
d Bucket or	Same place where I w		1 3		1	2	96	1	Used soap
d Bucket or	Same place where I w			1	1	2	96	1	Used soap
e the house	Same place where I w			3	4	6	9	7	Used soap
e compound	Same place where I w			1	1	2	96	7	Used soap
e the house	Same place where I w			1	1	1	96	3	Used soap
d Bucket or	Same place where I w			1 1 5	2 6		96	1	Used soap
d Bucket or	Same place where I w			1 1 4	2 5 6		96	7	Used soap
d Bucket or	Same place where I w			1 3 5		2	9	1	Used soap
e the house	Same place where I w			1	1	2	9	2	Used soap
en/sink insi	Same place where I w		2 4	2 5		2	1	2	Used soap
e compound	Same place where I w		1 3		1	2	9	2	Used soap
e the house	Same place where I w			1 3 4	1 6		9	96	Used soap
d Bucket or	Same place where I w			1 3 5		2	9	1	Used soap
d Bucket or	Same place where I w		1 2		1	2	96	1	Used soap
e the house	Same place where I w		1 3	1 5	2 6		9	1	Used soap
e the house	Same place where I w			1	1	2	96	1	Used soap
d Bucket or	Same place where I w			1 1 4	2 6		9	1	Used soap
e the house	Same place where I w			1	1	2	96	1	Used soap
e the house	Same place where I w			1	1	2	96	1	Used soap
d bucket/ je	Same place where I w		2 4		3	2	7	7	Used soap
tap/ water	Same place where I w		1 2		1	1	96	2	Used soap
Sink outsid	Same place where I w			2	2	2	1	7	Used soap
e the house	Same place where I w			1	1	2	96	1	Used soap
d Bucket or	Same place where I w			1 3 5		2	9	2	Used soap
e the house	Same place where I w			1	1	2	9	3	Used soap
e the house	Same place where I w			2 3 5		2	96	1	Used soap
d Bucket or	Same place where I w		1 3	1 4	3 7		8	1	Used soap
e the house	Same place where I w			1	1	2	96	1	Used soap
d Bucket or	Same place where I w		1 3		1	2	96	1	Used soap
e the house	Same place where I w			1 1 4	2 6		9	96	Used soap
en/sink insi	Same place where I w			1	3	2	5	1	Used soap
d Bucket or	Same place where I w		1 2 4		3	2	2	2	Used soap
d bucket/ je	Same place where I w		2 4	3 5	2 6	2 9		1	Used soap
tap/ water	Same place where I w		2 4		3	6 1 96		1	Used soap
e the house	Same place where I w			1 1 4	2 6		9	1	Used soap

d Bucket or	Same place where I w		4 3 5		2	8	1	Used soap
d Bucket or	Same place where I w		1 3 4		2	9	7	Used soap
d Bucket or	Same place where I w	1 2		1	2	96	2	Used soap
e the house	Same place where I w	1 3		1	2	96	1	Used soap
d Bucket or	Same place where I w		2 1 4	3 7		2	1	Used soap
d Bucket or	Same place where I w	1 3		1	2	9	2	Used soap
d Bucket or	Same place where I w	2 96	3 5		2	9	2	Used soap
e the house	Same place where I w		1	1	2	2	2	Used soap
e the house	Same place where I w		1 1 4	2 6		9	1	Used soap
d bucket/ je	Same place where I w		1 2 5		2	2	1	Used soap
d Bucket or	Other (Spe	living alone	1	3	2	9	1	Used soap
d bucket/ je	Same place where I w		2 3 5	2 6		2	1	Used soap
d Bucket or	Same place where I w		1 1 4	2 6		9	1	Used soap
e the house	Same place where I w		1	1	2	9	3	Used soap
d Bucket or	Same place where I w		1	3	1	96	1	Used soap
e the house	Same place where I w	1 2 3 4		1	2	96	1	Used soap
d Bucket or	Same place where I w		1 1 5	2 6		96	1	Used soap
e the house	Same place where I w		4	2	7	2	2	Used soap
d Bucket or	Same place where I w		1	1	2	96	7	Used soap
d Bucket or	Same place where I w		96 1 5	2 6		96	1	Used soap
ıen/sink insi	Same place where I w	2 4	2 5		2	1	1	Used soap
e the house	Same place where I w	1 3	1 5	2 6		9	2	Used soap
e compoun	Same place where I w	1 2 3		1	2	9	7	Used soap
e the house	Same place where I w	1 2	3 5	2 6		96	1	Used soap
e the house	Same place where I w		1	1	2	96	1	Used soap
d Bucket or	Same place where I w		1	2	2	96	1	Used soap
d bucket/ je	Same place where I w	2 4	3 5	4 7	4 8		1	Used soap
e compoun	Same place where I w		1	1	2	9	2	Used soap
ıen/sink insi	Same place where I w		1	1	2	96	1	Used soap
e compoun	Same place where I w	1 3		1	2	9	3	Used soap
e the house	Same place where I w		2 1 4	2 6		9	1	Used soap
ıen/sink insi	Same place where I w	1 2 3 4	2 5		2	1	2	Used soap
e the house	Same place where I w		2 3 4	4 7		8	2	Used soap
ıen/sink insi	Same place where I w		2 2 5	2 7		1	1	Used soap
e the house	Same place where I w		1	1	2	96	3	Used soap
d bucket/ je	Same place where I w		2 2 4		2	4	7	Used soap
d Bucket or	Same place where I w	1 3	1 5	2 6		96	1	Used soap
e the house	Same place where I w		1 1 5	2 6		9	1	Used soap
e the house	Same place where I w		1 1 5	2 5		5	1	Used soap
e the house	Same place where I w		1 1 5	2 6		96	1	Used soap
d Bucket or	Same place where I w		4	4 4 6		2	7	Used soap
e the house	Same place where I w		1	1	2	96	1	Used soap
e the house	Same place where I w		1	1	2	9	3	Used soap
e the house	Same place where I w		1	1	2	96	1	Used soap
Designate	Same place where I w		2 2 5	3 7		2	2	Used soap
d Bucket or	Same place where I w	1 2		1	2	96	1	Used soap
ıen/sink insi	Same place where I w		2 2 5		2	1	2	Used soap
d Bucket or	Same place where I w	1 3		1	2	96	1	Used soap
d Bucket or	Same place where I w		1 1 4	2 6		9	1	Used soap

d Bucket or	Same place where I w	1 3	1 5	2 6		9		1	Used soap
e compound	Same place where I w	1 3		1	2	9		7	Used soap
d Bucket or	Same place where I w		2 2 3 5		2	2		1	Used soap
e compound	Same place where I w	1 3		1	2	9		2	Used soap
e compound	Same place where I w	1 4		1	2	9		2	Used soap
e the house	Same place where I w	1 2	1 4	2 6		9		1	Used soap
d Bucket or	Same place where I w	1 2		1	1	96		1	Used soap
e compound	Same place where I w		1	3	2	9		2	Used soap
ien/sink insi	Same place where I w	1 2 3	2 5	2 6		5		7	Used soap
d Bucket or	Same place where I w		1 1 4	4 6		96		7	Used soap
d Bucket or	Same place where I w	1 3	3 5	2 6		9		1	Used soap
e the house	Same place where I w		1	1	2	96		1	Used soap
ien/sink insi	Same place where I w		2	2 2 6		1		2	Used soap
e the house	Same place where I w		1 1 5	2 6		9		1	Used soap
d Bucket or	Same place where I w		1	1	2	96		7	Used soap
e the house	They do not wash thei	1 3		1	2	96		1	Used soap
e the house	Same place where I w		1	1	2	96		3	Used soap
d Bucket or	Same place where I w	1 2		1	2	96		1	Used soap
e the house	Same place where I w	1 3		1	2	9		1	Used soap
e the house	Same place where I w		1 1 4	2 6		9		1	Used soap
d bucket/ je	Same place where I w		1 1 4		3	2		7	Used soap
d Bucket or	Same place where I w	2 4		3	2	2		7	Used soap
d Bucket or	Same place where I w	2 4		3	2	96		7	Used soap
e the house	Same place where I w		1	1	2	9		3	Used soap
e the house	Same place where I w	1 3	1 5	2 6		9		1	Used soap
d bucket/ je	Same place where I w	1 2 3 4		1	3	96	1 2 3		Used soap
e the house	Same place where I w		1 1 4	2 6		9		1	Used soap
d Bucket or	Same place where I w	1 3		1	2	96		7	Used soap
d Bucket or	Same place where I w		1	1	2	96		1	Used soap
e the house	Same place where I w		1 1 4	2 6		9		1	Used soap
d Bucket or	Same place where I w		1	1	2	96		1	Used soap
e the house	Same place where I w		1 1 4	2 6		9		7	Used soap
e compound	Same place where I w	1 3	3 5		2	9		3	Used soap
d Bucket or	Same place where I w	1 3		1	2	96		1	Used soap
e compound	Same place where I w		1	1	7	9		1	Used soap
e the house	Same place where I w		1	1	2	96		1	Used soap
d bucket/ je	Same place where I w		2	2 2 6		3		2	Used soap
d Bucket or	Same place where I w		1 1 5	2 6		96		1	Used soap
e the house	Same place where I w		1	1	2	9		7	Used soap
d Bucket or	Same place where I w		1 1 5	2 6		96	1 3		Used soap
e the house	Same place where I w		1	1	2	96		1	Used soap
e compound	Same place where I w	1 3		3 4 7		9	2 3		Used soap
e the house	Same place where I w		2	2	3	8		1	Used soap
e the house	Same place where I w		1	1	2	9		7	Used soap
e the house	Same place where I w		1 1 4	2 6		9		1	Used soap
e compound	Same place where I w	1 3		1	2	9		7	Used soap
tap/ water :	Same place where I w		2	1	3	96		2	Used soap
d Bucket or	Same place where I w		1	1	2	96		7	Used soap
d Bucket or	Same place where I w	2 4	3 5	2 6		9		1	Used soap

d Bucket or	Same place where I w		4		3		2	4	7	Used soap
e the house	Same place where I w	1 4			1		7	9	1	Used soap
e compoun	Same place where I w	1 3 4			1		2	9	2	Used soap
d Bucket or	Same place where I w		1		1		2	96	7	Used soap
e the house	Same place where I w		1	2 4		2 6		9	1	Used soap
e compoun	Same place where I w		1	3 4			2	96	1	Used soap
e the house	Same place where I w		1		1		2	9	3	Used soap
e the house	Same place where I w		1	3 5			2	9	1	Used soap
tap/ water	Same place where I w	2 4			1		2	96	1	Used soap
d Bucket or	Same place where I w		1	1 4		2 6		9	1	Used soap
e the house	Same place where I w	1 3			1		2	9	7	Used soap
d Bucket or	Same place where I w		1		1		2	96	1	Used soap
e the house	Same place where I w		1		1		2	96	1	Used soap
tap/ water	Same place where I w		2	1 5		2 7		96	1	Used soap
e the house	Same place where I w		1		1		2	96	1	Used soap
e the house	Same place where I w		1		1		1	96	1	Used soap
tap/ water	Same place where I w		1		1		3	9	1	Used soap
d Bucket or	Same place where I w		1		1		2	96	1	Used soap
e the house	Same place where I w	1 3			1		2	9	2	Used soap
d Bucket or	Same place where I w		1		1		2	96	7	Used soap
e the house	Same place where I w		1	1 4		1 6		9	1	Used soap
e the house	Same place where I w		1		1		2	9	2	Used soap
d Bucket or	Same place where I w	1 3			1		2	96	1	Used soap
ien/sink insi	Same place where I w	1 2 4			1		2	96	1	Used soap
e the house	Same place where I w		1		1		2	9	1	Used soap
d Bucket or	Same place where I w	1 2			1		2	96	1	Used soap
e the house	Same place where I w		1		1		2	96	1	Used soap
e the house	Same place where I w	1 2		1 5		2 6		96	1	Used soap
e the house	Same place where I w	1 2		1 4		2 6		9	1	Used soap
tap/ water	Same place where I w	2 4		1 4			3	96	1	Used soap
ien/sink insi	Same place where I w	1 2 3 4		2 5		3 6		1	2	Used soap
d Bucket or	Same place where I w	1 2			1		2	96	1	Used soap
e the house	Same place where I w		1	1 4		2 6		9	1	Used soap
d Bucket or	Same place where I w	1 2			1	3 6		5	1	Used soap
d Bucket or	Same place where I w	1 2			1		2	96	1	Used soap
e the house	Same place where I w		1	1 5		2 6		9	96	Used soap
d Bucket or	Same place where I w		2	1 5		3 7		5	1	Used soap
e the house	Same place where I w	1 2			1		2	9	1	Used soap
e the house	Same place where I w		1	1 5		2 6		9	1	Used soap
d Bucket or	Same place where I w		96	1 5		2 6		96	1	Used soap
e the house	Same place where I w	2 4		3 5		2 6		9	2	Used soap
d Bucket or	Same place where I w		2	3 5			2	2	2	Used soap
tap/ water	Same place where I w		2	1 4		3 7		96	1	Used soap
e the house	Same place where I w		1		1		2	96	1	Used soap
e the house	Same place where I w		1	1 3			2	96	1	Used soap
d Bucket or	Same place where I w	2 3		1 4		4 7		96	1	Used soap
d Bucket or	Same place where I w		1		1		2	96	1	Used soap
d Bucket or	Same place where I w	2 4		3 5		2 6		9	1	Used soap
d bucket/ je	Same place where I w		2	3 5			1	4	1	Used soap

d Bucket or	Same place where I w	1 3			1		2	96		2	Used soap
e the house	Same place where I w		1		1		2	96		1	Used soap
e the house	Same place where I w		1		2		2	96		1	Used soap
e the house	Same place where I w		1 1 3				2	96		1	Used soap
d Bucket or	Same place where I w	1 2			3		3	2	1 2		Used soap
e the house	Same place where I w		1		1		2	96		1	Used soap
d Bucket or	Same place where I w		2		3		2	3		1	Used soap
d Bucket or	Same place where I w		3		1		2	96		1	Used soap
e the house	Same place where I w		1 1 4			2 6		9		1	Used soap
e the house	Same place where I w		1		1 4 7			9		1	Used soap
e the house	Same place where I w		1		1		2	9		2	Used soap
d bucket/ je	Same place where I w	1 2			1 4 6			96		7	Used soap
e the house	Same place where I w	1 3		1 5		2 6		9		1	Used soap
d Bucket or	Same place where I w		2 3 5			2 6		96		1	Used soap
e the house	Same place where I w		1		1		1	96		1	Used soap
d Bucket or	Same place where I w	1 3			1		2	96		1	Used soap
e the house	Same place where I w		1		1		2	96		1	Used soap
e the house	Same place where I w		1		3 2 6			9		1	Used soap
e the house	Same place where I w	1 3		3 5		2 6		9		1	Used soap
e the house	Same place where I w	1 3		3 4		2 6		96		7	Used soap
d Bucket or	Same place where I w	1 2			1		2	96		1	Used soap
ıen/sink insi	Same place where I w	2 4			2		2	1		7	Used soap
d Bucket or	Same place where I w		1 3 4				2	9		7	Used soap
e the house	Same place where I w		1		1		6	9		7	Used soap
e the house	Same place where I w	1 3		1 5		2 6		96		1	Used soap
d Bucket or	Same place where I w		2 1 4			4 7		4		2	Used soap
e the house	Same place where I w	1 2		1 5		2 6		9		1	Used soap
e the house	Same place where I w		1		1		2	96		1	Used soap
d Bucket or	Same place where I w		1		1		1	96		1	Used soap
e the house	Same place where I w		1		1		2	96		1	Used soap
e the house	Same place where I w		1		1		2	9		3	Used soap
d Bucket or	Same place where I w		1		1		2	96		7	Used soap
d bucket/ je	Same place where I w		96		1		2	96		7	Used soap
e the house	Same place where I w		1 1 4				6	8		1	Used soap
e the house	Same place where I w	1 3			1		2	96		7	Used soap
e the house	Same place where I w		1 3 5				2	96		1	Used soap
d Bucket or	Same place where I w		1		1		2	96		7	Used soap
e the house	Same place where I w	2 4		3 5		2 6		9		1	Used soap
d Bucket or	Same place where I w		1		1		2	96		1	Used soap
e the house	Same place where I w		1		1		2	9		3	Used soap
d bucket/ je	Same place where I w		2		2		2	2		2	Used soap
e compoun	Same place where I w	1 3			1		2	9		3	Used soap
e the house	Same place where I w		1		1		2	9		2	Used soap
d Bucket or	Same place where I w	1 2		1 4		4 7		96		7	Used soap
d bucket/ je	Same place where I w	1 2 4		2 4			2	8		1	Used soap
d Bucket or	Same place where I w		1		1		2	96		1	Used soap
tap/ water :	Same place where I w	2 4			1		3	96		2	Used soap
d Bucket or	Same place where I w	1 2 3 4		1 4		2 7		96		7	Used soap
d Bucket or	Same place where I w		2 3 5			2 6		4		1	Used soap

Sato handv	Same place where I w		2 2 5	2 6		96	1	Used soap
d Bucket or	Same place where I w	1 3	3 5	2 6		9	1	Used soap
d Bucket or	Same place where I w		1 1 5	2 6		96	1	Used soap
d Bucket or	Same place where I w	1 3		1	1	96	1	Used soap
d Bucket or	Same place where I w	1 3		1	2	96	7	Used soap
e the house	Same place where I w		1	1	2	96	1	Used soap
d Bucket or	Same place where I w		1 1 5	2 6		96	1	Used soap
d bucket/ je	Same place where I w		2 3 5	2 6		3 1 3		Used soap
e the house	Same place where I w	1 3		1	2	96	1	Used soap
d Bucket or	Same place where I w	1 2		1	2	96	1	Used soap
d Bucket or	Same place where I w		1	1	2	96	1	Used soap
e compound	Same place where I w	1 3		1	6	9	2	Used soap
e compound	Same place where I w	1 3		1	2	9	7	Used soap
d Bucket or	Same place where I w		1	1	2	96	3	Used soap
e the house	Same place where I w		1 1 4	2 6		9	1	Used soap
d Bucket or	Same place where I w		1	1	2	96	1	Used soap
d bucket/ je	Same place where I w	2 4	2 5		2	2	2	Used soap
e the house	Same place where I w	1 4		1	2	96	1	Used soap
e the house	Same place where I w		1	1	2	9	2	Used soap
e the house	Same place where I w		1	1	2	9	1	Used soap
d Bucket or	Same place where I w		1 1 4	2 6		9	1	Used soap
ien/sink insi	Same place where I w		1	1	2	1	2	Used soap
ien/sink insi	Same place where I w		2 2 5		2	1	1	Used soap
ien/sink insi	Same place where I w	2 4	2 5		3 9 96		1	Used soap
d Bucket or	Same place where I w		2	1	2	96	7	Used soap
e compound	Same place where I w	1 2 3 4		1	2	9	1	Used soap
ien/sink insi	Same place where I w		2 2 5		2	1 2 3		Used soap
e the house	Same place where I w	1 3	1 5	2 6		9	1	Used soap
e the house	Same place where I w	2 4	1 5	2 6		9	1	Used soap
e the house	Same place where I w		1	1	2	96	2	Used soap
tap/ water :	Same place where I w	2 4		1 3 6		96	1	Used soap
e the house	Same place where I w		1 3 5		2	9	1	Used soap
tap/ water :	Same place where I w		1	1	2	9	3	Used soap
d Bucket or	Same place where I w	1 3		1	2	96	1	Used soap
e the house	Other (Spe	I dont know	1	1	2	9	2	Used soap
d Bucket or	Same place where I w		1 1 4		6	9	7	Used soap
e the house	Same place where I w		1 1 5	2 6		9	1	Used soap
e the house	Same place where I w		1	1	2	9	2	Used soap
e the house	Same place where I w		1 1 4		2	9	3	Used soap
d Bucket or	Same place where I w		2	1	2	96	1	Used soap
d Bucket or	Same place where I w	1 3		3	4	8	2	Used soap
d Bucket or	They do not wash thei	1 2 3 4	1 5	2 6		9	2	Used soap
tap/ water :	Same place where I w		2	1 3 6		96	7	Used soap
d Bucket or	Same place where I w		1	1	2	96	7	Used soap
d Bucket or	Same place where I w		1 1 5	2 6		96	1	Used soap
ien/sink insi	Same place where I w	2 4		2	2	1	96	Used soap
d Bucket or	Same place where I w		1 3 5		2	9	1	Used soap
e compound	Same place where I w	1 3		1 4 7		9	1	Used soap
e the house	Same place where I w		1 3 5	2 6		96	1	Used soap

e the house	Same place where I w		1	4	6	9		2	Used soap
d Bucket or	Same place where I w	1 3	1 5	2 6		9		1	Used soap
d Bucket or	Same place where I w		1	1	2	96		7	Used soap
ıen/sink insi	Same place where I w		1 1 4	2 6		9		1	Used soap
d Bucket or	Same place where I w		1	2	2	8		1	Used soap
e the house	Same place where I w		1	1	2	9		1	Used soap
d Bucket or	Same place where I w		2	3	4	8		1	Used soap
e the house	Same place where I w		1	1	2	9		2	Used soap
e the house	Same place where I w	1 2		1	6	9	1 2		Used soap
e the house	Same place where I w		1 1 4	2 6		9		1	Used soap
e the house	Same place where I w		1	1 2 6		9		2	Used soap
ıen/sink insi	Same place where I w		96 1 4	2 6		9		1	Used soap
e compound	Same place where I w	1 3		1	2	96		2	Used soap
e the house	Same place where I w		1 1 5	2 6		9		2	Used soap
d Bucket or	Same place where I w		2 3 5	2 6		2		1	Used soap
d Bucket or	Same place where I w		1	1	2	96		7	Used soap
d Bucket or	Same place where I w	1 3	1 5	2 6		96		1	Used soap
d Bucket or	Same place where I w	2 4	3 5		2	4		7	Used soap
e the house	Same place where I w	1 3	3 5	2 6		9		1	Used soap
e the house	Same place where I w	1 3		1	2	9		7	Used soap
e compound	Same place where I w		1 1 4	2 7		96		1	Used soap
e the house	Same place where I w		1 1 4	2 6		9		1	Used soap
d Bucket or	Same place where I w		2	3	2	7		7	Used soap
e the house	Same place where I w		3	1	2	96		96	Used soap
e the house	Same place where I w		1 1 5	2 6		96		1	Used soap
d Bucket or	Same place where I w	1 3		1	2	96		7	Used soap
e the house	Same place where I w	2 4		1	2	96		1	Used soap
e the house	Same place where I w		1	1	2	96		1	Used soap
e the house	Same place where I w		1 1 5	2 6		96		1	Used soap
e the house	Same place where I w		1 1 4	2 6		9		1	Used soap
d Bucket or	Same place where I w		1	1 4 7		96		1	Used soap
e the house	Same place where I w	1 3 4		1	2	96		1	Used soap
d Bucket or	Same place where I w		1 3 5		2	9		1	Used soap
d Bucket or	Same place where I w		2 2 5	3 7		5		1	Used soap
e the house	Same place where I w		1	1	2	96		1	Used soap
d Bucket or	Same place where I w		96 1 5	2 6		96		1	Used soap
d Bucket or	Same place where I w	1 2		1	2	96		1	Used soap
d bucket/ je	Same place where I w		2	3	4	2		2	Used soap
e the house	Same place where I w		1	1	2	96		1	Used soap
e the house	Same place where I w	1 2 3		1	2	9		1	Used soap
e compound	Same place where I w		1	1	2	96		1	Used soap
d Bucket or	Same place where I w		1	1	2	96		1	Used soap
e the house	Same place where I w		1	1	6	9		1	Used soap
d Bucket or	Same place where I w	1 3		1	2	96		1	Used soap
e the house	Same place where I w		1	1	2	96		1	Used soap
d Bucket or	Same place where I w		1	1	2	96		7	Used soap
e the house	Same place where I w		1	1	2	96		1	Used soap
He doesnt	Other (Spe	Na	2	1	2	96		7	Used soap
e the house	Same place where I w		1	1	2	9		2	Used soap

e the house	Same place where I w			1	1 4		2 6		9		1	Used soap
d Bucket or	Same place where I w			1	1 5		2 6		96		1	Used soap
e the house	Same place where I w			1	2 4		2 6		9		1	Used soap
e the house	Same place where I w			2		1		2	9		3	Used soap
d Bucket or	Same place where I w			1		1		1	96		1	Used soap
e the house	Same place where I w		1 3			1		1	96		1	Used soap
e the house	Same place where I w			1		1		6	9		1	Used soap
d Bucket or	Same place where I w			1	1 5		2 6		96		1	Used soap
e compound	Same place where I w		2 4			1		2	9		3	Used soap
d Bucket or	Same place where I w			1	1 4		2 5 6		96		7	Used soap
e compound	Same place where I w		1 3			1		2	9		3	Used soap
d Bucket or	Same place where I w			96	1 5		2 6		96		1	Used soap
e the house	Same place where I w			1		1		1	96		1	Used soap
e the house	Same place where I w			1	1 5			2	9	1 2		Used soap
e the house	Same place where I w		1 2 3 4			1		6	9		1	Used soap
d Bucket or	Same place where I w			1	1 4		2 6		9		1	Used soap
e the house	Same place where I w			1	1 4		2 6		9		1	Used soap
e the house	Same place where I w			1		1		2	96		1	Used soap
d Bucket or	Same place where I w		1 3			1		2	96		7	Used soap
e the house	Same place where I w		1 3 4			1		2	9		1	Used soap
e the house	Same place where I w		1 2			1		1	96		1	Used soap
d Bucket or	Same place where I w			2	1 4		2 6		9		7	Used soap
d Bucket or	Same place where I w		1 2		1 4		4 7		96		1	Used soap
e the house	Same place where I w		1 2		1 4		2 6		9		1	Used soap
e the house	Same place where I w			1	3 4			2	96		7	Used soap
e the house	Same place where I w		1 2		1 4		2 6		9		1	Used soap
d Bucket or	Same place where I w			2	3 5			2	8		1	Used soap
e the house	Same place where I w			1		1		2	96		1	Used soap
e compound	Same place where I w		1 3			1		2	9		1	Used soap
e the house	Same place where I w			1		1		2	96		1	Used soap
e the house	Same place where I w			1		1		2	9		1	Used soap
e the house	Same place where I w			1	3 4			2	96		1	Used soap
d Bucket or	Same place where I w			1	1 5		2 6		96		1	Used soap
e the house	Same place where I w			1		4	2 7		9		1	Used soap
e the house	Same place where I w		1 3			1		1	96		1	Used soap
d Bucket or	Same place where I w			1		1		2	96		7	Used soap
d Bucket or	Same place where I w			1	1 3			4	96		1	Used soap
e the house	Same place where I w			1	1 4		2 6		9		1	Used soap
e the house	Same place where I w			1		1		2	96		1	Used soap
d Bucket or	Same place where I w			1	1 4		4 7		96		1	Used soap
d Bucket or	Same place where I w			1		1		2	96		1	Used soap
d bucket/ je	Same place where I w			2		3		2	8		7	Used soap
e the house	Same place where I w			1		1		1	96		7	Used soap
e the house	Same place where I w			1	1 4		2 6		9		1	Used soap
d bucket/ je	Same place where I w			1		3		2	8		1	Used soap
d Bucket or	Same place where I w			1	1 5		2 6		96		1	Used soap
e the house	Other (Spe	NA		1	1 4		2 6		9		1	Used soap
ien/sink insi	Same place where I w			2	2 4			2	1		7	Used soap
d Bucket or	Same place where I w		1 2			1		2	96		7	Used soap

ıen/sink insi	Same place where I w		2		1		2		96		1	Used soap
e compoun	Same place where I w	1 3			3		4		9		2	Used soap
d Bucket or	Same place where I w	1 3			1		2		96		1	Used soap
ıen/sink insi	Same place where I w		2		2		3		1	1 2		Used soap
tap/ water	Same place where I w	2 4		3 4			3		96		7	Used soap
e compoun	Same place where I w	1 3			1		2		9		1	Used soap
e the house	Same place where I w		1 1 4			2 6			9		1	Used soap
e the house	Same place where I w		1 1 5			2 6			96	1 3		Used soap
ıen/sink insi	Same place where I w		2 2 5			4 6			2		2	Used soap
d Bucket or	Same place where I w		1 3 5				2		9		1	Used soap
d Bucket or	Same place where I w	1 3			4		6		96	1 3		Used soap
d bucket/ je	Same place where I w	2 4			2		2		4		7	Used soap
e the house	Same place where I w		1 1 4			2 6			9		1	Used soap
d Bucket or	Same place where I w	1 3		1 5		2 6			96		1	Used soap
e the house	Same place where I w	1 2 3		1 5		2 6			9		1	Used soap
e the house	Same place where I w		1		1		2		96		1	Used soap
e the house	Same place where I w		3		1		2		9		2	Used soap
d Bucket or	Same place where I w		1		1		2		96		1	Used soap
d Bucket or	Same place where I w		2		1		2		8		2	Used soap
e compoun	Same place where I w		1		1		2		9		4	Used soap
e the house	Same place where I w		1		1		2		9		1	Used soap
e the house	Same place where I w		1 3 5				2		9		1	Used soap
e the house	Same place where I w		1		1		2		9		2	Used soap
d bucket/ je	Same place where I w		2 3 5			2 6			3		1	Used soap
e the house	Same place where I w		1 1 4			2 6			9		1	Used soap
d Bucket or	Same place where I w		1 1 4			4 6			96		7	Used soap
e compoun	Same place where I w	1 3			1		2 9 96				3	Used soap
e the house	Same place where I w		1		1		2		2		1	Used soap
e the house	Same place where I w		1		1		2		9		3	Used soap
e the house	Same place where I w		1		1		2		9		1	Used soap
e the house	Same place where I w		1		1		2		9		7	Used soap
e the house	Same place where I w		1		1		2		96		1	Used soap
e the house	Same place where I w	1 4			1		4		96		2	Used soap
d Bucket or	Same place where I w		1		1		2		96		1	Used soap
e the house	Same place where I w		1 2 4			2 6			9		1	Used soap
e compoun	Same place where I w		1		1		7		9		3	Used soap
e compoun	Same place where I w	1 3			1		2		9		3	Used soap
d Bucket or	Same place where I w		1 1 5			4 7			96		1	Used soap
ıen/sink insi	Same place where I w	1 3			1		2		1		7	Used soap
d Bucket or	Same place where I w	1 3		1 5		2 6			9		1	Used soap
d Bucket or	Same place where I w	1 2			1		2		96		1	Used soap
e the house	Same place where I w	1 3			1		2		96		1	Used soap
d Bucket or	Same place where I w		1 1 5			2 6			96		1	Used soap
ıen/sink insi	Same place where I w	2 4			1		6		9		1	Used soap
d Bucket or	Same place where I w		1		1		2		96		7	Used soap
ıen/sink insi	Same place where I w	1 3		3 5		2 6			96		1	Used soap
e the house	Same place where I w		1		1		2		9		2	Used soap
e the house	Same place where I w	2 4		1 5		2 6		9 96			1	Used soap
e the house	Same place where I w	1 2			1		2		96		1	Used soap

e the house	Same place where I w			1	1	2	96	1	Used soap
e the house	Same place where I w		1 2 3 4	3 5	2 6		96	1	Used soap
d Bucket or	Same place where I w		1 3		1	1	96	1	Used soap
d bucket/ je	Same place where I w			2 2 4		2	2	1	Used soap
d Bucket or	Same place where I w			1 1 4		2	96	7	Used soap
e the house	Same place where I w		1 3		1	2	96	1	Used soap
d Bucket or	Same place where I w			2 1 4	4 7		96	2	Used soap
e the house	Same place where I w			1	1	2	9	7	Used soap
d Bucket or	Same place where I w		1 3	3 5	2 6		9	1	Used soap
e the house	Same place where I w			1 1 4	2 6		96	7	Used soap
e the house	Other (Spe	Sink	1 2 3 4	2 5		2 1 9		3	Used soap
e the house	Same place where I w			1 1 4		2	9	1	Used soap
e the house	Same place where I w			1	1	2	9	2	Used soap
e compoun	Same place where I w			1	1	2	9	2	Used soap
e compoun	Same place where I w		1 3		1	2	9	1	Used soap
d Bucket or	Same place where I w			2	3	4	8	1	Used soap
e the house	Other (Spe	stays alone		1 3 5		2	9	1	Used soap
tap/ water :	Same place where I w			2 1 5	2 6		96	1	Used soap
e the house	Same place where I w		1 4		1	1	96	1	Used soap
e compoun	Same place where I w		1 3		1	2	9	1	Used soap
d Bucket or	Same place where I w		1 2		1	2	96	1	Used soap
en/sink insi	Same place where I w			1 1 4		6	96	7	Used ash/s
e the house	Same place where I w		2 4	1 5	2 6	9 96		1	Used ash/s
d Bucket or	Same place where I w			2	1	4	8	2	Used ash/s
e the house	Same place where I w			1 1 4	2 6		96	7	Used ash/s

q42a	q43	q44a	q44b	q45a	q45b	q46	q47	q48a
1 5 6 12	82	Using own clothes		Poured out into the op		They appe	Yes, some	Detergent/p
1 10 96	5	Did not dry		Left in the bucket		Visibly clea	Yes, some	Bar soap, c
1 4 10 96	12	Did not dry		Poured out in a draina		Visibly clea	Yes, some	Detergent/p
1 12	50	Air dried		Poured out into the op		They appe	No	
96	0	Other (Spe	Didnt wash	Other (Spe	N/a	Visibly clea	Yes, some	Liquid soap
1 10 96	15	Did not dry		Left in the bucket		They appe	No	
1 11	60	Air dried		Poured out into the op		Visibly clea	Yes, some	Detergent/p
96	0	Other (Spe	N	Other (Spe	Na	Visibly clea	Yes, some	Detergent/p
1 11 12	50	Air dried		Poured out into the op		They appe	Yes, some	Detergent/p
1	5	Did not dry		Drains into the open		They appe	Yes, some	Bar soap, s
96	0	Other (Spe	Did not was	Other (Spe	NA	They appe	Yes, some	Bathing soa
1 11	1	Did not dry		Left in the bucket		They appe	Yes, some	Bar soap, s
1 4 5 7 8 9	150	Using own clothes		Left in the bucket		They appe	Yes, some	Liquid soap
1 10	3	Did not dry		Left in the bucket		They appe	Yes, some	Bar soap fo
1 10 12	2	Using own clothes		Poured out into the op		Visibly clea	Yes, some	Bathing soa
1 2 4 6 8 9	20	Using other piece of cl		Left in the bucket		Visibly clea	Yes, alway	Bar soap, c
1 4 6 9 12	1	Air dried		Poured out into the op		They appe	No	
1 2 3	5	Air dried		Poured out into the op		Visibly clea	Yes, some	Detergent/p
1 4 5	10	Did not dry		Left in the bucket		Visibly clea	Yes, some	Bar soap, c
1 4 5 12	80	Air dried		Left in the bucket		They appe	Yes, some	Detergent/p
1 4 5 10	5	Did not dry		Left in the bucket		Visibly clea	Yes, some	Bar soap, c
1 11	6	Did not dry		Left in the bucket		Visibly clea	Yes, some	Bathing soa
1 4 10 12	5	Air dried		Drains into the open		Visibly clea	Yes, some	Bar soap fo
1 3	5	Air dried		Poured out into the op		Visibly clea	Yes, some	Bar soap, s
1 3 5 8 10	3	Did not dry		Poured out into the op		Visibly clea	Yes, some	Bar soap, s
1 3 4 10 12	4	Using own clothes		Left in the bucket		Visibly clea	Yes, some	Bar soap, s
1 4 5 6 12	128	Using own clothes		Poured out into the op		Visibly clea	Yes, some	Bathing soa
1 4 11 96	8	Other (Spe	did not den	Other (Spe	did not der	Visibly clea	Yes, some	Liquid soap
1 4 5 6 12	5	Using own clothes		Left in the bucket		Visibly clea	Yes, some	Bar soap, s
1 3 4	5	Air dried		Poured out into the op		Visibly clea	Yes, some	Liquid soap
1 5 6	10	Air dried		Left in the bucket		They appe	Yes, some	Bar soap, s
1 3 4	5	Air dried		Poured out into the op		Visibly clea	Yes, some	Liquid soap
1 3 4 5 6 7	8	Did not dry		Left in the bucket		Visibly clea	Yes, some	Bar soap fo
1 3	5	Air dried		Poured out into the op		Visibly clea	Yes, some	Bar soap, s
1 3 4	5	Air dried		Poured out into the op		Visibly clea	Yes, some	Liquid soap
1 3 4	5	Air dried		Poured out into the op		Visibly clea	Yes, some	Liquid soap
1 3 4 5 6 7	20	Did not dry		Left in the bucket		Visibly clea	Yes, some	Bathing soa
1 3 4	5	Air dried		Poured out into the op		Visibly clea	Yes, some	Detergent/p
1 2 3 4 6 8	20	Air dried		Poured out into the op		Visibly clea	Yes, some	Bar soap, s
1 4 11 12	178	Using own clothes		Poured out into the op		Visibly clea	Yes, some	Bar soap, c
1 5 6 10	15	Air dried		Drains into the open		They appe	Yes, some	Bar soap, s
1 2 3 4 5 9	65	Air dried		Poured out into the op		They appe	Yes, alway	Bar soap, s
1 2 3 4	7	Air dried		Pours into the sink		Visibly clea	Yes, some	Liquid soap
1 2 3	5	Air dried		Poured out into the op		Visibly clea	Yes, some	Bathing soa
1 3 4 12	8	Using own clothes		Left in the bucket		Visibly clea	Yes, some	Bar soap, s
1 8	5	Did not dry		Drains into the open		Visibly clea	Yes, some	Bathing soa
1 4 5 6 8 1	2	Did not dry		Drains into the open		Visibly clea	Yes, some	Liquid soap
1 3 4	5	Using other piece of cl		Poured out into the op		Visibly clea	Yes, some	Liquid soap

1 5 6 9	250	Air dried	Poured out into the op	Visibly clea	Yes, some	Liquid soap
1 4 5 6	10	Air dried	Poured out into the op	They appe	No	
1 3 4	5	Air dried	Poured out into the op	Visibly clea	Yes, some	Liquid soap
1 4 5 11 12	350	Air dried	Left in the bucket	Visibly clea	Yes, some	Liquid soap
1 3 4	5	Air dried	Poured out into the op	Visibly clea	Yes, some	Liquid soap
1 4 8 11	10	Did not dry	Poured out in a draina	Visibly clea	Yes, some	Bathing soa
1 3 4 5 96	15	Did not dry	Drains into the open	Visibly clea	Yes, some	Liquid soap
1 4 5 6 11	200	Using own clothes	Poured out into the op	They appe	Yes, some	Detergent/p
1 3 4 5 11	32	Did not dry	Left in the bucket	Visibly clea	Yes, some	Liquid soap
1 2 3 4 6 7	15	Using own clothes	Left in the bucket	Visibly clea	Yes, some	Bathing soa
1 5 7	120	Using other piece of cl	Poured out into the op	Visibly clea	Yes, some	Liquid soap
1 4 5 11	25	Did not dry	Left in the bucket	Visibly clea	Yes, some	Bar soap, c
1 4 10 12	5	Using own clothes	Drains into the open	Visibly clea	Yes, some	Bar soap fo
1 3 4 11	200	Using own clothes	Left in the bucket	Visibly clea	Yes, some	Liquid soap
1 3 4	5	Air dried	Poured out into the op	Visibly clea	Yes, some	Liquid soap
1 4 10	4	Using own clothes	Pours into the sink	They appe	Yes, some	Liquid soap
1 4 12	5	Using own clothes	Left in the bucket	Visibly clea	Yes, some	Bar soap, s
1 4 9 11 12	20	Using own clothes	Left in the bucket	Visibly clea	Yes, alway	Liquid soap
1 3 9	5	Did not dry	Left in the bucket	Visibly clea	Yes, some	Bathing soa
1 3	5	Did not dry	Drains into the open	They appe	Yes, some	Bathing soa
1 2 3 4 5 6	20	Air dried	Poured out into the op	Visibly clea	Yes, some	Bathing soa
1 4 10 12	3	Using own clothes	Left in the bucket	They appe	Yes, some	Bar soap, c
1 4 10 96	15	Did not dry	Left in the bucket	Visibly clea	Yes, some	Liquid soap
1 4 5 11	5	Did not dry	Left in the bucket	Visibly clea	Yes, some	Bar soap fo
1 4 5 12	1	Air dried	Drains into the open	They appe	Yes, some	Bathing soa
1 3 4	5	Air dried	Poured out into the op	Visibly clea	Yes, some	Liquid soap
1 5 6 11	102	Air dried	Left in the bucket	They appe	Yes, some	Liquid soap
1 4 10	2	Did not dry	Poured out into the op	Visibly clea	Yes, some	Bar soap, s
1 3 4	5	Air dried	Poured out into the op	Visibly clea	Yes, some	Liquid soap
1 3 4 5 12	246	Air dried	Poured out into the op	Visibly clea	Yes, some	Bar soap, s
1 5 6 7 8 9	143	Did not dry	Poured out into the op	They appe	Yes, some	Other (spe
1 4 5 8 12	120	Using other piece of cl	Poured out into the op	They appe	Yes, some	Liquid soap
1 3	5	Air dried	Poured out into the op	Visibly clea	Yes, some	Liquid soap
1 2 3 4 5 6	18	Air dried	Poured out into the op	Visibly clea	Yes, some	Liquid soap
1 5 6 7	140	Using own clothes	Poured out into the op	They appe	Yes, some	Detergent/p
1 4 5 10	5	Did not dry	Poured out into the op	Visibly clea	Yes, some	Liquid soap
1 4 5 6 11	250	Air dried	Left in the bucket	Visibly clea	Yes, some	Liquid soap
1 2 4	10	Air dried	Poured out into the op	Visibly clea	Yes, some	Bar soap, s
1 8 11	4	Using own clothes	Left in the bucket	Visibly clea	Yes, some	Bar soap, s
1 4 5 7 8 1	120	Air dried	Left in the bucket	They appe	Yes, some	Detergent/p
1 4 5 6 11	1	Using other piece of cl	Left in the bucket	Visibly clea	Yes, alway	Other (spe
1 3 5 10	2	Did not dry	Poured out into the op	Visibly clea	Yes, some	Bathing soa
1 3 4 5 11	25	Did not dry	Left in the bucket	Visibly clea	Yes, some	Detergent/p
1 5 6 11	120	Did not dry	Poured out into the op	They appe	Yes, some	Detergent/p
1 5 6 8 12	75	Using own clothes	Left in the bucket	They appe	Yes, some	Bar soap, s
1 4 5 11	31	Did not dry	Left in the bucket	Visibly clea	Yes, some	Liquid soap
1 3 5 7 8 1	250	Using own clothes	Poured out into the op	They appe	Yes, some	Liquid soap
1 3 4	5	Air dried	Drains into the open	Visibly clea	Yes, some	Liquid soap
1 4 9 11	3	Did not dry	Left in the bucket	Visibly clea	Yes, some	Bar soap, s

1 3 11 12	2	Using other piece of cl		Poured out into the op	Visibly clea	Yes, some	Bathing soa
1 4 5 9 11	216	Using own clothes		Left in the bucket	Visibly clea	Yes, some	Liquid soap
1 4 11	5	Did not dry		Poured out into the op	Visibly clea	Yes, some	Bar soap fo
96	0	Other (Spe	Na	Other (Spe Na	Visibly clea	Yes, some	Liquid soap
1 3 10 11	3	Did not dry		Poured out into the op	Visibly clea	Yes, some	Bathing soa
1 2 3	10	Air dried		Poured out in a draina	Visibly clea	Yes, alway	Liquid soap
1 4 11	5	Did not dry		Left in the bucket	Visibly clea	Yes, some	Bar soap fo
96	0	Other (Spe	Rwfused	Poured out into the op	Visibly clea	Yes, some	Detergent/p
1 11	20	Did not dry		Left in the bucket	Visibly clea	Yes, alway	Liquid soap
1 3	5	Air dried		Poured out into the op	Visibly clea	Yes, some	Liquid soap
1 3 10 12	2	Using own clothes		Left in the bucket	Visibly clea	Yes, some	Bar soap, s
8 10	2	Did not dry		Left in the bucket	They appe	Yes, some	Bar soap, s
1 5 10	6	Did not dry		Left in the bucket	Visibly clea	Yes, some	Liquid soap
1 3 4 6 9 1	140	Using own clothes		Left in the bucket	Visibly clea	Yes, some	Detergent/p
1 4 11 12	5	Using own clothes		Left in the bucket	Visibly clea	Yes, some	Bar soap, s
1 4 12	147	Air dried		Poured out into the op	Visibly clea	No	
1 4 5 6 11	10	Using own clothes		Poured out into the op	Visibly clea	Yes, some	Bathing soa
1 2 3 4	5	Air dried		Poured out in a draina	Visibly clea	Yes, alway	Bar soap, s
1 3	2	Did not dry		Poured out into the op	Visibly clea	Yes, some	Liquid soap
1 4 5 7 11	16	Did not dry		Poured out into the op	Visibly clea	Yes, some	Liquid soap
4 5 11	40	Did not dry		Drains into the open	Visibly clea	Yes, some	Detergent/p
1 5 6 11	250	Using own clothes		Poured out into the op	Visibly clea	Yes, alway	Detergent/p
1 3 4	5	Air dried		Poured out into the op	Visibly clea	Yes, some	Liquid soap
1 3 4 6 10	32	Using own clothes		Left in the bucket	Visibly clea	Yes, some	Liquid soap
1 3 4	5	Air dried		Poured out into the op	Visibly clea	Yes, some	Bar soap, c
1 3 10 11 1	2	Using own clothes		Poured out into the op	Visibly clea	Yes, some	Bar soap, s
1 3 4 5 7 1	15	Did not dry		Poured out into the op	Visibly clea	Yes, some	Detergent/p
1 4 10	5	Did not dry		Left in the bucket	They appe	Yes, some	Bar soap fo
1 5 11	4	Did not dry		Left in the bucket	Visibly clea	Yes, some	Bathing soa
1 5 6 11	8	Did not dry		Poured in toilet/bathro	Visibly clea	Yes, some	Bathing soa
1 4 5 12	120	Air dried		Poured out into the op	They appe	Yes, some	Liquid soap
1 3 5 8 11	10	Using other piece of cl		Left in the bucket	Visibly clea	Yes, some	Bar soap, s
1 4 5 8 13	3	Did not dry		Poured out in a draina	Visibly clea	Yes, some	Liquid soap
1 3 4 11 12	8	Using own clothes		Left in the bucket	They appe	Yes, some	Bar soap fo
1 3 4	5	Air dried		Poured out into the op	Visibly clea	Yes, some	Bar soap, s
1 4 12	4	Using own clothes		Left in the bucket	Visibly clea	Yes, some	Bathing soa
1 4 5 12	5	Air dried		Drains into the open	Visibly clea	Yes, some	Bar soap fo
1 4 5 12	5	Using other piece of cl		Left in the bucket	Visibly clea	Yes, some	Bar soap fo
1 2 3 4 5 6	300	Using own clothes		Poured out into the op	Visibly clea	Yes, alway	Liquid soap
1 3 4	5	Air dried		Poured out in a draina	Visibly clea	Yes, some	Bar soap, c
1 3 4 12	5	Using own clothes		Poured out into the op	Visibly clea	Yes, some	Liquid soap
1 4 10	3	Using own clothes		Poured out in a draina	Visibly clea	Yes, some	Liquid soap
1 10 96	15	Did not dry		Left in the bucket	Visibly clea	Yes, some	Bathing soa
1 3 4 96	31	Did not dry		Drains into the open	Visibly clea	Yes, some	Liquid soap
1 4 5 10 12	5	Using own clothes		Left in the bucket	Visibly clea	Yes, some	Bathing soa
1 3 5 9	147	Air dried		Poured out into the op	Visibly clea	Yes, some	Liquid soap
1 4 6 11 12	30	Other (Spe	Rubbed on	Poured out into the op	They appe	Yes, some	Liquid soap
1 3 4	5	Air dried		Poured out into the op	Visibly clea	Yes, some	Liquid soap
1 2 3 4 5 6	15	Using other piece of cl		Poured out into the op	Visibly clea	Yes, some	Bathing soa

1 3 4	5	Air dried	Poured out into the op	Visibly clea	Yes, some	Liquid soap
1 3 5	5	Air dried	Poured out into the op	Visibly clea	Yes, some	Bar soap, c
1 3 4 10	5	Did not dry	Left in the bucket	Visibly clea	Yes, some	Bathing soa
1 4 11	5	Did not dry	Left in the bucket	Visibly clea	Yes, some	Bathing soa
1 4 11	35	Did not dry	Left in the bucket	Visibly clea	Yes, some	Liquid soap
1 3 5 6 11	30	Did not dry	Poured out into the op	Visibly clea	Yes, some	Bar soap, s
4 5 6 8 9 1	2	Did not dry	Left in the bucket	Visibly clea	Yes, some	Bar soap, s
1 4 5 10	5	Did not dry	Drains into the open	Visibly clea	Yes, some	Bar soap fo
1 4 8	8	Did not dry	Poured out in a draina	Visibly clea	Yes, some	Bathing soa
1 3 11	5	Did not dry	Left in the bucket	Visibly clea	Yes, some	Bathing soa
1 2 3 4 5 6	20	Air dried	Left in the bucket	Visibly clea	Yes, some	Bar soap, s
1 3 5 10	3	Did not dry	Drains into the open	Visibly clea	Yes, some	Bar soap, s
1 5 8 9 11	257	Using own clothes	Poured out into the op	Visibly clea	Yes, alway	Detergent/p
1 5 11	4	Did not dry	Left in the bucket	Visibly clea	Yes, some	Bathing soa
1 3 4	5	Air dried	Poured out into the op	Visibly clea	Yes, some	Bar soap, c
1 3 11 12	3	Using own clothes	Poured out into the op	Visibly clea	Yes, some	Bar soap, s
1 3 4 10	3	Using own clothes	Left in the bucket	Visibly clea	Yes, some	Bar soap, c
1 2 3	8	Air dried	Drains into the open	Visibly clea	No	No soap us
1 3	5	Air dried	Poured out into the op	Visibly clea	Yes, some	Bar soap, s
1 3 4	5	Air dried	Poured out into the op	Visibly clea	Yes, some	Liquid soap
1 4 5 11	10	Did not dry	Left in the bucket	Visibly clea	Yes, some	Bar soap fo
1 3 4	5	Air dried	Poured out into the op	Visibly clea	Yes, some	Bar soap, c
1 3 4 11	13	Did not dry	Left in the bucket	Visibly clea	Yes, alway	Liquid soap
1 4 5 11	5	Did not dry	Left in the bucket	Visibly clea	Yes, some	Bar soap fo
1 4 5 12	5	Using own clothes	Drains into the open	Visibly clea	Yes, some	Liquid soap
1 4 5 6 11	10	Air dried	Poured out into the op	They appe	Yes, some	Bar soap, s
1 4 11	3	Using own clothes	Left in the bucket	Visibly clea	Yes, some	Bar soap, s
1 3 4 96	21	Did not dry	Left in the bucket	Visibly clea	Yes, some	Liquid soap
1 3 4	5	Air dried	Poured out into the op	Visibly clea	Yes, some	Detergent/p
1 5 6 8 12	91	Air dried	Poured out into the op	Visibly clea	Yes, some	Detergent/p
1 4 7 8	120	Using other piece of cl	Left in the bucket	They appe	Yes, some	Liquid soap
1 4 5 11	21	Did not dry	Left in the bucket	Visibly clea	Yes, some	Liquid soap
1 5 6 8 12	143	Using own clothes	Poured out into the op	Visibly clea	Yes, some	Detergent/p
1 5 6 8 9 1	266	Air dried	Poured out into the op	Visibly clea	Yes, some	Detergent/p
1 3 5 9 11	36	Did not dry	Pours into the sink	Visibly clea	No	No soap us
1 3 4	5	Air dried	Left in the bucket	Visibly clea	Yes, some	Detergent/p
1 3	5	Air dried	Poured out into the op	They appe	No	
1 3 96	10	Did not dry	Left in the bucket	They appe	Yes, some	Liquid soap
1 3 4 8 12	10	Using other piece of cl	Drains into the open	They appe	Yes, some	Bar soap fo
1 3 4 96	25	Did not dry	Poured out into the op	Visibly clea	Yes, some	Liquid soap
1 4 5 8 12	5	Using own clothes	Left in the bucket	Visibly clea	Yes, some	Bar soap fo
1 5 8 12	120	Using own clothes	Left in the bucket	Visibly clea	Yes, some	Detergent/p
1 3 11 12	3	Using own clothes	Poured out into the op	Visibly clea	No	
1 3 4 11 12	10	Using own clothes	Poured out into the op	Visibly clea	Yes, some	Bar soap, s
1 2 3	5	Air dried	Poured out into the op	Visibly clea	Yes, some	Bar soap, s
1 3 4	5	Air dried	Poured out into the op	Visibly clea	Yes, some	Liquid soap
1 3	5	Air dried	Poured out into the op	Visibly clea	Yes, some	Liquid soap
1 5 11	4	Did not dry	Poured out into the op	Visibly clea	Yes, some	Bathing soa
1 3 4	5	Air dried	Poured out into the op	Visibly clea	Yes, some	Detergent/p

1 2 3 4 5 6	15	Air dried		Poured out into the op		Visibly clea	Yes, some	Bar soap, s
1 4 6 7 8 1	254	Using own clothes		Poured out into the op		Visibly clea	Yes, some	Bar soap, s
1 4 11 12	3	Using own clothes		Left in the bucket		Visibly clea	Yes, some	Bar soap, c
1 2 3	10	Air dried		Poured out into the op		Visibly clea	Yes, some	Bar soap, s
3 4 6 8 11	15	Did not dry		Poured out into the op		Visibly clea	Yes, some	Bar soap, s
1 2 3 11 12	3	Using other piece of cl		Left in the bucket		Visibly clea	Yes, some	Bathing soa
1 3 6 10 12	3	Using own clothes		Poured out into the op		They appe	Yes, some	Bar soap, s
1 3 4	5	Air dried		Left in the bucket		Visibly clea	Yes, some	Liquid soap
1 3 4	5	Air dried		Poured out into the op		Visibly clea	Yes, some	Bar soap, c
1 11	4	Did not dry		Poured out into the op		Visibly clea	Yes, some	Bathing soa
1 3 4	5	Air dried		Drains into the open		Visibly clea	Yes, some	Bar soap, c
1 4 5 10 12	5	Using own clothes		Poured out in a draina		Visibly clea	Yes, some	Bar soap, c
1 5 10	5	Did not dry		Left in the bucket		Visibly clea	Yes, some	Bathing soa
1 3 4 11	3	Did not dry		Left in the bucket		Visibly clea	Yes, some	Bathing soa
1 5 6 12	150	Using own clothes		Poured out in a draina		Visibly clea	Yes, some	Detergent/
1 4 5 6 8 1	15	Did not dry		Poured out into the op		Visibly clea	Yes, some	Bar soap fo
1 3	5	Air dried		Poured out into the op		Visibly clea	Yes, some	Liquid soap
1 12 96	10	Using other piece of cl		Left in the bucket		Visibly clea	No	
1 4 5 8 11	3	Did not dry		Left in the bucket		Visibly clea	Yes, some	Bar soap, s
1 2 5 6 11	247	Air dried		Left in the bucket		Visibly clea	Yes, some	Liquid soap
1 4 5 11	30	Did not dry		Left in the bucket		Visibly clea	Yes, some	Liquid soap
1 4 9 12 96	15	Using other piece of cl		Left in the bucket		Visibly clea	Yes, some	Bar soap, s
1 5 6 10	7	Did not dry		Left in the bucket		Visibly clea	Yes, some	Liquid soap
1 5	62	Air dried		Poured out into the op		They appe	Yes, some	Detergent/
1 3 4	5	Air dried		Poured out in a draina		Visibly clea	Yes, some	Liquid soap
1 5 10	4	Did not dry		Drains into the open		Visibly clea	Yes, some	Bar soap, c
1 2 3	5	Air dried		Poured out into the op		Visibly clea	Yes, some	Bar soap, s
1 4 11 13	5	Using own clothes		Left in the bucket		Visibly clea	Yes, some	Bar soap fo
1 3 4 11 12	15	Using own clothes		Left in the bucket		Visibly clea	Yes, some	Bar soap, s
1 5 10	6	Did not dry		Left in the bucket		Visibly clea	Yes, some	Bar soap, s
1 2 3	10	Air dried		Poured out into the op		Visibly clea	Yes, some	Bar soap, c
3 4 11	35	Did not dry		Left in the bucket		Visibly clea	Yes, some	Liquid soap
1 4 5 8 9 1	4	Did not dry		Poured out into the op		Visibly clea	Yes, some	Bathing soa
1 2 3	5	Air dried		Poured out into the op		Visibly clea	Yes, alway	Bar soap, s
1 4 6 10 12	60	Did not dry		Drains into the open		Visibly clea	Yes, some	Bar soap, c
1 4 8 11 12	25	Air dried		Poured out into the op		Visibly clea	Yes, some	Detergent/
1 4 5 12	95	Using other piece of cl		Poured out into the op		They appe	Yes, some	Liquid soap
1 6 8 11	120	Air dried		Poured out into the op		They appe	Yes, some	Detergent/
1 4 5 6 12	120	Using own clothes		Left in the bucket		They appe	Yes, some	Liquid soap
1 4 5 7	120	Using other piece of cl		Poured out into the op		They appe	Yes, some	Liquid soap
1 4 6 12	15	Using own clothes		Drains into the open		Visibly clea	Yes, some	Bathing soa
1 4 5 12	140	Air dried		Poured out into the op		They appe	Yes, alway	Liquid soap
1 3 4	5	Air dried		Poured out in a draina		Visibly clea	Yes, some	Bar soap, c
1 4 5 6 11	250	Air dried		Left in the bucket		Visibly clea	Yes, some	Bathing soa
1 4 5 12	125	Air dried		Left in the bucket		They appe	Yes, some	Bathing soa
1 12 96	20	Air dried		Left in the bucket		Visibly clea	Yes, some	Bathing soa
96	0	Other (Spe	Didn't wasl	Other (Spe	NA	They appe	Yes, alway	Liquid soap
1 3 4 5 10	40	Did not dry		Poured out in a draina		Visibly clea	Yes, some	Detergent/
1 2 3 4 5 6	15	Air dried		Poured out into the op		Visibly clea	Yes, some	Bathing soa

1 3 4 96	30	Did not dry	Poured out into the op	Visibly clea	No	
1 4 6 10 12	3	Using own clothes	Pours into the sink	Visibly clea	Yes, some	Bar soap, s
1 4 96	140	Air dried	Drains into the open	They appe	Yes, alway	Liquid soap
1 4 5 6 11	1	Did not dry	Left in the bucket	Visibly clea	Yes, some	Bathing soa
1 3 4 11	21	Did not dry	Left in the bucket	Visibly clea	Yes, some	Detergent/p
4 6 7 9	1	Did not dry	Left in the bucket	Visibly clea	Yes, some	Bar soap, s
1 2 3 4	5	Using other piece of cl	Poured out into the op	Visibly clea	Yes, some	Liquid soap
1 11 96	15	Did not dry	Left in the bucket	Visibly clea	No	
1 3 4	10	Using own clothes	Drains into the open	Visibly clea	Yes, alway	Bathing soa
1 3 4	5	Air dried	Pours into the sink	Visibly clea	Yes, some	Bar soap, s
1 4 11	4	Did not dry	Left in the bucket	They appe	Yes, some	Bathing soa
1 4 10 12	198	Using own clothes	Drains into the open	Visibly clea	Yes, some	Detergent/p
1 4 12	3	Using own clothes	Drains into the open	Visibly clea	Yes, some	Bar soap, s
1 4 6 11 13	20	Did not dry	Poured out into the op	Visibly clea	Yes, some	Liquid soap
1 11 96	10	Did not dry	Left in the bucket	Visibly clea	Yes, some	Liquid soap
1 10 96	15	Did not dry	Left in the bucket	They appe	Yes, some	Bar soap, c
1 3 4	5	Air dried	Poured out into the op	Visibly clea	Yes, some	Liquid soap
1 5 11	12	Did not dry	Left in the bucket	Visibly clea	Yes, some	Bathing soa
1 3	5	Air dried	Poured out into the op	Visibly clea	Yes, some	Bar soap, s
1 2 3 5 11	4	Using own clothes	Left in the bucket	Visibly clea	Yes, alway	Bathing soa
1 4 5 12	57	Air dried	Poured out into the op	They appe	Yes, some	Liquid soap
1 4 12	5	Using own clothes	Left in the bucket	Visibly clea	Yes, some	Bar soap fo
1 3 4	5	Air dried	Poured out into the op	Visibly clea	Yes, some	Bar soap, s
1 2 3 11	8	Did not dry	Poured in toilet/bathro	Visibly clea	Yes, some	Bathing soa
1 3 4 5 6 7	8	Did not dry	Left in the bucket	They appe	Yes, some	Bathing soa
1 3 4	5	Air dried	Poured out into the op	Visibly clea	Yes, some	Liquid soap
1 3 4 5 10	5	Air dried	Poured out into the op	Visibly clea	Yes, some	Bar soap, s
1 4 6 7 8 9	300	Using own clothes	Left in the bucket	Visibly clea	Yes, some	Bar soap, c
1 4 10 12	5	Using own clothes	Poured out into the op	Visibly clea	Yes, some	Bar soap, c
1 5 11	3	Using own clothes	Left in the bucket	Visibly clea	Yes, some	Bathing soa
1 4 5 12	10	Using own clothes	Left in the bucket	Visibly clea	Yes, some	Bar soap, s
1 4 5 11	15	Using own clothes	Left in the bucket	Visibly clea	Yes, some	Detergent/p
1 4 5 6 11	5	Air dried	Left in the bucket	They appe	Yes, some	Bar soap, s
1 3 4	5	Air dried	Poured out into the op	Visibly clea	Yes, some	Bar soap, c
1 6 8 9 11	169	Using own clothes	Poured out into the op	Visibly clea	Yes, some	Detergent/p
1 3	5	Air dried	Poured out into the op	Visibly clea	Yes, some	Bar soap, s
1 11 96	20	Did not dry	Poured out into the op	They appe	No	
1 3 4 5 11	25	Did not dry	Left in the bucket	Visibly clea	Yes, some	Liquid soap
1 11	5	Did not dry	Left in the bucket	Visibly clea	Yes, some	Liquid soap
1 3 4	5	Air dried	Poured out into the op	Visibly clea	Yes, some	Liquid soap
1 4 11	2	Did not dry	Left in the bucket	Visibly clea	Yes, alway	Liquid soap
1 4 8 9 11	358	Air dried	Poured out into the op	Visibly clea	Yes, some	Detergent/p
1 3 4 11 12	3	Using own clothes	Poured out into the op	Visibly clea	Yes, some	Bathing soa
1 3 4 5 11	35	Did not dry	Left in the bucket	Visibly clea	Yes, some	Liquid soap
1 3 4 5 11	35	Did not dry	Left in the bucket	Visibly clea	Yes, alway	Liquid soap
1 4 5 6 8 1	15	Air dried	Poured out into the op	Visibly clea	Yes, some	Bar soap, s
1 3 4 12	8	Using own clothes	Left in the bucket	Visibly clea	Yes, some	Bathing soa
1 8 10	18	Did not dry	Left in the bucket	Visibly clea	Yes, some	Bathing soa
1 3 5 6 9	15	Did not dry	Left in the bucket	Visibly clea	No	

1 5 11	4	Did not dry	Poured out in a draina	Visibly clea	Yes, some	Bar soap, s
1 3 10 12	4	Using own clothes	Poured out into the op	Visibly clea	Yes, some	Bathing soa
1 4 5 10	10	Did not dry	Left in the bucket	Visibly clea	Yes, alway	Liquid soap
1 3 4 5 96	40	Did not dry	Drains into the open	Visibly clea	Yes, some	Detergent/p
1 3 4	5	Did not dry	Left in the bucket	Visibly clea	Yes, some	Bar soap, s
1 4 10	5	Did not dry	Left in the bucket	Visibly clea	Yes, some	Bar soap fo
1 4 9 12	5	Using own clothes	Poured out into the op	Visibly clea	Yes, some	Bar soap, c
1 3 4 11	34	Did not dry	Left in the bucket	Visibly clea	Yes, some	Detergent/p
1 3 4	5	Air dried	Poured out into the op	Visibly clea	Yes, some	Bar soap, c
1 4 5 12	5	Using own clothes	Left in the bucket	Visibly clea	Yes, some	Bar soap fo
1 11 96	10	Did not dry	Poured out in a draina	They appe	No	
1 3 4	5	Air dried	Poured out into the op	Visibly clea	Yes, some	Bar soap, s
1 2 4 5 6 7	15	Air dried	Poured out into the op	Visibly clea	Yes, some	Bar soap, s
1 3 4	5	Air dried	Poured out into the op	Visibly clea	Yes, some	Detergent/p
1 4 11 12	3	Using own clothes	Left in the bucket	Visibly clea	Yes, some	Bar soap, c
1 3 4 5 8	10	Did not dry	Left in the bucket	Visibly clea	Yes, some	Bar soap, s
1 4 5 6 12	15	Air dried	Drains into the open	Visibly clea	Yes, some	Bar soap, s
1 3 4 5 11	270	Air dried	Left in the bucket	Visibly clea	Yes, alway	Liquid soap
1 4 8 9 11	1	Using own clothes	Left in the bucket	Visibly clea	Yes, alway	Bar soap, s
1 2 3 4 5 6	20	Air dried	Poured out into the op	Visibly clea	Yes, alway	Bathing soa
1 5 6 8 12	140	Air dried	Left in the bucket	They appe	Yes, some	Detergent/p
1 4 8 9 12	147	Using own clothes	Poured out into the op	They appe	Yes, some	Liquid soap
1 3	10	Did not dry	Left in the bucket	Visibly clea	Yes, some	Bar soap, s
1 2 3 5 7 9	43	Air dried	Poured out into the op	Visibly clea	Yes, some	Liquid soap
1 2 4 5 7 8	15	Air dried	Poured out into the op	Visibly clea	Yes, some	Bar soap, s
1 2 3 4 5 6	45	Using other piece of cl	Poured out into the op	Visibly clea	Yes, alway	Bathing soa
1 2 3 4 6 8	10	Using own clothes	Left in the bucket	Visibly clea	Yes, alway	Bar soap, s
2 3 4 5 8 1	31	Air dried	Poured out into the op	Visibly clea	Yes, alway	Bar soap, s
1 2 3 10 12	3	Using other piece of cl	Pours into the sink	Visibly clea	Yes, alway	Bar soap, s
1 2 3 4 5 6	25	Using other piece of cl	Poured out into the op	Visibly clea	Yes, some	Bar soap, s
1 2 3 4 5 8	300	Using own clothes	Poured out in a draina	Visibly clea	Yes, some	Bathing soa
1 2 5 10 12	5	Air dried	Left in the bucket	Visibly clea	Yes, alway	Liquid soap
1 2 3 4 5 9	25	Air dried	Poured out into the op	Visibly clea	Yes, some	Bathing soa
1 3 10	2	Did not dry	Poured out into the op	They appe	Yes, some	Bar soap, s
1 2 3 4 5 7	36	Air dried	Poured out into the op	Visibly clea	Yes, alway	Liquid soap
1 2 11 12 9	15	Using own clothes	Drains into the open	Visibly clea	Yes, alway	Liquid soap
1 2 5 11	7	Did not dry	Poured in toilet/bathro	Visibly clea	Yes, alway	Bathing soa
1 2 5 11	6	Did not dry	Left in the bucket	Visibly clea	Yes, some	Bar soap, s
1 2 3 4 5 6	20	Using own clothes	Left in the bucket	Visibly clea	Yes, some	Bathing soa
1 2 3 5 6 1	6	Using own clothes	Poured out into the op	Visibly clea	Yes, alway	Bathing soa
1 2 3 6 10	8	Using other piece of cl	Pours into the sink	Visibly clea	Yes, alway	Bar soap, s
1 2 3 5 8 1	17	Using other piece of cl	Poured out in a draina	Visibly clea	Yes, some	Bar soap, s
1 4 7 9 10	5	Air dried	Pours into the sink	Visibly clea	Yes, alway	Bathing soa
1 2 5 6 10	153	Using own clothes	Poured out into the op	They appe	Yes, some	Detergent/p
1 2 3 4 6 7	20	Using own clothes	Left in the bucket	Visibly clea	Yes, some	Bar soap fo
1 2 3 4 8 1	15	Using own clothes	Left in the bucket	Visibly clea	Yes, some	Bathing soa
1 2 4 7 10	39	Air dried	Poured out into the op	Visibly clea	Yes, some	Detergent/p
1 2 3 4 5 6	8	Air dried	Poured out into the op	Visibly clea	Yes, alway	Liquid soap
1 2 3 11 12	3	Using own clothes	Poured out into the op	They appe	Yes, some	Bathing soa

1 2 3 4 5 6	25	Using other piece of cl	Left in the bucket	Visibly clea	Yes, alway	Bathing soa
1 2 3 4 5 1	15	Using other piece of cl	Left in the bucket	Visibly clea	Yes, some	Liquid soap
1 2 3 4 6 8	8	Air dried	Poured out into the op	Visibly clea	Yes, alway	Bar soap, s
1 2 3 4 5 6	37	Using other piece of cl	Poured out into the op	Visibly clea	Yes, some	Bar soap, s
1 2 3 5 6 1	5	Using own clothes	Poured out into the op	Visibly clea	Yes, alway	Bar soap, c
1 2 4 6 11	4	Did not dry	Poured out into the op	Visibly clea	Yes, some	Bathing soa
1 2 3 6 7 8	27	Using own clothes	Left in the bucket	Visibly clea	Yes, some	Bathing soa
1 2 3 4 5 1	3	Using own clothes	Left in the bucket	Visibly clea	Yes, some	Bathing soa
12 96	10	Using own clothes	Left in the bucket	Visibly clea	Yes, some	Detergent/p
1 2 3 4 5 9	10	Using own clothes	Poured out into the op	Visibly clea	Yes, alway	Liquid soap
1 2 3 4 10	5	Using own clothes	Poured out into the op	Visibly clea	Yes, some	Bar soap, c
1 2 3 4 5 1	125	Did not dry	Poured out into the op	Visibly clea	Yes, some	Liquid soap
1 2 3 4	5	Air dried	Poured out into the op	Visibly clea	Yes, some	Bar soap, s
1 2 3 4 5 6	8	Air dried	Poured out into the op	Visibly clea	Yes, alway	Bar soap, s
1 2 3 5 7 9	20	Air dried	Poured out into the op	Visibly clea	Yes, some	Liquid soap
1 2 4 5 6 8	25	Using other piece of cl	Left in the bucket	Visibly clea	Yes, alway	Bar soap, s
1 2 4 5 6 7	25	Air dried	Poured out into the op	Visibly clea	Yes, some	Liquid soap
1 2 3 4 11	5	Air dried	Poured out in a draina	Visibly clea	Yes, some	Bar soap, s
1 2 3 4 5 7	18	Air dried	Poured out into the op	Visibly clea	Yes, alway	Bar soap, s
1 2 3 4 12	10	Using own clothes	Poured out into the op	Visibly clea	Yes, some	Bar soap fo
1 2 3 4	10	Air dried	Poured out into the op	Visibly clea	Yes, alway	Bar soap, s
1 2 3 4 5 6	60	Air dried	Poured out into the op	Visibly clea	Yes, some	Bathing soa
1 2 3 4 6 1	60	Using other piece of cl	Poured out in a draina	Visibly clea	Yes, alway	Bar soap fo
1 2 3 4 5 1	31	Did not dry	Poured out into the op	Visibly clea	Yes, alway	Bar soap, s
1 2 3 4 5 6	2	Did not dry	Left in the bucket	Visibly clea	Yes, alway	Liquid soap
1 2 3 4 5 6	50	Using own clothes	Poured out into the op	Visibly clea	Yes, some	Bathing soa
1 2 3 6 7 1	30	Did not dry	Poured out into the op	Visibly clea	Yes, alway	Liquid soap
1 2 5 7 8 1	250	Using own clothes	Poured out into the op	Visibly clea	Yes, some	Liquid soap
1 2 3 4 5 6	31	Using own clothes	Poured out into the op	They appe	Yes, some	Bar soap, s
1 3 4 5 6 8	20	Air dried	Poured out into the op	Visibly clea	Yes, some	Bar soap, s
1 2 3 4 5 6	10	Did not dry	Left in the bucket	Visibly clea	Yes, some	Liquid soap
1 2 3 4 11	30	Did not dry	Left in the bucket	Visibly clea	Yes, alway	Bar soap fo
1 2 3 4 6 9	10	Using own clothes	Left in the bucket	Visibly clea	Yes, alway	Bathing soa
1 2 3	5	Air dried	Poured out into the op	Visibly clea	Yes, some	Liquid soap
1 2 4 6 8 1	3	Using own clothes	Left in the bucket	Visibly clea	Yes, alway	Bathing soa
1 3 6 9 11	20	Air dried	Poured out into the op	Visibly clea	Yes, some	Bar soap, s
1 2 3 4 11	30	Did not dry	Left in the bucket	Visibly clea	Yes, some	Bar soap, s
1 2 3 5 6 1	30	Did not dry	Drains into the open	Visibly clea	Yes, alway	Bar soap, s
1 2 3 4 5 1	10	Using own clothes	Left in the bucket	Visibly clea	Yes, some	Bar soap, c
1 2 3 4 5 7	10	Air dried	Poured out into the op	Visibly clea	Yes, alway	Bar soap, s
1 2 3 4 5 1	3	Using other piece of cl	Left in the bucket	They appe	Yes, some	Bar soap fo
1 2 3 5 11	5	Using other piece of cl	Left in the bucket	Visibly clea	Yes, some	Bar soap, c
1 2 3 4 5 6	15	Air dried	Poured out into the op	Visibly clea	Yes, some	Bar soap, s
1 2 3 4 8 9	1	Did not dry	Left in the bucket	Visibly clea	Yes, alway	Bar soap fo
1 2 3 4 5 6	5	Using own clothes	Drains into the open	Visibly clea	Yes, some	Liquid soap
96	0	Other (Spe did not den	Other (Spe did not der	Visibly clea	Yes, alway	Bar soap fo
1 2 3 4 5 1	10	Using own clothes	Poured out in a draina	Visibly clea	Yes, some	Bar soap fo
1 2 5 8 10	30	Air dried	Poured out in a draina	Visibly clea	Yes, alway	Bathing soa
1 2 3 4 5 6	15	Did not dry	Poured out into the op	Visibly clea	Yes, some	Bathing soa

2 3 4 5 11	49	Using own clothes		Left in the bucket		Visibly clea	Yes, some	Liquid soap
1 2 4 11 12	5	Using own clothes		Poured out into the op		Visibly clea	Yes, alway	Bar soap, s
1 2 3 5 8 1	13	Did not dry		Poured out in a draina		Visibly clea	Yes, alway	Bar soap fo
1 2 3 4 5 6	30	Air dried		Poured out into the op		Visibly clea	Yes, some	Bar soap, s
1 2 5 10	6	Did not dry		Pours into the sink		Visibly clea	Yes, alway	Bathing soa
1 2 3 4 5 6	15	Air dried		Poured out into the op		Visibly clea	Yes, some	Bar soap, s
1 2 3 4 5 6	20	Using other piece of cl		Pours into the sink		Visibly clea	Yes, alway	Liquid soap
2 3 4 10	10	Did not dry		Pours into the sink		Visibly clea	Yes, alway	Liquid soap
1 2 3 4 5 6	12	Did not dry		Left in the bucket		Visibly clea	Yes, alway	Bar soap fo
1 2 3 5 8 9	15	Using own clothes		Left in the bucket		Visibly clea	Yes, some	Bathing soa
1 2 3 4	15	Did not dry		Left in the bucket		Visibly clea	Yes, alway	Bathing soa
1 2 5 10	10	Did not dry		Other (Spe	She fetche	Visibly clea	Yes, some	Liquid soap
1 2 3 4 5 7	25	Did not dry		Poured out in a draina		Visibly clea	Yes, alway	Liquid soap
1 2 3 4 7 8	30	Did not dry		Poured out into the op		Visibly clea	Yes, alway	Detergent/p
1 2 3 4 5 6	240	Did not dry		Left in the bucket		Visibly clea	Yes, alway	Bar soap, c
1 2 3 4 11	30	Using other piece of cl		Left in the bucket		Visibly clea	Yes, alway	Bathing soa
1 2 3 4 5 1	10	Using other piece of cl		Left in the bucket		Visibly clea	Yes, alway	Bar soap, c
1 2 3 4 5 6	12	Air dried		Poured out into the op		Visibly clea	Yes, alway	Liquid soap
1 2 4 5 8 1	30	Using own clothes		Poured out in a draina		Visibly clea	Yes, alway	Bathing soa
1 2 3 4 5 6	15	Using other piece of cl		Poured out into the op		Visibly clea	Yes, some	Bar soap, s
1 2 3 4 5 1	10	Using other piece of cl		Drains into the open		Visibly clea	Yes, alway	Bar soap, c
1 2 3 4 10	8	Using own clothes		Left in the bucket		Visibly clea	Yes, alway	Bar soap fo
1 2 3 4 5 7	8	Air dried		Poured out into the op		Visibly clea	Yes, alway	Bar soap, s
1 2 3 4 6 1	10	Using own clothes		Poured out into the op		Visibly clea	Yes, some	Bathing soa
1 2 3 4 5 6	12	Using own clothes		Poured out into the op		Visibly clea	Yes, alway	Bar soap, s
1 2 3 4 9 1	60	Using other piece of cl		Poured out into the op		Visibly clea	Yes, some	Bathing soa
1 2 3 4 5 6	16	Air dried		Poured out into the op		Visibly clea	Yes, some	Bar soap, s
1 2 3 5 11	5	Did not dry		Poured out into the op		Visibly clea	Yes, some	Bathing soa
1 2 3 4 6 7	15	Air dried		Poured out into the op		Visibly clea	Yes, alway	Bar soap, s
1 2 3 4 8 9	10	Air dried		Poured out into the op		Visibly clea	Yes, alway	Bathing soa
1 2 3 4 5 6	13	Did not dry		Pours into the sink		Visibly clea	Yes, some	Bar soap, s
1 2 3 4 5 6	20	Using own clothes		Left in the bucket		Visibly clea	Yes, some	Liquid soap
1 2 4 6 7 9	2	Using own clothes		Left in the bucket		Visibly clea	Yes, some	Bar soap, s
1 2 4 5 9 1	20	Using other piece of cl		Left in the bucket		Visibly clea	Yes, alway	Liquid soap
1 2 5 8 11	15	Using own clothes		Left in the bucket		Visibly clea	Yes, some	Bathing soa
1 2 3 4 5 8	15	Air dried		Left in the bucket		Visibly clea	Yes, some	Bar soap fo
1 2 3	5	Did not dry		Left in the bucket		Visibly clea	Yes, some	Bar soap, s
1 2 3 4 5 6	15	Air dried		Poured out into the op		Visibly clea	Yes, alway	Bar soap, s
1 2 3 4 5 1	2	Did not dry		Poured out into the op		Visibly clea	Yes, alway	Bar soap, c
2 3 4 5 11	42	Did not dry		Poured in toilet/bathro		Visibly clea	Yes, some	Bar soap, c
1 2 4 9 12	20	Using own clothes		Left in the bucket		Visibly clea	Yes, alway	Bathing soa
1 2 4 5	10	Using own clothes		Left in the bucket		Visibly clea	Yes, alway	Bar soap, s
1 2 3 4 6 1	45	Did not dry		Poured out in a draina		Visibly clea	Yes, some	Bathing soa
1 2 3 4 5 1	3	Using own clothes		Poured out into the op		Visibly clea	Yes, alway	Bar soap fo
1 2 3 4 11	60	Air dried		Poured out into the op		Visibly clea	Yes, some	Bathing soa
1 2 3 4 9 1	20	Using other piece of cl		Poured out into the op		Visibly clea	Yes, some	Bathing soa
1 2 3 4 6 9	10	Using other piece of cl		Left in the bucket		Visibly clea	Yes, alway	Bathing soa
1 2 4 5 11	47	Did not dry		Left in the bucket		Visibly clea	Yes, alway	Liquid soap
4 5 96	5	Other (Spe	did not den	Other (Spe	didn't demo	Visibly clea	Yes, some	Bar soap fo

1 2 3 4 8 1	20	Using own clothes		Poured out into the op		Visibly clea	Yes, alway	Bar soap, c
1 2 3 4 5 6	40	Using other piece of cl		Drains into the open		Visibly clea	Yes, some	Bathing soa
1 2 3 4 5 6	30	Air dried		Poured in toilet/bathro		Visibly clea	Yes, some	Bathing soa
1 2 3 4 5 6	8	Air dried		Poured out into the op		Visibly clea	Yes, alway	Bar soap, s
1 2 3 4 6 9	14	Did not dry		Poured out into the op		Visibly clea	Yes, alway	Bar soap, s
1 2 4 5 7 1	10	Using other piece of cl		Poured out in a draina		They appe	Yes, alway	Bathing soa
1 2 4 5 7 9	20	Air dried		Poured out into the op		Visibly clea	Yes, some	Bar soap, s
96	0	Other (Spe	no water tc	Other (Spe	no water tc	Visibly clea	Yes, alway	Bar soap, c
1 2 5 11 12	13	Using other piece of cl		Left in the bucket		Visibly clea	Yes, some	Bathing soa
1 2 3 4 6 7	8	Air dried		Poured out into the op		Visibly clea	Yes, alway	Bar soap, s
1 2 3 4 5 6	12	Air dried		Poured out into the op		Visibly clea	Yes, alway	Bar soap, s
2 3 4 96	32	Did not dry		Poured out into the op		Visibly clea	Yes, some	Liquid soap
1 2 4 11	10	Using own clothes		Left in the bucket		They appe	Yes, alway	Liquid soap
1 2 4 5 7 9	20	Air dried		Poured out into the op		Visibly clea	Yes, some	Bar soap, s
1 2 3 4 5 1	15	Did not dry		Left in the bucket		Visibly clea	Yes, some	Bar soap, s
1 2 4 5 6 7	18	Did not dry		Pours into the sink		Visibly clea	Yes, some	Bar soap, c
2 3 4 5 11	47	Did not dry		Left in the bucket		Visibly clea	Yes, some	Liquid soap
1 2 3 4 5 6	30	Air dried		Poured out into the op		Visibly clea	Yes, alway	Bar soap, s
1 2 3 4 5 7	43	Air dried		Poured out into the op		Visibly clea	Yes, alway	Liquid soap
1 2 4 5 6 9	190	Using own clothes		Poured out into the op		Visibly clea	Yes, some	Liquid soap
1 2 5 6 7 8	20	Did not dry		Left in the bucket		Visibly clea	Yes, alway	Bar soap, s
1 2 3 4 6 7	20	Air dried		Poured out into the op		Visibly clea	Yes, alway	Liquid soap
1 2 3 4	10	Air dried		Poured out into the op		Visibly clea	Yes, some	Detergent/p
1 2 3 4 5 6	5	Using other piece of cl		Poured out in a draina		Visibly clea	Yes, alway	Bar soap, c
1 2 3 6 11	5	Did not dry		Left in the bucket		Visibly clea	Yes, alway	Bathing soa
1 2 3 4 8 1	10	Using own clothes		Poured out into the op		Visibly clea	Yes, some	Bar soap fo
2 3 4 11 13	42	Did not dry		Poured out into the op		Visibly clea	Yes, some	Liquid soap
1 2 3 4 11	4	Using own clothes		Poured out into the op		Visibly clea	Yes, alway	Bathing soa
1 2 3 5 6 8	15	Air dried		Left in the bucket		Visibly clea	Yes, some	Bar soap, c
1 2 4 5 7 9	20	Air dried		Poured out into the op		Visibly clea	Yes, some	Bathing soa
1 2 3 4 5 7	20	Air dried		Poured out into the op		Visibly clea	Yes, some	Bar soap, s
1 2 3 4 8 9	3	Air dried		Left in the bucket		Visibly clea	Yes, alway	Bar soap, c
1 2 3 4 5 6	30	Air dried		Poured out into the op		Visibly clea	Yes, some	Bar soap, c
1 2 3 6 7 9	30	Did not dry		Poured out into the op		Visibly clea	Yes, alway	Bar soap, s
1 2 3 5 10	4	Did not dry		Left in the bucket		Visibly clea	Yes, alway	Bar soap, s
1 2 3 4 5 6	36	Did not dry		Poured in toilet/bathro		Visibly clea	Yes, alway	Detergent/p
1 2 3 4 5 6	7	Air dried		Poured out into the op		Visibly clea	Yes, alway	Bar soap, s
1 2 5 6 9 1	10	Did not dry		Left in the bucket		Visibly clea	Yes, some	Bathing soa
2 3 4 11 12	15	Using own clothes		Poured out into the op		Visibly clea	Yes, some	Bar soap, s
1 2 3 4 5 6	50	Using other piece of cl		Poured out in a draina		Visibly clea	Yes, some	Bathing soa
1 2 3 4 11	6	Using other piece of cl		Pours into the sink		Visibly clea	Yes, alway	Liquid soap
1 2 3 5 11	11	Did not dry		Left in the bucket		Visibly clea	Yes, some	Liquid soap
1 2 3 4 5 6	22	Did not dry		Poured out into the op		Visibly clea	Yes, some	Liquid soap
1 2 3 4 5 6	20	Using other piece of cl		Poured out into the op		They appe	Yes, alway	Bar soap, s
1 2 3 4 5 6	40	Air dried		Poured out into the op		Visibly clea	Yes, some	Bathing soa
1 2 3 4 5 6	4	Using other piece of cl		Poured out into the op		Visibly clea	Yes, alway	Bathing soa
1 4 5 11	10	Did not dry		Left in the bucket		Visibly clea	Yes, alway	Bar soap fo
1 2 3 4 5 1	10	Using own clothes		Pours into the sink		Visibly clea	Yes, some	Liquid soap
1 2 3 4 10	3	Using other piece of cl		Left in the bucket		Visibly clea	Yes, alway	Bar soap, s

1 2 3 4 5 1	6	Did not dry	Poured out into the op	Visibly clea	Yes, alway	Bar soap, s
1 2 3 4 5 6	15	Air dried	Poured out into the op	Visibly clea	Yes, alway	Liquid soap
1 2 3 4 5 6	3	Using own clothes	Left in the bucket	Visibly clea	Yes, alway	Bathing soa
1 2 3 4 5 1	10	Air dried	Poured out into the op	Visibly clea	Yes, alway	Bar soap, s
1 2 3 4 5 1	15	Using other piece of cl	Left in the bucket	Visibly clea	Yes, alway	Bar soap fo
1 2 3	5	Using serviettes/tissue	Poured out in a draina	Visibly clea	Yes, some	Liquid soap
2 4 11 13	50	Using own clothes	Drains into the open	Visibly clea	Yes, some	Liquid soap
1 2 3 4 5 6	56	Using other piece of cl	Left in the bucket	Visibly clea	Yes, alway	Liquid soap
1 2 3 4 7 8	5	Using other piece of cl	Left in the bucket	Visibly clea	Yes, alway	Liquid soap
1 5 8 9 11	10	Did not dry	Left in the bucket	Visibly clea	Yes, alway	Bar soap, s
1 2 3 5 6 7	20	Air dried	Poured out into the op	Visibly clea	Yes, some	Bar soap, s
1 2 3 4 6 7	6	Air dried	Poured out into the op	Visibly clea	Yes, alway	Bar soap, s
1 2 3 4 11	10	Using own clothes	Left in the bucket	Visibly clea	Yes, some	Liquid soap
1 2 3 4	10	Using own clothes	Poured out into the op	Visibly clea	Yes, some	Detergent/p
1 2 3 4 5 6	20	Air dried	Poured out into the op	Visibly clea	Yes, some	Bathing soa
1 2 3 4 5 6	6	Air dried	Poured out into the op	Visibly clea	Yes, alway	Bar soap, s
1 2 4 6 7 8	20	Air dried	Pours into the sink	Visibly clea	Yes, some	Liquid soap
1 2 3 4 5 1	10	Did not dry	Pours into the sink	Visibly clea	Yes, alway	Bar soap, s
1 2 3 4 5 6	15	Air dried	Poured out into the op	Visibly clea	Yes, some	Bar soap, s
1 2 3 4 5 6	40	Using other piece of cl	Poured out into the op	Visibly clea	Yes, some	Bathing soa
1 2 3 4 8 1	1	Air dried	Left in the bucket	Visibly clea	Yes, some	Bar soap, s
1 2 3 4 5 6	20	Did not dry	Left in the bucket	Visibly clea	Yes, alway	Bathing soa
1 2 3 4 11	4	Did not dry	Poured out in a draina	Visibly clea	Yes, some	Bar soap, c
1 2 3 4 5 6	15	Air dried	Poured out into the op	Visibly clea	Yes, alway	Liquid soap
1 2 3 5 6 1	20	Air dried	Left in the bucket	Visibly clea	Yes, alway	Bar soap, s
1 2 3 4 5 1	15	Air dried	Poured out into the op	Visibly clea	Yes, alway	Bar soap, s
2 3 4 5 11	62	Did not dry	Left in the bucket	Visibly clea	Yes, some	Liquid soap
1 2 4 7 11	15	Did not dry	Poured out in a draina	Visibly clea	Yes, alway	Bathing soa
1 2 3 4 5 9	10	Other (Spe demonstra	Other (Spe demonstra	Visibly clea	Yes, alway	Bar soap, c
1 2 3 4 5 6	20	Using other piece of cl	Other (Specify)	Visibly clea	Yes, some	Bar soap, c
1 2 3 4 6 1	8	Air dried	Left in the bucket	Visibly clea	Yes, alway	Bar soap, s
1 2 3 4 7 8	3	Did not dry	Left in the bucket	Visibly clea	Yes, some	Bar soap, c
1 2 3 4 6 1	30	Using other piece of cl	Left in the bucket	Visibly clea	Yes, some	Bar soap fo
1 2 3 4	20	Did not dry	Left in the bucket	Visibly clea	Yes, alway	Bathing soa
1 2 3 4	5	Air dried	Poured out into the op	Visibly clea	Yes, some	Liquid soap
1 2 3 4 9 1	2	Using other piece of cl	Poured out into the op	Visibly clea	Yes, alway	Bar soap, c
2 3 5 12	5	Did not dry	Poured out into the op	Visibly clea	Yes, some	Bar soap fo
1 2 3 4 5 6	10	Air dried	Left in the bucket	Visibly clea	Yes, some	Bar soap, c
1 2 3 5 6 1	10	Did not dry	Left in the bucket	Visibly clea	Yes, alway	Bathing soa
1 2 6 8 10	20	Using other piece of cl	Poured out in a draina	Visibly clea	Yes, alway	Bar soap, s
1 2 3 5 6 1	45	Did not dry	Pours into the sink	Visibly clea	Yes, alway	Liquid soap
1 2 4 5 6 8	20	Using other piece of cl	Left in the bucket	Visibly clea	Yes, alway	Bar soap, s
1 2 3 4	8	Did not dry	Left in the bucket	Visibly clea	Yes, alway	Bathing soa
1 2 5 6 10	10	Did not dry	Left in the bucket	Visibly clea	Yes, some	Bathing soa
1 2 4 5 6 8	20	Air dried	Poured out into the op	Visibly clea	Yes, some	Bar soap, s
2 3 4 9 10	10	Using own clothes	Poured out in a draina	Visibly clea	Yes, some	Liquid soap
1 2 3 4 8 9	3	Using own clothes	Poured out in a draina	Visibly clea	Yes, alway	Bar soap, s
2 4 5 11	30	Did not dry	Left in the bucket	Visibly clea	Yes, some	Liquid soap
1 2 3 4 5 6	15	Using other piece of cl	Poured out into the op	Visibly clea	Yes, some	Liquid soap

1 5 10	5	Air dried	Left in the bucket		They appe	Yes, some	Bar soap, s
1 2 3 4 6 1	15	Air dried	Poured out into the op		Visibly clea	Yes, some	Bar soap, c
1 3 4 5 6 1	10	Did not dry	Other (Spe	did not der	Visibly clea	Yes, alway	Bar soap, s
1 2 3 4 11	4	Did not dry	Left in the bucket		Visibly clea	Yes, some	Bar soap fc
1 2 5 10	4	Did not dry	Drains into the open		Visibly clea	Yes, alway	Liquid soap
1 2 3 6 11	3	Using own clothes	Left in the bucket		Visibly clea	Yes, some	Detergent/p
1 2 3 4 5 6	15	Air dried	Poured out into the op		Visibly clea	Yes, some	Bar soap, s
1 2 3 4 11	10	Did not dry	Left in the bucket		Visibly clea	Yes, some	Bathing soa
1 2 3 4 11	45	Did not dry	Left in the bucket		Visibly clea	Yes, some	Liquid soap
1 2 3 4 5 1	15	Using own clothes	Left in the bucket		Visibly clea	Yes, some	Bar soap, c
1 2 3 5 6 7	36	Air dried	Pours into the sink		Visibly clea	Yes, alway	Bar soap, c
1 11 12	10	Did not dry	Left in the bucket		Visibly clea	Yes, some	Bathing soa
1 2 3 6 7 9	31	Did not dry	Poured out in a draina		Visibly clea	Yes, alway	Detergent/p
1 2 3 4 5 6	10	Air dried	Poured out into the op		Visibly clea	Yes, alway	Bar soap, s
1 2 4 5 6 1	52	Did not dry	Pours into the sink		Visibly clea	Yes, alway	Liquid soap
1 2 3 4 11	30	Did not dry	Left in the bucket		Visibly clea	Yes, some	Bar soap fc
1 2 11	8	Using own clothes	Left in the bucket		Visibly clea	Yes, some	Bathing soa
1 2 3 4 11	5	Did not dry	Drains into the open		Visibly clea	Yes, some	Bar soap, c
1 2 3 4	5	Air dried	Poured out into the op		Visibly clea	Yes, some	Liquid soap
3 4 5 10 13	15	Air dried	Poured out into the op		Visibly clea	Yes, some	Liquid soap
1 2 3 4 5 6	20	Using other piece of cl	Poured out into the op		Visibly clea	Yes, some	Bar soap, s
1 2 5 8 9 1	52	Did not dry	Poured out into the op		Visibly clea	Yes, some	Liquid soap
1 2 3 9 11	1	Using own clothes	Left in the bucket		Visibly clea	Yes, alway	Bar soap, s
1 2 3 4 5 6	20	Air dried	Poured out into the op		Visibly clea	Yes, alway	Bar soap, s
1 2 4 6 9 1	20	Using own clothes	Left in the bucket		Visibly clea	Yes, alway	Bathing soa
1 2 3 4 10	5	Did not dry	Drains into the open		Visibly clea	Yes, some	Bar soap, s
1 2 3 4 5 7	50	Did not dry	Left in the bucket		Visibly clea	Yes, alway	Liquid soap
1 2 3 4 5 8	30	Using other piece of cl	Poured out into the op		Visibly clea	Yes, some	Liquid soap
1 2 3 4 11	10	Did not dry	Left in the bucket		Visibly clea	Yes, some	Bathing soa
96	0	Other (Spe did not der	Other (Spe	did not der	Visibly clea	Yes, alway	Bar soap, c
1 2 3 4 10	10	Using own clothes	Poured out into the op		Visibly clea	Yes, some	Bar soap fc
1 2 3 4 5 6	15	Using own clothes	Poured out into the op		Visibly clea	Yes, some	Bar soap, s
2 3 4 5 6 8	10	Did not dry	Left in the bucket		Visibly clea	Yes, alway	Liquid soap
1 2 3 4 5 6	20	Air dried	Poured out into the op		Visibly clea	Yes, some	Bar soap, s
1 2 3 4 5 6	20	Using other piece of cl	Poured out into the op		Visibly clea	Yes, some	Bar soap, c
1 2 3 4 5 6	20	Using own clothes	Poured out into the op		Visibly clea	Yes, some	Liquid soap
1 2 3 4 5 6	9	Using own clothes	Poured out in a draina		Visibly clea	Yes, alway	Liquid soap
1 2 3 4 6 8	60	Using other piece of cl	Left in the bucket		Visibly clea	Yes, some	Bar soap, c
1 2 3 4 5 6	8	Air dried	Poured out into the op		Visibly clea	Yes, alway	Bar soap, s
1 2 3 4 6 1	30	Air dried	Poured out into the op		Visibly clea	Yes, alway	Bar soap, s
1 2 3 5 7 8	15	Air dried	Poured out into the op		Visibly clea	Yes, some	Bar soap, s
1 2 3 4 8 9	24	Did not dry	Poured out into the op		Visibly clea	Yes, alway	Detergent/p
1 2 3 4 5 6	8	Air dried	Poured out into the op		Visibly clea	Yes, alway	Bar soap, s
2 3 4 11	10	Air dried	Left in the bucket		Visibly clea	Yes, alway	Bar soap, c
1 2 3 4 5 6	12	Air dried	Poured out into the op		Visibly clea	Yes, alway	Bar soap, s
1 2 3 4	5	Air dried	Poured out into the op		Visibly clea	Yes, alway	Detergent/p
1 2 4 6 7 8	20	Air dried	Poured out into the op		Visibly clea	Yes, some	Bar soap, s
1 2 3 4 5 6	15	Air dried	Poured out into the op		Visibly clea	Yes, alway	Bar soap, s
1 2 3 4 5 6	10	Using other piece of cl	Poured out into the op		Visibly clea	Yes, alway	Bar soap, s

2 3 5 6 10	10	Using serviettes/tissue	Pours into the sink		Visibly clea	Yes, some	Liquid soap
1 2 3 4 9	20	Did not dry	Left in the bucket		Visibly clea	Yes, alway	Bathing soa
1 2 4 11 13	45	Did not dry	Drains into the open		Visibly clea	Yes, alway	Liquid soap
1 2 3 4 11	3	Using own clothes	Poured out into the op		Visibly clea	Yes, alway	Bathing soa
1 2 3 4 6 9	45	Using other piece of cl	Poured out into the op		Visibly clea	Yes, alway	Bar soap, c
1 2 3 4 5 6	8	Air dried	Poured out into the op		Visibly clea	Yes, alway	Bar soap, s
1 2 3 4 5 6	20	Air dried	Poured out into the op		Visibly clea	Yes, some	Bar soap, s
1 2 3 4 11	10	Using other piece of cl	Drains into the open		Visibly clea	Yes, some	Bar soap fo
1 2 4 10 12	5	Using other piece of cl	Left in the bucket		Visibly clea	Yes, alway	Liquid soap
1 2 5 8 10	15	Air dried	Poured out in a draina		Visibly clea	Yes, alway	Liquid soap
1 2 3 4 10	5	Using own clothes	Poured out in a draina		Visibly clea	Yes, alway	Bar soap fo
96	0	Did not dry	Other (Spe	N/a	Visibly clea	Yes, alway	Bar soap fo
1 2 3 4 6 7	28	Using own clothes	Pours into the sink		Visibly clea	Yes, alway	Liquid soap
1 2 3 4	5	Did not dry	Left in the bucket		Visibly clea	Yes, alway	Bar soap, s
1 2 3 5 11	12	Did not dry	Drains into the open		Visibly clea	Yes, some	Bathing soa
1 2 3 4 6 1	60	Using other piece of cl	Poured out in a draina		Visibly clea	Yes, some	Bathing soa
1 2 4 11	30	Did not dry	Left in the bucket		Visibly clea	Yes, alway	Bar soap, c
1 2 3 4 5 7	12	Air dried	Poured out into the op		Visibly clea	Yes, some	Bathing soa
2 3 4 5 11	52	Did not dry	Poured out in a draina		Visibly clea	Yes, alway	Liquid soap
2 3 4 5 6 1	10	Air dried	Left in the bucket		Visibly clea	Yes, some	Liquid soap
1 2 3 4 5 6	15	Air dried	Poured out into the op		Visibly clea	Yes, some	Bar soap, s
2 3 4 5 11	32	Did not dry	Left in the bucket		Visibly clea	Yes, alway	Bar soap, c
1 2 5 11 12	146	Using own clothes	Left in the bucket		Visibly clea	Yes, some	Liquid soap
1 2 3 4 5 8	10	Air dried	Poured out into the op		Visibly clea	Yes, alway	Bar soap, s
2 3 4 5 8 1	8	Did not dry	Poured out into the op		They appe	Yes, alway	Bar soap, s
2 3 5 6 7 8	72	Using own clothes	Poured out into the op		Visibly clea	Yes, alway	Liquid soap
2 4 11 12	147	Using own clothes	Poured out into the op		Visibly clea	Yes, some	Liquid soap
1 2 3 5 6 7	20	Air dried	Poured out into the op		Visibly clea	Yes, some	Bathing soa
1 2 3 5 7 8	46	Did not dry	Poured out into the op		Visibly clea	Yes, alway	Bar soap, s
1 2 3 4 5 6	61	Did not dry	Poured out into the op		Visibly clea	Yes, alway	Liquid soap
1 2 3 4 8 1	15	Using own clothes	Left in the bucket		Visibly clea	Yes, some	Bar soap fo
2 3 4 5 11	39	Using own clothes	Left in the bucket		Visibly clea	Yes, some	Liquid soap
1 2 3 4 5 6	20	Air dried	Left in the bucket		Visibly clea	Yes, some	Bar soap, s
1 2 3 4 5 6	14	Air dried	Pours into the sink		Visibly clea	Yes, alway	Bar soap, s
1 2 3 4 5 6	14	Air dried	Poured out into the op		Visibly clea	Yes, alway	Bar soap, s
1 2 3 4 5 6	5	Using other piece of cl	Poured out in a draina		Visibly clea	Yes, some	Bar soap, c
1 2 4 5 11	90	Air dried	Left in the bucket		Visibly clea	Yes, some	Bathing soa
1 2 3 4 11	10	Did not dry	Poured out into the op		Visibly clea	Yes, some	Bar soap fo
4 5 7 9	5	Air dried	Other (Spe	Used sanit	Visibly clea	Yes, some	Bar soap, s
1 2 3 4 5 6	18	Air dried	Poured out into the op		Visibly clea	Yes, some	Bar soap, s
1 2 3 4 11	7	Did not dry	Pours into the sink		Visibly clea	Yes, alway	Bar soap, c
1 2 3 4 5 6	40	Air dried	Poured in toilet/bathro		Visibly clea	Yes, some	Bathing soa
1 2 5 10	15	Air dried	Left in the bucket		Visibly clea	Yes, alway	Liquid soap
1 2 3 4 9 1	2	Air dried	Left in the bucket		Visibly clea	Yes, alway	Bar soap, s
2 3 4 9 11	22	Using own clothes	Left in the bucket		Visibly clea	Yes, some	Liquid soap
1 2 3 4 5 6	14	Air dried	Poured out into the op		Visibly clea	Yes, alway	Bar soap, s
1 2 3 4 11	20	Did not dry	Poured out in a draina		Visibly clea	Yes, alway	Bar soap, c
1 2 3 4 9 1	20	Using other piece of cl	Left in the bucket		Visibly clea	Yes, some	Bar soap fo
1 2 3 5 6 8	12	Air dried	Poured out into the op		Visibly clea	Yes, alway	Bathing soa

1 2 3 4 5 6	30	Air dried	Left in the bucket	Visibly clea	Yes, some	Bar soap fo
1 2 3 5 8 1	5	Using own clothes	Pours into the sink	Visibly clea	Yes, alway	Bar soap, s
1 2 6 8 9 1	20	Did not dry	Left in the bucket	Visibly clea	Yes, alway	Bar soap, s
2 3 4 5 96	53	Did not dry	Drains into the open	Visibly clea	Yes, some	Liquid soap
2 3 4 5 6 1	53	Did not dry	Poured out into the op	Visibly clea	Yes, alway	Liquid soap
1 2 3 9 10	3	Using own clothes	Pours into the sink	Visibly clea	Yes, alway	Bar soap, s
1 2 3 5 6 1	300	Air dried	Poured out into the op	Visibly clea	Yes, some	Liquid soap
1 2 3 4 10	15	Air dried	Pours into the sink	Visibly clea	Yes, some	Bar soap, c
1 2 3 4 6 1	5	Using own clothes	Poured out into the op	Visibly clea	Yes, some	Bar soap, s
1 2 3 5 10	6	Did not dry	Left in the bucket	Visibly clea	Yes, alway	Bathing soa
2 4 5 11	37	Did not dry	Left in the bucket	Visibly clea	Yes, some	Liquid soap
1 2 3 4 6 9	45	Using other piece of cl	Left in the bucket	Visibly clea	Yes, some	Bathing soa
1 2 3 4	10	Did not dry	Left in the bucket	Visibly clea	Yes, some	Bathing soa
1 2 3 4 5 6	45	Using own clothes	Left in the bucket	Visibly clea	Yes, alway	Bar soap, c
1 2 3 4 5 6	32	Did not dry	Drains into the open	Visibly clea	Yes, alway	Bar soap, c
1 2 3 4 6 8	16	Air dried	Poured out in a draina	Visibly clea	Yes, some	Bar soap, s
1 2 3 4 11	10	Using own clothes	Left in the bucket	Visibly clea	Yes, some	Liquid soap
1 2 3 4 5 6	20	Air dried	Poured out into the op	Visibly clea	Yes, some	Bar soap, s
1 2 3 4 8 1	15	Air dried	Left in the bucket	Visibly clea	Yes, some	Bar soap fo
1 2 3 4	5	Did not dry	Drains into the open	Visibly clea	Yes, alway	Bar soap, c
1 2 3 4	5	Air dried	Poured out into the op	Visibly clea	Yes, some	Liquid soap
1 2 3 4 5 1	8	Did not dry	Left in the bucket	Visibly clea	Yes, some	Bathing soa
1 2 3 4 5 6	15	Air dried	Left in the bucket	Visibly clea	Yes, alway	Bathing soa
1 2 3 4 5 6	30	Air dried	Poured out into the op	Visibly clea	Yes, some	Bathing soa
1 2 3 4 5 1	10	Using own clothes	Poured out in a draina	Visibly clea	Yes, alway	Bar soap, s
1 2 3 4 5 7	12	Did not dry	Pours into the sink	Visibly clea	Yes, some	Bar soap, s
1 2 3 4 5 6	20	Air dried	Poured out into the op	Visibly clea	Yes, some	Bar soap fo
1 2 3 4 5 6	20	Air dried	Poured out into the op	Visibly clea	Yes, some	Liquid soap
1 2 4 5 6 8	30	Using other piece of cl	Left in the bucket	Visibly clea	Yes, some	Liquid soap
1 2 3 4 5 6	10	Air dried	Poured out into the op	Visibly clea	Yes, alway	Bar soap, s
1 2 3 4 5 7	20	Air dried	Poured out into the op	Visibly clea	Yes, some	Bar soap, s
1 2 4 5 6 1	95	Did not dry	Left in the bucket	Visibly clea	Yes, some	Liquid soap
1 2 3 4 5 6	8	Air dried	Poured out into the op	Visibly clea	Yes, alway	Bar soap, s
2 3 4 5 11	43	Using own clothes	Left in the bucket	Visibly clea	Yes, alway	Detergent/p
1 2 3 4 10	5	Did not dry	Poured out into the op	Visibly clea	Yes, alway	Bar soap, s
1 3 4 11	23	Did not dry	Left in the bucket	Visibly clea	Yes, some	Liquid soap
1 2 3 5 8 1	19	Air dried	Drains into the open	Visibly clea	Yes, alway	Bar soap, s
1 2 3 4 5 6	6	Air dried	Poured out in a draina	Visibly clea	Yes, alway	Bar soap, c
1 2 3 4 5 1	10	Did not dry	Left in the bucket	Visibly clea	Yes, alway	Bar soap fo
1 2 3 5 6 8	20	Air dried	Poured out into the op	Visibly clea	Yes, some	Bathing soa
2 5 6 8 10	15	Did not dry	Poured in toilet/bathro	Visibly clea	Yes, alway	Liquid soap
1 2 3 4 5 6	20	Using other piece of cl	Poured out into the op	Visibly clea	Yes, some	Bathing soa
1 2 3 5 10	12	Did not dry	Left in the bucket	Visibly clea	Yes, some	Bathing soa
1 2 3 4 5 6	30	Using own clothes	Pours into the sink	Visibly clea	Yes, some	Bar soap, s
1 2 3 4 5 7	20	Air dried	Poured out into the op	Visibly clea	Yes, some	Bathing soa
1 2 4 5 7 9	15	Air dried	Poured out into the op	Visibly clea	Yes, some	Bar soap, s
1 2 3 4 11	15	Using own clothes	Poured out into the op	Visibly clea	Yes, some	Bar soap, s
1 2 3 4 6 8	46	Air dried	Pours into the sink	Visibly clea	Yes, alway	Liquid soap
1 2 3 5 7 8	12	Using other piece of cl	Pours into the sink	Visibly clea	Yes, alway	Bar soap, s

1 2 3 4 5 6	4	Did not dry	Left in the bucket		Visibly clea	Yes, some	Bar soap, s
1 2 3 5 7 8	9	Did not dry	Poured out into the op		Visibly clea	Yes, alway	Bathing soa
2 3 4 6 11	54	Using own clothes	Left in the bucket		Visibly clea	Yes, alway	Liquid soap
1 2 4 6 8 9	10	Using own clothes	Poured out into the op		Visibly clea	Yes, some	Bar soap, s
1 2 4 6 7 8	20	Air dried	Poured out into the op		Visibly clea	Yes, some	Bar soap, s
1 2 3 4 5 6	15	Air dried	Poured out into the op		Visibly clea	Yes, alway	Bar soap, s
1 2 4 5 10	10	Did not dry	Drains into the open		Visibly clea	Yes, some	Bathing soa
1 2 3 4 6 9	13	Did not dry	Left in the bucket		Visibly clea	Yes, some	Bar soap fo
1 2 3 4 5 6	15	Air dried	Poured out into the op		Visibly clea	Yes, alway	Bar soap, s
1 2 3 4 5 6	30	Using other piece of cl	Poured out into the op		Visibly clea	Yes, some	Bar soap, s
1 2 3 4 5 6	15	Using other piece of cl	Poured out into the op		Visibly clea	Yes, some	Bathing soa
1 2 3 4 10	10	Did not dry	Left in the bucket		Visibly clea	Yes, some	Bar soap fo
1 2 3 4 5 6	15	Air dried	Poured out into the op		Visibly clea	Yes, alway	Liquid soap
1 2 3 4 5 1	20	Did not dry	Pours into the sink		Visibly clea	Yes, alway	Bathing soa
1 2 4 6 11	4	Using own clothes	Poured out into the op		Visibly clea	Yes, alway	Bar soap, s
1 2 3 4 11	6	Air dried	Poured out into the op		Visibly clea	Yes, some	Bar soap, s
1 2 3 6 9 1	4	Using own clothes	Poured out into the op		Visibly clea	Yes, alway	Bathing soa
1 2 3 6 7 1	6	Using own clothes	Poured out into the op		Visibly clea	Yes, some	Bathing soa
1 2 3 4	10	Did not dry	Poured out into the op		Visibly clea	Yes, alway	Bar soap fo
1 2 3 4 5 6	15	Did not dry	Left in the bucket		Visibly clea	Yes, some	Bar soap, c
1 2 3 4 11	5	Using other piece of cl	Poured out into the op		Visibly clea	Yes, some	Bathing soa
1 2 3 4 6 8	18	Air dried	Pours into the sink		Visibly clea	Yes, some	Bar soap, s
1 2 3 4 5 6	58	Using own clothes	Left in the bucket		Visibly clea	Yes, alway	Liquid soap
1 2 3 4 5 6	10	Did not dry	Left in the bucket		Visibly clea	Yes, alway	Bathing soa
1 2 3 4 11	120	Using other piece of cl	Poured out into the op		Visibly clea	Yes, some	Bathing soa
1 2 3 4 5 6	90	Using other piece of cl	Left in the bucket		Visibly clea	Yes, alway	Bar soap, s
1 2 3 4	5	Air dried	Poured out into the op		Visibly clea	Yes, some	Bar soap, c
1 2 3 4 5 8	12	Did not dry	Left in the bucket		Visibly clea	Yes, some	Bar soap, c
1 2 3 4 5 6	20	Air dried	Poured out into the op		Visibly clea	Yes, some	Bar soap, s
1 2 3 4 8 9	10	Using own clothes	Poured out into the op		Visibly clea	Yes, some	Liquid soap
1 2 3 4 7 8	2	Air dried	Left in the bucket		Visibly clea	Yes, some	Bar soap, c
1 2 3 4 11	5	Did not dry	Poured out into the op		Visibly clea	Yes, some	Bar soap, c
1 2 3 4 5 7	10	Air dried	Pours into the sink		Visibly clea	Yes, some	Bathing soa
1 2 3 4 11	30	Did not dry	Left in the bucket		Visibly clea	Yes, some	Bar soap fo
1 2 3 4 5 6	20	Air dried	Drains into the open		Visibly clea	Yes, some	Bathing soa
1 2 3 4 9 1	8	Using other piece of cl	Pours into the sink		Visibly clea	Yes, alway	Liquid soap
1 3 4 11	15	Did not dry	Other (Spe	She used v	Visibly clea	Yes, some	Bar soap fo
1 2 4 6 9 1	10	Did not dry	Left in the bucket		Visibly clea	Yes, alway	Bathing soa
1 2 3 4	6	Air dried	Poured out into the op		Visibly clea	Yes, some	Liquid soap
1 2 9 10 11	10	Did not dry	Left in the bucket		Visibly clea	Yes, alway	Bathing soa
1 2 3 4 5 1	5	Did not dry	Poured out in a draina		Visibly clea	Yes, alway	Bathing soa
1 2 3 4 5 6	55	Using other piece of cl	Pours into the sink		Visibly clea	Yes, alway	Liquid soap
1 2 3 4 5 6	20	Air dried	Poured out into the op		Visibly clea	Yes, some	Bar soap, s
1 2 4 6 10	10	Did not dry	Drains into the open		Visibly clea	Yes, some	Bathing soa
2 3 4 10	10	Did not dry	Drains into the open		Visibly clea	Yes, alway	Liquid soap
1 2 3 4 5 6	58	Did not dry	Poured out into the op		Visibly clea	Yes, alway	Bathing soa
1 2 3 4 10	5	Using own clothes	Drains into the open		Visibly clea	Yes, some	Bar soap, c
1 2 3 4 5 1	10	Did not dry	Poured out into the op		Visibly clea	Yes, some	Bar soap fo
1 2 3 4 10	10	Using other piece of cl	Poured out in a draina		Visibly clea	Yes, alway	Bar soap fo

1 2 3 4 5 6	12	Using other piece of cl	Poured out into the op	Visibly clea	Yes, alway	Bar soap, c
1 2 3 4 5 6	50	Using other piece of cl	Poured out into the op	Visibly clea	Yes, some	Bar soap, c
1 2 4 5 6 8	35	Using serviettes/tissue	Left in the bucket	Visibly clea	Yes, alway	Liquid soap
1 2 3 4 5 6	15	Using own clothes	Left in the bucket	Visibly clea	Yes, some	Bathing soa
2 3 4 5 11	45	Using other piece of cl	Left in the bucket	Visibly clea	Yes, some	Liquid soap
1 2 3 4 5 7	6	Did not dry	Left in the bucket	They appe	Yes, alway	Liquid soap
1 2 3 12	4	Using own clothes	Left in the bucket	Visibly clea	Yes, some	Bar soap, s
1 2 3 4 6 7	15	Air dried	Poured out into the op	They appe	Yes, some	Bathing soa
1 2 3 4 11	6	Using other piece of cl	Drains into the open	Visibly clea	Yes, some	Bar soap fo
1 2 8 12	10	Air dried	Left in the bucket	Visibly clea	Yes, alway	Bathing soa
1 2 3 4 5 6	10	Using own clothes	Left in the bucket	Visibly clea	Yes, some	Bar soap, s
1 2 3 4 5 9	15	Air dried	Poured out into the op	Visibly clea	Yes, some	Liquid soap
1 2 4 5 6 8	25	Using other piece of cl	Poured out into the op	Visibly clea	Yes, alway	Bar soap, c
1 2 4 10 11	10	Using other piece of cl	Left in the bucket	Visibly clea	Yes, some	Bathing soa
1 2 3 4 5 6	8	Air dried	Left in the bucket	Visibly clea	Yes, alway	Bathing soa
1 2 3 4 5 7	20	Air dried	Left in the bucket	Visibly clea	Yes, some	Bar soap, s
1 2 3 4 5 7	34	Did not dry	Poured out into the op	Visibly clea	Yes, alway	Detergent/p
1 2 3 4 5 9	10	Air dried	Poured out into the op	Visibly clea	Yes, alway	Bar soap, s
1 2 3 4	20	Did not dry	Left in the bucket	Visibly clea	Yes, some	Bar soap, s
1 2 3 4 11	10	Using other piece of cl	Poured out into the op	Visibly clea	Yes, some	Bar soap, s
1 2 4 5 6 7	241	Air dried	Poured in toilet/bathro	Visibly clea	Yes, some	Detergent/p
1 2 3 4 5 1	12	Did not dry	Poured out in a draina	Visibly clea	Yes, some	Bathing soa
1 2 3 4 5 6	20	Using own clothes	Left in the bucket	Visibly clea	Yes, some	Bar soap fo
1 2 6 8 10	20	Using other piece of cl	Drains into the open	Visibly clea	Yes, some	Bathing soa
1 2 3 4 5 6	9	Using other piece of cl	Poured out in a draina	Visibly clea	Yes, alway	Bar soap, c
1 2 3 10	15	Did not dry	Left in the bucket	Visibly clea	Yes, some	Bar soap, s
1 2 5 11	8	Did not dry	Left in the bucket	Visibly clea	Yes, some	Bathing soa
1 2 3 4 5 6	15	Did not dry	Left in the bucket	Visibly clea	Yes, alway	Bar soap fo
1 2 5 6 10	15	Using other piece of cl	Poured out in a draina	Visibly clea	Yes, alway	Bar soap, s
1 2 3 4 5 6	15	Air dried	Poured out into the op	Visibly clea	Yes, alway	Bar soap, s
1 2 3 4 5 6	10	Air dried	Poured in toilet/bathro	Visibly clea	Yes, alway	Detergent/p
1 2 4 5 11	4	Using own clothes	Poured out into the op	Visibly clea	Yes, some	Bar soap, s
1 2 3 4 5 8	10	Did not dry	Poured out into the op	Visibly clea	Yes, alway	Detergent/p
1 2 5 9 11	10	Using other piece of cl	Poured out into the op	Visibly clea	Yes, alway	Liquid soap
1 2 3 5 7 8	28	Using own clothes	Poured out into the op	Visibly clea	Yes, some	Liquid soap
1 2 3 4 6 7	56	Air dried	Pours into the sink	Visibly clea	Yes, alway	Liquid soap
1 4 8 11	10	Air dried	Poured out in a draina	Visibly clea	Yes, alway	Bathing soa
1 2 5 10	8	Did not dry	Left in the bucket	Visibly clea	Yes, alway	Bathing soa
1 2 3 4 6 8	3	Using other piece of cl	Left in the bucket	Visibly clea	Yes, alway	Bar soap fo
1 2 5 6 9 1	20	Using other piece of cl	Left in the bucket	Visibly clea	Yes, alway	Liquid soap
1 2 3 5 6 8	49	Did not dry	Poured out into the op	Visibly clea	Yes, alway	Bathing soa
1 2 3 4 5 6	20	Air dried	Poured out into the op	Visibly clea	Yes, some	Bar soap, s
1 2 3 11 12	5	Using own clothes	Poured out into the op	Visibly clea	Yes, some	Bar soap, c
1 2 3 4 5 6	12	Air dried	Poured out into the op	Visibly clea	Yes, alway	Liquid soap
1 2 3 4 11	5	Did not dry	Poured out into the op	Visibly clea	Yes, alway	Bar soap, c
1 2 3 5 6 7	45	Air dried	Poured out into the op	Visibly clea	Yes, alway	Liquid soap
1 2 5 6 7 9	20	Air dried	Left in the bucket	Visibly clea	Yes, alway	Bar soap, s
1 2 3 4 5 6	10	Air dried	Poured out into the op	Visibly clea	Yes, alway	Bar soap, s
1 2 3 4 5 6	40	Air dried	Drains into the open	Visibly clea	Yes, some	Bar soap fo

1 2 3 4 5 9	6	Air dried		Left in the bucket		They appe	Yes, some	Bar soap, s
1 2 3 8 10	5	Air dried		Drains into the open		They appe	Yes, some	Detergent/p
96	0	Other (Spe	Did not wa	Other (Spe	NA	Visibly clea	Yes, alway	Bathing soa
1 2 3 4 11	20	Did not dry		Poured in toilet/bathro		Visibly clea	Yes, some	Bar soap fo
1 2 3 4 5 6	10	Using other piece of cl		Poured out into the op		Visibly clea	Yes, some	Bathing soa
1 2 3 4 5 6	15	Air dried		Poured out into the op		Visibly clea	Yes, some	Bar soap, s
1 2 3 4 5 1	100	Using own clothes		Left in the bucket		Visibly clea	Yes, some	Liquid soap
1 2 3 4 6 1	20	Did not dry		Poured out in a draina		Visibly clea	Yes, alway	Liquid soap
1 2 3 4 5 6	30	Using own clothes		Drains into the open		Visibly clea	Yes, some	Bathing soa
1 2 3 4 5 6	15	Air dried		Poured out into the op		Visibly clea	Yes, some	Bar soap, s
1 2 3 4 11	6	Using other piece of cl		Left in the bucket		Visibly clea	Yes, alway	Bathing soa
1 2 3 5 8 1	15	Air dried		Poured out into the op		Visibly clea	Yes, alway	Bar soap, s
1 2 3 4 5 6	4	Air dried		Left in the bucket		Visibly clea	Yes, alway	Bar soap, c
1 2 5 10	8	Did not dry		Poured out in a draina		Visibly clea	Yes, alway	Bathing soa
1 2 3 4 5 6	15	Air dried		Pours into the sink		Visibly clea	Yes, alway	Bar soap, s
1 2 3 4 5 8	30	Using other piece of cl		Poured out in a draina		Visibly clea	Yes, some	Bathing soa
1 2 3 8	8	Did not dry		Poured out into the op		Visibly clea	Yes, alway	Bathing soa
1 2 5 8 9 1	20	Air dried		Left in the bucket		Visibly clea	Yes, some	Bar soap, s
1 4 9 10 12	20	Using own clothes		Poured out into the op		Visibly clea	Yes, some	Detergent/p
1 2 3 5 6 1	15	Air dried		Poured out into the op		Visibly clea	Yes, alway	Liquid soap
1 3 5 7 8 1	20	Air dried		Poured out into the op		Visibly clea	Yes, some	Bar soap, s
1 2 3 4 5 6	180	Using other piece of cl		Poured out in a draina		Visibly clea	Yes, alway	Bar soap, c
1 2 3 4 5 1	100	Did not dry		Left in the bucket		Visibly clea	Yes, some	Detergent/p
1 4 7 9 11	20	Using other piece of cl		Poured out in a draina		Visibly clea	Yes, alway	Bathing soa
96	0	Did not dry		Drains into the open		Visibly clea	Yes, alway	Bathing soa
1 2 3 5 6 7	6	Air dried		Poured out into the op		Visibly clea	Yes, alway	Bar soap, s
1 2 4	5	Air dried		Poured out into the op		Visibly clea	Yes, some	Liquid soap
2 3 4 5 11	36	Did not dry		Left in the bucket		Visibly clea	Yes, alway	Liquid soap
1 2 3 4 5 1	10	Did not dry		Left in the bucket		Visibly clea	Yes, alway	Bathing soa
1 2 3 4 11	20	Did not dry		Left in the bucket		Visibly clea	Yes, some	Bar soap fo
1 2 3 4 8 1	8	Air dried		Poured out into the op		Visibly clea	Yes, alway	Bar soap, s
1 2 5 10	7	Did not dry		Left in the bucket		Visibly clea	Yes, some	Liquid soap
96	0	Other (Spe	did not den	Other (Spe	did not der	Visibly clea	Yes, alway	Bar soap, c
1 2 4 5 6 7	268	Did not dry		Left in the bucket		They appe	Yes, some	Bar soap, c
1 2 3 5 7 9	20	Air dried		Poured out into the op		Visibly clea	Yes, some	Liquid soap
1 2 3 4 7 1	20	Using own clothes		Poured out in a draina		Visibly clea	Yes, alway	Bathing soa
1 2 3 4 5 8	10	Did not dry		Other (Spe	she did not	Visibly clea	Yes, alway	Liquid soap
1 2 3 4 5 6	10	Using own clothes		Left in the bucket		Visibly clea	Yes, some	Bar soap, s
1 2 4 6 7 1	20	Air dried		Left in the bucket		Visibly clea	Yes, some	Bar soap, s
1 2 3 4 9	12	Did not dry		Left in the bucket		Visibly clea	Yes, alway	Bar soap fo
1 2 4 5 10	3	Using other piece of cl		Pours into the sink		Visibly clea	Yes, alway	Liquid soap
1 2 3 4	10	Did not dry		Left in the bucket		Visibly clea	Yes, some	Bar soap fo
1 2 3 7 8 9	190	Did not dry		Left in the bucket		Visibly clea	Yes, some	Liquid soap
1 2 3 4 5 6	20	Using other piece of cl		Poured out in a draina		Visibly clea	Yes, some	Bathing soa
1 2 3 4 5 6	2	Did not dry		Left in the bucket		Visibly clea	Yes, some	Bar soap, s
2 3 4 5 11	20	Did not dry		Left in the bucket		Visibly clea	Yes, some	Detergent/p
1 2 3 4 9 1	70	Air dried		Poured out into the op		Visibly clea	Yes, alway	Bar soap, c
1 2 3 4 5 6	8	Air dried		Poured out into the op		Visibly clea	Yes, alway	Bar soap, s
2 3 4 5 11	20	Did not dry		Left in the bucket		Visibly clea	Yes, some	Liquid soap

1 2 3 4 5 6	120	Using other piece of cl	Poured out in a draina	Visibly clea	Yes, some	Bar soap fo	
1 2 4 6 8 1	20	Using other piece of cl	Poured out in a draina	Visibly clea	Yes, alway	Bar soap, c	
1 2 3 4 5	10	Did not dry	Drains into the open	Visibly clea	Yes, alway	Liquid soap	
1 2 3 4 5 6	2	Using own clothes	Left in the bucket	Visibly clea	Yes, alway	Bar soap, s	
1 2 4 5 7 9	20	Air dried	Poured out into the op	Visibly clea	Yes, some	Bar soap, s	
1 2 3 4 5 6	18	Air dried	Poured out into the op	Visibly clea	Yes, some	Bar soap, s	
1 2 3 4 5 1	10	Using other piece of cl	Left in the bucket	Visibly clea	Yes, some	Liquid soap	
1 2 3 5 6 8	20	Air dried	Poured out into the op	Visibly clea	Yes, some	Bar soap, s	
1 2 3 4 5 1	5	Using own clothes	Left in the bucket	Visibly clea	Yes, some	Bar soap, s	
1 2 4 5 8 1	15	Did not dry	Poured out in a draina	Visibly clea	Yes, alway	Bar soap, s	
1 2 3 4 5 6	20	Air dried	Poured out into the op	Visibly clea	Yes, some	Bar soap, s	
1 2 3 4 6 8	20	Did not dry	Poured out in a draina	Visibly clea	Yes, alway	Bathing soa	
1 2 4 5 6 8	46	Air dried	Poured out into the op	Visibly clea	Yes, alway	Liquid soap	
1 2 3 4 10	7	Did not dry	Drains into the open	Visibly clea	Yes, some	Bathing soa	
4 5 96	5	Other (Spe	did not den	Other (Spe	did not der	Visibly clea	Yes, some	Bar soap fo
2 3 4 5 7 8	46	Did not dry	Poured out into the op	Visibly clea	Yes, alway	Liquid soap	
1 2 4 12	3	Using own clothes	Poured out into the op	Visibly clea	Yes, some	Bar soap, s	
1 2 5 6 9 1	20	Air dried	Poured out into the op	Visibly clea	Yes, some	Bar soap, s	
1 2 3 4 5 6	5	Using other piece of cl	Left in the bucket	Visibly clea	Yes, alway	Liquid soap	
1 2 3 4 5 6	64	Using own clothes	Left in the bucket	Visibly clea	Yes, alway	Liquid soap	
1 2 3 4 5 6	6	Using other piece of cl	Left in the bucket	Visibly clea	Yes, some	Bathing soa	
1 2 3 4 5 6	20	Air dried	Poured out in a draina	Visibly clea	Yes, some	Bar soap, s	
1 2 3 4 11	7	Air dried	Drains into the open	Visibly clea	Yes, alway	Bar soap, c	
1 2 3 4 10	5	Did not dry	Left in the bucket	Visibly clea	Yes, alway	Bar soap fo	
1 2 3 4 5 6	90	Air dried	Poured out in a draina	Visibly clea	Yes, some	Bathing soa	
1 2 3 4 5 1	10	Using other piece of cl	Poured out into the op	Visibly clea	Yes, some	Bar soap, c	
1 2 3 4 5 6	20	Air dried	Drains into the open	Visibly clea	Yes, alway	Bar soap, s	
1 2 3 4 5 6	120	Did not dry	Poured out into the op	Visibly clea	Yes, alway	Liquid soap	
1 2 3 4 5 8	10	Did not dry	Left in the bucket	Visibly clea	Yes, alway	Bar soap, c	
1 2 3 4 6 8	15	Did not dry	Pours into the sink	Visibly clea	Yes, alway	Bar soap, c	
1 2 3 4 5 8	3	Using own clothes	Left in the bucket	Visibly clea	Yes, alway	Bar soap, c	
2 3 4 11	30	Did not dry	Left in the bucket	Visibly clea	Yes, some	Liquid soap	
1 2 3 5 9 1	4	Did not dry	Poured out into the op	Visibly clea	Yes, alway	Bar soap, c	
1 3 4	5	Air dried	Poured out into the op	Visibly clea	Yes, some	Bar soap, c	
1 2 5 10	12	Did not dry	Left in the bucket	Visibly clea	Yes, some	Bathing soa	
1 2 5 8 11	4	Using own clothes	Poured out into the op	Visibly clea	Yes, alway	Bar soap, s	
1 2 3 4 5 6	20	Air dried	Poured out into the op	Visibly clea	Yes, some	Bar soap, s	
1 2 3 4 11	5	Using other piece of cl	Drains into the open	Visibly clea	Yes, some	Liquid soap	
1 2 3 6 7 8	10	Using other piece of cl	Poured out into the op	Visibly clea	Yes, alway	Bar soap fo	
1 2 3 4 5 6	30	Air dried	Poured out into the op	Visibly clea	Yes, some	Bar soap fo	
1 2 5 9 11	20	Using own clothes	Poured out in a draina	Visibly clea	Yes, alway	Bathing soa	
1 2 3 4 5 6	25	Did not dry	Left in the bucket	Visibly clea	Yes, alway	Liquid soap	
1 2 3 4 5 8	30	Did not dry	Poured out into the op	Visibly clea	Yes, alway	Detergent/p	
1 2 3 4 5 7	56	Air dried	Poured out in a draina	Visibly clea	Yes, alway	Liquid soap	
1 2 4 5 11	1	Air dried	Left in the bucket	Visibly clea	Yes, alway	Bathing soa	
1 2 3 4 5 8	3	Using other piece of cl	Left in the bucket	Visibly clea	Yes, alway	Bar soap, c	
1 2 3	10	Air dried	Poured out in a draina	Visibly clea	Yes, some	Bar soap, c	
1 2 3 4 11	30	Using other piece of cl	Left in the bucket	Visibly clea	Yes, some	Bathing soa	
1 3 4 7 8 1	20	Air dried	Poured out into the op	Visibly clea	Yes, some	Bar soap, s	

1 2 3 4 5 6	14	Using other piece of cl	Poured out into the op	Visibly clea	Yes, alway	Bar soap, s
2 3 4 11	46	Did not dry	Poured out into the op	Visibly clea	Yes, some	Liquid soap
1 2 3 4 5 6	15	Using other piece of cl	Left in the bucket	They appe	Yes, alway	Bar soap, s
1 2 3 4 5 6	15	Using own clothes	Left in the bucket	Visibly clea	Yes, alway	Bathing soa
1 2 5 11 12	60	Using other piece of cl	Poured out in a draina	Visibly clea	Yes, some	Liquid soap
1 2 3 4 6 7	10	Air dried	Poured out into the op	Visibly clea	Yes, some	Bar soap, s
1 2 3 4 8 1	10	Using other piece of cl	Poured out in a draina	Visibly clea	Yes, alway	Bathing soa
1 2 3 4 6 8	1	Did not dry	Left in the bucket	Visibly clea	Yes, alway	Bar soap, s
1 3 5 8 11	20	Air dried	Poured out into the op	Visibly clea	Yes, some	Bar soap, s
1 2 3 4 5 6	20	Did not dry	Left in the bucket	Visibly clea	Yes, some	Bathing soa
1 2 3 4 10	4	Air dried	Left in the bucket	They appe	Yes, some	Liquid soap
1 2 3 4 5 8	79	Did not dry	Poured out into the op	Visibly clea	Yes, some	Liquid soap
1 2 3 4 5 6	12	Did not dry	Poured out into the op	Visibly clea	Yes, alway	Bar soap, s
1 2 3 4 5 7	14	Air dried	Poured out into the op	Visibly clea	Yes, alway	Bar soap, s
1 3 5 8 11	20	Air dried	Poured out into the op	Visibly clea	Yes, some	Bar soap, s
1 2 3 4 5 6	15	Air dried	Poured out into the op	Visibly clea	Yes, alway	Bar soap, s
1 2 3	5	Air dried	Poured out into the op	Visibly clea	Yes, some	Liquid soap
1 2 3 4 5 6	9	Using other piece of cl	Poured out in a draina	Visibly clea	Yes, alway	Bathing soa
1 2 9 11 12	3	Using own clothes	Poured out into the op	They appe	Yes, some	Bar soap, s
1 2 3 4 5 6	30	Using own clothes	Poured out into the op	Visibly clea	Yes, some	Bar soap fo
1 2 3 4 5 6	15	Did not dry	Poured out into the op	Visibly clea	Yes, some	Bar soap, s
1 2 3 4 5 6	10	Did not dry	Drains into the open	Visibly clea	Yes, alway	Bar soap, c
1 2 3 4 5 6	150	Did not dry	Left in the bucket	Visibly clea	Yes, some	Liquid soap
1 2 3 4 5 6	61	Air dried	Pours into the sink	Visibly clea	Yes, alway	Liquid soap
1 2 3 4 5 1	52	Air dried	Poured out into the op	Visibly clea	Yes, alway	Liquid soap
1 2 4 6 9 1	20	Using own clothes	Poured out in a draina	Visibly clea	Yes, alway	Liquid soap
1 3 4 5	10	Did not dry	Left in the bucket	Visibly clea	Yes, alway	Bathing soa
1 3 5 7 9 1	20	Air dried	Poured out into the op	Visibly clea	Yes, some	Bathing soa
1 2 3 4 11	20	Did not dry	Left in the bucket	Visibly clea	Yes, some	Bar soap, s
1 2 3 4 5 6	15	Air dried	Poured out into the op	Visibly clea	Yes, alway	Bar soap, s
1 2 4 5 7 8	30	Air dried	Poured out in a draina	Visibly clea	Yes, alway	Bathing soa
1 2 3 4 5 6	10	Air dried	Poured out into the op	Visibly clea	Yes, alway	Bar soap, s
1 2 3 4 10	10	Did not dry	Left in the bucket	Visibly clea	Yes, some	Liquid soap
1 2 3 4 5 1	8	Using other piece of cl	Poured out in a draina	Visibly clea	Yes, alway	Bar soap fo
1 2 4 9 10	5	Using own clothes	Left in the bucket	Visibly clea	Yes, alway	Liquid soap
1 2 3 4 5 1	5	Using other piece of cl	Pours into the sink	Visibly clea	Yes, alway	Liquid soap
1 2 3 4 5 6	8	Using other piece of cl	Poured out into the op	Visibly clea	Yes, alway	Bar soap, s
1 2 3 4 5 6	15	Did not dry	Poured out in a draina	Visibly clea	Yes, alway	Liquid soap
1 2 4 5 6 8	100	Using own clothes	Left in the bucket	Visibly clea	Yes, some	Bar soap fo
1 2 11	2	Did not dry	Poured in toilet/bathro	Visibly clea	Yes, alway	Bathing soa
1 2 5 10	6	Did not dry	Drains into the open	Visibly clea	Yes, some	Bar soap, c
1 2 3 4 5 6	8	Air dried	Poured out into the op	Visibly clea	Yes, alway	Bar soap, s
1 2 4 6 7 9	20	Air dried	Poured out into the op	Visibly clea	Yes, some	Bathing soa
1 2 3 4 9 1	5	Did not dry	Left in the bucket	Visibly clea	Yes, alway	Bathing soa
1 2 3 7 11	5	Using own clothes	Poured in toilet/bathro	Visibly clea	Yes, alway	Bathing soa
1 2 3 4 5 8	4	Using other piece of cl	Left in the bucket	Visibly clea	Yes, alway	Liquid soap
1 2 3 4 5 6	60	Using serviettes/tissue	Left in the bucket	Visibly clea	Yes, some	Bathing soa
1 2 3 4 5 7	60	Did not dry	Pours into the sink	Visibly clea	Yes, alway	Bar soap, c
1 2 4 10 12	10	Did not dry	Left in the bucket	Visibly clea	Yes, some	Bar soap, s

1 2 3 5 7 1	10	Air dried		Drains into the open		Visibly clea	Yes, alway	Bar soap, s
1 2 4 5 96	8	Other (Spe	demonstra	Other (Spe	did not der	Visibly clea	Yes, alway	Bar soap, c
1 2 3 5 6 8	20	Air dried		Poured out into the op		Visibly clea	Yes, some	Liquid soap
1 2 3 4 5 8	4	Using other piece of cl		Poured out into the op		Visibly clea	Yes, alway	Bathing soa
1 2 3 4 5 6	20	Air dried		Left in the bucket		Visibly clea	Yes, some	Bathing soa
2 3 6 7 9 1	33	Did not dry		Poured out into the op		Visibly clea	Yes, alway	Liquid soap
2 3 4 5 6 7	12	Using other piece of cl		Left in the bucket		Visibly clea	Yes, alway	Liquid soap
2 3 4 5 11	47	Did not dry		Left in the bucket		Visibly clea	Yes, alway	Liquid soap
1 5 9 11 12	15	Using other piece of cl		Left in the bucket		Visibly clea	Yes, alway	Bathing soa
1 2 3 4 5 1	10	Air dried		Left in the bucket		Visibly clea	Yes, some	Bar soap, c
1 2 4 11 12	10	Using own clothes		Poured out in a draina		Visibly clea	Yes, some	Bathing soa
1 2 3 4 5 6	10	Air dried		Left in the bucket		Visibly clea	Yes, some	Bar soap, c
1 2 3 4 5 6	5	Using own clothes		Left in the bucket		Visibly clea	Yes, alway	Bar soap, c
1 2 3	5	Air dried		Poured out into the op		Visibly clea	Yes, some	Detergent/p
1 2 3 6 11	8	Air dried		Left in the bucket		Visibly clea	Yes, alway	Bar soap, c
1 2 3 4 5 7	17	Did not dry		Drains into the open		Visibly clea	Yes, some	Bathing soa
1 2 3 4 5 6	20	Air dried		Poured out into the op		Visibly clea	Yes, some	Bar soap, s
1 2 4 5 6 7	30	Using other piece of cl		Left in the bucket		Visibly clea	Yes, alway	Liquid soap
1 2 3 4 5 6	20	Did not dry		Left in the bucket		Visibly clea	Yes, alway	Bathing soa
1 2 3 4 5 6	20	Air dried		Poured out into the op		Visibly clea	Yes, some	Bathing soa
1 2 4 5 10	6	Using own clothes		Pours into the sink		Visibly clea	Yes, alway	Bar soap, c
1 2 3 4	10	Did not dry		Left in the bucket		Visibly clea	Yes, alway	Liquid soap
1 2 3 6 7 9	35	Did not dry		Poured in toilet/bathro		Visibly clea	Yes, some	Liquid soap
1 2 5 7 8 9	15	Using other piece of cl		Left in the bucket		Visibly clea	Yes, alway	Bathing soa
2 3 4 5 6 7	6	Air dried		Poured out into the op		Visibly clea	Yes, alway	Bar soap, s
1 2 3 4 5 6	10	Air dried		Poured out into the op		Visibly clea	Yes, alway	Bar soap, s
1 2 3 4 5 6	10	Did not dry		Left in the bucket		Visibly clea	Yes, alway	Bar soap, c
1 2 3 4 5 6	150	Air dried		Left in the bucket		Visibly clea	Yes, some	Liquid soap
1 2 3 4 5 6	8	Air dried		Left in the bucket		They appe	Yes, alway	Bar soap, s
1 2 3 4 5 6	33	Using own clothes		Left in the bucket		Visibly clea	Yes, alway	Detergent/p
1 2 3 4 5 6	2	Air dried		Left in the bucket		Visibly clea	Yes, alway	Bathing soa
1 2 3 4 5 6	21	Did not dry		Pours into the sink		Visibly clea	Yes, alway	Detergent/p
1 2 3 5 6 1	9	Using other piece of cl		Left in the bucket		Visibly clea	Yes, some	Liquid soap
1 2 3 4 5 6	15	Did not dry		Pours into the sink		Visibly clea	Yes, alway	Bar soap, c
2 3 4 96	40	Did not dry		Drains into the open		Visibly clea	Yes, alway	Detergent/p
1 2 3 6 10	10	Air dried		Left in the bucket		Visibly clea	Yes, some	Liquid soap
1 2 3 11	10	Air dried		Poured out into the op		They appe	Yes, some	Bar soap, s
1 2 12	15	Using other piece of cl		Left in the bucket		Visibly clea	Yes, alway	Liquid soap
1 2 3 5 10	15	Using own clothes		Poured out into the op		Visibly clea	Yes, alway	Bathing soa
1 2 3 4 5 6	30	Air dried		Poured in toilet/bathro		Visibly clea	Yes, some	Bathing soa
1 2 3	5	Air dried		Poured out into the op		Visibly clea	Yes, some	Liquid soap
1 2 3 4 6 8	15	Air dried		Poured out into the op		Visibly clea	Yes, alway	Bar soap, s
2 3 4 5 11	47	Did not dry		Left in the bucket		Visibly clea	Yes, alway	Liquid soap
1 2 3 4 5 6	10	Air dried		Poured out into the op		Visibly clea	Yes, alway	Bar soap, s
2 3 4 5 10	1	Air dried		Drains into the open		Visibly clea	Yes, alway	Liquid soap
1 2 3 4 5 7	20	Air dried		Poured out into the op		Visibly clea	Yes, some	Bar soap, s
1 2 3 4 5 6	12	Did not dry		Pours into the sink		Visibly clea	Yes, alway	Liquid soap
1 2 3 6 7 9	20	Air dried		Poured out into the op		Visibly clea	Yes, some	Bar soap, s
1 2 3 4 6 7	2	Using other piece of cl		Left in the bucket		Visibly clea	Yes, alway	Bathing soa

1 2 3 4 6 9	30	Using other piece of cl		Left in the bucket		Visibly clea	Yes, some	Bar soap, c
1 2 3 4 7 8	33	Did not dry		Left in the bucket		Visibly clea	Yes, alway	Bar soap, s
1 2 3 4 5 6	10	Air dried		Drains into the open		Visibly clea	Yes, some	Liquid soap
1 2 3 5 6 7	57	Air dried		Poured out into the op		Visibly clea	Yes, alway	Liquid soap
1 2 5 6 8 1	40	Did not dry		Poured out into the op		Visibly clea	Yes, alway	Liquid soap
1 2 3 4 6 7	3	Using other piece of cl		Left in the bucket		Visibly clea	Yes, alway	Liquid soap
1 2 3 7 9 1	8	Using own clothes		Left in the bucket		Visibly clea	Yes, alway	Bar soap, c
1 2 3 10	30	Did not dry		Poured out into the op		Visibly clea	Yes, alway	Liquid soap
1 2 3 5 6 1	12	Did not dry		Left in the bucket		Visibly clea	Yes, some	Bar soap, s
1 2 3 4 5 9	10	Did not dry		Left in the bucket		Visibly clea	Yes, some	Bathing soa
1 2 3 4 11	25	Using other piece of cl		Poured out in a draina		Visibly clea	Yes, some	Bar soap fo
1 2 3 5 6 7	8	Air dried		Poured out into the op		Visibly clea	Yes, alway	Bar soap, s
96	0	Other (Spe	Did not wa	Other (Spe	Did not wa	Visibly clea	Yes, alway	Liquid soap
1 2 4 5 6 8	35	Using other piece of cl		Poured out in a draina		Visibly clea	Yes, alway	Bathing soa
1 2 3 4 5 8	5	Using own clothes		Poured out into the op		Visibly clea	Yes, alway	Bar soap fo
1 2 3 5 6 7	20	Air dried		Poured out into the op		Visibly clea	Yes, some	Bar soap, s
2 3 4 5 11	33	Using own clothes		Left in the bucket		Visibly clea	Yes, alway	Detergent/p
1 3 5 7 8 1	20	Air dried		Poured out into the op		Visibly clea	Yes, some	Bar soap, s
1 2 3 4 5 7	67	Air dried		Poured out into the op		Visibly clea	Yes, alway	Bathing soa
1 2 4 5 9 1	20	Using other piece of cl		Poured out in a draina		Visibly clea	Yes, some	Bathing soa
96	0	Other (Spe	Did not wa	Other (Spe	Did not wa	Visibly clea	Yes, alway	Bar soap, c
1 2 5 10	2	Using other piece of cl		Poured out into the op		Visibly clea	Yes, some	Liquid soap
1 2 3 4 5 6	10	Using other piece of cl		Drains into the open		Visibly clea	Yes, alway	Liquid soap
1 2 3 4 5 7	300	Air dried		Left in the bucket		Visibly clea	Yes, alway	Detergent/p
1 2 3 4 6	45	Did not dry		Poured out into the op		Visibly clea	Yes, some	Bar soap, s
1 2 3 4 5 6	77	Did not dry		Drains into the open		Visibly clea	Yes, some	Bathing soa
1 2 3 4 6 1	2	Using own clothes		Left in the bucket		Visibly clea	Yes, alway	Bar soap, s
1 2 3 4 11	5	Did not dry		Drains into the open		Visibly clea	Yes, some	Bar soap fo
1 2 4 5 6 7	15	Air dried		Poured out into the op		Visibly clea	Yes, some	Bar soap, s
1 2 3 4 6 1	3	Using own clothes		Poured out into the op		Visibly clea	Yes, alway	Bathing soa
1 2 3 4 5 6	20	Air dried		Poured out into the op		Visibly clea	Yes, some	Bar soap, s
1 5 11 12	10	Using own clothes		Poured out into the op		Visibly clea	Yes, some	Bar soap, s
2 3 4 5 8 1	45	Using own clothes		Poured out in a draina		Visibly clea	Yes, alway	Detergent/p
1 2 3 4 6 9	20	Air dried		Poured out into the op		Visibly clea	Yes, some	Bar soap, s
1 2 4 5 8 9	15	Air dried		Poured out in a draina		Visibly clea	Yes, alway	Bar soap, s
1 2 3 4 5 6	14	Air dried		Poured out into the op		Visibly clea	Yes, alway	Bar soap, s
1 2 3	5	Air dried		Left in the bucket		Visibly clea	Yes, some	Liquid soap
1 2 3 4 5 6	20	Using other piece of cl		Poured out into the op		Visibly clea	Yes, some	Bathing soa
1 2 4 11 13	10	Air dried		Left in the bucket		Visibly clea	Yes, some	Bar soap fo
1 2 3 4 5 6	20	Air dried		Poured out into the op		Visibly clea	Yes, some	Bathing soa
1 2 3 4 5 6	10	Air dried		Poured out into the op		Visibly clea	Yes, alway	Bar soap, s
2 3 8 9 10	50	Air dried		Poured in toilet/bathro		Visibly clea	Yes, alway	Detergent/p
1 2 3 4 5 6	10	Air dried		Poured out into the op		Visibly clea	Yes, alway	Bar soap, s
1 4 11	5	Did not dry		Poured out into the op		Visibly clea	Yes, alway	Bar soap fo
1 2 3 4 5 8	2	Did not dry		Left in the bucket		Visibly clea	Yes, alway	Bathing soa
1 2 3 6 7 8	46	Using own clothes		Poured in toilet/bathro		Visibly clea	Yes, alway	Bar soap, s
1 2 3 4 5 1	47	Did not dry		Left in the bucket		Visibly clea	Yes, some	Liquid soap
1 2 3 4 5 1	5	Did not dry		Poured out into the op		Visibly clea	Yes, some	Bar soap, c
1 2 3 4 5 6	120	Using other piece of cl		Left in the bucket		Visibly clea	Yes, alway	Bar soap, c

1 2 3 4 10	5	Using own clothes		Poured out in a draina		Visibly clea	Yes, some	Bar soap, c
1 2 4 5 6 7	20	Air dried		Poured out into the op		Visibly clea	Yes, alway	Bathing soa
1 2 3 4 5 6	53	Did not dry		Drains into the open		Visibly clea	Yes, alway	Liquid soap
1 2 3 4 5 6	10	Using own clothes		Poured out in a draina		Visibly clea	Yes, alway	Bar soap fo
1 2 5 8 10	30	Using own clothes		Poured out in a draina		Visibly clea	Yes, some	Bathing soa
1 2 3 5 96	10	Other (Spe	did not der	Other (Spe	did not der	Visibly clea	Yes, alway	Bar soap, c
2 3 4 5 11	31	Did not dry		Left in the bucket		Visibly clea	Yes, some	Detergent/p
1 2 3 4 5 6	15	Using other piece of cl		Left in the bucket		Visibly clea	Yes, alway	Bar soap, c
1 2 3 5 6 7	41	Using other piece of cl		Left in the bucket		Visibly clea	Yes, alway	Bar soap, s
1 2 3 4 9 1	2	Using own clothes		Left in the bucket		Visibly clea	Yes, alway	Bar soap fo
1 2 4 6 7 8	25	Did not dry		Poured out into the op		Visibly clea	Yes, some	Liquid soap
1 2 3 4 5 7	15	Air dried		Poured out into the op		Visibly clea	Yes, some	Bar soap, s
1 2 3 4 5 6	8	Air dried		Poured out into the op		Visibly clea	Yes, alway	Bar soap, c
1 2 3 4 5 6	20	Using own clothes		Poured out in a draina		Visibly clea	Yes, some	Bar soap, c
1 2 3 4 5 7	8	Air dried		Poured out into the op		Visibly clea	Yes, alway	Bar soap, s
1 2 3 11 12	3	Using own clothes		Poured out into the op		Visibly clea	Yes, alway	Bar soap, s
2 4 5 11	40	Did not dry		Left in the bucket		Visibly clea	Yes, some	Liquid soap
1 2 3 4 5 6	20	Air dried		Left in the bucket		Visibly clea	Yes, some	Bathing soa
5 9 11 96	10	Did not dry		Poured out into the op		Visibly clea	Yes, alway	Liquid soap
1 2 3 4 5 1	15	Using own clothes		Left in the bucket		Visibly clea	Yes, alway	Bathing soa
1 2 4 5 6 8	25	Air dried		Left in the bucket		Visibly clea	Yes, alway	Bar soap, s
1 2 3 4 96	42	Did not dry		Drains into the open		Visibly clea	Yes, some	Liquid soap
1 2 3 4 6 7	15	Air dried		Poured out into the op		Visibly clea	Yes, some	Bar soap, s
1 2 3 4 5 1	25	Using own clothes		Left in the bucket		Visibly clea	Yes, some	Bathing soa
2 3 4 5 11	40	Did not dry		Left in the bucket		Visibly clea	Yes, some	Liquid soap
1 2 3 4 5 6	20	Air dried		Poured out into the op		Visibly clea	Yes, some	Bar soap, s
1 2 3 4 5 6	12	Air dried		Poured out into the op		Visibly clea	Yes, alway	Bar soap, s
1 2 3 4 5 8	20	Air dried		Poured out into the op		Visibly clea	Yes, some	Bar soap, s
1 2 3 4 5 6	8	Air dried		Left in the bucket		Visibly clea	Yes, alway	Bathing soa
1 2 3 5 10	13	Air dried		Pours into the sink		Visibly clea	Yes, some	Bathing soa
1 2 3 4 6 7	43	Air dried		Pours into the sink		Visibly clea	Yes, alway	Liquid soap
1 2 3 5 6 1	4	Using own clothes		Poured out into the op		Visibly clea	Yes, some	Bathing soa
1 2 4 8 11	20	Using own clothes		Poured out in a draina		Visibly clea	Yes, alway	Bathing soa
1 2 4 5 7 1	15	Using own clothes		Left in the bucket		Visibly clea	Yes, alway	Bar soap, s
1 2 4 7 9 1	20	Air dried		Poured out into the op		Visibly clea	Yes, some	Bar soap, s
1 2 3 4 5 8	2	Did not dry		Poured out in a draina		Visibly clea	Yes, alway	Bathing soa
1 3 4 5 10	10	Air dried		Drains into the open		Visibly clea	Yes, some	Bar soap, c
1 2 3 4 7 8	10	Using own clothes		Poured out into the op		Visibly clea	Yes, alway	Bar soap, s
1 2 4 5 8 9	20	Using other piece of cl		Poured out into the op		Visibly clea	Yes, some	Bathing soa
1 2 3 4 5 6	30	Air dried		Poured out into the op		Visibly clea	Yes, some	Bathing soa
1 2 3 4 6 9	120	Using other piece of cl		Poured out into the op		Visibly clea	Yes, some	Liquid soap
1 2 3 6 8 1	5	Did not dry		Left in the bucket		Visibly clea	Yes, some	Liquid soap
1 2 5 10	5	Did not dry		Poured out in a draina		Visibly clea	Yes, alway	Liquid soap
1 2 3 4 11	2	Using own clothes		Poured out into the op		Visibly clea	Yes, some	Bar soap, s
1 3 4 11	5	Did not dry		Left in the bucket		Visibly clea	Yes, some	Bar soap fo
1 2 3 5 10	5	Did not dry		Left in the bucket		Visibly clea	Yes, alway	Bathing soa
1 2 3 4 5 6	15	Air dried		Poured out into the op		Visibly clea	Yes, some	Bar soap, s
1 2 3 4 5 6	60	Did not dry		Poured out in a draina		Visibly clea	Yes, alway	Bar soap, c
1 2 3 4 6 1	10	Did not dry		Left in the bucket		Visibly clea	Yes, some	Bar soap, s

1 2 3 4 5 6	20	Air dried		Poured out into the op	Visibly clea	Yes, some	Liquid soap
1 2 3 4 10	12	Air dried		Left in the bucket	Visibly clea	Yes, alway	Bar soap, s
1 2 3 4 8 1	12	Air dried		Poured out into the op	Visibly clea	Yes, alway	Bar soap, s
1 2 3 4 5 6	15	Using own clothes		Left in the bucket	Visibly clea	Yes, some	Bar soap fo
1 2 4 6 8 9	20	Air dried		Left in the bucket	Visibly clea	Yes, some	Bar soap, s
1 2 3 4 7 1	6	Air dried		Poured out into the op	Visibly clea	Yes, alway	Bar soap, s
1 2 3 5 10	10	Did not dry		Poured out into the op	Visibly clea	Yes, alway	Bar soap fo
1 2 3 4 5 6	15	Using own clothes		Poured out into the op	Visibly clea	Yes, alway	Bar soap, s
1 2 3 5 7 8	3	Using own clothes		Left in the bucket	Visibly clea	Yes, alway	Bar soap, c
1 2 3 4 96	8	Did not dry		Left in the bucket	Visibly clea	Yes, some	Bar soap fo
2 3 4 5 6 1	51	Using own clothes		Left in the bucket	Visibly clea	Yes, some	Liquid soap
1 2 3 4 10	5	Did not dry		Drains into the open	Visibly clea	Yes, some	Bar soap fo
1 2 3 4 6 1	60	Using other piece of cl		Left in the bucket	Visibly clea	Yes, some	Bar soap fo
1 2 3 4 5 6	20	Air dried		Drains into the open	Visibly clea	Yes, some	Bar soap fo
1 2 3 6 8 9	8	Air dried		Poured out into the op	Visibly clea	Yes, some	Bar soap, s
1 2 4 5 7 8	20	Air dried		Poured out into the op	Visibly clea	Yes, some	Bar soap, s
1 2 3 6 11	4	Using own clothes		Poured out into the op	Visibly clea	Yes, alway	Bar soap, s
1 2 3 4 5 6	3	Did not dry		Left in the bucket	Visibly clea	Yes, alway	Bar soap, s
1 2 3 4 11	30	Using other piece of cl		Left in the bucket	Visibly clea	Yes, some	Bar soap, c
1 2 5 11	8	Did not dry		Left in the bucket	Visibly clea	Yes, some	Bathing soa
1 2 3 11 12	2	Using own clothes		Poured out into the op	Visibly clea	Yes, some	Bar soap, s
1 2 3 4 6 8	67	Did not dry		Poured out in a draina	Visibly clea	Yes, alway	Liquid soap
1 2 3 96	5	Other (Spe	did not der	Other (Spe did not der	Visibly clea	Yes, alway	Other (spe
1 5 9 11	10	Using own clothes		Left in the bucket	Visibly clea	Yes, some	Detergent/p
1 2 3 4 5 6	20	Using other piece of cl		Poured out into the op	Visibly clea	Yes, some	Bar soap, s
1 2 3 4 5 1	14	Did not dry		Left in the bucket	Visibly clea	Yes, alway	Liquid soap
1 2 3 4 5 6	90	Did not dry		Poured out into the op	Visibly clea	Yes, alway	Bar soap, c
1 2 3 4 5 7	15	Air dried		Poured out into the op	Visibly clea	Yes, alway	Bathing soa
1 2 7 12	3	Air dried		Poured in toilet/bathro	Visibly clea	Yes, some	Bathing soa
1 2 3 4 6 7	14	Air dried		Poured out into the op	Visibly clea	Yes, alway	Bar soap, s
2 3 4 5 11	47	Did not dry		Left in the bucket	Visibly clea	Yes, alway	Liquid soap
1 2 3 4 5 1	4	Air dried		Left in the bucket	Visibly clea	Yes, some	Bar soap fo
1 2 3 4 11	5	Did not dry		Drains into the open	Visibly clea	Yes, alway	Bar soap, c
1 2 4 5 6 7	30	Using other piece of cl		Poured out in a draina	Visibly clea	Yes, alway	Bar soap, s
1 2 3 4 11	7	Using own clothes		Drains into the open	Visibly clea	Yes, some	Bar soap fo
1 2 3 4 5 6	25	Air dried		Left in the bucket	Visibly clea	Yes, some	Bar soap, c
1 2 3 4 11	8	Using own clothes		Poured out into the op	Visibly clea	Yes, alway	Bar soap fo
1 2 3 4 5 9	50	Did not dry		Left in the bucket	Visibly clea	Yes, some	Bathing soa
1 2 3 4 5 6	20	Air dried		Poured out into the op	Visibly clea	Yes, some	Bathing soa
2 3 4 5 11	34	Using own clothes		Left in the bucket	Visibly clea	Yes, some	Liquid soap
1 2 3 4 5 6	14	Air dried		Poured out in a draina	Visibly clea	Yes, alway	Bar soap, s
1 2 3 4 6 9	35	Did not dry		Poured out into the op	Visibly clea	Yes, alway	Liquid soap
2 3 4 5 11	51	Did not dry		Left in the bucket	Visibly clea	Yes, some	Liquid soap
1 2 3 4 5 6	25	Air dried		Left in the bucket	Visibly clea	Yes, some	Bathing soa
1 2 3 4 8 1	5	Using own clothes		Left in the bucket	Visibly clea	Yes, alway	Bar soap, s
1 2 3 4 11	10	Did not dry		Left in the bucket	Visibly clea	Yes, some	Bar soap, c
1 2 3 4 6 8	64	Using own clothes		Poured out in a draina	Visibly clea	Yes, alway	Bathing soa
1 2 5 11	9	Did not dry		Left in the bucket	Visibly clea	Yes, some	Bathing soa
1 2 3 4 5 6	20	Air dried		Drains into the open	Visibly clea	Yes, some	Bathing soa

1 2 3 4 5 6	60	Air dried	Drains into the open	Visibly clea	Yes, some	Bar soap, c
1 2 3 4 6 9	45	Using other piece of cl	Left in the bucket	Visibly clea	Yes, alway	Bar soap, c
1 2 3 4 5 6	20	Air dried	Poured out into the op	Visibly clea	Yes, some	Bar soap, s
1 2 3 4 11	3	Using other piece of cl	Left in the bucket	Visibly clea	Yes, some	Bar soap, s
1 2 3 4 11	5	Using other piece of cl	Poured out into the op	Visibly clea	Yes, some	Bar soap, s
1 2 3 4 5 6	10	Air dried	Poured out into the op	Visibly clea	Yes, alway	Bar soap, s
1 2 3 4 5 6	90	Air dried	Poured out into the op	Visibly clea	Yes, alway	Bar soap, c
1 2 3 4 5 6	60	Using other piece of cl	Drains into the open	Visibly clea	Yes, some	Bar soap fo
1 2 3 4 11	3	Using own clothes	Poured out in a draina	Visibly clea	Yes, alway	Liquid soap
1 2 3 4 5 7	20	Air dried	Poured out into the op	Visibly clea	Yes, some	Bathing soa
1 2 3 4 5 6	10	Air dried	Left in the bucket	Visibly clea	Yes, some	Bar soap fo
1 2 4 5 6 7	20	Did not dry	Left in the bucket	Visibly clea	Yes, alway	Liquid soap
1 2 3 4 7 1	23	Air dried	Left in the bucket	Visibly clea	Yes, alway	Liquid soap
1 2 3 4 5 6	15	Air dried	Drains into the open	Visibly clea	Yes, some	Detergent/p
1 2 3 4 5 6	2	Air dried	Left in the bucket	Visibly clea	Yes, alway	Bar soap, c
1 2 3 4 5 6	15	Air dried	Poured out into the op	Visibly clea	Yes, alway	Bar soap, s
1 2 3 4 6 7	10	Using other piece of cl	Left in the bucket	Visibly clea	Yes, alway	Liquid soap
1 2 3 4 8 1	4	Using own clothes	Poured out into the op	Visibly clea	Yes, some	Bar soap, s
2 3 4 5 11	120	Did not dry	Poured out into the op	Visibly clea	Yes, alway	Liquid soap
1 2 3 4 11	40	Did not dry	Left in the bucket	Visibly clea	Yes, some	Liquid soap
1 2 3 4 6 8	2	Air dried	Left in the bucket	Visibly clea	Yes, alway	Bar soap, c
1 2 3 4 5 6	15	Using other piece of cl	Pours into the sink	Visibly clea	Yes, alway	Liquid soap
1 2 3 4 5 6	15	Did not dry	Pours into the sink	Visibly clea	Yes, alway	Bar soap, s
1 2 3 4 9 1	60	Using other piece of cl	Poured out in a draina	Visibly clea	Yes, alway	Bathing soa
1 2 3 4 10	5	Using own clothes	Poured out in a draina	Visibly clea	Yes, alway	Bar soap fo
1 2 3 5 8 1	21	Using other piece of cl	Poured out in a draina	Visibly clea	Yes, some	Bathing soa
1 2 3 4 10	10	Did not dry	Pours into the sink	Visibly clea	Yes, alway	Liquid soap
1 2 3 4 9 1	45	Did not dry	Left in the bucket	Visibly clea	Yes, some	Bar soap, s
1 2 3 4 5 6	120	Did not dry	Poured out into the op	Visibly clea	Yes, alway	Bathing soa
2 3 4 5 96	42	Did not dry	Poured out into the op	Visibly clea	Yes, some	Liquid soap
1 2 4 10	5	Did not dry	Left in the bucket	Visibly clea	Yes, some	Bar soap, c
1 2 3 4 5 8	10	Using own clothes	Left in the bucket	Visibly clea	Yes, alway	Bar soap fo
2 4 5 11	33	Did not dry	Left in the bucket	Visibly clea	Yes, alway	Detergent/p
1 3 6 7 10	20	Air dried	Poured out into the op	Visibly clea	Yes, some	Bar soap, s
1 2 5 6 7 8	250	Using other piece of cl	Poured out into the op	Visibly clea	Yes, some	Liquid soap
1 4 6 11	10	Air dried	Poured out in a draina	Visibly clea	Yes, alway	Bar soap fo
1 2 4 8 10	20	Using own clothes	Left in the bucket	Visibly clea	Yes, alway	Bathing soa
2 3 4 5 6 1	56	Using own clothes	Left in the bucket	Visibly clea	Yes, alway	Liquid soap
1 2 3 4	10	Air dried	Poured out into the op	Visibly clea	Yes, some	Detergent/p
1 2 9 10	5	Did not dry	Poured out into the op	Visibly clea	Yes, alway	Bathing soa
2 4 5 7 9 1	20	Air dried	Poured out into the op	Visibly clea	Yes, some	Liquid soap
1 2 3 4 10	30	Using other piece of cl	Left in the bucket	Visibly clea	Yes, alway	Liquid soap
1 2 3 4 5 6	15	Using other piece of cl	Poured out into the op	Visibly clea	Yes, some	Bar soap fo
1 2 3 4 5 6	5	Did not dry	Left in the bucket	Visibly clea	Yes, alway	Bar soap fo
1 2 3 4 5 6	15	Using other piece of cl	Poured out into the op	Visibly clea	Yes, some	Bathing soa
1 2 3 4 5 6	37	Did not dry	Pours into the sink	Visibly clea	Yes, some	Bathing soa
1 2 3 4 5 6	10	Did not dry	Left in the bucket	Visibly clea	Yes, alway	Bar soap, c
1 2 3 4 5 6	60	Did not dry	Drains into the open	Visibly clea	Yes, alway	Liquid soap
1 2 5 6 11	12	Air dried	Left in the bucket	Visibly clea	Yes, some	Bar soap, s

1 2 3	5	Air dried		Poured out into the op	Visibly clea	Yes, some	Liquid soap
1 2 3 4 6 9	30	Did not dry		Left in the bucket	Visibly clea	Yes, some	Bar soap fo
1 2 3 4 12	5	Using other piece of cl		Poured out into the op	Visibly clea	Yes, alway	Bar soap fo
1 2 3 4 5 6	3	Air dried		Poured out into the op	Visibly clea	Yes, alway	Bar soap fo
1 2 3 4 5 6	10	Air dried		Poured out into the op	Visibly clea	Yes, alway	Bar soap, s
2 3 4 5 11	34	Did not dry		Left in the bucket	Visibly clea	Yes, some	Bar soap, c
1 2 4 5 7 8	20	Air dried		Left in the bucket	Visibly clea	Yes, some	Bar soap, s
2 3 4 5 6 1	55	Using own clothes		Poured out into the op	Visibly clea	Yes, some	Liquid soap
1 3 4 5 6 9	20	Did not dry		Poured out into the op	Visibly clea	Yes, alway	Detergent/p
1 2 3 5 6 7	20	Did not dry		Poured out in a draina	Visibly clea	Yes, alway	Bathing soa
2 3 4 5 11	35	Did not dry		Left in the bucket	Visibly clea	Yes, some	Liquid soap
1 2 3 4 5 6	2	Air dried		Left in the bucket	Visibly clea	Yes, some	Bar soap, s
1 2 3 5 6 1	30	Did not dry		Poured out into the op	Visibly clea	Yes, alway	Liquid soap
1 2 3 4 8 1	4	Did not dry		Left in the bucket	Visibly clea	Yes, some	Liquid soap
1 2 3 4 5 6	20	Air dried		Drains into the open	Visibly clea	Yes, some	Bar soap, c
1 2 3 4 11	7	Did not dry		Poured out in a draina	Visibly clea	Yes, some	Bar soap, c
1 2 3 4 5 6	40	Using other piece of cl		Poured out in a draina	Visibly clea	Yes, some	Bar soap, s
1 2 3 4 5 1	6	Did not dry		Pours into the sink	Visibly clea	Yes, alway	Bar soap, c
1 2 3 4 5 6	30	Using other piece of cl		Poured out in a draina	Visibly clea	Yes, alway	Bar soap, c
1 3 4 8 11	10	Using own clothes		Poured out into the op	They appe	Yes, some	Detergent/p
1 2 3 4 5 6	15	Using own clothes		Left in the bucket	Visibly clea	Yes, some	Bathing soa
1 2 3 4 5 7	3	Did not dry		Left in the bucket	Visibly clea	Yes, alway	Bar soap, s
1 2 3 4 11	5	Using own clothes		Drains into the open	Visibly clea	Yes, some	Bar soap, s
1 2 5 6 11	0	Other (Spe	She said sl	Poured out into the op	They appe	Yes, some	Liquid soap
1 2 3 4 5 6	20	Air dried		Poured out into the op	Visibly clea	Yes, some	Bar soap, s
1 2 3 4 11	5	Did not dry		Drains into the open	Visibly clea	Yes, some	Bar soap fo
1 2 3 4 5 7	20	Did not dry		Left in the bucket	Visibly clea	Yes, alway	Bathing soa
1 2 3 4 5 6	15	Air dried		Poured out into the op	Visibly clea	Yes, alway	Bar soap, s
1 2 3 4	15	Did not dry		Left in the bucket	Visibly clea	Yes, alway	Bar soap, s
1 2 4 9 11	20	Air dried		Left in the bucket	Visibly clea	Yes, alway	Bathing soa
1 2 3 4 5 1	10	Did not dry		Poured out into the op	Visibly clea	Yes, some	Bar soap, c
1 2 3 11 12	3	Using own clothes		Poured out into the op	Visibly clea	Yes, some	Liquid soap
1 2 3 4 5 6	10	Did not dry		Left in the bucket	Visibly clea	Yes, some	Bar soap fo
1 2 3 4 5 1	15	Did not dry		Drains into the open	Visibly clea	Yes, alway	Bar soap, c
1 2 3 4 5 6	6	Air dried		Poured out into the op	Visibly clea	Yes, alway	Bar soap, s
1 2 3 4 5 7	15	Air dried		Poured out into the op	Visibly clea	Yes, some	Bathing soa
1 2 3 6 8 1	20	Air dried		Poured out into the op	Visibly clea	Yes, some	Bar soap, s
1 2 5 10 12	296	Using own clothes		Left in the bucket	Visibly clea	Yes, some	Liquid soap
1 2 3 4 5 7	14	Air dried		Poured out into the op	Visibly clea	Yes, alway	Bar soap, s
1 2 4 5 6 8	26	Air dried		Poured out in a draina	Visibly clea	Yes, alway	Bar soap, s
1 2 3 4 5 6	16	Air dried		Poured out into the op	Visibly clea	Yes, alway	Bar soap, s
1 2 3 11 12	10	Did not dry		Poured out into the op	Visibly clea	Yes, some	Bar soap fo
1 2 3 4 5 1	20	Using own clothes		Poured out in a draina	Visibly clea	Yes, alway	Bar soap, s
1 2 3 4 6 8	7	Using own clothes		Poured out into the op	Visibly clea	Yes, some	Bar soap, s
1 2 3 4 5 6	8	Air dried		Poured out into the op	Visibly clea	Yes, alway	Bar soap, s
1 2 3 4 6 8	3	Did not dry		Left in the bucket	Visibly clea	Yes, some	Bar soap, s
1 2 3 4 5 6	8	Air dried		Poured out into the op	Visibly clea	Yes, alway	Liquid soap
1 2 5 12	0	Using own clothes		Poured out in a draina	They appe	Yes, some	Liquid soap
2 4 5 11 13	56	Using own clothes		Drains into the open	Visibly clea	Yes, alway	Bar soap, c

1 2 4 5 10	20	Using own clothes	Pours into the sink	Visibly clea	Yes, alway	Bathing soa
1 2 3 4 5 6	20	Using other piece of cl	Left in the bucket	Visibly clea	Yes, some	Bathing soa
1 2 4 10 12	20	Air dried	Poured out in a draina	Visibly clea	Yes, some	Bathing soa
2 3 4 11	30	Did not dry	Left in the bucket	Visibly clea	Yes, some	Liquid soap
1 2 4 11	3	Did not dry	Poured out into the op	Visibly clea	Yes, some	Bathing soa
1 2 4 5 6 7	25	Did not dry	Left in the bucket	Visibly clea	Yes, alway	Bathing soa
1 2 5 6 8 1	20	Using own clothes	Drains into the open	Visibly clea	Yes, alway	Bar soap, s
1 2 3 4 5 6	10	Air dried	Left in the bucket	Visibly clea	Yes, some	Bathing soa
1 2 3 5 6 9	24	Did not dry	Poured out into the op	Visibly clea	Yes, alway	Detergent/p
1 2 3 5 8 1	13	Using own clothes	Poured out into the op	Visibly clea	Yes, some	Bathing soa
1 2 3 5 6 7	27	Using own clothes	Drains into the open	Visibly clea	Yes, alway	Detergent/p
1 2 3 4 5 6	20	Using other piece of cl	Poured out into the op	Visibly clea	Yes, some	Bathing soa
1 2 3 4 6 1	15	Air dried	Left in the bucket	Visibly clea	Yes, alway	Liquid soap
1 2 4 11 12	15	Using own clothes	Left in the bucket	Visibly clea	Yes, alway	Bar soap, s
1 2 4 9 11	15	Did not dry	Left in the bucket	Visibly clea	Yes, alway	Bar soap, c
1 2 3 4 5 6	3	Using other piece of cl	Poured out into the op	Visibly clea	Yes, alway	Bathing soa
1 2 3 4 5 1	2	Using own clothes	Left in the bucket	Visibly clea	Yes, alway	Bar soap, c
1 2 3 4 5 8	10	Using own clothes	Poured out into the op	Visibly clea	Yes, alway	Bar soap, s
1 2 3 4 11	5	Using own clothes	Drains into the open	Visibly clea	Yes, alway	Bar soap, s
1 2 3 5 6 7	37	Air dried	Poured out in a draina	Visibly clea	Yes, alway	Bathing soa
1 2 4 6 11	4	Using own clothes	Poured out into the op	Visibly clea	Yes, alway	Bar soap, s
1 2 3 4 5 6	2	Using own clothes	Left in the bucket	Visibly clea	Yes, some	Liquid soap
1 2 5 11	9	Did not dry	Poured out into the op	Visibly clea	Yes, some	Bathing soa
1 2 3 4 5 7	3	Did not dry	Left in the bucket	Visibly clea	Yes, alway	Bathing soa
1 2 3 4 5 6	10	Other (Spe demonstra	Other (Spe demonstra	Visibly clea	Yes, alway	Bar soap, c
1 2 3 7 8 1	2	Air dried	Left in the bucket	Visibly clea	Yes, alway	Bar soap, s
1 2 3 4 5 6	10	Using own clothes	Left in the bucket	Visibly clea	Yes, alway	Bar soap, s
1 2 3 4 6 8	10	Using own clothes	Left in the bucket	Visibly clea	Yes, alway	Bar soap, s
1 2 3 4 5 7	46	Did not dry	Poured out in a draina	Visibly clea	Yes, alway	Bathing soa
1 2 3 4 5 8	15	Using own clothes	Left in the bucket	Visibly clea	Yes, some	Bar soap, c
1 2 3	10	Air dried	Poured out into the op	Visibly clea	Yes, some	Bar soap, s
1 2 3 4 5 1	10	Air dried	Left in the bucket	Visibly clea	Yes, some	Bathing soa
1 2 3 4 5 6	15	Air dried	Poured out into the op	Visibly clea	Yes, some	Bathing soa
1 2 4 7 9 1	20	Air dried	Poured out in a draina	Visibly clea	Yes, some	Bar soap, s
1 2 3 4 6 1	5	Using own clothes	Poured out into the op	Visibly clea	Yes, alway	Liquid soap
1 2 3 4 5 6	28	Air dried	Drains into the open	Visibly clea	Yes, alway	Bar soap, s
1 2 3 4 11	10	Air dried	Left in the bucket	Visibly clea	Yes, some	Bar soap, s
1 2 4 5 6 8	20	Using own clothes	Poured out in a draina	Visibly clea	Yes, alway	Bathing soa
1 2 3 4 5 6	8	Air dried	Poured out into the op	Visibly clea	Yes, alway	Bar soap, s
1 2 3 5 11	7	Did not dry	Poured out into the op	Visibly clea	Yes, alway	Bathing soa
1 2 4 5 7 9	15	Air dried	Poured out into the op	Visibly clea	Yes, some	Bar soap, s
1 2 3 4 5 8	10	Using own clothes	Left in the bucket	Visibly clea	Yes, some	Bar soap, s
1 2 11	3	Did not dry	Poured out in a draina	Visibly clea	Yes, some	Bathing soa
1 2 5 10 12	10	Air dried	Poured out in a draina	Visibly clea	Yes, some	Bathing soa
1 2 3 4 5 7	14	Air dried	Poured out into the op	Visibly clea	Yes, alway	Bar soap, s
1 2 3 4 11	10	Air dried	Poured out into the op	Visibly clea	Yes, alway	Bathing soa
1 2 3 8 10	2	Air dried	Poured out into the op	Visibly clea	Yes, alway	Bar soap, c
1 2 3 4 5 1	15	Air dried	Poured out in a draina	Visibly clea	Yes, some	Bar soap, c
1 2 3 4 11	10	Using other piece of cl	Poured out into the op	Visibly clea	Yes, some	Bar soap fo

1 2 4 6 7 1	6	Using other piece of cl		Poured out in a draina	Visibly clea	Yes, alway	Bar soap, c
2 3 4 5 6 7	60	Air dried		Poured out into the op	Visibly clea	Yes, alway	Liquid soap
1 3 5 6 7 9	20	Air dried		Poured out into the op	Visibly clea	Yes, some	Bar soap, s
1 2 3 4 5 6	15	Air dried		Pours into the sink	Visibly clea	Yes, alway	Liquid soap
96	0	Other (Spe	did not dem	Other (Spe	did not der	Visibly clea	Yes, alway	Liquid soap
1 2 3 4 6 7	33	Did not dry		Poured out in a draina	Visibly clea	Yes, alway	Bathing soa
1 4 7 9 11	20	Air dried		Poured out in a draina	Visibly clea	Yes, alway	Bar soap, s
1 2 3 4 5 6	40	Using other piece of cl		Poured out into the op	Visibly clea	Yes, some	Bathing soa
1 2 3 4 5 6	5	Did not dry		Pours into the sink	Visibly clea	Yes, alway	Liquid soap
96	0	Other (Spe	did not dem	Other (Spe	did not der	Visibly clea	Yes, alway	Bar soap, c
2 3 4 7 8 1	25	Air dried		Poured out into the op	Visibly clea	Yes, alway	Detergent/p
1 3 4 10 12	4	Using own clothes		Left in the bucket	Visibly clea	Yes, some	Bar soap, s
1 2 3 4 5 7	20	Using other piece of cl		Pours into the sink	Visibly clea	Yes, alway	Bathing soa
1 2 3 4 9	30	Did not dry		Poured out into the op	Visibly clea	Yes, some	Bathing soa
1 2 3 4 5 6	60	Did not dry		Poured out into the op	Visibly clea	Yes, some	Bar soap fo
1 2 3 4 5 6	12	Air dried		Poured out into the op	Visibly clea	Yes, alway	Bar soap, s
1 2 3 4 5 1	190	Using own clothes		Poured out into the op	Visibly clea	Yes, some	Liquid soap
1 2 3 4 8 1	12	Air dried		Poured out into the op	Visibly clea	Yes, some	Bar soap, s
1 2 3 4 5 1	15	Air dried		Drains into the open	Visibly clea	Yes, some	Liquid soap
1 2 4 5 6 1	200	Using own clothes		Left in the bucket	Visibly clea	Yes, some	Detergent/p
1 2 3 4 5 7	10	Did not dry		Poured out into the op	Visibly clea	Yes, alway	Bar soap, s
1 2 3 4 5 8	10	Did not dry		Left in the bucket	Visibly clea	Yes, alway	Bathing soa
1 2 4 11 12	34	Using own clothes		Left in the bucket	Visibly clea	Yes, some	Liquid soap
1 2 3 4 5 6	20	Air dried		Poured out in a draina	Visibly clea	Yes, alway	Bathing soa
1 2 3 4 5 7	5	Using own clothes		Left in the bucket	Visibly clea	Yes, alway	Bathing soa
1 2 3 4 5 6	15	Air dried		Left in the bucket	Visibly clea	Yes, some	Bathing soa
1 2 3 4 6 7	58	Air dried		Drains into the open	Visibly clea	Yes, alway	Detergent/p
1 2 3 4 5 6	12	Air dried		Poured out in a draina	Visibly clea	Yes, alway	Liquid soap
2 4 5 6 11	37	Using own clothes		Left in the bucket	Visibly clea	Yes, some	Detergent/p
1 2 3 4 5 6	6	Did not dry		Drains into the open	Visibly clea	Yes, alway	Bar soap, s
1 2 3 4 6 7	3	Did not dry		Left in the bucket	Visibly clea	Yes, alway	Bar soap, s
1 2 3 4 5 6	14	Air dried		Poured out into the op	Visibly clea	Yes, alway	Bar soap, s
1 2 3 11	10	Did not dry		Left in the bucket	Visibly clea	Yes, some	Liquid soap
1 2 3 4 12	5	Using own clothes		Left in the bucket	Visibly clea	Yes, some	Bar soap fo
1 2 4 5 8 9	20	Air dried		Poured out in a draina	Visibly clea	Yes, alway	Bar soap, s
1 2 4 5 9 1	20	Air dried		Poured out in a draina	Visibly clea	Yes, alway	Bar soap, s
1 2 3 4 5 6	67	Using other piece of cl		Poured out into the op	Visibly clea	Yes, alway	Detergent/p
1 2 5 11	5	Did not dry		Left in the bucket	Visibly clea	Yes, alway	Bathing soa
1 2 3 4 5 7	25	Using own clothes		Pours into the sink	Visibly clea	Yes, alway	Bathing soa
1 2 3 4 5 6	120	Using other piece of cl		Left in the bucket	Visibly clea	Yes, some	Bar soap fo
1 2 3 5 8 1	1	Air dried		Poured out into the op	Visibly clea	Yes, some	Bar soap, s
1 2 3 5 6 7	20	Air dried		Poured out into the op	Visibly clea	Yes, some	Bar soap, s
1 2 3 4 5 9	10	Using other piece of cl		Poured out into the op	Visibly clea	Yes, some	Bathing soa
1 2 3 4 5 6	45	Using other piece of cl		Poured out into the op	Visibly clea	Yes, alway	Bar soap, s
1 2 3 4 11	5	Using own clothes		Left in the bucket	Visibly clea	Yes, alway	Bar soap fo
1 2 3 4 6 8	2	Did not dry		Left in the bucket	Visibly clea	Yes, some	Bar soap, s
1 2 5 9 11	140	Air dried		Left in the bucket	They appe	Yes, some	Liquid soap
1 2 3 4 5 6	120	Using other piece of cl		Poured out in a draina	Visibly clea	Yes, alway	Bathing soa
1 2 4 5 6 7	20	Air dried		Poured out into the op	Visibly clea	Yes, some	Bar soap, s

1 2 3 4 7 1	10	Air dried		Poured out into the op		Visibly clea	Yes, some	Bathing soa
1 2 3 4 5 6	13	Air dried		Poured out in a draina		Visibly clea	Yes, some	Bathing soa
1 2 3 6 11	4	Air dried		Poured out into the op		Visibly clea	Yes, alway	Bathing soa
1 2 3 4 5 6	25	Air dried		Poured out in a draina		Visibly clea	Yes, some	Bar soap fo
96	0	Other (Spe	did not den	Other (Spe	did not der	Visibly clea	Yes, alway	Liquid soap
1 2 3 4 5 6	30	Using own clothes		Left in the bucket		They appe	Yes, some	Bathing soa
1 2 4 11	5	Did not dry		Pours into the sink		Visibly clea	Yes, alway	Liquid soap
1 2 3 4 11	10	Did not dry		Poured out into the op		Visibly clea	Yes, some	Bathing soa
1 2 3 4 11	45	Air dried		Left in the bucket		Visibly clea	Yes, some	Bar soap fo
1 2 3 4 10	5	Using own clothes		Left in the bucket		Visibly clea	Yes, some	Bar soap, s
1 2 3 4 5 7	47	Did not dry		Poured out in a draina		Visibly clea	Yes, alway	Detergent/p
1 2 3	10	Air dried		Poured out into the op		Visibly clea	Yes, some	Bar soap, s
2 5 11	30	Using own clothes		Left in the bucket		Visibly clea	Yes, alway	Liquid soap
1 2 3 4 6 1	180	Using other piece of cl		Poured out into the op		Visibly clea	Yes, alway	Liquid soap
1 2 4 6 7 9	64	Did not dry		Poured out into the op		Visibly clea	Yes, alway	Liquid soap
1 2 3 4 5 6	20	Using own clothes		Left in the bucket		Visibly clea	Yes, some	Bathing soa
1 2 3 4	10	Did not dry		Left in the bucket		Visibly clea	Yes, alway	Bar soap fo
1 2 3 4 5 6	10	Using other piece of cl		Poured in toilet/bathro		Visibly clea	Yes, some	Bathing soa
1 2 3 6 8 1	4	Did not dry		Poured out into the op		Visibly clea	Yes, some	Bar soap, s
1 2 3 4 6 7	20	Air dried		Poured out into the op		Visibly clea	Yes, some	Bar soap, s
1 2 4 5 7 9	20	Air dried		Poured out into the op		Visibly clea	Yes, some	Bathing soa
96	0	Other (Spe	Did not wa	Other (Spe	NA	They appe	Yes, alway	Bar soap, s
1 2 3 4 5 6	120	Using serviettes/tissue		Poured in toilet/bathro		Visibly clea	Yes, alway	Bar soap fo
2 3 4 5 10	47	Did not dry		Left in the bucket		Visibly clea	Yes, alway	Liquid soap
96	0	Other (Spe	Did not wa	Other (Spe	NA	They appe	Yes, some	Liquid soap

q48b	q50a	q50b	q60b	q61	q61_label	q62a	q62b	q63a
oowder soa	Detergent/powder soap			Compound, shared		Flush to pit latrine		Flush/pour
dedicated o	Bar soap			Compound, shared		Pit latrine with concret		Pit latrine w
oowder soa	Detergent/powder soap			In the household		Flush to septic tank		Flush/pour
	Detergent/powder soap			Compound, shared		Flush to pit latrine		Flush/pour
o, dedicatec	Liquid soap			Compound, not sharec		Flush to pit latrine		Flush/pour
	Bar soap			Compound, shared		Flush to piped sewer s		Flush/pour
oowder soa	Detergent/powder soap			Compound, shared		Flush to pit latrine		Flush/pour
oowder soa	Detergent/powder soap			Compound, not sharec		Flush to pit latrine		Flush/pour
oowder soa	Bar soap			Compound, shared		Pit latrine with concret		Pit latrine w
same as wh	Bar soap			Compound, shared		Pit latrine with concret		Pit latrine w
ap, same a	Bar soap			Neighboring compoun		Flush to septic tank		Flush/pour
same as wh	Bar soap			Compound, shared		Flush to septic tank		Flush/pour
o, dedicatec	Liquid soap			Compound, not sharec		Flush to pit latrine		Flush/pour
or all house	Bar soap			Compound, shared		Pit latrine with concret		Pit latrine w
ap, same a	Bar soap			Compound, shared		Pit latrine with concret		Pit latrine w
dedicated o	Bar soap			Compound, shared		Pit latrine with concret		Pit latrine w
	Other (spe	Does not use soap for		Compound, shared		Pit latrine without slab		Pit latrine w
oowder soa	Detergent/powder soap			Compound, not sharec		Flush to septic tank		Flush/pour
dedicated o	Bar soap			Compound, shared		Flush to piped sewer s		Flush/pour
oowder soa	Detergent/powder soap			Public latrine		Flush to pit latrine		Flush/pour
dedicated o	Bar soap			Compound, shared		Flush to pit latrine		Flush/pour
ap, same a	Bar soap			Compound, shared		Pit latrine with concret		Pit latrine w
or all house	Bar soap			Compound, shared		Pit latrine with concret		Pit latrine w
same as wh	Bar soap			Compound, not sharec		Pit latrine without slab		Pit latrine w
same as wh	Bar soap			Compound, shared		Pit latrine with concret		Pit latrine w
same as wh	Bar soap			Compound, shared		Pit latrine with concret		Pit latrine w
ap, same a	Bathing soap			Compound, not sharec		Pit latrine with concret		Pit latrine w
o, dedicatec	Liquid soap			Compound, shared		Flush to piped sewer s		Flush/pour
same as wh	Bar soap			Compound, shared		Pit latrine with concret		Pit latrine w
o, dedicatec	Liquid soap			Compound, not sharec		Flush to septic tank		Flush/pour
same as wh	Bar soap			Compound, shared		Pit latrine with concret		Pit latrine w
o, dedicatec	Liquid soap			In the household		Flush to septic tank		Flush/pour
or all house	Bar soap			Compound, shared		Flush to septic tank		Flush/pour
same as wh	Bar soap			Compound, not sharec		Flush to septic tank		Flush/pour
o, dedicatec	Liquid soap			In the household		Pit latrine with concret		Pit latrine w
o, dedicatec	Liquid soap			Neighboring compoun		Pit latrine with concret		Pit latrine w
ap, same a	Bar soap			Compound, shared		Flush to pit latrine		Flush/pour
oowder soa	Detergent/powder soap			Compound, not sharec		Flush to septic tank		Flush/pour
same as wh	Bar soap		Neighbouring toilet/compound					Pit latrine w
dedicated o	Bar soap			Compound, shared		Flush to pit latrine		Flush/pour
same as wh	Liquid soap			Compound, shared		Pit latrine with concret		Pit latrine w
same as wh	Bar soap			Compound, shared		Flush to pit latrine		Flush/pour
o, dedicatec	Liquid soap			In the household		Pit latrine with concret		Pit latrine w
ap, same a	Bar soap			In the household		Pit latrine with concret		Pit latrine w
same as wh	Liquid soap			Compound, shared		Flush to piped sewer s		Flush/pour
ap, same a	Liquid soap			Neighboring compoun		Flush to piped sewer s		Flush/pour
o, dedicatec	Liquid soap			In the household		Flush to piped sewer s		Flush/pour
o, dedicatec	Liquid soap			Neighboring compoun		Flush to septic tank		Flush/pour

), dedicatec	Liquid soap			Compound, shared	Flush to pit latrine	Flush/pour
	Bar soap			Compound, shared	Flush to pit latrine	Flush/pour
), dedicatec	Liquid soap		Open spaces/bush			No facility/t
), also for o	Liquid soap			In the household	Flush to pit latrine	Flush/pour
), dedicatec	Liquid soap			Compound, not sharec	Flush to septic tank	Flush/pour
ap, same a:	Bar soap			Compound, shared	Pit latrine with concret	Pit latrine v
), dedicatec	Liquid soap			Compound, shared	Pit latrine with concret	Pit latrine v
oowder soa	Detergent/powder soap			Compound, shared	Flush to piped sewer s	Flush/pour
), dedicatec	Liquid soap			Compound, not sharec	Flush to septic tank	Flush/pour
ap, same a:	Liquid soap			Compound, not sharec	Pit latrine with concret	Pit latrine v
), dedicatec	Liquid soap			Compound, shared	Flush to pit latrine	Flush/pour
dedicated o	Bar soap			Neighboring compoun	Flush to septic tank	Flush/pour
or all house	Bar soap			Compound, shared	Pit latrine with concret	Pit latrine v
), dedicatec	Liquid soap			In the household	Flush to pit latrine	Flush/pour
), dedicatec	Liquid soap			Compound, not sharec	Pit latrine with concret	Pit latrine v
), dedicatec	Liquid soap			In the household	Flush to septic tank	Flush/pour
same as wh	Bar soap			Compound, shared	Flush to piped sewer s	Flush/pour
), dedicatec	Liquid soap			Compound, shared	Flush to piped sewer s	Flush/pour
ap, same a:	Bathing soap			Compound, shared	Flush to piped sewer s	Flush/pour
ap, same a:	Bar soap			Compound, shared	Flush to pit latrine	Flush/pour
ap, same a:	Bathing soap			Compound, shared	Pit latrine with concret	Pit latrine v
dedicated o	Bar soap			Compound, not sharec	Flush to pit latrine	Flush/pour
), dedicatec	Liquid soap			Compound, shared	Flush to piped sewer s	Flush/pour
or all house	Bar soap		Neighbouring toilet/compound			Pit latrine v
ap, same a:	Bar soap			Compound, shared	Pit latrine without slab	Pit latrine v
), dedicatec	Liquid soap			Compound, not sharec	Flush to septic tank	Flush/pour
), dedicatec	Liquid soap			Compound, shared	Flush to pit latrine	Flush/pour
same as wh	Bar soap			Compound, shared	Flush to septic tank	Flush/pour
), dedicatec	Liquid soap		Open spaces/bush			No facility/t
same as wh	Bar soap			Compound, shared	Pit latrine with concret	Pit latrine v
Any soap t	Detergent/powder soap			In the household	Flush to donâ€™t kno	Flush/pour
), dedicatec	Liquid soap			Compound, shared	Pit latrine with concret	Pit latrine v
), dedicatec	Liquid soap			In the household	Pit latrine with concret	Pit latrine v
), dedicatec	Liquid soap			Compound, shared	Pit latrine with concret	Pit latrine v
oowder soa	Detergent/powder soap			Compound, not sharec	Pit latrine without slab	Pit latrine v
), dedicatec	Liquid soap			Neighboring compoun	Pit latrine with concret	Pit latrine v
), dedicatec	Liquid soap			Compound, shared	Flush to pit latrine	Flush/pour
same as wh	Bar soap			In the household	Flush to septic tank	Flush/pour
same as wh	Bar soap			Compound, shared	Pit latrine with concret	Pit latrine v
oowder soa	Detergent/powder soap			Neighboring compoun	Flush to pit latrine	Flush/pour
Sanitizer	Other (spe	Sanitizer		Compound, shared	Flush to piped sewer s	Flush/pour
ap, same a:	Bar soap			Compound, shared	Flush to pit latrine	Flush/pour
oowder soa	Detergent/powder soap			Compound, shared	Flush to septic tank	Flush/pour
oowder soa	Detergent/powder soap			Compound, not sharec	Flush to pit latrine	Flush/pour
same as wh	Bar soap			Compound, shared	Flush to pit latrine	Flush/pour
), dedicatec	Liquid soap			Compound, shared	Flush to septic tank	Flush/pour
), dedicatec	Liquid soap			Compound, shared	Flush to pit latrine	Flush/pour
), dedicatec	Liquid soap			In the household	Flush to septic tank	Flush/pour
same as wh	Bar soap			Compound, not sharec	Pit latrine with concret	Pit latrine v

ap, same a	Bar soap			Compound, shared	Flush to septic tank	Flush/pour
), dedicatec	Liquid soap			In the household	Flush to donâ€™t kno	Flush to do
or all house	Bar soap			Community latrine	Flush to septic tank	Flush/pour
), dedicatec	Bar soap			Compound, shared	Pit latrine with concret	Pit latrine v
ap, same a	Bar soap			Compound, shared	Flush to pit latrine	Flush/pour
), dedicatec	Liquid soap			In the household	Pit latrine with concret	Pit latrine v
or all house	Bar soap			Compound, shared	Pit latrine with concret	Pit latrine v
powder soa	Detergent/powder soap			In the household	Flush to pit latrine	Flush/pour
), dedicatec	Liquid soap			Compound, not sharec	Flush to septic tank	Flush/pour
), dedicatec	Liquid soap			Compound, shared	Pit latrine with concret	Pit latrine v
same as wh	Bar soap			Compound, shared	Flush to pit latrine	Flush/pour
same as wh	Bar soap			Neighboring compoun	Flush to piped sewer s	Other (Spe
), dedicatec	Liquid soap			Compound, shared	Pit latrine with concret	Pit latrine v
powder soa	Detergent/powder soap			Compound, shared	Flush to pit latrine	Flush/pour
same as wh	Bar soap		Open spaces/bush			No facility/b
	Liquid soap		Open spaces/bush			No facility/b
ap, same a	Bar soap			Compound, shared	Pit latrine with concret	Pit latrine v
same as wh	Bar soap			In the household	Ventilated improved pi	Flush/pour
), dedicatec	Liquid soap			Compound, shared	Pit latrine with concret	Pit latrine v
), dedicatec	Liquid soap			Compound, shared	Flush to septic tank	Flush/pour
powder soa	Detergent/powder soap			Compound, shared	Pit latrine without slab	Pit latrine v
powder soa	Detergent/powder soap			Compound, shared	Flush to pit latrine	Flush/pour
), dedicatec	Liquid soap			In the household	Flush to septic tank	Flush/pour
), dedicatec	Liquid soap			Compound, not sharec	Flush to septic tank	Flush/pour
dedicated o	Liquid soap			In the household	Flush to septic tank	Flush/pour
same as wh	Bar soap			Compound, shared	Pit latrine with concret	Pit latrine v
powder soa	Detergent/powder soap			Compound, not sharec	Flush to septic tank	Flush/pour
or all house	Bar soap			Compound, shared	Pit latrine with concret	Pit latrine v
ap, same a	Liquid soap			Compound, shared	Flush to piped sewer s	Flush/pour
ap, same a	Bar soap			In the household	Flush to septic tank	Flush/pour
), dedicatec	Liquid soap			Compound, shared	Pit latrine with concret	Pit latrine v
same as wh	Liquid soap			Compound, shared	Pit latrine with concret	Pit latrine v
), dedicatec	Liquid soap			Compound, not sharec	Flush to septic tank	Flush/pour
or all house	Liquid soap			Compound, shared	Pit latrine with concret	Pit latrine v
same as wh	Bar soap			Compound, shared	Pit latrine with concret	Pit latrine v
ap, same a	Liquid soap			Compound, shared	Flush to pit latrine	Flush/pour
or all house	Bar soap			Compound, shared	Pit latrine with concret	Pit latrine v
or all house	Bar soap			Compound, shared	Pit latrine with concret	Pit latrine v
), also for o	Liquid soap			Compound, shared	Flush to pit latrine	Flush/pour
dedicated o	Bar soap			In the household	Flush to septic tank	Flush/pour
), dedicatec	Liquid soap			In the household	Flush to septic tank	Flush/pour
), dedicatec	Liquid soap			In the household	Flush to septic tank	Flush/pour
ap, same a	Liquid soap			Compound, shared	Flush to piped sewer s	Flush/pour
), dedicatec	Liquid soap			Compound, shared	Flush to septic tank	Flush/pour
ap, same a	Bar soap			Compound, shared	Pit latrine with concret	Pit latrine v
), dedicatec	Liquid soap			Compound, not sharec	Flush to pit latrine	Flush/pour
), dedicatec	Liquid soap			Compound, not sharec	Pit latrine with concret	Pit latrine v
), dedicatec	Liquid soap			In the household	Pit latrine with concret	Pit latrine v
ap, same a	Other (spe	Mixture of liquid soap		Compound, shared	Pit latrine with concret	Pit latrine v

o, dedicatec	Liquid soap			Compound, not sharec	Composting/EcoSan	Pit latrine v
dedicated o	Bar soap		Open spaces/bush			No facility/b
ap, same a:	Liquid soap			Compound, shared	Pit latrine with concret	Pit latrine v
ap, same a:	Bar soap			Compound, shared	Flush to pit latrine	Flush/pour
o, dedicatec	Liquid soap			In the household	Flush to septic tank	Flush/pour
same as wh	Bar soap			Compound, shared	Flush to septic tank	Flush/pour
same as wh	Bar soap			Compound, shared	Flush to septic tank	Flush/pour
or all house	Bar soap			Compound, shared	Pit latrine with concret	Pit latrine v
ap, same a:	Bathing soap			Compound, shared	Flush to piped sewer s	Flush/pour
ap, same a:	Bar soap			Compound, not sharec	Pit latrine with concret	Pit latrine v
same as wh	Liquid soap			Compound, shared	Flush to pit latrine	Flush/pour
same as wh	Bar soap			Compound, shared	Pit latrine with concret	Pit latrine v
powder soa	Detergent/powder soap			Compound, shared	Flush to pit latrine	Flush/pour
ap, same a:	Bar soap			Compound, shared	Flush to septic tank	Flush/pour
dedicated o	Bar soap			In the household	Flush to septic tank	Flush/pour
same as wh	Bar soap			Neighboring compoun	Pit latrine with concret	Pit latrine v
dedicated o	Bar soap			Compound, shared	Pit latrine with concret	Pit latrine v
sed	Other (spe	No soap		In the household	Pit latrine without slab	Pit latrine v
same as wh	Bar soap			Compound, not sharec	Flush to septic tank	Flush/pour
o, dedicatec	Liquid soap			In the household	Flush to septic tank	Flush/pour
or all house	Bar soap			Compound, shared	Pit latrine with concret	Pit latrine v
dedicated o	Bar soap			In the household	Flush to septic tank	Flush/pour
o, dedicatec	Liquid soap			Compound, not sharec	Flush to septic tank	Flush/pour
or all house	Bar soap			Compound, shared	Flush to pit latrine	Flush/pour
o, dedicatec	Liquid soap			Compound, shared	Pit latrine without slab	Pit latrine v
same as wh	Bar soap			Compound, not sharec	Pit latrine without slab	Pit latrine v
same as wh	Bar soap			Compound, shared	Pit latrine without slab	Pit latrine v
o, dedicatec	Liquid soap		Open spaces/bush			No facility/b
powder soa	Detergent/powder soap			Compound, not sharec	Pit latrine with concret	Flush/pour
powder soa	Detergent/powder soap			Compound, not sharec	Pit latrine with concret	Pit latrine v
o, dedicatec	Liquid soap			Compound, shared	Flush to pit latrine	Flush/pour
o, dedicatec	Liquid soap			Compound, shared	Flush to septic tank	Flush/pour
powder soa	Detergent/powder soap			Compound, not sharec	Flush to donâ€™t kno	Flush/pour
powder soa	Detergent/powder soap			Compound, not sharec	Pit latrine with concret	Pit latrine v
sed	Other (spe	Do not use soap		In the household	Flush to septic tank	Flush/pour
powder soa	Detergent/powder soap			Compound, not sharec	Flush to septic tank	Flush/pour
	Bar soap			Compound, not sharec	Pit latrine with concret	Pit latrine v
o, also for o	Bar soap			Compound, shared	Flush to pit latrine	Flush/pour
or all house	Liquid soap			Compound, shared	Pit latrine with concret	Pit latrine v
o, dedicatec	Liquid soap			Compound, shared	Flush to septic tank	Flush/pour
or all house	Bar soap			Neighboring compoun	Pit latrine with concret	Pit latrine v
powder soa	Detergent/powder soa		Open spaces/bush			No facility/b
	Bar soap			Compound, shared	Pit latrine with concret	Pit latrine v
same as wh	Liquid soap			Compound, shared	Flush to pit latrine	Flush/pour
same as wh	Bar soap			In the household	Pit latrine with concret	Pit latrine v
o, dedicatec	Liquid soap			In the household	Flush to septic tank	Flush/pour
o, dedicatec	Liquid soap			Compound, not sharec	Flush to septic tank	Flush/pour
ap, same a:	Liquid soap			Compound, shared	Flush to pit latrine	Flush/pour
powder soa	Detergent/powder soap			Compound, not sharec	Flush to septic tank	Flush/pour

same as wh	Bar soap		Compound, shared	Pit latrine with concret	Pit latrine v
same as wh	Bar soap		Compound, not share	Flush to piped sewer s	Flush/pour
dedicated o	Liquid soap		Compound, shared	Pit latrine with concret	Pit latrine v
same as wh	Bar soap		In the household	Flush to septic tank	Flush/pour
same as wh	Bar soap	Use toilet at work			No facility/b
ap, same a	Bathing soap		Compound, shared	Flush to septic tank	Flush/pour
same as wh	Bar soap		Compound, shared	Pit latrine with concret	Pit latrine v
), dedicatec	Liquid soap		In the household	Pit latrine with concret	Pit latrine v
dedicated o	Bar soap		In the household	Flush to septic tank	Flush/pour
ap, same a	Bar soap		Compound, shared	Flush to pit latrine	Flush/pour
dedicated o	Bar soap		In the household	Flush to septic tank	Flush/pour
dedicated o	Bar soap		Compound, not share	Flush to septic tank	Flush/pour
ap, same a	Bar soap		Compound, shared	Flush to pit latrine	Flush/pour
ap, same a	Bar soap		Compound, shared	Pit latrine with concret	Pit latrine v
powder soa	Detergent/powder soap		Compound, not share	Flush to pit latrine	Flush/pour
or all house	Bar soap		Compound, shared	Pit latrine with concret	Pit latrine v
), dedicatec	Liquid soap		Compound, not share	Flush to septic tank	Flush/pour
	Bar soap		Compound, shared	Flush to donâ€™t kno	Flush to do
same as wh	Bar soap		Compound, shared	Flush to piped sewer s	Flush/pour
), dedicatec	Liquid soap		In the household	Flush to pit latrine	Flush/pour
), dedicatec	Liquid soap		Compound, not share	Flush to septic tank	Flush/pour
same as wh	Bar soap		Compound, shared	Flush to piped sewer s	Flush/pour
), dedicatec	Liquid soap		Compound, not share	Pit latrine without slab	Pit latrine v
powder soa	Detergent/powder soap		Neighboring compoun	Pit latrine without slab	Pit latrine v
), dedicatec	Liquid soap		In the household	Flush to septic tank	Flush/pour
dedicated o	Liquid soap		Compound, shared	Pit latrine with concret	Pit latrine v
same as wh	Bar soap		Compound, shared	Pit latrine with concret	Pit latrine v
or all house	Bar soap	Open spaces/bush			No facility/b
same as wh	Liquid soap		Compound, shared	Pit latrine with concret	Pit latrine v
same as wh	Bar soap		Compound, shared	Pit latrine with concret	Pit latrine v
dedicated o	Bar soap		In the household	Flush to septic tank	Flush/pour
), dedicatec	Liquid soap		Compound, not share	Flush to septic tank	Flush/pour
ap, same a	Bar soap		Compound, shared	Flush to piped sewer s	Flush/pour
same as wh	Bar soap		Compound, shared	Pit latrine with concret	Pit latrine v
dedicated o	Bar soap		Compound, shared	Flush to piped sewer s	Flush/pour
powder soa	Detergent/powder soap		Compound, not share	Pit latrine with concret	Pit latrine v
), dedicatec	Liquid soap	Open spaces/bush			No facility/b
powder soa	Detergent/powder soap		Compound, shared	Flush to pit latrine	Flush/pour
), also for o	Liquid soap		Compound, not share	Flush to pit latrine	Flush/pour
), dedicatec	Liquid soap		Compound, shared	Flush to piped sewer s	Flush/pour
ap, same a	Bar soap		Compound, shared	Flush to septic tank	Flush/pour
), dedicatec	Liquid soap		Compound, not share	Pit latrine without slab	Pit latrine v
dedicated o	Bar soap		In the household	Flush to septic tank	Flush/pour
ap, same a	Bar soap		Compound, shared	Flush to septic tank	Flush/pour
ap, same a	Bathing soap		Compound, shared	Flush to pit latrine	Flush/pour
ap, same a	Liquid soap		Compound, shared	Flush to pit latrine	Flush/pour
), dedicatec	Liquid soap		Compound, shared	Flush to piped sewer s	Flush/pour
powder soa	Detergent/powder soap		Compound, shared	Flush to septic tank	Flush/pour
ap, same a	Bathing soap		Compound, shared	Pit latrine with concret	Pit latrine v

	Detergent/powder soa	Neighbouring toilet/compound			Flush/pour
same as wh	Bar soap		In the household	Flush to septic tank	Flush/pour
), also for o	Liquid soap		Compound, shared	Flush to pit latrine	Flush/pour
ap, same a:	Bar soap		Compound, shared	Pit latrine with concret	Pit latrine v
powder soa	Detergent/powder soap		Compound, shared	Flush to septic tank	Flush/pour
same as wh	Bar soap		Compound, shared	Flush to septic tank	Flush/pour
), dedicatec	Liquid soap		In the household	Pit latrine without slab	Pit latrine v
	Bar soap	Neighbouring toilet/compound			Flush/pour
ap, same a:	Bar soap		Compound, not sharec	Flush to septic tank	Flush/pour
same as wh	Bar soap		In the household	Pit latrine with concret	Pit latrine v
ap, same a:	Bar soap		Compound, shared	Pit latrine with concret	Pit latrine v
powder soa	Liquid soap		Compound, not sharec	Flush to piped sewer s	Flush/pour
same as wh	Bar soap		Compound, shared	Pit latrine with concret	Pit latrine v
), dedicatec	Liquid soap		Compound, not sharec	Flush to pit latrine	Flush/pour
), dedicatec	Liquid soap		Compound, shared	Flush to piped sewer s	Flush/pour
dedicated o	Bar soap		Compound, shared	Flush to piped sewer s	Flush/pour
), dedicatec	Liquid soap		Compound, not sharec	Flush to septic tank	Flush/pour
ap, same a:	Bar soap		Compound, shared	Flush to pit latrine	Flush/pour
same as wh	Bar soap		In the household	Pit latrine with concret	Pit latrine v
ap, same a:	Bar soap		Compound, shared	Pit latrine with concret	Pit latrine v
), dedicatec	Liquid soap		Compound, shared	Pit latrine with concret	Pit latrine v
or all house	Bar soap		Compound, shared	Pit latrine with concret	Pit latrine v
same as wh	Bar soap		In the household	Flush to septic tank	Flush/pour
ap, same a:	Bar soap		In the household	Flush to donâ€™t kno	Flush to do
ap, same a:	Liquid soap		Compound, shared	Flush to pit latrine	Flush/pour
), dedicatec	Liquid soap		In the household	Pit latrine with concret	Pit latrine v
same as wh	Bar soap		Compound, shared	Flush to pit latrine	Flush/pour
dedicated o	Bar soap		In the household	Flush to piped sewer s	Flush/pour
dedicated o	Bar soap		Neighboring compoun	Pit latrine with concret	Pit latrine v
ap, same a:	Bar soap		Compound, shared	Pit latrine with concret	Pit latrine v
same as wh	Bar soap		Compound, shared	Pit latrine with concret	Pit latrine v
powder soa	Detergent/powder soap		Compound, not sharec	Flush to septic tank	Flush/pour
same as wh	Bar soap		Compound, shared	Flush to piped sewer s	Flush/pour
dedicated o	Bar soap		In the household	Flush to septic tank	Flush/pour
powder soa	Detergent/powder soap		Compound, shared	Flush to pit latrine	Flush/pour
same as wh	Bar soap		Compound, not sharec	Pit latrine with concret	Pit latrine v
	Liquid soap		Compound, shared	Flush to piped sewer s	Flush/pour
), dedicatec	Liquid soap		Compound, shared	Flush to septic tank	Flush/pour
), dedicatec	Liquid soap		Compound, shared	Flush to pit latrine	Flush/pour
), dedicatec	Liquid soap		In the household	Flush to pit latrine	Flush/pour
), dedicatec	Liquid soap		Compound, shared	Flush to septic tank	Flush/pour
powder soa	Detergent/powder soap		Compound, not sharec	Pit latrine with concret	Pit latrine v
ap, same a:	Bar soap		Compound, shared	Pit latrine with concret	Pit latrine v
), dedicatec	Liquid soap	Neighbouring toilet/compound			Flush/pour
), dedicatec	Liquid soap		Compound, not sharec	Flush to septic tank	Flush/pour
same as wh	Bar soap		Compound, shared	Pit latrine with concret	Pit latrine v
ap, same a:	Liquid soap		Compound, shared	Flush to piped sewer s	Flush/pour
ap, same a:	Liquid soap		Compound, shared	Pit latrine with concret	Pit latrine v
	Bar soap		Compound, shared	Pit latrine with concret	Pit latrine v

same as wh	Bar soap	Compound, shared	Flush to septic tank	Flush/pour
ap, same a	Bar soap	Neighboring compoun	Pit latrine with concret	Pit latrine w
o, dedicatec	Liquid soap	Compound, shared	Flush to piped sewer s	Flush/pour
powder soa	Detergent/powder soap	Compound, shared	Flush to septic tank	Flush/pour
same as wh	Bar soap	Compound, shared	Pit latrine with concret	Pit latrine w
or all house	Bar soap	Compound, shared	Pit latrine with concret	Pit latrine w
dedicated o	Bar soap	Compound, shared	Pit latrine with concret	Pit latrine w
powder soa	Detergent/powder soap	Compound, shared	Pit latrine with concret	Pit latrine w
dedicated o	Bar soap	Neighboring compoun	Flush to septic tank	Flush/pour
or all house	Bar soap	Compound, shared	Pit latrine with concret	Pit latrine w
	Bathing soap	Neighboring compoun	Flush to piped sewer s	Flush/pour
same as wh	Bar soap	Compound, not sharec	Flush to septic tank	Flush/pour
same as wh	Bar soap	Compound, shared	Pit latrine with concret	Pit latrine w
powder soa	Detergent/powder soap	Compound, not sharec	Flush to septic tank	Flush/pour
dedicated o	Bar soap	Compound, shared	Pit latrine with concret	Pit latrine w
same as wh	Bar soap	Compound, shared	Flush to piped sewer s	Flush/pour
same as wh	Bar soap	In the household	Flush to septic tank	Flush/pour
o, dedicatec	Liquid soap	Compound, shared	Flush to piped sewer s	Flush/pour
same as wh	Bar soap	Compound, shared	Flush to piped sewer s	Flush/pour
ap, same a	Bar soap	Compound, shared	Pit latrine with concret	Pit latrine w
powder soa	Detergent/powder soap	Compound, not sharec	Flush to pit latrine	Flush/pour
o, also for o	Liquid soap	Compound, shared	Flush to pit latrine	Flush/pour
same as wh	Bar soap	Compound, shared	Pit latrine with concret	Pit latrine w
o, dedicatec	Liquid soap	Compound, not sharec	Pit latrine without slab	Pit latrine w
same as wh	Bar soap	Compound, shared	Pit latrine with concret	Pit latrine w
ap, same a	Bathing soap	Compound, shared	Flush to piped sewer s	Flush/pour
same as wh	Liquid soap	Compound, shared	Pit latrine with concret	Pit latrine w
same as wh	Bar soap	Compound, shared	Flush to septic tank	Flush/pour
same as wh	Bar soap	In the household	Flush to septic tank	Flush/pour
same as wh	Liquid soap	Compound, shared	Flush to piped sewer s	Flush/pour
ap, same a	Bathing soap	In the household	Flush to pit latrine	Flush/pour
o, dedicatec	Liquid soap	Compound, not sharec	Pit latrine with concret	Pit latrine w
ap, same a	Liquid soap	Compound, shared	Flush to piped sewer s	Flush/pour
same as wh	Bar soap	Compound, shared	Pit latrine with concret	Pit latrine w
o, dedicatec	Liquid soap	Compound, not sharec	Flush to septic tank	Flush/pour
o, dedicatec	Liquid soap	Compound, not sharec	Flush to septic tank	Flush/pour
ap, same a	Liquid soap	Neighboring compoun	Flush to pit latrine	Flush/pour
same as wh	Bar soap	Compound, shared	Flush to pit latrine	Flush/pour
ap, same a	Liquid soap	Compound, shared	Pit latrine with concret	Pit latrine w
ap, same a	Bar soap	Neighboring compoun	Pit latrine without slab	Pit latrine w
same as wh	Bar soap	In the household	Flush to septic tank	Flush/pour
same as wh	Bar soap	Compound, shared	Flush to septic tank	Flush/pour
ap, same a	Liquid soap	In the household	Flush to septic tank	Flush/pour
powder soa	Detergent/powder soap	Compound, not sharec	Pit latrine with concret	Pit latrine w
or all house	Bar soap	Compound, shared	Pit latrine with concret	Pit latrine w
ap, same a	Liquid soap	Compound, shared	Flush to donâ€™t kno	Flush to do
powder soa	Detergent/powder soap	Compound, not sharec	Pit latrine with concret	Pit latrine w
o, dedicatec	Liquid soap	Compound, shared	Pit latrine with concret	Pit latrine w
ap, same a	Bar soap	Compound, shared	Pit latrine with concret	Pit latrine w

ap, same a	Bathing soap	Compound, shared	Flush to pit latrine	Flush/pour
), also for o	Liquid soap	Compound, shared	Pit latrine with concret	Pit latrine v
same as wh	Bar soap	Compound, shared	Flush to pit latrine	Flush/pour
same as wh	Liquid soap	Compound, shared	Flush to donâ€™t kno	Flush to do
dedicated o	Bar soap	In the household	Flush to septic tank	Flush/pour
ap, same a	Bar soap	Compound, shared	Pit latrine with concret	Pit latrine v
ap, same a	Liquid soap	Compound, shared	Pit latrine with concret	Pit latrine v
ap, same a	Bar soap	Compound, shared	Pit latrine with concret	Pit latrine v
powder soa	Liquid soap	In the household	Flush to piped sewer s	Flush/pour
), also for o	Liquid soap	Compound, shared	Flush to pit latrine	Flush/pour
dedicated o	Bar soap	Compound, shared	Pit latrine with concret	Pit latrine v
), dedicatec	Liquid soap	Compound, not sharec	Flush to septic tank	Flush/pour
same as wh	Bar soap	Neighbouring toilet/compound		Flush/pour
same as wh	Bar soap	Compound, shared	Pit latrine without slab	Pit latrine v
), dedicatec	Liquid soap	Compound, shared	Pit latrine without slab	Pit latrine v
same as wh	Bar soap	Neighboring compoun	Pit latrine with concret	Pit latrine v
), dedicatec	Liquid soap	Compound, shared	Flush to septic tank	Flush/pour
same as wh	Bar soap	Compound, shared	Flush to piped sewer s	Flush/pour
same as wh	Bar soap	Compound, shared	Pit latrine with concret	Pit latrine v
or all house	Bar soap	Compound, shared	Pit latrine with concret	Pit latrine v
same as wh	Bar soap	Compound, shared	Pit latrine with concret	Pit latrine v
ap, same a	Bar soap	Compound, shared	Flush to piped sewer s	Flush/pour
or all house	Bar soap	Compound, shared	Flush to piped sewer s	Flush/pour
same as wh	Bar soap	Compound, shared	Flush to pit latrine	Flush/pour
), also for o	Liquid soap	Compound, shared	Flush to septic tank	Flush/pour
ap, same a	Bar soap	Compound, shared	Flush to piped sewer s	Flush/pour
), dedicatec	Liquid soap	In the household	Flush to septic tank	Flush/pour
), dedicatec	Liquid soap	Compound, not sharec	Flush to pit latrine	Flush/pour
same as wh	Bar soap	Neighbouring toilet/compound		Flush/pour
same as wh	Bar soap	Compound, shared	Pit latrine with concret	Pit latrine v
), dedicatec	Liquid soap	Compound, shared	Flush to septic tank	Flush/pour
or all house	Bathing soap	Compound, shared	Flush to piped sewer s	Flush/pour
ap, same a	Bar soap	Compound, shared	Pit latrine with concret	Pit latrine v
), dedicatec	Liquid soap	In the household	Flush to septic tank	Flush/pour
ap, same a	Bar soap	Compound, shared	Flush to septic tank	Flush/pour
same as wh	Bar soap	Compound, shared	Pit latrine with concret	Pit latrine v
same as wh	Bar soap	Neighboring compoun	Flush to piped sewer s	Flush/pour
same as wh	Bar soap	Compound, shared	Pit latrine with concret	Pit latrine v
dedicated o	Bar soap	Compound, shared	Flush to pit latrine	Flush/pour
same as wh	Bar soap	Compound, shared	Pit latrine with concret	Pit latrine v
or all house	Liquid soap	Compound, shared	Pit latrine with concret	Pit latrine v
dedicated o	Bathing soap	Neighboring compoun	Pit latrine with concret	Pit latrine v
same as wh	Bar soap	Compound, shared	Pit latrine with concret	Pit latrine v
or all house	Bar soap	Compound, shared	Flush to septic tank	Flush/pour
), dedicatec	Liquid soap	Compound, shared	Flush to septic tank	Flush/pour
or all house	Bar soap	Compound, shared	Flush to piped sewer s	Flush/pour
or all house	Bar soap	Compound, shared	Flush to piped sewer s	Flush/pour
ap, same a	Bar soap	Compound, shared	Pit latrine with concret	Pit latrine v
ap, same a	Bar soap	Compound, shared	Pit latrine with concret	Pit latrine v

), dedicatec	Liquid soap	In the household	Flush to septic tank	Flush/pour
same as wh	Bar soap	Compound, shared	Flush to septic tank	Flush/pour
or all house	Bar soap	Compound, shared	Flush to piped sewer s	Flush/pour
same as wh	Bar soap	Compound, shared	Flush to piped sewer s	Flush/pour
ap, same a	Liquid soap	In the household	Flush to piped sewer s	Flush/pour
same as wh	Bar soap	Compound, shared	Pit latrine with concret	Pit latrine v
), dedicatec	Liquid soap	In the household	Flush to piped sewer s	Flush/pour
), dedicatec	Liquid soap	In the household	Flush to septic tank	Flush/pour
or all house	Other (spe any that is available s	Compound, shared	Flush to piped sewer s	Flush/pour
ap, same a	Bathing soap	Compound, shared	Flush to donâ€™t kno	Flush to do
ap, same a	Bathing soap	Neighboring compoun	Flush to piped sewer s	Flush/pour
), dedicatec	Liquid soap	Compound, shared	Pit latrine with concret	Pit latrine v
), dedicatec	Liquid soap	In the household	Flush to septic tank	Flush/pour
powder soa	Detergent/powder soap	Compound, shared	Pit latrine with concret	Pit latrine v
dedicated o	Liquid soap	Compound, shared	Flush to piped sewer s	Flush/pour
ap, same a	Liquid soap	Compound, shared	Flush to piped sewer s	Flush/pour
dedicated o	Liquid soap	Compound, shared	Flush to septic tank	Flush/pour
), dedicatec	Liquid soap	Compound, shared	Flush to pit latrine	Flush/pour
ap, same a	Bar soap	Compound, shared	Pit latrine with concret	Pit latrine v
same as wh	Bar soap	Compound, shared	Pit latrine with concret	Pit latrine v
dedicated o	Bar soap	Compound, shared	Pit latrine with concret	Pit latrine v
or all house	Bar soap	Compound, shared	Flush to septic tank	Flush/pour
same as wh	Bar soap	Compound, shared	Pit latrine with concret	Pit latrine v
ap, same a	Bar soap	Compound, shared	Pit latrine with concret	Pit latrine v
same as wh	Bar soap	Compound, shared	Pit latrine with concret	Pit latrine v
ap, same a	Bar soap	Compound, shared	Flush to piped sewer s	Flush/pour
same as wh	Bar soap	Compound, shared	Pit latrine with concret	Pit latrine v
ap, same a	Bar soap	Compound, shared	Flush to septic tank	Flush/pour
same as wh	Bar soap	Compound, shared	Pit latrine without slab	Pit latrine v
ap, same a	Bar soap	Compound, shared	Pit latrine with concret	Pit latrine v
same as wh	Bathing soap	In the household	Flush to piped sewer s	Flush/pour
), dedicatec	Liquid soap	Compound, shared	Pit latrine with concret	Pit latrine v
same as wh	Bar soap	Compound, shared	Pit latrine without slab	Pit latrine v
), dedicatec	Liquid soap	Compound, shared	Pit latrine with concret	Pit latrine v
ap, same a	Bar soap	Compound, shared	Pit latrine with concret	Pit latrine v
or all house	Bar soap	Compound, shared	Pit latrine with concret	Pit latrine v
same as wh	Bathing soap	Compound, shared	Pit latrine with concret	Pit latrine v
same as wh	Bar soap	Compound, shared	Flush to septic tank	Flush/pour
dedicated o	Bar soap	Compound, shared	Flush to piped sewer s	Flush/pour
dedicated o	Bar soap	In the household	Flush to septic tank	Flush/pour
ap, same a	Bar soap	Neighboring compoun	Pit latrine without slab	Pit latrine v
same as wh	Bar soap	Compound, shared	Flush to pit latrine	Flush/pour
ap, same a	Liquid soap	Community latrine	Pit latrine with concret	Pit latrine v
or all house	Bar soap	Compound, shared	Flush to piped sewer s	Flush/pour
ap, same a	Bathing soap	Compound, shared	Other(Spe Flush/pour	Flush/pour
ap, same a	Bar soap	Compound, shared	Pit latrine with concret	Pit latrine v
ap, same a	Liquid soap	Compound, shared	Pit latrine with concret	Pit latrine v
), dedicatec	Liquid soap	Compound, shared	Flush to septic tank	Flush/pour
or all house	Bar soap	Compound, shared	Flush to piped sewer s	Flush/pour

ledicated o	Bar soap	Compound, shared	Flush to piped sewer s	Flush/pour
ap, same a	Bar soap	Compound, shared	Flush to piped sewer s	Flush/pour
ap, same a	Bar soap	Compound, shared	Flush to piped sewer s	Flush/pour
same as wh	Bar soap	Compound, shared	Pit latrine with concret	Pit latrine v
same as wh	Bar soap	Compound, shared	Pit latrine with concret	Pit latrine v
ap, same a	Bar soap	Neighboring compoun	Pit latrine with concret	Pit latrine v
same as wh	Bar soap	Compound, shared	Pit latrine with concret	Pit latrine v
ledicated o	Bar soap	Compound, shared	Flush to piped sewer s	Flush/pour
ap, same a	Bar soap	Compound, shared	Flush to pit latrine	Flush/pour
same as wh	Bar soap	Compound, not share	Pit latrine without slab	Pit latrine v
same as wh	Bar soap	Compound, shared	Flush to septic tank	Flush/pour
), dedicatec	Liquid soap	Compound, shared	Flush to septic tank	Flush/pour
), dedicatec	Liquid soap	Neighboring compoun	No facility	No facility/b
same as wh	Bar soap	Compound, shared	Pit latrine with concret	Pit latrine v
same as wh	Bar soap	Compound, shared	Flush to pit latrine	Flush/pour
ledicated o	Bar soap	Compound, shared	Flush to pit latrine	Flush/pour
), dedicatec	Liquid soap	Compound, not share	Pit latrine with concret	Pit latrine v
same as wh	Bar soap	Compound, shared	Pit latrine without slab	Pit latrine v
), dedicatec	Liquid soap	Compound, not share	Flush to pit latrine	Flush/pour
), dedicatec	Liquid soap	Compound, not share	Flush to pit latrine	Flush/pour
same as wh	Bar soap	Neighboring compoun	Pit latrine with concret	Pit latrine v
), dedicatec	Detergent/powder soap	Compound, shared	Pit latrine with concret	Pit latrine v
powder soa	Detergent/powder soap	In the household	Flush to septic tank	Flush/pour
ledicated o	Liquid soap	Compound, not share	Pit latrine with concret	Pit latrine v
ap, same a	Bar soap	Compound, shared	Pit latrine with concret	Pit latrine v
or all house	Bar soap	Compound, shared	Pit latrine with concret	Pit latrine v
), dedicatec	Liquid soap	In the household	Flush to septic tank	Flush/pour
ap, same a	Bar soap	Compound, shared	Pit latrine with concret	Pit latrine v
ledicated o	Bar soap	Compound, shared	Pit latrine with concret	Pit latrine v
ap, same a	Bathing soap	Compound, shared	Pit latrine with concret	Pit latrine v
same as wh	Bar soap	Compound, shared	Pit latrine with concret	Pit latrine v
ledicated o	Bar soap	Compound, shared	Pit latrine with concret	Pit latrine v
ledicated o	Bar soap	Compound, shared	Flush to pit latrine	Flush/pour
same as wh	Bar soap	Compound, shared	Flush to pit latrine	Flush/pour
same as wh	Bar soap	Compound, shared	Flush to septic tank	Flush/pour
powder soa	Detergent/powder soap	Compound, shared	Flush to septic tank	Flush/pour
same as wh	Bar soap	Neighboring compoun	Pit latrine without slab	Compostin
ap, same a	Bar soap	Compound, shared	Pit latrine with concret	Pit latrine v
same as wh	Bar soap	Compound, not share	Flush to septic tank	Flush/pour
ap, same a	Bar soap	Compound, shared	Flush to piped sewer s	Flush/pour
), dedicatec	Liquid soap	In the household	Flush to septic tank	Flush/pour
), dedicatec	Liquid soap	Compound, shared	Flush to septic tank	Flush/pour
), also for o	Liquid soap	Compound, not share	Flush to pit latrine	Flush/pour
same as wh	Bar soap	Compound, shared	Pit latrine with concret	Pit latrine v
ap, same a	Bar soap	Compound, shared	Flush to piped sewer s	Flush/pour
ap, same a	Bathing soap	Compound, shared	Flush to piped sewer s	Flush/pour
or all house	Bar soap	Neighboring compoun	Flush to donâ€™t kno	Flush to do
), dedicatec	Liquid soap	In the household	Flush to septic tank	Flush/pour
same as wh	Bar soap	Compound, shared	Flush to piped sewer s	Flush/pour

same as wh	Liquid soap	Compound, shared	Flush to piped sewer s	Flush/pour
), dedicatec	Liquid soap	Compound, shared	Pit latrine with concret	Pit latrine v
ap, same a	Bathing soap	Compound, shared	Flush to piped sewer s	Flush/pour
same as wh	Bar soap	Compound, shared	Pit latrine with concret	Pit latrine v
or all house	Bar soap	In the household	Flush to piped sewer s	Flush/pour
), dedicatec	Liquid soap	In the household	Flush to septic tank	Flush/pour
), dedicatec	Liquid soap	Compound, not sharec	Flush to piped sewer s	Flush/pour
), dedicatec	Liquid soap	Compound, shared	Flush to septic tank	Flush/pour
), dedicatec	Bar soap	Compound, shared	Flush to piped sewer s	Flush/pour
same as wh	Bar soap	Compound, shared	Pit latrine with concret	Pit latrine v
same as wh	Bar soap	Compound, shared	Pit latrine with concret	Pit latrine v
same as wh	Bar soap	Compound, shared	Pit latrine with concret	Pit latrine v
), dedicatec	Liquid soap	Compound, shared	Pit latrine with concret	Pit latrine v
powder soa	Detergent/powder soap	In the household	Pit latrine with concret	Pit latrine v
ap, same a	Bar soap	Compound, shared	Pit latrine with concret	Pit latrine v
same as wh	Bar soap	Compound, shared	Pit latrine with concret	Pit latrine v
), dedicatec	Liquid soap	In the household	Flush to septic tank	Flush/pour
same as wh	Bar soap	In the household	Flush to piped sewer s	Flush/pour
same as wh	Bar soap	Compound, shared	Pit latrine with concret	Pit latrine v
ap, same a	Bar soap	Compound, shared	Flush to pit latrine	Flush/pour
same as wh	Bar soap	Compound, shared	Flush to piped sewer s	Flush/pour
ap, same a	Bar soap	Compound, shared	Flush to septic tank	Flush/pour
dedicated o	Bar soap	Compound, shared	Flush to septic tank	Flush/pour
), dedicatec	Liquid soap	Compound, shared	Pit latrine with concret	Pit latrine v
same as wh	Bar soap	Compound, shared	Pit latrine with concret	Pit latrine v
same as wh	Bar soap	Compound, shared	Pit latrine with concret	Pit latrine v
), dedicatec	Liquid soap	Compound, not sharec	Pit latrine with concret	Pit latrine v
ap, same a	Bar soap	Compound, shared	Pit latrine with concret	Pit latrine v
dedicated o	Bar soap	Compound, shared	Flush to piped sewer s	Flush/pour
dedicated o	Bar soap	Compound, shared	Flush to septic tank	Flush/pour
same as wh	Bar soap	Compound, shared	Pit latrine with concret	Pit latrine v
dedicated o	Bar soap	Compound, shared	Flush to piped sewer s	Flush/pour
or all house	Bar soap	Neighboring compoun	Flush to piped sewer s	Flush/pour
ap, same a	Bathing soap	Compound, shared	Flush to piped sewer s	Flush/pour
), dedicatec	Liquid soap	In the household	Flush to septic tank	Flush/pour
dedicated o	Bar soap	Compound, shared	Pit latrine with concret	Pit latrine v
or all house	Bar soap	Compound, shared	Flush to pit latrine	Flush/pour
dedicated o	Bar soap	Compound, shared	Pit latrine with concret	Pit latrine v
ap, same a	Bar soap	Compound, shared	Flush to piped sewer s	Flush/pour
same as wh	Bar soap	Compound, shared	Pit latrine with concret	Pit latrine v
), dedicatec	Liquid soap	In the household	Flush to septic tank	Flush/pour
same as wh	Bar soap	Compound, shared	Pit latrine with concret	Pit latrine v
ap, same a	Bar soap	Compound, shared	Flush to piped sewer s	Flush/pour
ap, same a	Bar soap	Compound, shared	Flush to septic tank	Flush/pour
same as wh	Bar soap	Compound, shared	Pit latrine with concret	Pit latrine v
), dedicatec	Liquid soap	In the household	Flush to septic tank	Flush/pour
same as wh	Detergent/powder soap	Compound, shared	Flush to septic tank	Other (Spe
), dedicatec	Liquid soap	In the household	Flush to piped sewer s	Flush/pour
), dedicatec	Liquid soap	Compound, shared	Flush to piped sewer s	Flush/pour

same as wh	Bar soap	Compound, shared	Pit latrine with concret	Pit latrine v
dedicated o	Bar soap	Compound, shared	Pit latrine with concret	Pit latrine v
same as wh	Bar soap	Compound, shared	Flush to piped sewer s	Flush/pour
or all house	Liquid soap	Compound, shared	Pit latrine with concret	Pit latrine v
o, dedicatec	Bar soap	Compound, shared	Flush to pit latrine	Flush/pour
powder soa	Detergent/powder soap	Compound, shared	Flush to septic tank	Flush/pour
same as wh	Bar soap	Compound, shared	Pit latrine with concret	Pit latrine v
ap, same a:	Bar soap	Compound, shared	Pit latrine with concret	Pit latrine v
o, dedicatec	Liquid soap	Compound, not sharec	Flush to septic tank	Flush/pour
dedicated o	Bar soap	Compound, shared	Flush to pit latrine	Flush/pour
dedicated o	Bar soap	In the household	Pit latrine with concret	Pit latrine v
ap, same a:	Bar soap	Neighboring compoun	Pit latrine with concret	Pit latrine v
powder soa	Detergent/powder soap	Compound, shared	Flush to septic tank	Flush/pour
same as wh	Bar soap	Compound, shared	Pit latrine with concret	Pit latrine v
o, dedicatec	Liquid soap	In the household	Flush to piped sewer s	Flush/pour
or all house	Bar soap	Compound, shared	Flush to piped sewer s	Flush/pour
ap, same a:	Bar soap	Compound, shared	Pit latrine without slab	Pit latrine v
dedicated o	Liquid soap	Compound, not sharec	Pit latrine with concret	Pit latrine v
o, dedicatec	Liquid soap	In the household	Flush to pit latrine	Flush/pour
o, dedicatec	Liquid soap	Compound, shared	Flush to pit latrine	Flush/pour
same as wh	Bar soap	Compound, shared	Flush to piped sewer s	Flush/pour
o, dedicatec	Liquid soap	Neighbouring toilet/compound		Pit latrine v
same as wh	Bar soap	Compound, shared	Flush to donâ€™t kno	Flush to do
same as wh	Bar soap	Compound, shared	Pit latrine with concret	Pit latrine v
ap, same a:	Bar soap	Compound, shared	Pit latrine with concret	Pit latrine v
same as wh	Bar soap	Compound, shared	Pit latrine with concret	Pit latrine v
o, dedicatec	Liquid soap	In the household	Flush to septic tank	Flush/pour
o, dedicatec	Liquid soap	Compound, shared	Flush to septic tank	Flush/pour
ap, same a:	Bar soap	Compound, not sharec	Pit latrine without slab	Pit latrine v
dedicated o	Bar soap	In the household	Flush to piped sewer s	Flush/pour
or all house	Bar soap	Compound, shared	Pit latrine with concret	Pit latrine v
same as wh	Liquid soap	In the household	Flush to piped sewer s	Flush/pour
o, dedicatec	Liquid soap	Compound, shared	Flush to piped sewer s	Flush/pour
same as wh	Bar soap	Neighbouring toilet/compound		Pit latrine v
dedicated o	Bar soap	Compound, shared	Pit latrine with concret	Pit latrine v
o, dedicatec	Liquid soap	Compound, shared	Flush to septic tank	Flush/pour
o, dedicatec	Liquid soap	Compound, shared	Flush to septic tank	Flush/pour
dedicated o	Bar soap	Compound, shared	Flush to piped sewer s	Flush/pour
same as wh	Bar soap	Compound, not sharec	Pit latrine with concret	Pit latrine v
same as wh	Bar soap	Compound, not sharec	Pit latrine with concret	Pit latrine v
same as wh	Bar soap	Compound, shared	Pit latrine with concret	Pit latrine v
powder soa	Detergent/powder soap	Compound, not sharec	Flush to septic tank	Flush/pour
same as wh	Bar soap	Compound, shared	Pit latrine with concret	Pit latrine v
dedicated o	Bar soap	Compound, shared	Pit latrine with concret	Pit latrine v
same as wh	Bar soap	Compound, shared	Pit latrine with concret	Pit latrine v
powder soa	Detergent/powder soap	In the household	Flush to septic tank	Flush/pour
same as wh	Bar soap	Compound, shared	Pit latrine with concret	Pit latrine v
same as wh	Bar soap	Compound, shared	Pit latrine with concret	Pit latrine v
same as wh	Bar soap	Compound, shared	Pit latrine with concret	Pit latrine v

ɔ, dedicatec	Liquid soap		In the household	Flush to piped sewer s	Flush/pour
ap, same a	Bathing soap		Compound, shared	Flush to septic tank	Flush/pour
ɔ, dedicatec	Liquid soap		Compound, not sharec	Pit latrine with concret	Pit latrine v
ap, same a	Bar soap		Compound, shared	Pit latrine with concret	Pit latrine v
dedicated o	Bar soap		Neighboring compoun	Flush to piped sewer s	Flush/pour
same as wh	Bar soap		Compound, shared	Flush to septic tank	Flush/pour
same as wh	Bar soap		Compound, shared	Flush to septic tank	Flush/pour
or all house	Bar soap		Compound, shared	Flush to piped sewer s	Flush/pour
ɔ, dedicatec	Liquid soap		Compound, shared	Pit latrine with concret	Pit latrine v
ɔ, dedicatec	Liquid soap		Compound, shared	Pit latrine with concret	Pit latrine v
or all house	Liquid soap		Compound, shared	Pit latrine with concret	Pit latrine v
or all house	Bar soap		Community latrine	Pit latrine with concret	Pit latrine v
ɔ, dedicatec	Liquid soap		In the household	Flush to piped sewer s	Flush/pour
same as wh	Bar soap		Compound, shared	Flush to piped sewer s	Flush/pour
ap, same a	Bar soap		Compound, shared	Pit latrine with concret	Pit latrine v
ap, same a	Bar soap		Compound, shared	Flush to piped sewer s	Flush/pour
dedicated o	Bar soap		In the household	Flush to piped sewer s	Flush/pour
ap, same a	Bathing soap		Compound, shared	Pit latrine with concret	Pit latrine v
ɔ, dedicatec	Liquid soap		Compound, shared	Pit latrine with concret	Pit latrine v
ɔ, dedicatec	Liquid soap		Compound, shared	Flush to pit latrine	Flush/pour
same as wh	Bar soap		Compound, shared	Pit latrine with concret	Pit latrine v
dedicated o	Liquid soap		In the household	Flush to septic tank	Flush/pour
ɔ, also for o	Liquid soap	Open spaces/bush			No facility/b
same as wh	Bar soap		Compound, shared	Pit latrine with concret	Pit latrine v
same as wh	Bar soap		Compound, shared	Pit latrine without slab	Pit latrine v
ɔ, dedicatec	Liquid soap	Open spaces/bush			No facility/b
ɔ, also for o	Liquid soap		Compound, shared	Pit latrine with concret	Pit latrine v
ap, same a	Bar soap		Compound, shared	Pit latrine with concret	Pit latrine v
same as wh	Bar soap		Compound, shared	Flush to pit latrine	Flush/pour
ɔ, dedicatec	Liquid soap		Compound, not sharec	Flush to pit latrine	Flush/pour
or all house	Bar soap		Compound, shared	Pit latrine with concret	Pit latrine v
ɔ, dedicatec	Liquid soap		In the household	Flush to septic tank	Flush/pour
same as wh	Bar soap		Compound, shared	Pit latrine with concret	Pit latrine v
same as wh	Bar soap		In the household	Flush to septic tank	Flush/pour
same as wh	Bar soap		Neighboring compoun	Pit latrine with concret	Pit latrine v
dedicated o	Bar soap		In the household	Flush to piped sewer s	Flush/pour
ap, same a	Bar soap		Compound, shared	Flush to piped sewer s	Flush/pour
or all house	Bar soap		Compound, not sharec	Pit latrine with concret	Pit latrine v
same as wh	Bar soap		Compound, shared	Flush to piped sewer s	Flush/pour
same as wh	Bar soap		Public latrine	Pit latrine with concret	Pit latrine v
dedicated o	Liquid soap		In the household	Flush to septic tank	Flush/pour
ap, same a	Liquid soap		In the household	Flush to piped sewer s	Flush/pour
ɔ, dedicatec	Liquid soap		Compound, shared	Flush to pit latrine	Flush/pour
same as wh	Bar soap		Compound, shared	Flush to piped sewer s	Flush/pour
ɔ, dedicatec	Liquid soap		In the household	Pit latrine with concret	Pit latrine v
same as wh	Bar soap		Compound, shared	Pit latrine with concret	Pit latrine v
dedicated o	Bar soap		Compound, shared	Flush to piped sewer s	Flush/pour
or all house	Bathing soap		Compound, shared	Flush to piped sewer s	Flush/pour
ap, same a	Bar soap		Compound, shared	Pit latrine with concret	Pit latrine v

or all house	Bar soap	Compound, shared	Flush to piped sewer s	Flush/pour
same as wh	Bar soap	Compound, shared	Flush to piped sewer s	Flush/pour
same as wh	Bar soap	Neighboring compoun	Pit latrine with concret	Pit latrine v
o, dedicatec	Liquid soap	Compound, shared	Flush to septic tank	Flush/pour
o, dedicatec	Liquid soap	Neighbouring toilet/compound		Pit latrine v
same as wh	Bar soap	In the household	Flush to septic tank	Flush/pour
o, dedicatec	Liquid soap	Compound, not sharec	Flush to pit latrine	Flush/pour
dedicated o	Bar soap	Compound, shared	Flush to septic tank	Flush/pour
same as wh	Bar soap	Compound, shared	Pit latrine with concret	Pit latrine v
ap, same a	Bar soap	Compound, shared	Flush to pit latrine	Flush/pour
o, dedicatec	Liquid soap	Compound, shared	Flush to septic tank	Flush/pour
ap, same a	Bar soap	Compound, shared	Flush to piped sewer s	Flush/pour
ap, same a	Bar soap	Compound, shared	Flush to piped sewer s	Flush/pour
dedicated o	Liquid soap	Neighboring compoun	Flush to piped sewer s	Flush/pour
dedicated o	Liquid soap	Compound, shared	Pit latrine with concret	Pit latrine v
same as wh	Bar soap	Compound, shared	Pit latrine with concret	Pit latrine v
o, dedicatec	Liquid soap	Compound, shared	Pit latrine with concret	Pit latrine v
same as wh	Bar soap	Neighbouring toilet/compound		Pit latrine v
or all house	Bar soap	Compound, shared	Flush to pit latrine	Flush/pour
dedicated o	Liquid soap	Compound, shared	Pit latrine with concret	Pit latrine v
o, dedicatec	Liquid soap	Compound, not sharec	Pit latrine with concret	Pit latrine v
ap, same a	Bar soap	Compound, shared	Flush to septic tank	Flush/pour
ap, same a	Bar soap	Compound, shared	Flush to piped sewer s	Flush/pour
ap, same a	Bar soap	In the household	Flush to piped sewer s	Flush/pour
same as wh	Liquid soap	Compound, shared	Flush to piped sewer s	Flush/pour
same as wh	Liquid soap	In the household	Flush to septic tank	Flush/pour
or all house	Bar soap	Compound, shared	Flush to piped sewer s	Flush/pour
o, dedicatec	Liquid soap	Compound, shared	Pit latrine with concret	Pit latrine v
o, dedicatec	Bar soap	Compound, shared	Pit latrine with concret	Pit latrine v
same as wh	Bar soap	Compound, shared	Pit latrine with concret	Pit latrine v
same as wh	Bar soap	Neighboring compoun	Pit latrine with concret	Pit latrine v
o, dedicatec	Liquid soap	Compound, shared	Pit latrine with concret	Pit latrine v
same as wh	Bar soap	Compound, not sharec	Pit latrine without slab	Pit latrine v
powder soa	Detergent/powder soa	Neighbouring toilet/compound		Flush/pour
same as wh	Liquid soap	Compound, shared	Flush to septic tank	Flush/pour
o, dedicatec	Liquid soap	Compound, shared	Flush to septic tank	Flush/pour
same as wh	Bar soap	Compound, shared	Pit latrine with concret	Pit latrine v
dedicated o	Bar soap	Compound, shared	Flush to septic tank	Flush/pour
or all house	Bar soap	Compound, shared	Flush to piped sewer s	Flush/pour
ap, same a	Bathing soap	Compound, shared	Pit latrine with concret	Pit latrine v
o, dedicatec	Liquid soap	In the household	Flush to septic tank	Flush/pour
ap, same a	Bar soap	Compound, shared	Pit latrine with concret	Pit latrine v
ap, same a	Bar soap	Compound, shared	Flush to pit latrine	Flush/pour
same as wh	Bar soap	In the household	Flush to piped sewer s	Flush/pour
ap, same a	Bar soap	Compound, shared	Pit latrine with concret	Pit latrine v
same as wh	Bar soap	Compound, shared	Pit latrine with concret	Pit latrine v
same as wh	Bar soap	Compound, shared	Flush to pit latrine	Flush/pour
o, dedicatec	Liquid soap	In the household	Flush to septic tank	Flush/pour
same as wh	Bar soap	In the household	Flush to septic tank	Flush/pour

same as wh	Bar soap	Compound, shared	Flush to piped sewer s		Flush/pour
ap, same a	Bar soap	Compound, shared	Flush to septic tank		Flush/pour
), dedicatec	Liquid soap	Compound, not share	Flush to septic tank		Flush/pour
same as wh	Bar soap	Compound, shared	Pit latrine with concret		Pit latrine w
same as wh	Bar soap	Compound, shared	Pit latrine without slab		Pit latrine w
same as wh	Bar soap	Compound, shared	Pit latrine with concret		Pit latrine w
ap, same a	Bar soap	Compound, shared	Flush to pit latrine		Flush/pour
or all house	Liquid soap	Compound, not share	Flush to donâ€™t kno		Flush to do
same as wh	Bar soap	Community latrine	Pit latrine with concret		Pit latrine w
same as wh	Bathing soap	Compound, shared	Flush to piped sewer s		Flush/pour
ap, same a	Bar soap	Compound, shared	Pit latrine with concret		Pit latrine w
or all house	Bar soap	Compound, shared	Pit latrine with concret		Pit latrine w
), dedicatec	Bar soap	In the household	Flush to septic tank		Flush/pour
ap, same a	Bar soap	In the household	Flush to septic tank		Flush/pour
same as wh	Bar soap	Community latrine	Flush to septic tank		Flush/pour
same as wh	Bar soap	Compound, shared	Pit latrine with concret		Pit latrine w
ap, same a	Bar soap	Compound, shared	Flush to pit latrine		Flush/pour
ap, same a	Bar soap	Compound, not share	Pit latrine with concret		Pit latrine w
or all house	Bar soap	Compound, shared	Flush to piped sewer s		Flush/pour
dedicated o	Bar soap	Compound, shared	Flush to pit latrine		Flush/pour
ap, same a	Bar soap	Compound, shared	Pit latrine with concret		Pit latrine w
same as wh	Bar soap	Compound, shared	Pit latrine with concret		Pit latrine w
), dedicatec	Liquid soap	Compound, not share	Flush to septic tank		Flush/pour
ap, same a	Bar soap	In the household	Flush to piped sewer s		Flush/pour
ap, same a	Liquid soap	Compound, shared	Flush to piped sewer s		Flush/pour
same as wh	Liquid soap	Compound, shared	Flush to piped sewer s		Flush/pour
dedicated o	Bar soap	In the household	Flush to septic tank		Flush/pour
dedicated o	Bar soap	Compound, shared	Flush to piped sewer s		Flush/pour
same as wh	Bar soap	Compound, shared	Flush to piped sewer s		Flush/pour
), dedicatec	Liquid soap	Compound, shared	Flush to piped sewer s		Flush/pour
dedicated o	Bar soap	Compound, shared	Flush to piped sewer s		Flush/pour
dedicated o	Bar soap	In the household	Flush to piped sewer s		Flush/pour
ap, same a	Bar soap	In the household	Flush to piped sewer s		Flush/pour
or all house	Liquid soap	Compound, shared	Other(Spe	Flush/pour	Other (Spe
ap, same a	Bar soap	Compound, not share	Flush to pit latrine		Flush/pour
), dedicatec	Liquid soap	Community latrine	Flush to piped sewer s		Flush/pour
or all house	Liquid soap	Compound, shared	Flush to pit latrine		Flush/pour
ap, same a	Bar soap	Compound, shared	Pit latrine with concret		Pit latrine w
), dedicatec	Liquid soap	In the household	Flush to septic tank		Flush/pour
ap, same a	Bathing soap	Compound, shared	Pit latrine with concret		Pit latrine w
ap, same a	Bathing soap	In the household	Flush to septic tank		Flush/pour
), dedicatec	Liquid soap	In the household	Flush to septic tank		Flush/pour
same as wh	Bar soap	Compound, shared	Pit latrine with concret		Pit latrine w
ap, same a	Liquid soap	In the household	Flush to septic tank		Flush/pour
), dedicatec	Liquid soap	Compound, shared	Flush to piped sewer s		Flush/pour
ap, same a	Liquid soap	Compound, shared	Flush to pit latrine		Flush to do
dedicated o	Liquid soap	Compound, not share	Pit latrine with concret		Pit latrine w
or all house	Bar soap	Community latrine	Pit latrine with concret		Pit latrine w
or all house	Bar soap	Compound, shared	Flush to piped sewer s		Flush/pour

dedicated o	Bar soap	Compound, shared	Flush to piped sewer s	Flush/pour
dedicated o	Bar soap	Compound, shared	Flush to septic tank	Flush/pour
o, dedicatec	Liquid soap	Compound, shared	Pit latrine with concret	Pit latrine w
ap, same a	Bar soap	Compound, shared	Flush to septic tank	Flush/pour
o, dedicatec	Liquid soap	In the household	Flush to septic tank	Flush/pour
o, dedicatec	Liquid soap	Compound, not sharec	Pit latrine without slab	Pit latrine w
same as wh	Bar soap	Compound, shared	Pit latrine with concret	Pit latrine w
ap, same a	Bar soap	Compound, shared	Pit latrine with concret	Pit latrine w
or all house	Detergent/powder soap	Compound, shared	Pit latrine with concret	Pit latrine w
ap, same a	Bar soap	Neighboring compoun	Pit latrine with concret	Pit latrine w
same as wh	Liquid soap	In the household	Flush to septic tank	Flush/pour
o, dedicatec	Liquid soap	Neighbouring toilet/compound		Pit latrine w
dedicated o	Liquid soap	In the household	Pit latrine with concret	Pit latrine w
ap, same a	Bar soap	Compound, shared	Pit latrine with concret	Pit latrine w
ap, same a	Bathing soap	Community latrine	Pit latrine with concret	Pit latrine w
same as wh	Bar soap	Compound, shared	Pit latrine with concret	Pit latrine w
powder soa	Detergent/powder soap	Compound, shared	Flush to septic tank	Flush/pour
same as wh	Bar soap	Compound, shared	Flush to septic tank	Flush/pour
same as wh	Bar soap	Compound, shared	Flush to piped sewer s	Flush/pour
same as wh	Bar soap	Compound, shared	Pit latrine with concret	Pit latrine w
powder soa	Liquid soap	Neighbouring toilet/compound		Flush to do
ap, same a	Bar soap	Compound, shared	Pit latrine with concret	Pit latrine w
or all house	Bar soap	Compound, shared	Pit latrine with concret	Pit latrine w
ap, same a	Bathing soap	Compound, shared	Pit latrine with concret	Pit latrine w
dedicated o	Bar soap	Compound, shared	Flush to piped sewer s	Flush/pour
same as wh	Bar soap	Compound, shared	Flush to septic tank	Flush/pour
ap, same a	Bar soap	Compound, shared	Flush to pit latrine	Flush/pour
or all house	Bar soap	Compound, not sharec	Flush to pit latrine	Flush/pour
same as wh	Bar soap	Compound, shared	Flush to pit latrine	Flush/pour
same as wh	Bar soap	Compound, shared	Pit latrine with concret	Pit latrine w
powder soa	Detergent/powder soap	Compound, shared	Pit latrine with concret	Pit latrine w
same as wh	Bar soap	Compound, shared	Pit latrine with concret	Pit latrine w
powder soa	Bar soap	Compound, shared	Flush to piped sewer s	Flush/pour
o, dedicatec	Liquid soap	Compound, shared	Pit latrine with concret	Pit latrine w
o, dedicatec	Bar soap	Compound, shared	Flush to pit latrine	Flush/pour
o, dedicatec	Liquid soap	In the household	Flush to septic tank	Flush/pour
ap, same a	Bar soap	Compound, shared	Pit latrine with concret	Pit latrine w
ap, same a	Bar soap	Compound, shared	Pit latrine with concret	Pit latrine w
or all house	Bar soap	Compound, shared	Flush to piped sewer s	Flush/pour
o, dedicatec	Liquid soap	Compound, shared	Pit latrine with concret	Pit latrine w
ap, same a	Bar soap	Compound, shared	Flush to pit latrine	Flush/pour
same as wh	Bar soap	Compound, not sharec	Pit latrine with concret	Pit latrine w
dedicated o	Bar soap	Compound, shared	Pit latrine with concret	Pit latrine w
o, dedicatec	Liquid soap	Compound, shared	Pit latrine with concret	Pit latrine w
dedicated o	Liquid soap	Compound, shared	Flush to piped sewer s	Flush/pour
o, dedicatec	Liquid soap	Compound, not sharec	Flush to septic tank	Flush/pour
same as wh	Bar soap	Compound, shared	Pit latrine with concret	Pit latrine w
same as wh	Bar soap	Compound, shared	Pit latrine with concret	Pit latrine w
or all house	Bar soap	Compound, shared	Flush to septic tank	Flush/pour

same as wh	Bar soap	Compound, shared	Pit latrine without slab	Pit latrine w
powder soa	Detergent/powder soap	Compound, not sharec	Pit latrine with concret	Pit latrine w
ap, same a	Bar soap	Neighboring compoun	Pit latrine with concret	Pit latrine w
or all house	Bar soap	Compound, shared	Flush to piped sewer s	Flush/pour
ap, same a	Bar soap	Compound, shared	Pit latrine with concret	Pit latrine w
same as wh	Bar soap	Compound, not sharec	Pit latrine without slab	Pit latrine w
o, dedicatec	Liquid soap	Compound, shared	Flush to septic tank	Flush/pour
o, dedicatec	Liquid soap	Compound, shared	Flush to piped sewer s	Flush/pour
ap, same a	Bar soap	Compound, not sharec	Flush to piped sewer s	Flush/pour
same as wh	Bar soap	Compound, shared	Pit latrine with concret	Pit latrine w
ap, same a	Bathing soap	Compound, shared	Flush to septic tank	Flush/pour
same as wh	Bar soap	Compound, shared	Pit latrine with concret	Ventilated i
dedicated o	Bar soap	Compound, shared	Flush to piped sewer s	Flush/pour
ap, same a	Bar soap	Compound, shared	Other(Spe Pit latrine w	Other (Spe
same as wh	Bar soap	In the household	Flush to donâ€™t kno	Flush to do
ap, same a	Bar soap	Compound, shared	Flush to piped sewer s	Flush/pour
ap, same a	Bar soap	Compound, shared	Flush to septic tank	Flush/pour
same as wh	Bar soap	Compound, shared	Flush to donâ€™t kno	Pit latrine w
powder soa	Detergent/powder soap	Compound, shared	Pit latrine with concret	Pit latrine w
o, dedicatec	Liquid soap	Compound, shared	Pit latrine with concret	Pit latrine w
same as wh	Bar soap	Compound, shared	Pit latrine with concret	Pit latrine w
dedicated o	Bar soap	Compound, shared	Flush to piped sewer s	Flush/pour
powder soa	Detergent/powder soap	Compound, shared	Bag	Flush/pour
ap, same a	Bar soap	Compound, shared	Pit latrine with concret	Pit latrine w
ap, same a	Bar soap	Compound, shared	Pit latrine with concret	Pit latrine w
same as wh	Bar soap	Neighboring compoun	Pit latrine with concret	Pit latrine w
o, dedicatec	Liquid soap	Compound, not sharec	Flush to septic tank	Flush/pour
o, dedicatec	Liquid soap	Compound, shared	Flush to septic tank	Flush/pour
ap, same a	Bar soap	Compound, shared	Flush to pit latrine	Flush/pour
or all house	Bar soap	Compound, shared	Flush to piped sewer s	Flush/pour
same as wh	Bar soap	Compound, shared	Pit latrine with concret	Pit latrine w
o, dedicatec	Liquid soap	Compound, shared	Flush to pit latrine	Flush/pour
dedicated o	Bar soap	Compound, shared	Flush to piped sewer s	Flush/pour
dedicated o	Bar soap	Compound, not sharec	Flush to pit latrine	Flush/pour
o, dedicatec	Bar soap	Compound, shared	Pit latrine with concret	Pit latrine w
ap, same a	Liquid soap	Compound, shared	Pit latrine with concret	Pit latrine w
o, dedicatec	Liquid soap	In the household	Flush to piped sewer s	Flush/pour
same as wh	Bar soap	Compound, shared	Flush to piped sewer s	Flush/pour
same as wh	Bar soap	Neighboring compoun	Pit latrine with concret	Pit latrine w
or all house	Bar soap	Compound, shared	Flush to piped sewer s	Flush/pour
o, dedicatec	Liquid soap	Compound, shared	Pit latrine with concret	Pit latrine w
or all house	Bar soap	Compound, shared	Flush to septic tank	Flush/pour
o, also for o	Liquid soap	Compound, shared	Pit latrine with concret	Pit latrine w
ap, same a	Bar soap	Compound, shared	Flush to piped sewer s	Flush/pour
same as wh	Bar soap	Neighboring compoun	Flush to piped sewer s	Flush/pour
powder soa	Detergent/powder soap	Compound, shared	Flush to septic tank	Flush/pour
dedicated o	Liquid soap	Compound, not sharec	Pit latrine with concret	Pit latrine w
same as wh	Bar soap	Compound, shared	Pit latrine with concret	Pit latrine w
o, dedicatec	Liquid soap	Compound, shared	Flush to septic tank	Flush/pour

or all house	Bar soap	Compound, shared	Flush to piped sewer s	Flush/pour
dedicated o	Bar soap	Neighboring compoun	Pit latrine with concret	Pit latrine w
o, dedicatec	Liquid soap	Compound, shared	Flush to piped sewer s	Flush/pour
same as wh	Bar soap	Compound, shared	Flush to septic tank	Flush/pour
same as wh	Bar soap	Compound, shared	Pit latrine with concret	Pit latrine w
same as wh	Bar soap	Compound, shared	Pit latrine with concret	Pit latrine w
o, dedicatec	Liquid soap	Compound, shared	Flush to pit latrine	Flush/pour
same as wh	Bar soap	Compound, shared	Pit latrine with concret	Pit latrine w
same as wh	Bathing soap	Compound, shared	Flush to septic tank	Flush/pour
same as wh	Bar soap	Compound, shared	Pit latrine without slab	Pit latrine w
same as wh	Bar soap	Compound, shared	Pit latrine with concret	Pit latrine w
ap, same a	Liquid soap	Compound, shared	Flush to piped sewer s	Flush/pour
o, dedicatec	Liquid soap	Compound, not share	Flush to pit latrine	Flush/pour
ap, same a	Liquid soap	Compound, shared	Flush to piped sewer s	Flush/pour
or all house	Bar soap	Compound, shared	Flush to piped sewer s	Flush/pour
o, dedicatec	Liquid soap	Compound, shared	Pit latrine with concret	Pit latrine w
same as wh	Bar soap	Compound, shared	Flush to septic tank	Pit latrine w
same as wh	Bar soap	Compound, shared	Pit latrine with concret	Pit latrine w
o, dedicatec	Liquid soap	Compound, shared	Flush to septic tank	Flush/pour
o, dedicatec	Liquid soap	Compound, not share	Flush to septic tank	Flush/pour
ap, same a	Bar soap	Neighboring compoun	Flush to septic tank	Flush/pour
same as wh	Bar soap	Compound, not share	Flush to septic tank	Flush/pour
dedicated o	Liquid soap	Compound, shared	Flush to septic tank	Flush/pour
or all house	Bar soap	Compound, shared	Pit latrine with concret	Pit latrine w
ap, same a	Bar soap	Compound, shared	Flush to piped sewer s	Flush/pour
dedicated o	Bar soap	Compound, shared	Flush to piped sewer s	Flush/pour
same as wh	Bar soap	Compound, not share	Pit latrine with concret	Pit latrine w
o, dedicatec	Liquid soap	Compound, shared	Flush to pit latrine	Flush/pour
dedicated o	Bar soap	Compound, shared	Flush to piped sewer s	Flush/pour
dedicated o	Bar soap	In the household	Flush to piped sewer s	Flush/pour
dedicated o	Bar soap	Compound, shared	Flush to septic tank	Flush/pour
o, dedicatec	Liquid soap	Compound, shared	Pit latrine with concret	Pit latrine w
dedicated o	Bar soap	Compound, shared	Pit latrine without slab	Pit latrine w
dedicated o	Bar soap	In the household	Flush to septic tank	Flush/pour
ap, same a	Bar soap	Compound, shared	Flush to septic tank	Flush/pour
same as wh	Bar soap	Compound, shared	Flush to pit latrine	Flush/pour
same as wh	Bar soap	Compound, shared	Pit latrine with concret	Pit latrine w
o, also for o	Liquid soap	Compound, shared	Pit latrine with concret	Pit latrine w
or all house	Bar soap	Compound, shared	Flush to pit latrine	Flush/pour
or all house	Bar soap	Compound, shared	Flush to piped sewer s	Flush/pour
ap, same a	Bar soap	Neighboring compoun	Pit latrine with concret	Pit latrine w
o, dedicatec	Liquid soap	Compound, shared	Flush to septic tank	Flush/pour
powder soa	Detergent/powder soap	Compound, shared	Flush to septic tank	Flush/pour
o, dedicatec	Liquid soap	Compound, shared	Flush to septic tank	Flush/pour
ap, same a	Bathing soap	Compound, shared	Flush to piped sewer s	Flush/pour
dedicated o	Bar soap	Compound, shared	Pit latrine with concret	Pit latrine w
dedicated o	Bar soap	In the household	Flush to septic tank	Flush/pour
ap, same a	Bar soap	Neighboring compoun	Flush to piped sewer s	Flush/pour
same as wh	Bar soap	Neighbouring toilet/compound		Pit latrine w

same as wh	Bar soap	Compound, shared	Pit latrine with concret	Pit latrine w
), dedicatec	Liquid soap	Compound, not sharec	Pit latrine with concret	Pit latrine w
same as wh	Bar soap	Compound, shared	Pit latrine with concret	Pit latrine w
ap, same a:	Liquid soap	Compound, shared	Flush to septic tank	Flush/pour
), also for o	Liquid soap	Compound, shared	Flush to piped sewer s	Flush/pour
same as wh	Bar soap	Compound, shared	Pit latrine with concret	Pit latrine w
ap, same a:	Bar soap	Compound, shared	Pit latrine with concret	Pit latrine w
same as wh	Bar soap	Compound, shared	Flush to septic tank	Flush/pour
same as wh	Bar soap	Compound, shared	Pit latrine with concret	Pit latrine w
ap, same a:	Bar soap	Compound, shared	Pit latrine with concret	Pit latrine w
), dedicatec	Liquid soap	Compound, shared	Flush to pit latrine	Flush/pour
), dedicatec	Liquid soap	Compound, shared	Flush to pit latrine	Flush/pour
same as wh	Bar soap	Compound, shared	Pit latrine with concret	Pit latrine w
same as wh	Bar soap	Compound, shared	Flush to septic tank	Flush/pour
same as wh	Bar soap	Compound, shared	Pit latrine with concret	Pit latrine w
same as wh	Bar soap	Compound, shared	Pit latrine with concret	Pit latrine w
), dedicatec	Liquid soap	In the household	Flush to septic tank	Flush/pour
ap, same a:	Liquid soap	Compound, not sharec	Pit latrine with concret	Pit latrine w
same as wh	Bar soap	Compound, shared	Pit latrine with concret	Pit latrine w
or all house	Bar soap	Compound, shared	Flush to piped sewer s	Flush/pour
same as wh	Bar soap	Compound, shared	Pit latrine with concret	Pit latrine w
dedicated o	Bar soap	Compound, shared	Flush to piped sewer s	Flush/pour
), dedicatec	Liquid soap	Neighbouring toilet/compound		Pit latrine w
), dedicatec	Liquid soap	In the household	Flush to septic tank	Flush/pour
), dedicatec	Liquid soap	Compound, shared	Flush to septic tank	Flush/pour
), dedicatec	Liquid soap	Neighboring compoun	Pit latrine with concret	Pit latrine w
ap, same a:	Bathing soap	Compound, shared	Flush to piped sewer s	Flush/pour
ap, same a:	Bar soap	Compound, shared	Pit latrine with concret	Pit latrine w
same as wh	Bar soap	Compound, shared	Flush to piped sewer s	Flush/pour
same as wh	Bar soap	Compound, shared	Pit latrine with concret	Pit latrine w
ap, same a:	Bar soap	Compound, shared	Pit latrine with concret	Pit latrine w
same as wh	Bar soap	Compound, shared	Flush to septic tank	Flush/pour
), dedicatec	Liquid soap	Compound, shared	Flush to pit latrine	Flush/pour
or all house	Bar soap	Compound, shared	Flush to piped sewer s	Flush/pour
), dedicatec	Bar soap	Compound, shared	Pit latrine without slab	Pit latrine w
), dedicatec	Liquid soap	Compound, shared	Flush to piped sewer s	Flush/pour
same as wh	Bar soap	Compound, shared	Pit latrine with concret	Pit latrine w
), dedicatec	Liquid soap	Compound, shared	Flush to piped sewer s	Flush/pour
or all house	Bar soap	Compound, shared	Pit latrine with concret	Pit latrine w
ap, same a:	Bar soap	In the household	Flush to septic tank	Flush/pour
dedicated o	Bar soap	Compound, shared	Flush to pit latrine	Flush/pour
same as wh	Bar soap	Compound, shared	Pit latrine with concret	Pit latrine w
ap, same a:	Bathing soap	Compound, shared	Pit latrine with concret	Pit latrine w
ap, same a:	Bar soap	Compound, shared	Flush to piped sewer s	Flush/pour
ap, same a:	Liquid soap	In the household	Flush to septic tank	Flush/pour
), dedicatec	Liquid soap	Compound, shared	Pit latrine with concret	Pit latrine w
ap, same a:	Liquid soap	In the household	Flush to piped sewer s	Flush/pour
dedicated o	Bar soap	In the household	Flush to piped sewer s	Flush/pour
same as wh	Bar soap	Compound, shared	Pit latrine with concret	Pit latrine w

same as wh	Bar soap	Compound, shared	Pit latrine without slab	Pit latrine w
dedicated o	Bar soap	Compound, shared	Flush to piped sewer s	Flush/pour
, dedicatec	Liquid soap	Community latrine	Pit latrine with concret	Pit latrine w
ap, same a	Bar soap	Compound, shared	Flush to septic tank	Flush/pour
ap, same a	Liquid soap	Compound, not share	Flush to pit latrine	Flush/pour
, dedicatec	Detergent/powder soap	Compound, not share	Flush to pit latrine	Flush/pour
, dedicatec	Liquid soap	Compound, shared	Flush to piped sewer s	Flush/pour
, dedicatec	Liquid soap	Compound, shared	Flush to pit latrine	Flush/pour
ap, same a	Bar soap	Compound, shared	Pit latrine with concret	Pit latrine w
dedicated o	Bar soap	Compound, shared	Pit latrine with concret	Pit latrine w
ap, same a	Bar soap	Compound, shared	Flush to piped sewer s	Flush/pour
dedicated o	Bar soap	Compound, shared	Flush to piped sewer s	Flush/pour
dedicated o	Bar soap	Compound, shared	Flush to septic tank	Flush/pour
powder soa	Detergent/powder soap	In the household	Flush to septic tank	Flush/pour
dedicated o	Bar soap	Compound, shared	Pit latrine with concret	Pit latrine w
ap, same a	Liquid soap	Compound, shared	Pit latrine with concret	Pit latrine w
same as wh	Bar soap	Compound, shared	Flush to piped sewer s	Flush/pour
, dedicatec	Liquid soap	Neighboring compoun	Pit latrine with concret	Pit latrine w
ap, same a	Liquid soap	Compound, shared	Pit latrine with concret	Pit latrine w
ap, same a	Bar soap	Compound, shared	Flush to piped sewer s	Flush/pour
dedicated o	Bar soap	In the household	Flush to septic tank	Flush/pour
, dedicatec	Liquid soap	Compound, shared	Flush to piped sewer s	Flush/pour
, also for o	Liquid soap	Compound, shared	Flush to septic tank	Flush/pour
ap, same a	Bar soap	Compound, shared	Pit latrine with concret	Pit latrine w
same as wh	Bar soap	Compound, shared	Pit latrine with concret	Pit latrine w
same as wh	Bar soap	Compound, shared	Pit latrine with concret	Pit latrine w
dedicated o	Bar soap	Compound, shared	Flush to septic tank	Flush/pour
, dedicatec	Bar soap	Compound, shared	Flush to pit latrine	Flush/pour
same as wh	Bar soap	Compound, shared	Flush to septic tank	Flush/pour
powder soa	Detergent/powder soap	Compound, shared	Flush to septic tank	Flush/pour
ap, same a	Bar soap	Compound, shared	Flush to piped sewer s	Flush/pour
powder soa	Liquid soap	In the household	Flush to piped sewer s	Flush/pour
, dedicatec	Liquid soap	Compound, shared	Flush to pit latrine	Flush/pour
dedicated o	Bar soap	Compound, shared	Flush to piped sewer s	Flush/pour
powder soa	Detergent/powder soap	Compound, not share	Flush to pit latrine	Flush/pour
, dedicatec	Liquid soap	Compound, shared	Pit latrine with concret	Pit latrine w
same as wh	Liquid soap	In the household	Pit latrine without slab	Pit latrine w
, dedicatec	Bar soap	Compound, shared	Pit latrine with concret	Pit latrine w
ap, same a	Bar soap	Compound, shared	Pit latrine with concret	Pit latrine w
ap, same a	Bar soap	In the household	Flush to piped sewer s	Flush/pour
, dedicatec	Liquid soap	In the household	Flush to septic tank	Flush/pour
same as wh	Bar soap	Community latrine	Pit latrine with concret	Pit latrine w
, dedicatec	Liquid soap	Compound, shared	Flush to septic tank	Flush/pour
same as wh	Bar soap	Compound, shared	Flush to septic tank	Flush/pour
, dedicatec	Liquid soap	Compound, not share	Flush to piped sewer s	Flush/pour
same as wh	Bar soap	Compound, shared	Pit latrine with concret	Pit latrine w
, dedicatec	Liquid soap	In the household	Flush to piped sewer s	Flush/pour
same as wh	Bar soap	Compound, shared	Pit latrine with concret	Pit latrine w
ap, same a	Bar soap	Compound, shared	Flush to septic tank	Flush/pour

ledicated o	Bar soap		Neighboring compoun	Flush to piped sewer s	Flush/pour
same as wh	Bar soap		In the household	Flush to septic tank	Flush/pour
), dedicatec	Liquid soap		Compound, shared	Flush to pit latrine	Flush/pour
), dedicatec	Liquid soap		In the household	Flush to septic tank	Flush/pour
), dedicatec	Liquid soap		In the household	Flush to septic tank	Flush/pour
), dedicatec	Liquid soap		Compound, shared	Flush to donâ€™t kno	Flush to do
ledicated o	Bar soap		Public latrine	Flush to septic tank	Flush/pour
), dedicatec	Liquid soap		Compound, shared	Pit latrine with concret	Pit latrine v
same as wh	Bar soap		Compound, shared	Flush to septic tank	Flush/pour
ap, same a	Bar soap		Compound, not sharec	Pit latrine without slab	Pit latrine v
or all house	Bar soap		Compound, shared	Flush to septic tank	Flush/pour
same as wh	Bar soap		Compound, shared	Pit latrine with concret	Pit latrine v
), dedicatec	Liquid soap		In the household	Flush to piped sewer s	Flush/pour
ap, same a	Bathing soap		Compound, shared	Pit latrine with concret	Pit latrine v
or all house	Bar soap		Compound, shared	Flush to septic tank	Flush/pour
same as wh	Bar soap		Compound, shared	Pit latrine with concret	Pit latrine v
powder soa	Detergent/powder soap		Neighboring compoun	Flush to septic tank	Flush/pour
same as wh	Bar soap		Compound, shared	Pit latrine with concret	Pit latrine v
ap, same a	Bathing soap		Compound, shared	Flush to pit latrine	Flush/pour
ap, same a	Bar soap		Compound, shared	Pit latrine with concret	Pit latrine v
ledicated o	Bar soap		In the household	Flush to piped sewer s	Flush/pour
), dedicatec	Bar soap		Compound, shared	Pit latrine with concret	Pit latrine v
), dedicatec	Liquid soap		Compound, not sharec	Flush to septic tank	Flush/pour
powder soa	Other (spe	Any that is available	Compound, not sharec	Pit latrine with concret	Pit latrine v
same as wh	Bar soap		Neighboring compoun	Pit latrine with concret	Pit latrine v
ap, same a	Liquid soap		Compound, shared	Flush to piped sewer s	Pit latrine v
same as wh	Bar soap		Neighbouring toilet/compound		Flush/pour
or all house	Liquid soap		Compound, shared	Pit latrine with concret	Pit latrine v
same as wh	Bar soap		Compound, shared	Pit latrine with concret	Pit latrine v
ap, same a	Bar soap		Compound, shared	Pit latrine with concret	Pit latrine v
same as wh	Bar soap		Compound, shared	Pit latrine with concret	Pit latrine v
same as wh	Bar soap		Compound, shared	Pit latrine with concret	Pit latrine v
powder soa	Detergent/powder soap		Compound, shared	Flush to pit latrine	Flush/pour
same as wh	Bar soap		Compound, shared	Pit latrine with concret	Pit latrine v
same as wh	Bar soap		Compound, shared	Pit latrine with concret	Pit latrine v
same as wh	Bar soap		Compound, shared	Pit latrine with concret	Pit latrine v
), dedicatec	Liquid soap		In the household	Flush to septic tank	Flush/pour
ap, same a	Bar soap		Compound, shared	Flush to septic tank	Flush/pour
or all house	Bar soap		Compound, shared	Pit latrine with concret	Pit latrine v
ap, same a	Bar soap		Compound, shared	Other(Spe Responder	Other (Spe
same as wh	Bar soap		Compound, shared	Flush to septic tank	Flush/pour
powder soa	Detergent/powder soap		Compound, not sharec	Pit latrine with concret	Pit latrine v
same as wh	Bar soap		Compound, shared	Pit latrine with concret	Pit latrine v
or all house	Bar soap		Neighboring compoun	Pit latrine with concret	Pit latrine v
ap, same a	Bar soap		Compound, shared	Pit latrine with concret	Pit latrine v
same as wh	Bar soap		Compound, shared	Pit latrine with concret	Pit latrine v
), dedicatec	Liquid soap		Compound, not sharec	Flush to septic tank	Flush/pour
ledicated o	Liquid soap		Compound, shared	Flush to piped sewer s	Flush/pour
ledicated o	Liquid soap		Community latrine	Flush to piped sewer s	Flush/pour

ledicated o	Bar soap	Compound, shared	Pit latrine with concret	Pit latrine v
ap, same a	Bar soap	Compound, shared	Pit latrine with concret	Pit latrine v
o, dedicatec	Liquid soap	Compound, not sharec	Flush to septic tank	Flush/pour
or all house	Liquid soap	Compound, shared	Flush to septic tank	Flush/pour
ap, same a	Bar soap	Compound, shared	Pit latrine with concret	Pit latrine v
ledicated o	Bar soap	Compound, shared	Flush to piped sewer s	Flush/pour
oowder soa	Detergent/powder soap	Compound, shared	Flush to septic tank	Flush/pour
ledicated o	Bar soap	Compound, shared	Flush to piped sewer s	Flush/pour
same as wh	Bar soap	In the household	Flush to septic tank	Flush/pour
or all house	Bar soap	Compound, shared	Pit latrine with concret	Pit latrine v
o, dedicatec	Liquid soap	Compound, shared	Flush to septic tank	Flush/pour
same as wh	Bar soap	Compound, shared	Pit latrine with concret	Pit latrine v
ledicated o	Bar soap	Compound, shared	Pit latrine with concret	Pit latrine v
ledicated o	Bar soap	Compound, not sharec	Flush to pit latrine	Flush/pour
same as wh	Bar soap	Compound, shared	Pit latrine with concret	Pit latrine v
same as wh	Bar soap	Compound, shared	Pit latrine with concret	Pit latrine v
o, dedicatec	Liquid soap	In the household	Flush to piped sewer s	Flush/pour
ap, same a	Bathing soap	Compound, shared	Pit latrine with concret	Pit latrine v
o, dedicatec	Liquid soap	Compound, shared	Pit latrine with concret	Pit latrine v
ap, same a	Bar soap	Compound, shared	Pit latrine with concret	Pit latrine v
same as wh	Bar soap	Compound, shared	Pit latrine with concret	Pit latrine v
o, dedicatec	Liquid soap	Neighbouring toilet/compound		Pit latrine v
same as wh	Bar soap	Compound, shared	Pit latrine with concret	Pit latrine v
ap, same a	Bathing soap	In the household	Flush to septic tank	Flush/pour
o, dedicatec	Liquid soap	Compound, not sharec	Flush to pit latrine	Flush/pour
same as wh	Bar soap	Compound, shared	Pit latrine with concret	Pit latrine v
same as wh	Bar soap	Compound, shared	Pit latrine with concret	Pit latrine v
same as wh	Bar soap	Compound, shared	Flush to septic tank	Flush/pour
ap, same a	Bar soap	Compound, shared	Flush to piped sewer s	Flush/pour
ap, same a	Bar soap	Compound, shared	Pit latrine with concret	Pit latrine v
o, dedicatec	Liquid soap	Compound, not sharec	Flush to septic tank	Flush/pour
ap, same a	Bar soap	Compound, shared	Flush to septic tank	Flush/pour
ap, same a	Bar soap	Neighboring compoun	Pit latrine with concret	Pit latrine v
same as wh	Bar soap	Compound, not sharec	Pit latrine with concret	Pit latrine v
same as wh	Bar soap	Compound, shared	Pit latrine with concret	Pit latrine v
ap, same a	Bathing soap	Compound, shared	Flush to septic tank	Flush/pour
ledicated o	Bar soap	Compound, shared	Pit latrine with concret	Pit latrine v
same as wh	Bar soap	Compound, shared	Flush to pit latrine	Flush/pour
ap, same a	Bar soap	Compound, shared	Pit latrine with concret	Pit latrine v
ap, same a	Bar soap	Compound, shared	Flush to piped sewer s	Flush/pour
o, dedicatec	Liquid soap	Compound, shared	Pit latrine with concret	Pit latrine v
o, dedicatec	Liquid soap	Compound, not sharec	Pit latrine with concret	Pit latrine v
o, dedicatec	Bar soap	Compound, shared	Pit latrine with concret	Pit latrine v
same as wh	Bar soap	Compound, shared	Pit latrine with concret	Pit latrine v
or all house	Bar soap	Compound, shared	Pit latrine with concret	Pit latrine v
ap, same a	Bar soap	Compound, shared	Flush to septic tank	Flush/pour
same as wh	Bar soap	Compound, shared	Pit latrine with concret	Pit latrine v
ledicated o	Liquid soap	Compound, shared	Flush to piped sewer s	Flush/pour
same as wh	Bar soap	Compound, shared	Flush to piped sewer s	Flush/pour

ɔ, dedicatec	Liquid soap	Compound, shared	Pit latrine with concret	Pit latrine v
same as wh	Bar soap	Compound, shared	Pit latrine with concret	Pit latrine v
same as wh	Bar soap	Compound, not sharec	Pit latrine with concret	Pit latrine v
or all house	Bar soap	Compound, shared	Pit latrine with concret	Pit latrine v
same as wh	Bar soap	Compound, shared	Pit latrine with concret	Pit latrine v
same as wh	Bar soap	In the household	Pit latrine with concret	Pit latrine v
or all house	Bar soap	Compound, shared	Flush to piped sewer s	Flush/pour
same as wh	Bar soap	Compound, shared	Pit latrine with concret	Pit latrine v
dedicated o	Bar soap	Compound, shared	Flush to piped sewer s	Flush/pour
or all house	Bar soap	Compound, shared	Flush to piped sewer s	Flush/pour
ɔ, dedicatec	Liquid soap	Compound, not sharec	Flush to pit latrine	Flush/pour
or all house	Bar soap	Neighboring compoun	Flush to pit latrine	Flush/pour
or all house	Bar soap	Compound, shared	Flush to septic tank	Flush/pour
or all house	Bar soap	Compound, shared	Flush to piped sewer s	Flush/pour
same as wh	Bar soap	Neighboring compoun	Pit latrine with concret	Pit latrine v
same as wh	Bar soap	Compound, shared	Pit latrine with concret	Pit latrine v
same as wh	Bar soap	Compound, shared	Pit latrine with concret	Pit latrine v
same as wh	Bar soap	Neighboring compoun	Flush to septic tank	Flush/pour
dedicated o	Liquid soap	Neighboring compoun	Flush to piped sewer s	Flush/pour
ap, same a	Bathing soap	Compound, shared	Flush to pit latrine	Flush/pour
same as wh	Bar soap	Neighboring compoun	Pit latrine with concret	Pit latrine v
ɔ, dedicatec	Liquid soap	In the household	Flush to septic tank	Flush/pour
did not der	Bar soap	Compound, shared	Flush to piped sewer s	Flush/pour
powder soa	Detergent/powder soap	Neighboring compoun	Flush to open pit	Pit latrine v
same as wh	Bar soap	Compound, shared	Flush to piped sewer s	Flush/pour
ɔ, dedicatec	Liquid soap	Compound, shared	Flush to septic tank	Flush/pour
dedicated o	Bar soap	Compound, shared	Flush to piped sewer s	Flush/pour
ap, same a	Bar soap	Compound, shared	Pit latrine with concret	Pit latrine v
ap, same a	Bar soap	In the household	Flush to septic tank	Flush/pour
same as wh	Bar soap	Compound, shared	Flush to septic tank	Flush/pour
ɔ, dedicatec	Liquid soap	Compound, shared	Flush to septic tank	Flush/pour
or all house	Bar soap	Compound, shared	Flush to septic tank	Flush/pour
dedicated o	Other (spe Sanitizer	Compound, shared	Pit latrine with concret	Pit latrine v
same as wh	Bar soap	Compound, shared	Flush to pit latrine	Flush/pour
or all house	Bar soap	Compound, shared	Pit latrine with concret	Pit latrine v
dedicated o	Bar soap	Compound, shared	Flush to septic tank	Flush/pour
or all house	Liquid soap	Compound, shared	Flush to septic tank	Flush/pour
ap, same a	Bar soap	Compound, shared	Flush to piped sewer s	Flush/pour
ap, same a	Bathing soap	Compound, shared	Pit latrine with concret	Pit latrine v
ɔ, dedicatec	Liquid soap	Compound, not sharec	Pit latrine with concret	Pit latrine v
same as wh	Liquid soap	Compound, shared	Flush to septic tank	Flush/pour
ɔ, dedicatec	Liquid soap	Compound, shared	Flush to septic tank	Flush/pour
ɔ, dedicatec	Liquid soap	Compound, shared	Flush to septic tank	Flush/pour
ap, same a	Bar soap	Compound, shared	Pit latrine with concret	Pit latrine v
same as wh	Bar soap	Compound, shared	Flush to septic tank	Flush/pour
dedicated o	Bar soap	Compound, shared	Pit latrine with concret	Pit latrine v
ap, same a	Bathing soap	Compound, not sharec	Flush to septic tank	Flush/pour
ap, same a	Bar soap	Compound, shared	Flush to septic tank	Flush/pour
ap, same a	Bar soap	Compound, shared	Flush to piped sewer s	Flush/pour

dedicated o	Bar soap	In the household	Flush to piped sewer s	Flush/pour
dedicated o	Bar soap	Compound, shared	Flush to donâ€™t kno	Flush to do
same as wh	Bar soap	Compound, shared	Flush to septic tank	Flush/pour
same as wh	Bar soap	Compound, shared	Pit latrine with concret	Pit latrine w
same as wh	Liquid soap	Compound, shared	Flush to piped sewer s	Flush/pour
same as wh	Bar soap	Compound, shared	Pit latrine with concret	Pit latrine w
dedicated o	Bar soap	Compound, shared	Pit latrine with concret	Pit latrine w
or all house	Bar soap	Compound, shared	Flush to piped sewer s	Flush/pour
o, dedicated	Bar soap	Compound, shared	Pit latrine with concret	Pit latrine w
ap, same as	Bar soap	Compound, shared	Pit latrine without slab	Pit latrine w
or all house	Bar soap	Compound, shared	Pit latrine with concret	Pit latrine w
o, dedicated	Liquid soap	Neighboring compoun	Pit latrine with concret	Pit latrine w
o, dedicated	Liquid soap	Compound, shared	Flush to septic tank	Flush/pour
powder soa	Detergent/powder soap	Compound, shared	Flush to pit latrine	Flush/pour
dedicated o	Bar soap	Compound, shared	Flush to septic tank	Flush/pour
same as wh	Bar soap	Compound, shared	Pit latrine with concret	Pit latrine w
o, dedicated	Liquid soap	Compound, shared	Pit latrine with concret	Pit latrine w
same as wh	Liquid soap	Compound, shared	Pit latrine with concret	Pit latrine w
o, dedicated	Liquid soap	Compound, shared	Flush to septic tank	Flush/pour
o, dedicated	Liquid soap	Compound, not share	Flush to septic tank	Flush/pour
dedicated o	Bar soap	Compound, shared	Flush to piped sewer s	Flush/pour
o, dedicated	Liquid soap	Compound, not share	Flush to septic tank	Flush/pour
same as wh	Bar soap	In the household	Flush to piped sewer s	Flush/pour
ap, same as	Bar soap	In the household	Flush to piped sewer s	Flush/pour
or all house	Bar soap	Compound, shared	Flush to piped sewer s	Flush/pour
ap, same as	Bathing soap	Compound, not share	Flush to septic tank	Flush/pour
o, dedicated	Liquid soap	In the household	Flush to piped sewer s	Flush/pour
same as wh	Bar soap	Compound, shared	Flush to piped sewer s	Flush/pour
ap, same as	Bar soap	Compound, shared	Flush to piped sewer s	Flush/pour
o, dedicated	Liquid soap	Compound, not share	Flush to septic tank	Flush/pour
dedicated o	Liquid soap	Compound, shared	Pit latrine with concret	Pit latrine w
or all house	Bar soap	Compound, shared	Flush to piped sewer s	Flush/pour
powder soa	Detergent/powder soap	Compound, shared	Flush to septic tank	Flush/pour
same as wh	Bar soap	Compound, shared	Pit latrine with concret	Pit latrine w
o, dedicated	Liquid soap	Open spaces/bush		No facility/b
or all house	Bar soap	In the household	Flush to donâ€™t kno	Flush to do
ap, same as	Bar soap	Compound, shared	Pit latrine with concret	Pit latrine w
o, dedicated	Liquid soap	Compound, shared	Flush to pit latrine	Flush/pour
powder soa	Detergent/powder soap	Compound, not share	Pit latrine with concret	Pit latrine w
ap, same as	Bar soap	Community latrine	Pit latrine with concret	Pit latrine w
o, dedicated	Liquid soap	Compound, shared	Pit latrine with concret	Pit latrine w
o, dedicated	Liquid soap	Compound, shared	Flush to septic tank	Flush/pour
or all house	Bar soap	Compound, shared	Pit latrine with concret	Pit latrine w
or all house	Bar soap	Compound, shared	Pit latrine with concret	Pit latrine w
ap, same as	Bar soap	In the household	Flush to septic tank	Flush/pour
ap, same as	Bar soap	In the household	Flush to piped sewer s	Flush/pour
dedicated o	Bar soap	Compound, shared	Flush to piped sewer s	Flush/pour
o, dedicated	Bar soap	Compound, shared	Pit latrine with concret	Pit latrine w
same as wh	Bar soap	Compound, shared	Pit latrine with concret	Pit latrine w

ɔ, dedicatec	Liquid soap	Compound, shared	Flush to septic tank	Flush/pour
ɔr all house	Bar soap	Compound, shared	Flush to piped sewer s	Flush/pour
ɔr all house	Liquid soap	Compound, shared	Flush to piped sewer s	Flush/pour
ɔr all house	Bar soap	Compound, shared	Flush to piped sewer s	Flush/pour
same as wh	Bar soap	Compound, not share	Pit latrine without slab	Pit latrine v
dedicated o	Bar soap	In the household	Flush to septic tank	Flush/pour
same as wh	Bar soap	Compound, shared	Pit latrine with concret	Pit latrine v
ɔ, dedicatec	Liquid soap	Compound, shared	Pit latrine with concret	Pit latrine v
powder soa	Bar soap	Compound, shared	Pit latrine with concret	Pit latrine v
ap, same a	Bar soap	Compound, not share	Pit latrine with concret	Pit latrine v
ɔ, dedicatec	Liquid soap	Compound, shared	Flush to septic tank	Flush/pour
same as wh	Bar soap	Compound, shared	Flush to piped sewer s	Flush/pour
ɔ, also for o	Liquid soap	Compound, not share	Flush to donâ€™t kno	Flush to do
ɔ, dedicatec	Liquid soap	Compound, shared	Flush to piped sewer s	Flush/pour
dedicated o	Bar soap	Compound, shared	Pit latrine with concret	Pit latrine v
dedicated o	Bar soap	Compound, shared	Flush to piped sewer s	Flush/pour
same as wh	Bar soap	Compound, shared	Flush to piped sewer s	Flush/pour
dedicated o	Liquid soap	In the household	Flush to piped sewer s	Flush/pour
dedicated o	Bar soap	Compound, shared	Flush to donâ€™t kno	Flush to do
powder soa	Detergent/powder soap	Compound, shared	Pit latrine with concret	Pit latrine v
ap, same a	Bar soap	Compound, not share	Pit latrine with concret	Pit latrine v
same as wh	Bar soap	Compound, shared	Flush to septic tank	Flush/pour
same as wh	Bar soap	Compound, shared	Pit latrine with concret	Pit latrine v
ɔ, dedicatec	Liquid soap	Prefer not to say		No facility/b
same as wh	Bar soap	Compound, shared	Flush to piped sewer s	Flush/pour
ɔr all house	Bar soap	Compound, shared	Flush to septic tank	Flush/pour
ap, same a	Bar soap	Compound, shared	Pit latrine with concret	Pit latrine v
same as wh	Bar soap	Compound, shared	Pit latrine with concret	Pit latrine v
same as wh	Bar soap	Compound, shared	Flush to pit latrine	Flush/pour
ap, same a	Bar soap	Neighboring compoun	Pit latrine without slab	Pit latrine v
dedicated o	Bar soap	Compound, shared	Flush to pit latrine	Flush/pour
ɔ, dedicatec	Liquid soap	Compound, not share	Flush to septic tank	Flush/pour
ɔr all house	Bar soap	Compound, shared	Flush to piped sewer s	Flush/pour
dedicated o	Bar soap	Compound, shared	Flush to piped sewer s	Flush/pour
same as wh	Bar soap	Neighboring compoun	Pit latrine without slab	Pit latrine v
ap, same a	Bar soap	Compound, shared	Flush to piped sewer s	Flush/pour
same as wh	Bar soap	Compound, shared	Pit latrine with concret	Pit latrine v
ɔ, dedicatec	Liquid soap	In the household	Flush to pit latrine	Flush/pour
same as wh	Bar soap	Compound, shared	Pit latrine with concret	Pit latrine v
same as wh	Bar soap	Compound, shared	Flush to pit latrine	Flush/pour
same as wh	Bar soap	Compound, shared	Pit latrine with concret	Pit latrine v
ɔr all house	Bar soap	Compound, shared	Pit latrine with concret	Pit latrine v
same as wh	Bar soap	Compound, shared	Pit latrine with concret	Pit latrine v
same as wh	Bar soap	Compound, not share	Pit latrine with concret	Pit latrine v
same as wh	Bar soap	Compound, shared	Flush to septic tank	Flush/pour
same as wh	Bar soap	Compound, shared	Flush to septic tank	Flush/pour
ɔ, dedicatec	Liquid soap	Compound, shared	Pit latrine with concret	Pit latrine v
ɔ, dedicatec	Liquid soap	Neighboring compoun	Pit latrine with concret	Pit latrine v
dedicated o	Liquid soap	In the household	Flush to piped sewer s	Flush/pour

ap, same a:	Bar soap	Compound, shared	Pit latrine with concret	Pit latrine v
ap, same a:	Bar soap	Compound, shared	Flush to septic tank	Flush/pour
ap, same a:	Bar soap	Compound, shared	Pit latrine with concret	Pit latrine v
), dedicatec	Liquid soap	Compound, not sharec	Flush to septic tank	Flush/pour
ap, same a:	Bar soap	Compound, shared	Flush to septic tank	Flush/pour
ap, same a:	Bathing soap	Compound, shared	Flush to donâ€™t kno	Flush to do
same as wh	Bar soap	Compound, shared	Pit latrine with concret	Pit latrine v
ap, same a:	Bar soap	Compound, shared	Flush to piped sewer s	Flush/pour
powder soa	Detergent/powder soap	Compound, shared	Pit latrine with concret	Pit latrine v
ap, same a:	Bar soap	Compound, shared	Pit latrine with concret	Pit latrine v
powder soa	Detergent/powder soap	Compound, not sharec	Bag	Bag
ap, same a:	Bar soap	Compound, shared	Flush to septic tank	Flush/pour
), dedicatec	Liquid soap	Compound, shared	Pit latrine with concret	Pit latrine v
same as wh	Bar soap	Compound, shared	Pit latrine with concret	Pit latrine v
dedicated o	Bar soap	Compound, not sharec	Flush to donâ€™t kno	Pit latrine v
ap, same a:	Bar soap	Compound, shared	Flush to piped sewer s	Flush/pour
dedicated o	Bar soap	Compound, shared	Flush to septic tank	Flush/pour
same as wh	Bar soap	Compound, shared	Pit latrine with concret	Pit latrine v
same as wh	Bar soap	Compound, shared	Pit latrine with concret	Pit latrine v
ap, same a:	Bar soap	Compound, shared	Pit latrine with concret	Pit latrine v
same as wh	Liquid soap	Compound, shared	Flush to septic tank	Flush/pour
), dedicatec	Liquid soap	Compound, shared	Flush to septic tank	Flush/pour
ap, same a:	Bathing soap	Compound, shared	Pit latrine with concret	Pit latrine v
ap, same a:	Bar soap	Compound, shared	Flush to septic tank	Flush/pour
dedicated o	Bar soap	Compound, shared	Flush to piped sewer s	Flush/pour
same as wh	Bar soap	Compound, shared	Flush to donâ€™t kno	Flush to do
same as wh	Bar soap	Compound, shared	Flush to septic tank	Flush/pour
same as wh	Bar soap	Compound, shared	Pit latrine with concret	Pit latrine v
ap, same a:	Bar soap	Compound, shared	Pit latrine with concret	Pit latrine v
dedicated o	Bar soap	Compound, shared	Flush to pit latrine	Flush/pour
same as wh	Bar soap	In the household	Pit latrine with concret	Pit latrine v
ap, same a:	Bar soap	Compound, shared	Flush to septic tank	Flush/pour
ap, same a:	Bar soap	Compound, shared	Flush to pit latrine	Flush/pour
same as wh	Bar soap	Compound, shared	Pit latrine with concret	Pit latrine v
), dedicatec	Bar soap	Compound, shared	Pit latrine without slab	Pit latrine v
same as wh	Bar soap	Compound, shared	Pit latrine with concret	Pit latrine v
same as wh	Bar soap	Compound, shared	Pit latrine with concret	Pit latrine v
ap, same a:	Bar soap	Neighboring compoun	Pit latrine with concret	Pit latrine v
same as wh	Bar soap	Compound, shared	Pit latrine with concret	Pit latrine v
ap, same a:	Bar soap	Compound, shared	Pit latrine with concret	Pit latrine v
same as wh	Bar soap	Compound, shared	Pit latrine with concret	Pit latrine v
same as wh	Bar soap	Compound, shared	Flush to pit latrine	Flush/pour
ap, same a:	Bar soap	Compound, shared	Flush to septic tank	Flush/pour
ap, same a:	Bar soap	Compound, shared	Pit latrine with concret	Pit latrine v
same as wh	Bar soap	Compound, shared	Flush to septic tank	Flush/pour
ap, same a:	Bar soap	Compound, shared	Flush to piped sewer s	Flush/pour
dedicated o	Bar soap	Compound, shared	Flush to piped sewer s	Flush/pour
dedicated o	Bar soap	Compound, shared	Flush to pit latrine	Flush/pour
or all house	Bar soap	Compound, shared	Flush to piped sewer s	Flush/pour

dedicated o	Bar soap	In the household	Flush to septic tank	Flush/pour
), dedicatec	Liquid soap	In the household	Flush to pit latrine	Flush/pour
same as wh	Bar soap	Compound, shared	Pit latrine with concret	Pit latrine v
), dedicatec	Liquid soap	In the household	Flush to septic tank	Flush/pour
), dedicatec	Liquid soap	In the household	Flush to piped sewer s	Flush/pour
ap, same a	Bathing soap	Compound, shared	Pit latrine with concret	Pit latrine v
same as wh	Detergent/powder soap	Compound, shared	Pit latrine with concret	Pit latrine v
ap, same a	Bar soap	Compound, shared	Flush to piped sewer s	Flush/pour
), dedicatec	Liquid soap	In the household	Flush to piped sewer s	Flush/pour
dedicated o	Bar soap	Compound, shared	Flush to piped sewer s	Flush/pour
powder soa	Detergent/powder soap	Compound, shared	Pit latrine with concret	Pit latrine v
same as wh	Bar soap	Neighboring compoun	Flush to septic tank	Flush/pour
ap, same a	Bar soap	Compound, shared	Pit latrine with concret	Pit latrine v
ap, same a	Bar soap	Compound, shared	Flush to piped sewer s	Flush/pour
or all house	Bar soap	Compound, not share	Flush to piped sewer s	Flush/pour
same as wh	Bar soap	Compound, shared	Pit latrine with concret	Flush/pour
), dedicatec	Liquid soap	Compound, not share	Flush to pit latrine	Flush/pour
same as wh	Bar soap	Compound, shared	Pit latrine with concret	Pit latrine v
), dedicatec	Liquid soap	Compound, shared	Pit latrine with concret	Pit latrine v
powder soa	Detergent/powder soap	Compound, not share	Flush to pit latrine	Flush/pour
same as wh	Bar soap	Neighboring compoun	Pit latrine without slab	Pit latrine v
ap, same a	Bar soap	Compound, shared	Flush to piped sewer s	Flush/pour
), dedicatec	Liquid soap	In the household	Flush to septic tank	Flush/pour
ap, same a	Bar soap	In the household	Flush to piped sewer s	Flush/pour
ap, same a	Bar soap	Compound, shared	Flush to piped sewer s	Flush/pour
ap, same a	Bar soap	Compound, shared	Flush to pit latrine	Flush/pour
powder soa	Detergent/powder soap	Compound, shared	Flush to septic tank	Flush/pour
), dedicatec	Liquid soap	Compound, shared	Ventilated improved pi	Pit latrine v
powder soa	Detergent/powder soap	Compound, shared	Flush to septic tank	Flush/pour
same as wh	Bar soap	Open spaces/bush		No facility/b
same as wh	Bar soap	Compound, shared	Pit latrine with concret	Flush/pour
same as wh	Bar soap	Compound, shared	Flush to septic tank	Flush/pour
), dedicatec	Liquid soap	Compound, shared	Pit latrine with concret	Pit latrine v
or all house	Bar soap	Compound, shared	Flush to pit latrine	Flush/pour
same as wh	Bar soap	Compound, shared	Pit latrine with concret	Pit latrine v
same as wh	Bar soap	Neighboring compoun	Pit latrine with concret	Pit latrine v
powder soa	Detergent/powder soap	In the household	Flush to septic tank	Flush/pour
ap, same a	Bar soap	Compound, shared	Flush to pit latrine	Flush/pour
ap, same a	Liquid soap	In the household	Flush to septic tank	Flush/pour
or all house	Bar soap	Compound, shared	Flush to piped sewer s	Flush/pour
same as wh	Bar soap	Neighboring compoun	Pit latrine with concret	Pit latrine v
same as wh	Bar soap	Compound, shared	Pit latrine with concret	Pit latrine v
ap, same a	Bar soap	Compound, shared	Flush to septic tank	Flush/pour
same as wh	Bar soap	Compound, shared	Flush to open pit	Pit latrine v
or all house	Bar soap	Compound, shared	Flush to piped sewer s	Flush/pour
same as wh	Bar soap	Compound, shared	Flush to pit latrine	Flush/pour
), dedicatec	Liquid soap	Compound, shared	Flush to pit latrine	Flush/pour
ap, same a	Liquid soap	Compound, shared	Flush to piped sewer s	Flush/pour
same as wh	Bar soap	Compound, shared	Pit latrine without slab	Compostin

ap, same a:	Bathing soap	Compound, not sharec	Pit latrine with concret	Pit latrine v
ap, same a:	Bathing soap	In the household	Flush to piped sewer s	Flush/pour
ap, same a:	Bar soap	Compound, shared	Pit latrine without slab	Pit latrine v
or all house	Bar soap	Compound, shared	Pit latrine with concret	Pit latrine v
o, dedicatec	Liquid soap	Compound, shared	Flush to piped sewer s	Flush/pour
ap, same a:	Bar soap	Compound, shared	Flush to pit latrine	Flush/pour
o, dedicatec	Liquid soap	Compound, shared	Pit latrine with concret	Pit latrine v
ap, same a:	Bar soap	Compound, shared	Pit latrine without slab	Pit latrine v
or all house	Liquid soap	Compound, shared	Flush to septic tank	Flush/pour
same as wh	Bar soap	Community latrine	Pit latrine with concret	Pit latrine v
powder soa	Detergent/powder soap	In the household	Flush to septic tank	Flush/pour
same as wh	Bar soap	Compound, not sharec	Flush to septic tank	Pit latrine v
o, dedicatec	Liquid soap	Compound, shared	Flush to piped sewer s	Flush/pour
o, also for o	Liquid soap	Compound, shared	Flush to pit latrine	Flush/pour
o, dedicatec	Liquid soap	Compound, not sharec	Pit latrine with concret	Pit latrine v
ap, same a:	Bathing soap	Compound, shared	Pit latrine with concret	Pit latrine v
or all house	Bar soap	Compound, shared	Flush to piped sewer s	Flush/pour
ap, same a:	Bar soap	Compound, shared	Flush to septic tank	Flush/pour
same as wh	Bar soap	Compound, shared	Flush to pit latrine	Flush/pour
same as wh	Bar soap	Compound, shared	Pit latrine with concret	Pit latrine v
ap, same a:	Bar soap	Compound, shared	Pit latrine with concret	Pit latrine v
same as wh	Bar soap	Neighboring compoun	Pit latrine with concret	Pit latrine v
or all house	Bar soap	Compound, shared	Flush to piped sewer s	Flush/pour
o, dedicatec	Liquid soap	Compound, not sharec	Flush to septic tank	Flush/pour
o, dedicatec	Liquid soap	Compound, shared	Flush to piped sewer s	Flush/pour

q63b	q64	q65a	q65a_1	q65a_2	q65a_3	q65a_4	q65a_5	q65a_6
flush toilet	11-15 peo	1 3	1	0	1	0	0	0
vith concret	Above 15 p	1 3	1	0	1	0	0	0
flush toilet	11-15 peo	3 4	0	0	1	1	0	0
flush toilet	11-15 peo	3	0	0	1	0	0	0
flush toilet	1-5 people	1	1	0	0	0	0	0
flush toilet	Above 15 p	1 3 4 5 6 7	1	0	1	1	1	1
flush toilet	Above 15 p	1 3	1	0	1	0	0	0
flush toilet	Above 15 p	1 3	1	0	1	0	0	0
vith concret	11-15 peo	1 3	1	0	1	0	0	0
vith concret	6-10 peopl	1 3	1	0	1	0	0	0
flush toilet	11-15 peo	3 4	0	0	1	1	0	0
flush toilet	Above 15 p	1 3 5	1	0	1	0	1	0
flush toilet	Do not sha	1	1	0	0	0	0	0
vith concret	6-10 peopl	3 4 5	0	0	1	1	1	0
vith concret	Above 15 p	1 3 6	1	0	1	0	0	1
vith concret	Above 15 p	2 4 6 7	0	1	0	1	0	1
vith concret	Above 15 p	1 3 6	1	0	1	0	0	1
flush toilet	11-15 peo	3	0	0	1	0	0	0
flush toilet	Above 15 p	4	0	0	0	1	0	0
flush toilet	Above 15 p	1 3 6	1	0	1	0	0	1
flush toilet	Donâ€™t k	1 3	1	0	1	0	0	0
vith concret	Above 15 p	3 4	0	0	1	1	0	0
vith concret	Above 15 p	1 3	1	0	1	0	0	0
vith concret	6-10 peopl	1	1	0	0	0	0	0
vith concret	Above 15 p	1 3	1	0	1	0	0	0
vith concret	11-15 peo	3 6	0	0	1	0	0	1
vith concret	Do not sha	1	1	0	0	0	0	0
flush toilet	Above 15 p	4	0	0	0	1	0	0
vith concret	Donâ€™t k	1 3 4 7	1	0	1	1	0	0
flush toilet	11-15 peo	1	1	0	0	0	0	0
vith concret	Above 15 p	1 3	1	0	1	0	0	0
flush toilet	6-10 peopl	1	1	0	0	0	0	0
flush toilet	Above 15 p	1 3	1	0	1	0	0	0
flush toilet	6-10 peopl	3	0	0	1	0	0	0
vith concret	6-10 peopl	3	0	0	1	0	0	0
vith concret	1-5 people	5	0	0	0	0	1	0
flush toilet	11-15 peo	1 3 4	1	0	1	1	0	0
flush toilet	11-15 peo	1	1	0	0	0	0	0
vith concret	Above 15 p	1 2 3 4 6	1	1	1	1	0	1
flush toilet	6-10 peopl	1 3	1	0	1	0	0	0
vith concret	Above 15 p	1 3	1	0	1	0	0	0
flush toilet	11-15 peo	1 3	1	0	1	0	0	0
vith concret	6-10 peopl	1	1	0	0	0	0	0
vith concret	6-10 peopl	1	1	0	0	0	0	0
flush toilet	11-15 peo	1 4 5	1	0	0	1	1	0
flush toilet	11-15 peo	1 4	1	0	0	1	0	0
flush toilet	1-5 people	1	1	0	0	0	0	0
flush toilet	6-10 peopl	4	0	0	0	1	0	0

flush toilet	Above 15 p	1 3	1	0	1	0	0	0
flush toilet	6-10 peopl	3	0	0	1	0	0	0
ɔush/field	Donâ€™t k	1	1	0	0	0	0	0
flush toilet	6-10 peopl	1	1	0	0	0	0	0
flush toilet	11-15 peop	1	1	0	0	0	0	0
vith concret	Above 15 p	2 3 4 5 6	0	1	1	1	1	1
vith concret	Above 15 p	1 2 3 4	1	1	1	1	0	0
flush toilet	Above 15 p	1 3	1	0	1	0	0	0
flush toilet	6-10 peopl	1 2	1	1	0	0	0	0
vith concret	1-5 people	1 2	1	1	0	0	0	0
flush toilet	Above 15 p	1 3	1	0	1	0	0	0
flush toilet	11-15 peop	1 3	1	0	1	0	0	0
vith concret	Donâ€™t k	1 3 4 7	1	0	1	1	0	0
flush toilet	Above 15 p	1	1	0	0	0	0	0
vith concret	1-5 people	1	1	0	0	0	0	0
flush toilet	1-5 people	1	1	0	0	0	0	0
flush toilet	11-15 peop	1 4	1	0	0	1	0	0
flush toilet	1-5 people	1 3 4 5	1	0	1	1	1	0
flush toilet	Above 15 p	1 3 4 5	1	0	1	1	1	0
flush toilet	11-15 peop	1 3 4	1	0	1	1	0	0
vith concret	Above 15 p	3	0	0	1	0	0	0
flush toilet	1-5 people	1	1	0	0	0	0	0
flush toilet	Above 15 p	1 3 4 5 6 7	1	0	1	1	1	1
vithout slab	Donâ€™t k	1 2	1	1	0	0	0	0
vith concret	Above 15 p	1 3	1	0	1	0	0	0
flush toilet	6-10 peopl	1	1	0	0	0	0	0
flush toilet	6-10 peopl	3 4	0	0	1	1	0	0
flush toilet	1-5 people	3	0	0	1	0	0	0
ɔush/field	Donâ€™t k	7	0	0	0	0	0	0
vith concret	Above 15 p	1 3	1	0	1	0	0	0
flush toilet	6-10 peopl	1	1	0	0	0	0	0
vith concret	6-10 peopl	1 3	1	0	1	0	0	0
vith concret	6-10 peopl	1	1	0	0	0	0	0
vith concret	Above 15 p	2 3 5	0	1	1	0	1	0
vithout slab	1-5 people	1	1	0	0	0	0	0
vith concret	11-15 peop	1 3	1	0	1	0	0	0
flush toilet	11-15 peop	3	0	0	1	0	0	0
flush toilet	Above 15 p	3	0	0	1	0	0	0
vith concret	Above 15 p	1 3	1	0	1	0	0	0
flush toilet	6-10 peopl	1 3	1	0	1	0	0	0
flush toilet	11-15 peop	1 3	1	0	1	0	0	0
flush toilet	6-10 peopl	1 3 6	1	0	1	0	0	1
flush toilet	11-15 peop	1 3	1	0	1	0	0	0
flush toilet	1-5 people	1	1	0	0	0	0	0
flush toilet	Above 15 p	1	1	0	0	0	0	0
flush toilet	11-15 peop	1 3	1	0	1	0	0	0
flush toilet	Above 15 p	1 3	1	0	1	0	0	0
flush toilet	11-15 peop	1	1	0	0	0	0	0
vith concret	Do not sha	1	1	0	0	0	0	0

flush toilet	1-5 people	3	0	0	1	0	0	0
onâ€™t kno	Do not sha	96	0	0	0	0	0	0
flush toilet	Donâ€™t k	1 3 4 5 7	1	0	1	1	1	0
vith concret	11-15 peop	1 3	1	0	1	0	0	0
flush toilet	Above 15 p	1 3	1	0	1	0	0	0
vith concret	11-15 peop	3	0	0	1	0	0	0
vith concret	Donâ€™t k	1 3 4 7	1	0	1	1	0	0
flush toilet	6-10 peopl	1	1	0	0	0	0	0
flush toilet	6-10 peopl	1	1	0	0	0	0	0
vith concret	11-15 peop	3	0	0	1	0	0	0
flush toilet	11-15 peop	1 3	1	0	1	0	0	0
Potty	Above 15 p	1	1	0	0	0	0	0
vith concret	1-5 people	1	1	0	0	0	0	0
flush toilet	11-15 peop	1 3	1	0	1	0	0	0
bush/field	Donâ€™t k	7	0	0	0	0	0	0
bush/field	Donâ€™t k	7	0	0	0	0	0	0
vith concret	Above 15 p	1 3	1	0	1	0	0	0
flush toilet	6-10 peopl	1	1	0	0	0	0	0
vith concret	Above 15 p	1 3 4	1	0	1	1	0	0
flush toilet	Above 15 p	1 2 3 4	1	1	1	1	0	0
vithout slab	11-15 peop	1 3	1	0	1	0	0	0
flush toilet	11-15 peop	1 3	1	0	1	0	0	0
flush toilet	11-15 peop	3	0	0	1	0	0	0
flush toilet	1-5 people	1 2	1	1	0	0	0	0
flush toilet	6-10 peopl	1	1	0	0	0	0	0
vithout slab	Above 15 p	1 3	1	0	1	0	0	0
flush toilet	6-10 peopl	1	1	0	0	0	0	0
vith concret	Donâ€™t k	1 3 4	1	0	1	1	0	0
flush toilet	1-5 people	1	1	0	0	0	0	0
flush toilet	6-10 peopl	1	1	0	0	0	0	0
vith concret	Above 15 p	1 3 5	1	0	1	0	1	0
vith concret	Above 15 p	1 3 4 5 6 7	1	0	1	1	1	1
flush toilet	1-5 people	1 2	1	1	0	0	0	0
vith concret	Above 15 p	1 2 3 4 5 6	1	1	1	1	1	1
vith concret	Above 15 p	3	0	0	1	0	0	0
flush toilet	11-15 peop	1 3 4 5	1	0	1	1	1	0
vith concret	Donâ€™t k	1 3 7	1	0	1	0	0	0
vith concret	6-10 peopl	1 3	1	0	1	0	0	0
flush toilet	6-10 peopl	4	0	0	0	1	0	0
flush toilet	Above 15 p	3	0	0	1	0	0	0
flush toilet	6-10 peopl	3	0	0	1	0	0	0
flush toilet	6-10 peopl	1 2	1	1	0	0	0	0
flush toilet	Above 15 p	1 3 4 5 7	1	0	1	1	1	0
flush toilet	6-10 peopl	1 3	1	0	1	0	0	0
vith concret	Donâ€™t k	1 3	1	0	1	0	0	0
flush toilet	6-10 peopl	1	1	0	0	0	0	0
vith concret	1-5 people	1	1	0	0	0	0	0
vith concret	6-10 peopl	3	0	0	1	0	0	0
vith concret	11-15 peop	1 3	1	0	1	0	0	0

vithout slab	1-5 people	1	1	0	0	0	0	0
)ush/field	Donâ€™t k	1	1	0	0	0	0	0
vith concret	6-10 peopl	1 3	1	0	1	0	0	0
flush toilet	1-5 people	1 4	1	0	0	1	0	0
flush toilet	1-5 people	1	1	0	0	0	0	0
flush toilet	11-15 peop	1 2 3	1	1	1	0	0	0
flush toilet	Above 15 p	3	0	0	1	0	0	0
vith concret	Donâ€™t k	1 3 4 7	1	0	1	1	0	0
flush toilet	Above 15 p	4	0	0	0	1	0	0
vith concret	6-10 peopl	1 96	1	0	0	0	0	0
flush toilet	11-15 peop	1 3 4	1	0	1	1	0	0
vith concret	11-15 peop	3 5	0	0	1	0	1	0
flush toilet	6-10 peopl	1 3	1	0	1	0	0	0
flush toilet	11-15 peop	1 3 4	1	0	1	1	0	0
flush toilet	1-5 people	1	1	0	0	0	0	0
vith concret	Above 15 p	1 3 5	1	0	1	0	1	0
vith concret	6-10 peopl	4	0	0	0	1	0	0
vith concret	11-15 peop	3	0	0	1	0	0	0
flush toilet	6-10 peopl	3	0	0	1	0	0	0
flush toilet	11-15 peop	3	0	0	1	0	0	0
vith concret	Donâ€™t k	3	0	0	1	0	0	0
flush toilet	11-15 peop	1	1	0	0	0	0	0
flush toilet	6-10 peopl	1	1	0	0	0	0	0
flush toilet	Donâ€™t k	1 2 3	1	1	1	0	0	0
vith concret	11-15 peop	3	0	0	1	0	0	0
vithout slab	1-5 people	1	1	0	0	0	0	0
vithout slab	6-10 peopl	1 3	1	0	1	0	0	0
)ush/field	Donâ€™t k	1 7	1	0	0	0	0	0
flush toilet	11-15 peop	3	0	0	1	0	0	0
vith concret	Donâ€™t k	1	1	0	0	0	0	0
flush toilet	11-15 peop	1 3	1	0	1	0	0	0
flush toilet	Above 15 p	1 3	1	0	1	0	0	0
flush toilet	1-5 people	1	1	0	0	0	0	0
vith concret	1-5 people	1	1	0	0	0	0	0
flush toilet	1-5 people	1	1	0	0	0	0	0
flush toilet	1-5 people	1	1	0	0	0	0	0
vith concret	6-10 peopl	1	1	0	0	0	0	0
flush toilet	Above 15 p	1 3 4	1	0	1	1	0	0
vith concret	11-15 peop	1 4	1	0	0	1	0	0
flush toilet	11-15 peop	1 3	1	0	1	0	0	0
vith concret	Above 15 p	1 4	1	0	0	1	0	0
)ush/field	Donâ€™t k	1 7	1	0	0	0	0	0
vith concret	Above 15 p	1 3	1	0	1	0	0	0
flush toilet	Above 15 p	1 3 4 5 6 7	1	0	1	1	1	1
vith concret	Above 15 p	3	0	0	1	0	0	0
flush toilet	1-5 people	1	1	0	0	0	0	0
flush toilet	6-10 peopl	1	1	0	0	0	0	0
flush toilet	Above 15 p	1 3 4	1	0	1	1	0	0
flush toilet	11-15 peop	3	0	0	1	0	0	0

vith concret	11-15 peop	3	0	0	1	0	0	0
flush toilet	1-5 people	1	1	0	0	0	0	0
vith concret	Above 15 p	1 3	1	0	1	0	0	0
flush toilet	6-10 peopl	1	1	0	0	0	0	0
ɔush/field	Do not sha	96	0	0	0	0	0	0
flush toilet	6-10 peopl	1 3	1	0	1	0	0	0
vith concret	Above 15 p	3 4 5 7	0	0	1	1	1	0
vith concret	1-5 people	1	1	0	0	0	0	0
flush toilet	1-5 people	1	1	0	0	0	0	0
flush toilet	Above 15 p	1 4	1	0	0	1	0	0
flush toilet	6-10 peopl	1	1	0	0	0	0	0
flush toilet	6-10 peopl	1	1	0	0	0	0	0
flush toilet	6-10 peopl	1 2 3 4	1	1	1	1	0	0
vith concret	6-10 peopl	1	1	0	0	0	0	0
flush toilet	1-5 people	1	1	0	0	0	0	0
vith concret	Above 15 p	1 3	1	0	1	0	0	0
flush toilet	11-15 peop	1	1	0	0	0	0	0
ɔnâ€™t kno	Above 15 p	1 3 4 5 7	1	0	1	1	1	0
flush toilet	6-10 peopl	1 3	1	0	1	0	0	0
flush toilet	6-10 peopl	1	1	0	0	0	0	0
flush toilet	6-10 peopl	1	1	0	0	0	0	0
flush toilet	Above 15 p	1 3 4 5 7	1	0	1	1	1	0
vithout slab	1-5 people	1	1	0	0	0	0	0
vithout slab	11-15 peop	3 5	0	0	1	0	1	0
flush toilet	11-15 peop	1	1	0	0	0	0	0
vith concret	6-10 peopl	1 3	1	0	1	0	0	0
vith concret	11-15 peop	3	0	0	1	0	0	0
ɔush/field	Donâ€™t k	7	0	0	0	0	0	0
vith concret	Above 15 p	1 3 4 5 6 7	1	0	1	1	1	1
vith concret	Above 15 p	1 3 4	1	0	1	1	0	0
flush toilet	11-15 peop	3	0	0	1	0	0	0
flush toilet	6-10 peopl	1	1	0	0	0	0	0
flush toilet	Above 15 p	1 3 4	1	0	1	1	0	0
vith concret	6-10 peopl	3	0	0	1	0	0	0
flush toilet	Above 15 p	3 4	0	0	1	1	0	0
vith concret	6-10 peopl	1	1	0	0	0	0	0
ɔush/field	Donâ€™t k	7	0	0	0	0	0	0
flush toilet	Donâ€™t k	3 4 5	0	0	1	1	1	0
flush toilet	1-5 people	1	1	0	0	0	0	0
flush toilet	Above 15 p	1 3	1	0	1	0	0	0
flush toilet	6-10 peopl	1 3 4	1	0	1	1	0	0
vithout slab	1-5 people	1	1	0	0	0	0	0
flush toilet	1-5 people	1	1	0	0	0	0	0
flush toilet	1-5 people	3	0	0	1	0	0	0
flush toilet	11-15 peop	1 3	1	0	1	0	0	0
flush toilet	Above 15 p	1 3 4	1	0	1	1	0	0
flush toilet	6-10 peopl	1 4	1	0	0	1	0	0
flush toilet	11-15 peop	1 2 3	1	1	1	0	0	0
vith concret	Above 15 p	3 4	0	0	1	1	0	0

flush toilet	Above 15 p	1 2 3 4	1	1	1	1	0	0
flush toilet	1-5 people	1	1	0	0	0	0	0
flush toilet	Above 15 p	3	0	0	1	0	0	0
vith concret	11-15 peop	3	0	0	1	0	0	0
flush toilet	Above 15 p	1 3	1	0	1	0	0	0
flush toilet	Above 15 p	3 4 6 7	0	0	1	1	0	1
vith concret	1-5 people	1	1	0	0	0	0	0
flush toilet	Above 15 p	1 3 4 5	1	0	1	1	1	0
flush toilet	6-10 peopl	1	1	0	0	0	0	0
vith concret	1-5 people	1	1	0	0	0	0	0
vith concret	Above 15 p	3	0	0	1	0	0	0
flush toilet	1-5 people	1	1	0	0	0	0	0
vith concret	11-15 peop	1 2 3	1	1	1	0	0	0
flush toilet	1-5 people	1	1	0	0	0	0	0
flush toilet	11-15 peop	4 5	0	0	0	1	1	0
flush toilet	6-10 peopl	1 4	1	0	0	1	0	0
flush toilet	6-10 peopl	1	1	0	0	0	0	0
flush toilet	Above 15 p	1 3 4	1	0	1	1	0	0
vith concret	6-10 peopl	1	1	0	0	0	0	0
vith concret	Above 15 p	3 4	0	0	1	1	0	0
vith concret	Donâ€™t k	1 4 7	1	0	0	1	0	0
vith concret	Above 15 p	1 3 4 5	1	0	1	1	1	0
flush toilet	6-10 peopl	1	1	0	0	0	0	0
onâ€™t kno	1-5 people	1	1	0	0	0	0	0
flush toilet	Above 15 p	1 3 4 6 7	1	0	1	1	0	1
vith concret	6-10 peopl	3	0	0	1	0	0	0
flush toilet	Above 15 p	1 2 5	1	1	0	0	1	0
flush toilet	11-15 peop	1	1	0	0	0	0	0
vith concret	Above 15 p	1 3 4	1	0	1	1	0	0
vith concret	Above 15 p	1 4 5	1	0	0	1	1	0
vith concret	11-15 peop	1 4	1	0	0	1	0	0
flush toilet	6-10 peopl	1	1	0	0	0	0	0
flush toilet	11-15 peop	1	1	0	0	0	0	0
flush toilet	Above 15 p	3	0	0	1	0	0	0
flush toilet	1-5 people	1 3	1	0	1	0	0	0
vith concret	6-10 peopl	3	0	0	1	0	0	0
flush toilet	Above 15 p	1 3 4 5	1	0	1	1	1	0
flush toilet	Above 15 p	1 3	1	0	1	0	0	0
flush toilet	Above 15 p	1 2 3 4	1	1	1	1	0	0
flush toilet	1-5 people	1	1	0	0	0	0	0
flush toilet	Above 15 p	4 5 7	0	0	0	1	1	0
vith concret	6-10 peopl	1	1	0	0	0	0	0
vith concret	6-10 peopl	1 3	1	0	1	0	0	0
flush toilet	6-10 peopl	1 3	1	0	1	0	0	0
flush toilet	6-10 peopl	1 2	1	1	0	0	0	0
vith concret	Above 15 p	1 3	1	0	1	0	0	0
flush toilet	Above 15 p	1 3 4 5	1	0	1	1	1	0
vith concret	11-15 peop	1 4	1	0	0	1	0	0
vith concret	Above 15 p	1 3 4 5	1	0	1	1	1	0

flush toilet	Above 15 p	1 3 4		1	0	1	1	0	0
vith concret	6-10 peopl	1 2 4		1	1	0	1	0	0
flush toilet	Donâ€™t k	4 5		0	0	0	1	1	0
flush toilet	6-10 peopl	1 2 3		1	1	1	0	0	0
vith concret	6-10 peopl	1 3 6		1	0	1	0	0	1
vith concret	Above 15 p	1 3		1	0	1	0	0	0
vith concret	1-5 people	1 3		1	0	1	0	0	0
vith concret	6-10 peopl	1 3		1	0	1	0	0	0
flush toilet	6-10 peopl		4	0	0	0	1	0	0
vith concret	Above 15 p	1 3		1	0	1	0	0	0
flush toilet	Above 15 p	1 3 4 5		1	0	1	1	1	0
flush toilet	Above 15 p		3	0	0	1	0	0	0
vith concret	Above 15 p		3	0	0	1	0	0	0
flush toilet	6-10 peopl		1	1	0	0	0	0	0
vith concret	Above 15 p	1 3 4		1	0	1	1	0	0
flush toilet	Above 15 p		4	0	0	0	1	0	0
flush toilet	6-10 peopl		1	1	0	0	0	0	0
flush toilet	Above 15 p	1 3		1	0	1	0	0	0
flush toilet	Above 15 p	1 3 6		1	0	1	0	0	1
vith concret	Above 15 p		3	0	0	1	0	0	0
flush toilet	1-5 people		1	1	0	0	0	0	0
flush toilet	6-10 peopl	1 3		1	0	1	0	0	0
vith concret	6-10 peopl	1 3		1	0	1	0	0	0
vithout slab	6-10 peopl		1	1	0	0	0	0	0
vith concret	11-15 peop	3 4		0	0	1	1	0	0
flush toilet	Above 15 p	1 3 4 5 6 7		1	0	1	1	1	1
vith concret	Above 15 p	1 3 4 5		1	0	1	1	1	0
flush toilet	Above 15 p	1 3 4		1	0	1	1	0	0
flush toilet	1-5 people		1	1	0	0	0	0	0
flush toilet	11-15 peop	1 3 4 6 7 9		1	0	1	1	0	1
flush toilet	6-10 peopl		1	1	0	0	0	0	0
vith concret	1-5 people		1	1	0	0	0	0	0
flush toilet	11-15 peop	1 3 4 5		1	0	1	1	1	0
vithout slab	Above 15 p	1 3 4		1	0	1	1	0	0
flush toilet	6-10 peopl	1 2		1	1	0	0	0	0
flush toilet	6-10 peopl		1	1	0	0	0	0	0
flush toilet	11-15 peop	1 4		1	0	0	1	0	0
flush toilet	Above 15 p	1 3 4		1	0	1	1	0	0
vith concret	Above 15 p	1 3 4 5 6		1	0	1	1	1	1
vith concret	Above 15 p	4 5		0	0	0	1	1	0
flush toilet	1-5 people		1	1	0	0	0	0	0
flush toilet	Above 15 p	1 3 4		1	0	1	1	0	0
flush toilet	1-5 people		1	1	0	0	0	0	0
vith concret	Above 15 p		3	0	0	1	0	0	0
vith concret	Donâ€™t k	1 4 5 6		1	0	0	1	1	1
nâ€™t kno	Above 15 p	1 3 4 5		1	0	1	1	1	0
vithout slab	6-10 peopl		1	1	0	0	0	0	0
vith concret	Above 15 p	1 2 3		1	1	1	0	0	0
vith concret	11-15 peop		3	0	0	1	0	0	0

flush toilet	Above 15 p	3 4 5 6	0	0	1	1	1	1
vith concret	11-15 peop	1 4	1	0	0	1	0	0
flush toilet	11-15 peop	1 2	1	1	0	0	0	0
onâ€™t kno	6-10 peopl	1 3 4	1	0	1	1	0	0
flush toilet	1-5 people	1	1	0	0	0	0	0
vith concret	Above 15 p	1 4 5	1	0	0	1	1	0
vith concret	Above 15 p	1 4	1	0	0	1	0	0
vith concret	Above 15 p	2 3 4 5 6 7	0	1	1	1	1	1
flush toilet	1-5 people	1	1	0	0	0	0	0
flush toilet	6-10 peopl	3	0	0	1	0	0	0
vith concret	Above 15 p	1 3 4	1	0	1	1	0	0
flush toilet	1-5 people	1	1	0	0	0	0	0
flush toilet	11-15 peop	3	0	0	1	0	0	0
vithout slab	11-15 peop	1 2 3 4	1	1	1	1	0	0
vithout slab	Above 15 p	1 2 3	1	1	1	0	0	0
vithout slab	Above 15 p	2 3 4 6	0	1	1	1	0	1
flush toilet	6-10 peopl	1	1	0	0	0	0	0
flush toilet	11-15 peop	1 3	1	0	1	0	0	0
vith concret	Above 15 p	3	0	0	1	0	0	0
vith concret	Above 15 p	1 3	1	0	1	0	0	0
vith concret	1-5 people	2 3	0	1	1	0	0	0
flush toilet	Above 15 p	1 3	1	0	1	0	0	0
flush toilet	Above 15 p	1 3 4 5	1	0	1	1	1	0
flush toilet	Above 15 p	3 4	0	0	1	1	0	0
flush toilet	Above 15 p	1 4	1	0	0	1	0	0
flush toilet	11-15 peop	1 3	1	0	1	0	0	0
flush toilet	1-5 people	1	1	0	0	0	0	0
flush toilet	6-10 peopl	1	1	0	0	0	0	0
flush toilet	Donâ€™t k	6 7	0	0	0	0	0	1
vithout slab	Donâ€™t k	1 2 5	1	1	0	0	1	0
flush toilet	1-5 people	3	0	0	1	0	0	0
flush toilet	Above 15 p	3 4 5	0	0	1	1	1	0
vith concret	11-15 peop	1 3	1	0	1	0	0	0
flush toilet	11-15 peop	1	1	0	0	0	0	0
flush toilet	Above 15 p	1 3 6 7	1	0	1	0	0	1
vith concret	Above 15 p	1 3	1	0	1	0	0	0
flush toilet	Above 15 p	1 3 4 5	1	0	1	1	1	0
vith concret	Above 15 p	1 3 4	1	0	1	1	0	0
flush toilet	Above 15 p	1 3	1	0	1	0	0	0
vith concret	Above 15 p	1 2 3	1	1	1	0	0	0
vith concret	6-10 peopl	1 3 4	1	0	1	1	0	0
vith concret	6-10 peopl	1 3	1	0	1	0	0	0
vith concret	Above 15 p	1 3 4 6 7	1	0	1	1	0	1
flush toilet	11-15 peop	3	0	0	1	0	0	0
flush toilet	11-15 peop	1 2 3 4	1	1	1	1	0	0
flush toilet	Above 15 p	4	0	0	0	1	0	0
flush toilet	11-15 peop	4	0	0	0	1	0	0
vith concret	11-15 peop	1 2 3 4 6	1	1	1	1	0	1
vith concret	11-15 peop	3	0	0	1	0	0	0

flush toilet	1-5 people	1	1	0	0	0	0	0
flush toilet	Above 15 p	1 3 4	1	0	1	1	0	0
flush toilet	Above 15 p	4	0	0	0	1	0	0
flush toilet	11-15 peop	1 3	1	0	1	0	0	0
flush toilet	6-10 peopl	1	1	0	0	0	0	0
vith concret	Above 15 p	1 3	1	0	1	0	0	0
flush toilet	1-5 people	1	1	0	0	0	0	0
flush toilet	6-10 peopl	1	1	0	0	0	0	0
flush toilet	Above 15 p	4	0	0	0	1	0	0
onâ€™t kno	Above 15 p	1 2 3 4 5 6	1	1	1	1	1	1
flush toilet	1-5 people	1 3 4	1	0	1	1	0	0
vith concret	Above 15 p	1 3 4	1	0	1	1	0	0
flush toilet	1-5 people	1 2	1	1	0	0	0	0
vith concret	6-10 peopl	1 2 3	1	1	1	0	0	0
flush toilet	Above 15 p	1 3 4	1	0	1	1	0	0
flush toilet	Above 15 p	1 3 4 5	1	0	1	1	1	0
flush toilet	6-10 peopl	1 4	1	0	0	1	0	0
flush toilet	Above 15 p	1 2 3	1	1	1	0	0	0
vith concret	Above 15 p	1 2 4 6	1	1	0	1	0	1
vith concret	11-15 peop	3	0	0	1	0	0	0
vith concret	6-10 peopl	1 3 4 5	1	0	1	1	1	0
flush toilet	Above 15 p	1 3 4 5	1	0	1	1	1	0
vith concret	6-10 peopl	1 3	1	0	1	0	0	0
vith concret	Above 15 p	1 3	1	0	1	0	0	0
vith concret	Above 15 p	1 2 3 4	1	1	1	1	0	0
flush toilet	Above 15 p	1 3 4	1	0	1	1	0	0
vith concret	Above 15 p	2 4 6	0	1	0	1	0	1
flush toilet	1-5 people	1	1	0	0	0	0	0
vithout slab	Above 15 p	1 2 3 4	1	1	1	1	0	0
vith concret	Above 15 p	1 3	1	0	1	0	0	0
flush toilet	6-10 peopl	1	1	0	0	0	0	0
vith concret	Above 15 p	1 3	1	0	1	0	0	0
vithout slab	11-15 peop	1 3	1	0	1	0	0	0
vith concret	6-10 peopl	1 3 5 6	1	0	1	0	1	1
vith concret	6-10 peopl	2 3 4 5 6	0	1	1	1	1	1
vith concret	Donâ€™t k	1 3	1	0	1	0	0	0
vith concret	11-15 peop	1 3 4	1	0	1	1	0	0
flush toilet	6-10 peopl	3	0	0	1	0	0	0
flush toilet	Above 15 p	3	0	0	1	0	0	0
flush toilet	1-5 people	1	1	0	0	0	0	0
vithout slab	Above 15 p	1 2 3 4 5 6	1	1	1	1	1	1
flush toilet	11-15 peop	1 3 4	1	0	1	1	0	0
vith concret	Above 15 p	1 3 4 5 6 7	1	0	1	1	1	1
flush toilet	6-10 peopl	1 3	1	0	1	0	0	0
flush toilet	Above 15 p	4 5	0	0	0	1	1	0
vith concret	6-10 peopl	1	1	0	0	0	0	0
vithout slab	11-15 peop	1 3 4	1	0	1	1	0	0
flush toilet	1-5 people	3	0	0	1	0	0	0
flush toilet	Above 15 p	4 5	0	0	0	1	1	0

flush toilet	Above 15 p	4	0	0	0	1	0	0
flush toilet	6-10 peopl	1 3	1	0	1	0	0	0
flush toilet	6-10 peopl	1 3	1	0	1	0	0	0
vith concret	Above 15 p	3	0	0	1	0	0	0
vith concret	Above 15 p	3 7	0	0	1	0	0	0
vith concret	11-15 peop	1 2 3 4 5 6	1	1	1	1	1	1
vith concret	Above 15 p	1 3 5	1	0	1	0	1	0
flush toilet	1-5 people	3	0	0	1	0	0	0
flush toilet	Above 15 p	1 3 4	1	0	1	1	0	0
vithout slab	Above 15 p	1 2 3	1	1	1	0	0	0
flush toilet	11-15 peop	1 2 3	1	1	1	0	0	0
flush toilet	11-15 peop	1 3	1	0	1	0	0	0
oush/field	Donâ€™t k	7	0	0	0	0	0	0
vith concret	Above 15 p	1 3 6	1	0	1	0	0	1
flush toilet	1-5 people	3	0	0	1	0	0	0
flush toilet	6-10 peopl	1	1	0	0	0	0	0
vith concret	1-5 people	1	1	0	0	0	0	0
vith concret	Above 15 p	1 2 3 4 6	1	1	1	1	0	1
flush toilet	6-10 peopl	1	1	0	0	0	0	0
flush toilet	11-15 peop	1 3	1	0	1	0	0	0
vith concret	Above 15 p	1 2 3 4 5 6	1	1	1	1	1	1
vith concret	11-15 peop	1 4 5	1	0	0	1	1	0
flush toilet	6-10 peopl	1	1	0	0	0	0	0
vith concret	6-10 peopl	1	1	0	0	0	0	0
vith concret	11-15 peop	1 2 4	1	1	0	1	0	0
vithout slab	Above 15 p	1 4	1	0	0	1	0	0
flush toilet	1-5 people	1	1	0	0	0	0	0
vith concret	11-15 peop	1 3 4	1	0	1	1	0	0
vith concret	Above 15 p	3 4 5	0	0	1	1	1	0
vith concret	Above 15 p	1 3 6	1	0	1	0	0	1
vith concret	Above 15 p	2 5	0	1	0	0	1	0
vith concret	Above 15 p	1 3	1	0	1	0	0	0
flush toilet	11-15 peop	1 3	1	0	1	0	0	0
flush toilet	Above 15 p	1 3 4	1	0	1	1	0	0
flush toilet	Donâ€™t k	1 3 4 6 7	1	0	1	1	0	1
flush toilet	Above 15 p	1 3 4	1	0	1	1	0	0
g/EcoSan t	6-10 peopl	1 2	1	1	0	0	0	0
vith concret	6-10 peopl	1	1	0	0	0	0	0
flush toilet	1-5 people	1	1	0	0	0	0	0
flush toilet	11-15 peop	1 3	1	0	1	0	0	0
flush toilet	1-5 people	1	1	0	0	0	0	0
flush toilet	11-15 peop	1 2 3 4	1	1	1	1	0	0
flush toilet	Above 15 p	3 4	0	0	1	1	0	0
vith concret	Above 15 p	3	0	0	1	0	0	0
flush toilet	1-5 people	1 3	1	0	1	0	0	0
flush toilet	Above 15 p	1 2 4	1	1	0	1	0	0
onâ€™t kno	Above 15 p	1 2 3 4 5 6	1	1	1	1	1	1
flush toilet	1-5 people	1	1	0	0	0	0	0
flush toilet	6-10 peopl	3	0	0	1	0	0	0

flush toilet	Above 15 p	1 4	1	0	0	1	0	0
vith concret	Above 15 p	3	0	0	1	0	0	0
flush toilet	11-15 peop	1 4	1	0	0	1	0	0
vith concret	Above 15 p	3	0	0	1	0	0	0
flush toilet	Do not sha	1	1	0	0	0	0	0
flush toilet	6-10 peopl	1	1	0	0	0	0	0
flush toilet	1-5 people	1	1	0	0	0	0	0
flush toilet	Above 15 p	1 3 4	1	0	1	1	0	0
flush toilet	1-5 people	3	0	0	1	0	0	0
vith concret	Above 15 p	2 4 5 6 7	0	1	0	1	1	1
vith concret	Above 15 p	3 5	0	0	1	0	1	0
vith concret	Above 15 p	3	0	0	1	0	0	0
vith concret	Above 15 p	1 3	1	0	1	0	0	0
vith concret	6-10 peopl	1	1	0	0	0	0	0
vith concret	11-15 peop	4	0	0	0	1	0	0
vith concret	Above 15 p	1 2 3	1	1	1	0	0	0
flush toilet	1-5 people	1	1	0	0	0	0	0
flush toilet	Do not sha	1	1	0	0	0	0	0
vith concret	Above 15 p	3 4 6	0	0	1	1	0	1
flush toilet	6-10 peopl	1 3	1	0	1	0	0	0
flush toilet	6-10 peopl	3	0	0	1	0	0	0
flush toilet	Above 15 p	1 3 4	1	0	1	1	0	0
flush toilet	Above 15 p	1 3 4 5	1	0	1	1	1	0
vith concret	6-10 peopl	3	0	0	1	0	0	0
vith concret	11-15 peop	1 2 4 5	1	1	0	1	1	0
vith concret	Above 15 p	3	0	0	1	0	0	0
vith concret	6-10 peopl	1 2	1	1	0	0	0	0
vith concret	6-10 peopl	1 3 4	1	0	1	1	0	0
flush toilet	6-10 peopl	3	0	0	1	0	0	0
flush toilet	6-10 peopl	1 3	1	0	1	0	0	0
vith concret	6-10 peopl	1 2 3	1	1	1	0	0	0
flush toilet	11-15 peop	1 3	1	0	1	0	0	0
flush toilet	Above 15 p	1 3 4 5	1	0	1	1	1	0
flush toilet	Above 15 p	1 3 4 5	1	0	1	1	1	0
flush toilet	Above 15 p	3	0	0	1	0	0	0
vithout slab	Above 15 p	1 3 5	1	0	1	0	1	0
flush toilet	Donâ€™t k	1 3	1	0	1	0	0	0
vith concret	Above 15 p	1 3	1	0	1	0	0	0
flush toilet	Above 15 p	4 5	0	0	0	1	1	0
vith concret	Above 15 p	2 3 5	0	1	1	0	1	0
flush toilet	1-5 people	1 2	1	1	0	0	0	0
vith concret	Above 15 p	1 2 3 4 5 6	1	1	1	1	1	1
flush toilet	11-15 peop	4	0	0	0	1	0	0
flush toilet	Above 15 p	1 3 4	1	0	1	1	0	0
vith concret	Above 15 p	1 2 4	1	1	0	1	0	0
flush toilet	1-5 people	1	1	0	0	0	0	0
NA	1-5 people	3 6	0	0	1	0	0	1
flush toilet	6-10 peopl	1	1	0	0	0	0	0
flush toilet	Above 15 p	1 3 4 5 6 7	1	0	1	1	1	1

vith concret	Above 15 p	1 4 5		1	0	0	1	1	0
vith concret	11-15 peop	1 3		1	0	1	0	0	0
flush toilet	6-10 peopl		4	0	0	0	1	0	0
vith concret	Above 15 p	1 3 4		1	0	1	1	0	0
flush toilet	11-15 peop	1 3 4		1	0	1	1	0	0
flush toilet	Above 15 p	1 2 3 4		1	1	1	1	0	0
vith concret	11-15 peop	3 4		0	0	1	1	0	0
vith concret	11-15 peop	1 3		1	0	1	0	0	0
flush toilet	1-5 people		1	1	0	0	0	0	0
flush toilet	Above 15 p	1 3 4		1	0	1	1	0	0
vith concret	Above 15 p	1 2		1	1	0	0	0	0
vith concret	11-15 peop	1 3 4 5 6		1	0	1	1	1	1
flush toilet	Above 15 p	1 3 4		1	0	1	1	0	0
vith concret	Above 15 p		3	0	0	1	0	0	0
flush toilet	1-5 people		1	1	0	0	0	0	0
flush toilet	Above 15 p	1 3 4		1	0	1	1	0	0
vithout slab	Above 15 p	1 3 4		1	0	1	1	0	0
vith concret	Do not sha		1	1	0	0	0	0	0
flush toilet	Above 15 p		3	0	0	1	0	0	0
flush toilet	Above 15 p	1 3		1	0	1	0	0	0
flush toilet	11-15 peop	1 3		1	0	1	0	0	0
vith concret	Above 15 p	1 2 3 4 5 7		1	1	1	1	1	0
onâ€™t kno	11-15 peop	1 3		1	0	1	0	0	0
vith concret	11-15 peop		3	0	0	1	0	0	0
vith concret	6-10 peopl	1 3 5		1	0	1	0	1	0
vith concret	Donâ€™t k	1 3		1	0	1	0	0	0
flush toilet	6-10 peopl		1	1	0	0	0	0	0
flush toilet	1-5 people	1 3		1	0	1	0	0	0
vithout slab	6-10 peopl	1 2		1	1	0	0	0	0
flush toilet	1-5 people		1	1	0	0	0	0	0
vith concret	Above 15 p	1 3 4 7		1	0	1	1	0	0
flush toilet	1-5 people		1	1	0	0	0	0	0
flush toilet	11-15 peop		4	0	0	0	1	0	0
vith concret	Above 15 p	1 3 5 6		1	0	1	0	1	1
vith concret	6-10 peopl	1 3		1	0	1	0	0	0
flush toilet	Above 15 p	1 2 3		1	1	1	0	0	0
flush toilet	11-15 peop	1 2 3		1	1	1	0	0	0
flush toilet	Above 15 p	1 3 4		1	0	1	1	0	0
vith concret	6-10 peopl	1 2 3		1	1	1	0	0	0
vith concret	Do not sha		1	1	0	0	0	0	0
vith concret	Above 15 p	1 2 3		1	1	1	0	0	0
flush toilet	6-10 peopl		1	1	0	0	0	0	0
vith concret	Above 15 p		3	0	0	1	0	0	0
vith concret	Above 15 p		3	0	0	1	0	0	0
vith concret	Above 15 p	1 3		1	0	1	0	0	0
flush toilet	11-15 peop		3	0	0	1	0	0	0
vith concret	Above 15 p	1 3 4 5 6		1	0	1	1	1	1
vith concret	11-15 peop		3	0	0	1	0	0	0
vith concret	Above 15 p		3	0	0	1	0	0	0

flush toilet	6-10 peopl	1 2		1	1	0	0	0	0
flush toilet	11-15 peop	1 3 4		1	0	1	1	0	0
vith concret	1-5 people		1	1	0	0	0	0	0
vith concret	11-15 peop		1	1	0	0	0	0	0
flush toilet	Above 15 p	1 3 4 5		1	0	1	1	1	0
flush toilet	Above 15 p	1 2		1	1	0	0	0	0
flush toilet	11-15 peop	1 3		1	0	1	0	0	0
flush toilet	Above 15 p	1 3 4		1	0	1	1	0	0
vith concret	1-5 people	1 2 3 4 6		1	1	1	1	0	1
vith concret	6-10 peopl	3 4 5		0	0	1	1	1	0
vith concret	Above 15 p	1 4 5 6		1	0	0	1	1	1
vith concret	Above 15 p	1 3 4 6		1	0	1	1	0	1
flush toilet	1-5 people		1	1	0	0	0	0	0
flush toilet	6-10 peopl		4	0	0	0	1	0	0
vith concret	Above 15 p	1 3 4		1	0	1	1	0	0
flush toilet	Above 15 p	1 3 4 5		1	0	1	1	1	0
flush toilet	6-10 peopl		1	1	0	0	0	0	0
vith concret	1-5 people		3	0	0	1	0	0	0
vith concret	6-10 peopl	1 3		1	0	1	0	0	0
flush toilet	Above 15 p	1 3		1	0	1	0	0	0
vith concret	Above 15 p	3 6		0	0	1	0	0	1
flush toilet	6-10 peopl		1	1	0	0	0	0	0
oush/field	Donâ€™t k		96	0	0	0	0	0	0
vith concret	Above 15 p		3	0	0	1	0	0	0
vithout slab	11-15 peop	1 3 5		1	0	1	0	1	0
oush/field	Donâ€™t k		1	1	0	0	0	0	0
vith concret	11-15 peop	1 3		1	0	1	0	0	0
vith concret	11-15 peop	1 3 5 6		1	0	1	0	1	1
flush toilet	Above 15 p	1 2 3 4		1	1	1	1	0	0
flush toilet	1-5 people		1	1	0	0	0	0	0
vith concret	Donâ€™t k	1 3 7		1	0	1	0	0	0
flush toilet	6-10 peopl	1 2		1	1	0	0	0	0
vith concret	Above 15 p	1 3 4 5 6 7		1	0	1	1	1	1
flush toilet	6-10 peopl		1	1	0	0	0	0	0
vith concret	Above 15 p		4	0	0	0	1	0	0
flush toilet	1-5 people		1	1	0	0	0	0	0
flush toilet	Above 15 p	4 5		0	0	0	1	1	0
vith concret	1-5 people		1	1	0	0	0	0	0
flush toilet	11-15 peop	1 3 4		1	0	1	1	0	0
vith concret	11-15 peop	3 4 5		0	0	1	1	1	0
flush toilet	1-5 people		1	1	0	0	0	0	0
flush toilet	1-5 people		1	1	0	0	0	0	0
flush toilet	6-10 peopl		1	1	0	0	0	0	0
flush toilet	6-10 peopl		3	0	0	1	0	0	0
vith concret	1-5 people	1 2		1	1	0	0	0	0
vith concret	Above 15 p	1 2 3 4 5		1	1	1	1	1	0
flush toilet	6-10 peopl		4	0	0	0	1	0	0
flush toilet	11-15 peop	1 3 4 5		1	0	1	1	1	0
vith concret	Above 15 p	1 2 3		1	1	1	0	0	0

flush toilet	11-15 peop	1 3		1	0	1	0	0	0
flush toilet	1-5 people	1 3		1	0	1	0	0	0
vith concret	Above 15 p	1 2 3 4 5 6		1	1	1	1	1	1
flush toilet	11-15 peop	1 3		1	0	1	0	0	0
vith concret	11-15 peop	1 3		1	0	1	0	0	0
flush toilet	1-5 people		1	1	0	0	0	0	0
flush toilet	11-15 peop		1	1	0	0	0	0	0
flush toilet	1-5 people	1 2 3		1	1	1	0	0	0
vith concret	Above 15 p	1 2 3 4		1	1	1	1	0	0
flush toilet	Above 15 p	1 3 4		1	0	1	1	0	0
flush toilet	6-10 peopl	1 3		1	0	1	0	0	0
flush toilet	Above 15 p	1 3 4		1	0	1	1	0	0
flush toilet	6-10 peopl		4	0	0	0	1	0	0
flush toilet	Above 15 p	1 3 4 5 7		1	0	1	1	1	0
vith concret	1-5 people	1 3 4		1	0	1	1	0	0
vith concret	Above 15 p	1 2 4		1	1	0	1	0	0
vith concret	Donâ€™t k	1 3 4		1	0	1	1	0	0
vith concret	Above 15 p	1 3 4 5 6		1	0	1	1	1	1
flush toilet	6-10 peopl	1 3		1	0	1	0	0	0
vith concret	6-10 peopl	1 2 3 4 5		1	1	1	1	1	0
vith concret	11-15 peop		1	1	0	0	0	0	0
flush toilet	Above 15 p	1 3 4		1	0	1	1	0	0
flush toilet	Above 15 p	1 3 4		1	0	1	1	0	0
flush toilet	1-5 people		1	1	0	0	0	0	0
flush toilet	Above 15 p	1 3 4 5		1	0	1	1	1	0
flush toilet	1-5 people		1	1	0	0	0	0	0
flush toilet	6-10 peopl	1 3		1	0	1	0	0	0
vith concret	Above 15 p	1 3		1	0	1	0	0	0
vith concret	Above 15 p	3 4 5 6		0	0	1	1	1	1
vith concret	Above 15 p		3	0	0	1	0	0	0
vith concret	Above 15 p	1 2 3 5		1	1	1	0	1	0
vith concret	6-10 peopl	1 3		1	0	1	0	0	0
vithout slab	11-15 peop	1 2 3		1	1	1	0	0	0
flush toilet	11-15 peop	1 3 4 6		1	0	1	1	0	1
flush toilet	Above 15 p	1 3 4		1	0	1	1	0	0
flush toilet	6-10 peopl	1 3		1	0	1	0	0	0
vith concret	11-15 peop		3	0	0	1	0	0	0
flush toilet	Above 15 p	1 3		1	0	1	0	0	0
flush toilet	Above 15 p		4	0	0	0	1	0	0
vith concret	Above 15 p	1 3 4		1	0	1	1	0	0
flush toilet	1-5 people		1	1	0	0	0	0	0
vith concret	6-10 peopl	1 3		1	0	1	0	0	0
flush toilet	Above 15 p	1 3 4		1	0	1	1	0	0
flush toilet	6-10 peopl	1 2		1	1	0	0	0	0
vith concret	Above 15 p	1 4		1	0	0	1	0	0
vith concret	Above 15 p	1 3 5		1	0	1	0	1	0
flush toilet	11-15 peop	1 4		1	0	0	1	0	0
flush toilet	1-5 people		1	1	0	0	0	0	0
flush toilet	1-5 people		1	1	0	0	0	0	0

flush toilet	Above 15 p	3 7	0	0	1	0	0	0
flush toilet	Above 15 p	1 3 5	1	0	1	0	1	0
flush toilet	1-5 people	1	1	0	0	0	0	0
vith concret	6-10 peopl	1 4 5	1	0	0	1	1	0
vithout slab	Above 15 p	1 3 5	1	0	1	0	1	0
vith concret	Above 15 p	3	0	0	1	0	0	0
flush toilet	Above 15 p	1 3 4	1	0	1	1	0	0
onâ€™t kno	1-5 people	1	1	0	0	0	0	0
vith concret	1-5 people	3	0	0	1	0	0	0
flush toilet	11-15 peop	1 3	1	0	1	0	0	0
vith concret	Above 15 p	1 3	1	0	1	0	0	0
vith concret	Donâ€™t k	1 3 7	1	0	1	0	0	0
flush toilet	1-5 people	1	1	0	0	0	0	0
flush toilet	6-10 peopl	1	1	0	0	0	0	0
flush toilet	Above 15 p	1 3 4 5 6 7	1	0	1	1	1	1
vith concret	6-10 peopl	1 3 4	1	0	1	1	0	0
flush toilet	6-10 peopl	1 3	1	0	1	0	0	0
vith concret	6-10 peopl	1	1	0	0	0	0	0
flush toilet	Above 15 p	4	0	0	0	1	0	0
flush toilet	Donâ€™t k	1 3	1	0	1	0	0	0
vith concret	Above 15 p	1 3 4 6 7	1	0	1	1	0	1
vith concret	11-15 peop	3	0	0	1	0	0	0
flush toilet	6-10 peopl	1	1	0	0	0	0	0
flush toilet	Do not sha	1	1	0	0	0	0	0
flush toilet	Above 15 p	1 3 4 5	1	0	1	1	1	0
flush toilet	Above 15 p	1 3 4 5 6	1	0	1	1	1	1
flush toilet	Above 15 p	3	0	0	1	0	0	0
flush toilet	6-10 peopl	4	0	0	0	1	0	0
flush toilet	6-10 peopl	1 3	1	0	1	0	0	0
flush toilet	6-10 peopl	1 3	1	0	1	0	0	0
flush toilet	Above 15 p	1 3	1	0	1	0	0	0
flush toilet	1-5 people	1	1	0	0	0	0	0
flush toilet	1-5 people	1	1	0	0	0	0	0
Flush/pour	Above 15 p	1 3 4 5 7	1	0	1	1	1	0
flush toilet	Do not sha	96	0	0	0	0	0	0
flush toilet	Above 15 p	1 3 4 5 6 7	1	0	1	1	1	1
flush toilet	11-15 peop	1 3 4	1	0	1	1	0	0
vith concret	6-10 peopl	2 4 5 6	0	1	0	1	1	1
flush toilet	6-10 peopl	1	1	0	0	0	0	0
vith concret	6-10 peopl	1 2 4	1	1	0	1	0	0
flush toilet	1-5 people	1	1	0	0	0	0	0
flush toilet	1-5 people	1	1	0	0	0	0	0
vith concret	Above 15 p	3	0	0	1	0	0	0
flush toilet	1-5 people	1	1	0	0	0	0	0
flush toilet	Above 15 p	3 4	0	0	1	1	0	0
onâ€™t kno	11-15 peop	1 2 3	1	1	1	0	0	0
vith concret	1-5 people	1	1	0	0	0	0	0
vith concret	Donâ€™t k	1 3 4 5 7	1	0	1	1	1	0
flush toilet	Above 15 p	1 3 4	1	0	1	1	0	0

flush toilet	6-10 peopl		4	0	0	0	1	0	0
flush toilet	Above 15 p	1 3		1	0	1	0	0	0
vith concret	Above 15 p	1 2 3 6		1	1	1	0	0	1
flush toilet	11-15 peop	1 3 4		1	0	1	1	0	0
flush toilet	6-10 peopl		1	1	0	0	0	0	0
vithout slab	6-10 peopl		1	1	0	0	0	0	0
vith concret	Above 15 p	1 3		1	0	1	0	0	0
vith concret	Above 15 p	3 4 5		0	0	1	1	1	0
vith concret	Above 15 p	1 3 4 7		1	0	1	1	0	0
vith concret	11-15 peop	1 2 3 5 6		1	1	1	0	1	1
flush toilet	6-10 peopl		1	1	0	0	0	0	0
vith concret	Above 15 p	2 3 5 6		0	1	1	0	1	1
vith concret	Do not sha	1 2		1	1	0	0	0	0
vith concret	6-10 peopl	2 4 6		0	1	0	1	0	1
vith concret	Above 15 p	1 3 4 5 6 7		1	0	1	1	1	1
vith concret	11-15 peop	1 3 6		1	0	1	0	0	1
flush toilet	Above 15 p	1 3 4		1	0	1	1	0	0
flush toilet	Above 15 p		3	0	0	1	0	0	0
flush toilet	Above 15 p	1 3 4		1	0	1	1	0	0
vith concret	6-10 peopl	1 3		1	0	1	0	0	0
onâ€™t kno	Above 15 p	1 3 5		1	0	1	0	1	0
vith concret	Above 15 p	1 3 4		1	0	1	1	0	0
vith concret	Above 15 p	1 3		1	0	1	0	0	0
vith concret	11-15 peop	1 2 3 4		1	1	1	1	0	0
flush toilet	Above 15 p	1 3 4		1	0	1	1	0	0
flush toilet	Above 15 p	1 3 4		1	0	1	1	0	0
flush toilet	Above 15 p	1 3 4		1	0	1	1	0	0
flush toilet	6-10 peopl		1	1	0	0	0	0	0
flush toilet	Above 15 p	1 2 3 4 5		1	1	1	1	1	0
vith concret	Above 15 p		3	0	0	1	0	0	0
vith concret	11-15 peop	1 2 3 4		1	1	1	1	0	0
vith concret	Above 15 p	1 3 4 6 7		1	0	1	1	0	1
flush toilet	Above 15 p		4	0	0	0	1	0	0
vith concret	Above 15 p	1 2 4 6		1	1	0	1	0	1
flush toilet	6-10 peopl	1 2 3		1	1	1	0	0	0
flush toilet	1-5 people		1	1	0	0	0	0	0
vith concret	11-15 peop	1 2 4 5 6		1	1	0	1	1	1
vith concret	Above 15 p	1 3 4		1	0	1	1	0	0
flush toilet	6-10 peopl	1 3		1	0	1	0	0	0
vith concret	Above 15 p	1 2 4		1	1	0	1	0	0
flush toilet	Above 15 p	1 3 4		1	0	1	1	0	0
vithout slab	6-10 peopl		1	1	0	0	0	0	0
vith concret	Donâ€™t k	1 3		1	0	1	0	0	0
vith concret	11-15 peop	1 2 3		1	1	1	0	0	0
flush toilet	Above 15 p	1 3 4		1	0	1	1	0	0
flush toilet	6-10 peopl	1 2 3		1	1	1	0	0	0
vith concret	Above 15 p	1 2 3 5		1	1	1	0	1	0
vith concret	Above 15 p	1 2 3		1	1	1	0	0	0
flush toilet	1-5 people		1	1	0	0	0	0	0

vithout slab	1-5 people	1 2	1	1	0	0	0	0
vith concret	1-5 people	1 2	1	1	0	0	0	0
vithout slab	Above 15 p	1 3 4	1	0	1	1	0	0
flush toilet	Above 15 p	1 3 4	1	0	1	1	0	0
vith concret	Above 15 p	1 3 4 5	1	0	1	1	1	0
vithout slab	6-10 peopl	1	1	0	0	0	0	0
flush toilet	11-15 peop	1 2 3	1	1	1	0	0	0
flush toilet	6-10 peopl	1 3 4	1	0	1	1	0	0
flush toilet	Do not sha	1	1	0	0	0	0	0
vith concret	6-10 peopl	3	0	0	1	0	0	0
flush toilet	1-5 people	1	1	0	0	0	0	0
improved pi	11-15 peop	1 2 3	1	1	1	0	0	0
flush toilet	6-10 peopl	1 3	1	0	1	0	0	0
Pit latrine v	6-10 peopl	1	1	0	0	0	0	0
nâ€™t kno	1-5 people	1	1	0	0	0	0	0
flush toilet	11-15 peop	1 3	1	0	1	0	0	0
flush toilet	6-10 peopl	3 4	0	0	1	1	0	0
vith concret	Above 15 p	2 3 5 6	0	1	1	0	1	1
vith concret	11-15 peop	1 3 4	1	0	1	1	0	0
vith concret	Above 15 p	3	0	0	1	0	0	0
vith concret	Above 15 p	1 3 4	1	0	1	1	0	0
flush toilet	Above 15 p	1 3 4 5	1	0	1	1	1	0
flush toilet	6-10 peopl	1 3	1	0	1	0	0	0
vith concret	Above 15 p	1 2 3 4	1	1	1	1	0	0
vith concret	11-15 peop	2 3	0	1	1	0	0	0
vith concret	11-15 peop	1 2 3 4	1	1	1	1	0	0
flush toilet	11-15 peop	3	0	0	1	0	0	0
flush toilet	11-15 peop	1 3	1	0	1	0	0	0
flush toilet	6-10 peopl	1 4	1	0	0	1	0	0
flush toilet	Above 15 p	1 3 4 5	1	0	1	1	1	0
vith concret	Above 15 p	3	0	0	1	0	0	0
flush toilet	Above 15 p	1 3 4	1	0	1	1	0	0
flush toilet	Above 15 p	4	0	0	0	1	0	0
flush toilet	1-5 people	1	1	0	0	0	0	0
vith concret	Above 15 p	2 4 6	0	1	0	1	0	1
vith concret	Above 15 p	1 2 3 4 5 6	1	1	1	1	1	1
flush toilet	1-5 people	1	1	0	0	0	0	0
flush toilet	Above 15 p	1 3 4	1	0	1	1	0	0
vith concret	Above 15 p	1 3 6	1	0	1	0	0	1
flush toilet	11-15 peop	3	0	0	1	0	0	0
vith concret	Above 15 p	1 2 3	1	1	1	0	0	0
flush toilet	Above 15 p	4 7 96	0	0	0	1	0	0
vith concret	Above 15 p	1 3	1	0	1	0	0	0
flush toilet	6-10 peopl	1 3	1	0	1	0	0	0
flush toilet	Above 15 p	1 3 4 5	1	0	1	1	1	0
flush toilet	11-15 peop	1 3	1	0	1	0	0	0
vith concret	6-10 peopl	1 2	1	1	0	0	0	0
vith concret	Above 15 p	1 2 3	1	1	1	0	0	0
flush toilet	11-15 peop	1 3	1	0	1	0	0	0

flush toilet	Above 15 p	1 3 4 5	1	0	1	1	1	0
vith concret	Above 15 p	1 3 6	1	0	1	0	0	1
flush toilet	6-10 peopl	3	0	0	1	0	0	0
flush toilet	1-5 people	3	0	0	1	0	0	0
vith concret	Above 15 p	1 3 6	1	0	1	0	0	1
vith concret	11-15 peop	3 5	0	0	1	0	1	0
flush toilet	Donâ€™t k	1 3	1	0	1	0	0	0
vith concret	6-10 peopl	1 3 6	1	0	1	0	0	1
flush toilet	6-10 peopl	1 3 4	1	0	1	1	0	0
vithout slab	Above 15 p	1 2 3 4 5 6	1	1	1	1	1	1
vith concret	11-15 peop	3	0	0	1	0	0	0
flush toilet	Above 15 p	1 3 4 5	1	0	1	1	1	0
flush toilet	6-10 peopl	1	1	0	0	0	0	0
flush toilet	11-15 peop	1 3 4	1	0	1	1	0	0
flush toilet	Above 15 p	3	0	0	1	0	0	0
vith concret	Above 15 p	1 3 4	1	0	1	1	0	0
vith concret	11-15 peop	1 3	1	0	1	0	0	0
vith concret	Above 15 p	1 3	1	0	1	0	0	0
flush toilet	Above 15 p	3	0	0	1	0	0	0
flush toilet	1-5 people	1	1	0	0	0	0	0
flush toilet	Donâ€™t k	1 3 4 7	1	0	1	1	0	0
flush toilet	6-10 peopl	1	1	0	0	0	0	0
flush toilet	1-5 people	1	1	0	0	0	0	0
vith concret	Above 15 p	1 3 4 5	1	0	1	1	1	0
flush toilet	Above 15 p	3 4 5	0	0	1	1	1	0
flush toilet	Above 15 p	1 3	1	0	1	0	0	0
vith concret	6-10 peopl	1	1	0	0	0	0	0
flush toilet	Above 15 p	1 2 3 4	1	1	1	1	0	0
flush toilet	Above 15 p	4	0	0	0	1	0	0
flush toilet	11-15 peop	3	0	0	1	0	0	0
flush toilet	Above 15 p	1 3	1	0	1	0	0	0
vith concret	6-10 peopl	1 3	1	0	1	0	0	0
vithout slab	Above 15 p	1 3	1	0	1	0	0	0
flush toilet	Above 15 p	3	0	0	1	0	0	0
flush toilet	11-15 peop	1 2 3 4	1	1	1	1	0	0
flush toilet	6-10 peopl	1 3	1	0	1	0	0	0
vith concret	Above 15 p	3	0	0	1	0	0	0
vith concret	Above 15 p	1 4 5	1	0	0	1	1	0
flush toilet	Above 15 p	3	0	0	1	0	0	0
flush toilet	6-10 peopl	1 3	1	0	1	0	0	0
vith concret	11-15 peop	1 2 3 4 5 6	1	1	1	1	1	1
flush toilet	Above 15 p	1 3 4	1	0	1	1	0	0
flush toilet	Above 15 p	1 2 3 4	1	1	1	1	0	0
flush toilet	11-15 peop	1 2 3 4	1	1	1	1	0	0
flush toilet	Above 15 p	1 3	1	0	1	0	0	0
vith concret	Above 15 p	1 3	1	0	1	0	0	0
flush toilet	Above 15 p	3	0	0	1	0	0	0
flush toilet	Above 15 p	1 3 4 5 6 7	1	0	1	1	1	1
vith concret	11-15 peop	1 3 6	1	0	1	0	0	1

vith concret	Above 15 p	3	0	0	1	0	0	0
vith concret	6-10 peopl	1	1	0	0	0	0	0
vith concret	Above 15 p	3	0	0	1	0	0	0
flush toilet	11-15 peop	1 4 5	1	0	0	1	1	0
flush toilet	Above 15 p	4	0	0	0	1	0	0
vith concret	11-15 peop	1 3 5	1	0	1	0	1	0
vith concret	6-10 peopl	4	0	0	0	1	0	0
flush toilet	11-15 peop	3 6	0	0	1	0	0	1
vith concret	Above 15 p	2 3 5	0	1	1	0	1	0
vith concret	11-15 peop	3	0	0	1	0	0	0
flush toilet	11-15 peop	1 3 4	1	0	1	1	0	0
flush toilet	Above 15 p	1 3 4	1	0	1	1	0	0
vith concret	Above 15 p	1 2 3	1	1	1	0	0	0
flush toilet	Above 15 p	3	0	0	1	0	0	0
vith concret	11-15 peop	1 3	1	0	1	0	0	0
vith concret	Above 15 p	2 3 4 5	0	1	1	1	1	0
flush toilet	Above 15 p	3	0	0	1	0	0	0
vith concret	1-5 people	1	1	0	0	0	0	0
vith concret	6-10 peopl	3	0	0	1	0	0	0
flush toilet	6-10 peopl	1 3	1	0	1	0	0	0
vith concret	11-15 peop	1 3 4	1	0	1	1	0	0
flush toilet	6-10 peopl	3	0	0	1	0	0	0
vith concret	Above 15 p	1 2 3 4 5	1	1	1	1	1	0
flush toilet	1-5 people	1	1	0	0	0	0	0
flush toilet	6-10 peopl	1 3	1	0	1	0	0	0
vith concret	Above 15 p	1 2 3 4 5 6	1	1	1	1	1	1
flush toilet	Above 15 p	4	0	0	0	1	0	0
vith concret	Above 15 p	2 4 5	0	1	0	1	1	0
flush toilet	Above 15 p	1 4	1	0	0	1	0	0
vith concret	11-15 peop	3	0	0	1	0	0	0
vith concret	Above 15 p	1 3 4 5 6	1	0	1	1	1	1
flush toilet	Above 15 p	3	0	0	1	0	0	0
flush toilet	Above 15 p	1 3	1	0	1	0	0	0
flush toilet	Above 15 p	1 3 4	1	0	1	1	0	0
vith concret	Above 15 p	1 3	1	0	1	0	0	0
flush toilet	6-10 peopl	1 3 4	1	0	1	1	0	0
vith concret	Above 15 p	1 2 3	1	1	1	0	0	0
flush toilet	Above 15 p	4	0	0	0	1	0	0
vith concret	11-15 peop	1 3	1	0	1	0	0	0
flush toilet	Do not sha	96	0	0	0	0	0	0
flush toilet	Above 15 p	1 2 3 4	1	1	1	1	0	0
vith concret	Above 15 p	1 2 3	1	1	1	0	0	0
vith concret	Above 15 p	1 3 6	1	0	1	0	0	1
flush toilet	Above 15 p	1 3 6	1	0	1	0	0	1
flush toilet	6-10 peopl	1	1	0	0	0	0	0
vith concret	6-10 peopl	1 3	1	0	1	0	0	0
flush toilet	6-10 peopl	1	1	0	0	0	0	0
flush toilet	1-5 people	1	1	0	0	0	0	0
vith concret	6-10 peopl	2 3 4 6	0	1	1	1	0	1

vithout slab	11-15 peop	3	0	0	1	0	0	0
flush toilet	11-15 peop	4	0	0	0	1	0	0
vith concret	Above 15 p	3 5	0	0	1	0	1	0
flush toilet	6-10 peopl	1 3	1	0	1	0	0	0
flush toilet	1-5 people	1	1	0	0	0	0	0
flush toilet	6-10 peopl	1 2	1	1	0	0	0	0
flush toilet	Above 15 p	4	0	0	0	1	0	0
flush toilet	1-5 people	1 3	1	0	1	0	0	0
vith concret	1-5 people	1 2 3 4	1	1	1	1	0	0
vith concret	6-10 peopl	1 3	1	0	1	0	0	0
flush toilet	11-15 peop	3	0	0	1	0	0	0
flush toilet	11-15 peop	1 3	1	0	1	0	0	0
flush toilet	Above 15 p	1 3	1	0	1	0	0	0
flush toilet	Above 15 p	3	0	0	1	0	0	0
vith concret	Above 15 p	3	0	0	1	0	0	0
vith concret	11-15 peop	1 4	1	0	0	1	0	0
flush toilet	6-10 peopl	1 3	1	0	1	0	0	0
vithout slab	Above 15 p	1 2 3 4 5 6	1	1	1	1	1	1
vith concret	6-10 peopl	1 5	1	0	0	0	1	0
flush toilet	11-15 peop	1 3	1	0	1	0	0	0
flush toilet	1-5 people	1	1	0	0	0	0	0
flush toilet	Above 15 p	1 3 4 5	1	0	1	1	1	0
flush toilet	Above 15 p	1 3 4	1	0	1	1	0	0
vith concret	Above 15 p	1 3 4 5	1	0	1	1	1	0
vith concret	Above 15 p	3	0	0	1	0	0	0
vith concret	Above 15 p	3	0	0	1	0	0	0
flush toilet	Above 15 p	1 3 4	1	0	1	1	0	0
flush toilet	6-10 peopl	3	0	0	1	0	0	0
flush toilet	Above 15 p	3	0	0	1	0	0	0
flush toilet	Above 15 p	1 3 4	1	0	1	1	0	0
flush toilet	Above 15 p	3	0	0	1	0	0	0
flush toilet	6-10 peopl	1 2	1	1	0	0	0	0
flush toilet	Above 15 p	1 3 4	1	0	1	1	0	0
flush toilet	6-10 peopl	3	0	0	1	0	0	0
flush toilet	1-5 people	1	1	0	0	0	0	0
vith concret	Above 15 p	1 3	1	0	1	0	0	0
vithout slab	6-10 peopl	1	1	0	0	0	0	0
vith concret	11-15 peop	1 3 5	1	0	1	0	1	0
vith concret	11-15 peop	1 2 3 5 6	1	1	1	0	1	1
flush toilet	Do not sha	96	0	0	0	0	0	0
flush toilet	11-15 peop	1	1	0	0	0	0	0
vith concret	Above 15 p	3 4 6	0	0	1	1	0	1
flush toilet	6-10 peopl	1 3	1	0	1	0	0	0
flush toilet	Above 15 p	1 2 3 4	1	1	1	1	0	0
flush toilet	1-5 people	1	1	0	0	0	0	0
vith concret	Above 15 p	1 2 4	1	1	0	1	0	0
flush toilet	Do not sha	1	1	0	0	0	0	0
vith concret	Above 15 p	1 2 3 4	1	1	1	1	0	0
flush toilet	Above 15 p	1 2 3	1	1	1	0	0	0

flush toilet	Above 15 p	1 3 4 5	1	0	1	1	1	0
flush toilet	11-15 peop	1	1	0	0	0	0	0
flush toilet	Donâ€™t k	1 3 4	1	0	1	1	0	0
flush toilet	1-5 people	1	1	0	0	0	0	0
flush toilet	6-10 peopl	1 2	1	1	0	0	0	0
onâ€™t kno	1-5 people	1 3	1	0	1	0	0	0
flush toilet	Above 15 p	1 7	1	0	0	0	0	0
vith concret	11-15 peop	2 3 4	0	1	1	1	0	0
flush toilet	6-10 peopl	1 3	1	0	1	0	0	0
vithout slab	6-10 peopl	1	1	0	0	0	0	0
flush toilet	11-15 peop	1 3	1	0	1	0	0	0
vith concret	Above 15 p	4	0	0	0	1	0	0
flush toilet	1-5 people	1	1	0	0	0	0	0
vith concret	Above 15 p	2 3 4	0	1	1	1	0	0
flush toilet	Above 15 p	1 3 4 5	1	0	1	1	1	0
vith concret	Above 15 p	1 3 4 6	1	0	1	1	0	1
flush toilet	6-10 peopl	1	1	0	0	0	0	0
vithout slab	Above 15 p	1 3 4	1	0	1	1	0	0
flush toilet	Above 15 p	1 3 4	1	0	1	1	0	0
vith concret	11-15 peop	1 2 6	1	1	0	0	0	1
flush toilet	1-5 people	1	1	0	0	0	0	0
vith concret	6-10 peopl	1 2 3	1	1	1	0	0	0
flush toilet	1-5 people	1	1	0	0	0	0	0
vith concret	6-10 peopl	1	1	0	0	0	0	0
vith concret	Above 15 p	1 3 4 5 7	1	0	1	1	1	0
vith concret	Above 15 p	1 3 4 5 6 7	1	0	1	1	1	1
flush toilet	Above 15 p	1 3 4	1	0	1	1	0	0
vith concret	Above 15 p	1 3 4 7	1	0	1	1	0	0
vith concret	Above 15 p	3	0	0	1	0	0	0
vith concret	Above 15 p	1 2 3 4 5 6	1	1	1	1	1	1
vith concret	Above 15 p	1 4 6	1	0	0	1	0	1
vith concret	1-5 people	1 2 4 6	1	1	0	1	0	1
flush toilet	Above 15 p	1 3 4 5	1	0	1	1	1	0
vith concret	Above 15 p	1 3 6	1	0	1	0	0	1
vith concret	Above 15 p	1 2 3 4 6 7	1	1	1	1	0	1
vith concret	Above 15 p	3	0	0	1	0	0	0
flush toilet	6-10 peopl	1	1	0	0	0	0	0
flush toilet	Above 15 p	1 3	1	0	1	0	0	0
vith concret	Donâ€™t k	1 3 4	1	0	1	1	0	0
Responder	Above 15 p	1 3	1	0	1	0	0	0
flush toilet	Above 15 p	3	0	0	1	0	0	0
vith concret	6-10 peopl	1	1	0	0	0	0	0
vith concret	11-15 peop	1 3	1	0	1	0	0	0
vith concret	6-10 peopl	1 2 3 4 5 6	1	1	1	1	1	1
vith concret	6-10 peopl	3	0	0	1	0	0	0
vith concret	6-10 peopl	1 3 4	1	0	1	1	0	0
flush toilet	6-10 peopl	1	1	0	0	0	0	0
flush toilet	Above 15 p	1 3 4	1	0	1	1	0	0
flush toilet	Above 15 p	1 3 4 5 6 7	1	0	1	1	1	1

vith concret	Above 15 p	1 3 4 5	1	0	1	1	1	0
vith concret	Above 15 p	1 2 3 4 5 6	1	1	1	1	1	1
flush toilet	6-10 peopl	1	1	0	0	0	0	0
flush toilet	6-10 peopl	1 3 4	1	0	1	1	0	0
vith concret	11-15 peop	2 3 6	0	1	1	0	0	1
flush toilet	Above 15 p	4	0	0	0	1	0	0
flush toilet	6-10 peopl	1 3	1	0	1	0	0	0
flush toilet	Above 15 p	4	0	0	0	1	0	0
flush toilet	6-10 peopl	1	1	0	0	0	0	0
vith concret	11-15 peop	1 3	1	0	1	0	0	0
flush toilet	11-15 peop	2 3 4	0	1	1	1	0	0
vith concret	1-5 people	3	0	0	1	0	0	0
vith concret	Above 15 p	1 2 3	1	1	1	0	0	0
flush toilet	6-10 peopl	1	1	0	0	0	0	0
vith concret	Above 15 p	1 2 3	1	1	1	0	0	0
vithout slab	6-10 peopl	1 3	1	0	1	0	0	0
flush toilet	6-10 peopl	1 3	1	0	1	0	0	0
vith concret	11-15 peop	4	0	0	0	1	0	0
vith concret	Do not sha	96	0	0	0	0	0	0
vith concret	11-15 peop	1 2 4 5	1	1	0	1	1	0
vith concret	11-15 peop	1 3 4 6	1	0	1	1	0	1
vith concret	Above 15 p	1 3 4	1	0	1	1	0	0
vith concret	Above 15 p	1 3 4 5	1	0	1	1	1	0
flush toilet	1-5 people	1	1	0	0	0	0	0
flush toilet	6-10 peopl	1	1	0	0	0	0	0
vith concret	Above 15 p	1 3	1	0	1	0	0	0
vith concret	Above 15 p	3	0	0	1	0	0	0
flush toilet	Above 15 p	1 3	1	0	1	0	0	0
flush toilet	11-15 peop	1 3	1	0	1	0	0	0
vith concret	6-10 peopl	1 2	1	1	0	0	0	0
flush toilet	1-5 people	1	1	0	0	0	0	0
flush toilet	11-15 peop	1 3	1	0	1	0	0	0
vith concret	11-15 peop	1 3 4 5 6	1	0	1	1	1	1
vith concret	1-5 people	1 2 4 6 7	1	1	0	1	0	1
vith concret	Above 15 p	2 3	0	1	1	0	0	0
flush toilet	Above 15 p	1 3 6	1	0	1	0	0	1
vith concret	6-10 peopl	1 3	1	0	1	0	0	0
flush toilet	Above 15 p	1 4	1	0	0	1	0	0
vith concret	Above 15 p	2 3 4	0	1	1	1	0	0
flush toilet	1-5 people	1 3	1	0	1	0	0	0
vith concret	Above 15 p	1 3 4 6 7	1	0	1	1	0	1
vith concret	1-5 people	1	1	0	0	0	0	0
vith concret	Above 15 p	1 3 4	1	0	1	1	0	0
vith concret	6-10 peopl	1 3	1	0	1	0	0	0
vith concret	Donâ€™t k	1 3	1	0	1	0	0	0
flush toilet	Above 15 p	1 3 4	1	0	1	1	0	0
vith concret	6-10 peopl	3	0	0	1	0	0	0
flush toilet	Above 15 p	1 3 4 5 7	1	0	1	1	1	0
flush toilet	Above 15 p	4	0	0	0	1	0	0

vith concret	Above 15 p	1 3 6	1	0	1	0	0	1
vith concret	Above 15 p	3	0	0	1	0	0	0
vith concret	1-5 people	1	1	0	0	0	0	0
vith concret	Donâ€™t k	1 3	1	0	1	0	0	0
vith concret	6-10 peopl	1 6 7	1	0	0	0	0	1
vith concret	Above 15 p	3	0	0	1	0	0	0
flush toilet	11-15 peop	4	0	0	0	1	0	0
vith concret	1-5 people	3	0	0	1	0	0	0
flush toilet	11-15 peop	1 4	1	0	0	1	0	0
flush toilet	6-10 peopl	3	0	0	1	0	0	0
flush toilet	1-5 people	1	1	0	0	0	0	0
flush toilet	Donâ€™t k	1 3 4 5 7	1	0	1	1	1	0
flush toilet	Above 15 p	1 3 4	1	0	1	1	0	0
flush toilet	11-15 peop	1 3	1	0	1	0	0	0
vith concret	Above 15 p	1 3 4 5	1	0	1	1	1	0
vithout slab	Above 15 p	1 3	1	0	1	0	0	0
vith concret	Above 15 p	1 3 4 6 7	1	0	1	1	0	1
flush toilet	Above 15 p	3 4 5	0	0	1	1	1	0
flush toilet	Above 15 p	1 3 4	1	0	1	1	0	0
flush toilet	Above 15 p	1 2 3 4	1	1	1	1	0	0
vith concret	1-5 people	1 4	1	0	0	1	0	0
flush toilet	1-5 people	1	1	0	0	0	0	0
flush toilet	6-10 peopl	4	0	0	0	1	0	0
vith concret	Above 15 p	1 2 3 4 6	1	1	1	1	0	1
flush toilet	11-15 peop	1 3	1	0	1	0	0	0
flush toilet	Above 15 p	1	1	0	0	0	0	0
flush toilet	Above 15 p	3 4 5 7	0	0	1	1	1	0
vith concret	Above 15 p	4	0	0	0	1	0	0
flush toilet	Do not sha	96	0	0	0	0	0	0
flush toilet	Above 15 p	3	0	0	1	0	0	0
flush toilet	11-15 peop	1 3	1	0	1	0	0	0
flush toilet	Above 15 p	1 3 4	1	0	1	1	0	0
vith concret	11-15 peop	1 3 4	1	0	1	1	0	0
flush toilet	Above 15 p	2 3 4 5 6	0	1	1	1	1	1
vith concret	Above 15 p	1 3 4	1	0	1	1	0	0
flush toilet	Above 15 p	1 3	1	0	1	0	0	0
flush toilet	Above 15 p	1 3 4	1	0	1	1	0	0
flush toilet	Above 15 p	1 3 4 5	1	0	1	1	1	0
vith concret	Above 15 p	3	0	0	1	0	0	0
vithout slab	11-15 peop	1 2	1	1	0	0	0	0
flush toilet	Above 15 p	3	0	0	1	0	0	0
flush toilet	Above 15 p	1 3 4	1	0	1	1	0	0
flush toilet	11-15 peop	1 3	1	0	1	0	0	0
vith concret	Above 15 p	1 3 4	1	0	1	1	0	0
flush toilet	6-10 peopl	1 3	1	0	1	0	0	0
vith concret	Above 15 p	1 3 5	1	0	1	0	1	0
flush toilet	11-15 peop	1 2	1	1	0	0	0	0
flush toilet	Above 15 p	1 3 4	1	0	1	1	0	0
flush toilet	11-15 peop	1 3	1	0	1	0	0	0

flush toilet	Do not sha	96	0	0	0	0	0	0
›nâ€™t kno	Above 15 ‚	1 4 5	1	0	0	1	1	0
flush toilet	11-15 peo‚	1 3	1	0	1	0	0	0
vith concret	Above 15 ‚	1 2 3 4	1	1	1	1	0	0
flush toilet	Above 15 ‚	1 3 4 5	1	0	1	1	1	0
vith concret	Above 15 ‚	3	0	0	1	0	0	0
vith concret	6-10 peopl	1 3	1	0	1	0	0	0
flush toilet	11-15 peo‚	1 3	1	0	1	0	0	0
vith concret	6-10 peopl	1 3	1	0	1	0	0	0
vithout slab	11-15 peo‚	1 3 6	1	0	1	0	0	1
vith concret	Donâ€™t k	1 3	1	0	1	0	0	0
vith concret	Above 15 ‚	1 2 3 4 5 6	1	1	1	1	1	1
flush toilet	11-15 peo‚	1 3 4	1	0	1	1	0	0
flush toilet	Above 15 ‚	1 3	1	0	1	0	0	0
flush toilet	Above 15 ‚	1 3	1	0	1	0	0	0
vith concret	11-15 peo‚	3	0	0	1	0	0	0
vith concret	Above 15 ‚	1 3 4 5	1	0	1	1	1	0
vith concret	Above 15 ‚	1 3 4	1	0	1	1	0	0
flush toilet	11-15 peo‚	1 3	1	0	1	0	0	0
flush toilet	6-10 peopl	1 2	1	1	0	0	0	0
flush toilet	Above 15 ‚	1 3	1	0	1	0	0	0
flush toilet	Do not sha	96	0	0	0	0	0	0
flush toilet	1-5 people	1	1	0	0	0	0	0
flush toilet	6-10 peopl	1	1	0	0	0	0	0
flush toilet	11-15 peo‚	1 3 4	1	0	1	1	0	0
flush toilet	Do not sha	96	0	0	0	0	0	0
flush toilet	Do not sha	1	1	0	0	0	0	0
flush toilet	11-15 peo‚	1 3 4 5	1	0	1	1	1	0
flush toilet	Above 15 ‚	1 3 4 5	1	0	1	1	1	0
flush toilet	1-5 people	1	1	0	0	0	0	0
vith concret	Donâ€™t k	1 3	1	0	1	0	0	0
flush toilet	Above 15 ‚	3	0	0	1	0	0	0
flush toilet	1-5 people	3	0	0	1	0	0	0
vith concret	Above 15 ‚	1 3 6	1	0	1	0	0	1
›ush/field	Donâ€™t k	3 4 7	0	0	1	1	0	0
›nâ€™t kno	6-10 peopl	1	1	0	0	0	0	0
vith concret	11-15 peo‚	1 3 4 5 6	1	0	1	1	1	1
flush toilet	11-15 peo‚	1 3	1	0	1	0	0	0
vith concret	6-10 peopl	3	0	0	1	0	0	0
vith concret	Above 15 ‚	1 2 3 4 5 6	1	1	1	1	1	1
vith concret	11-15 peo‚	1 3	1	0	1	0	0	0
flush toilet	6-10 peopl	1 4	1	0	0	1	0	0
vith concret	Above 15 ‚	1 3	1	0	1	0	0	0
vith concret	Above 15 ‚	1 3 4 5	1	0	1	1	1	0
flush toilet	1-5 people	1	1	0	0	0	0	0
flush toilet	1-5 people	1	1	0	0	0	0	0
flush toilet	Above 15 ‚	3 5	0	0	1	0	1	0
vith concret	1-5 people	1 2 3 4	1	1	1	1	0	0
vith concret	1-5 people	1	1	0	0	0	0	0

flush toilet	11-15 peop	3	0	0	1	0	0	0
flush toilet	Above 15 p	1 3 4 5	1	0	1	1	1	0
flush toilet	6-10 peopl	1 4	1	0	0	1	0	0
flush toilet	Above 15 p	2 3	0	1	1	0	0	0
vithout slab	1-5 people	1 2	1	1	0	0	0	0
flush toilet	6-10 peopl	1	1	0	0	0	0	0
vith concret	Above 15 p	1 3 5	1	0	1	0	1	0
vith concret	6-10 peopl	1 3	1	0	1	0	0	0
vith concret	Above 15 p	1 3 4 5 6 7	1	0	1	1	1	1
vith concret	Do not sha	1 2	1	1	0	0	0	0
flush toilet	11-15 peop	1 3	1	0	1	0	0	0
flush toilet	Above 15 p	1 3	1	0	1	0	0	0
onâ€™t kno	11-15 peop	1 2 3 4	1	1	1	1	0	0
flush toilet	Above 15 p	1 3	1	0	1	0	0	0
vith concret	Above 15 p	1 3	1	0	1	0	0	0
flush toilet	6-10 peopl	1 4	1	0	0	1	0	0
flush toilet	6-10 peopl	1 3	1	0	1	0	0	0
flush toilet	1-5 people	1	1	0	0	0	0	0
onâ€™t kno	Above 15 p	1 3 4 5 7	1	0	1	1	1	0
vith concret	1-5 people	3	0	0	1	0	0	0
vith concret	6-10 peopl	1	1	0	0	0	0	0
flush toilet	Above 15 p	3	0	0	1	0	0	0
vith concret	11-15 peop	1 4 6 7	1	0	0	1	0	1
oush/field	Donâ€™t k	7	0	0	0	0	0	0
flush toilet	6-10 peopl	1 3	1	0	1	0	0	0
flush toilet	11-15 peop	1 3 4	1	0	1	1	0	0
vith concret	11-15 peop	1 3 4 5	1	0	1	1	1	0
vith concret	Above 15 p	3	0	0	1	0	0	0
flush toilet	Above 15 p	1 3	1	0	1	0	0	0
vithout slab	11-15 peop	1 2 3 4 6	1	1	1	1	0	1
flush toilet	Donâ€™t k	1 3	1	0	1	0	0	0
flush toilet	1-5 people	1	1	0	0	0	0	0
flush toilet	Above 15 p	4	0	0	0	1	0	0
flush toilet	11-15 peop	4	0	0	0	1	0	0
vithout slab	1-5 people	1 2 3 4	1	1	1	1	0	0
flush toilet	11-15 peop	1 3	1	0	1	0	0	0
vith concret	Above 15 p	1 3	1	0	1	0	0	0
flush toilet	11-15 peop	1 3	1	0	1	0	0	0
vith concret	11-15 peop	1 2 3	1	1	1	0	0	0
flush toilet	11-15 peop	1 3 4	1	0	1	1	0	0
vith concret	11-15 peop	3 4 6	0	0	1	1	0	1
vith concret	Donâ€™t k	1 3	1	0	1	0	0	0
vith concret	Above 15 p	1 3 4 5 6	1	0	1	1	1	1
vith concret	6-10 peopl	1	1	0	0	0	0	0
flush toilet	Above 15 p	1 2 3	1	1	1	0	0	0
flush toilet	11-15 peop	1 3 4 5	1	0	1	1	1	0
vith concret	Above 15 p	3	0	0	1	0	0	0
vith concret	Above 15 p	3 4 5 6 7	0	0	1	1	1	1
flush toilet	6-10 peopl	1	1	0	0	0	0	0

vith concret	1-5 people	1 2 3 5	1	1	1	0	1	0
flush toilet	11-15 peo	1 3	1	0	1	0	0	0
vith concret	6-10 peopl	1 2 3 4	1	1	1	1	0	0
flush toilet	1-5 people	1	1	0	0	0	0	0
flush toilet	6-10 peopl	1 3 5 7	1	0	1	0	1	0
onâ€™t kno	Above 15 p	1 3 4 5	1	0	1	1	1	0
vith concret	Above 15 p	1 2 3 5 6	1	1	1	0	1	1
flush toilet	1-5 people	1 3	1	0	1	0	0	0
vith concret	Above 15 p	1 2 3 4	1	1	1	1	0	0
vith concret	Above 15 p	1 3 4	1	0	1	1	0	0
	1-5 people	1	1	0	0	0	0	0
flush toilet	6-10 peopl	1 3	1	0	1	0	0	0
vith concret	Above 15 p	3	0	0	1	0	0	0
vith concret	11-15 peo	1 2 3 4 6	1	1	1	1	0	1
vith concret	Above 15 p	2 3 4 6	0	1	1	1	0	1
flush toilet	6-10 peopl	1 3	1	0	1	0	0	0
flush toilet	Above 15 p	1 3	1	0	1	0	0	0
vith concret	Above 15 p	3	0	0	1	0	0	0
vith concret	Above 15 p	1 3 4	1	0	1	1	0	0
vith concret	11-15 peo	1 3	1	0	1	0	0	0
flush toilet	Above 15 p	1 3 4 6	1	0	1	1	0	1
flush toilet	Above 15 p	1 4	1	0	0	1	0	0
vith concret	Above 15 p	1 3 4	1	0	1	1	0	0
flush toilet	Above 15 p	1 3	1	0	1	0	0	0
flush toilet	11-15 peo	4	0	0	0	1	0	0
onâ€™t kno	Above 15 p	1 3	1	0	1	0	0	0
flush toilet	6-10 peopl	1 4	1	0	0	1	0	0
vith concret	Above 15 p	3	0	0	1	0	0	0
vith concret	Above 15 p	1 3 4	1	0	1	1	0	0
flush toilet	Donâ€™t k	1 3	1	0	1	0	0	0
vithout slab	1-5 people	1	1	0	0	0	0	0
flush toilet	Donâ€™t k	1 4	1	0	0	1	0	0
flush toilet	11-15 peo	1 3	1	0	1	0	0	0
vith concret	Above 15 p	1 2 4 6	1	1	0	1	0	1
vithout slab	Above 15 p	1 2 4	1	1	0	1	0	0
vith concret	Above 15 p	3	0	0	1	0	0	0
vith concret	11-15 peo	1 3	1	0	1	0	0	0
vith concret	11-15 peo	2 3 6	0	1	1	0	0	1
vith concret	11-15 peo	3	0	0	1	0	0	0
vith concret	Above 15 p	1 3 4	1	0	1	1	0	0
vith concret	Above 15 p	1 3	1	0	1	0	0	0
flush toilet	11-15 peo	1 4	1	0	0	1	0	0
flush toilet	Above 15 p	1 3	1	0	1	0	0	0
vith concret	Above 15 p	2 3 4 5 6	0	1	1	1	1	1
flush toilet	Above 15 p	3	0	0	1	0	0	0
flush toilet	11-15 peo	1 3	1	0	1	0	0	0
flush toilet	Above 15 p	3	0	0	1	0	0	0
flush toilet	Above 15 p	1 3	1	0	1	0	0	0
flush toilet	Above 15 p	1 3 4	1	0	1	1	0	0

flush toilet	1-5 people		1	1	0	0	0	0	0
flush toilet	6-10 peopl	1 2		1	1	0	0	0	0
vith concret	Above 15 p	1 3 4		1	0	1	1	0	0
flush toilet	Above 15 p	1 2		1	1	0	0	0	0
flush toilet	6-10 peopl		3	0	0	1	0	0	0
vith concret	Above 15 p	1 3 4		1	0	1	1	0	0
vith concret	6-10 peopl	1 2 3 4		1	1	1	1	0	0
flush toilet	6-10 peopl	1 3		1	0	1	0	0	0
flush toilet	6-10 peopl		1	1	0	0	0	0	0
flush toilet	Above 15 p		4	0	0	0	1	0	0
vith concret	11-15 peop		5	0	0	0	0	1	0
flush toilet	Above 15 p	2 4 6		0	1	0	1	0	1
vith concret	11-15 peop	1 2 3 4 5 6		1	1	1	1	1	1
flush toilet	Above 15 p	1 3 4		1	0	1	1	0	0
flush toilet	Above 15 p	1 3 4 5		1	0	1	1	1	0
flush toilet	Above 15 p	1 2 3		1	1	1	0	0	0
flush toilet	Do not sha		96	0	0	0	0	0	0
vith concret	1-5 people		3	0	0	1	0	0	0
vith concret	Donâ€™t k	1 3		1	0	1	0	0	0
flush toilet	Above 15 p	1 3		1	0	1	0	0	0
vithout slab	11-15 peop	1 3 4		1	0	1	1	0	0
flush toilet	Above 15 p	4 5		0	0	0	1	1	0
flush toilet	1-5 people		1	1	0	0	0	0	0
flush toilet	6-10 peopl		1	1	0	0	0	0	0
flush toilet	6-10 peopl	1 3		1	0	1	0	0	0
flush toilet	Above 15 p	1 3 4		1	0	1	1	0	0
flush toilet	11-15 peop	1 2 3 4		1	1	1	1	0	0
vith concret	11-15 peop	1 2 3		1	1	1	0	0	0
flush toilet	6-10 peopl	1 3		1	0	1	0	0	0
oush/field	Donâ€™t k		7	0	0	0	0	0	0
flush toilet	Above 15 p	3 5 7		0	0	1	0	1	0
flush toilet	Above 15 p		3	0	0	1	0	0	0
vith concret	Above 15 p	1 3		1	0	1	0	0	0
flush toilet	11-15 peop	1 3		1	0	1	0	0	0
vith concret	11-15 peop	1 3 5		1	0	1	0	1	0
vith concret	Above 15 p	2 3 5 6 7		0	1	1	0	1	1
flush toilet	1-5 people		1	1	0	0	0	0	0
flush toilet	Above 15 p	1 2 3 4		1	1	1	1	0	0
flush toilet	1-5 people		1	1	0	0	0	0	0
flush toilet	Above 15 p	1 4		1	0	0	1	0	0
vith concret	Above 15 p	1 3 4		1	0	1	1	0	0
vith concret	Above 15 p	1 3 6		1	0	1	0	0	1
flush toilet	Above 15 p	1 3		1	0	1	0	0	0
vith concret	Above 15 p	1 3 4		1	0	1	1	0	0
flush toilet	Above 15 p	1 3 4		1	0	1	1	0	0
flush toilet	Above 15 p	1 3 4		1	0	1	1	0	0
flush toilet	Above 15 p	1 3		1	0	1	0	0	0
flush toilet	11-15 peop	1 3 4 5		1	0	1	1	1	0
g/EcoSan t	Above 15 p	3 4		0	0	1	1	0	0

vith concret	Above 15 p		3	0	0	1	0	0	0
flush toilet	1-5 people		1	1	0	0	0	0	0
vithout slab	Above 15 p	1 3 4		1	0	1	1	0	0
vith concret	Donâ€™t k	1 3		1	0	1	0	0	0
flush toilet	Above 15 p	4 5		0	0	0	1	1	0
flush toilet	Above 15 p	1 2 3 4 5 6		1	1	1	1	1	1
vith concret	1-5 people		1	1	0	0	0	0	0
vithout slab	Donâ€™t k	1 5		1	0	0	0	1	0
flush toilet	Above 15 p	1 3 4 5		1	0	1	1	1	0
vith concret	Above 15 p	1 3 4 7		1	0	1	1	0	0
flush toilet	1-5 people		1	1	0	0	0	0	0
vith concret	6-10 peopl		3	0	0	1	0	0	0
flush toilet	11-15 peop	1 3		1	0	1	0	0	0
flush toilet	11-15 peop	1 3 4		1	0	1	1	0	0
vith concret	6-10 peopl		1	1	0	0	0	0	0
vith concret	11-15 peop		3	0	0	1	0	0	0
flush toilet	Above 15 p		4	0	0	0	1	0	0
flush toilet	6-10 peopl	1 3		1	0	1	0	0	0
flush toilet	Above 15 p	1 3		1	0	1	0	0	0
vith concret	Above 15 p	1 3 6		1	0	1	0	0	1
vith concret	Above 15 p	1 2 5		1	1	0	0	1	0
vith concret	Above 15 p	1 3 4 5		1	0	1	1	1	0
flush toilet	Above 15 p	1 3 4 5		1	0	1	1	1	0
flush toilet	1-5 people		1	1	0	0	0	0	0
flush toilet	Above 15 p	1 3		1	0	1	0	0	0

q65a_7	q65a_96	q65b	q66a	q66b	q67	q68	q68_1	q68_2
0	0		A bathing area in the (		No			
0	0		A bathing area in the (		No			
0	0		A bathing area in the (		No			
0	0		A bathing area in the (		No			
0	0		A bathing area in the (		No			
1	0		From a basin in a roor		Yes	3	0	0
0	0		A bathing area in the (		Yes	3	0	0
0	0		A bathing area in the (		No			
0	0		A bathing area in the (		No			
0	0		A bathing area in the (		No			
0	0		A bathing area in the (		No			
0	0		A bathing area in the (		Yes	3	0	0
0	0		A bathing area in the (		No			
0	0		A bathing area in the (		No			
0	0		A bathing area in the (		Yes	3	0	0
1	0		A bathing area in the (		Yes	3	0	0
0	0		A bathing area in the (		No			
0	0		Latrine		No			
0	0		A bathing area in the (		No			
0	0		From a basin in a roor		No			
0	0		A bathing area in the (		Yes	2 3	0	1
0	0		A bathing area in the (		No			
0	0		A bathing area in the (		No			
0	0		A bathing area in the (		No			
0	0		A bathing area in the (		No			
0	0		A bathing area in the (		No			
0	0		A bathing area in the (		No			
0	0		A bathing area in the (		No			
1	0		A bathing area in the (		No			
0	0		A bathing area in the (		No			
0	0		A bathing area in the (		No			
0	0		In a shower/tub/bath r		No			
0	0		A bathing area in the (		Yes	3	0	0
0	0		From a basin in a roor		No			
0	0		Latrine		No			
0	0		A bathing area in the (		No			
0	0		From a basin in a roor		No			
0	0		A bathing area in the (		Yes	3	0	0
0	0		A bathing area in the (		Yes	2 3	0	1
0	0		A bathing area in the (		No			
0	0		From a basin in a roor		No			
0	0		A bathing area in the (		No			
0	0		Latrine		Yes	3	0	0
0	0		From a basin in a roor		No			
0	0		A bathing area in the (		No			
0	0		Latrine		No			
0	0		Latrine		No			
0	0		A bathing area in the (		No			

0	0	A bathing area in the (		No			
0	0	A bathing area in the (		No			
0	0	A bathing area in the (		No			
0	0	In a shower/tub/bath r		No			
0	0	A bathing area in the (		No			
0	0	A bathing area in the (		Yes	3	0	0
0	0	Latrine		No			
0	0	A bathing area in the (		No			
0	0	Latrine		No			
0	0	A bathing area in the (		No			
0	0	A bathing area in the (		No			
0	0	Latrine		No			
1	0	A bathing area in the (		No			
0	0	In a shower/tub/bath r		No			
0	0	A bathing area in the (		No			
0	0	In a shower/tub/bath r		No			
0	0	Latrine		No			
0	0	In a shower/tub/bath r		No			
0	0	A bathing area in the (		No			
0	0	A bathing area in the (		No			
0	0	A bathing area in the (		Yes	1	1	0
0	0	Latrine		No			
1	0	Other (Spe	At workpla	No			
0	0	A bathing area in the (		No			
0	0	A bathing area in the (		No			
0	0	Latrine		No			
0	0	A bathing area in the (		No			
0	0	A bathing area in the (		No			
1	0	A bathing area in the (		No			
0	0	A bathing area in the (		No			
0	0	In a shower/tub/bath r		No			
0	0	A bathing area in the (		No			
0	0	From a basin in a roor		Yes	3	0	0
0	0	A bathing area in the (		Yes	1	1	0
0	0	A bathing area in the (		No			
0	0	A bathing area in the (		Yes	1	1	0
0	0	A bathing area in the (		No			
0	0	A bathing area in the (		No			
0	0	A bathing area in the (		Yes	1	1	0
0	0	A bathing area in the (		No			
0	0	A bathing area in the (		No			
0	0	A bathing area in the (		No			
0	0	A bathing area in the (		No			
0	0	A bathing area in the (		No			
0	0	A bathing area in the (		No			
0	0	Latrine		No			
0	0	A bathing area in the (		No			
0	0	In a shower/tub/bath r		No			
0	0	A bathing area in the (		No			

0	0	A bathing area in the c	Yes	2	0	1
0	0	In a shower/tub/bath r	No			
1	0	Public bathing facility	No			
0	0	A bathing area in the c	No			
0	0	A bathing area in the c	Yes	1	1	0
0	0	From a basin in a roor	No			
1	0	A bathing area in the c	No			
0	0	In a shower/tub/bath r	No			
0	0	A bathing area in the c	No			
0	0	A bathing area in the c	No			
0	0	A bathing area in the c	No			
0	0	A bathing area outside	No			
0	0	A bathing area in the c	No			
0	0	A bathing area in the c	No			
1	0	A bathing area in the c	No			
1	0	A bathing area in the c	No			
0	0	A bathing area in the c	No			
0	0	In a shower/tub/bath r	Yes	3	0	0
0	0	A bathing area in the c	No			
0	0	In a shower/tub/bath r	No			
0	0	Latrine	No			
0	0	A bathing area in the c	No			
0	0	A bathing area in the c	Yes	3	0	0
0	0	A bathing area in the c	No			
0	0	A bathing area in the c	No			
0	0	A bathing area in the c	No			
0	0	A bathing area in the c	No			
0	0	A bathing area in the c	No			
0	0	A bathing area in the c	No			
0	0	In a shower/tub/bath r	No			
0	0	From a basin in a roor	No			
1	0	A bathing area in the c	No			
0	0	A bathing area in the c	No			
1	0	A bathing area in the c	No			
0	0	A bathing area in the c	No			
0	0	A bathing area in the c	No			
1	0	A bathing area in the c	Yes	2	0	1
0	0	A bathing area in the c	No			
0	0	In a shower/tub/bath r	No			
0	0	In a shower/tub/bath r	No			
0	0	In a shower/tub/bath r	No			
0	0	In a shower/tub/bath r	No			
1	0	From a basin in a roor	No			
0	0	Latrine	No			
0	0	A bathing area in the c	Yes	1	1	0
0	0	From a basin in a roor	Yes	3	0	0
0	0	A bathing area in the c	No			
0	0	Latrine	No			
0	0	A bathing area in the c	No			

0	0		A bathing area in the (	Yes	3	0	0
0	0		A bathing area in the (	No			
0	0		A bathing area in the (	No			
0	0		A bathing area in the (	No			
0	0		Latrine	No			
0	0		Latrine	No			
0	0		A bathing area in the (	No			
1	0		A bathing area in the (	Yes	3	0	0
0	0		A bathing area in the (	No			
0	1	People in t	Latrine	No			
0	0		Latrine	No			
0	0		A bathing area in the (	Yes	3	0	0
0	0		A bathing area in the (	No			
0	0		A bathing area in the (	No			
0	0		In a shower/tub/bath r	No			
0	0		In a shower/tub/bath r	Yes	1	1	0
0	0		A bathing area in the (	No			
0	0		A bathing area in the (	No			
0	0		A bathing area in the (	No			
0	0		Latrine	No			
0	0		A bathing area in the (	No			
0	0		In a shower/tub/bath r	Yes	3	0	0
0	0		Latrine	No			
0	0		A bathing area in the (	No			
0	0		A bathing area in the (	No			
0	0		A bathing area in the (	No			
0	0		A bathing area in the (	No			
1	0		A bathing area in the (	Yes	2	0	1
0	0		A bathing area in the (	No			
0	0		A bathing area in the (	No			
0	0		A bathing area in the (	Yes	2	0	1
0	0		Latrine	No			
0	0		A bathing area in the (	No			
0	0		A bathing area in the (	No			
0	0		Latrine	No			
0	0		Latrine	Yes	3	0	0
0	0		In a shower/tub/bath r	No			
0	0		A bathing area in the (	No			
0	0		A bathing area in the (	No			
0	0		A bathing area in the (	No			
0	0		A bathing area in the (	No			
1	0		A bathing area in the (	Yes	1	1	0
0	0		A bathing area in the (	No			
1	0		A bathing area in the (	No			
0	0		A bathing area in the (	No			
0	0		In a shower/tub/bath r	No			
0	0		A bathing area in the (	No			
0	0		A bathing area in the (	No			
0	0		Public bathing facility	No			

0	0		A bathing area in the c		Yes	1	1	0
0	0		A bathing area in the c		No			
0	0		A bathing area in the c		Yes	1	1	0
0	0		A bathing area in the c		No			
0	1	Co workers	From a basin in a roor		No			
0	0		A bathing area in the c		No			
1	0		A bathing area in the c		No			
0	0		In a shower/tub/bath r		No			
0	0		In a shower/tub/bath r		No			
0	0		A bathing area in the c		No			
0	0		In a shower/tub/bath r		No			
0	0		A bathing area in the c		No			
0	0		A bathing area in the c		No			
0	0		A bathing area in the c		No			
0	0		A bathing area in the c		No			
0	0		A bathing area in the c		No			
0	0		A bathing area in the c		No			
1	0		A bathing area in the c		No			
0	0		A bathing area in the c		No			
0	0		In a shower/tub/bath r		Yes	2	0	1
0	0		Latrine		No			
1	0		From a basin in a roor		No			
0	0		Other (Spe	From a bas	No			
0	0		A bathing area in the c		No			
0	0		In a shower/tub/bath r		No			
0	0		A bathing area in the c		No			
0	0		Latrine		No			
1	0		Lake/ocean/pond/river		No			
1	0		A bathing area in the c		No			
0	0		A bathing area in the c		No			
0	0		A bathing area in the c		No			
0	0		Latrine		Yes	2	0	1
0	0		From a basin in a roor		No			
0	0		A bathing area in the c		No			
0	0		A bathing area in the c		No			
0	0		Latrine		No			
1	0		A bathing area in the c		No			
0	0		A bathing area in the c		Yes	3	0	0
0	0		A bathing area in the c		No			
0	0		From a basin in a roor		No			
0	0		A bathing area in the c		No			
0	0		A bathing area in the c		No			
0	0		In a shower/tub/bath r		No			
0	0		A bathing area in the c		No			
0	0		A bathing area in the c		No			
0	0		A bathing area in the c		No			
0	0		From a basin in a roor		No			
0	0		A bathing area in the c		No			
0	0		A bathing area in the c		Yes	3	0	0

0	0	A bathing area outside	No			
0	0	In a shower/tub/bath r	No			
0	0	A bathing area in the c	No			
0	0	A bathing area in the c	No			
0	0	A bathing area in the c	No			
1	0	A bathing area in the c	Yes	1	1	0
0	0	A bathing area in the c	No			
0	0	A bathing area outside	Yes	3	0	0
0	0	Latrine	No			
0	0	A bathing area in the c	No			
0	0	Public bathing facility	No			
0	0	A bathing area in the c	No			
0	0	A bathing area in the c	No			
0	0	Latrine	No			
0	0	A bathing area in the c	No			
0	0	A bathing area in the c	No			
0	0	A bathing area in the c	No			
0	0	A bathing area in the c	No			
0	0	In a shower/tub/bath r	No			
0	0	A bathing area in the c	No			
1	0	A bathing area in the c	No			
0	0	A bathing area in the c	No			
0	0	In a shower/tub/bath r	No			
0	0	In a shower/tub/bath r	No			
1	0	A bathing area in the c	Yes	2	0	1
0	0	A bathing area in the c	Yes	3	0	0
0	0	A bathing area in the c	No			
0	0	In a shower/tub/bath r	No			
0	0	A bathing area in the c	No			
0	0	A bathing area in the c	No			
0	0	A bathing area in the c	No			
0	0	A bathing area in the c	No			
0	0	A bathing area in the c	No			
0	0	In a shower/tub/bath r	No			
0	0	A bathing area in the c	No			
0	0	A bathing area in the c	Yes	3	0	0
0	0	A bathing area in the c	Yes	3	0	0
0	0	Latrine	Yes	3	0	0
0	0	A bathing area in the c	No			
0	0	In a shower/tub/bath r	No			
1	0	A bathing area in the c	No			
0	0	A bathing area in the c	No			
0	0	A bathing area in the c	No			
0	0	A bathing area outside	No			
0	0	A bathing area in the c	No			
0	0	A bathing area in the c	No			
0	0	Latrine	No			
0	0	A bathing area in the c	No			
0	0	A bathing area in the c	No			

0	0		A bathing area in the (	No			
0	0		A bathing area in the (	Yes	1	1	0
0	0		A bathing area in the (	No			
0	0		Latrine	No			
0	0		From a basin in a roor	No			
0	0		A bathing area in the (	No			
0	0		A bathing area in the (	No			
0	0		A bathing area in the (	No			
0	0		A bathing area in the (	Yes	3	0	0
0	0		A bathing area in the (	Yes	1	1	0
0	0		From a basin in a roor	No			
0	0		A bathing area in the (	No			
0	0		A bathing area in the (	Yes	1	1	0
0	0		A bathing area in the (	No			
0	0		A bathing area in the (	No			
0	0		A bathing area in the (	No			
0	0		Latrine	No			
0	0		A bathing area in the (	No			
0	0		A bathing area in the (	No			
0	0		A bathing area in the (	Yes	3	0	0
0	0		A bathing area in the (	No			
0	0		A bathing area in the (	No			
0	0		From a basin in a roor	No			
0	0		A bathing area in the (	No			
0	0		A bathing area in the (	Yes	1	1	0
1	0		A bathing area in the (	No			
0	0		A bathing area in the (	No			
0	0		A bathing area in the (	No			
0	0		From a basin in a roor	No			
1	1	We share \	Latrine	No			
0	0		In a shower/tub/bath r	No			
0	0		A bathing area in the (	No			
0	0		A bathing area in the (	No			
0	0		A bathing area in the (	No			
0	0		Latrine	No			
0	0		Latrine	No			
0	0		A bathing area in the (	No			
0	0		A bathing area in the (	No			
0	0		A bathing area in the (	No			
0	0		A bathing area in the (	No			
0	0		In a shower/tub/bath r	No			
0	0		A bathing area in the (	No			
0	0		Latrine	No			
0	0		A bathing area in the (	No			
0	0		A bathing area in the (	No			
0	0		A bathing area in the (	No			
0	0		Latrine	No			
0	0		A bathing area in the (	No			
0	0		A bathing area in the (	No			

0	0	A bathing area in the c	No				
0	0	A bathing area in the c	No				
0	0	A bathing area in the c	No				
0	0	Latrine	No				
0	0	In a shower/tub/bath r	No				
0	0	A bathing area in the c	No				
0	0	A bathing area in the c	Yes		2	0	1
1	0	A bathing area in the c	Yes		3	0	0
0	0	In a shower/tub/bath r	Yes		2	0	1
0	0	Latrine	No				
0	0	A bathing area in the c	No				
0	0	Latrine	No				
0	0	A bathing area in the c	No				
0	0	A bathing area in the c	No				
0	0	A bathing area in the c	Yes	1 3		1	0
0	0	Public bathing facility	No				
0	0	Latrine	No				
0	0	A bathing area in the c	No				
0	0	A bathing area in the c	No				
0	0	A bathing area in the c	No				
0	0	A bathing area in the c	Yes		3	0	0
0	0	Latrine	No				
0	0	A bathing area in the c	No				
0	0	Latrine	No				
0	0	A bathing area in the c	No				
0	0	A bathing area in the c	No				
0	0	From a basin in a roor	No				
0	0	A bathing area in the c	No				
1	0	A bathing area in the c	No				
0	0	A bathing area in the c	Yes		3	0	0
0	0	A bathing area in the c	No				
0	0	A bathing area in the c	No				
0	0	A bathing area in the c	No				
0	0	In a shower/tub/bath r	Yes		2	0	1
1	0	A bathing area in the c	No				
0	0	A bathing area in the c	Yes		3	0	0
0	0	A bathing area in the c	No				
0	0	A bathing area in the c	No				
0	0	A bathing area in the c	No				
0	0	A bathing area in the c	No				
0	0	From a basin in a roor	Yes		3	0	0
0	0	A bathing area in the c	No				
1	0	A bathing area in the c	Yes		1	1	0
0	0	Latrine	No				
0	0	Latrine	No				
0	0	A bathing area in the c	No				
0	0	A bathing area in the c	Yes		3	0	0
0	0	A bathing area in the c	No				
0	0	From a basin in a roor	No				

0	0	From a basin in a roor	No			
0	0	A bathing area in the (	Yes	1	1	0
0	0	A bathing area in the (	No			
0	0	A bathing area in the (	No			
0	0	A bathing area in the (	No			
0	0	A bathing area in the (	No			
0	0	In a shower/tub/bath r	No			
0	0	In a shower/tub/bath r	No			
0	0	A bathing area in the (	No			
1	0	A bathing area in the (	Yes	1	1	0
0	0	A bathing area in the (	No			
0	0	A bathing area in the (	Yes	3	0	0
0	0	Latrine	No			
0	0	Latrine	No			
0	0	A bathing area in the (	No			
0	0	A bathing area in the (	Yes	3	0	0
0	0	A bathing area in the (	No			
0	0	A bathing area in the (	Yes	1	1	0
0	0	A bathing area in the (	No			
0	0	A bathing area in the (	Yes	3	0	0
0	0	A bathing area in the (	No			
0	0	A bathing area in the (	No			
0	0	A bathing area in the (	No			
0	0	A bathing area in the (	No			
0	0	A bathing area in the (	No			
0	0	A bathing area in the (	No			
0	0	A bathing area in the (	Yes	1	1	0
0	0	A bathing area in the (	No			
0	0	A bathing area in the (	No			
0	0	A bathing area in the (	No			
0	0	In a shower/tub/bath r	No			
0	0	A bathing area in the (	No			
0	0	A bathing area in the (	No			
0	0	A bathing area in the (	No			
0	0	A bathing area in the (	No			
0	0	A bathing area in the (	Yes	2	0	1
0	0	A bathing area in the (	No			
0	0	A bathing area in the (	No			
0	0	A bathing area in the (	No			
0	0	Latrine	No			
1	0	From a basin in a roor	No			
0	0	Latrine	No			
1	0	A bathing area outside	Yes	3	0	0
0	0	A bathing area in the (	No			
0	0	A bathing area in the (	No			
0	0	A bathing area in the (	No			
0	0	A bathing area in the (	No			
0	0	Latrine	No			
0	0	A bathing area in the (	No			

0	0	A bathing area in the (	No				
0	0	A bathing area in the (	No				
0	0	Latrine	No				
0	0	A bathing area outside	No				
1	0	A bathing area in the (	No				
0	0	A bathing area in the (	No				
0	0	A bathing area in the (	Yes		3	0	0
0	0	A bathing area in the (	No				
0	0	A bathing area in the (	No				
0	0	A bathing area in the (	Yes		3	0	0
0	0	A bathing area in the (	No				
0	0	A bathing area in the (	No				
1	0	A bathing area in the (	No				
0	0	A bathing area in the (	Yes	1 3		1	0
0	0	A bathing area in the (	No				
0	0	A bathing area in the (	Yes		1	1	0
0	0	Latrine	No				
0	0	From a basin in a roor	No				
0	0	Latrine	No				
0	0	A bathing area in the (	Yes		3	0	0
1	0	A bathing area in the (	No				
0	0	A bathing area in the (	Yes		2	0	1
0	0	Latrine	No				
0	0	A bathing area in the (	No				
0	0	A bathing area in the (	No				
0	0	A bathing area in the (	No				
0	0	In a shower/tub/bath r	No				
0	0	A bathing area in the (	No				
0	0	A bathing area in the (	No				
0	0	A bathing area in the (	Yes		3	0	0
0	0	A bathing area in the (	Yes		1	1	0
0	0	A bathing area in the (	Yes		1	1	0
0	0	A bathing area in the (	No				
0	0	A bathing area in the (	No				
1	0	A bathing area in the (	Yes		3	0	0
0	0	A bathing area in the (	No				
0	0	A bathing area in the (	No				
0	0	A bathing area in the (	No				
0	0	Latrine	No				
0	0	A bathing area in the (	No				
0	0	Latrine	No				
0	0	A bathing area in the (	No				
0	0	Latrine	No				
0	0	A bathing area in the (	Yes		1	1	0
0	0	A bathing area in the (	No				
0	0	A bathing area in the (	No				
1	0	A bathing area in the (	No				
0	0	In a shower/tub/bath r	No				
0	0	A bathing area in the (	No				

0	0	A bathing area in the	No				
0	0	A bathing area in the	No				
0	0	A bathing area in the	Yes		1	1	0
0	0	A bathing area in the	No				
0	0	From a basin in a roo	No				
0	0	From a basin in a roo	No				
0	0	Latrine	No				
0	0	A bathing area in the	No				
0	0	Latrine	No				
1	0	A bathing area in the	Yes	2 3		0	1
0	0	A bathing area in the	Yes		2	0	1
0	0	A bathing area in the	No				
0	0	A bathing area in the	Yes		2	0	1
0	0	A bathing area in the	No				
0	0	Latrine	Yes		2	0	1
0	0	A bathing area in the	No				
0	0	In a shower/tub/bath r	No				
0	0	In a shower/tub/bath r	No				
0	0	A bathing area in the	Yes		2	0	1
0	0	A bathing area in the	No				
0	0	A bathing area in the	Yes	1 2 3		1	1
0	0	A bathing area in the	No				
0	0	A bathing area in the	No				
0	0	A bathing area in the	No				
0	0	A bathing area in the	No				
0	0	A bathing area in the	No				
0	0	A bathing area in the	No				
0	0	A bathing area in the	No				
0	0	A bathing area in the	No				
0	0	Latrine	No				
0	0	From a basin in a roo	No				
0	0	A bathing area in the	Yes		1	1	0
0	0	A bathing area outside	No				
0	0	A bathing area in the	No				
0	0	A bathing area in the	No				
0	0	A bathing area in the	No				
0	0	A bathing area in the	No				
0	0	A bathing area in the	No				
0	0	A bathing area in the	No				
0	0	A bathing area in the	No				
0	0	Latrine	No				
1	0	A bathing area in the	No				
0	0	A bathing area in the	No				
0	0	A bathing area in the	No				
0	0	A bathing area in the	Yes		3	0	0
0	0	In a shower/tub/bath r	No				
0	0	Latrine	No				
0	0	A bathing area in the	No				
1	0	A bathing area in the	No				

0	0	Public bathing facility	No				
0	0	A bathing area in the (	No				
0	0	A bathing area in the (	No				
0	0	A bathing area in the (	No				
0	0	A bathing area in the (	No				
0	0	A bathing area in the (	No				
0	0	A bathing area in the (	Yes		3	0	0
0	0	A bathing area in the (	No				
0	0	Latrine	No				
0	0	A bathing area in the (	No				
0	0	In a shower/tub/bath r	No				
0	0	A bathing area in the (	No				
0	0	A bathing area in the (	No				
0	0	A bathing area in the (	No				
0	0	Latrine	No				
0	0	A bathing area in the (	No				
0	0	A bathing area in the (	No				
0	0	A bathing area in the (	No				
0	0	Public bathing facility	No				
0	0	A bathing area in the (	Yes		1	1	0
0	0	A bathing area in the (	No				
1	0	A bathing area in the (	No				
0	0	A bathing area in the (	No				
0	0	A bathing area in the (	Yes		3	0	0
0	0	A bathing area in the (	Yes		1	1	0
0	0	A bathing area in the (	Yes		1	1	0
0	0	From a basin in a roor	No				
0	0	A bathing area in the (	No				
0	0	A bathing area in the (	No				
0	0	In a shower/tub/bath r	No				
1	0	A bathing area in the (	No				
0	0	Latrine	No				
0	0	A bathing area in the (	No				
0	0	A bathing area in the (	Yes	1 2 3		1	1
0	0	A bathing area in the (	No				
0	0	A bathing area in the (	No				
0	0	A bathing area in the (	No				
0	0	A bathing area in the (	No				
0	0	A bathing area in the (	No				
0	0	A bathing area in the (	Yes		2	0	1
0	0	A bathing area in the (	Yes		3	0	0
0	0	Latrine	No				
0	0	A bathing area in the (	No				
0	0	A bathing area in the (	No				
0	0	A bathing area in the (	No				
0	0	A bathing area in the (	No				
0	0	A bathing area in the (	Yes		3	0	0
0	0	A bathing area in the (	No				
0	0	A bathing area in the (	No				

0	0	A bathing area in the	No				
0	0	A bathing area in the	No				
0	0	Latrine	No				
0	0	A bathing area in the	Yes		3	0	0
0	0	A bathing area in the	No				
0	0	A bathing area in the	No				
0	0	A bathing area in the	No				
0	0	A bathing area in the	No				
0	0	A bathing area in the	No				
0	0	A bathing area in the	No				
0	0	A bathing area in the	Yes	1 2		1	1
0	0	A bathing area in the	No				
0	0	In a shower/tub/bath r	No				
0	0	A bathing area in the	No				
0	0	A bathing area in the	No				
0	0	A bathing area in the	Yes		1	1	0
0	0	In a shower/tub/bath r	Yes		2	0	1
0	0	A bathing area in the	No				
0	0	Latrine	No				
0	0	A bathing area in the	No				
0	0	A bathing area in the	Yes		2	0	1
0	0	Latrine	No				
1	0	In a shower/tub/bath r	No				
0	0	A bathing area in the	Yes		1	1	0
0	0	A bathing area in the	No				
0	0	A bathing area in the	No				
0	0	From a basin in a roor	No				
0	0	A bathing area in the	Yes		3	0	0
0	0	From a basin in a roor	No				
0	0	A bathing area in the	No				
1	0	A bathing area in the	Yes		1	1	0
0	0	Latrine	No				
1	0	A bathing area in the	Yes	2 3		0	1
0	0	In a shower/tub/bath r	No				
0	0	A bathing area in the	No				
0	0	Latrine	No				
0	0	A bathing area in the	No				
0	0	A bathing area in the	No				
0	0	A bathing area in the	No				
0	0	Public bathing facility	Yes		1	1	0
0	0	Latrine	No				
0	0	Latrine	No				
0	0	A bathing area in the	No				
0	0	A bathing area in the	No				
0	0	A bathing area in the	No				
0	0	A bathing area in the	No				
0	0	A bathing area in the	No				
0	0	A bathing area in the	No				
0	0	A bathing area in the	No				

0	0	A bathing area in the	No				
0	0	In a shower/tub/bath r	No				
1	0	A bathing area in the	Yes		2	0	1
0	0	Latrine	Yes		1	1	0
0	0	In a shower/tub/bath r	No				
0	0	From a basin in a roor	No				
0	0	In a shower/tub/bath r	No				
0	0	A bathing area in the	No				
0	0	A bathing area in the	No				
0	0	A bathing area in the	No				
0	0	Latrine	No				
0	0	A bathing area in the	No				
0	0	A bathing area in the	No				
1	0	From a basin in a roor	No				
0	0	A bathing area in the	Yes		1	1	0
0	0	In a shower/tub/bath r	Yes		2	0	1
0	0	A bathing area in the	No				
0	0	A bathing area in the	Yes	1 2 3		1	1
0	0	A bathing area in the	No				
0	0	In a shower/tub/bath r	No				
0	0	Latrine	No				
0	0	A bathing area in the	No				
0	0	A bathing area in the	No				
0	0	In a shower/tub/bath r	No				
0	0	A bathing area in the	No				
0	0	Latrine	No				
0	0	A bathing area in the	No				
0	0	A bathing area in the	No				
0	0	A bathing area in the	No				
0	0	A bathing area in the	No				
0	0	A bathing area in the	Yes	1 3		1	0
0	0	Latrine	No				
0	0	From a basin in a roor	No				
0	0	A bathing area outside	No				
0	0	Public bathing facility	No				
0	0	Latrine	No				
0	0	A bathing area in the	Yes		3	0	0
0	0	A bathing area in the	No				
0	0	A bathing area in the	No				
0	0	A bathing area in the	Yes		3	0	0
0	0	A bathing area in the	No				
0	0	Public bathing facility	No				
0	0	A bathing area in the	No				
0	0	Latrine	No				
0	0	A bathing area in the	Yes		3	0	0
0	0	A bathing area in the	Yes		2	0	1
0	0	Latrine	No				
0	0	In a shower/tub/bath r	No				
0	0	In a shower/tub/bath r	Yes		3	0	0

1	0	A bathing area in the (	Yes	1 3		1	0
0	0	A bathing area in the (	No				
0	0	A bathing area in the (	No				
0	0	A bathing area in the (	Yes		1	1	0
0	0	A bathing area in the (	Yes	1 2 3		1	1
0	0	A bathing area in the (	No				
0	0	A bathing area in the (	No				
0	0	Latrine	No				
0	0	Public bathing facility	Yes		3	0	0
0	0	In a shower/tub/bath r	No				
0	0	A bathing area in the (	No				
1	0	A bathing area in the (	Yes		2	0	1
0	0	Latrine	No				
0	0	In a shower/tub/bath r	Yes		1	1	0
1	0	A bathing area in the (	Yes		1	1	0
0	0	A bathing area in the (	No				
0	0	A bathing area in the (	No				
0	0	A bathing area in the (	No				
0	0	A bathing area in the (	No				
0	0	A bathing area in the (	No				
1	0	Public bathing facility	No				
0	0	A bathing area in the (	Yes		2	0	1
0	0	Latrine	No				
0	0	In a shower/tub/bath r	No				
0	0	A bathing area in the (	No				
0	0	A bathing area in the (	No				
0	0	In a shower/tub/bath r	No				
0	0	A bathing area in the (	No				
0	0	A bathing area in the (	No				
0	0	A bathing area in the (	No				
0	0	Latrine	No				
0	0	Latrine	No				
0	0	Latrine	No				
1	0	A bathing area in the (	Yes		3	0	0
0	0	A bathing area in the (	No				
1	0	A bathing area in the (	No				
0	0	A bathing area in the (	No				
0	0	A bathing area in the (	No				
0	0	In a shower/tub/bath r	Yes		3	0	0
0	0	A bathing area in the (	No				
0	0	Latrine	Yes		2	0	1
0	0	Latrine	No				
0	0	A bathing area in the (	Yes		2	0	1
0	0	A bathing area in the (	No				
0	0	A bathing area in the (	No				
0	0	Latrine	No				
0	0	Public bathing facility	No				
1	0	A bathing area in the (	Yes		1	1	0
0	0	A bathing area in the (	No				

0	0	A bathing area in the	Yes	1 3		1	0
0	0	Latrine	No				
0	0	A bathing area in the	No				
0	0	A bathing area in the	Yes	1 2 3		1	1
0	0	Latrine	Yes		3	0	0
0	0	A bathing area in the	Yes		2	0	1
0	0	A bathing area in the	No				
0	0	A bathing area in the	Yes		3	0	0
1	0	A bathing area in the	No				
0	0	A bathing area in the	No				
0	0	Latrine	No				
0	0	From a basin in a roor	Yes	2 3		0	1
0	0	Public bathing facility	No				
0	0	A bathing area in the	No				
1	0	A bathing area in the	Yes	1 3		1	0
0	0	A bathing area in the	Yes		2	0	1
0	0	Latrine	No				
0	0	A bathing area in the	No				
0	0	A bathing area in the	Yes		3	0	0
0	0	A bathing area in the	No				
0	0	A bathing area in the	No				
0	0	A bathing area in the	No				
0	0	A bathing area in the	No				
0	0	A bathing area in the	No				
0	0	A bathing area in the	No				
0	0	A bathing area in the	No				
0	0	A bathing area in the	No				
0	0	Latrine	No				
0	0	A bathing area in the	No				
0	0	A bathing area in the	No				
0	0	Latrine	No				
1	0	A bathing area in the	No				
0	0	A bathing area in the	No				
0	0	A bathing area in the	No				
0	0	Latrine	No				
0	0	In a shower/tub/bath r	No				
0	0	A bathing area in the	No				
0	0	A bathing area in the	No				
0	0	A bathing area in the	No				
0	0	A bathing area in the	No				
0	0	A bathing area in the	No				
0	0	A bathing area in the	Yes		1	1	0
0	0	A bathing area in the	Yes		3	0	0
0	0	A bathing area in the	No				
0	0	Public bathing facility	No				
0	0	In a shower/tub/bath r	No				
0	0	A bathing area in the	No				
0	0	A bathing area in the	No				
0	0	A bathing area in the	No				

0	0	A bathing area in the (	No				
0	0	Latrine	No				
0	0	From a basin in a roor	No				
0	0	A bathing area in the (	Yes		1	1	0
0	0	A bathing area in the (	No				
0	0	Latrine	No				
0	0	Latrine	No				
0	0	A bathing area in the (	No				
0	0	Latrine	No				
0	0	A bathing area in the (	No				
0	0	Latrine	No				
0	0	A bathing area in the (	No				
0	0	Latrine	No				
0	0	A bathing area in the (	No				
0	0	In a shower/tub/bath r	No				
0	0	A bathing area in the (	No				
0	0	A bathing area in the (	No				
0	0	Latrine	No				
0	0	A bathing area in the (	No				
0	0	A bathing area in the (	Yes		1	1	0
0	0	A bathing area in the (	Yes		3	0	0
0	0	A bathing area in the (	No				
0	0	Latrine	No				
0	0	A bathing area in the (	No				
0	0	A bathing area in the (	No				
0	0	A bathing area in the (	No				
0	0	A bathing area in the (	Yes		3	0	0
0	0	A bathing area in the (	No				
0	0	A bathing area in the (	No				
0	0	A bathing area in the (	No				
0	0	A bathing area in the (	No				
0	0	A bathing area in the (	No				
0	0	A bathing area in the (	No				
0	0	A bathing area in the (	No				
0	0	A bathing area in the (	Yes		2	0	1
1	0	A bathing area in the (	No				
0	0	In a shower/tub/bath r	No				
0	0	A bathing area in the (	No				
0	0	Public bathing facility	Yes		3	0	0
0	0	A bathing area in the (	No				
0	0	In a shower/tub/bath r	No				
1	0	A bathing area in the (	No				
0	0	A bathing area in the (	No				
0	0	Latrine	No				
0	0	A bathing area in the (	No				
0	0	A bathing area in the (	Yes		1	1	0
0	0	Latrine	No				
0	0	A bathing area in the (	No				
0	0	A bathing area in the (	Yes	1 3		1	0

0	0	A bathing area in the c	No			
0	0	A bathing area in the c	No			
0	0	A bathing area in the c	No			
0	0	A bathing area in the c	No			
0	0	A bathing area in the c	No			
0	0	A bathing area in the c	Yes	3	0	0
0	0	A bathing area in the c	No			
0	0	A bathing area in the c	Yes	3	0	0
0	0	A bathing area in the c	No			
1	0	A bathing area in the c	No			
0	0	A bathing area in the c	Yes	2	0	1
0	0	From a basin in a roor	No			
0	0	Latrine	No			
0	0	A bathing area in the c	No			
0	0	A bathing area in the c	No			
0	0	A bathing area in the c	No			
0	0	A bathing area in the c	No			
0	0	A bathing area in the c	Yes	3	0	0
0	0	A bathing area in the c	No			
0	0	In a shower/tub/bath r	No			
1	0	From a basin in a roor	No			
0	0	A bathing area in the c	No			
0	0	A bathing area in the c	No			
0	0	A bathing area in the c	No			
0	0	A bathing area in the c	No			
0	0	A bathing area in the c	No			
0	0	A bathing area in the c	No			
0	0	A bathing area in the c	No			
0	0	A bathing area in the c	No			
0	0	In a shower/tub/bath r	No			
0	0	A bathing area in the c	No			
0	0	A bathing area in the c	No			
0	0	A bathing area in the c	Yes	3	0	0
0	0	A bathing area in the c	No			
0	0	A bathing area in the c	No			
0	0	A bathing area in the c	No			
0	0	A bathing area in the c	Yes	1	1	0
0	0	A bathing area in the c	No			
0	0	A bathing area in the c	No			
0	0	A bathing area in the c	No			
0	0	A bathing area in the c	No			
0	0	A bathing area in the c	Yes	3	0	0
0	0	Latrine	No			
0	0	Latrine	No			
0	0	A bathing area in the c	No			
0	0	From a basin in a roor	No			
0	0	A bathing area in the c	No			
1	0	A bathing area outside	No			
0	0	A bathing area in the c	Yes	3	0	0

0	0	A bathing area in the c	No			
0	0	A bathing area in the c	No			
0	0	Public bathing facility	No			
0	0	A bathing area in the c	No			
0	0	A bathing area in the c	No			
0	0	From a basin in a roor	Yes	2	0	1
0	0	A bathing area in the c	No			
0	0	Latrine	No			
0	0	A bathing area in the c	Yes	3	0	0
0	0	In a shower/tub/bath r	No			
0	0	A bathing area in the c	No			
0	0	Latrine	No			
0	0	A bathing area in the c	No			
0	0	A bathing area in the c	No			
0	0	A bathing area in the c	Yes	3	0	0
0	0	A bathing area in the c	Yes	3	0	0
0	0	Latrine	No			
0	0	A bathing area in the c	No			
0	0	A bathing area in the c	No			
0	0	Latrine	No			
0	0	A bathing area in the c	No			
0	0	A bathing area in the c	No			
0	0	Latrine	Yes	3	0	0
0	0	Latrine	No			
0	0	Latrine	No			
0	0	A bathing area in the c	No			
0	0	A bathing area in the c	No			
0	0	A bathing area in the c	Yes	3	0	0
0	0	A bathing area in the c	No			
0	0	A bathing area in the c	No			
0	0	A bathing area in the c	Yes	2	0	1
0	0	A bathing area in the c	Yes	2	0	1
0	0	A bathing area in the c	No			
0	0	In a shower/tub/bath r	No			
0	0	A bathing area in the c	No			
0	0	A bathing area in the c	No			
0	0	A bathing area in the c	No			
0	0	A bathing area in the c	No			
0	0	A bathing area in the c	No			
0	0	In a shower/tub/bath r	No			
0	0	A bathing area in the c	Yes	1	1	0
0	0	A bathing area in the c	No			
0	0	A bathing area in the c	Yes	3	0	0
0	0	A bathing area in the c	No			
0	0	In a shower/tub/bath r	No			
0	0	A bathing area in the c	No			
0	0	In a shower/tub/bath r	No			
0	0	In a shower/tub/bath r	No			
0	0	A bathing area in the c	No			

0	0	A bathing area in the (	No				
0	0	A bathing area in the (	No				
0	0	A bathing area in the (	Yes		2	0	1
0	0	A bathing area in the (	No				
0	0	A bathing area in the (	No				
0	0	Latrine	No				
0	0	A bathing area in the (	No				
0	0	Latrine	No				
0	0	Latrine	Yes		2	0	1
0	0	A bathing area in the (	No				
0	0	A bathing area in the (	No				
0	0	A bathing area in the (	No				
0	0	Latrine	No				
0	0	A bathing area in the (	No				
0	0	A bathing area in the (	No				
0	0	A bathing area in the (	No				
0	0	Latrine	No				
1	0	From a basin in a roor	No				
0	0	A bathing area in the (	No				
0	0	A bathing area in the (	No				
0	0	In a shower/tub/bath r	No				
0	0	A bathing area in the (	No				
0	0	Latrine	No				
0	0	A bathing area in the (	No				
0	0	A bathing area in the (	No				
0	0	A bathing area in the (	No				
0	0	A bathing area in the (	No				
0	0	A bathing area in the (	No				
0	0	A bathing area in the (	No				
0	0	Latrine	No				
0	0	Latrine	No				
0	0	In a shower/tub/bath r	No				
0	0	A bathing area in the (	No				
0	0	In a shower/tub/bath r	No				
0	0	Latrine	No				
0	0	A bathing area in the (	Yes		2	0	1
0	0	A bathing area in the (	No				
0	0	A bathing area in the (	No				
0	0	A bathing area in the (	No				
0	0	Latrine	No				
0	0	In a shower/tub/bath r	No				
0	0	A bathing area in the (	Yes		1	1	0
0	0	Latrine	No				
0	0	A bathing area in the (	No				
0	0	A bathing area in the (	No				
0	0	A bathing area in the (	Yes	2 3		0	1
0	0	In a shower/tub/bath r	No				
0	0	A bathing area in the (	Yes		3	0	0
0	0	Latrine	No				

0	0	A bathing area outside	No				
0	0	In a shower/tub/bath r	No				
0	0	A bathing area in the c	No				
0	0	Latrine	No				
0	0	Latrine	Yes		3	0	0
0	0	A bathing area in the c	Yes		1	1	0
1	0	A bathing area in the c	No				
0	0	Latrine	No				
0	0	A bathing area in the c	No				
0	0	A bathing area in the c	Yes		3	0	0
0	0	A bathing area in the c	No				
0	0	A bathing area in the c	No				
0	0	A bathing area in the c	No				
0	0	Latrine	No				
0	0	A bathing area in the c	No				
0	0	A bathing area in the c	Yes		3	0	0
0	0	A bathing area in the c	No				
0	0	A bathing area in the c	No				
0	0	A bathing area in the c	No				
0	0	Public bathing facility	Yes	1 2 3		1	1
0	0	A bathing area in the c	No				
0	0	A bathing area in the c	No				
0	0	Latrine	No				
0	0	A bathing area in the c	No				
1	0	A bathing area outside	No				
1	0	Latrine	No				
0	0	From a basin in a roor	No				
1	0	A bathing area in the c	No				
0	0	A bathing area in the c	Yes		2	0	1
1	0	A bathing area in the c	Yes	1 2		1	1
0	0	A bathing area in the c	Yes		2	0	1
0	0	From a basin in a roor	No				
0	0	Latrine	No				
0	0	A bathing area in the c	Yes		3	0	0
1	0	Public bathing facility	No				
0	0	A bathing area in the c	No				
0	0	In a shower/tub/bath r	Yes		3	0	0
0	0	A bathing area in the c	No				
0	0	A bathing area in the c	No				
0	0	A bathing area in the c	No				
0	0	A bathing area in the c	Yes		1	1	0
0	0	Latrine	No				
0	0	A bathing area in the c	No				
1	0	From a basin in a roor	No				
0	0	A bathing area in the c	Yes		1	1	0
0	0	A bathing area in the c	No				
0	0	A bathing area in the c	No				
0	0	A bathing area in the c	No				
1	0	A bathing area outside	No				

0	0	A bathing area in the (	No				
1	0	A bathing area in the (	Yes		3	0	0
0	0	A bathing area in the (	No				
0	0	A bathing area in the (	Yes		1	1	0
0	0	A bathing area in the (	No				
0	0	A bathing area in the (	No				
0	0	Latrine	No				
0	0	A bathing area in the (	No				
0	0	In a shower/tub/bath r	No				
0	0	From a basin in a roor	Yes		1	1	0
0	0	Latrine	No				
0	0	Public bathing facility	No				
0	0	A bathing area in the (	No				
0	0	A bathing area in the (	No				
0	0	A bathing area in the (	No				
0	0	A bathing area in the (	Yes		3	0	0
0	0	In a shower/tub/bath r	No				
0	0	A bathing area in the (	Yes		1	1	0
0	0	Latrine	No				
0	0	A bathing area in the (	No				
0	0	A bathing area in the (	No				
0	0	A bathing area outside	No				
0	0	A bathing area in the (	Yes		1	1	0
0	0	In a shower/tub/bath r	No				
0	0	Latrine	No				
0	0	A bathing area in the (	Yes		3	0	0
0	0	A bathing area in the (	No				
0	0	A bathing area in the (	No				
0	0	Latrine	No				
0	0	A bathing area in the (	No				
0	0	A bathing area in the (	No				
0	0	A bathing area in the (	Yes	2 3		0	1
0	0	A bathing area in the (	No				
1	0	A bathing area in the (	No				
0	0	A bathing area in the (	Yes		1	1	0
0	0	A bathing area in the (	No				
0	0	A bathing area in the (	No				
0	0	A bathing area in the (	No				
0	0	A bathing area in the (	No				
0	0	Latrine	No				
1	0	In a shower/tub/bath r	Yes		3	0	0
0	0	A bathing area in the (	No				
0	0	A bathing area in the (	No				
0	0	A bathing area in the (	No				
0	0	A bathing area in the (	No				
0	0	A bathing area in the (	No				
0	0	A bathing area in the (	Yes		3	0	0
1	0	A bathing area in the (	No				
0	0	A bathing area in the (	No				

0	0	A bathing area in the c	Yes	3	0	0
0	0	A bathing area in the c	Yes	1	1	0
0	0	A bathing area in the c	No			
0	0	A bathing area in the c	No			
1	0	A bathing area in the c	Yes	3	0	0
0	0	A bathing area in the c	No			
0	0	A bathing area in the c	No			
0	0	A bathing area in the c	Yes	3	0	0
0	0	A bathing area in the c	No			
0	0	A bathing area in the c	No			
0	0	Latrine	No			
1	0	Public bathing facility	No			
0	0	A bathing area in the c	No			
0	0	Latrine	No			
0	0	Public bathing facility	No			
0	0	A bathing area in the c	Yes	3	0	0
1	0	A bathing area in the c	No			
0	0	A bathing area outside	Yes	1	1	0
0	0	A bathing area outside	No			
0	0	A bathing area in the c	No			
0	0	A bathing area in the c	No			
0	0	Latrine	No			
0	0	A bathing area in the c	No			
0	0	A bathing area in the c	No			
0	0	A bathing area in the c	No			
0	0	A bathing area in the c	No			
1	0	A bathing area in the c	No			
0	0	A bathing area in the c	No			
0	0	From a basin in a roor	No			
0	0	A bathing area in the c	No			
0	0	A bathing area in the c	No			
0	0	A bathing area in the c	No			
0	0	A bathing area in the c	No			
0	0	A bathing area in the c	No			
0	0	A bathing area in the c	No			
0	0	A bathing area in the c	No			
0	0	A bathing area in the c	No			
0	0	A bathing area in the c	No			
0	0	A bathing area in the c	Yes	2	0	1
0	0	Latrine	No			
0	0	A bathing area in the c	No			
0	0	Latrine	No			
0	0	A bathing area in the c	No			
0	0	A bathing area in the c	No			
0	0	A bathing area in the c	No			
0	0	A bathing area in the c	No			
0	0	In a shower/tub/bath r	No			
0	0	A bathing area in the c	No			
0	0	A bathing area in the c	No			

0	0	Latrine	No			
0	0	From a basin in a roor	No			
0	0	Latrine	Yes	1	1	0
0	0	A bathing area in the (	No			
0	0	Latrine	No			
0	0	A bathing area in the (	No			
0	0	A bathing area in the (	No			
0	0	A bathing area in the (	No			
0	0	A bathing area in the (	Yes	3	0	0
0	0	A bathing area in the (	Yes	3	0	0
0	0	A bathing area in the (	No			
1	0	In a shower/tub/bath r	Yes	3	0	0
0	0	Latrine	No			
0	0	A bathing area in the (	No			
0	0	A bathing area in the (	No			
0	0	A bathing area in the (	Yes	2	0	1
0	0	A bathing area in the (	No			
0	0	A bathing area in the (	No			
0	0	A bathing area in the (	Yes	1	1	0
0	0	Latrine	No			
0	0	A bathing area in the (	No			
0	0	A bathing area in the (	No			
0	0	In a shower/tub/bath r	No			
0	0	In a shower/tub/bath r	Yes	2	0	1
0	0	A bathing area in the (	No			
0	0	Latrine	No			
0	0	In a shower/tub/bath r	No			
0	0	A bathing area in the (	No			
0	0	A bathing area in the (	No			
0	0	Latrine	Yes	2	0	1
0	0	A bathing area in the (	No			
0	0	A bathing area in the (	No			
0	0	Latrine	No			
0	0	A bathing area in the (	Yes	3	0	0
1	0	A bathing area in the (	No			
0	0	From a basin in a roor	No			
0	0	A bathing area in the (	No			
0	0	A bathing area in the (	No			
0	0	A bathing area in the (	No			
0	0	A bathing area in the (	No			
0	0	A bathing area in the (	Yes	3	0	0
0	0	A bathing area in the (	No			
0	0	A bathing area in the (	Yes	1	1	0
0	0	A bathing area in the (	No			
0	0	In a shower/tub/bath r	No			
0	0	Latrine	No			
0	0	A bathing area in the (	No			
0	0	A bathing area in the (	No			
0	0	A bathing area in the (	No			

0	0	A bathing area in the (	No				
0	0	A bathing area in the (	Yes		1	1	0
0	0	A bathing area in the (	No				
0	0	Latrine	No				
0	0	A bathing area in the (	No				
0	0	Latrine	No				
0	0	A bathing area in the (	Yes	2 3		0	1
0	0	A bathing area in the (	No				
1	0	A bathing area in the (	Yes		1	1	0
0	0	Latrine	No				
0	0	A bathing area in the (	No				
0	0	A bathing area in the (	No				
0	0	Latrine	No				
0	0	A bathing area in the (	No				
0	0	A bathing area in the (	No				
0	0	A bathing area in the (	No				
0	0	A bathing area in the (	No				
0	0	Latrine	No				
1	0	A bathing area in the (	No				
0	0	Latrine	No				
0	0	A bathing area in the (	No				
0	0	From a basin in a roor	No				
1	0	A bathing area in the (	No				
1	0	A bathing area in the (	No				
0	0	Latrine	No				
0	0	Latrine	No				
0	0	A bathing area in the (	No				
0	0	A bathing area in the (	No				
0	0	A bathing area in the (	No				
0	0	From a basin in a roor	No				
0	0	A bathing area in the (	No				
0	0	A bathing area in the (	No				
0	0	A bathing area in the (	No				
0	0	A bathing area in the (	No				
0	0	A bathing area in the (	No				
0	0	A bathing area in the (	No				
0	0	A bathing area in the (	Yes		3	0	0
0	0	In a shower/tub/bath r	Yes		2	0	1
0	0	A bathing area in the (	No				
0	0	A bathing area in the (	No				
0	0	A bathing area in the (	Yes		2	0	1
0	0	A bathing area in the (	Yes		2	0	1
0	0	A bathing area in the (	No				
0	0	A bathing area in the (	Yes		2	0	1
0	0	A bathing area in the (	No				
0	0	A bathing area in the (	No				
0	0	A bathing area in the (	Yes		2	0	1
1	0	Public bathing facility	No				
0	0	Latrine	No				

0	0	A bathing area in the (	No				
0	0	Public bathing facility	No				
0	0	A bathing area in the (	Yes	1 2 3		1	1
0	0	Latrine	No				
1	0	A bathing area in the (	Yes		1	1	0
0	0	A bathing area in the (	No				
0	0	A bathing area in the (	No				
0	0	Latrine	No				
0	0	A bathing area in the (	No				
0	0	A bathing area in the (	No				
0	0	From a basin in a roor	No				
0	0	A bathing area in the (	No				
0	0	A bathing area in the (	No				
0	0	A bathing area in the (	No				
0	0	Public bathing facility	Yes	2 3		0	1
0	0	A bathing area in the (	No				
0	0	A bathing area in the (	No				
0	0	A bathing area in the (	Yes		3	0	0
0	0	A bathing area in the (	No				
0	0	A bathing area in the (	No				
0	0	A bathing area in the (	Yes		3	0	0
0	0	A bathing area in the (	No				
0	0	A bathing area in the (	No				
0	0	A bathing area in the (	No				
0	0	A bathing area in the (	No				
0	0	A bathing area in the (	No				
0	0	Latrine	No				
0	0	A bathing area in the (	Yes		1	1	0
0	0	From a basin in a roor	No				
0	0	A bathing area in the (	Yes		3	0	0
0	0	A bathing area in the (	No				
0	0	A bathing area in the (	No				
0	0	A bathing area in the (	No				
0	0	A bathing area in the (	No				
0	0	A bathing area in the (	No				
0	0	A bathing area in the (	Yes		1	1	0
0	0	A bathing area in the (	No				
0	0	A bathing area in the (	No				
0	0	A bathing area in the (	Yes		3	0	0
0	0	A bathing area in the (	No				
0	0	A bathing area in the (	Yes		2	0	1
0	0	A bathing area in the (	No				
0	0	Latrine	No				
0	0	A bathing area in the (	No				
0	0	A bathing area in the (	No				
0	0	A bathing area in the (	No				
0	0	A bathing area in the (	No				
0	0	A bathing area in the (	Yes		1	1	0
0	0	Latrine	No				

0	0	In a shower/tub/bath r	No				
0	0	From a basin in a roor	No				
0	0	A bathing area in the (	Yes	1 2 3		1	1
0	0	In a shower/tub/bath r	No				
0	0	A bathing area in the (	No				
0	0	A bathing area in the (	No				
0	0	A bathing area in the (	No				
0	0	A bathing area in the (	No				
0	0	In a shower/tub/bath r	No				
0	0	A bathing area in the (	No				
0	0	Public bathing facility	Yes		2	0	1
0	0	A bathing area in the (	No				
0	0	From a basin in a roor	No				
0	0	A bathing area in the (	Yes		3	0	0
0	0	A bathing area in the (	No				
0	0	A bathing area in the (	No				
0	0	A bathing area in the (	No				
0	0	A bathing area in the (	No				
0	0	A bathing area in the (	No				
0	0	A bathing area in the (	No				
0	0	A bathing area in the (	No				
0	0	A bathing area in the (	No				
0	0	In a shower/tub/bath r	No				
0	0	In a shower/tub/bath r	No				
0	0	A bathing area in the (	Yes		1	1	0
0	0	A bathing area in the (	Yes		2	0	1
0	0	Latrine	No				
0	0	A bathing area in the (	No				
0	0	A bathing area in the (	No				
1	0	A bathing area in the (	No				
1	0	From a basin in a roor	No				
0	0	A bathing area in the (	No				
0	0	A bathing area in the (	No				
0	0	A bathing area in the (	No				
0	0	A bathing area in the (	No				
1	0	Public bathing facility	No				
0	0	Latrine	No				
0	0	A bathing area in the (	Yes		1	1	0
0	0	In a shower/tub/bath r	No				
0	0	A bathing area in the (	No				
0	0	A bathing area in the (	Yes		2	0	1
0	0	A bathing area in the (	Yes		3	0	0
0	0	A bathing area in the (	No				
0	0	A bathing area in the (	No				
0	0	A bathing area in the (	No				
0	0	A bathing area in the (	No				
0	0	A bathing area in the (	No				
0	0	In a shower/tub/bath r	No				
0	0	A bathing area in the (	Yes		3	0	0

0	0	A bathing area in the c	No				
0	0	In a shower/tub/bath r	No				
0	0	A bathing area in the c	No				
0	0	Public bathing facility	No				
0	0	A bathing area in the c	No				
1	0	A bathing area in the c	No				
0	0	In a shower/tub/bath r	No				
0	0	A bathing area in the c	No				
0	0	A bathing area in the c	No				
1	0	A bathing area in the c	No				
0	0	In a shower/tub/bath r	No				
0	0	A bathing area in the c	No				
0	0	A bathing area in the c	No				
0	0	A bathing area in the c	No				
0	0	A bathing area in the c	No				
0	0	A bathing area in the c	Yes		3	0	0
0	0	A bathing area in the c	No				
0	0	A bathing area in the c	No				
0	0	A bathing area in the c	No				
0	0	A bathing area in the c	Yes	1 3		1	0
0	0	A bathing area in the c	Yes		3	0	0
0	0	A bathing area in the c	No				
0	0	From a basin in a roor	No				
0	0	Latrine	No				
0	0	A bathing area in the c	No				

q68_3	q69	q70a	q70b	q71	q72a	q72b
	5	None		Yes	Flush to pit latrine	
	6	None		Yes	Pit latrine with concrete slab	
	5	A brick/stone wall and		Yes	Flush to septic tank	
	5	None		Yes	Flush to pit latrine	
	1	Iron sheet enclosure a		Yes	Flush to pit latrine	
1	57	Iron sheet enclosure a		Yes	Flush to Sewer system	
1	1	A brick/stone wall		Yes	Flush to pit latrine	
	11	A brick/stone wall		Yes	Flush to pit latrine	
	5	None		Yes	Pit latrine with concrete slab	
	3	A brick/stone wall and		Yes	Pit latrine with concrete slab	
	56	Iron sheet enclosure a		No		
1	15	Iron sheet enclosure a		Yes	Flush to septic tank	
	1	Iron sheet enclosure a		Yes	Flush to pit latrine	
	5	Iron sheet enclosure a		Yes	Pit latrine with concrete slab	
1	9	Wire/timber/mud/stone		Yes	Pit latrine with concrete slab	
1	30	A brick/stone wall and		Yes	Pit latrine with concrete slab	
	33	Iron sheet enclosure a		Yes	Pit latrine with concrete slab	
	2	None		Yes	Flush to septic tank	
	48	Iron sheet enclosure a		Yes	Flush to pit latrine	
	1	None		Yes	Flush to pit latrine	
1	22	Iron sheet enclosure a		Yes	Flush to pit latrine	
	23	A brick/stone wall and		Yes	Pit latrine with concrete slab	
	8	Iron sheet enclosure a		Yes	Pit latrine with concrete slab	
	1	None		Yes	Pit latrine with concrete slab	
	10	A brick/stone wall and		Yes	Pit latrine with concrete slab	
	6	Iron sheet enclosure a		Yes	Pit latrine with concrete slab	
	1	A brick/stone wall		Yes	Pit latrine with concrete slab	
	24	Iron sheet enclosure a		Yes	No facility/bush/field	
	3	None		Yes	Pit latrine with concrete slab	
	1	None		Yes	Flush to septic tank	
	12	A brick/stone wall and		Yes	Pit latrine with concrete slab	
	1	None		Yes	Flush to septic tank	
1	30	A brick/stone wall and		Yes	Flush to septic tank	
	1	None		Yes	Flush to septic tank	
	1	None		Yes	Pit latrine with concrete slab	
	1	None		No		
	30	A brick/stone wall and		Yes	Flush to pit latrine	
1	1	None		Yes	Flush to septic tank	
1	5	Other (spe	Metallic Ga	No		
	3	A brick/stone wall		Yes	Flush to pit latrine	
	23	None		Yes	Pit latrine with concrete slab	
	4	None		Yes	Flush to pit latrine	
1	1	None		Yes	Pit latrine with concrete slab	
	1	None		Yes	Pit latrine with concrete slab	
	32	A brick/stone wall and		Yes	Flush to Sewer system	
	30	Iron sheet enclosure a		Yes	Bag	
	15	Wire/timber/mud/stone		Yes	Flush to Sewer system	
	1	None		No		

	1	A brick/stone wall	Yes	Flush to pit latrine
	8	A brick/stone wall and	Yes	Flush to pit latrine
	1	None	No	
	1	A brick/stone wall	Yes	Flush to pit latrine
	1	None	Yes	Flush to septic tank
1	69	Iron sheet enclosure a	Yes	Pit latrine with concrete slab
	1	None	Yes	Pit latrine with concrete slab
	1	A brick/stone wall	Yes	Flush to Sewer system
	1	None	Yes	Flush to septic tank
	3	A brick/stone wall and	Yes	Pit latrine with concrete slab
	1	A brick/stone wall	Yes	Flush to pit latrine
	9	None	Yes	Flush to septic tank
	14	Iron sheet enclosure a	Yes	Pit latrine with concrete slab
	8	A brick/stone wall and	Yes	Flush to pit latrine
	1	None	Yes	Pit latrine with concrete slab
	8	A brick/stone wall	Yes	Pit latrine with concrete slab
	6	A brick/stone wall and	Yes	Flush to Sewer system
	17	Iron sheet enclosure a	Yes	Flush to Sewer system
	45	A brick/stone wall and	Yes	Flush to Sewer system
	105	A brick/stone wall and	Yes	Flush to pit latrine
0	9	Iron sheet enclosure a	Yes	Pit latrine with concrete slab
	4	Iron sheet enclosure a	Yes	Flush to pit latrine
	45	Iron sheet enclosure a	Yes	Flush to Sewer system
	4	None	No	
	34	Iron sheet enclosure a	Yes	Pit latrine with concrete slab
	1	None	Yes	Flush to septic tank
	3	None	Yes	Flush to pit latrine
	3	A brick/stone wall and	Yes	Flush to septic tank
	1	None	No	
	8	None	Yes	Pit latrine with concrete slab
	1	None	Yes	Flush to pit latrine
	3	None	Yes	Pit latrine with concrete slab
1	1	None	Yes	Pit latrine with concrete slab
0	3	Iron sheet enclosure a	Yes	Pit latrine with concrete slab
	2	None	Yes	Pit latrine with concrete slab
0	1	None	Yes	Pit latrine with concrete slab
	6	A brick/stone wall	Yes	Flush to pit latrine
	1	None	Yes	Flush to septic tank
0	11	Iron sheet enclosure a	Yes	Pit latrine with concrete slab
	1	None	Yes	Flush to pit latrine
	55	Iron sheet enclosure a	Yes	Flush to Sewer system
	4	Iron sheet enclosure a	Yes	Flush to pit latrine
	4	A brick/stone wall and	Yes	Flush to septic tank
	1	None	Yes	Flush to pit latrine
	2	None	Yes	Flush to pit latrine
	8	A brick/stone wall and	Yes	Flush to septic tank
	8	None	Yes	Flush to pit latrine
	1	None	Yes	Flush to septic tank
	12	None	Yes	Pit latrine with concrete slab

0	10	None	Yes	Flush to septic tank
	1	A brick/stone wall	Yes	Flush donâ€™t know where
	14	None	Yes	Flush to septic tank
	3	None	Yes	Pit latrine with concrete slab
0	4	Iron sheet enclosure a	Yes	Flush to pit latrine
	1	None	Yes	Pit latrine with concrete slab
	2	None	Yes	Pit latrine with concrete slab
	1	None	Yes	Flush to pit latrine
	9	Wire/timber/mud/stone	Yes	Flush to septic tank
	1	None	Yes	Pit latrine with concrete slab
	6	Iron sheet enclosure a	Yes	Flush to pit latrine
	2	Iron sheet enclosure a	Yes	Flush to septic tank
	3	A brick/stone wall and	Yes	Pit latrine with concrete slab
	3	A brick/stone wall	Yes	Flush to pit latrine
	1	None	No	
	1	None	No	
	10	Iron sheet enclosure a	Yes	Pit latrine with concrete slab
1	1	None	Yes	Flush to septic tank
	9	None	Yes	Flush to onsite, open pit
	6	A brick/stone wall and	Yes	Flush to septic tank
	5	A brick/stone wall and	Yes	Pit latrine without slab
	7	None	Yes	Flush to pit latrine
1	1	None	Yes	Flush to septic tank
	1	Wire/timber/mud/stone	Yes	Flush to septic tank
	1	None	Yes	Flush to septic tank
	7	None	Yes	Pit latrine with concrete slab
	1	A brick/stone wall and	Yes	Flush to septic tank
	11	None	Yes	Pit latrine with concrete slab
	2	A brick/stone wall and	Yes	Flush to Sewer system
	20	A brick/stone wall and	Yes	Flush to septic tank
	1	A brick/stone wall	Yes	Pit latrine with concrete slab
	51	A brick/stone wall and	Yes	Ventilated improved pit latrine
	7	A brick/stone wall and	Yes	Flush to septic tank
	15	Iron sheet enclosure a	Yes	Ventilated improved pit latrine
	1	None	Yes	Pit latrine with concrete slab
	7	A brick/stone wall and	Yes	Flush to pit latrine
0	14	Iron sheet enclosure a	Yes	Pit latrine with concrete slab
	5	Iron sheet enclosure a	Yes	Pit latrine with concrete slab
	1	A brick/stone wall	Yes	Pit latrine with concrete slab
	1	A brick/stone wall	Yes	Flush to septic tank
	1	None	Yes	Flush to septic tank
	1	A brick/stone wall and	Yes	Flush to septic tank
	45	Iron sheet enclosure a	Yes	Flush to Sewer system
	3	Wire/timber/mud/stone	Yes	Flush to septic tank
0	9	Iron sheet enclosure a	Yes	Pit latrine with concrete slab
1	1	A brick/stone wall	Yes	Flush to pit latrine
	1	None	Yes	Pit latrine with concrete slab
	1	None	Yes	Pit latrine with concrete slab
	8	Iron sheet enclosure a	Yes	Pit latrine with concrete slab

1	1	None	Yes	Pit latrine with concrete slab
	1	None	No	
	5	A brick/stone wall and	Yes	Pit latrine with concrete slab
	12	A brick/stone wall	Yes	Flush to pit latrine
	1	Iron sheet enclosure a	Yes	Flush to septic tank
	4	A brick/stone wall and	Yes	Flush to septic tank
	31	Iron sheet enclosure a	Yes	Flush to septic tank
1	14	Iron sheet enclosure a	Yes	Pit latrine with concrete slab
	35	Iron sheet enclosure a	Yes	Flush to Sewer system
	1	Wire/timber/mud/stone	Yes	Pit latrine with concrete slab
	30	A brick/stone wall and	Yes	Flush to pit latrine
1	7	Iron sheet enclosure a	Yes	Pit latrine with concrete slab
	4	None	Yes	Flush to pit latrine
	7	A brick/stone wall and	Yes	Flush to septic tank
	1	None	Yes	Flush to septic tank
0	3	None	No	
	6	A brick/stone wall and	No	
	3	None	Yes	Pit latrine with concrete slab
	1	None	Yes	Flush to septic tank
	1	None	Yes	Flush to septic tank
	4	A brick/stone wall and	Yes	Pit latrine with concrete slab
1	1	None	Yes	Flush to septic tank
	1	None	Yes	Flush to pit latrine
	8	Iron sheet enclosure a	Yes	Flush to pit latrine
	9	None	Yes	Pit latrine with concrete slab
	3	None	Yes	Pit latrine without slab
	6	None	Yes	Pit latrine with concrete slab
0	3	None	No	
	1	None	Yes	Flush to septic tank
	1	None	Yes	Pit latrine with concrete slab
0	3	A brick/stone wall	Yes	Flush to pit latrine
	12	None	Yes	Flush to septic tank
	1	None	Yes	Flush to pit latrine
	1	None	Yes	Pit latrine with concrete slab
	1	A brick/stone wall and	Yes	Flush to septic tank
1	1	None	Yes	Pit latrine with concrete slab
	1	None	Yes	Pit latrine with concrete slab
	35	Iron sheet enclosure a	Yes	Flush to pit latrine
	15	A brick/stone wall and	Yes	Pit latrine with concrete slab
	7	None	Yes	Flush to septic tank
	1	None	Yes	Pit latrine with concrete slab
0	4	None	No	
	5	Iron sheet enclosure a	Yes	Pit latrine with concrete slab
	22	A brick/stone wall and	Yes	Flush to pit latrine
	1	None	Yes	Pit latrine with concrete slab
	1	None	Yes	Flush to septic tank
	1	None	Yes	Flush to septic tank
	51	A brick/stone wall and	Yes	Flush to pit latrine
	1	None	Yes	Flush to septic tank

0	8	A brick/stone wall	Yes	Pit latrine with concrete slab
	1	None	Yes	Flush to Sewer system
0	4	None	Yes	Pit latrine with concrete slab
	1	None	Yes	Flush to septic tank
	2	None	No	
	8	A brick/stone wall and	Yes	Flush to septic tank
	5	None	Yes	Pit latrine with concrete slab
	1	None	Yes	Pit latrine with concrete slab
	1	None	Yes	Flush to septic tank
	33	A brick/stone wall and	Yes	Flush to pit latrine
	1	None	Yes	Flush to septic tank
	5	A brick/stone wall and	Yes	Flush to septic tank
	3	Iron sheet enclosure a	Yes	Flush to pit latrine
	10	Iron sheet enclosure a	Yes	Pit latrine with concrete slab
	1	A brick/stone wall	Yes	Flush to pit latrine
	4	None	Yes	Pit latrine with concrete slab
	1	None	Yes	Flush to septic tank
	25	Iron sheet enclosure a	Yes	Flush donâ€™t know where
	30	Iron sheet enclosure a	Yes	Flush to Sewer system
0	1	None	Yes	Flush to pit latrine
	1	None	Yes	Flush to septic tank
	46	Iron sheet enclosure a	Yes	Flush to Sewer system
	4	A brick/stone wall and	Yes	Pit latrine without slab
	1	None	Yes	Pit latrine without slab
	1	None	Yes	Flush to septic tank
	6	Wire/timber/mud/stone	Yes	Pit latrine with concrete slab
	1	None	Yes	Pit latrine with concrete slab
	1	None	No	
	15	A brick/stone wall and	Yes	Ventilated improved pit latrine
	63	A brick/stone wall and	Yes	Pit latrine with concrete slab
	1	None	Yes	Pit latrine with concrete slab
0	1	None	Yes	Flush to septic tank
	46	Iron sheet enclosure a	Yes	Pit latrine with concrete slab
	1	None	Yes	Pit latrine with concrete slab
	24	Iron sheet enclosure a	Yes	Flush to Sewer system
	3	None	Yes	Pit latrine with concrete slab
	1	None	No	
1	6	None	Yes	Flush to pit latrine
	3	None	Yes	Flush to pit latrine
	1	A brick/stone wall	Yes	Flush to Sewer system
	7	Wire/timber/mud/stone	Yes	Flush to septic tank
	2	A brick/stone wall	Yes	Pit latrine with concrete slab
	1	None	Yes	Flush to septic tank
	1	A brick/stone wall and	Yes	Flush to septic tank
	2	None	Yes	Flush to pit latrine
	11	Iron sheet enclosure a	Yes	Flush to pit latrine
	45	Iron sheet enclosure a	Yes	Flush to Sewer system
	9	None	Yes	Flush to septic tank
1	14	Iron sheet enclosure a	Yes	Pit latrine with concrete slab

	1	None	No	
	3	A brick/stone wall and	No	
	9	A brick/stone wall and	Yes	Flush to pit latrine
	8	Iron sheet enclosure a	No	
	9	A brick/stone wall and	Yes	Flush to septic tank
0	43	Wire/timber/mud/stone	Yes	Flush to septic tank
	1	None	Yes	Pit latrine with concrete slab
1	16	None	No	
	1	A brick/stone wall and	Yes	Flush to septic tank
	1	None	Yes	Pit latrine with concrete slab
	8	Iron sheet enclosure a	Yes	Pit latrine with concrete slab
	1	A brick/stone wall	Yes	Flush to Sewer system
	6	Iron sheet enclosure a	Yes	Pit latrine with concrete slab
	1	None	Yes	Flush to pit latrine
	7	A brick/stone wall	Yes	Flush to Sewer system
	7	Iron sheet enclosure a	Yes	Flush to Sewer system
	1	None	Yes	Flush to septic tank
	23	Wire/timber/mud/stone	Yes	Flush to pit latrine
	1	None	Yes	Pit latrine with concrete slab
	7	Iron sheet enclosure a	Yes	Pit latrine with concrete slab
	1	Wire/timber/mud/stone	Yes	Pit latrine with concrete slab
	4	A brick/stone wall	Yes	Pit latrine with concrete slab
	1	None	Yes	Flush to septic tank
	9	A brick/stone wall and	Yes	Flush donâ€™t know where
0	98	A brick/stone wall and	Yes	Flush to pit latrine
1	1	None	Yes	Pit latrine with concrete slab
	16	None	Yes	Flush to pit latrine
	1	None	Yes	Flush to Sewer system
	1	Iron sheet enclosure a	Yes	Pit latrine with concrete slab
	10	Wire/timber/mud/stone	Yes	Pit latrine with concrete slab
	20	A brick/stone wall and	Yes	Pit latrine with concrete slab
	1	Iron sheet enclosure a	Yes	Flush to septic tank
	25	A brick/stone wall and	Yes	Flush to Sewer system
	1	None	Yes	Flush to septic tank
	6	A brick/stone wall and	Yes	Flush to pit latrine
1	4	None	Yes	Pit latrine with concrete slab
1	30	Iron sheet enclosure a	Yes	Flush to Sewer system
1	14	A brick/stone wall and	Yes	Flush to septic tank
	15	A brick/stone wall and	Yes	Flush to pit latrine
	1	None	Yes	Flush to septic tank
	30	Wire/timber/mud/stone	Yes	Flush to septic tank
	1	None	Yes	Pit latrine with concrete slab
	2	None	Yes	Pit latrine with concrete slab
	1	None	No	
	1	None	Yes	Flush to septic tank
	10	Iron sheet enclosure a	Yes	Pit latrine with concrete slab
	130	A brick/stone wall and	Yes	Flush to Sewer system
	13	A brick/stone wall and	Yes	Ventilated improved pit latrine
	18	A brick/stone wall and	Yes	Pit latrine with concrete slab

	18	A brick/stone wall and	Yes	Flush to pit latrine	
0	2	None	No		
	28	Iron sheet enclosure a	Yes	Flush to Sewer system	
	4	None	Yes	Flush to septic tank	
	9	Wire/timber/mud/stone	Yes	Pit latrine with concrete slab	
	4	Iron sheet enclosure a	Yes	Pit latrine with concrete slab	
	3	Iron sheet enclosure a	Yes	Pit latrine with concrete slab	
	7	None	Yes	Pit latrine with concrete slab	
1	1	None	Yes	Flush to septic tank	
0	10	Iron sheet enclosure a	Yes	Pit latrine with concrete slab	
	9	Iron sheet enclosure a	Yes	Other (Spe	Flush/pour toilet in another compo
	1	None	Yes	Flush to septic tank	
0	18	A brick/stone wall and	Yes	Pit latrine with concrete slab	
	1	None	Yes	Flush to septic tank	
	15	None	Yes	Pit latrine without slab	
	20	A brick/stone wall and	Yes	Flush to Sewer system	
	27	A brick/stone wall and	Yes	Flush to septic tank	
	4	A brick/stone wall	Yes	Flush to Sewer system	
	7	Iron sheet enclosure a	Yes	Flush donâ€™t know where	
1	10	Iron sheet enclosure a	Yes	Pit latrine with concrete slab	
	1	None	Yes	Flush to pit latrine	
	9	None	Yes	Flush to pit latrine	
	2	A brick/stone wall and	Yes	Pit latrine with concrete slab	
	1	A brick/stone wall and	Yes	Pit latrine without slab	
0	4	None	Yes	Pit latrine with concrete slab	
	35	A brick/stone wall and	Yes	Flush to pit latrine	
	21	A brick/stone wall and	Yes	Pit latrine with concrete slab	
	4	None	Yes	Flush to septic tank	
	2	A brick/stone wall and	No		
	17	A brick/stone wall and	Yes	Flush to Sewer system	
	1	A brick/stone wall	Yes	Flush to pit latrine	
	3	Iron sheet enclosure a	Yes	Pit latrine with concrete slab	
	12	A brick/stone wall and	Yes	Flush to Sewer system	
	5	None	Yes	Pit latrine with concrete slab	
	4	A brick/stone wall and	Yes	Flush to septic tank	
	1	None	Yes	Flush to septic tank	
	2	Wire/timber/mud/stone	Yes	Flush to pit latrine	
	12	A brick/stone wall and	Yes	Flush to pit latrine	
	15	Iron sheet enclosure a	Yes	Pit latrine with concrete slab	
	8	Iron sheet enclosure a	Yes	Pit latrine with concrete slab	
	4	Wire/timber/mud/stone	No		
	32	A brick/stone wall and	Yes	Flush to septic tank	
	42	A brick/stone wall and	Yes	Flush to septic tank	
	10	None	Yes	Pit latrine with concrete slab	
	20	A brick/stone wall and	Yes	Pit latrine with concrete slab	
	22	A brick/stone wall and	Yes	Flush donâ€™t know where	
	10	None	Yes	Pit latrine with concrete slab	
	6	None	Yes	Pit latrine with concrete slab	
	7	Iron sheet enclosure a	Yes	Pit latrine with concrete slab	

	58	A brick/stone wall and	Yes	Flush to pit latrine
	23	A brick/stone wall and	Yes	Pit latrine with concrete slab
	3	None	Yes	Flush to pit latrine
	20	A brick/stone wall and	Yes	Flush to pit latrine
	3	A brick/stone wall	No	
	5	None	Yes	Flush to onsite, open pit
0	47	A brick/stone wall and	Yes	Pit latrine with concrete slab
1	5	None	Yes	Pit latrine with concrete slab
0	12	A brick/stone wall and	Yes	Flush to Sewer system
	15	None	Yes	Flush to onsite, open pit
	15	Iron sheet enclosure a	Yes	Pit latrine with concrete slab
	1	None	Yes	Flush to septic tank
	1	None	No	
	6	None	Yes	Pit latrine without slab
1	7	Iron sheet enclosure a	Yes	Pit latrine with concrete slab
	20	A brick/stone wall and	No	
	10	A brick/stone wall and	Yes	Flush to septic tank
	22	A brick/stone wall and	Yes	Flush to Sewer system
	4	A brick/stone wall and	Yes	Pit latrine with concrete slab
	5	None	Yes	Pit latrine with concrete slab
1	5	Iron sheet enclosure a	Yes	Pit latrine with concrete slab
	33	A brick/stone wall and	Yes	Flush to Sewer system
	25	Iron sheet enclosure a	Yes	Flush to Sewer system
	10	A brick/stone wall and	Yes	Flush to pit latrine
	12	Iron sheet enclosure a	Yes	Flush to septic tank
	23	Iron sheet enclosure a	Yes	Flush to Sewer system
	3	A brick/stone wall and	Yes	Flush to septic tank
	3	A brick/stone wall	Yes	Flush to pit latrine
	1	None	No	
1	6	Iron sheet enclosure a	Yes	Pit latrine with concrete slab
	10	Wire/timber/mud/stone	Yes	Flush to Sewer system
	45	Iron sheet enclosure a	Yes	Flush to Sewer system
	8	Iron sheet enclosure a	Yes	Pit latrine with concrete slab
0	1	None	Yes	Flush to septic tank
	11	None	Yes	Flush to septic tank
1	3	Iron sheet enclosure a	Yes	Pit latrine with concrete slab
	20	Iron sheet enclosure a	Yes	Flush to Sewer system
	10	A brick/stone wall and	Yes	Pit latrine with concrete slab
	12	None	Yes	Flush to pit latrine
	4	Iron sheet enclosure a	Yes	Pit latrine with concrete slab
1	11	Iron sheet enclosure a	Yes	Pit latrine with concrete slab
	10	None	Yes	Pit latrine with concrete slab
0	4	None	Yes	Pit latrine with concrete slab
	12	Iron sheet enclosure a	No	
	6	A brick/stone wall and	Yes	Flush to septic tank
	40	Iron sheet enclosure a	Yes	Flush to Sewer system
1	30	Iron sheet enclosure a	Yes	Flush to Sewer system
	15	Iron sheet enclosure a	Yes	Pit latrine with concrete slab
	5	None	Yes	Pit latrine with concrete slab

	1	A brick/stone wall and	Yes	Flush to septic tank
0	54	Iron sheet enclosure a	Yes	Flush to septic tank
	25	Iron sheet enclosure a	Yes	Flush to Sewer system
	20	A brick/stone wall and	Yes	Flush to Sewer system
	30	A brick/stone wall and	Yes	Flush to Sewer system
	31	A brick/stone wall and	Yes	Pit latrine with concrete slab
	5	A brick/stone wall and	Yes	Flush to septic tank
	24	A brick/stone wall and	No	
	60	A brick/stone wall and	Yes	Flush to Sewer system
0	30	A brick/stone wall and	Yes	Flush donâ€™t know where
	7	Iron sheet enclosure a	No	
1	11	Wire/timber/mud/stone	Yes	Pit latrine with concrete slab
	6	A brick/stone wall and	Yes	Flush to septic tank
	4	Iron sheet enclosure a	Yes	Pit latrine with concrete slab
	25	A brick/stone wall and	Yes	Flush to Sewer system
1	25	None	Yes	Flush to Sewer system
	28	A brick/stone wall and	Yes	Flush to septic tank
0	32	A brick/stone wall and	Yes	Flush to pit latrine
	35	Iron sheet enclosure a	Yes	Pit latrine with concrete slab
1	11	Other (spe Metallic ga	Yes	Pit latrine with concrete slab
	12	A brick/stone wall and	Yes	Pit latrine with concrete slab
	31	A brick/stone wall and	Yes	Flush to septic tank
	2	None	Yes	Pit latrine with concrete slab
	8	None	Yes	Pit latrine with concrete slab
	10	A brick/stone wall and	Yes	Pit latrine with concrete slab
	7	Iron sheet enclosure a	Yes	Flush to Sewer system
0	7	Iron sheet enclosure a	Yes	Pit latrine with concrete slab
	1	A brick/stone wall and	Yes	Flush to septic tank
	10	None	Yes	Pit latrine without slab
	10	Iron sheet enclosure a	Yes	Pit latrine with concrete slab
	1	A brick/stone wall and	Yes	Flush to Sewer system
	7	Iron sheet enclosure a	Yes	Pit latrine with concrete slab
	3	Iron sheet enclosure a	Yes	Pit latrine without slab
	5	Iron sheet enclosure a	Yes	Pit latrine with concrete slab
	12	Iron sheet enclosure a	Yes	Pit latrine with concrete slab
0	8	None	Yes	Pit latrine with concrete slab
	14	Iron sheet enclosure a	Yes	Pit latrine with concrete slab
	3	A brick/stone wall and	Yes	Flush to septic tank
	45	Iron sheet enclosure a	Yes	Flush to Sewer system
	1	None	Yes	Flush to septic tank
	5	Iron sheet enclosure a	No	
	3	A brick/stone wall and	Yes	Flush to pit latrine
1	8	Iron sheet enclosure a	No	
	12	Iron sheet enclosure a	Yes	Flush to Sewer system
	24	Iron sheet enclosure a	Yes	Flush to Sewer system
	1	Iron sheet enclosure a	Yes	Pit latrine with concrete slab
	10	A brick/stone wall and	Yes	Pit latrine with concrete slab
	2	None	Yes	Flush to septic tank
	120	Iron sheet enclosure a	Yes	Bag

	28	Iron sheet enclosure a	Yes	Flush to Sewer system
	23	A brick/stone wall and	Yes	Flush to Sewer system
	81	A brick/stone wall and	Yes	Flush to Sewer system
	6	None	Yes	Pit latrine with concrete slab
	18	A brick/stone wall	Yes	Pit latrine with concrete slab
	12	Iron sheet enclosure a	No	
1	10	None	Yes	Pit latrine with concrete slab
	8	Iron sheet enclosure a	Yes	Flush to Sewer system
	17	A brick/stone wall and	Yes	Flush to pit latrine
1	6	Wire/timber/mud/stone	Yes	Pit latrine without slab
	5	Iron sheet enclosure a	Yes	Pit latrine with concrete slab
	6	A brick/stone wall and	Yes	Flush to septic tank
	1	None	No	
1	6	Iron sheet enclosure a	Yes	Pit latrine with concrete slab
	24	A brick/stone wall and	Yes	Flush to pit latrine
0	1	A brick/stone wall and	Yes	Flush to pit latrine
	1	Iron sheet enclosure a	Yes	Pit latrine with concrete slab
	26	None	Yes	Pit latrine with concrete slab
	1	None	Yes	Flush to pit latrine
1	1	None	Yes	Flush to pit latrine
	60	Iron sheet enclosure a	Yes	Pit latrine with concrete slab
0	6	Iron sheet enclosure a	Yes	Pit latrine with concrete slab
	2	None	Yes	Flush to septic tank
	15	A brick/stone wall and	Yes	Pit latrine with concrete slab
	14	None	Yes	Pit latrine with concrete slab
	1	None	Yes	Pit latrine with concrete slab
	1	None	Yes	Flush to septic tank
	9	Iron sheet enclosure a	Yes	Pit latrine with concrete slab
	6	None	Yes	Pit latrine with concrete slab
1	10	Iron sheet enclosure a	Yes	Pit latrine with concrete slab
0	8	None	Yes	Pit latrine with concrete slab
0	20	Iron sheet enclosure a	Yes	Pit latrine with concrete slab
	11	Iron sheet enclosure a	Yes	Flush to pit latrine
	30	None	Yes	Flush to pit latrine
1	15	Iron sheet enclosure a	Yes	Flush to septic tank
	3	A brick/stone wall and	Yes	Flush to septic tank
	1	None	Yes	Pit latrine without slab
	4	A brick/stone wall and	Yes	Pit latrine with concrete slab
	1	None	Yes	Flush to septic tank
	29	A brick/stone wall and	Yes	Flush to Sewer system
	6	A brick/stone wall and	Yes	Flush to septic tank
	6	A brick/stone wall and	Yes	Flush to septic tank
	5	A brick/stone wall and	Yes	Flush to pit latrine
0	6	Iron sheet enclosure a	Yes	Pit latrine with concrete slab
	5	A brick/stone wall and	Yes	Flush to Sewer system
	35	Iron sheet enclosure a	Yes	Flush to Sewer system
	10	A brick/stone wall	No	
	16	A brick/stone wall and	Yes	Flush to septic tank
	32	A brick/stone wall	Yes	Flush to Sewer system

	22	A brick/stone wall and	Yes	Flush to Sewer system
	5	Iron sheet enclosure a	Yes	Pit latrine with concrete slab
0	17	Iron sheet enclosure a	Yes	Flush to Sewer system
	9	Iron sheet enclosure a	Yes	Pit latrine with concrete slab
	17	Iron sheet enclosure a	Yes	Flush to Sewer system
	1	None	Yes	Flush to septic tank
	1	None	Yes	Flush to Sewer system
	10	A brick/stone wall and	Yes	Flush to septic tank
	39	A brick/stone wall	Yes	Flush to Sewer system
1	73	Iron sheet enclosure a	Yes	Pit latrine with concrete slab
0	6	Iron sheet enclosure a	Yes	Pit latrine with concrete slab
	14	A brick/stone wall	Yes	Pit latrine with concrete slab
0	9	None	Yes	Pit latrine with concrete slab
	1	None	Yes	Pit latrine with concrete slab
0	5	None	Yes	Pit latrine with concrete slab
	8	None	Yes	Pit latrine with concrete slab
	8	None	Yes	Flush to septic tank
	13	A brick/stone wall and	No	
0	6	None	Yes	Pit latrine with concrete slab
	11	A brick/stone wall and	Yes	Flush to pit latrine
1	45	A brick/stone wall and	Yes	Flush to Sewer system
	7	A brick/stone wall and	Yes	Flush to septic tank
	18	A brick/stone wall and	Yes	Flush to septic tank
	2	A brick/stone wall and	Yes	Pit latrine with concrete slab
	10	A brick/stone wall	Yes	Pit latrine with concrete slab
	13	Iron sheet enclosure a	Yes	Pit latrine with concrete slab
	3	A brick/stone wall and	Yes	Pit latrine with concrete slab
	10	Iron sheet enclosure a	Yes	Pit latrine with concrete slab
	10	Iron sheet enclosure a	Yes	Flush to Sewer system
	6	Wire/timber/mud/stone	Yes	Flush to septic tank
	2	None	Yes	Pit latrine with concrete slab
0	47	Iron sheet enclosure a	Yes	Flush to Sewer system
	14	Iron sheet enclosure a	Yes	Flush to Sewer system
	32	Iron sheet enclosure a	Yes	Flush to Sewer system
	1	None	Yes	Flush to septic tank
	54	Iron sheet enclosure a	Yes	Pit latrine with concrete slab
	11	A brick/stone wall	Yes	Flush to pit latrine
	15	Wire/timber/mud/stone	Yes	Pit latrine with concrete slab
	22	Iron sheet enclosure a	Yes	Flush to Sewer system
	50	Iron sheet enclosure a	Yes	Pit latrine with concrete slab
	3	A brick/stone wall and	Yes	Flush to septic tank
	10	Iron sheet enclosure a	Yes	Pit latrine with concrete slab
	31	A brick/stone wall and	Yes	Flush to Sewer system
	60	A brick/stone wall and	Yes	Flush to septic tank
1	8	None	Yes	Pit latrine with concrete slab
	1	None	Yes	Flush to septic tank
	14	Iron sheet enclosure a	Yes	Flush to septic tank
	6	None	Yes	Flush to Sewer system
	37	A brick/stone wall and	Yes	Flush to Sewer system

	13	A brick/stone wall and	Yes	Flush to somewhere else
	5	Iron sheet enclosure a	Yes	Pit latrine with concrete slab
	30	A brick/stone wall and	Yes	Flush to Sewer system
	30	A brick/stone wall and	Yes	Pit latrine with concrete slab
	20	A brick/stone wall and	Yes	Flush to pit latrine
	32	None	Yes	Flush to septic tank
1	4	None	Yes	Pit latrine with concrete slab
	3	None	Yes	Pit latrine with concrete slab
	1	Iron sheet enclosure a	Yes	Flush to septic tank
	7	None	Yes	Flush to pit latrine
	1	None	Yes	Pit latrine with concrete slab
	5	Iron sheet enclosure a	Yes	Pit latrine without slab
	6	A brick/stone wall and	Yes	Flush to septic tank
	8	None	Yes	Pit latrine without slab
	1	None	Yes	Flush to Sewer system
	57	Iron sheet enclosure a	Yes	Flush to Sewer system
	9	A brick/stone wall and	Yes	Pit latrine without slab
	10	A brick/stone wall and	Yes	Pit latrine with concrete slab
	1	None	Yes	Flush to septic tank
0	9	Iron sheet enclosure a	Yes	Flush to pit latrine
	20	A brick/stone wall and	Yes	Flush to Sewer system
	1	None	No	
	10	Iron sheet enclosure a	Yes	Flush to septic tank
1	2	Iron sheet enclosure a	Yes	Pit latrine with concrete slab
0	16	A brick/stone wall	Yes	Flush to septic tank
0	10	None	Yes	Pit latrine with concrete slab
	1	None	Yes	Flush to septic tank
	10	Iron sheet enclosure a	Yes	Flush to septic tank
	4	Wire/timber/mud/stone	Yes	Pit latrine without slab
	32	Iron sheet enclosure a	Yes	Flush to Sewer system
	12	Iron sheet enclosure a	Yes	Pit latrine with concrete slab
	18	A brick/stone wall and	Yes	Flush to Sewer system
	48	Iron sheet enclosure a	Yes	Flush to Sewer system
1	20	None	No	
	5	Iron sheet enclosure a	Yes	Pit latrine with concrete slab
	18	A brick/stone wall and	Yes	Flush to septic tank
	7	Iron sheet enclosure a	Yes	Flush to septic tank
	28	Iron sheet enclosure a	Yes	Flush to Sewer system
	3	None	Yes	Pit latrine with concrete slab
0	1	Wire/timber/mud/stone	Yes	Pit latrine with concrete slab
1	15	Iron sheet enclosure a	Yes	Pit latrine with concrete slab
	4	None	Yes	Flush to septic tank
	10	Iron sheet enclosure a	Yes	Pit latrine with concrete slab
	13	A brick/stone wall	Yes	Pit latrine with concrete slab
	4	None	Yes	Pit latrine with concrete slab
	1	None	Yes	Flush to septic tank
1	6	Other (spe Metallic Ga	Yes	Pit latrine with concrete slab
	3	None	Yes	Pit latrine with concrete slab
	5	Iron sheet enclosure a	Yes	Pit latrine with concrete slab

	1	A brick/stone wall and	Yes	Flush to Sewer system
	7	Iron sheet enclosure a	Yes	Flush to septic tank
	1	None	Yes	Pit latrine with concrete slab
1	4	Iron sheet enclosure a	Yes	Pit latrine with concrete slab
	14	None	No	
	5	A brick/stone wall	Yes	Flush to septic tank
	27	A brick/stone wall and	Yes	Flush to septic tank
	15	A brick/stone wall and	Yes	Flush to Sewer system
	4	Iron sheet enclosure a	Yes	Pit latrine with concrete slab
	4	A brick/stone wall and	Yes	Pit latrine with concrete slab
0	3	A brick/stone wall and	Yes	Pit latrine with concrete slab
	12	Iron sheet enclosure a	No	
	1	A brick/stone wall and	Yes	Flush to Sewer system
	15	Iron sheet enclosure a	Yes	Flush to Sewer system
	19	A brick/stone wall and	Yes	Pit latrine with concrete slab
0	10	None	Yes	Flush to Sewer system
0	1	None	Yes	Flush to Sewer system
	15	None	Yes	Pit latrine with concrete slab
	5	A brick/stone wall and	Yes	Pit latrine with concrete slab
	10	A brick/stone wall and	Yes	Flush to pit latrine
0	2	None	Yes	Pit latrine with concrete slab
	1	None	Yes	Flush to septic tank
	5	None	No	
0	6	None	Yes	Pit latrine with concrete slab
	1	None	No	
	1	None	No	
	1	Iron sheet enclosure a	Yes	Pit latrine with concrete slab
1	3	Iron sheet enclosure a	Yes	Pit latrine with concrete slab
	2	A brick/stone wall and	Yes	Flush to pit latrine
	2	None	Yes	Flush to pit latrine
0	7	None	Yes	Pit latrine with concrete slab
	1	None	Yes	Flush to septic tank
1	6	Other (spe Metallic ga	Yes	Pit latrine with concrete slab
	2	A brick/stone wall	Yes	Flush to septic tank
	2	A brick/stone wall	Yes	Pit latrine with concrete slab
	5	A brick/stone wall and	Yes	Flush to Sewer system
	45	Iron sheet enclosure a	Yes	Flush to Sewer system
	2	None	Yes	Pit latrine with concrete slab
	38	Iron sheet enclosure a	Yes	Flush to Sewer system
0	7	None	Yes	Pit latrine with concrete slab
	6	A brick/stone wall and	Yes	Flush to septic tank
	18	A brick/stone wall and	Yes	Flush to Sewer system
	3	A brick/stone wall and	Yes	Flush to pit latrine
	15	Iron sheet enclosure a	Yes	Flush to Sewer system
	1	None	Yes	Pit latrine with concrete slab
	8	A brick/stone wall and	Yes	Pit latrine with concrete slab
	21	A brick/stone wall and	Yes	Flush to Sewer system
	8	A brick/stone wall and	Yes	Flush to Sewer system
	20	None	Yes	Pit latrine with concrete slab

	23	Iron sheet enclosure a	Yes	Flush to Sewer system
	9	A brick/stone wall and	Yes	Flush to Sewer system
0	48	None	Yes	Pit latrine with concrete slab
0	3	A brick/stone wall and	Yes	Flush to septic tank
	1	None	No	
	14	None	Yes	Flush to septic tank
	5	None	Yes	Flush to pit latrine
	6	A brick/stone wall and	Yes	Flush to septic tank
	11	None	Yes	Pit latrine with concrete slab
	27	A brick/stone wall and	Yes	Flush to pit latrine
	3	A brick/stone wall and	Yes	Flush to septic tank
	10	Iron sheet enclosure a	Yes	Flush to Sewer system
	9	Iron sheet enclosure a	Yes	Pit latrine with concrete slab
	14	Iron sheet enclosure a	No	
0	12	A brick/stone wall and	Yes	Pit latrine with concrete slab
0	4	Other (spe Metallic ga	Yes	Pit latrine with concrete slab
	9	None	Yes	Pit latrine with concrete slab
1	15	None	No	
	6	None	Yes	Flush to pit latrine
	8	Iron sheet enclosure a	Yes	Pit latrine with concrete slab
	1	None	Yes	Pit latrine with concrete slab
	18	A brick/stone wall and	Yes	Flush to septic tank
	24	A brick/stone wall and	Yes	Flush to Sewer system
	18	A brick/stone wall and	Yes	Flush to Sewer system
	15	A brick/stone wall and	Yes	Flush to Sewer system
	10	A brick/stone wall and	Yes	Flush to septic tank
	25	A brick/stone wall and	Yes	Flush to Sewer system
	5	None	Yes	Pit latrine with concrete slab
	50	Iron sheet enclosure a	Yes	Pit latrine with concrete slab
	10	None	Yes	Pit latrine with concrete slab
1	11	Iron sheet enclosure a	Yes	Pit latrine with concrete slab
	5	A brick/stone wall and	Yes	Pit latrine with concrete slab
	1	None	Yes	Pit latrine without slab
	6	A brick/stone wall and	No	
	39	A brick/stone wall and	Yes	Flush to septic tank
	12	A brick/stone wall and	Yes	Flush to septic tank
1	8	A brick/stone wall and	Yes	Pit latrine with concrete slab
	23	Iron sheet enclosure a	Yes	Flush to septic tank
	23	Iron sheet enclosure a	Yes	Flush to Sewer system
1	5	Wire/timber/mud/stone	Yes	Pit latrine with concrete slab
	35	A brick/stone wall and	Yes	Flush to septic tank
	1	A brick/stone wall and	Yes	Pit latrine with concrete slab
	38	A brick/stone wall and	Yes	Flush to pit latrine
	12	A brick/stone wall and	Yes	Flush to Sewer system
1	5	Other (spe Mettalic Ga	Yes	Pit latrine with concrete slab
0	8	None	Yes	Pit latrine with concrete slab
	12	A brick/stone wall and	Yes	Flush to pit latrine
	1	A brick/stone wall	Yes	Flush to septic tank
1	8	A brick/stone wall and	Yes	Flush to septic tank

1	42	Iron sheet enclosure a	Yes	Flush to septic tank	
	11	Iron sheet enclosure a	Yes	Flush to septic tank	
	1	A brick/stone wall and	Yes	Flush to septic tank	
0	6	None	Yes	Pit latrine with concrete slab	
1	15	Iron sheet enclosure a	Yes	Pit latrine without slab	
	12	None	Yes	Pit latrine with concrete slab	
	91	A brick/stone wall and	Yes	Flush to pit latrine	
	20	A brick/stone wall and	Yes	Flush donâ€™t know where	
1	10	Iron sheet enclosure a	Yes	Pit latrine with concrete slab	
	40	A brick/stone wall and	Yes	Flush to Sewer system	
	14	A brick/stone wall and	Yes	Pit latrine with concrete slab	
0	18	Iron sheet enclosure a	Yes	Pit latrine with concrete slab	
	13	A brick/stone wall and	Yes	Flush to septic tank	
0	12	A brick/stone wall and	Yes	Flush to septic tank	
0	7	None	No		
	4	A brick/stone wall and	Yes	Pit latrine with concrete slab	
	19	Iron sheet enclosure a	Yes	Flush to pit latrine	
	2	None	Yes	Pit latrine with concrete slab	
	10	None	Yes	Flush to pit latrine	
	10	Iron sheet enclosure a	Yes	Flush to pit latrine	
	10	Iron sheet enclosure a	Yes	Pit latrine with concrete slab	
0	5	Iron sheet enclosure a	Yes	Pit latrine with concrete slab	
	1	None	Yes	Flush to septic tank	
	8	A brick/stone wall and	Yes	Flush to Sewer system	
	4	A brick/stone wall and	Yes	Flush to Sewer system	
	24	Iron sheet enclosure a	Yes	Flush to Sewer system	
	1	None	Yes	Flush to septic tank	
	110	Iron sheet enclosure a	Yes	Flush to Sewer system	
	13	A brick/stone wall and	Yes	Flush to Sewer system	
	9	A brick/stone wall and	Yes	Flush to Sewer system	
	43	Iron sheet enclosure a	Yes	Flush to Sewer system	
	11	A brick/stone wall and	Yes	Flush to Sewer system	
	1	A brick/stone wall and	Yes	Flush to Sewer system	
1	49	Iron sheet enclosure a	Yes	Other (Spe	Flush/pour toilet to a drainage tre
	1	Wire/timber/mud/stone	Yes	Flush to pit latrine	
	3	Iron sheet enclosure a	No		
	11	A brick/stone wall and	Yes	Ventilated improved pit latrine	
	22	A brick/stone wall and	Yes	Pit latrine with concrete slab	
1	1	None	Yes	Flush to septic tank	
	10	Iron sheet enclosure a	Yes	Pit latrine with concrete slab	
0	16	A brick/stone wall and	Yes	Flush to septic tank	
	1	A brick/stone wall and	Yes	Flush to septic tank	
0	12	Iron sheet enclosure a	Yes	Pit latrine with concrete slab	
	5	A brick/stone wall and	Yes	Flush to septic tank	
	60	Iron sheet enclosure a	Yes	Flush to Sewer system	
	4	Iron sheet enclosure a	Yes	Flush donâ€™t know where	
	4	Iron sheet enclosure a	Yes	Pit latrine with concrete slab	
0	6	A brick/stone wall and	Yes	Pit latrine with concrete slab	
	11	A brick/stone wall and	Yes	Flush to Sewer system	

1	20	Iron sheet enclosure a	Yes	Flush to Sewer system
	50	A brick/stone wall and	Yes	Flush to septic tank
	19	Iron sheet enclosure a	Yes	Pit latrine with concrete slab
1	28	A brick/stone wall and	Yes	Flush to septic tank
1	1	None	Yes	Flush to septic tank
0	2	Wire/timber/mud/stone	Yes	Pit latrine without slab
	11	A brick/stone wall and	Yes	Pit latrine with concrete slab
1	9	Iron sheet enclosure a	Yes	Pit latrine with concrete slab
	20	A brick/stone wall and	Yes	Pit latrine with concrete slab
	3	Iron sheet enclosure a	Yes	Pit latrine with concrete slab
	3	A brick/stone wall and	Yes	Flush to septic tank
1	4	None	No	
	6	Iron sheet enclosure a	Yes	Pit latrine without slab
	12	A brick/stone wall and	Yes	Pit latrine with concrete slab
1	7	Iron sheet enclosure a	No	
0	6	None	Yes	Pit latrine with concrete slab
	9	A brick/stone wall and	Yes	Flush to septic tank
	8	None	Yes	Flush to septic tank
1	36	None	Yes	Flush to Sewer system
	7	A brick/stone wall and	Yes	Pit latrine with concrete slab
	10	A brick/stone wall and	No	
	28	A brick/stone wall and	Yes	Pit latrine with concrete slab
	8	Iron sheet enclosure a	Yes	Pit latrine with concrete slab
	4	Iron sheet enclosure a	Yes	Pit latrine with concrete slab
	11	A brick/stone wall and	Yes	Flush to Sewer system
	10	A brick/stone wall and	Yes	Flush to septic tank
	28	A brick/stone wall and	Yes	Flush to pit latrine
	1	Iron sheet enclosure a	Yes	Flush to pit latrine
	12	Iron sheet enclosure a	Yes	Flush to pit latrine
	13	Iron sheet enclosure a	Yes	Pit latrine with concrete slab
	3	None	Yes	Pit latrine with concrete slab
	5	None	Yes	Flush to pit latrine
	31	Iron sheet enclosure a	Yes	Flush to Sewer system
	6	Iron sheet enclosure a	Yes	Pit latrine with concrete slab
	2	A brick/stone wall	Yes	Flush to pit latrine
	1	A brick/stone wall and	Yes	Flush to septic tank
	16	Iron sheet enclosure a	Yes	Pit latrine with concrete slab
	18	A brick/stone wall and	Yes	Pit latrine with concrete slab
	63	Iron sheet enclosure a	Yes	Flush to Sewer system
	9	Iron sheet enclosure a	Yes	Pit latrine with concrete slab
	8	A brick/stone wall and	Yes	Flush to pit latrine
0	1	None	Yes	Pit latrine without slab
1	20	None	Yes	Pit latrine with concrete slab
	5	A brick/stone wall	Yes	Pit latrine with concrete slab
	27	A brick/stone wall and	Yes	Flush to Sewer system
	4	A brick/stone wall	Yes	Flush to septic tank
	25	Iron sheet enclosure a	Yes	Pit latrine with concrete slab
	13	A brick/stone wall and	Yes	Pit latrine with concrete slab
	8	A brick/stone wall and	Yes	Flush to septic tank

	1	None	Yes	Pit latrine without slab	
	5	None	Yes	Pit latrine with concrete slab	
	35	Iron sheet enclosure a	No		
0	12	Iron sheet enclosure a	Yes	Flush to Sewer system	
	25	A brick/stone wall and	Yes	Pit latrine with concrete slab	
	2	Iron sheet enclosure a	Yes	Pit latrine without slab	
	7	A brick/stone wall and	Yes	Flush to septic tank	
	66	Iron sheet enclosure a	Yes	Flush to Sewer system	
	20	A brick/stone wall and	Yes	Flush to Sewer system	
	8	Wire/timber/mud/stone	Yes	Pit latrine with concrete slab	
	33	A brick/stone wall and	Yes	Flush to septic tank	
	5	A brick/stone wall and	Yes	Pit latrine with concrete slab	
	60	Iron sheet enclosure a	Yes	Flush to Sewer system	
	2	A brick/stone wall and	Yes	Other (Spe	Pit latrine withvtiled floor and vent
	6	A brick/stone wall and	Yes	Flush donâ€™t know where	
	36	A brick/stone wall and	Yes	Flush to Sewer system	
	24	Iron sheet enclosure a	Yes	Flush to septic tank	
	5	Iron sheet enclosure a	Yes	Pit latrine with concrete slab	
	5	A brick/stone wall and	Yes	Pit latrine with concrete slab	
0	14	None	Yes	Pit latrine with concrete slab	
1	10	None	Yes	Pit latrine with concrete slab	
	28	Iron sheet enclosure a	Yes	Flush to Sewer system	
	3	A brick/stone wall and	Yes	Flush to Sewer system	
	10	Iron sheet enclosure a	Yes	Pit latrine with concrete slab	
	20	Iron sheet enclosure a	Yes	Pit latrine with concrete slab	
	4	None	Yes	Pit latrine with concrete slab	
1	1	None	Yes	Flush to septic tank	
	9	A brick/stone wall and	Yes	Flush to septic tank	
	22	A brick/stone wall and	Yes	Flush to pit latrine	
	8	None	Yes	Flush to Sewer system	
	8	None	Yes	Pit latrine with concrete slab	
	22	A brick/stone wall and	Yes	Flush to pit latrine	
	40	None	Yes	Flush to Sewer system	
	1	None	Yes	Flush to pit latrine	
0	12	None	Yes	Pit latrine with concrete slab	
	10	Iron sheet enclosure a	Yes	Pit latrine with concrete slab	
	26	A brick/stone wall and	No		
	50	A brick/stone wall and	Yes	Pit latrine with concrete slab	
1	6	None	Yes	Pit latrine with concrete slab	
	7	A brick/stone wall and	Yes	Flush to Sewer system	
	8	A brick/stone wall and	Yes	Pit latrine with concrete slab	
	20	Wire/timber/mud/stone	Yes	Flush to septic tank	
	1	None	Yes	Pit latrine with concrete slab	
	18	A brick/stone wall and	Yes	Flush to Sewer system	
	41	Iron sheet enclosure a	Yes	Other (Spe	Not within the compound
0	10	A brick/stone wall and	Yes	Flush to pit latrine	
	1	None	Yes	Pit latrine with concrete slab	
	5	None	Yes	Pit latrine with concrete slab	
1	5	A brick/stone wall and	Yes	Flush to septic tank	

	28	Iron sheet enclosure a	Yes	Flush to Sewer system
	4	Iron sheet enclosure a	No	
	34	Wire/timber/mud/stone	Yes	Flush to Sewer system
	13	Iron sheet enclosure a	Yes	Flush to septic tank
	11	Iron sheet enclosure a	Yes	Pit latrine with concrete slab
1	7	Wire/timber/mud/stone	Yes	Pit latrine with concrete slab
	44	Iron sheet enclosure a	Yes	Flush to pit latrine
1	2	Iron sheet enclosure a	Yes	Pit latrine with concrete slab
	18	A brick/stone wall and	Yes	Flush to septic tank
	30	Iron sheet enclosure a	Yes	Pit latrine without slab
0	2	Iron sheet enclosure a	Yes	Pit latrine with concrete slab
	66	Iron sheet enclosure a	Yes	Flush to Sewer system
	1	None	Yes	Flush to pit latrine
	37	A brick/stone wall and	Yes	Flush to Sewer system
	10	Iron sheet enclosure a	Yes	Flush to Sewer system
	10	A brick/stone wall and	Yes	Pit latrine with concrete slab
	5	A brick/stone wall and	Yes	Pit latrine with concrete slab
1	25	None	Yes	Pit latrine with concrete slab
	17	Iron sheet enclosure a	Yes	Flush to septic tank
	1	None	Yes	Flush to septic tank
	36	Iron sheet enclosure a	No	
	1	A brick/stone wall and	Yes	Flush to septic tank
	9	Iron sheet enclosure a	Yes	Flush to septic tank
	9	Iron sheet enclosure a	Yes	Pit latrine with concrete slab
	16	Iron sheet enclosure a	Yes	Flush to Sewer system
	40	A brick/stone wall and	Yes	Flush to Sewer system
	1	Iron sheet enclosure a	Yes	Pit latrine with concrete slab
	8	A brick/stone wall and	Yes	Flush to pit latrine
	28	A brick/stone wall and	Yes	Flush to Sewer system
	8	Iron sheet enclosure a	Yes	Flush to Sewer system
	18	A brick/stone wall	Yes	Flush to septic tank
	4	None	Yes	Pit latrine with concrete slab
1	4	None	Yes	Pit latrine without slab
	1	None	Yes	Flush to septic tank
	14	A brick/stone wall and	Yes	Flush to septic tank
	12	Iron sheet enclosure a	Yes	Flush to pit latrine
0	3	None	Yes	Pit latrine with concrete slab
	10	A brick/stone wall and	Yes	Pit latrine with concrete slab
	11	Iron sheet enclosure a	Yes	Flush to pit latrine
	20	A brick/stone wall and	Yes	Flush to Sewer system
	11	None	Yes	Pit latrine with concrete slab
1	37	A brick/stone wall and	Yes	Flush to septic tank
	3	A brick/stone wall and	Yes	Flush to septic tank
	6	A brick/stone wall and	Yes	Flush to septic tank
	26	Iron sheet enclosure a	Yes	Flush to Sewer system
	12	Iron sheet enclosure a	Yes	Pit latrine with concrete slab
	1	None	Yes	Flush to septic tank
	20	Iron sheet enclosure a	No	
1	8	Iron sheet enclosure a	No	

	8	None	Yes	Pit latrine with concrete slab
	1	None	Yes	Pit latrine with concrete slab
	13	Iron sheet enclosure a	Yes	Pit latrine with concrete slab
	30	Iron sheet enclosure a	Yes	Flush to septic tank
	33	Iron sheet enclosure a	Yes	Flush to Sewer system
0	4	Iron sheet enclosure a	Yes	Pit latrine with concrete slab
	4	A brick/stone wall and	Yes	Pit latrine with concrete slab
	24	Iron sheet enclosure a	Yes	Flush to septic tank
1	11	None	Yes	Pit latrine with concrete slab
	60	A brick/stone wall and	Yes	Flush to onsite, open pit
	6	None	Yes	Flush to pit latrine
	8	A brick/stone wall and	Yes	Flush to pit latrine
	19	Iron sheet enclosure a	Yes	Pit latrine with concrete slab
	12	Iron sheet enclosure a	Yes	Flush to septic tank
1	2	Iron sheet enclosure a	Yes	Pit latrine with concrete slab
1	3	None	Yes	Pit latrine with concrete slab
	1	None	Yes	Flush to septic tank
	1	A brick/stone wall and	Yes	Pit latrine with concrete slab
	3	Iron sheet enclosure a	Yes	Pit latrine with concrete slab
	14	A brick/stone wall and	Yes	Flush to Sewer system
	7	Wire/timber/mud/stone	Yes	Pit latrine with concrete slab
	35	A brick/stone wall and	Yes	Flush to Sewer system
1	1	Iron sheet enclosure a	No	
	1	A brick/stone wall and	Yes	Flush to septic tank
	2	A brick/stone wall and	Yes	Flush to septic tank
	9	Wire/timber/mud/stone	Yes	Pit latrine with concrete slab
	15	Iron sheet enclosure a	Yes	No facility/bush/field
1	5	Iron sheet enclosure a	Yes	Pit latrine with concrete slab
	21	Iron sheet enclosure a	Yes	Flush to septic tank
	3	Iron sheet enclosure a	Yes	Pit latrine with concrete slab
0	7	Iron sheet enclosure a	Yes	Pit latrine with concrete slab
0	13	Iron sheet enclosure a	Yes	Flush to septic tank
	12	Iron sheet enclosure a	Yes	Flush to pit latrine
	8	A brick/stone wall and	Yes	Flush to Sewer system
	9	Iron sheet enclosure a	Yes	Pit latrine with concrete slab
	15	A brick/stone wall and	Yes	Flush to Sewer system
	8	A brick/stone wall and	Yes	Pit latrine with concrete slab
	40	Iron sheet enclosure a	Yes	Flush to Sewer system
	10	A brick/stone wall	Yes	Pit latrine with concrete slab
	6	None	No	
0	86	A brick/stone wall and	Yes	Flush to pit latrine
	10	A brick/stone wall	Yes	Pit latrine with concrete slab
1	4	Iron sheet enclosure a	Yes	Pit latrine with concrete slab
	21	Iron sheet enclosure a	Yes	Flush to Sewer system
	3	None	Yes	Pit latrine with concrete slab
	6	None	Yes	Pit latrine with concrete slab
	4	A brick/stone wall	Yes	Flush to Sewer system
	3	Iron sheet enclosure a	Yes	Pit latrine with concrete slab
	10	Iron sheet enclosure a	Yes	Pit latrine with concrete slab

	4	Iron sheet enclosure a	Yes	Pit latrine without slab
	48	A brick/stone wall and	Yes	Flush to Sewer system
0	3	None	Yes	Pit latrine with concrete slab
	14	Iron sheet enclosure a	Yes	Flush to septic tank
	2	Iron sheet enclosure a	Yes	Flush to pit latrine
	2	Iron sheet enclosure a	Yes	Flush to pit latrine
	18	Iron sheet enclosure a	Yes	Flush to Sewer system
	2	A brick/stone wall and	Yes	Flush to pit latrine
0	2	A brick/stone wall	Yes	Pit latrine with concrete slab
	8	Iron sheet enclosure a	Yes	Pit latrine with concrete slab
	16	Iron sheet enclosure a	Yes	Flush to Sewer system
	23	A brick/stone wall and	Yes	Flush to Sewer system
	43	Iron sheet enclosure a	Yes	Flush to septic tank
	1	None	Yes	Flush to septic tank
	11	Iron sheet enclosure a	Yes	Pit latrine with concrete slab
	11	A brick/stone wall and	Yes	Pit latrine with concrete slab
	22	A brick/stone wall and	Yes	Flush to Sewer system
	6	None	No	
	10	A brick/stone wall and	Yes	Pit latrine with concrete slab
	30	A brick/stone wall and	Yes	Flush to Sewer system
	5	Iron sheet enclosure a	Yes	Pit latrine with concrete slab
	4	None	Yes	Flush to Sewer system
	7	A brick/stone wall and	Yes	Flush to septic tank
	13	A brick/stone wall and	Yes	Pit latrine with concrete slab
	8	None	Yes	Pit latrine with concrete slab
	9	Iron sheet enclosure a	Yes	Pit latrine with concrete slab
	11	A brick/stone wall and	Yes	Flush to septic tank
	4	None	Yes	Flush to pit latrine
	10	None	Yes	Flush to septic tank
	5	A brick/stone wall and	Yes	Flush to septic tank
	78	Iron sheet enclosure a	Yes	Flush to Sewer system
	1	None	Yes	Flush to Sewer system
	37	A brick/stone wall and	Yes	Flush to pit latrine
	20	Iron sheet enclosure a	Yes	Flush to Sewer system
	1	None	Yes	Flush to pit latrine
0	20	Iron sheet enclosure a	Yes	Pit latrine with concrete slab
	3	Wire/timber/mud/stone	Yes	Pit latrine without slab
	4	Iron sheet enclosure a	Yes	Pit latrine with concrete slab
	22	None	Yes	Pit latrine with concrete slab
	5	A brick/stone wall and	Yes	Flush to Sewer system
	1	None	Yes	Flush to septic tank
0	9	A brick/stone wall	Yes	Pit latrine with concrete slab
	4	A brick/stone wall and	Yes	Flush to septic tank
	12	None	Yes	Flush to septic tank
	1	Iron sheet enclosure a	Yes	Flush to Sewer system
1	10	None	Yes	Pit latrine with concrete slab
	30	Iron sheet enclosure a	Yes	Flush to Sewer system
1	10	Other (spe Metallic Ga	Yes	Pit latrine with concrete slab
	30	Wire/timber/mud/stone	Yes	Flush to Sewer system

	4	Iron sheet enclosure a	Yes	Flush to Sewer system	
	1	None	Yes	Flush to septic tank	
	20	None	Yes	Flush to pit latrine	
	1	A brick/stone wall and	Yes	Flush to septic tank	
1	1	None	Yes	Flush to septic tank	
0	27	Iron sheet enclosure a	Yes	Other (Spe	The toilet is locked and theres nc
	1	None	Yes	Flush to septic tank	
	6	A brick/stone wall	Yes	Flush donâ€™t know where	
	10	A brick/stone wall and	Yes	Flush to septic tank	
1	2	Iron sheet enclosure a	Yes	Pit latrine without slab	
	32	Iron sheet enclosure a	Yes	Flush to septic tank	
	6	Iron sheet enclosure a	Yes	Pit latrine with concrete slab	
	9	Iron sheet enclosure a	Yes	Flush to Sewer system	
	10	None	Yes	Pit latrine with concrete slab	
	20	Iron sheet enclosure a	Yes	Flush to septic tank	
1	9	Iron sheet enclosure a	Yes	Pit latrine with concrete slab	
	1	None	Yes	Pit latrine with concrete slab	
	10	None	Yes	Pit latrine without slab	
	8	A brick/stone wall and	Yes	Flush to pit latrine	
1	1	Iron sheet enclosure a	Yes	Pit latrine with concrete slab	
	21	Iron sheet enclosure a	Yes	Flush to Sewer system	
	10	Iron sheet enclosure a	Yes	Pit latrine with concrete slab	
	40	A brick/stone wall and	Yes	Flush to septic tank	
	1	None	Yes	Pit latrine with concrete slab	
	5	Iron sheet enclosure a	No		
	22	A brick/stone wall and	Yes	Flush to Sewer system	
	32	None	No		
	21	A brick/stone wall and	Yes	Pit latrine with concrete slab	
0	5	A brick/stone wall	Yes	Pit latrine with concrete slab	
0	9	Iron sheet enclosure a	Yes	Pit latrine with concrete slab	
0	14	None	Yes	Pit latrine with concrete slab	
	2	Iron sheet enclosure a	Yes	Pit latrine with concrete slab	
	6	A brick/stone wall and	Yes	Flush to pit latrine	
1	4	None	Yes	Pit latrine with concrete slab	
	11	Iron sheet enclosure a	Yes	Pit latrine with concrete slab	
	13	Iron sheet enclosure a	Yes	Pit latrine with concrete slab	
1	1	None	Yes	Flush to septic tank	
	7	A brick/stone wall and	Yes	Flush to septic tank	
	8	None	Yes	Pit latrine with concrete slab	
	9	A brick/stone wall and	Yes	Other (Spe	Respondent didn't allow me to obs
0	15	A brick/stone wall and	Yes	Flush to pit latrine	
	4	A brick/stone wall and	Yes	Pit latrine with concrete slab	
	6	Iron sheet enclosure a	Yes	Pit latrine with concrete slab	
	10	Iron sheet enclosure a	No		
0	18	None	Yes	Pit latrine with concrete slab	
	7	A brick/stone wall and	Yes	Pit latrine with concrete slab	
	1	None	Yes	Flush to septic tank	
	35	A brick/stone wall and	Yes	Flush to Sewer system	
	8	Iron sheet enclosure a	No		

	7	A brick/stone wall and	Yes	Pit latrine with concrete slab
1	30	Iron sheet enclosure a	Yes	Pit latrine with concrete slab
	1	None	Yes	Flush to septic tank
0	21	A brick/stone wall and	Yes	Flush to septic tank
	4	Iron sheet enclosure a	Yes	Pit latrine with concrete slab
	24	Iron sheet enclosure a	Yes	Flush to Sewer system
	7	A brick/stone wall and	Yes	Flush to septic tank
	18	Iron sheet enclosure a	Yes	Flush to Sewer system
	1	A brick/stone wall and	Yes	Flush to septic tank
0	11	Wire/timber/mud/stone	Yes	Pit latrine with concrete slab
	6	A brick/stone wall and	Yes	Flush to septic tank
	8	Iron sheet enclosure a	Yes	Pit latrine with concrete slab
	8	Iron sheet enclosure a	Yes	Pit latrine with concrete slab
	1	Wire/timber/mud/stone	Yes	Flush to pit latrine
	6	None	Yes	Pit latrine with concrete slab
1	7	Iron sheet enclosure a	Yes	Pit latrine with concrete slab
	3	Iron sheet enclosure a	Yes	Flush to Sewer system
0	9	A brick/stone wall and	Yes	Pit latrine with concrete slab
	1	None	Yes	Pit latrine with concrete slab
	8	A brick/stone wall and	Yes	Pit latrine with concrete slab
	6	Iron sheet enclosure a	Yes	Pit latrine with concrete slab
	1	Wire/timber/mud/stone	No	
0	18	None	Yes	Pit latrine with concrete slab
	8	A brick/stone wall and	Yes	Flush to septic tank
	1	None	Yes	Flush to pit latrine
1	6	Iron sheet enclosure a	Yes	Pit latrine with concrete slab
	6	None	Yes	Pit latrine with concrete slab
	8	A brick/stone wall and	Yes	Flush to septic tank
	60	Iron sheet enclosure a	Yes	Flush to Sewer system
	4	A brick/stone wall and	Yes	Pit latrine with concrete slab
	1	None	Yes	Flush to septic tank
1	13	Iron sheet enclosure a	Yes	Flush to septic tank
	10	Iron sheet enclosure a	Yes	Pit latrine with concrete slab
	4	A brick/stone wall and	Yes	Pit latrine with concrete slab
0	6	Iron sheet enclosure a	Yes	Pit latrine with concrete slab
	20	Iron sheet enclosure a	Yes	Flush to septic tank
	11	A brick/stone wall	Yes	Pit latrine with concrete slab
	16	Iron sheet enclosure a	Yes	Flush to pit latrine
	50	A brick/stone wall	Yes	Pit latrine with concrete slab
	10	Wire/timber/mud/stone	Yes	Flush to Sewer system
1	10	A brick/stone wall and	Yes	Pit latrine with concrete slab
	2	None	Yes	Pit latrine with concrete slab
	10	A brick/stone wall and	Yes	Pit latrine with concrete slab
	6	Iron sheet enclosure a	Yes	Pit latrine with concrete slab
	4	Iron sheet enclosure a	Yes	Pit latrine with concrete slab
	30	A brick/stone wall and	Yes	Flush to septic tank
1	9	None	Yes	Pit latrine with concrete slab
	72	None	Yes	Flush to Sewer system
	10	A brick/stone wall and	Yes	Flush to Sewer system

1	9	None	Yes	Pit latrine with concrete slab
0	9	Iron sheet enclosure a	Yes	Pit latrine with concrete slab
	4	Iron sheet enclosure a	Yes	Pit latrine with concrete slab
	13	Iron sheet enclosure a	Yes	Pit latrine with concrete slab
1	1	Iron sheet enclosure a	Yes	Pit latrine with concrete slab
	4	None	Yes	Pit latrine with concrete slab
	12	Iron sheet enclosure a	Yes	Flush to Sewer system
1	5	A brick/stone wall and	Yes	Pit latrine with concrete slab
	39	Iron sheet enclosure a	Yes	Flush to Sewer system
	100	Iron sheet enclosure a	Yes	Flush to Sewer system
	1	None	Yes	Flush to septic tank
	9	A brick/stone wall and	No	
	32	Iron sheet enclosure a	Yes	Flush to septic tank
	24	A brick/stone wall and	Yes	Flush to Sewer system
	10	A brick/stone wall and	No	
1	8	None	Yes	Pit latrine with concrete slab
	3	Iron sheet enclosure a	Yes	Pit latrine with concrete slab
0	11	Iron sheet enclosure a	No	
	5	Iron sheet enclosure a	No	
	50	A brick/stone wall and	Yes	Flush to pit latrine
	3	Iron sheet enclosure a	No	
	4	A brick/stone wall and	Yes	Flush to septic tank
	13	Iron sheet enclosure a	Yes	Flush to Sewer system
	25	None	Yes	Pit latrine with concrete slab
	20	A brick/stone wall and	Yes	Flush to Sewer system
	20	Wire/timber/mud/stone	Yes	Flush to septic tank
	58	Iron sheet enclosure a	Yes	Flush to Sewer system
	11	Iron sheet enclosure a	Yes	Pit latrine with concrete slab
	9	A brick/stone wall and	No	
	16	None	Yes	Flush to septic tank
	6	A brick/stone wall and	Yes	Flush to septic tank
	25	A brick/stone wall and	Yes	Flush to septic tank
	17	A brick/stone wall and	Yes	Pit latrine with concrete slab
	6	Iron sheet enclosure a	Yes	Flush to pit latrine
	10	Iron sheet enclosure a	Yes	Pit latrine with concrete slab
	13	None	Yes	Flush to septic tank
	30	A brick/stone wall and	Yes	Flush to septic tank
	15	Iron sheet enclosure a	Yes	Ventilated improved pit latrine
0	10	None	Yes	Pit latrine with concrete slab
	1	None	Yes	Flush to pit latrine
	6	Iron sheet enclosure a	Yes	Flush to septic tank
	4	A brick/stone wall and	Yes	Flush to septic tank
	4	A brick/stone wall and	Yes	Flush to septic tank
	13	A brick/stone wall and	Yes	Pit latrine with concrete slab
	17	Iron sheet enclosure a	Yes	Flush to septic tank
	1	Iron sheet enclosure a	Yes	Pit latrine with concrete slab
	1	A brick/stone wall and	Yes	Flush to septic tank
	23	A brick/stone wall and	Yes	Flush to septic tank
	18	A brick/stone wall and	Yes	Flush to Sewer system

	17	A brick/stone wall and	Yes	Flush to Sewer system
	28	Iron sheet enclosure a	Yes	Flush donâ€™t know where
0	27	A brick/stone wall and	Yes	Flush to septic tank
	18	Iron sheet enclosure a	Yes	Pit latrine with concrete slab
	35	A brick/stone wall and	Yes	Flush to Sewer system
	7	Iron sheet enclosure a	Yes	Pit latrine with concrete slab
	2	Iron sheet enclosure a	Yes	Pit latrine with concrete slab
	13	A brick/stone wall and	Yes	Flush to Sewer system
1	2	A brick/stone wall and	Yes	Pit latrine with concrete slab
1	8	None	Yes	Pit latrine without slab
	5	None	Yes	Pit latrine with concrete slab
1	3	Iron sheet enclosure a	No	
	6	A brick/stone wall and	Yes	Flush to septic tank
	8	A brick/stone wall and	Yes	Flush to pit latrine
	12	Iron sheet enclosure a	Yes	Flush to septic tank
0	7	Iron sheet enclosure a	Yes	Pit latrine with concrete slab
	13	Iron sheet enclosure a	Yes	Pit latrine with concrete slab
	10	A brick/stone wall and	Yes	Pit latrine with concrete slab
0	12	A brick/stone wall and	Yes	Flush to septic tank
	1	None	Yes	Flush to septic tank
	28	A brick/stone wall	Yes	Flush to Sewer system
	6	A brick/stone wall and	Yes	Flush to septic tank
	50	A brick/stone wall and	No	
0	3	Iron sheet enclosure a	Yes	Flush to Sewer system
	20	A brick/stone wall and	Yes	Flush to Sewer system
	2	A brick/stone wall and	Yes	Flush to septic tank
	82	A brick/stone wall and	No	
	7	None	Yes	Flush to Sewer system
	52	Iron sheet enclosure a	Yes	Flush to Sewer system
0	1	None	Yes	Flush to septic tank
	10	Wire/timber/mud/stone	Yes	Pit latrine with concrete slab
	21	Iron sheet enclosure a	Yes	Flush to Sewer system
	3	None	Yes	Flush to septic tank
1	4	Other (spe Metallic Ga	Yes	Pit latrine with concrete slab
	1	None	No	
	6	A brick/stone wall and	Yes	Flush donâ€™t know where
	4	Iron sheet enclosure a	Yes	Pit latrine with concrete slab
	7	A brick/stone wall and	Yes	Flush to pit latrine
	1	None	Yes	Pit latrine with concrete slab
	2	A brick/stone wall and	No	
1	6	Wire/timber/mud/stone	Yes	Pit latrine with concrete slab
	17	None	Yes	Flush to septic tank
0	13	A brick/stone wall and	Yes	Pit latrine with concrete slab
	26	Iron sheet enclosure a	Yes	Pit latrine with concrete slab
	10	A brick/stone wall and	Yes	Flush to septic tank
	23	A brick/stone wall and	Yes	Flush to Sewer system
	19	Iron sheet enclosure a	Yes	Flush to Sewer system
	4	A brick/stone wall and	Yes	Pit latrine with concrete slab
	10	Wire/timber/mud/stone	Yes	Pit latrine with concrete slab

	1	None	Yes	Flush to septic tank
0	72	Iron sheet enclosure a	Yes	Flush to Sewer system
	15	A brick/stone wall and	Yes	Flush to Sewer system
	13	Iron sheet enclosure a	Yes	Flush to Sewer system
	1	None	Yes	Pit latrine without slab
	1	None	Yes	Flush to septic tank
1	9	None	Yes	Pit latrine with concrete slab
	4	A brick/stone wall and	Yes	Pit latrine with concrete slab
0	36	Iron sheet enclosure a	Yes	Pit latrine with concrete slab
	1	Iron sheet enclosure a	Yes	Pit latrine with concrete slab
	9	A brick/stone wall and	Yes	Flush to septic tank
	60	Iron sheet enclosure a	Yes	Flush to Sewer system
	4	A brick/stone wall and	Yes	Flush donâ€™t know where
	28	Iron sheet enclosure a	Yes	Flush to Sewer system
	9	Wire/timber/mud/stone	Yes	Pit latrine with concrete slab
	40	A brick/stone wall and	Yes	Flush to Sewer system
	17	A brick/stone wall and	Yes	Flush to Sewer system
	7	Iron sheet enclosure a	Yes	Flush to Sewer system
	56	None	Yes	Flush donâ€™t know where
	2	Wire/timber/mud/stone	Yes	Pit latrine with concrete slab
	4	Wire/timber/mud/stone	Yes	Pit latrine with concrete slab
	26	Iron sheet enclosure a	Yes	Flush to septic tank
	10	A brick/stone wall and	Yes	Pit latrine with concrete slab
	2	None	No	
	24	A brick/stone wall and	Yes	Flush to Sewer system
	19	A brick/stone wall and	Yes	Flush to septic tank
	21	A brick/stone wall and	Yes	Flush to pit latrine
	13	None	Yes	Pit latrine with concrete slab
	8	A brick/stone wall and	Yes	Flush to pit latrine
	4	Iron sheet enclosure a	No	
	7	None	Yes	Flush to pit latrine
	8	Iron sheet enclosure a	Yes	Flush to septic tank
	54	Iron sheet enclosure a	Yes	Flush to Sewer system
	19	Iron sheet enclosure a	Yes	Flush to Sewer system
	2	None	Yes	Pit latrine without slab
	11	A brick/stone wall and	Yes	Flush to Sewer system
1	16	Iron sheet enclosure a	Yes	Pit latrine with concrete slab
0	1	None	Yes	Flush to pit latrine
	4	None	Yes	Pit latrine with concrete slab
	3	A brick/stone wall and	Yes	Flush to pit latrine
0	2	None	Yes	Pit latrine with concrete slab
0	11	None	Yes	Pit latrine with concrete slab
	8	Iron sheet enclosure a	Yes	Pit latrine with concrete slab
0	1	Iron sheet enclosure a	Yes	Pit latrine with concrete slab
	12	A brick/stone wall and	Yes	Flush to septic tank
	17	A brick/stone wall and	Yes	Flush to septic tank
0	9	A brick/stone wall	Yes	Pit latrine with concrete slab
	3	None	Yes	Pit latrine with concrete slab
	1	None	Yes	Flush to Sewer system

	4	Iron sheet enclosure a	Yes	Pit latrine with concrete slab
	25	A brick/stone wall and	Yes	Flush to septic tank
1	7	Iron sheet enclosure a	Yes	Pit latrine without slab
	1	None	Yes	Flush to septic tank
0	17	Wire/timber/mud/stone	Yes	Flush to pit latrine
	39	Iron sheet enclosure a	Yes	Flush to pit latrine
	6	Iron sheet enclosure a	Yes	Pit latrine with concrete slab
	8	Iron sheet enclosure a	Yes	Flush to Sewer system
	6	A brick/stone wall and	Yes	Pit latrine with concrete slab
	28	A brick/stone wall and	Yes	Pit latrine with concrete slab
	1	A brick/stone wall and	Yes	Bag
	6	A brick/stone wall and	Yes	Flush to septic tank
	11	Iron sheet enclosure a	Yes	Pit latrine with concrete slab
	30	A brick/stone wall	Yes	Pit latrine with concrete slab
1	25	Iron sheet enclosure a	Yes	Pit latrine with concrete slab
	30	Iron sheet enclosure a	Yes	Flush to Sewer system
	10	Iron sheet enclosure a	Yes	Flush to septic tank
1	6	None	Yes	Pit latrine with concrete slab
	20	A brick/stone wall and	Yes	Pit latrine with concrete slab
	4	A brick/stone wall and	Yes	Pit latrine with concrete slab
1	30	Iron sheet enclosure a	Yes	Pit latrine with concrete slab
	19	Iron sheet enclosure a	Yes	Flush to septic tank
	25	A brick/stone wall and	Yes	Pit latrine with concrete slab
	28	Iron sheet enclosure a	Yes	Flush to septic tank
	35	Iron sheet enclosure a	Yes	Flush to Sewer system
	33	Iron sheet enclosure a	Yes	Flush to somewhere else
	21	A brick/stone wall and	Yes	Flush to septic tank
0	10	Iron sheet enclosure a	Yes	Pit latrine with concrete slab
	12	A brick/stone wall and	Yes	Flush to septic tank
1	14	Iron sheet enclosure a	Yes	Flush to pit latrine
	1	None	Yes	Pit latrine with concrete slab
	12	Iron sheet enclosure a	Yes	Flush to septic tank
	17	Iron sheet enclosure a	Yes	Flush to pit latrine
	19	Iron sheet enclosure a	Yes	Pit latrine with concrete slab
	6	None	Yes	Pit latrine without slab
0	12	Iron sheet enclosure a	Yes	Pit latrine with concrete slab
	5	None	Yes	Pit latrine with concrete slab
	22	A brick/stone wall and	No	
1	8	Iron sheet enclosure a	Yes	Pit latrine with concrete slab
	7	Other (spe Part of the	Yes	Pit latrine with concrete slab
0	6	Iron sheet enclosure a	Yes	Pit latrine with concrete slab
	11	A brick/stone wall and	Yes	Flush to pit latrine
	10	A brick/stone wall and	Yes	Flush to septic tank
	10	A brick/stone wall	Yes	Pit latrine with concrete slab
	18	None	Yes	Flush to septic tank
	23	A brick/stone wall and	Yes	Flush to Sewer system
	21	Iron sheet enclosure a	Yes	Flush to Sewer system
0	4	Iron sheet enclosure a	Yes	Flush to pit latrine
	38	A brick/stone wall and	Yes	Flush to Sewer system

	3	A brick/stone wall and	No	
	1	None	Yes	Flush to pit latrine
1	8	None	Yes	Pit latrine with concrete slab
	2	Iron sheet enclosure a	Yes	Flush to septic tank
	10	A brick/stone wall and	Yes	Flush to Sewer system
	6	A brick/stone wall and	Yes	Pit latrine with concrete slab
	15	Iron sheet enclosure a	Yes	Pit latrine with concrete slab
	8	A brick/stone wall and	Yes	Flush to Sewer system
	20	A brick/stone wall	Yes	Flush to Sewer system
	30	Iron sheet enclosure a	Yes	Flush to Sewer system
0	8	None	Yes	Pit latrine with concrete slab
	1	None	No	
	8	Iron sheet enclosure a	Yes	Pit latrine with concrete slab
1	12	Iron sheet enclosure a	Yes	Flush to Sewer system
	38	Iron sheet enclosure a	Yes	Flush to Sewer system
	7	A brick/stone wall and	Yes	Pit latrine with concrete slab
	2	None	Yes	Flush to pit latrine
	14	Iron sheet enclosure a	Yes	Pit latrine with concrete slab
	11	None	Yes	Pit latrine with concrete slab
	3	None	Yes	Flush to pit latrine
	1	None	No	
	26	Iron sheet enclosure a	Yes	Flush to Sewer system
	1	A brick/stone wall and	Yes	Flush to septic tank
	5	A brick/stone wall and	Yes	Flush to Sewer system
0	18	Iron sheet enclosure a	Yes	Flush to Sewer system
0	23	A brick/stone wall and	Yes	Flush to pit latrine
	6	A brick/stone wall and	Yes	Flush to septic tank
	4	Iron sheet enclosure a	Yes	Pit latrine with concrete slab
	3	A brick/stone wall and	Yes	Flush to septic tank
	1	None	No	
	59	None	Yes	Pit latrine without slab
	9	None	Yes	Flush to septic tank
	11	None	Yes	Pit latrine with concrete slab
	6	None	Yes	Flush to pit latrine
	20	Iron sheet enclosure a	Yes	Pit latrine with concrete slab
	8	Iron sheet enclosure a	Yes	Pit latrine with concrete slab
	5	A brick/stone wall	Yes	Flush to septic tank
0	16	Wire/timber/mud/stone	Yes	Flush to pit latrine
	1	A brick/stone wall and	Yes	Flush to septic tank
	21	Iron sheet enclosure a	Yes	Flush to Sewer system
0	10	Iron sheet enclosure a	Yes	Pit latrine with concrete slab
1	20	None	Yes	Pit latrine with concrete slab
	14	A brick/stone wall and	Yes	Flush to septic tank
	11	A brick/stone wall and	Yes	Pit latrine with concrete slab
	42	A brick/stone wall and	Yes	Flush to Sewer system
	50	A brick/stone wall	Yes	Flush to pit latrine
	10	A brick/stone wall	Yes	Flush to pit latrine
	28	Iron sheet enclosure a	Yes	Flush to Sewer system
1	10	None	Yes	Pit latrine without slab

	12	Iron sheet enclosure a	Yes	Pit latrine with concrete slab
	13	A brick/stone wall and	Yes	Flush to Sewer system
	40	None	Yes	Flush to pit latrine
	8	Iron sheet enclosure a	Yes	Pit latrine with concrete slab
	20	Iron sheet enclosure a	Yes	Flush to Sewer system
	42	Iron sheet enclosure a	Yes	Flush to pit latrine
	1	A brick/stone wall and	Yes	Pit latrine with concrete slab
	1	None	Yes	Pit latrine without slab
	16	Iron sheet enclosure a	Yes	Flush to septic tank
	6	None	Yes	Pit latrine with concrete slab
	1	A brick/stone wall and	Yes	Flush to septic tank
	1	None	Yes	Pit latrine with concrete slab
	10	A brick/stone wall and	Yes	Flush to Sewer system
	4	None	Yes	Flush to pit latrine
	1	None	Yes	Pit latrine with concrete slab
1	5	Iron sheet enclosure a	Yes	Pit latrine with concrete slab
	36	Iron sheet enclosure a	Yes	Flush to Sewer system
	6	A brick/stone wall and	Yes	Flush to septic tank
	7	Iron sheet enclosure a	Yes	Flush to pit latrine
1	15	None	Yes	Pit latrine with concrete slab
1	11	None	Yes	Pit latrine with concrete slab
	22	Iron sheet enclosure a	No	
	13	Iron sheet enclosure a	Yes	Flush to Sewer system
	1	None	Yes	Flush to septic tank
	39	Iron sheet enclosure a	Yes	Flush to Sewer system
